# Atomically precise single-atom cobalt photocatalyst for enhanced decarboxylative cross-couplings

**DOI:** 10.1039/d5sc04589d

**Published:** 2025-09-16

**Authors:** Qian Yang, Mengting Wu, Wanlin Wang, Wentao Wang, Han Wang, Yurong Tang, Magnus Rueping, Yunfei Cai

**Affiliations:** a School of Chemistry and Chemical Engineering, Chongqing University 174 Shazheng Street Chongqing 400044 China yf.cai@cqu.edu.cn; b Dalian Institute of Chemical Physics, Chinese Academy of Sciences Dalian 116023 China; c KAUST Catalysis Center (KCC), King Abdullah University of Science and Technology (KAUST) Thuwal, 23955-6900 Saudi Arabia

## Abstract

The development of single-atom photocatalysts (SAPs) with superior activity, selectivity and reusability is a promising yet challenging strategy for advancing heterogeneous metallaphotocatalytic organic transformations in sustainable chemical synthesis. In this study, we report the controllable fabrication and application of novel cobalt-based SAPs, a class of atomically dispersed Co/photo bifunctional heterogeneous catalysts, for highly efficient decarboxylative Heck-type coupling reactions of carboxylic acids with olefins. The optimal SAP (Co_SA_–K-PHI, containing 1 wt% Co and 11 wt% K), supported on ionic carbon nitride, can be facilely prepared *via* a mild and straightforward Co/K exchange approach using potassium poly(heptazine imide) (K-PHI) and Co-containing precursors. The close proximity and synergistic interactions between photoactive centers and single-atomic Co sites endow the catalyst with exceptional catalytic performance, enabling remarkable activity even at very low Co loading (0.07–0.34 mol%) and delivering high yields with good selectivity in decarboxylative Heck couplings, surpassing multi-component homogeneous catalyst systems. Furthermore, the integrated catalyst exhibits high stability and can be recycled at least six times without loss of activity and selectivity. This heterogeneous metallaphotocatalytic protocol not only facilitates the efficient synthesis of a diverse array of multi-substituted alkenes with good functional group tolerance, but also allows for the late-stage functionalization of bioactive natural products and the synthesis of pharmaceutically relevant compounds. This work offers new insights into the synergistic effects between atomically dispersed metals and light-harvesting carriers to enhance catalytic activity and selectivity.

## Abstract 2

This work presents a novel single-atom cobalt photocatalyst that enables enhanced decarboxylative Heck coupling at remarkably low Co loading, attributed to the proximity and synergistic effects between photoactive centers and single-atomic Co sites.

## Graphical abstract

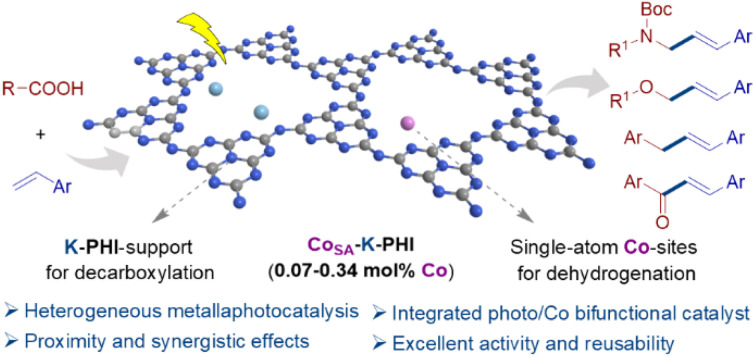

## Introduction

Metallaphotocatalysis, combining light-driven photocatalysis and transition metal catalysis, is an intriguing catalytic strategy for chemical synthesis and shows immense potential in developing challenging reactivity in organic chemistry.^1^ Homogeneous metallaphotocatalytic systems, though demonstrating remarkable synthetic capabilities, present inherent challenges including catalyst instability, particularly structural vulnerability to radicals and metal aggregation into nanoparticles, as well as difficulties in catalyst recycling.^2^ In this context, the development of heterogeneous metallaphotocatalysis, *via* the rational design and construction of stable and reusable metal-photo bifunctional catalysts, presents a promising avenue to address these limitations.^3^ Recent research has increasingly focused on developing metal-based single-atom photocatalysts (SAPs), featuring atomically dispersed metal sites anchored on light-harvesting supports.^4^ Due to their high atom utilization efficiency and the synergistic/proximity effects between photocatalytic and active metallic sites, SAPs have the potential to enhance the reactivity and selectivity of organic transformations even at low metal loadings.^4*b*^ However, creating such precisely regulated SAPs remains challenging. One major reason lies in the difficulty of controlling the active site speciation and structural uniformity of atomically dispersed catalysts.^5^ Moreover, metal-support interactions, while crucial for the stability of SAPs, may result in strong or near-saturated coordination and/or luminance quenching, potentially diminishing or deactivating the activity of both photocatalytic and metal centers.^4*a*^

In parallel with this, multi-substituted alkenes are ubiquitous in many natural products, bioactive molecules, and organic materials, and serve as valuable organic synthons for further derivatization into high-value chemicals.^6^ Decarboxylative Heck-type coupling, employing naturally abundant, non-toxic and structurally diverse carboxylic acids as feedstocks, stands out as one of the most straightforward approaches for rapidly constructing functionalized alkenes.^7^ Over the past decades, Pd-catalyzed and photo-induced systems have been developed, yet rely on the use of stoichiometric oxidants, pre-functionalized activated acid, and/or high temperatures.^8^ Recently, dual catalytic systems intergrating photoredox with palladium or cobalt have emerged, enabling oxidant-free decarboxylative Heck couplings under mild conditions.^9^ Despite their novelty and significance, these homogeneous metallaphotocatalytic systems contain two catalytic components with distinct catalytic centers surrounded by solvent, resulting in an increase in their average distance with decreasing concentration,^3^ which consequently leads to a drop in catalytic efficiency and the need for high catalyst loadings (>5 mol%). Moreover, their homogenous nature also gives rise to inherent issues related to catalyst recovery and reuse, which poses obstacles for enhancing sustainability.

**Fig. 1:**
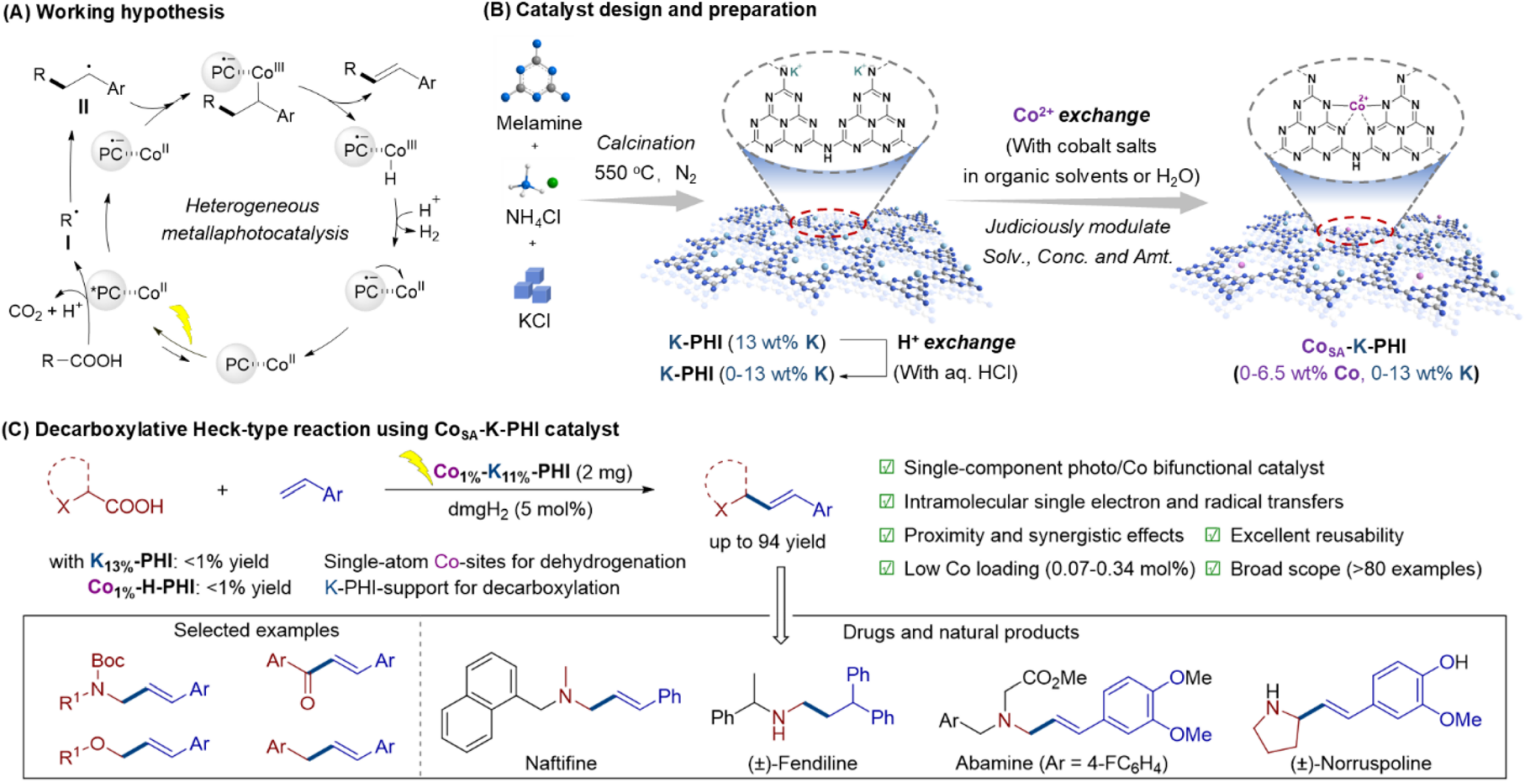
Heterogeneous metallaphotocatalysis using Co-based single-atom photocatalyst. (A) Working hypothesis. (B) Catalyst design and preparation. (C) Decarboxylative Heck-type reaction using Co_SA_–K-PHI catalyst.

Given all aforementioned factors, the development of an efficient and recyclable heterogeneous SAP system for enhanced decarboxylative Heck coupling is highly desirable yet challenging. We envision that a cobalt-based SAP may be highly effective for promoting decarboxylative Heck coupling of aliphatic acids with olefins (Fig. 1A). The photoactive support is expected to generate alkyl radicals I *via* single electron oxidation of carboxylic acids and CO_2_-extrusion, while the embedded Co center should capture the alkyl C-centered radical intermediates II and facilitate β-H abstraction to yield the desired multi-substituted alkenes. On the other hand, carbon nitride has garnered significant attention for its remarkable photocatalytic performance^10,11^ and has also emerged as a robust and versatile heterogeneous platform to serve as a support material.^12^ In particular, potassium poly(heptazine imide) (K-PHI), a crystalline subclass of ionic carbon nitride, has a well-defined and highly ordered structure, consisting of negatively charged poly(heptazine imide) (PHI) layers that are charge-balanced by potassium (K^+^) cations.^13^ This unique structural configuration allows for the substitution of K^+^ ions within the matrix with cationic cobalt (Co^2+^) species, offering a promising way to create isolated cobalt sites on a robust photocatalyst support.

In this study, we report the successful fabrication of Co-SAPs on ionic carbon nitride *via* a cation-exchange strategy, utilizing K-PHI and Co-containing precursors. Through this soft-chemical approach under mild conditions, we synthesized various Co–K-PHI or Co–PHI SAPs with precisely controlled Co and K contents (0–6.5 wt% for Co, 0–13 wt% for K) by adjusting Co species concentration and employing K-PHI precursors with varying K contents during the Co/K exchange (Fig. 1B). We demonstrated that Co–K-PHI containing 1 wt% Co and 11 wt% K emerged as a highly effective and cooperative Co-SAP, exhibiting exceptional catalytic performance at remarkably low Co loading (0.07–0.34 mol%) and achieving high yields with good selectivity in visible-light-driven decarboxylative Heck coupling. The newly developed heterogeneous Co-based SAP system not only enables the efficient synthesis of a diverse array of stereo-defined multi-substituted alkenes with good functional group tolerance, but also permits late-stage functionalization of bioactive natural products and the synthesis of pharmaceutically relevant compounds (Fig. 1C).

## Results and discussion

### Catalyst synthesis

We first prepared K-PHI supports with varying K contents to serve as a photocatalyst carrier for Co sites. Specifically, K-PHI with a high K content was synthesized by calcination of melamine and NH_4_Cl at 550 °C using KCl as a molten salt template.^12*d*^ The K content was determined to be 13 wt% by inductively coupled plasma-optical emission spectrometry (ICP-OES). K-PHI supports with lower K contents (6 wt% or 3 wt%) were obtained *via* a subsequent H/K exchange process using aqueous HCl (0.2 M, 7 mL g^−1^ or 11 mL g^−1^) with K-PHI (13 wt%). These materials were termed K_13%_-PHI, K_6%_-PHI and K_3%_-PHI. To incorporate Co into K-PHI framework, cobalt salts (CoCl_2_, Co(OAc)_2_ or Co(acac)_2_) were thoroughly mixed with K_13%_-PHI, K_6%_-PHI, or K_3%_-PHI in solvents such as DMF, MeCN, EtOH or H_2_O at room temperature for 12 hours, followed by centrifugation and extensive washing with DMF and a DMF/H_2_O (20/1) mixture to remove Co salts and other impurities. A series of representative Co–K-PHI materials with varying Co and K contents were synthesized, including Co_0.5%_–K_11%_-PHI, Co_1%_–K_11%_-PHI, Co_2%_–K_10%_-PHI, Co_3%_–K_8.8%_-PHI, Co_4%_–K_7.6%_-PHI, Co_4.8%_–K_6.5%_-PHI, Co_6.5%_–K_2.1%_-PHI, Co_1%_–K_7%_-PHI, Co_1%_–K_4.5%_-PHI and Co_1%_–H-PHI (Tables S1–S4 and Fig. S1, see the SI for additional details). It should be mentioned that during the exchange process in DMF, MeCN, and EtOH, only a partial release of K^+^ ions from K-PHI occurred, with substitution by Co^2+^ at a molar ratio close to 2 : 1. In contrast, when the procedure was conducted in H_2_O, Co/K exchange proceeded more rapidly and thoroughly, enabling efficient preparation of Co–K-PHI materials with high Co content. However, partial H/K exchange also occurred, resulting in a relatively lower K content in the obtained materials. The other transition-metal-based materials including Ni–K-PHI, Cu–K-PHI and Fe–K-PHI were obtained by following the same strategy.

### Identification of the active catalyst

**Fig. 2:**
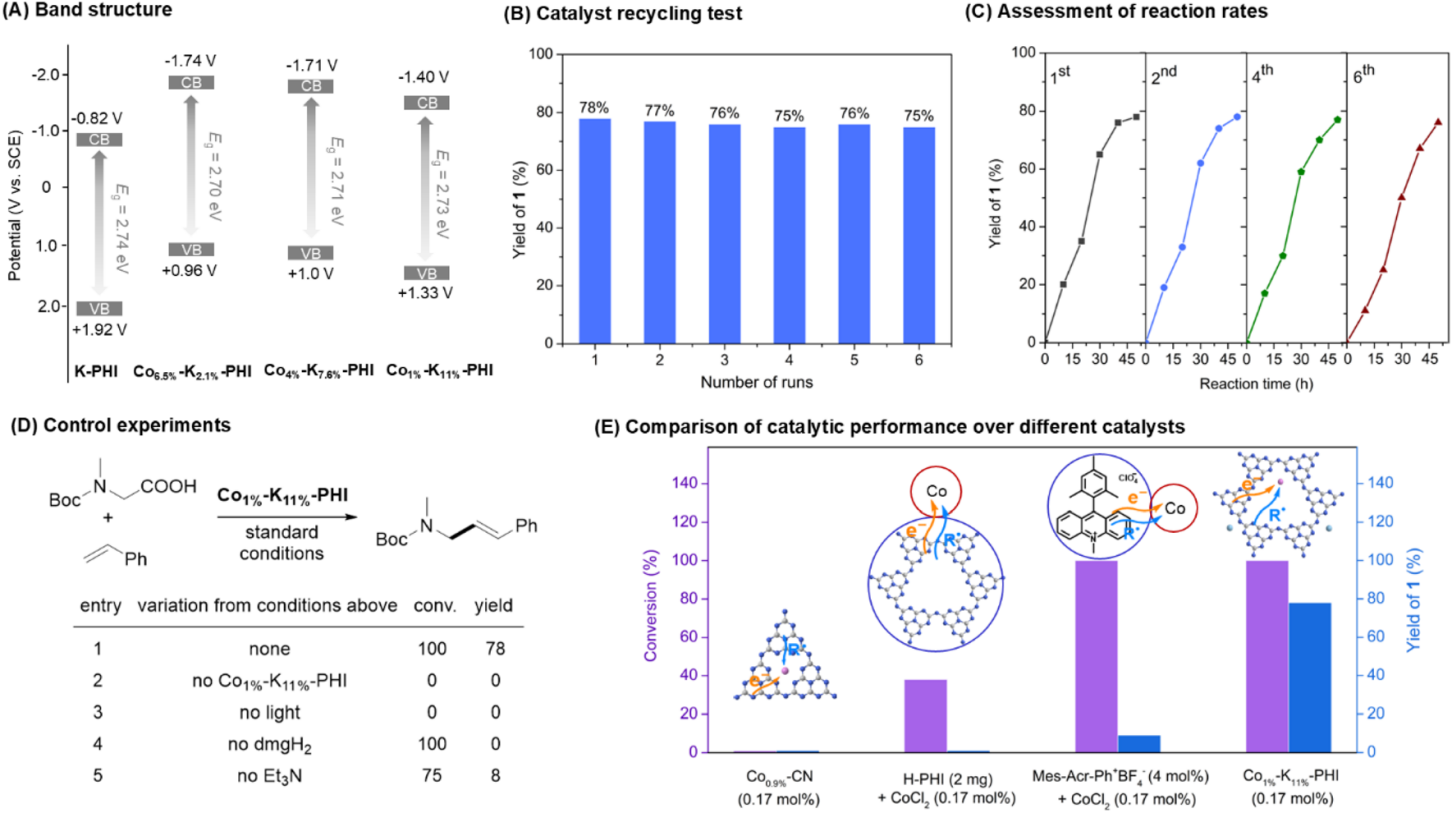
Study on catalytic activity and stability of Co_SA_–K-PHI. (A) Band structure. (B) Catalyst recycling test. (C) Assessment of reaction rates. (D) Control experiments. (E) Comparison of catalytic performance over different catalysts.

Our catalytic investigations were initiated using readily available substrates, *N*-Boc-*N*-methylglycine and styrene, with Et_3_N as the base and dimethylglyoxime (dmgH_2_) as the ligand in toluene under blue LEDs irradiation for the synthesis of the synthetically valuable cinnamyl amine 1*via* a decarboxylative Heck-type coupling reaction (Tables 1 and S5–S10). The prepared materials, such as Co_6.5%_–K_2.1%_-PHI, Co_4.8%_–K_6.5%_-PHI, Co_4%_–K_7.6%_-PHI, and Co_3%_–K_8.8%_-PHI, had high cobalt contents, which are theoretically beneficial for dehydrogenation and product formation. However, these catalysts showed either no activity or minimal yields (entries 1–4). To explore this contradiction, control and characterization experiments were performed. In the model reaction, K-PHI enabled acid conversion, generating various side products likely through decarboxylation-initiated styrene polymerization and alkyl radical dimerization (entry 5 and Fig. S7). In the decarboxylative radical addition of *N*-Boc-*N*-methylglycine to 4-benzylidene-2,6-di-*tert*-butylcyclohexa-2,5-dien-1-one, K-PHI achieved a high yield, while cobalt-containing materials with high Co contents were inactive (Table S11 and Fig. S8). These results indicate that excessive cobalt incorporation into the K-PHI framework reduces decarboxylation catalytic activity. The X-ray diffraction (XRD), attenuated total reflectance infrared (ATR-IR), and UV-vis diffuse reflectance spectra (DRS) analyses showed that these materials have similar crystal structures and compositions to K-PHI, with comparable light absorption and a band gap of about 2.74 eV (Fig. S2–S5). However, Mott–Schottky measurements indicated that, compared to K-PHI, these Co-containing materials have a much lower valence band potential (*E*_VB_), which makes oxidative decarboxylation unfavorable (Fig. 2A and S6). Gratifyingly, catalysts with low cobalt and high potassium content, such as Co_2%_–K_10%_-PHI and Co_1%_–K_11%_-PHI, possess suitable *E*_VB_ and exhibit significantly improved intrinsic catalytic activity, affording the desired cinnamyl amine 1 in high yields with good selectivity (entries 6 and 7).

**Table 1:** Screening of metal-modified PHI catalysts^a^

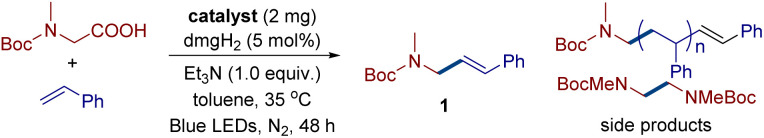				
Entry	Catalyst	Co (mol%)	conv.^b^ (%)	yield^b^ (1, %)
1	Co_6.5%_–K_2.1%_-PHI	1.11	0	0
2	Co_4.8%_–K_6.5%_-PHI	0.82	0	0
3	Co_4%_–K_7.6%_-PHI	0.68	0	0
4	Co_3%_–K_8.8%_-PHI	0.51	12	10
5	K-PHI	—	80	0
6	Co_2%_–K_10%_-PHI	0.34	100	78
7	Co_1%_–K_11%_-PHI	0.17	100	78(75)^c^
8	Co_0.5%_–K_11%_-PHI	0.085	51	25
9^d^	Co_0.5%_–K_11%_-PHI	0.085	100	48
10	Co_1%_–K_7%_-PHI	0.17	83	41
11	Co_1%_–K_4.5%_-PHI	0.17	40	0
12	Co_1%_–H-PHI	0.17	20	0
13	Fe_0.9%_–K_11.2%_-PHI	—	100	0
14	Cu_1%_–K_11.7%_-PHI	—	100	0
15	Ni_0.8%_–K_12.2%_-PHI	—	100	0
16^e^	Co_1%_–K_11%_-PHI	0.07	100	70

a Reaction conditions: *N*-Boc-*N*-methylglycine (0.2 mmol), styrene (1.0 mmol), catalyst (2 mg), Et_3_N (0.2 mmol), and dmgH_2_ (5 mol%) in toluene (2 mL) under N_2_ atmosphere and blue LEDs irradiation (24 W, 460 ± 5 nm) without extra heating (at 35 ± 5 °C) for 48 h.

b Conversion of *N*-Boc-*N*-methylglycine and yield of product 1 determined by ^1^H NMR analysis using 1,3,5-trimethoxybenzene as an internal standard.

c Data in parentheses represents the isolated yield of 1.

d 96 h.

e Reaction was conducted on a 2 mmol scale with 8 mg of catalyst for 96 h.

In contrast, catalysts with further reduced Co content, such as Co_0.5%_–K_11%_-PHI, resulted in decreased yield and selectivity of the product due to their higher decarboxylation activity, which increased side products, and lower dehydrogenation activity, which reduced the formation of the desired product (entries 8 and 9). Catalysts with reduced potassium content (*e.g.*, Co_1%_–K_7%_-PHI, Co_1%_–K_4.5%_-PHI, and Co_1%_–H-PHI) demonstrated significantly diminished catalytic performance and lower product yields with poor selectivity (entries 10–12). This trend may be attributed to the presence of K^+^ ions within the Co–K-PHI framework, where short-range induced dipole effects facilitate effective electron transfer from K-PHI framework to the Co active sites,^14^ thereby enhancing the reactivity and selectivity. When testing other earth-abundant metal-based materials, such as Fe_0.9%_–K_11.2%_-PHI, Cu_1%_–K_11.7%_-PHI, and Ni_0.8%_–K_12.2%_-PHI, full conversion of acid was observed, yet only a series of side products formed through decarboxylation-initiated processes, underscoring the critical role of the Co species as the active site in the catalytic process (entries 13–15). Notably, using Co_1%_–K_11%_-PHI as a single-component bifunctional catalyst, the catalyst loading could be reduced to 0.07 mol%, achieving a 70% yield (entry 16). In addition, the Co_1%_–K_11%_-PHI catalyst exhibited high stability and reusability, maintaining its catalytic efficiency over six cycles without any noticeable loss of activity, as evidenced by consistent reaction rates (Fig. 2B and C). This stability is further supported by the minimal leaching of cobalt from the catalyst, with only 3% per cycle as determined by ICP-OES analysis (Table S12). When leached cobalt was used in combination with the K-PHI catalyst in further tests, no products were formed. Meanwhile, the catalyst also showed no visible change in structural integrity, crystallinity, and optical property after recycling experiments, which was confirmed by XRD, FTIR and UV-vis DRS (Fig. S11–S13). These finding strongly indicated that cobalt is permanently bound to the PHI support, ensuring that a truly heterogeneous reaction process.

To verify the critical components of this heterogeneous metallaphotocatalytic system, additional control experiments were conducted (Fig. 2D). The reaction did not proceed without Co_1%_–K_11%_-PHI or light. It also showed extremely poor selectivity in the absence of the ligand or Et_3_N. These results highlight the indispensable role of ligand activated Co–K-PHI as a photo/Co bifunctional catalyst and the necessity of both light irradiation and base for the decarboxylative Heck-type coupling of styrene. Under otherwise identical conditions, Co_0.9%_–CN (with graphitic carbon nitride as the support),^15^ was inactive, underscoring the crucial role of the ionic carbon nitride as a photocatalytic carrier in influencing reactivity. A physical mixture of the CoCl_2_ and HPHI also exhibited negligible catalytic activity for the Heck coupling reaction. Furthermore, Co_1%_–K_11%_-PHI demonstrates far superior catalytic performance compared to previously known multi-component homogeneous catalysts, which require 5 mol% of Co loading to achieve comparable reactivity and selectivity (entry 16 *vs.* Table S5 entries 20–25 and Fig. 2E). These findings clearly confirm the critical role of the close proximity between Co sites and photocatalytic centers in Co–K-PHI, which facilitates efficient single electron transfer (SET) and synergistic interactions, thereby enhancing both activity and selectivity. In contrast, the catalytic efficiency of the H-PHI + CoCl_2_ and the Mes-Acr-Ph^+^BF_4_^−^ + CoCl_2_ system is significantly reduced, likely due to the inevitable interference of charge transfer and radical diffusion processes by the surrounding solvents.

### Structural characterization of atomically dispersed cobalt catalyst

**Fig. 3:**
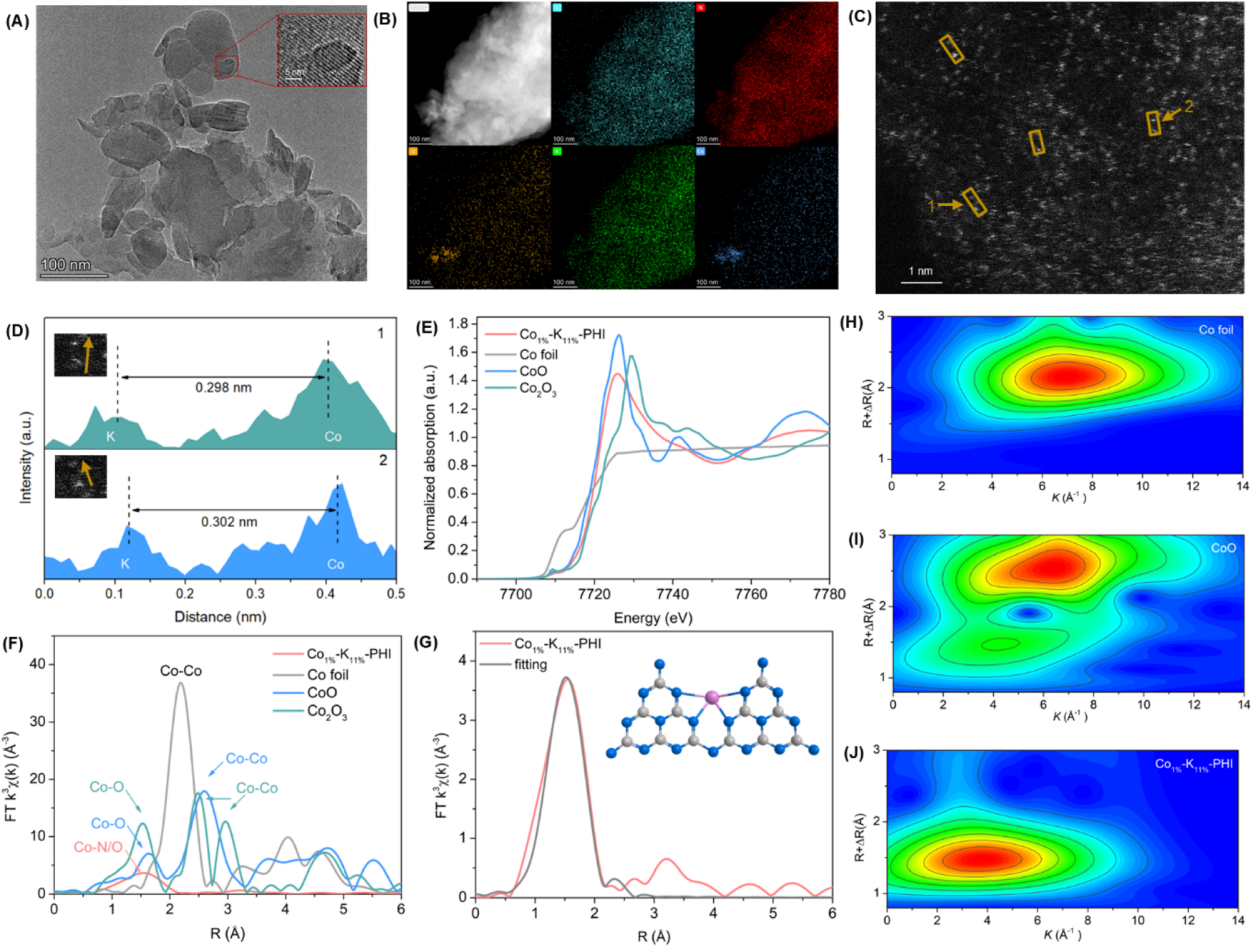
Characterizations of the Co_1%_–K_11%_-PHI SAP. (A) TEM image (inset: HRTEM image). (B) EDX elemental mappings of C, N, O, K, Co. (C) HAADF-STEM image. (D) Intensity profiles along the lines at position 1 and 2 in HAADF-STEM image. (E) Co K-edge XANES spectra. (F) FT-EXAFS spectra of Co foil, CoO, Co_2_O_3_ and Co_1%_–K_11%_-PHI. (G) The corresponding EXAFS fitting curves of Co_1%_–K_11%_-PHI (inset: the structure of the cobalt site in Co_1%_–K_11%_-PHI). (H–J) Wavelet transform of Co foil (H), CoO(i) and Co_1%_–K_11%_-PHI (J).

To further elucidate the origin of the observed remarkable catalytic activity and selectivity, the optimal Co_1%_–K_11%_-PHI catalyst was further characterized. Transmission electron microscopy (TEM), high-angle annular dark-field scanning transmission electron microscopy (HAADF-STEM) were employed to investigate the catalyst's morphology. The TEM image shows that Co_1%_–K_11%_-PHI exhibits a layered structure with nanometer-sized domains (Fig. 3A). Energy-dispersive X-ray spectroscopy (EDX) elemental mappings reveal the uniform dispersion of C, N, O, K, and Co elements throughout the catalyst (Fig. 3B). The HAADF-STEM image (Fig. 3C) indicates the atomically dispersed Co and K species on the support, distinguishable by the pronounced electron scattering effect, with no metal particles or clusters observed. The intensity profiles also show that the average distance between Co–K atom pairs in regions 1 and 2 is approximately 0.3 nm (Fig. 3D). X-ray photoelectron spectra (XPS) and X-ray absorption spectroscopy (XAS) were used to delve deeper into the chemical states and coordination structures of Co atoms in the Co_1%_–K_11%_-PHI catalyst. The Co 2p XPS spectrum displayed two distinct peaks at 781.3 eV (Co 2p_3/2_) and 797.1 eV (Co 2p_1/2_), which are characteristic of Co^2+^ species (Fig. S14).

The Co K-edge X-ray absorption near edge structure (XANES) spectra showed that the absorption energy of Co_1%_–K_11%_-PHI closely matches that of CoO, confirming the Co^2+^ oxidation state and corroborating the XPS findings (Fig. 3E). The Fourier transformation (FT) *k*^3^-weighted extended X-ray absorption fine structure (EXAFS) spectrum of Co_1%_–K_11%_-PHI (Fig. 3F) revealed a peak at 1.54 Å, indicative of Co–N/O coordination. The absence of Co–Co peaks suggested atomical dispersion of Co, consistent with HAADF-STEM observations.^16^ EXAFS fitting indicated that the Co center adopts Co–N_4_–O_2_ structure with a coordination number of approximately 6 (Fig. 3G and Table S14). While EXAFS could not discriminate the contribution from coordinated N and O atoms due to their similar scattering factors, Co–O bonds possibly from H_2_O might be present. Wavelet transform (WT) analysis shows an intensity maximum at about 4 Å^−1^ in *k* space, confirming Co–N first-shell coordination (Fig. 3H–J). Collectively, these results confirm that the Co species in the Co_1%_–K_11%_-PHI exist as single Co atoms, likely coordinated with four pyridinic nitrogen of adjacent triazine units (inset of Fig. 3G).

### Substrate scope and synthetic application

**Fig. 4:**
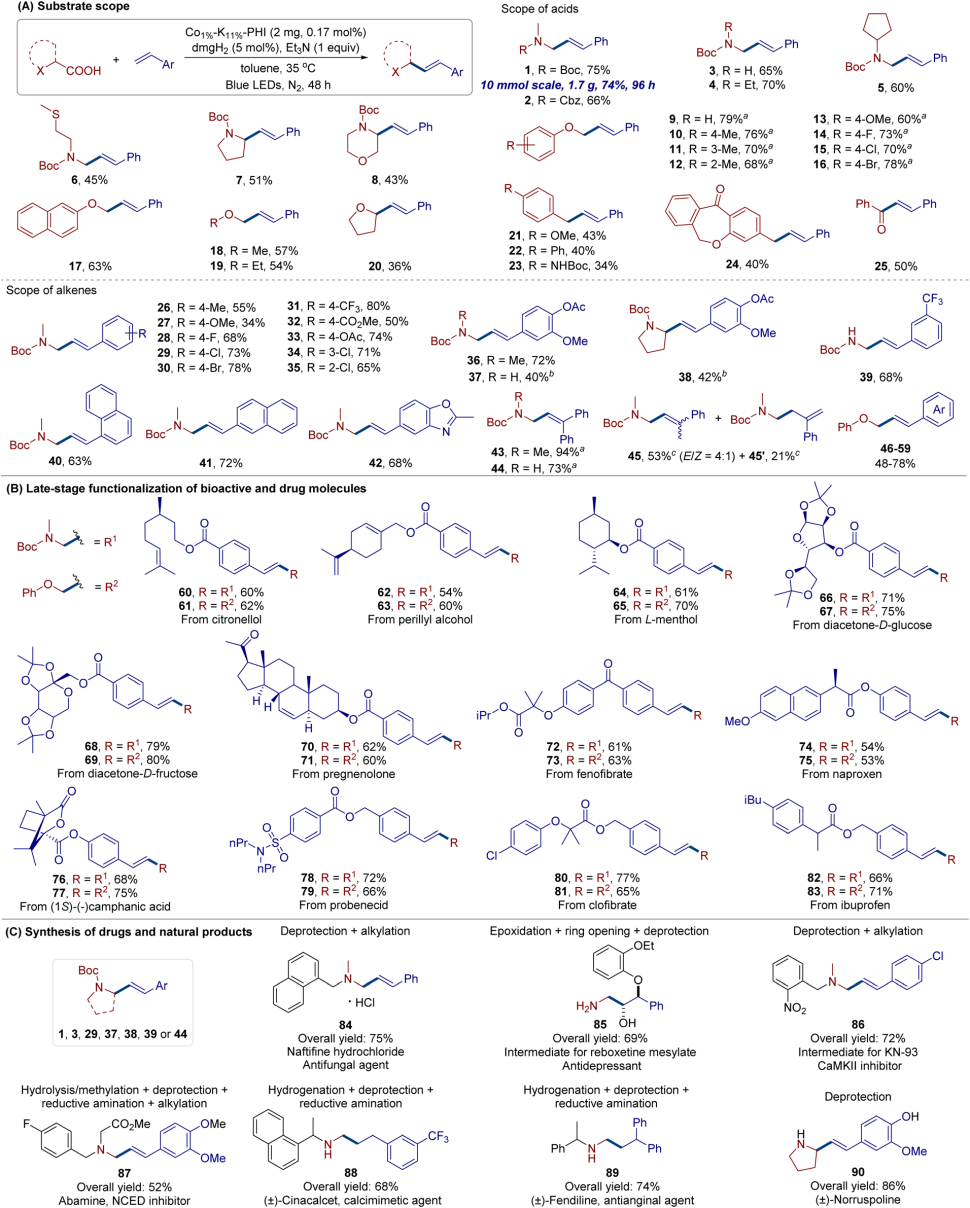
Substrate scope and synthetic applications of Co_1%_–K_11%_-PHI SAP catalyzed decarboxylative Heck-Type coupling. Isolated yield was given. (A) Substrate scope. Reaction was conducted on a 0.2 mmol scale under standard conditions. ^*a*^Performed with 2 equiv. of alkene. ^*b*^Performed with 4 mg of Co_1%_–K_11%_-PHI. ^*c*^Yield and *E*/*Z* ratio were determined by ^1^H NMR analysis using trimethyl benzene-1,3,5-tricarboxylate as an internal standard. (B) Late-stage functionalization of bioactive and drug molecules. Reaction was conducted on a 0.1 mmol scale under standard conditions. (C) Synthesis of drugs and natural products. See SI for detailed reaction conditions and procedures.

Encouraged by the outstanding photo/Co dual-catalytic activity of Co_1%_–K_11%_-PHI and having established optimal reaction conditions (Table 1, entry 7), we investigated the generality of this Co-SAP catalyzed decarboxylative Heck coupling of carboxylic acids and olefins (Fig. 4 and S16). First, multiple types of carboxylic acids were evaluated using styrene as the model alkene with a catalyst loading of 0.17 mol%. A range of readily available α-amino acids, including glycine derivatives bearing different *N*-protecting groups (*e.g.*, Cbz, Boc) and/or various *N*-alkyl groups (methyl, ethyl, cyclopentyl, or (2-methylthio)ethyl), along with *N*-protected proline and morpholine-derived substrates, underwent decarboxylative Heck-type coupling smoothly, yielding corresponding allylic amines in moderate to good yields (1–8, 43–75%). These allylic amines are common structural motifs in many natural products and pharmaceuticals and serve as versatile synthetic intermediates for constructing complex molecules and functional materials.^17^ Aryloxy acetic acids also functioned well as substrates for directly synthesizing aryl cinnamyl ethers (9–17, 60–79%), which are valuable synthetic precursors for preparing allyl-substituted phenols *via* direct Claisen rearrangement^18^ or functionalized chromans *via* cation induced arylations.^19^

Additionally, a variety of acyclic and cyclic α-alkyloxy acids were compatible with the reaction conditions, producing alkyl allyl ethers in 36–57% yields (18–20). Benzylic and α-keto acids were also suitable for this protocol, yielding 1,3-diaryl-1-propene and enone products in moderate yields (21–25). Subsequently, the scope of alkenes was evaluated using *N*-Boc-*N*-methylglycine as a model acid due to its functionality in pharmaceuticals. Diverse styrenes with various substituents (Me, MeO, F, Cl, Br, CF_3_, CO_2_Me, and OAc), vinylnaphthalene, vinylbenzoxazole, and 1,1-diphenylethylene all underwent heterogeneous photo/Co dual-catalyzed decarboxylative coupling to afford the desired cinnamyl amine products (26–44) in 34–94% yields. α-Methylstyrene also yielded 53% of allylic amine 45 (4 : 1 *E*/*Z*) and 21% of homoallylic amine 45′. It should be noted that full conversion of carboxylic acid was observed in all cases. However, in some instances, relatively low yields were attributed to the formation of side products resulting from photocatalytic decarboxylative radical-mediated polymerization of alkene and radical dimerization. Aliphatic alkenes (such as 1-dodecene or cyclopentene) failed to yield any desired coupling products under the current catalytic system, presumably due to the relatively unfavorable formation of the corresponding less stable alkyl radical intermediates upon decarboxylative alkyl radical addition. Furthermore, switching from *N*-Boc-*N*-methylglycine to phenoxyacetic acid as the model acid and reacting with various alkenes enables the efficient synthesis of diverse substituted cinnamyl phenyl ethers (46–59) in high yields (48–78%, please check SI for details). The gram-scale synthesis of allylic amine 1 was successfully achieved by scaling up the model reaction 50-fold, which still afforded the desired product in a 74% yield.

To demonstrate the synthetic potential of this heterogeneous metallaphotocatalytic protocol, we explored its application in late-stage diversification and the synthesis of bioactive compounds and natural products. Natural product-derived olefins, such as citronellol, phytol, l-menthol, d-glucose, d-fructose, and pregnenolone derivatives, readily underwent decarboxylative Heck coupling with *N*-Boc-*N*-methylglycine or phenoxyacetic acid, yielding the corresponding allylamine or allyl ether derivatives (60–71) in 54–80% yields. Similarly, various drug molecule derivatives, including fenofibrate (antihyperlipidemic agent), naproxen (analgesic), camphanic acid, probenecid (uricosuric agent), clofibrate (blood lipid-lowering drug), and ibuprofen (anti-inflammatory drug), were effectively transformed into desired products (72–83) in 53–77% yields. Moreover, the allylic amine derivatives (1, 3, 29, 37, 38, 39 and 44) obtained through this Co-based SAP system can be further elaborated through limited-step transformations to access diverse intermediates, pharmaceuticals, and natural products, such as the antifungal agent naftifine 84, key intermediates (85, 86) for synthesizing KN-93 (Ca^2+^/calmodulin-dependent protein kinase II inhibitor) and reboxetine (an antidepressant drug), abamine 87 (abscisic acid biosynthesis inhibitor), (±)-cinacalcet 88 (secondary hyperparathyroidism treatment), (±)-fendiline 89 (antianginal drug), and natural products (±)-norruspoline 90.

### Mechanistic studies and plausible mechanism

**Fig. 5:**
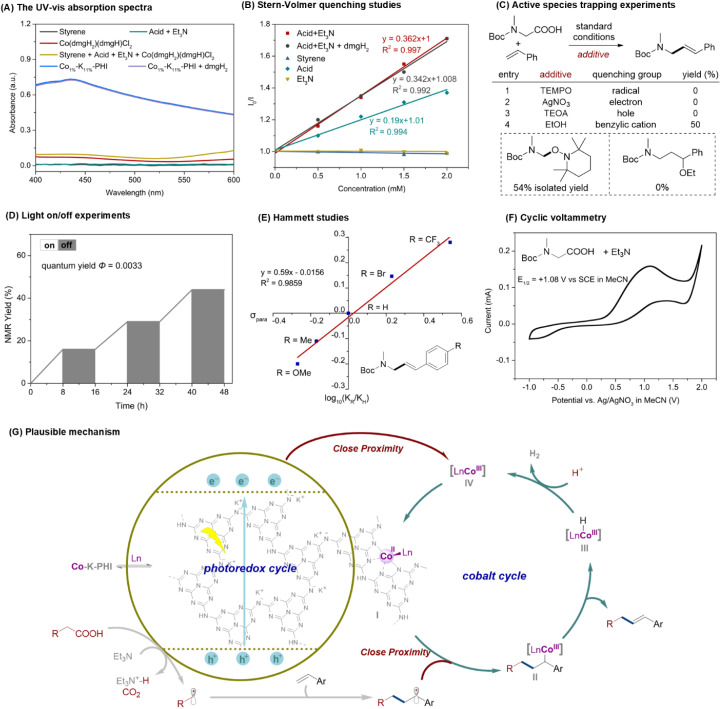
Mechanistic studies. (A) The UV-vis absorption spectra. (B) Stern–Volmer quenching studies. (C) Active species trapping experiments. (D) Light on/off experiments. (E) Hammett studies. (F) Cyclic voltammetry. (G) Plausible mechanism.

Finally, mechanistic experiments were conducted to elucidate the mechanism of this heterogeneous metallaphotocatalytic decarboxylative Heck-type coupling (Fig. 5). UV-vis absorption spectra of the reaction components showed that Co_1%_–K_11%_-PHI was the sole light-absorbing species around 460 nm (Fig. 5A). Stern–Volmer quenching studies clearly demonstrated that both *N*-Boc-*N*-methylglycine and the combination of *N*-Boc-*N*-methylglycine and Et_3_N could quench the excited state of Co_1%_–K_11%_-PHI, but the quenching extent by the combination of *N*-Boc-*N*-methylglycine and Et_3_N was much larger than that of *N*-Boc-*N*-methylglycine (Fig. 5B and S18–S22). In contrast, styrene, or Et_3_N alone fail to quench the emission of the excited photocatalyst. Radical trapping experiments using TEMPO as a scavenger confirmed the involvement of alkyl radicals, as evidenced by the formation of the radical/TEMPO adduct in 54% yield and the absence of the desired product (Fig. 5C, entry 1). Additionally, the reaction was significantly inhibited by the addition of AgNO_3_ (an electron scavenger) or triethanolamine (TEOA, a hole scavenger), demonstrating the importance of both electrons (e^−^) and holes (h^+^) in the process (Fig. 5C, entries 2 and 3). The inclusion of ethanol (EtOH) as a benzylic cation inhibitor resulted in no detection of the corresponding alcohol product (Fig. 5C, entry 4), ruling out a benzylic cation intermediate.^20^ Light on/off experiments and a quantum yield (*Φ* = 0.0033) indicated that continuous light irradiation is essential and that a radical chain mechanism is not involved (Fig. 5D). A positive Hammett plot slope (*ρ* = 0.59, Fig. 5E) suggests that benzylic radicals formed from electron-poor styrenes are more readily captured by the Co^II^ center. Cyclic voltammetry (CV) determined the oxidation potential of the ammonium salt of *N*-Boc-*N*-methylglycine to be +1.1 V *vs.* SCE in MeCN (Fig. 5F and S25). The valence band (VB) and conduction band (CB) potentials of Co_1%_–K_11%_-PHI are +1.33 V and −1.40 V, respectively, which thermodynamically favor the single-electron oxidation of the carboxylate and the reduction of Co^III^ species [E(Co^III^/Co^II^) = −0.48 V *vs.* SCE].^21^

Based on these findings and previous reports,^9*a*,22^ a tentative mechanism for the Co-SAP catalyzed decarboxylative Heck-type reaction is proposed (Fig. 5G). In this integrated photo/Co bifunctional heterogeneous catalyst system, the PHI support acts as the photocatalyst, while the embedded Co^II^ centers serve as the metal catalytic sites. Upon photoexcitation, electron–hole pairs are generated on the surface of Co_1%_–K_11%_-PHI. The photogenerated holes in the VB induce single-electron oxidation of the carboxylate, followed by CO_2_-extrusion to form an alkyl radical. This alkyl radical adds to styrene, forming a benzylic radical that is captured by the Co^II^ center to generate alkyl-Co^III^ species (II). Under light irradiation, homolytic cleavage of the Co^III^–C bond and subsequent β-hydride abstraction yield the desired product and Co^III^–H species (III). The Co^III^–H species then react with a proton to release H_2_ and form Co^III^ intermediate (IV). Finally, the photogenerated electrons transfer from the PHI support to Co^III^*via* intramolecular ligand-to-metal charge transfer, regenerating of Co^II^ species (I) and completing the catalytic cycle. The close proximity and highly synergistic effects in Co_SA_–K-PHI significantly enhance the Co/photo dual catalytic activity, thereby improving the overall catalytic efficiency and reaction selectivity.

## Conclusions

In summary, we have developed a novel Co-based single-atom photocatalyst anchored on ionic carbon nitride (K-PHI) for the decarboxylative Heck-type coupling of carboxylic acids with olefins. Key to this success was the implementation of a mild Co/K exchange strategy, which enabled the synthesis of a bifunctional catalyst with specific, atomically dispersed Co and K on the K-PHI photocatalyst carrier. This catalyst, benefiting from the close proximity and synergistic interactions between photoactive centers and single-atomic Co sites, delivers high yields even at very low Co loadings, outperforming multi-component homogeneous catalysts. It allows for the efficient synthesis of a diverse array of multi-substituted alkenes with good functional group tolerance and has shown practical applications in the late-stage functionalization of natural products and the synthesis of pharmaceutically relevant compounds. Furthermore, this Co–K-PHI SAP maintains its activity and selectivity over multiple cycles, confirming its stability and practical utility. We believe that this work provides novel insights into the synergistic effects between atomically dispersed metals and light-harvesting carriers to boost catalytic activity and selectivity, opening new avenues for developing PHI-supported SAPs in sustainable chemical synthesis. Maintains its activity and selectivity over multiple cycles, confirming its stability and practical utility.

## Author contributions

Q. Y. and W. L. W. performed photocatalytic experiments; Q. Y., M. T. W and H. W. synthesized Co_SA_–K-PHI catalysts; Q. Y. and W. T. W. characterized the Co_SA_–K-PHI catalysts and checked data; Y. R. T., W. T. W., M. R. and Y. F. C. conceived and supervised the research study. Q. Y., M. R. and Y. F. C. wrote the paper with input from all authors. All authors discussed the results.

## Conflicts of interest

There are no conflicts to declare.

## Supplementary Material

SC-016-D5SC04589D-s001

## Data Availability

Data for this article, including experimental precedure, supplementary tables and figures, characterization data of the products, *etc.*, are available in the SI. See DOI: https://doi.org/10.1039/d5sc04589d.
