# Supplementary material for: Atomically precise single-atom cobalt photocatalyst for enhanced decarboxylative cross-couplings
Source: Chem Sci. 2025 Sep 16;16(41):19154–63. doi: 10.1039/d5sc04589d (PMC12451602; doi:10.1039/d5sc04589d)

Supplementary Information (SI) for Chemical Science.
This journal is © The Royal Society of Chemistry 2025

Electronic Supplementary Material (ESI) for Chemical Science.

Supplementary Information

Atomically Precise Single-Atom Cobalt Photocatalyst for Enhanced Decarboxylative Cross-Couplings

Qian Yang,[a] Mengting Wu,[a] Wanlin Wang,[a] Wentao Wang,*[b] Han Wang,[a] Yurong Tang,*[a] Magnus Rueping,*[c] and Yunfei Cai*[a]

[a]School of Chemistry and Chemical Engineering, Chongqing University, 174 Shazheng Street, Chongqing 400044, China

[b]Dalian Institute of Chemical Physics, Chinese Academy of Sciences, Dalian 116023, China

[c]KAUST Catalysis Center (KCC), King Abdullah University of Science and Technology (KAUST), Thuwal, 23955-6900, Saudi Arabia

Table of Contents

1 General Information S2

2 Procedure for the Preparation of Starting Materials S3

3 Procedure for the Preparation of Co_{SA}-K-PHI Catalysts S9

4 Characterization of Co_{SA}-K-PHI S13

5 Optimization of the Reaction Conditions and Control Experiments S16

6 Experimental Procedure S20

7 Mechanistic Investigations S28

8 Characterization Date for the Products S33

9 Synthetic Applications S59

10 References S68

11 NMR Spectra S70

1 General Information

All reactions were carried out using oven-dried glassware and magnetic stirring under an inert atmosphere (N_2) unless otherwise stated. All chemical were obtained from commercial supplier and were used without further purification unless otherwise stated. All solvents were dried and distilled under N_2 prior to use. Solvents for chromatography were of technical grade and distilled prior to use. Analytical thin layer chromatography was carried out using silica gel GF254, visualized under UV light (at 254 nm). All NMR spectra were measured at room temperature using a a Bruker 400 (400 MHz for ^{1}H, 101 MHz for ^{13}C and 377 MHz for ^{19}F) NMR spectrometer in $CDCl_3$ solutions with internal solvent signals (for ^{1}H and ^{13}C) as reference (7.26, 77.2 for $CDCl_3$). All chemical shifts are reported in δ-scale as parts per million [ppm] (multiplicity, coupling constant *J*, number of protons) relative to the solvent residual peaks as the internal standard. The following abbreviations were used to express the multiplicities: s = singlet; d = doublet; t = triplet; q = quartet; m = multiplet; dd = doublet of doublets; dt = doublet of triplets; td = triplet of doublets; br = broad. Melting points were measured using a melting point apparatus in open glass capillaries.

High resolution mass spectra were acquired on a SolariX 15.0 T FT-MS (ESI). The light-promoted reactions were carried out by using standard blue LEDs with 28 blue LED beads (EPISTAR, 1 W LED beads and wavelength 460 ± 5 nm), the output power at 3 cm distance from the light source 19 mW/cm^2. Irradiance of the LED modules was measured using CEL-NP2000 optical power and energy meter equipped. Inductively coupled plasma optical emission spectrometry (ICP-OES) data was obtained from iCAP 6300 Duo. Degassing was performed under N_2 atmosphere. Fourier transform infrared (FTIR) spectra from 4000 to 400 cm^{-1} were recorded in KBr discs on a Nicolet iS50 FTIR spectrometer. XRD patterns of the samples were recorded using X' Pert Pro X-ray diffractometer (Philips) with Cu Kα radiation (λ = 1.54 Å). Diffuse-reflectance UV-vis (DRUV-vis) absorption spectra of the samples in solid state has been acquired using Shimadzu UV-3600. Photocurrent performance was performed with a Zahner Ennium electrochemical workstation. Transmission electron microscopy (TEM) images and EDX elemental mappings were obtained on a Talos F200S transmission electron microscope. Aberration-corrected high-angle annular dark field scanning transmission electron microscopy (HAADF-STEM) images were conducted on a double-corrected Titan ETEM G2 80-300 transmission electron microscopy instrument at voltage of 300 kV. X-ray photoelectron spectroscopy (XPS) was performed with a ESCALAB250Xi electron spectrometer using monochromatic Al Ka radiation. Cyclic voltammetry (CV) was performed on CHI610E. XAFS and EXAFS data were collected in fluorescence mode on the X-ray Absorption Spectroscopy beamline at the Shanghai Synchrotron (200 mA, 3.0 GeV). The powder samples were homogeneously mixed with cellulose to obtain a metal concentration around 1000 ppm. A Si (111) single crystal was used to monochromatize the X-ray beam. XANES and EXAFS data reduction and analysis were processed by Athena software.

2 Procedure for the Preparation of Starting Materials

2.1 Preparation of *N*-alkylated-*N*-Boc-glycine[1]

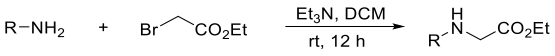

The product was synthesized by following a reported procedure with modifications.[1a] Amine (10.0 mmol, 1.0 equiv) was dissolved in 200 mL dichloromethane (DCM), then the solution of ethyl 2-bromoacetate (12.0 mmol, 1.2 equiv) and triethylamine (15.0 mmol, 1.5 equiv) was added into the reaction flask. The reaction mixture was stirred at room temperature for 12 h. The mixture was cooled to room temperature, followed by washing with deionization water three times (3 × 20 mL) and brine once (20 mL), and the residue was dried over anhydrous $MgSO_4$. After removing DCM solvent under vacuum, a crude product was obtained and further purified through silica gel column chromatography to give the product *N*-alkylated-glycine ethyl ester.

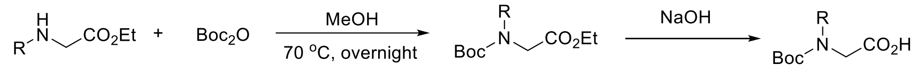

The product was synthesized by following a reported procedure with modifications.[1b] *N*-Alkylated-glycine ethyl ester (10.0 mmol, 1.0 equiv) was dissolved in 20 mL methanol (MeOH), and then di-*tert*-butyl dicarbonate (12.0 mmol, 1.2 equiv) was added into the reaction round-bottomed flask. The reaction mixture was heated to reflux for overnight. An intermediate was obtained after removing solvent MeOH under vacuum and then used directly for the next step. The obtained intermediate was dissolved in a mixed solution of 10 mL MeOH and 5 mL tetrahydrofuran (THF), and then a solution of NaOH (0.1 M, 3 mL) was added dropwise into the mixture, followed by stirring the reaction at room temperature for 5 hours. After that, the mixture was adjusted to neutral pH with a HCl solution (0.1 M). After removing the organic solvent under vacuum, the residue was acidified to pH 3-4 by HCl solution (0.1 M) at 0 °C and then extracted with ethyl acetate (EA). After dried over anhydrous $MgSO_4$, the EA phase was concentrated under vacuum to obtain the product.

***N*-(*tert*-butoxycarbonyl)-*N*-cyclopentylglycine[1a] (a5)**

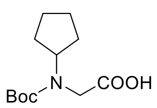

a5

White solid. **^{1}H NMR** (400 MHz, $CDCl_3$) δ 9.25 (br, 1H), 4.51 (br, 1H), 3.84 (br, 2H), 1.94 – 1.82 (m, 2H), 1.72 – 1.60 (m, 2H), 1.59 – 1.51 (m, 2H), 1.45 (s, 9H), 1.40 – 1.29 (m, 2H). **^{13}C NMR** (101 MHz, $CDCl_3$) δ 176.5, 155.1, 80.6, 56.5, 44.9, 29.4, 28.3, 23.6.

***N*-(*tert*-butoxycarbonyl)-*N*-(2-(methylthio)ethyl)glycine[1b] (a6)**

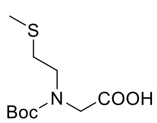

a6

Yellow oil. **^{1}H NMR** (400 MHz, $CDCl_3$) δ 8.39 (br, 1H), 4.03 (d, J = 27.5 Hz, 2H), 3.47 (dt, J = 20.1, 7.4 Hz, 2H), 2.77 – 2.61 (m, 2H), 2.13 (d, J = 3.7 Hz, 3H), 1.46 (d, J = 19.0 Hz, 9H). **^{13}C NMR** (101 MHz, $CDCl_3$) δ 175.4 (d, J = 57.7 Hz), 155.3 (d, J = 79.2 Hz), 81.0 (d, J = 21.4 Hz), 49.8 (d, J = 53.8 Hz), 48.3 (d, J = 29.7 Hz), 32.4 (d, J = 24.3 Hz), 28.3 (d, J = 14.6 Hz), 15.5 (d, J = 8.7 Hz).

2.2 Preparation of Alkenes

Alkenes **b36**[2], **b42**[3], **b60**[4], **b62**[4], **b64**[4], **b66**[4], **b68**[5], **b70**[6], **b72**[4], **b74**[7], **b76**[8], **b78**[5], **b80**[7], and **b82**[8] were known compounds and prepared according to reported methods. The following starting materials were prepared according to reported literature procedures.

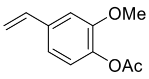
b36

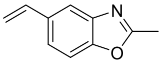
b42

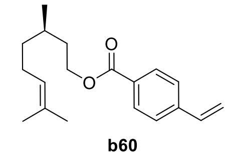
b60

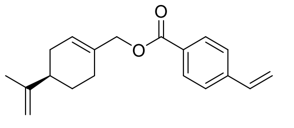
b62

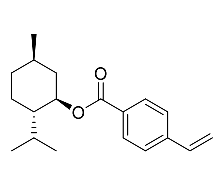
b64

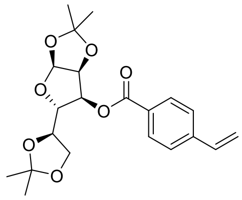
b66

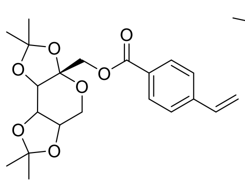
b68

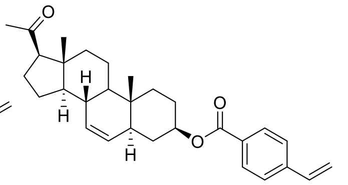
b70

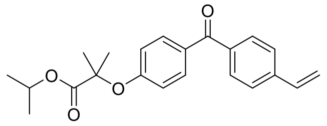
b72

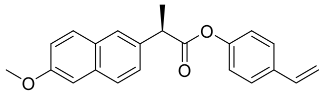
b74

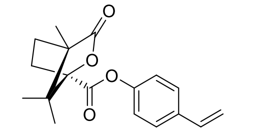
b76

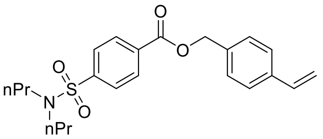
b78

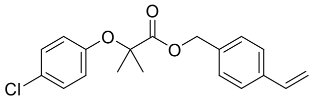
b80

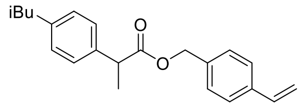
b82

2-methoxy-4-vinylphenyl acetate[2] (b36)

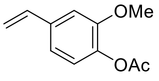
b36

A mixture of 4-vinylguaiacol (10.0 mmol, 1.0 equiv) and sodium acetate (0.5 mmol, 5 mmol%) was dissolved in acetic anhydride (15.0 mmol, 1.5 equiv), and stirred at 90 °C for 30 min. In the workup procedure, 5 mL of ethyl acetate was added to the reaction mixture and washed with a Saturated brine solution (3 × 5 mL). The organic layer was acidified with a 1.0 M HCl solution to a pH of 2, the layers were separated, and the product was precipitated in 5 mL of brine solution. The precipitate was dried at 50 °C in a vacuum oven overnight. Yellow oil (1.52 g, 79%). **^{1}H NMR** (400 MHz, $CDCl_3$) δ 6.98 (d, J = 9.3 Hz, 3H), 6.66 (dd, J = 17.6, 10.8 Hz, 1H), 5.68 (d, J = 17.6 Hz, 1H), 5.23 (d, J = 10.9 Hz, 1H), 3.82 (s, 3H), 2.29 (s, 3H). **^{13}C NMR** (101 MHz, $CDCl_3$) δ 169.1, 151.1, 139.5, 136.7, 136.3, 122.8, 118.9, 114.1, 109.9, 55.8, 20.7.

2-methyl-5-vinylbenzo[*d*]oxazole[3] (b42)

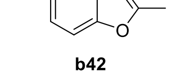
b42

To a suspension of 5-bromo-2-methylbenzoxazole (10.0 mmol, 1.0 equiv) in 1,4-dioxane (20 mL) and water (1 mL) was added potassium vinyltrifluoroborate (12.0 mmol, 1.2 equiv), Cs_2CO_3 (20.0 mmol, 2.0 equiv) and tetrakis(triphenylphosphorus) palladium (0) (0.5 mmol, 5 mol%). The mixture was stirred at reflux under nitrogen for 5 h. The mixture was then poured onto ice-water (20 mL) and extracted with EA (3×30 mL). The organic phases

were combined, dried over anhydrous $MgSO_4$, filtered and concentrated in vacuo. The residue was purified by chromatography on silica gel (PE/EA = 20:1) to afford the product as a colorless oil (1.26 g, 79%). **^{1}H NMR** (400 MHz, $CDCl_3$) δ 7.66 (d, *J* = 1.7 Hz, 1H), 7.41 – 7.27 (m, 2H), 6.78 (dd, *J* = 17.5, 10.9 Hz, 1H), 5.73 (d, *J* = 17.5 Hz, 1H), 5.23 (d, *J* = 10.9 Hz, 1H), 2.60 (s, 3H). **^{13}C NMR** (101 MHz, $CDCl_3$) δ 164.4, 150.7, 142.0, 136.6, 134.3, 123.0, 116.9, 113.5, 110.0, 14.5.

(*S*)-3,7-dimethyloct-6-en-1-yl 4-vinylbenzoate[4] (b60)

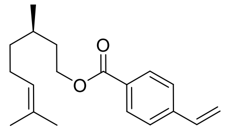

b60

To a solution of 4-vinylbenzoic acid (10.0 mmol, 1.0 equiv) in DCM (35 mL) at room temperature were added EDCI·HCl (22.0 mmol, 2.2 equiv), DMAP (2.0 mmol, 20 mol%), and citronellol (10.0 mmol, 1.0 equiv). The reaction mixture was stirred at room temperature until the disappearance of citronellol as monitored by TLC. The mixture was diluted with water, extracted with DCM (3 × 15 mL), dried over anhydrous Na_2SO_4, and filtered. The residue was purified by chromatography on silica gel (PE/EA = 7:1) to afford the product as a yellow oil (2.29 g, 80%). **^{1}H NMR** (400 MHz, $CDCl_3$) δ 7.99 (d, *J* = 8.5 Hz, 2H), 7.45 (d, *J* = 8.4 Hz, 2H), 6.75 (dd, *J* = 17.6, 10.9 Hz, 1H), 5.86 (d, *J* = 17.6 Hz, 1H), 5.37 (d, *J* = 10.9 Hz, 1H), 5.14 – 5.05 (m, 1H), 4.52 – 4.12 (m, 2H), 2.12 – 1.91 (m, 2H), 1.88 – 1.75 (m, 1H), 1.70 – 1.53 (m, 8H), 1.47 – 1.34 (m, 1H), 1.27 – 1.21 (m, 1H), 0.97 (d, *J* = 6.5 Hz, 3H). **^{13}C NMR** (101 MHz, $CDCl_3$) δ 166.5, 141.8, 136.1, 131.4, 129.9, 129.7, 126.1, 124.6, 116.4, 63.5, 37.0, 35.5, 29.6, 25.7, 25.4, 19.5, 17.7.

(*S*)-(4-(prop-1-en-2-yl)cyclohex-1-en-1-yl)methyl 4-vinylbenzoate[4] (b62)

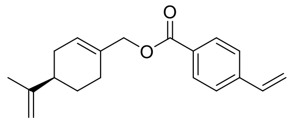

b62

To a solution of 4-vinylbenzoic acid (10.0 mmol, 1.0 equiv) in DCM (35 mL) at room temperature were added EDCI·HCl (22.0 mmol, 2.2 equiv), DMAP (2.0 mmol, 20 mol%), and perillol (10.0 mmol, 1.0 equiv). The reaction mixture was stirred at room temperature until the disappearance of citronellol as monitored by TLC. The mixture was diluted with water, extracted with DCM (3 × 15 mL), dried over anhydrous Na_2CO_3, and filtered. The residue was purified by chromatography on silica gel (PE/EA = 7:1) to afford the product as a yellow oil (2.12 g, 75%). **^{1}H NMR** (400 MHz, $CDCl_3$) δ 8.01 (d, *J* = 8.4 Hz, 2H), 7.45 (d, *J* = 8.4 Hz, 2H), 6.75 (dd, *J* = 17.6, 10.9 Hz, 1H), 6.02 – 5.69 (m, 2H), 5.37 (d, *J* = 10.9 Hz, 1H), 4.85 – 4.57 (m, 4H), 2.25 – 2.11 (m, 4H), 2.06 – 1.94 (m, 1H), 1.91 – 1.82 (m, 1H), 1.74 (s, 3H), 1.60 – 1.45 (m, 1H). **^{13}C NMR** (101 MHz, $CDCl_3$) δ 166.2, 149.6, 141.9, 136.1, 132.7, 129.9, 129.6, 126.1, 125.6, 116.4, 108.8, 68.8, 40.9, 30.5, 27.4, 26.5, 20.8.

(1*R*,2*S*,5*R*)-2-isopropyl-5-methylcyclohexyl 4-vinylbenzoate[4] (b64)

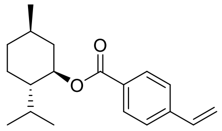

b64

To a solution of 4-vinylbenzoic acid (10.0 mmol, 1.0 equiv) in DCM (35 mL) at room temperature were added EDCI·HCl (22.0 mmol, 2.2 equiv), DMAP (2.0 mmol, 20 mol%), and (-)-menthol (10.0 mmol, 1.0 equiv). The reaction mixture was stirred at room temperature until the disappearance of (-)-menthol as monitored by TLC. The mixture was diluted with water, extracted with DCM (3 × 15 mL), dried over anhydrous Na_2CO_3, and filtered. The solvent was removed under reduced pressure. The residue was purified by chromatography on silica gel (PE/EA = 7:1) to afford the product as a yellow oil. (2.18 g, 76%). **^{1}H NMR** (400 MHz, $CDCl_3$) δ 8.00 (d, *J* = 8.4 Hz, 2H), 7.45 (d, *J* = 8.4 Hz, 2H), 6.75 (dd, *J* = 17.6, 10.9 Hz, 1H), 5.85 (d, *J* = 17.6 Hz, 1H), 5.37 (d, *J* = 11.0 Hz, 1H), 4.93 (td, *J* = 10.8, 4.4 Hz, 1H), 2.18 – 2.07 (m, 1H), 2.00 – 1.91 (m, 1H), 1.77 – 1.68 (m, 2H), 1.60 – 1.51 (m, 2H), 1.16 – 1.05 (m, 2H), 0.92 (dd, *J* = 6.9, 4.7 Hz, 7H), 0.79 (d, *J* = 7.0 Hz, 3H). **^{13}C NMR** (101 MHz, $CDCl_3$) δ 165.9, 141.8, 136.1, 130.0, 129.9, 126.1, 116.3, 74.8, 47.3, 41.0, 34.4, 31.5, 26.6, 23.7, 22.1, 20.8, 16.6.

(3a*S*,5*S*,6*S*,6a*S*)-5-((*R*)-2,2-dimethyl-1,3-dioxolan-4-yl)-2,2-dimethyltetrahydrofuro[2,3-*d*][1,3]dioxol-6-yl 4-vinylbenzoate[4] (b66)

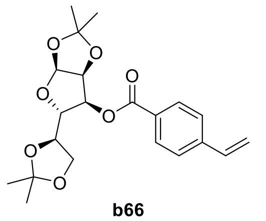

b66

To a solution of 4-vinylbenzoic acid (10.0 mmol, 1.0 equiv) in DCM (35 mL) at room temperature were added EDCI·HCl (22.0 mmol, 2.2 equiv), DMAP (2.0 mmol, 20 mol%), and diacetone-D-glucose (10.0 mmol, 1.0 equiv). The reaction mixture was stirred at room temperature until the disappearance of diacetone-D-glucose as monitored by TLC. The mixture was diluted with water, extracted with DCM (3 × 15 mL), dried over anhydrous Na_2CO_3, and filtered. The solvent was removed under reduced pressure. The residue was purified by chromatography on silica gel (PE/EA = 1:1) to afford the product as a white solid. (2.77 g, 71%). **^{1}H NMR** (400 MHz, $CDCl_3$) δ 8.04 – 7.89 (m, 2H), 7.54 – 7.40 (m, 2H), 6.76 (dd, *J* = 17.5, 10.8 Hz, 1H), 5.95 (d, *J* = 3.7 Hz, 1H), 5.87 (d, *J* = 17.6 Hz, 1H), 5.50 (d, *J* = 2.6 Hz, 1H), 5.41 (d, *J* = 10.9 Hz, 1H), 4.63 (d, *J* = 3.7 Hz, 1H), 4.41 – 4.29 (m, 2H), 4.16 – 4.04 (m, 2H), 1.56 (s, 3H), 1.41 (s, 3H), 1.32 (s, 3H), 1.27 (s, 3H). **^{13}C NMR** (101 MHz, $CDCl_3$) δ 165.0, 142.5, 135.9, 130.0, 128.6, 126.3, 117.0, 112.4, 109.4, 105.2, 83.4, 80.0, 76.6, 72.6, 67.3, 26.8, 26.8, 26.2, 25.2.

((3a*S*)-2,2,7,7-tetramethyltetrahydro-3a*H*-bis([1,3]dioxolo)[4,5-*b*:4',5'-*d*]pyran-3a-yl)methyl 4-vinylbenzoate[5] (b68)

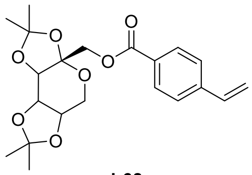

b68

To a solution of 4-vinylbenzoic acid (10.0 mmol, 1.0 equiv) in DCM (35 mL) at room temperature were added DIC (12.0 mmol, 1.2 equiv), DMAP (4.0 mmol, 40 mol%), and diacetonefructose (10.0 mmol, 1.0 equiv). The reaction mixture was stirred at room temperature until the disappearance of diacetonefructose as monitored by TLC. The mixture was diluted with water, extracted with DCM (3 × 15 mL), dried over anhydrous Na_2CO_3, and filtered. The solvent was removed under reduced pressure. The residue was purified by chromatography on silica gel (PE/EA =

1:1) to afford the product as a yellow oil. (3.20 g, 82%). **^{1}H NMR** (400 MHz, $CDCl_3$) δ 8.03 (d, *J* = 7.9 Hz, 2H), 7.46 (d, *J* = 8.0 Hz, 2H), 6.75 (dd, *J* = 17.6, 10.8 Hz, 1H), 5.87 (d, *J* = 17.6 Hz, 1H), 5.39 (d, *J* = 10.9 Hz, 1H), 4.67 (dd, *J* = 16.4, 9.8 Hz, 2H), 4.48 (s, 1H), 4.30 (dd, *J* = 24.0, 9.8 Hz, 2H), 3.96 (d, *J* = 13.0 Hz, 1H), 3.81 (d, *J* = 13.0 Hz, 1H), 1.55 (s, 3H), 1.47 (s, 3H), 1.36 (d, *J* = 8.5 Hz, 6H). **^{13}C NMR** (101 MHz, $CDCl_3$) δ 165.7, 142.1, 136.0, 130.1, 129.0, 126.1, 116.6, 109.2, 108.9, 101.7, 70.8, 70.6, 70.1, 65.2, 61.4, 26.5, 25.9, 25.5, 24.0.

4-vinylbenzyl 4-(*N*,*N*-dipropylsulfamoyl)benzoate[6] (b70)

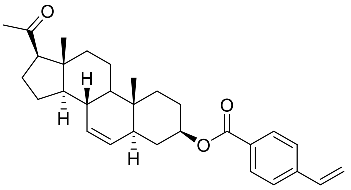

b70

To a solution of 4-vinylbenzoic acid (10.0 mmol, 1.0 equiv) in DCM (35 mL) at room temperature were added DCC (12.0 mmol, 1.2 equiv), DMAP (4.0 mmol, 40 mol%), and pregnenolone (10.0 mmol, 1.0 equiv). The reaction mixture was stirred at room temperature until the disappearance of pregnenolone as monitored by TLC. The mixture was diluted with water, extracted with DCM (3 × 15 mL), dried over anhydrous Na_2CO_3, and filtered. The solvent was removed under reduced pressure. The residue was purified by chromatography on silica gel (PE/EA = 10:1) to afford the product as a white solid. (4.33 g, 97%). **^{1}H NMR** (400 MHz, $CDCl_3$) δ 7.99 (d, *J* = 8.4 Hz, 2H), 7.45 (d, *J* = 8.4 Hz, 2H), 6.75 (dd, *J* = 17.6, 10.9 Hz, 1H), 5.86 (d, *J* = 17.6 Hz, 1H), 5.42 (d, *J* = 5.0 Hz, 1H), 5.38 (d, *J* = 10.9 Hz, 1H), 4.96 – 4.77 (m, 1H), 2.55 (t, *J* = 8.9 Hz, 1H), 2.47 (d, *J* = 7.8 Hz, 2H), 2.25 – 2.15 (m, 1H), 2.13 (s, 3H), 2.10 – 1.87 (m, 4H), 1.82 – 1.59 (m, 5H), 1.57 – 1.44 (m,3H), 1.34 – 1.13 (m, 4H), 1.07 (s, 3H), 0.65 (s, 3H). **^{13}C NMR** (101 MHz, $CDCl_3$) δ 209.6, 165.8, 141.8, 139.7, 136.1, 129.9, 129.9, 126.0, 122.5, 116.4, 74.4, 63.7, 56.9, 49.9, 44.0, 38.8, 38.2, 37.1, 36.7, 31.9, 31.8, 31.6, 27.9, 24.5, 22.9, 21.1, 19.4, 13.2.

isopropyl 2-methyl-2-(4-(4-vinylbenzoyl)phenoxy)propanoate[4] (b72)

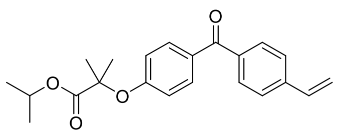

b72

Fenofibrate (10.0 mmol, 1.0 equiv), potassium vinyltrifluoroborate (17.5 mmol, 1.75 equiv), palladium(II) chloride (0.5 mmol, 5 mol%), RuPhos (1.0 mmol, 10 mol%) and Cs_2CO_3 (30.0 mmol, 3.0 equiv) were added to a solution of in THF (10 mL) and water (1 mL). The reaction mixture was degassed by argon sparging for 15 min, and then stirred at 85 ºC in oil bath for 22 h under argon. More water was added and the mixture was extracted three times with EA. The combined organic fractions were washed once with water, once with brine and dried over anhydrous $MgSO_4$, concentrated in vacuum, and the residue was purified by chromatography on silica gel (PE/EA = 9:1) to afford the product as a white solid. (3.42 g, 97%). **^{1}H NMR** (400 MHz, $CDCl_3$) δ 7.84 – 7.67 (m, 4H), 7.49 (d, *J* = 8.3 Hz, 2H), 6.92 – 6.83 (m, 2H), 6.78 (dd, *J* = 17.6, 10.9 Hz, 1H), 5.88 (d, *J* = 17.5 Hz, 1H), 5.39 (dd, *J* = 10.9, 0.8 Hz, 1H), 5.13 – 5.04 (m, 1H), 1.66 (s, 6H), 1.21 (d, *J* = 6.3 Hz, 6H). **^{13}C NMR** (101 MHz, $CDCl_3$) δ 195.0, 173.2, 159.5, 141.1, 137.2, 136.1, 131.9, 130.8, 130.2, 126.0, 117.3, 117.2, 116.3, 79.4, 69.3, 25.4, 21.5.

4-vinylphenyl (*R*)-2-(6-methoxynaphthalen-2-yl)propanoate[7] (b74)

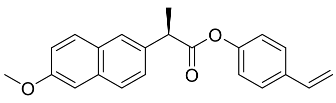

b74

To a solution of 4-vinylphenol (10.0 mmol, 1.0 equiv) in DCM (35 mL) at room temperature were added EDCI·HCl (25.0 mmol, 2.5 equiv), DMAP (1 mmol, 10 mol%), and naproxen (10.0 mmol, 1.0 equiv). The reaction mixture was stirred at room temperature until the disappearance of naproxen as monitored by TLC. The mixture was diluted with water, extracted with DCM (3 × 15 mL), dried over anhydrous Na_2CO_3, and filtered. The solvent was removed under reduced pressure. The residue was purified by chromatography on silica gel (PE/EA = 10:1) to afford the product as a white solid. (2.73 g, 82%). **^{1}H NMR** (400 MHz, $CDCl_3$) δ 7.82 – 7.70 (m, 3H), 7.49 (dd, *J* = 8.5, 1.9 Hz, 1H), 7.40 – 7.31 (m, 2H), 7.20 – 7.09 (m, 2H), 6.99 – 6.90 (m, 2H), 6.66 (dd, *J* = 17.6, 10.9 Hz, 1H), 5.66 (dd, *J* = 17.6, 0.9 Hz, 1H), 5.21 (dd, *J* = 10.9, 0.9 Hz, 1H), 4.09 (q, *J* = 7.1 Hz, 1H), 3.92 (s, 3H), 1.69 (d, *J* = 7.2 Hz, 3H). **^{13}C NMR** (101 MHz, $CDCl_3$) δ 173.2, 157.8, 150.4, 135.9, 135.3, 135.1, 133.8, 129.3, 129.0, 127.4, 127.1, 126.2, 126.1, 121.5, 119.1, 114.0, 105.6, 55.4, 45.6, 18.5.

4-vinylphenyl (1*R*)-4,7,7-trimethyl-3-oxo-2-oxabicyclo[2.2.1]heptane-1-carboxylate[8] (b76)

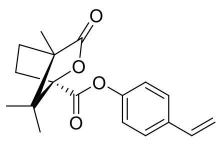

b76

To a solution of 4-vinylphenol (10.0 mmol, 1.0 equiv) and (1*S*)-(-)-camphanic acid (10.0 mmol, 1.0 equiv) in DCM (25 mL), DMAP (1.0 mmol, 10 mol%) and EDCI·HCl (25.0 mmol, 2.5 equiv) was added. The mixture was stirred at room temperature under N_2 until the reaction was complete by TLC monitoring. The mixture was diluted with water (10 mL) and the DCM layer was separated, dried over anhydrous Na_2SO_4 and concentrated. The residue was purified by chromatography on silica gel (PE/EA = 20:1) to afford the product as a white solid. (1.20 g, 40%). **^{1}H NMR** (400 MHz, $CDCl_3$) δ 7.50 – 7.39 (m, 2H), 7.14 – 7.05 (m, 2H), 6.71 (dd, *J* = 17.6, 10.9 Hz, 1H), 5.72 (dd, *J* = 17.6, 0.8 Hz, 1H), 5.27 (dd, *J* = 10.9, 0.8 Hz, 1H), 2.61 – 2.51 (m, 1H), 2.45 – 2.14 (m, 1H), 2.04 – 1.93 (m, 1H), 1.81 – 1.71 (m, 1H), 1.16 (d, *J* = 7.7 Hz, 6H), 1.11 (s, 3H). **^{13}C NMR** (101 MHz, $CDCl_3$) δ 177.8, 166.1, 149.5, 136.0, 135.7, 127.3, 121.4, 114.5, 90.8, 54.9, 54.7, 30.8, 29.0, 16.9, 16.9, 9.7.

4-vinylbenzyl 4-(*N*,*N*-dipropylsulfamoyl)benzoate[5] (b78)

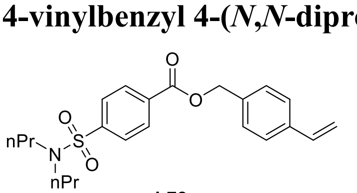

b78

To a stirring suspension of probenecid (10.0 mmol, 1.0 equiv), K_2CO_3 (15.0 mmol, 1.5 equiv) and KI (15.0 mmol, 1.5 equiv) in DMF (20 mL) was added 4-vinylbenzyl chloride (11.0 mmol, 1.1 equiv). The mixture was stirred for 24 h at room temperature, then diluted with water and extracted with EA (20 mL ×3). The combined organic layers were washed for three times with water, and dried over anhydrous Na_2SO_4, and filtered. The solvent was removed under reduced pressure. The residue was purified by chromatography on silica gel (PE/EA = 9:1) to afford the product as a white solid. (3.01 g, 75%). **^{1}H NMR** (400 MHz, $CDCl_3$) δ 8.17 (d, *J* = 8.5 Hz, 2H), 7.87 (d, *J* = 8.5 Hz, 2H), 7.46 – 7.38 (m, 4H), 6.73 (dd, *J* = 17.6, 10.9 Hz, 1H), 5.77 (dd, *J* = 17.6, 0.9 Hz, 1H), 5.37 (s, 2H), 5.28 (dd, *J* = 10.9, 0.9 Hz, 1H), 3.18 – 3.00 (m, 4H), 1.69 – 1.43 (m, 4H), 0.86 (t, *J* = 7.4 Hz,

6H). **^{13}C NMR** (101 MHz, $CDCl_3$) δ 165.1, 144.4, 137.9, 136.3, 134.9, 133.4, 130.3, 128.7, 127.0, 126.5, 114.6, 67.1, 49.9, 21.9, 11.1.

4-vinylbenzyl 2-(4-chlorophenoxy)-2-methylpropanoate[7] (b80)

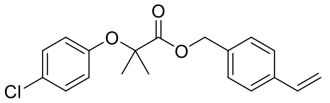

b80

To a stirring suspension of 2-(4-chlorophenoxy) isobutyric acid (10.0 mmol, 1.0 equiv), K_2CO_3 (15.0 mmol, 1.5 equiv) and KI (15.0 mmol, 1.5 equiv) in DMF (20 mL) was added 4-vinylbenzyl chloride (11.0 mmol, 1.1 equiv). The mixture was stirred for 24 h at room temperature, then diluted with water and extracted with EA (20 mL ×3). The combined organic layers were washed for three times with water, and dried over anhydrous Na_2SO_4, and filtered. The solvent was removed under reduced pressure. The residue was purified by chromatography on silica gel (PE/EA = 9:1) to afford the product as a white solid. (2.78 g, 84%). **^{1}H NMR** (400 MHz, $CDCl_3$) δ 7.39 – 7.33 (m, 2H), 7.24 – 7.18 (m, 2H), 7.11 – 7.05 (m, 2H), 6.76 – 6.64 (m, 3H), 5.76 (dd, J = 17.6, 0.9 Hz, 1H), 5.27 (dd, J = 10.9, 0.9 Hz, 1H), 5.16 (s, 2H), 1.58 (s, 6H). **^{13}C NMR** (101 MHz, $CDCl_3$) δ 173.7, 154.0, 137.8, 136.3, 134.7, 129.1, 128.8, 127.2, 126.4, 120.5, 114.6, 79.6, 67.0, 25.4.

4-vinylbenzyl 2-(4-isobutylphenyl)propanoate[8] (b82)

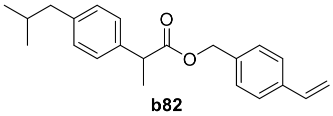

b82

To a stirring suspension of ibuprofen (10.0 mmol, 1.0 equiv), K_2CO_3 (15.0 mmol, 1.5 equiv) and KI (15.0 mmol, 1.5 equiv) in DMF (20 mL) was added 4-vinylbenzyl chloride (11.0 mmol, 1.1 equiv). The mixture was stirred for 24 h at room temperature, then diluted with water and extracted with EA (20 mL ×3). The combined organic layers were washed for three times with water, and dried over anhydrous Na_2SO_4, and filtered. The solvent was removed under reduced pressure. The residue was purified by chromatography on silica gel (PE/EA = 10:1) to afford the product as a white solid. (2.81 g, 87%). **^{1}H NMR** (400 MHz, $CDCl_3$) δ 7.32 (d, J = 8.2 Hz, 2H), 7.18 (dd, J = 10.9, 8.1 Hz, 4H), 7.08 (d, J = 8.2 Hz, 2H), 6.67 (dd, J = 17.6, 10.9 Hz, 1H), 5.71 (dd, J = 17.6, 0.9 Hz, 1H), 5.32 – 5.17 (m, 1H), 5.11 – 5.06 (m, 2H), 3.73 (q, J = 7.2 Hz, 1H), 2.44 (d, J = 7.2 Hz, 2H), 1.92 – 1.72 (m, 1H), 1.50 (d, J = 7.1 Hz, 3H), 0.89 (d, J = 6.6 Hz, 6H). **^{13}C NMR** (101 MHz, $CDCl_3$) δ 174.5, 140.6, 137.7, 137.4, 136.4, 135.7, 129.4, 128.1, 127.3, 126.3, 114.2, 66.1, 45.2, 45.1, 30.3, 22.4, 18.5.

3 Procedure for the Preparation of Co_{SA}-K-PHI Catalysts

3.1 Preparation of K-PHI as photocatalyst carrier

Preparation of $K_{13\%}$-PHI: Melamine (6 g) was thoroughly grinded with NH_4Cl (18 g) and KCl (60 g). The mixture was heated to 550 °C at a rate of 2.3 °C min^{-1} and kept at this temperature for 4 h under nitrogen flow (90 mL/min). After cooling to room temperature, the solid mixture was washed with Deionized water (5 × 9 mL) then dried overnight at 60 °C to obtain final product K-PHI (2.8 g).[9] According to the ICP-OES results, the K content in K-PHI was consistently maintained at 12–14 wt% in different batches.

Table S1. The content of K in sample.

sample	K (wt%)[a]
K-PHI (batch 1)	12.3
K-PHI (batch 2)	13.2
K-PHI (batch 3)	12.7
K-PHI (batch 4)	13.0
K-PHI (batch 5)	13.8

[a]Determined by ICP-OES.

Preparation of $K_{6\%}$-PHI and $K_{3\%}$-PHI: K-PHI (13 wt% K, 400 mg) was treated with 7 mL/g or 11 mL/g aqueous HCl (0.2 M) stirred for 30 min. After that, the product was washed with deionized water several times until pH = 7 and collected by filtration, followed by drying at 60 °C under vacuum. According to the ICP-OES results, the K content in the samples was determined to be 6 wt% and 3 wt%, respectively. The resulting powders were denoted as $K_{6\%}$-PHI and $K_{3\%}$-PHI.

3.2 Preparation of Co_{SA}-K-PHI: K-PHI was added to a solution of $CoCl_2$ in an oven-dried sealed tube equipped with a magnetic stir bar. The mixture was stirred at room temperature for 12 h and centrifuged. The resulting solid was washed with DMF (4 × 5 mL) and a DMF/H_2O (20/1, 4 mL) mixture, then separated by centrifugation and dried overnight in an oven at 60 °C under vacuum. The obtained greenish-yellow powder was denoted as $Co_{x\%}$-$K_{y\%}$-PHI, where x and y indicated the weight percentage of Co and K in the material, respectively. The detailed information is shown below:

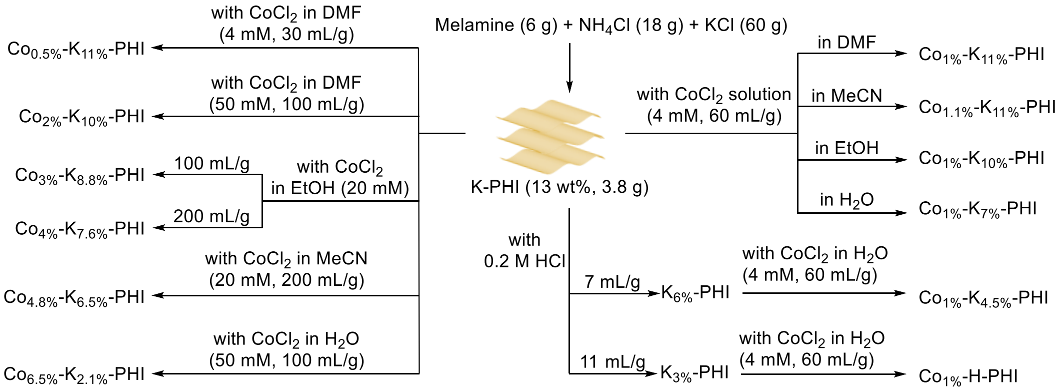

Figure S1. Schematic illustration for the synthesis of Co_{SA}-K-PHI.

Table S2. The content of Co and K in sample.

preparation condition		sample		sample name
K-PHI (mg)[a]	$CoCl_2$ in solvent	Co (wt%)[b]	K (wt%)[b]	
100	50 mM, 10 mL, H_2O	6.5	2.1	$Co_{6.5\%}$-$K_{2.1\%}$-PHI
100	20 mM, 20 mL, MeCN	4.83	6.48	$Co_{4.8\%}$-$K_{6.5\%}$-PHI
100	20 mM, 20 mL, EtOH	4.02	7.6	$Co_{4\%}$-$K_{7.6\%}$-PHI
100	20 mM, 10 mL, EtOH (batch 1)	3.1	8.76	$Co_{3.1\%}$-$K_{8.8\%}$-PHI
100	20 mM, 10 mL, EtOH (batch 2)	2.97	8.8	$Co_{3\%}$-$K_{8.8\%}$-PHI
100	20 mM, 10 mL, EtOH (batch 3)	2.81	9.1	$Co_{2.8\%}$-$K_{9\%}$-PHI
100	15 mM, 10 mL, EtOH (batch 1)	2.33	9.3	$Co_{2.3\%}$-$K_{9.3\%}$-PHI
100	15 mM, 10 mL, EtOH (batch 2)	2.28	10.4	$Co_{2.3\%}$-$K_{10.4\%}$-PHI
100	50 mM, 10 mL, DMF (batch 1)	1.98	10.3	$Co_{2\%}$-$K_{10\%}$-PHI
100	50 mM, 10 mL, DMF (batch 2)	1.93	10.26	$Co_{1.9\%}$-$K_{10\%}$-PHI
100	30 mM, 10 mL, DMF	1.68	10.6	$Co_{1.7\%}$-$K_{10.6\%}$-PHI
100	15 mM, 10 mL, DMF	1.41	10.9	$Co_{1.4\%}$-$K_{11\%}$-PHI
100	4 mM, 6 mL, DMF (batch 1)	1.17	11.2	$Co_{1.2\%}$-$K_{11\%}$-PHI
100	4 mM, 6 mL, DMF (batch 2)	1.03	11.1	$Co_{1\%}$-$K_{11\%}$-PHI
100	4 mM, 6 mL, DMF (batch 3)	0.92	11.4	$Co_{0.9\%}$-$K_{11\%}$-PHI
100	4 mM, 6 mL, MeCN	1.14	11.3	$Co_{1.1\%}$-$K_{11\%}$-PHI
100	4 mM, 6 mL, EtOH	1.04	10.3	$Co_{1\%}$-$K_{10\%}$-PHI
100	4 mM, 3 mL, DMF	0.52	11.3	$Co_{0.5\%}$-$K_{11\%}$-PHI
100	4 mM, 6 mL, H_2O	1.04	6.97	$Co_{1\%}$-$K_{7\%}$-PHI
100[c]	4 mM, 6 mL, H_2O	1	4.5	$Co_{1\%}$-$K_{4.5\%}$-PHI
100[d]	4 mM, 6 mL, H_2O	1.02	-	$Co_{1\%}$-H-PHI

[a]K-PHI (13 wt% K). [b]Determined by ICP-OES. [c]Using K-PHI with a 6 wt% K content as the precursor. [d]Using K-PHI with a 3 wt% K content as the precursor.

Table S3. The content of Co and K in sample prepared with different cobalt salts.

preparation condition		sample		sample name
K-PHI (mg)[a]	metal salt in DMF	Co (wt%)[b]	K (wt%)[b]	
100	$CoCl_2 \cdot 6H_2O$, 4 mM, 6 mL	1.03	11.4	$Co_{1\%}$-$K_{11\%}$-PHI
100	$Co(OAc)_2$, 4 mM, 6 mL	1.2	10.8	$Co_{1.2\%}$-$K_{11\%}$-PHI
100	$Co(acac)_2$, 4 mM, 6 mL	0.83	11.3	$Co_{0.8\%}$-$K_{11\%}$-PHI

[a]K-PHI (12.7 wt% K). [b]Determined by ICP-OES.

3.3 Preparation of M-K-PHI (M = Fe, Ni, Cu): Ni-K-PHI, Cu-K-PHI, and Fe-K-PHI were prepared with the same procedure for the syntheisis of Co-K-PHI. The only difference was use of $NiCl_2 \cdot 6H_2O$, $CuCl_2$, and $FeCl_3$ as the metal salt to replace the $CoCl_2$.

Table S4. The content of metal in sample prepared with different metal salts.

preparation condition		sample		sample name
K-PHI (mg)[a]	metal salt in DMF	M (wt%)[b]	K (wt%)[b]	
100	$NiCl_2 \cdot 6H_2O$, 6 mM, 3 mL	0.8	12.2	$Ni_{0.8\%}$-$K_{12.2\%}$-PHI
100	$CuCl_2$, 6 mM, 3 mL	1	11.7	$Cu_{1\%}$-$K_{11.7\%}$-PHI
100	$FeCl_3$, 6 mM, 3 mL	0.9	11.2	$Fe_{0.9\%}$-$K_{11.2\%}$-PHI

[a]K-PHI (14 wt% K). [b]Determined by ICP-OES.

3.4 Preparation of Co-modified carbon nitride[10]: Dicyandiamide (3 g, 36 mmol) mixed with deionized water (15 mL) was heated and stirred at 100 °C with 2 mmol of $CoCl_2$ added. The mixed solution was continually heated at 100 °C to remove water. The resulting mixtures were then heated at 550 °C for 4 h under nitrogen flow (90 mL/min). The sample was then cooled to room temperature under nitrogen atmosphere. According to the ICP-OES results, the content of Co in material was determined to be 0.9 wt %, The sample was denoted as $Co_{0.9\%}$-CN.

3.5 Preparation of protonated poly-(heptazine imide): K-PHI (200 mg) was treated with 1 M HCl (4 mL) stirred for 45 min. Every 15 min acid solution was changed with a fresh portion. After that, the product was washed with deionized water several times until pH = 7 and collected by filtration, followed by drying at 60 °C under vacuum. This sample is denoted as H-PHI.

4 Characterization of Co_{SA}-K-PHI

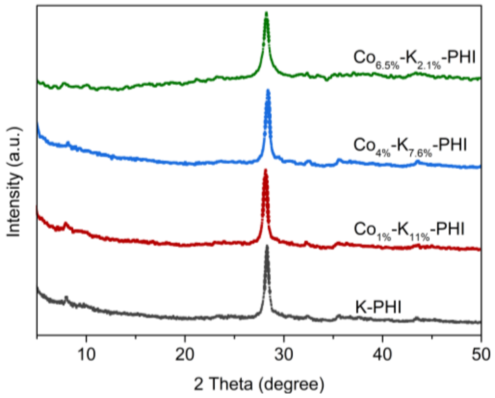

Figure S2. XRD patterns.

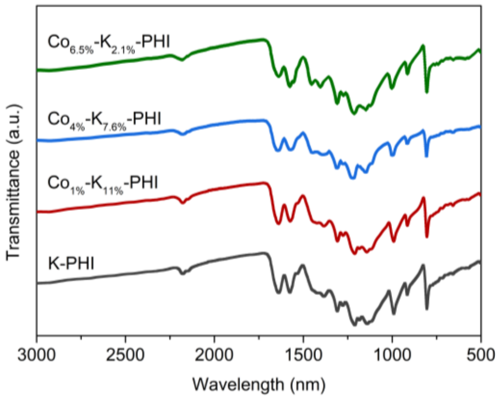

Figure S3. FTIR spectra.

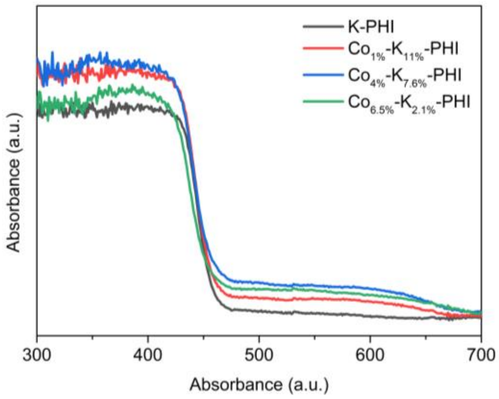

Figure S4. UV-vis spectra.

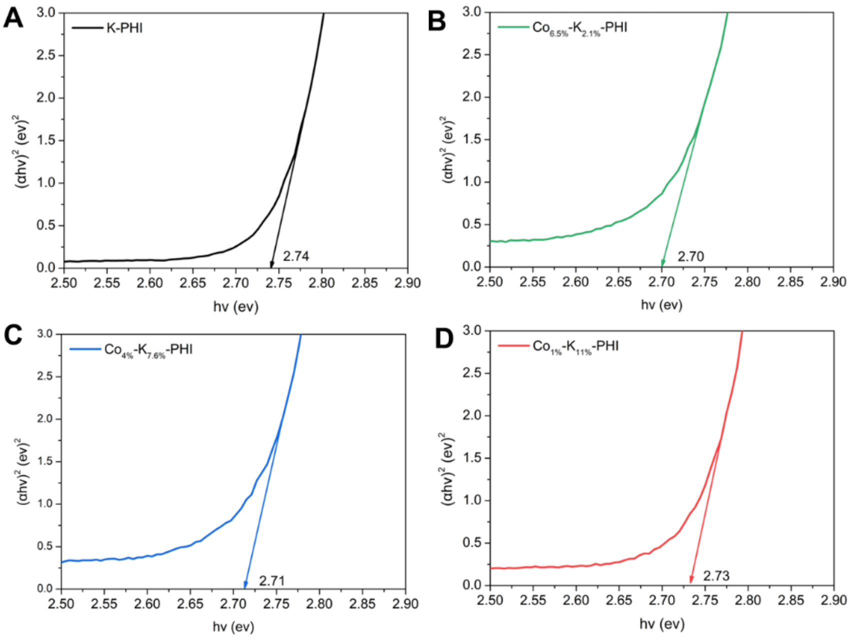

Figure S5. Tauc plots of K-PHI (A), $Co_{6.5\%}$-$K_{2.1\%}$-PHI (B), $Co_{4\%}$-$K_{7.6\%}$-PHI (C), and $Co_{1\%}$-$K_{11\%}$-PHI (D).

The band gaps of materials were calculated from the curve of photon energy by $(ahv)^2$. As can be seen from Figure S5, the values of K-PHI, $Co_{6.5\%}$-$K_{2.1\%}$-PHI, $Co_{4\%}$-$K_{7.6\%}$-PHI, and $Co_{1\%}$-$K_{11\%}$-PHI are 2.74 eV, 2.70 eV, 2.71 eV and 2.73 eV, respectively.

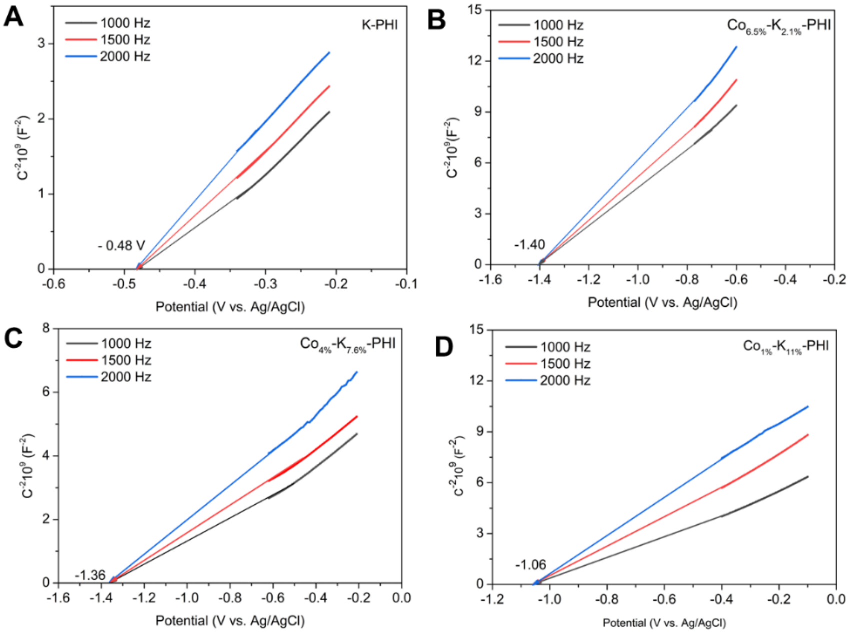

Figure S6. Mott-Schottky plots of K-PHI (A), $Co_{6.5\%}$-$K_{2.1\%}$-PHI (B), $Co_{4\%}$-$K_{7.6\%}$-PHI (C), and $Co_{1\%}$-$K_{11\%}$-PHI (D), Experimental conditions: 0.2 M Na_2SO_4, pH 6.6.

The flat band potentials of the catalyst were measured by Mott-Schottky plots at frequencies of 1000, 1500, and 2000 Hz (Figure S6). The measurement processes were performed in 0.2 M Na_2SO_4 aqueous solution, Ag/AgCl electrode (saturated KCl solution) and platinum wire were used as reference electrode and counter electrode,

respectively. The working electrode was prepared on indium-tin oxide (ITO) glass that was cleaned by sonication in deionized (DI) water for 30 min and dried at 353 K. 5 mg sample was dispersed in 1 mL EtOH with sonication to get the slurry. The slurry and nafion (5 wt %, 10 μL) were spread onto a 2 cm × 1 cm pretreated ITO glass and air-drying.

The intercept of the curve on the abscissa is the flat band potential of the sample and its values (vs. Ag/AgCl) are -0.47 V, -1.40 V, -1.36 V, and -1.06 V of K-PHI, $Co_{6.5\%}$-$K_{2.1\%}$-PHI, $Co_{4\%}$-$K_{7.6\%}$-PHI, and $Co_{1\%}$-$K_{11\%}$-PHI. The slope of the curve is positive, which reveal the sample is n-type semiconductors. For n-type semiconductor, the flat band potential is about 0.3 V below the conduction band (CB) potential.[11-13] According to the equation of:

$$E_{vs\ SCE} = E_{vs\ Ag/AgCl} - 0.042\ V$$

All potentials were converted to the standard calomel electrode (SCE). Therefore, the conduction band (CB) potentials of K-PHI, $Co_{6.5\%}$-$K_{2.1\%}$-PHI, $Co_{4\%}$-$K_{7.6\%}$-PHI, and $Co_{1\%}$-$K_{11\%}$-PHI are -0.82 V, -1.74 V, -1.71 V, and -1.40 V (vs. SCE), respectively.

5 Optimization of the Reaction Conditions and Control Experiments

Table S5. Evaluation of catalyst.

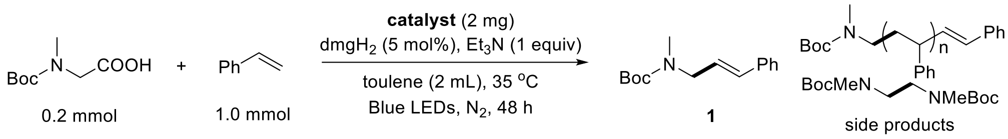

entry[a]	catalyst	M (mol%)	conv.[b] (%)	yield[b] (**1**, %)
1	$Co_{6.5\%}$-$K_{2.1\%}$-PHI	1.11	0	0
2	$Co_{4.8\%}$-$K_{6.5\%}$-PHI	0.82	0	0
3	$Co_{4\%}$-$K_{7.6\%}$-PHI	0.68	0	0
4	$Co_{3.1\%}$-$K_{8.8\%}$-PHI	0.53	13	8
5	$Co_{3\%}$-$K_{8.8\%}$-PHI	0.51	12	10
6	$Co_{2.8\%}$-$K_{9\%}$-PHI	0.48	14	11
7	$Co_{2.3\%}$-$K_{10.4\%}$-PHI	0.39	59	50
8	$Co_{2.3\%}$-$K_{9.3\%}$-PHI	0.39	26	20
9	K-PHI	-	80	0
10	$Co_{2\%}$-$K_{10\%}$-PHI	0.34	100	78
11	$Co_{1.9\%}$-$K_{10\%}$-PHI	0.32	100	76
12	$Co_{1.7\%}$-$K_{10.6\%}$-PHI	0.29	100	78
13	$Co_{1.4\%}$-$K_{11\%}$-PHI	0.24	100	77
14	$Co_{1.2\%}$-$K_{11\%}$-PHI	0.20	100	78
15	$Co_{1\%}$-$K_{11\%}$-PHI	0.17	100	78 (75)[c]
16	$Co_{0.9\%}$-$K_{11\%}$-PHI	0.15	100	77
17	$Co_{0.5\%}$-$K_{11\%}$-PHI	0.085	51	25
18[d]	$Co_{0.5\%}$-$K_{11\%}$-PHI	0.085	100	48
19	$Co_{1\%}$-$K_{7\%}$-PHI	0.17	83	41
20	$Co_{1\%}$-$K_{4.5\%}$-PHI	0.17	40	0
21	$Co_{1\%}$-H-PHI	0.17	20	0
22	$Fe_{0.9\%}$-$K_{11.2\%}$-PHI	0.18	100	0
23	$Cu_{1\%}$-$K_{11.7\%}$-PHI	0.16	100	0
24	$Ni_{0.8\%}$-$K_{12.2\%}$-PHI	0.17	100	0
25[e]	$Co_{1\%}$-$K_{11\%}$-PHI	0.07	100	70
26	H-PHI (2 mg) + $CoCl_2$ (0.17 mol%)	-	36	0
27	H-PHI (2 mg) + $CoCl_2$ (1 mol%)	-	38	0
28	K-PHI (2 mg) + $CoCl_2$ (0.17 mol%)	-	100	53
29	Mes-Acr-$Ph^+BF_4^-$ (4 mol%) + $CoCl_2$ (0.17 mol%)	-	100	9
30	Mes-Acr-$Ph^+BF_4^-$ (4 mol%) + $CoCl_2$ (0.5 mol%)	-	100	30
31	Mes-Acr-$Ph^+BF_4^-$ (4 mol%) + $CoCl_2$ (1 mol%)	-	100	50
32	Mes-Acr-$Ph^+BF_4^-$ (4 mol%) + $CoCl_2$ (2 mol%)	-	100	64
33	Mes-Acr-$Ph^+BF_4^-$ (4 mol%) + $CoCl_2$ (5 mol%)	-	100	70
34	Mes-Acr-$Ph^+BF_4^-$ (2 mol%) + $CoCl_2$ (5 mol%)	-	58	16

[a]*N*-Boc-*N*-methylglycine (0.2 mmol, 1.0 equiv), styrene (1.0 mmol, 5.0 equiv), Et_3N (0.2 mmol, 1.0

equiv), $dmgH_2$ (5 mol%) and catalyst in toluene (2 mL) under N_2 atmosphere and blue LEDs irradiation (24 W, 460 ± 5 nm) without extra heating (at 35 ± 5 °C) for 48 h. [b]Determined by ^{1}H NMR analysis using trimethyl benzene-1,3,5-tricarboxylate as an internal standard. [c]Isolated yield in parenthesis. [d]96 h. [e]Reaction was conducted on a 2 mmol scale with 8 mg of catalyst for 96 h.

Note: Different batches of $Co_{1\%}$-$K_{11\%}$-PHI catalysts show similar catalytic activity for the reaction (+/-3% based on ^{1}H-NMR with internal standard).

Table S6. Evaluation of solvent.

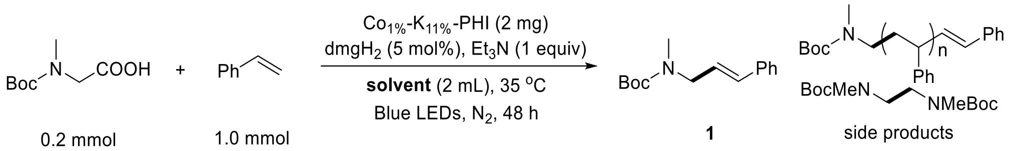

entry[a]	solvent	conv.[b] (%)	yield[b] (**1**, %)
1	toluene	100	78
2	PhCl	100	69
3	MeCN	80	29
4	DCM	11	trace
5	THF	75	20
6	DMF	100	0

[a]*N*-Boc-*N*-methylglycine (0.2 mmol, 1.0 equiv), styrene (1.0 mmol, 5.0 equiv), Et_3N (0.2 mmol, 1.0 equiv), $dmgH_2$ (5 mol%) and $Co_{1\%}$-$K_{11\%}$-PHI (2 mg, 0.17 mol% Co) in solvent (2 mL) under N_2 atmosphere and blue LEDs irradiation (24 W, 460 ± 5 nm) without extra heating (at 35 ± 5 °C) for 48 h. [b]Determined by ^{1}H NMR analysis using trimethyl benzene-1,3,5-tricarboxylate as an internal standard.

Table S7. Evaluation of base.

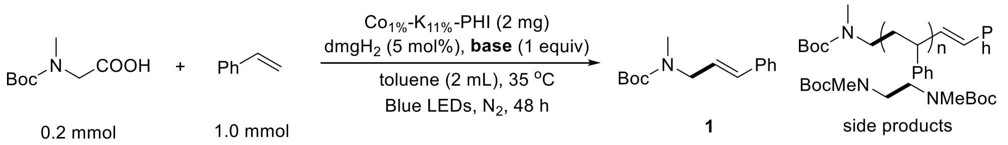

entry[a]	base	conv.[b] (%)	yield[b] (**1**, %)
1	Et_3N	100	78
2	pyridine	100	73
3	DMAP	100	75
4	CsF	100	25
5	Na_2CO_3	100	30
6	NaOAc	100	50
7	K_3PO_4	100	8
8	Et_3N (0.5 equiv)	100	76
9	Et_3N (2 equiv)	100	77

[a]*N*-Boc-*N*-methylglycine (0.2 mmol, 1.0 equiv), styrene (1.0 mmol, 5.0 equiv), base (0.2 mmol, 1.0 equiv), $dmgH_2$ (5 mol%) and $Co_{1\%}$-$K_{11\%}$-PHI (2 mg, 0.17 mol% Co) in toluene (2 mL) under N_2

atmosphere and blue LEDs irradiation (24 W, 460 ± 5 nm) without extra heating (at 35 ± 5 °C) for 48 h. [b]Determined by ^{1}H NMR analysis using trimethyl benzene-1,3,5-tricarboxylate as an internal standard.

Table S8. Evaluation of ligand

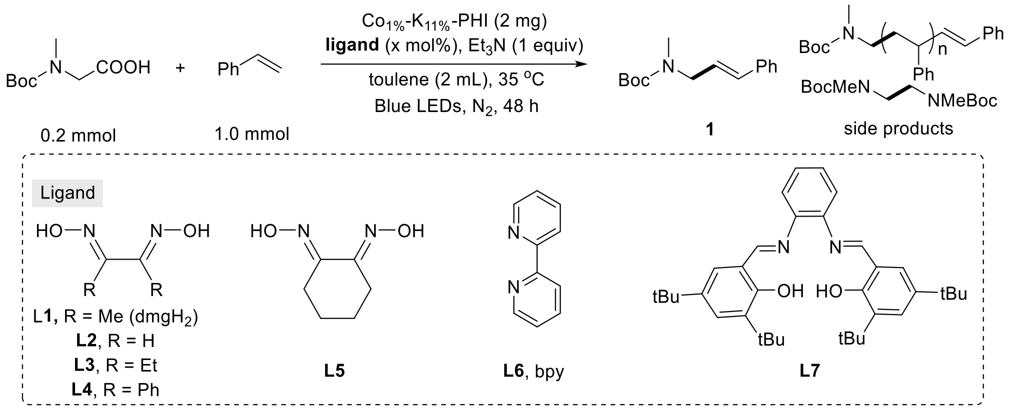

entry[a]	ligand	x (mol%)	conv.[b] (%)	yield[b] (**1**, %)
1	**L1**	5	100	78
2	**L2**	5	100	10
3	**L3**	5	100	72
4	**L4**	5	100	77
5	**L5**	5	65	51
6[c]	**L5**	5	100	65
7	**L6**	5	100	0
8	**L7**	5	100	0
9	**L1**	1	100	32
10	**L1**	2	100	60
11	**L1**	10	100	76

[a]*N*-Boc-*N*-methylglycine (0.2 mmol, 1.0 equiv), styrene (1.0 mmol, 5.0 equiv), Et_3N (0.2 mmol, 1.0 equiv), ligand (x mol%) and $Co_{1\%}$-$K_{11\%}$-PHI (0.17 mol% Co) in toluene (2 mL) under N_2 atmosphere and blue LEDs irradiation (24 W, 460 ± 5 nm) without extra heating (at 35 ± 5 °C) for 48 h. [b]Determined by ^{1}H NMR analysis using trimethyl benzene-1,3,5-tricarboxylate as an internal standard. [c]72 h.

Table S9. Evaluation of the amount of styrene with *N*-Boc-*N*-methylglycine as the limiting reagent.

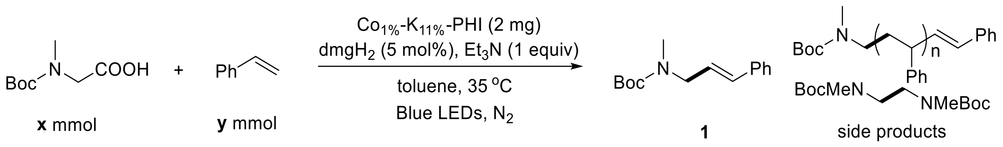

entry[a]	x (mmol)	y (mmol)	conv.[b] (%)	yield[b] (**1**, %)
1	0.2	0.2	100	33
2	0.2	0.4	100	50
3	0.2	1.0	100	78
4	0.2	2.0	100	84
5	0.4	0.2	50	11

[a]*N*-Boc-*N*-methylglycine (x mmol), styrene (y mmol), Et_3N (1.0 equiv), $dmgH_2$ (5 mol%) and $Co_{1\%}$-$K_{11\%}$-PHI (2 mg) in toluene (2 mL) under N_2 atmosphere and blue LEDs irradiation (24 W, 460 ± 5 nm) without extra heating (at 35 ± 5 °C) for 48 h. [b]Determined by ^1H NMR analysis using trimethyl benzene-1,3,5-tricarboxylate as an internal standard.

Table S10. Control experiments.

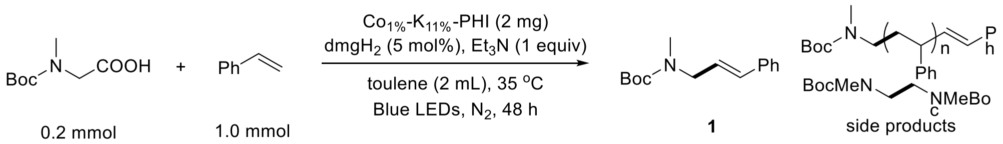

entry[a]	variation from standard condition	conv.[b] (%)	yield[b] (**1**, %)
1	none	100	78
2	no $dmgH_2$	100	0
3	no EtN_3	75	8
4	no $Co_{1\%}$-$K_{11\%}$-PHI	0	0
5	no light	0	0

[a]*N*-Boc-*N*-methylglycine (0.2 mmol, 1.0 equiv), styrene (1.0 mmol, 5.0 equiv), Et_3N (0.2 mmol, 1.0 equiv), $dmgH_2$ (5 mol%) and $Co_{1\%}$-$K_{11\%}$-PHI (2 mg, 0.17 mol% Co) in toluene (2 mL) under N_2 atmosphere and blue LEDs irradiation (24 W, 460 ± 5 nm) without extra heating (at 35 ± 5 °C) for 48 h. [b]Determined by ^1H NMR analysis using trimethyl benzene-1,3,5-tricarboxylate as an internal standard.

6 Experimental Procedure

6.1 Decarboxylation-Initiated Styrene Polymerization and Alkyl Radical Dimerization

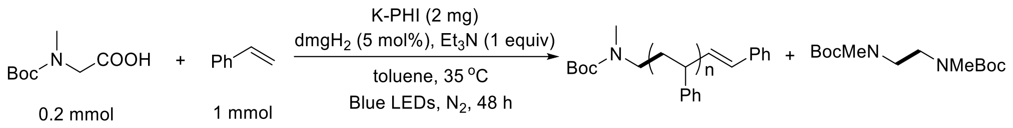

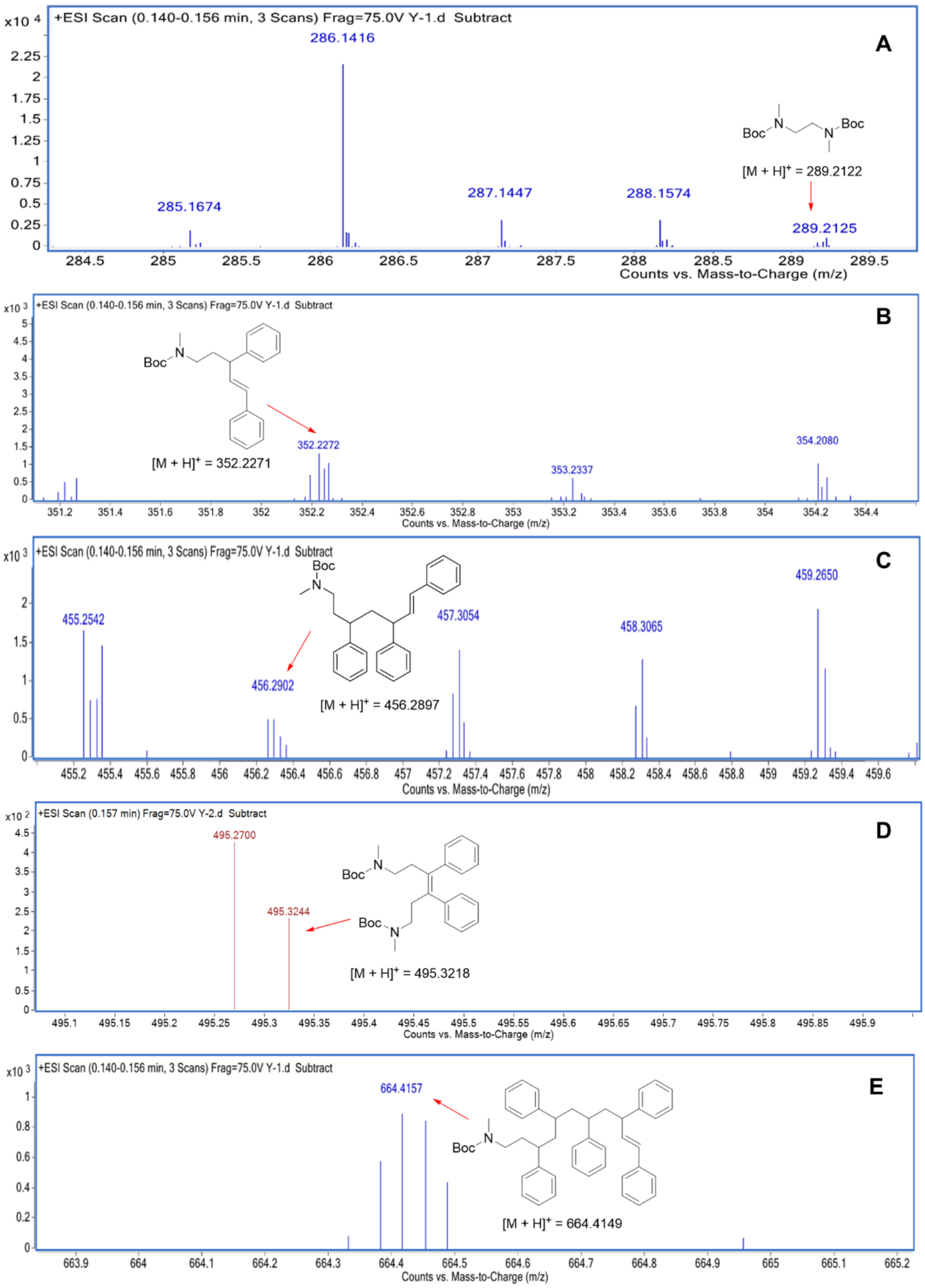

Figure S7. HRMS spectra of the side products.

6.2 Decarboxylative Radical Addition of *N*-Boc-*N*-methylglycine to *p*-QM

Table S11. Evaluate the decarboxylation activities of different catalysts

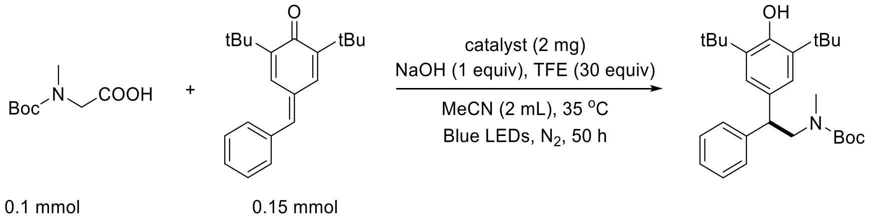

entry[a]	catalyst	conv.[b] (%)	yield[b] (**1**, %)
1	K-PHI	100	99
2	$Co_{1\%}$-$K_{11\%}$-PHI	64	63
3	$Co_{2\%}$-$K_{10\%}$-PHI	53	50
4	$Co_{3\%}$-$K_{8.8\%}$-PHI	10	8
5	$Co_{4\%}$-$K_{7.6\%}$-PHI	0	0
6	$Co_{6.5\%}$-$K_{2.1\%}$-PHI	0	0

[a]*N*-Boc-*N*-methylglycine (0.1 mmol, 1.0 equiv), 4-benzylidene-2,6-di-*tert*-butylcyclohexa-2,5-dien-1-one (0.15 mmol, 1.5 equiv), NaOH (0.1 mmol, 1.0 equiv), TFE (30 equiv) and catalyst (2 mg) in MeCN (2 mL) under N_2 atmosphere and blue LEDs irradiation (24 W, 460 ± 5 nm) without extra heating (at 35 ± 5 °C) for 50 h. [b]Determined by ^1H NMR analysis using 1,3,5-trimethoxybenzene as an internal standard.

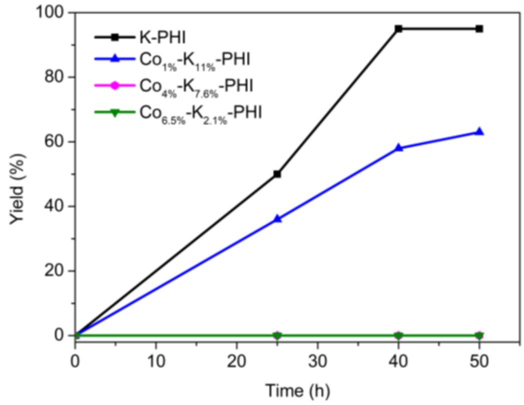

Figure S8. Comparison of the decarboxylation catalytic activity of different catalysts.

To a 10 mL oven-dried sealed tube equipped with a magnetic stir bar was added *N*-Boc-*N*-methylglycine (0.1 mmol, 1.0 equiv), 4-benzylidene-2,6-di-*tert*-butylcyclohexa-2,5-dien-1-one (0.15 mmol, 1.5 equiv), NaOH (0.1 mmol, 1.0 equiv), and catalyst (2.0 mg). It was capped with a rubber septum, evacuated and backfilled with argon. Then, degassed MeCN (2 mL) and TFE (30 equiv) were added via syringe. The reaction mixture was stirred and irradiated by blue LEDs (24 W, 460 ± 5 nm) without extra heating (35 ± 5 °C) the indicated time. Then the reaction mixture was concentrated under reduced pressure to evaporate the solvent, and the crude residue was purified by silica gel column chromatography. The final product was characterized by ^1H-NMR, ^{13}C-NMR and HRMS.

***tert*-butyl (2-(3,5-di-*tert*-butyl-4-hydroxyphenyl)-2-phenylethyl)(methyl)carbamate**

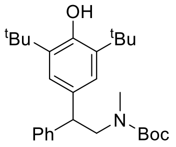

White solid. mp = 132 – 134 °C. **^{1}H NMR** (400 MHz, $CDCl_3$) δ 7.32 – 7.25 (m, 4H), 7.23 – 7.13 (m, 1H), 7.04 (d, *J* = 21.0 Hz, 2H), 5.06 (d, *J* = 9.3 Hz, 1H), 4.29 – 4.13 (m, 7.9 Hz, 1H), 4.01 – 3.80 (m, 1H), 3.67 (dd, *J* = 13.8, 7.4 Hz, 1H), 2.63 (d, *J* = 28.2 Hz, 3H), 1.39 (d, *J* = 8.5 Hz, 27H). **^{13}C NMR** (101 MHz, $CDCl_3$) δ 155.6, 152.4, 142.8, 142.5, 135.7, 132.7, 128.4, 128.4, 128.3, 126.5, 126.2, 124.7, 79.3, 79.1, 55.0, 53.6, 50.0, 49.4, 34.9, 34.7, 34.4, 30.3, 28.4. **HRMS (ESI)** m/z calcd. For $C_{28}H_{42}NO_3$ $[M+H]^+$ 440.3159, found 440.3155.

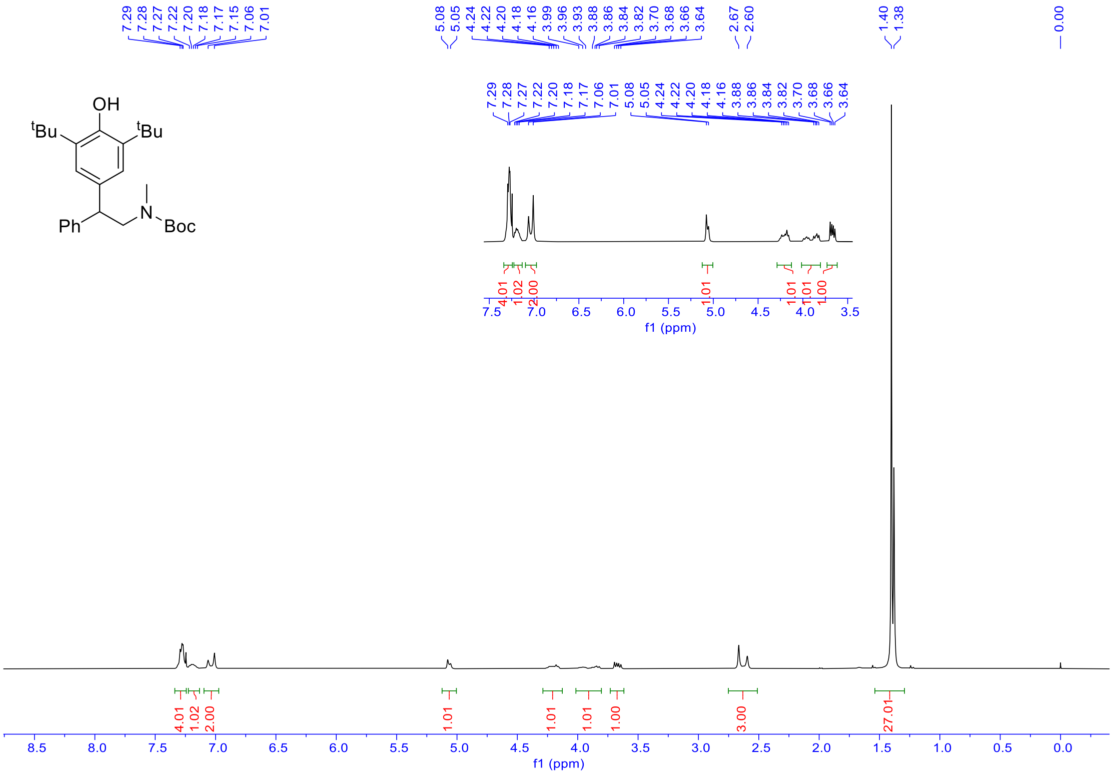

Figure S9 ^{1}H NMR spectra (400 MHz, $CDCl_3$)

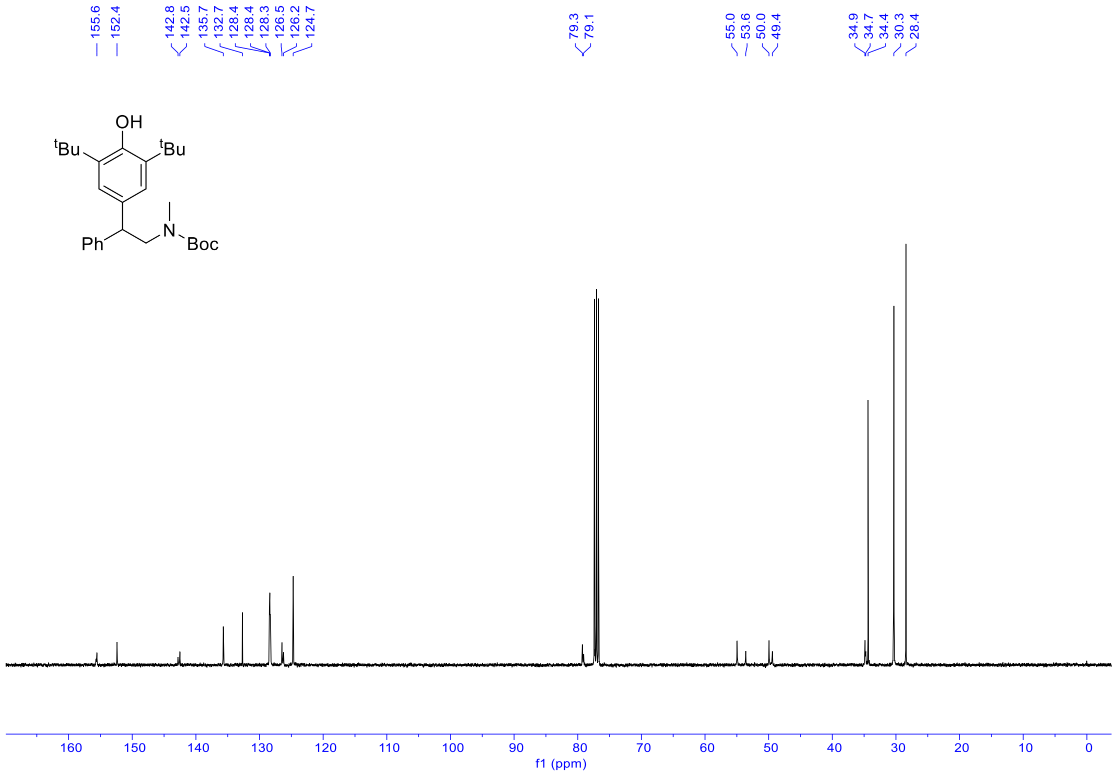

Figure S10 ^{13}C NMR spectra (101 MHz, $CDCl_3$)

6.3 Catalyst Recycling

After completion of the reaction, the reaction mixture was centrifuged at 10000 rpm for 10 min to separate $Co_{1\%}$-$K_{11\%}$-PHI and the liquid mixture. The recovered $Co_{1\%}$-$K_{11\%}$-PHI was washed with toluene (2 × 1 mL), centrifuged and dried in a high vacuum. Then the recovered $Co_{1\%}$-$K_{11\%}$-PHI was suspended in toluene (0.5 mL) and transferred to a new reaction tube. The falcon tube was washed with toluene (2 × 0.5 mL) to ensure the complete transfer of the heterogeneous material and used for the next cycle. Afterward, the reactants, Et_3N, fresh $dmgH_2$, and toluene (0.5 mL) were added. The tube was closed with a rubber septum and the reaction mixture was degassed by three cycles vacuum/N_2 of "freeze-pump-thaw". Then the reaction mixture was stirred and irradiated by blue LEDs (4 × 24 W, 460 ± 5 nm) without extra heating (35 ± 5 °C) for the indicated time. An independent fan was used to maintain the temperature inside the irradiation reaction system.

Table S12. Amount of cobalt in recovered $Co_{1\%}$-$K_{11\%}$-PHI

entry	metallaphotocatalyst	Co (wt%)[a]
1	fresh $Co_{1\%}$-$K_{11\%}$-PHI	1
2	1st recovered $Co_{1\%}$-$K_{11\%}$-PHI	0.93
3	3rd recovered $Co_{1\%}$-$K_{11\%}$-PHI	0.90
4	5th recovered $Co_{1\%}$-$K_{11\%}$-PHI	0.85

[a]Determined by ICP-OES.

Table S13. Control experiment with recovered Co1%-K11%-PHI[a]

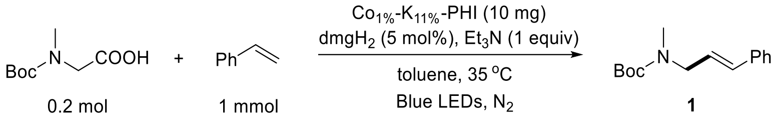

cycle	catalyst (10 mg)	$dmgH_2$ (mol%)	yield[b] (**1**, %)
1	fresh Co1%-K11%-PHI	5	75
2	1st recovered Co1%-K11%-PHI	-	38
3	2nd recovered Co1%-K11%-PHI	-	0
4	3rd recovered Co1%-K11%-PHI	5	72

[a]*N*-Boc-*N*-methylglycine (0.2 mmol, 1.0 equiv), styrene (1.0 mmol, 5.0 equiv), Et_3N (0.2 mmol, 1.0 equiv), $dmgH_2$ (5 mol%) and catalyst in toluene (2 mL) under N_2 atmosphere and blue LEDs irradiation (24 W, 460 ± 5 nm) without extra heating (at 35 ± 5 °C) for 36 h. [b]Isolated yield.

Note: In order to ensure effective recovery of the heterogeneous catalyst for reuse experiments, a loading of 10 mg of catalyst was used, which is higher than the typical amount in standard reactions, facilitating reliable separation and minimization of material loss.

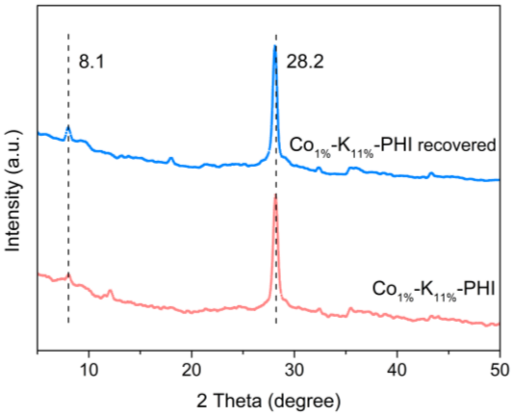

Figure S11. XRD patterns of Co1%-K11%-PHI and recovered Co1%-K11%-PHI.

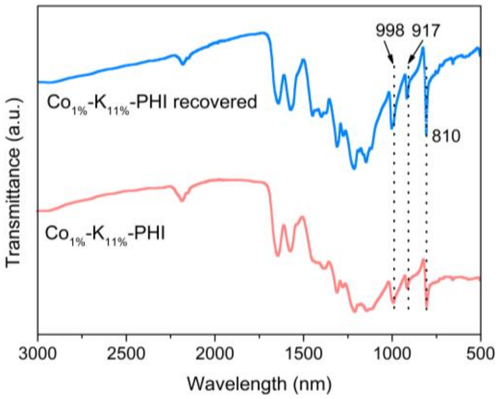

Figure S12. FTIR spectra of Co1%-K11%-PHI and recovered Co1%-K11%-PHI.

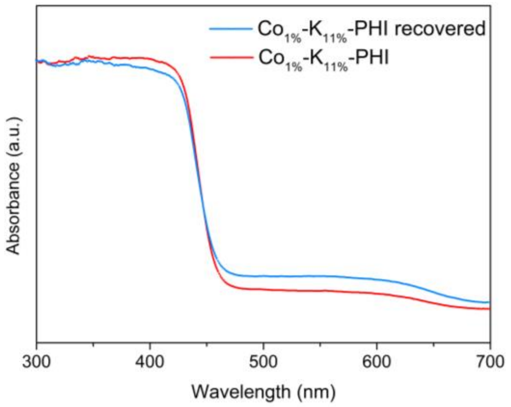

Figure S13. UV-vis spectra of $Co_{1\%}$-$K_{11\%}$-PHI and recovered $Co_{1\%}$-$K_{11\%}$-PHI.

6.4 Structural Characterization of Atomically Dispersed Cobalt Catalyst.

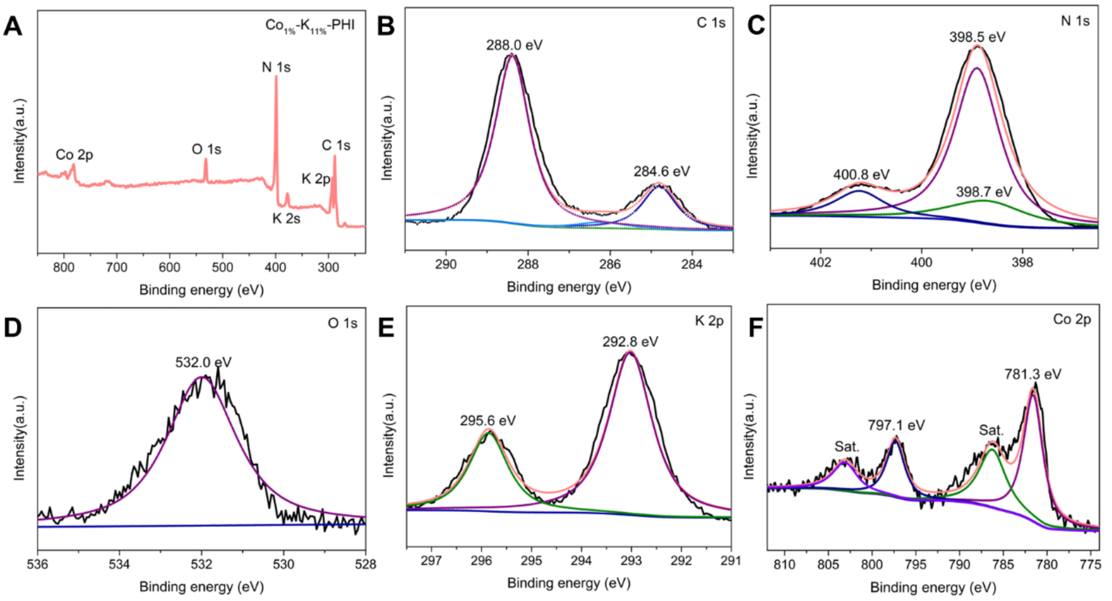

Figure S14. XPS spectra of $Co_{1\%}$-$K_{11\%}$-PHI: (A) survey spectrum, (B) C 1s, (C) N 1s, (D) O 1s, (E) K 2p and (F) Co 2p high-resolution spectra.

Table S14. Fitting results of Co K-edge FT-EXAFS curves

Sample	Path	CN^a	R(Å)^b	σ^2 (Å^2)^c	ΔE_0(eV)^d	R factor	*Range of k*	*Range of R*
Co K-edge (S_0^2=0.838)								
Co foil	Co-Co	12.0*	2.492	0.0062	7.6	0.0010	3.0-13.9	1.0-3.0
CoO	Co-O	6.0	2.106	0.0101	-3.0	0.0143	3.0-11.0	1.0-3.2
	Co-Co	12.0	3.004	0.0098	-4.0			
$Co_{1\%}$-$K_{11\%}$-PHI	Co-N	3.9	2.023	0.0051	0.4	0.0141	3.0-9.0	1.2-2.0
	Co-O	1.9	2.145					

$^a CN$, coordination number; $^b R$, the distance between absorber and backscatter atoms; $^c\sigma^2$, the Debye Waller factor value; $^d\Delta E_0$, inner potential correction to account for the

difference in the inner potential between the sample and the reference compound; *R* factor indicates the goodness of the fit. S_0^2 was fixed to 0.838, according to the experimental EXAFS fit of Co foil by fixing *CN* as the known crystallographic value. * This value was fixed during EXAFS fitting, based on the known structure of Co. fitting space: R space; *k*-weight = 3. A reasonable range of EXAFS fitting parameters: $0.700 < S_0^2 < 1.000$; $CN > 0$; $\sigma^2 > 0$ Å^2; $|\Delta E_0| < 15$ eV; *R* factor < 0.02.

6.5 General Procedure for Co$_{1\%}$-K$_{11\%}$-PHI Catalyzed Decarboxylative Heck Reaction

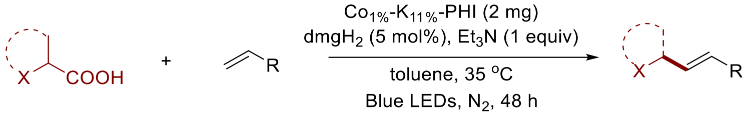

To a 10 mL oven-dried sealed tube equipped with a magnetic stir bar was added the corresponding acid (0.2 mmol, 1.0 equiv), dmgH$_2$ (5 mol%) and Co$_{1\%}$-K$_{11\%}$-PHI (2.0 mg, 0.17 mol% Co), Et$_3$N (0.2 mmol, 1.0 equiv), and dry toluene (2.0 mL). The tube was closed with a rubber septum and the reaction mixture was degassed by three cycles vacuum/N$_2$ of "freeze-pump-thaw". Subsequently, the alkene (1 mmol, 5.0 equiv) was added via syringe. The reaction mixture was stirred and irradiated by blue LEDs (24 W, 460 ± 5 nm) without extra heating (35 ± 5 °C) for 48 h. In each case, the blue LEDs was placed 3 cm from the reaction tube (Figure S15). An independent fan was used to maintain the temperature inside the irradiation reaction system. Upon completion, the reaction mixture was concentrated under reduced pressure to evaporate the solvent, and the crude residue was purified by silica gel column chromatography. The final product was characterized by ^{1}H-NMR, ^{13}C-NMR and HRMS.

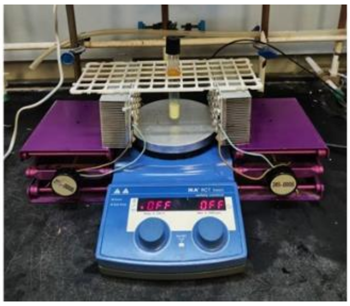

Figure S15. Photograph for the photochemical reaction set-up

6.6 Substrate Scope

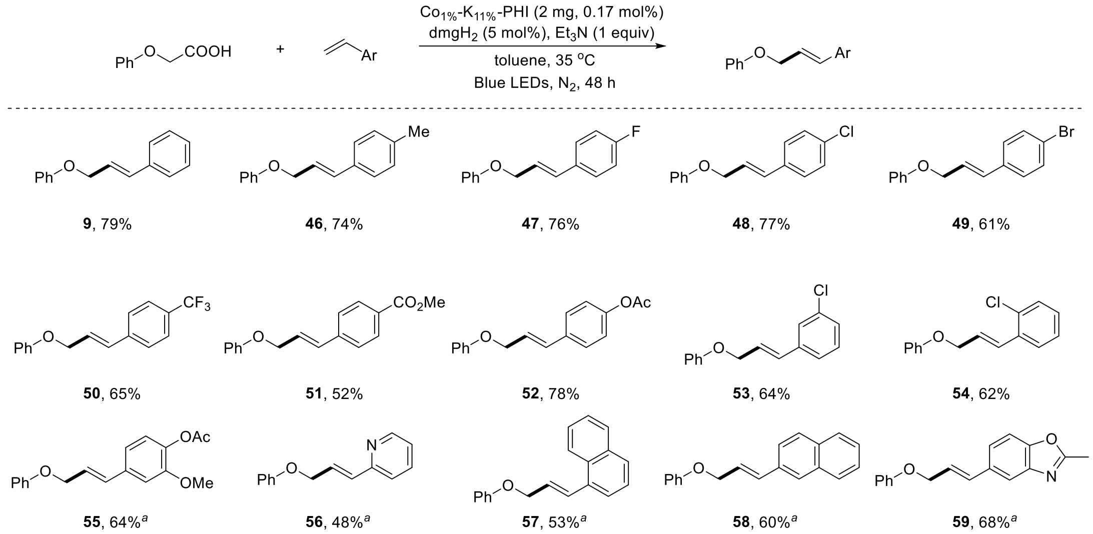

Figure S16. Substrate scope of alkenes with phenoxyacetic acid for decarboxylative Heck reaction. Reaction conditions: phenoxyacetic acid (0.2 mmol), $Co_{1\%}$-$K_{11\%}$-PHI (2 mg, 0.17 mol%), alkene (0.4 mmol, 2.0 equiv), Et_3N (0.2 mmol, 1.0 equiv) and $dmgH_2$ (5 mol%) in toluene (2 mL) under N_2 atmosphere and blue LEDs irradiation (24 W, 460 ± 5 nm) without extra heating (at 35 ± 5 °C). [a]Performed with 5 equiv of alkene.

6.7 Gram-Scale Experiment

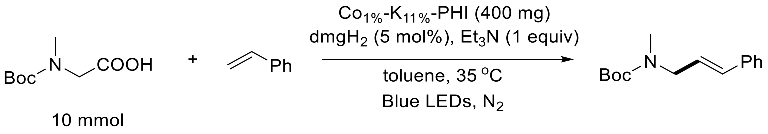

To a 250 mL oven-dried Schlenk tube equipped with a magnetic stir bar was added *N*-Boc-*N*-methylglycine (10 mmol, 1.0 equiv), $dmgH_2$ (10 mol%) and $Co_{1\%}$-$K_{11\%}$-PHI (400 mg), Et_3N (10 mmol, 1.0 equiv), and dry toluene (100 mL). Subsequently, the styrene (50 mmol, 5.0 equiv) was added via syringe. The resulting mixture was degassed via ‘freeze-pump-thaw’ procedure (3 times). Then the reaction mixture was stirred and irradiated by blue LEDs (2 × 24 W, 460 ± 5 nm) without extra heating (35 ± 5 °C) for 96 h. The blue LEDs was placed 5 cm from the reaction tube (Figure S17). An independent fan was used to maintain the temperature inside the irradiation reaction system. Upon completion, the reaction mixture was concentrated under reduced pressure to evaporate the solvent, and the crude residue was purified by silica gel column chromatography to afford product **1** (1.83 g, 74% yield).

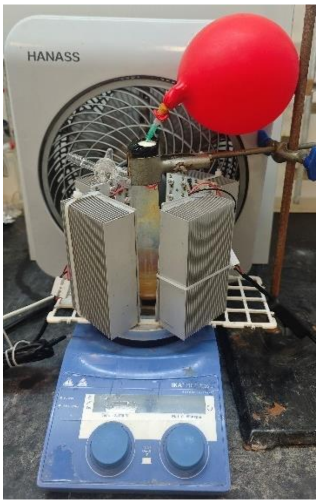

Figure S17. Photograph for the photochemical reaction set-up

7 Mechanistic Investigations

7.1 Stern−Volmer Quenching Studies.

Stern−Volmer quenching studies were carried out using a 0.1 mg/mL solution of photocatalyst $Co_{1\%}$-$K_{11\%}$PHI and variable concentrations (0, 0.5, 1.0, 1.5, 2.0 mM) of the combination of *N*-Boc-*N*-methylglycine and Et_3N, the combination of *N*-Boc-*N*-methylglycine, Et_3N, and $dmgH_2$, styrene, *N*-Boc-*N*-methylglycine, or Et_3N in degassed toluene (via three freeze-pump-thaw cycles). The intensity of the emission peak at 467 nm (λex = 350 nm) for $Co_{1\%}$-$K_{11\%}$-PHI expressed as the ratio I_0/I, where I_0 is the emission intensity of $Co_{1\%}$-$K_{11\%}$-PHI at 467 nm in the absence of a quencher and I is the observed intensity, as a function of the quencher concentration was measured. Fluorescence emission spectra and Stern-Volmer plots for each component are given in Figures S18-S22.

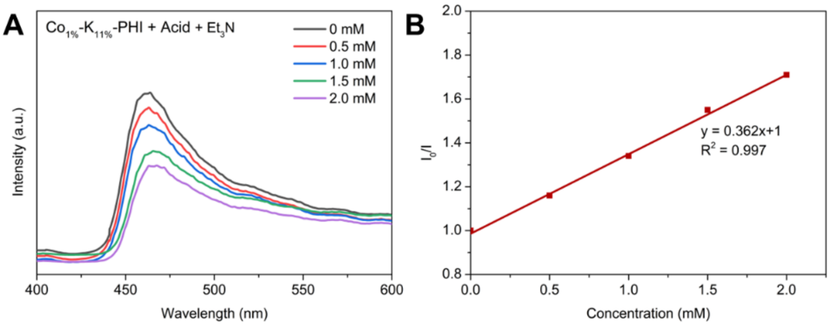

Figure S18. Emission spectra (A) and Stern-Volmer plot (B) of $Co_{1\%}$-$K_{11\%}$-PHI at different concentrations of the combination of *N*-Boc-*N*-methylglycine and Et_3N.

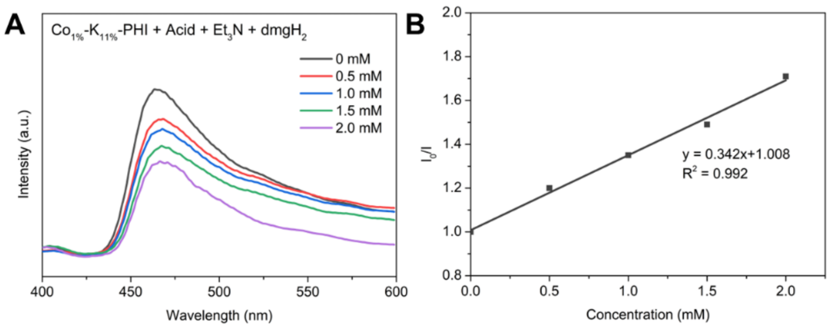

Figure S19. Emission spectra (A) and Stern-Volmer plot (B) of $Co_{1\%}$-$K_{11\%}$-PHI at different concentrations of the combination of *N*-Boc-*N*-methylglycine, Et_3N and $dmgH_2$.

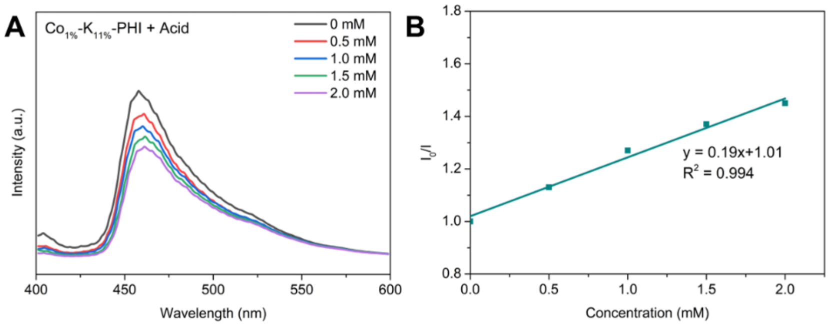

Figure S20. Emission spectra (A) and Stern-Volmer plot (B) of $Co_{1\%}$-$K_{11\%}$-PHI at different concentrations of *N*-Boc-*N*-methylglycine.

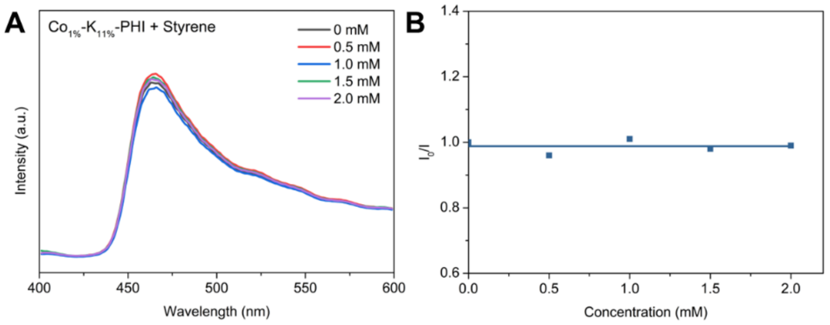

Figure S21. Emission spectra (A) and Stern-Volmer plot (B) of $Co_{1\%}$-$K_{11\%}$-PHI at different concentrations of styrene.

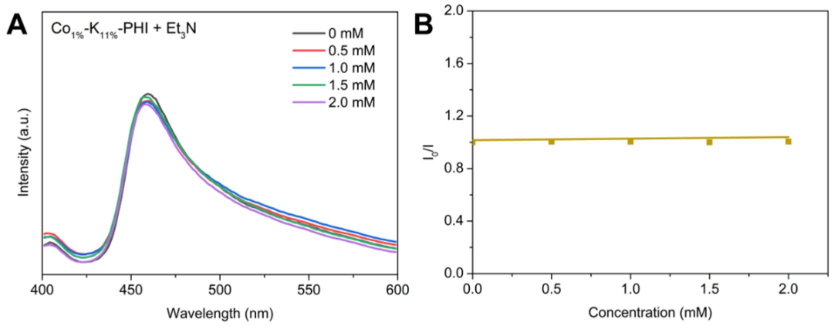

Figure S22. Emission spectra (A) and Stern-Volmer plot (B) of $Co_{1\%}$-$K_{11\%}$-PHI at different concentrations of Et_3N.

7.2 Radical Trapping Experiments

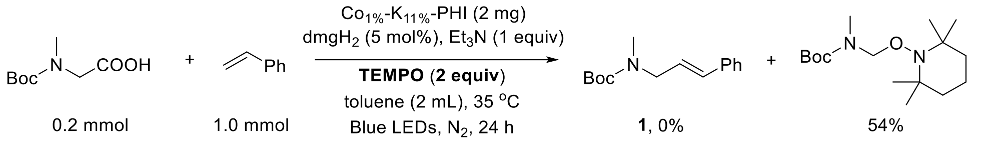

To a 10 mL oven-dried sealed tube equipped with a magnetic stir bar was added the corresponding acid (0.2 mmol, 1.0 equiv), $dmgH_2$ (5 mol%) and $Co_{1\%}$-$K_{11\%}$-PHI (2.0 mg, 0.17 mol% Co), Et_3N (0.2 mmol, 1.0 equiv), TEMPO (0.4 mmol, 2 equiv), and dry toluene (2.0 mL). The tube was closed with a rubber septum and the reaction mixture was degassed by three cycles vacuum/N_2 of "freeze-pump-thaw". Subsequently, the styrene (1 mmol, 5.0 equiv) was added via syringe. The reaction mixture was stirred and irradiated by blue LEDs (24 W, 460 ± 5 nm) without extra heating (35 ± 5 °C). After 48 h, no desired product **1** was observed by TLC, indicating that the reaction was completely inhibited. The reaction mixture was concentrated under reduced pressure to evaporate the solvent, and the crude residue was purified by silica gel column chromatography (petroleum ether/ethyl acetate 50/1) to afford the the radical trapping product (32.4 mg, 54% yield). The radical trapping product was characterized by ^{1}H-NMR, ^{13}C-NMR and HRMS.

***tert*-butyl methyl(((2,2,6,6-tetramethylpiperidin-1-yl)oxy)methyl)carbamate**

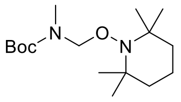

^{1}H NMR (400 MHz, $CDCl_3$) δ 4.93 – 4.77 (m, 2H), 3.06 – 2.91 (m, 3H), 1.59 – 1.41 (m, 15H), 1.18 (s, 6H), 1.10 (s, 6H). **^{13}C NMR** (101 MHz, $CDCl_3$) δ 155.5, 83.7, 80.2, 59.8, 39.8, 34.5, 33.1, 28.5, 20.1, 17.2. **HRMS (ESI)** m/z calcd. For $C_{16}H_{33}N_2O_3$ $[M+H]^+$ 301.2486, found 301.2485.

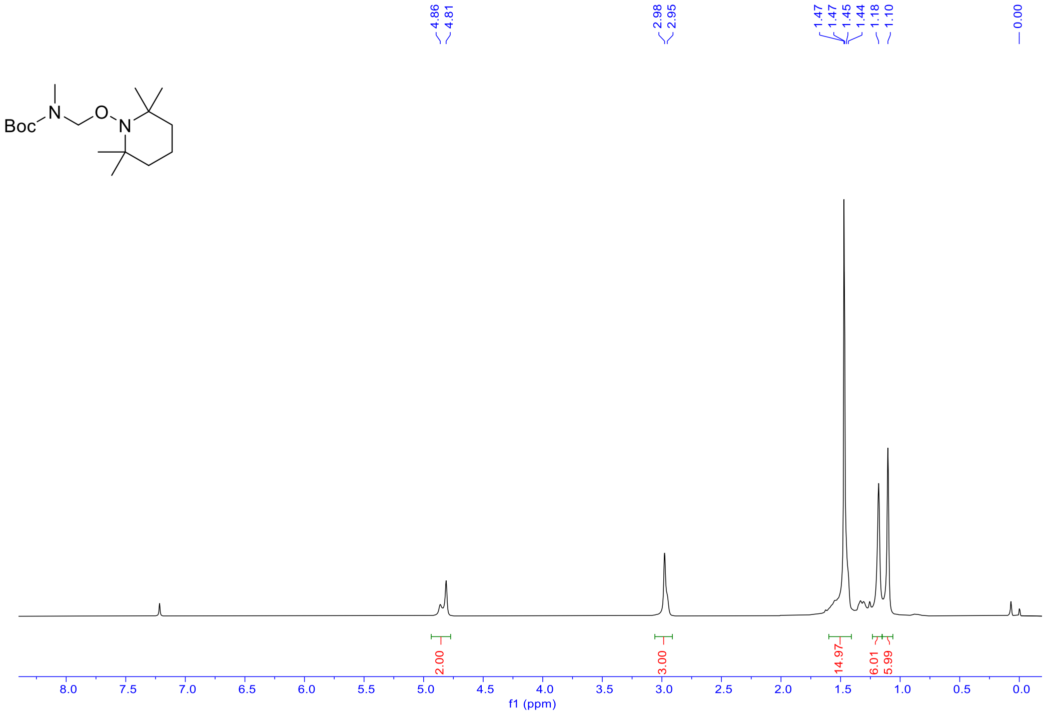

Figure S23. ^{1}H NMR spectra of **TEMPO adduct** (400 MHz, $CDCl_3$)

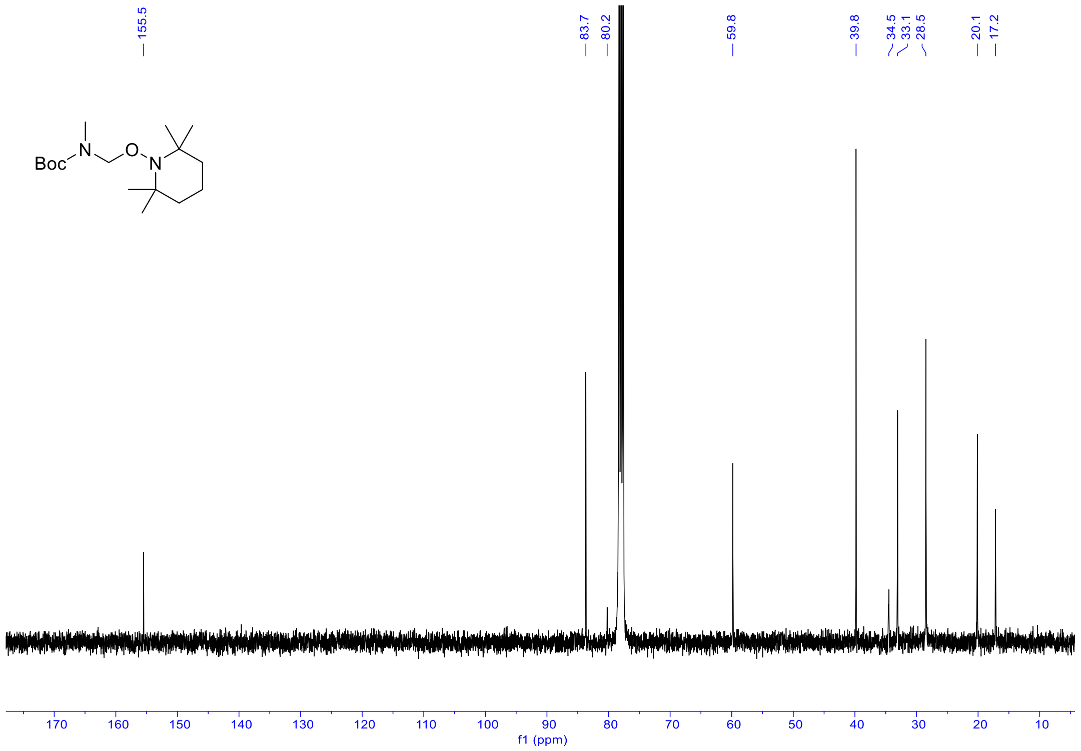

Figure S24. ^{13}C NMR spectra of **TEMPO adduct** (101 MHz, $CDCl_3$)

7.3 Hammett Studies

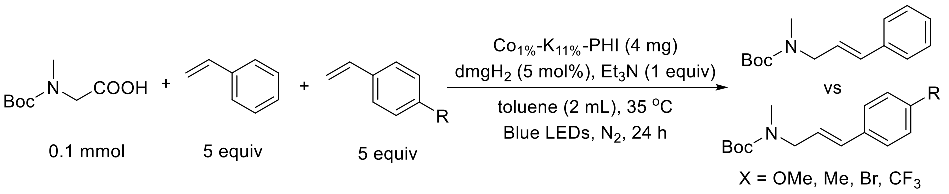

To a 10 mL oven-dried sealed tube equipped with a magnetic stir bar was added the corresponding acid (0.1 mmol, 1.0 equiv), $dmgH_2$ (5 mol%), $Co_{1\%}$-$K_{11\%}$-PHI (4.0 mg, 0.34 mol% Co), Et_3N (0.2 mmol, 1.0 equiv), and dry toluene (2.0 mL). The tube was closed with a rubber septum and the reaction mixture was degassed by three cycles vacuum/N_2 of “freeze-pump-thaw”. Subsequently, the styrene (0.5 mmol, 5.0 equiv) and the competing 4-substituted styrene (0.5 mmol, 5.0 equiv) were added via syringe. The reaction mixture was stirred and irradiated by blue LEDs (24 W, 460 ± 5 nm) without extra heating (35 ± 5 °C) for 24 h. Following completion of the reaction, the ratios between the two potential products (K_R/K_H) were determined by ^1H NMR analysis of the crude material, using trimethyl benzene-1,3,5-tricarboxylate as reference. Log(K_R/K_H) values were then plotted against the appropriate σ value for that substrate.

Table S15. Data analysis for para-substituted styrene derivatives

R	K_R/K_H	$log_{10}(K_R/K_H)$	σ_{para}
MeO	1/1.6	-0.2	-0.27
Me	1/1.29	-0.11	-0.17
H	1	0	0
Br	1.4/1	0.146	0.54
CF_3	1.9/1	0.28	0.23

7.4 Cyclic Voltammetry

Cyclic voltammetry (CV) measurements were taken on CHI660E using a platinum as working electrode (WE), $Ag/AgNO_3$ (0.01 M $AgNO_3$, 0.1 M NBu_4PF_6, MeCN) as reference electrode (RE), a platinum wire as counter electrode. Each compound was studied in a 50 mM concentration in a 0.1 M Bu_4NPF_6/MeCN solution (30 mL). Before voltammograms were recorded, the solution was purged with N_2, and a N_2 flow was kept in the headspace volume of the electrochemical cell during CV measurements. A potential scan rate of 50 mV/s was chosen, and the potential window ranging from +2.0 V to −2.5 V (and backwards) was investigated. Cyclic voltammetry was performed under roomtemperature conditions (25 – 27 °C). The potentials were given relative to the Fc/Fc^+ redox couple with ferrocene as internal standard. For conversion to SCE as reference, it is known that SCE is 400 mV more negative than Fc/Fc^+ in MeCN with NBu_4PF_6 as supporting electrolyte.[14]

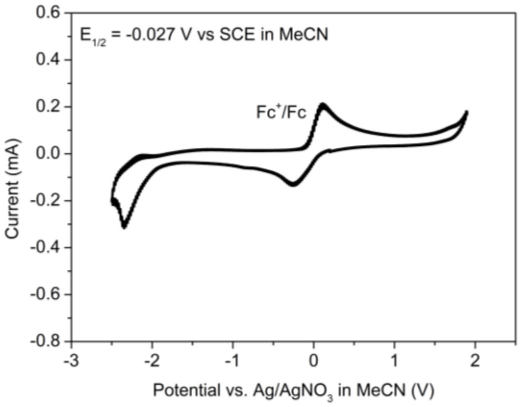

Figure S25. Cyclic voltammetry of Ferrocene in MeCN.

$E_{1/2}(Fc/Fc^+)$ = -0.027 V vs $Ag/AgNO_3$ in MeCN, and the potential values were then converted to the SCE reference system according to the equation $E_{SCE} = E_{Ag/AgNO3} + 0.43$ V.[15]

8 Characterization Data for the Products

***tert*-butyl cinnamyl(methyl)carbamate[16] (1)**

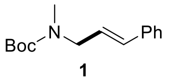

1

According to general procedure starting from *N*-Boc-*N*-methylglycine (0.2 mmol, 1.0 equiv), styrene (1.0 mmol, 5.0 equiv), Et_3N (0.2 mmol, 1.0 equiv), $dmgH_2$ (5 mol%) and $Co_{1\%}$-$K_{11\%}$-PHI (2 mg, 0.17 mol% Co) in toluene (2 mL) for 48 h, the product **1** was isolated as yellow oil after flash chromatography (petroleum ether/ethyl acetate 30/1), 37 mg (75% yield). **^{1}H NMR** (400 MHz, $CDCl_3$) δ 7.36 (d, *J* = 7.1 Hz, 2H), 7.31 (t, *J* = 7.5 Hz, 2H), 7.25 – 7.19 (m, 1H), 6.46 (d, *J* = 15.8 Hz, 1H), 6.14 (dt, *J* = 15.7, 6.2 Hz, 1H), 3.98 (s, 2H), 2.86 (s, 3H), 1.48 (s, 9H). **^{13}C NMR** (101 MHz, $CDCl_3$) δ 155.8, 136.8, 131.9, 128.6, 127.6, 126.4, 125.3, 79.6, 50.7, 33.8, 28.5.

Benzyl cinnamyl(methyl)carbamate[16] (2)

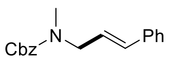

2

According to general procedure starting from *N*-Cbz-*N*-methylglycine (0.2 mmol, 1.0 equiv), styrene (1.0 mmol, 5.0 equiv), Et_3N (0.2 mmol, 1.0 equiv), $dmgH_2$ (5 mol%) and $Co_{1\%}$-$K_{11\%}$-PHI (2 mg, 0.17 mol% Co) in toluene (2 mL) for 48 h, the product **2** was isolated as yellow oil after flash chromatography (petroleum ether/ethyl acetate 20/1), 37 mg (66% yield). **^{1}H NMR** (400 MHz, $CDCl_3$, mixture of rotamers) δ 7.44 – 7.28 (m, 9H), 7.26 – 7.21 (m, 1H), 6.47 (t, *J* = 19.0 Hz, 1H), 6.15 (t, *J* = 17.1 Hz, 1H), 5.16 (s, 2H), 4.05 (s, 2H), 3.07 – 2.82 (m, 3H). **^{13}C NMR** (101 MHz, $CDCl_3$, mixture of rotamers) δ 156.3, 136.9, 136.6, 132.7, 132.1, 128.6, 128.5, 128.0, 127.9, 127.7, 126.4, 124.7, 67.2, 51.1, 50.8, 34.4, 33.5.

***tert*-butyl cinnamylcarbamate[16] (3)**

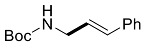

3

According to general procedure starting from *N*-Boc-*N*-glycine (0.2 mmol, 1.0 equiv), styrene (1.0 mmol, 5.0 equiv), Et_3N (0.2 mmol, 1.0 equiv), $dmgH_2$ (5 mol%) and $Co_{1\%}$-$K_{11\%}$-PHI (2 mg, 0.17 mol% Co) in toluene (2 mL) for 48 h, the product **3** was isolated as white solid after flash chromatography (petroleum ether/ethyl acetate 20/1), 30.5 mg (65% yield). **^{1}H NMR** (400 MHz, $CDCl_3$) δ 7.37 – 7.27 (m, 4H), 7.27 – 7.18 (m, 1H), 6.50 (d, *J* = 15.8 Hz, 1H), 6.26 – 6.10 (m, 1H), 4.70 (s, 1H), 3.90 (s, 2H), 1.47 (s, 9H). **^{13}C NMR** (101 MHz, $CDCl_3$) δ 155.8, 136.7, 131.5, 128.6, 127.6, 126.4, 79.5, 42.8, 28.4.

***tert*-butyl cinnamyl(ethyl)carbamate (4)**

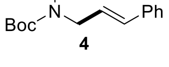

4

According to general procedure starting from *N*-Boc-*N*-ethylglycine (0.2 mmol, 1.0 equiv), styrene (1.0 mmol, 5.0 equiv), Et_3N (0.2 mmol, 1.0 equiv), $dmgH_2$ (5 mol%) and $Co_{1\%}$-$K_{11\%}$-PHI (2 mg, 0.17 mol% Co) in toluene (2 mL) for 48 h, the product **4** was isolated as yellow oil after flash chromatography (petroleum ether/ethyl acetate 20/1), 36.5 mg (70% yield). **^{1}H NMR** (400 MHz, $CDCl_3$) δ 7.36 (d, *J* = 7.0 Hz, 2H), 7.30 (t, *J* = 7.5 Hz, 2H), 7.25 – 7.19 (m, 1H), 6.45 (d, *J* = 15.9 Hz, 1H), 6.15 (dt, *J* = 16.1, 6.3 Hz, 1H), 3.97 (s, 2H), 3.26 (s, 2H), 1.48 (s, 9H), 1.11 (t, *J* = 7.1 Hz, 3H). **^{13}C NMR** (101 MHz, $CDCl_3$) δ 155.4, 136.9, 131.5, 128.6, 127.5, 126.4, 126.2, 79.4, 48.6, 41.3, 28.5, 13.6. **HRMS (ESI)** m/z calcd. For $C_{16}H_{23}NNaO_2$ $[M+Na]^+$ 284.1621, found 284.1626.

***tert*-butyl cinnamyl(cyclopentyl)carbamate (5)**

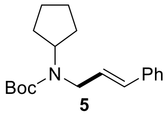

According to general procedure starting from *N*-Boc-*N*-cyclopentylglycine (0.2 mmol, 1.0 equiv), styrene (1.0 mmol, 5.0 equiv), Et_3N (0.2 mmol, 1.0 equiv), $dmgH_2$ (5 mol%) and $Co_{1\%}$-$K_{11\%}$-PHI (2 mg, 0.17 mol% Co) in toluene (2 mL) for 48 h, the product **5** was isolated as yellow oil after flash chromatography (petroleum ether/ethyl acetate 20/1), 36 mg (60% yield). **^{1}H NMR** (400 MHz, $CDCl_3$) δ 7.37 – 7.28 (m, 4H), 7.25 – 7.18 (m, 1H), 6.43 (d, *J* = 15.9 Hz, 1H), 6.17 (dt, *J* = 15.9, 5.6 Hz, 1H), 4.34 (s, 1H), 3.89 (d, *J* = 5.6 Hz, 2H), 1.84 (dt, *J* = 10.9, 3.4 Hz, 2H), 1.77 – 1.62 (m, 2H), 1.54 (dt, *J* = 10.3, 6.1 Hz, 4H), 1.46 (s, 9H). **^{13}C NMR** (101 MHz, $CDCl_3$) δ 155.7, 137.1, 130.5, 128.6, 127.8, 127.3, 126.2, 79.4, 57.4, 45.6, 29.5, 28.6, 23.8. **HRMS (ESI)** m/z calcd. For $C_{19}H_{27}NNaO_2$ $[M+Na]^+$ 324.1934, found 324.1491.

***tert*-butyl cinnamyl(2-(methylthio)ethyl)carbamate (6)**

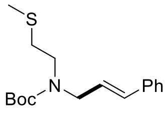

According to general procedure starting from *N*-Boc-*N*-(2-(methylthio)ethyl)glycine (0.2 mmol, 1.0 equiv), styrene (1.0 mmol, 5.0 equiv), Et_3N (0.2 mmol, 1.0 equiv), $dmgH_2$ (5 mol%) and $Co_{1\%}$-$K_{11\%}$-PHI (2 mg, 0.17 mol% Co) in toluene (2 mL) for 48 h, the product **6** was isolated as yellow oil after flash chromatography (petroleum ether/ethyl acetate 20/1), 27.6 mg (45% yield). **^{1}H NMR** (400 MHz, $CDCl_3$, mixture of rotamers) δ 7.39 – 7.28 (m, 4H), 7.26 – 7.21 (m, 1H), 6.47 (d, *J* = 16.0 Hz, 1H), 6.25 – 6.08 (m, 1H), 4.12 – 3.39 (m, 2H), 3.51 – 3.33 (m, 2H), 2.72 – 2.60 (m, 2H), 2.16 – 2.07 (m, 3H), 1.48 (s, 9H). **^{13}C NMR** (101 MHz, $CDCl_3$, mixture of rotamers) δ 155.3, 136.7, 132.4, 131.6, 128.6, 127.7, 126.4, 125.7, 79.9, 50.0, 49.4, 46.3, 32.3, 28.5, 15.5. **HRMS (ESI)** m/z calcd. For $C_{17}H_{26}NO_2S$ $[M+H]^+$ 308.16788, found 308.1685.

***tert*-butyl (*R*,*E*)-2-styrylpyrrolidine-1-carboxylate[17] (7)**

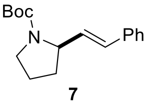

According to general procedure starting from *N*-Boc-D-proline (0.2 mmol, 1.0 equiv), styrene (1.0 mmol, 5.0 equiv), Et_3N (0.2 mmol, 1.0 equiv), $dmgH_2$ (5 mol%) and $Co_{1\%}$-$K_{11\%}$-PHI (2 mg, 0.17 mol% Co) in toluene (2 mL) for 48 h, the product **7** was isolated as white solid after flash chromatography (petroleum ether/ethyl acetate 20/1), 28 mg (51% yield). **^{1}H NMR** (400 MHz, $CDCl_3$) δ 7.35 (d, *J* = 7.7 Hz, 2H), 7.29 (t, *J* = 7.4 Hz, 2H), 7.21 (t, *J* = 7.1 Hz, 1H), 6.39 (d, *J* = 15.7 Hz, 1H), 6.19 – 6.00 (m, 1H), 4.62 – 4.31 (m, 1H), 3.58 – 3.33 (m, 2H), 2.22 – 2.04 (m, 1H), 2.01 – 1.79 (m, 3H), 1.54 – 1.39 (m, 9H). **^{13}C NMR** (101 MHz, $CDCl_3$) δ 154.7, 137.1, 130.8, 129.4, 128.5, 127.3, 126.3, 79.2, 59.0, 46.3, 32.6, 28.5, 23.1.

***tert*-butyl (*S*,*E*)-3-styrylmorpholine-4-carboxylate[18] (8)**

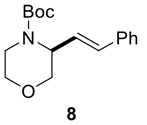

According to general procedure starting from *N*-Boc-morpholine-3-carboxylic acid (0.2 mmol, 1.0 equiv), styrene (1.0 mmol, 5.0 equiv), Et_3N (0.2 mmol, 1.0 equiv), $dmgH_2$ (5 mol%) and $Co_{1\%}$-$K_{11\%}$-PHI (2 mg, 0.17 mol% Co) in toluene (2 mL) for 48 h, the product **8** was isolated as yellow oil after flash chromatography (petroleum ether/ethyl acetate 20/1), 25 mg (43% yield). **^{1}H NMR** (400 MHz, $CDCl_3$) δ 7.39 (d, *J* = 7.0 Hz, 2H), 7.32 (t, *J* = 7.6 Hz, 2H), 7.24 – 7.20 (m, 1H), 6.57 (d, *J* = 15.8 Hz, 1H), 6.40 (dd, *J* = 16.0, 6.4 Hz, 1H), 4.59 (br, 1H), 3.97 (d, *J* = 11.6 Hz, 1H), 3.90 (dd, *J* = 11.4, 3.7 Hz, 1H), 7.78 – 7.70 (m, 2H), 3.53 (td,

J = 11.8, 2.9 Hz, 1H), 3.24 (td, J = 12.9, 3.6 Hz, 1H), 1.48 (s, 9H). **^{13}C NMR** (101 MHz, $CDCl_3$) δ 155.0, 136.7, 132.4, 128.6, 127.7, 126.5, 125.9, 80.2, 70.0, 67.0, 53.1, 28.4.

(cinnamyloxy)benzene[19] **(9)**

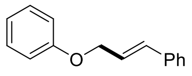

9

According to general procedure starting from 2-phenoxyacetic acid (0.2 mmol, 1.0 equiv), styrene (0.4 mmol, 2.0 equiv), Et_3N (0.2 mmol, 1.0 equiv), $dmgH_2$ (5 mol%) and $Co_{1\%}$-$K_{11\%}$-PHI (2 mg, 0.17 mol% Co) in toluene (2 mL) for 48 h, the product **9** was isolated as white solid after flash chromatography (petroleum ether/ethyl acetate 100/1), 33 mg (79% yield). **^{1}H NMR** (400 MHz, $CDCl_3$) δ 7.44 – 7.37 (m, 2H), 7.36 – 7.26 (m, 4H), 7.29 – 7.20 (m, 1H), 7.01 – 6.91 (m, 3H), 6.73 (d, J = 16.0 Hz, 1H), 6.42 (dt, J = 16.0, 5.8 Hz, 1H), 4.69 (dd, J = 5.9, 1.5 Hz, 2H). **^{13}C NMR** (101 MHz, $CDCl_3$) δ 157.6, 135.4, 131.9, 128.5, 127.5, 126.8, 125.5, 123.5, 119.9, 113.7, 67.5.

1-(cinnamyloxy)-4-methylbenzene[19] **(10)**

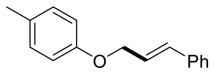

10

According to general procedure starting from 2-(*p*-tolyloxy)acetic acid (0.2 mmol, 1.0 equiv), styrene (0.4 mmol, 2.0 equiv), Et_3N (0.2 mmol, 1.0 equiv), $dmgH_2$ (5 mol%) and $Co_{1\%}$-$K_{11\%}$-PHI (2 mg, 0.17 mol% Co) in toluene (2 mL) for 48 h, the product **10** was isolated as yellow oil after flash chromatography (petroleum ether/ethyl acetate 100/1), 34 mg (76% yield). **^{1}H NMR** (400 MHz, $CDCl_3$) δ 7.39 (d, J = 7.9 Hz, 2H), 7.35 – 7.26 (m, 2H), 7.27 – 7.19 (m, 1H), 7.08 (d, J = 8.1 Hz, 2H), 6.90 – 6.84 (m, 2H), 6.71 (d, J = 15.8 Hz, 1H), 6.40 (dt, J = 15.9, 5.7 Hz, 1H), 4.65 (dd, J = 5.8, 1.5 Hz, 2H), 2.28 (s, 3H). **^{13}C NMR** (101 MHz, $CDCl_3$) δ 156.6, 136.6, 132.9, 130.2, 130.0, 128.6, 127.9, 126.6, 124.8, 114.7, 68.8, 20.6.

1-(cinnamyloxy)-3-methylbenzene[20] **(11)**

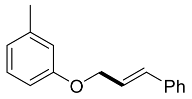

11

According to general procedure starting from 2-(*m*-tolyloxy)acetic acid (0.2 mmol, 1.0 equiv), styrene (0.4 mmol, 2.0 equiv), Et_3N (0.2 mmol, 1.0 equiv), $dmgH_2$ (5 mol%) and $Co_{1\%}$-$K_{11\%}$-PHI (2 mg, 0.17 mol% Co) in toluene (2 mL) for 48 h, the product **11** was isolated as yellow oil after flash chromatography (petroleum ether/ethyl acetate 40/1), 31.5 mg (70% yield). **^{1}H NMR** (400 MHz, $CDCl_3$) δ 7.41 (d, J = 7.4 Hz, 2H), 7.32 (d, J = 7.5 Hz, 2H), 7.26 – 7.20 (m, 1H), 7.14 (t, J = 7.6 Hz, 2H), 6.91 – 6.83 (m, 2H), 6.73 (d, J = 15.9 Hz, 1H), 6.43 (dt, J = 16.0, 5.6 Hz, 1H), 4.70 (dd, J = 5.7, 1.6 Hz, 2H), 2.28 (s, 3H). **^{13}C NMR** (101 MHz, $CDCl_3$) δ 156.9, 136.7, 132.3, 130.8, 128.6, 127.9, 127.1, 126.8, 126.6, 125.1, 120.6, 111.5, 68.7, 16.4.

1-(cinnamyloxy)-2-methylbenzene[20] **(12)**

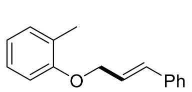

12

According to general procedure starting from 2-(*o*-tolyloxy)acetic acid (0.2 mmol, 1.0 equiv), styrene (0.4 mmol, 2.0 equiv), Et_3N (0.2 mmol, 1.0 equiv), $dmgH_2$ (5 mol%) and $Co_{1\%}$-$K_{11\%}$-PHI (2 mg, 0.17 mol% Co) in toluene (2 mL) for 48 h, the product **12** was isolated as yellow oil after flash chromatography (petroleum ether/ethyl acetate 100/1), 30.5 mg (68% yield). **^{1}H NMR** (400 MHz, $CDCl_3$) δ 7.43 (d, J = 1.6 Hz, 2H), 7.33 (t, J = 7.5 Hz, 2H), 7.29 – 7.21 (m, 1H), 7.15 (t, J = 7.0 Hz, 2H), 6.94 – 6.84 (m, 2H), 6.74 (d, J = 16.0 Hz,

1H), 6.43 (dt, J = 16.0, 5.6 Hz, 1H), 4.70 (dd, J = 5.6, 1.6 Hz, 2H), 2.28 (s, 3H). **^{13}C NMR** (101 MHz, CDCl$_3$) δ 156.9, 136.6, 132.3, 130.8, 128.6, 127.8, 127.1, 126.8, 126.6, 125.1, 120.6, 111.5, 68.7, 16.4.

1-(cinnamyloxy)-4-methoxybenzene[20] (13)

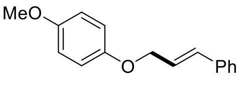

13

According to general procedure starting from 2-(4-methoxyphenoxy)acetic acid (0.2 mmol, 1.0 equiv), styrene (0.4 mmol, 2.0 equiv), Et$_3$N (0.2 mmol, 1.0 equiv), dmgH$_2$ (5 mol%) and Co$_{1\%}$-K$_{11\%}$-PHI (2 mg, 0.17 mol% Co) in toluene (2 mL) for 48 h, the product **13** was isolated as white solid after flash chromatography (petroleum ether/ethyl acetate 100/1), 28.8 mg (60% yield). **^{1}H NMR** (400 MHz, CDCl$_3$) δ 7.44 – 7.38 (m, 2H), 7.35 – 7.29 (m, 2H), 7.28 – 7.21 (m, 1H), 6.93 – 6.88 (m, 2H), 6.87 – 6.81 (m, 2H), 6.74 (d, J = 1.8 Hz, 1H), 6.41 (dt, J = 16.0, 5.8 Hz, 1H), 4.65 (dd, J = 5.8, 1.5 Hz, 2H), 3.77 (s, 3H). **^{13}C NMR** (101 MHz, CDCl$_3$) δ 151.5, 150.3, 134.0, 130.3, 126.1, 125.3, 124.0, 122.3, 113.3, 112.1, 66.9, 53.2.

1-(cinnamyloxy)-4-fluorobenzene[20] (14)

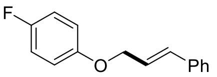

14

According to general procedure starting from 2-(4-fluorophenoxy)acetic acid (0.2 mmol, 1.0 equiv), styrene (0.4 mmol, 2.0 equiv), Et$_3$N (0.2 mmol, 1.0 equiv), dmgH$_2$ (5 mol%) and Co$_{1\%}$-K$_{11\%}$-PHI (2 mg, 0.17 mol% Co) in toluene (2 mL) for 48 h, the product **14** was isolated as white solid after flash chromatography (petroleum ether/ethyl acetate 100/1), 33.3 mg (73% yield). **^{1}H NMR** (400 MHz, CDCl$_3$) δ 7.40 (d, J = 7.1 Hz, 2H), 7.32 (t, J = 7.5 Hz, 2H), 7.24 (d, J = 6.2 Hz, 1H), 7.01 – 6.94 (m, 2H), 6.92 – 6.85 (m, 2H), 6.71 (d, J = 16.1 Hz, 1H), 6.39 (dt, J = 16.0, 5.8 Hz, 1H), 4.65 (dd, J = 5.8, 1.6 Hz, 2H). **^{13}C NMR** (101 MHz, CDCl$_3$) δ 157.4 (d, J = 238.4 Hz), 154.8 (d, J = 2.1 Hz), 136.4, 133.1, 128.6, 128.0, 126.6, 124.3, 115.9 (d, J = 15.6 Hz), 115.8 (d, J = 15.4 Hz), 69.3. **^{19}F NMR** (377 MHz, CDCl$_3$) δ -123.8 (s).

1-chloro-4-(cinnamyloxy)benzene[19] (15)

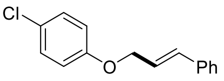

15

According to general procedure starting from 2-(4-chlorophenoxy)acetic acid (0.2 mmol, 1.0 equiv), styrene (0.4 mmol, 2.0 equiv), Et$_3$N (0.2 mmol, 1.0 equiv), dmgH$_2$ (5 mol%) and Co$_{1\%}$-K$_{11\%}$-PHI (2 mg, 0.17 mol% Co) in toluene (2 mL) for 48 h, the product **15** was isolated as white solid after flash chromatography (petroleum ether/ethyl acetate 100/1), 34.5 mg (70% yield). **^{1}H NMR** (400 MHz, CDCl$_3$) δ 7.43 – 7.38 (m, 2H), 7.34 – 7.29 (m, 2H), 7.26 – 7.21 (m, 3H), 6.94 – 6.84 (m, 2H), 6.71 (dt, J = 16.0, 1.7 Hz, 1H), 6.38 (dt, J = 15.9, 5.8 Hz, 1H), 4.66 (dd, J = 5.8, 1.5 Hz, 2H). **^{13}C NMR** (101 MHz, CDCl$_3$) δ 157.3, 136.3, 133.3, 129.4, 128.7, 128.0, 126.6, 125.8, 124.0, 116.1, 69.0.

1-bromo-4-(cinnamyloxy)benzene[21] (16)

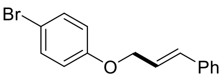

16

According to general procedure starting from 2-(4-bromophenoxy)acetic acid (0.2 mmol, 1.0 equiv), styrene (0.4 mmol, 2.0 equiv), Et$_3$N (0.2 mmol, 1.0 equiv), dmgH$_2$ (5 mol%) and Co$_{1\%}$-K$_{11\%}$-PHI (2 mg, 0.17 mol% Co) in toluene (2 mL) for 48 h, the product **16** was isolated as yellow oil after flash chromatography (petroleum

ether/ethyl acetate 80/1), 45 mg (78% yield). **^{1}H NMR** (400 MHz, $CDCl_3$) δ 7.38 (t, *J* = 8.5 Hz, 4H), 7.32 (t, *J* = 7.4 Hz, 2H), 7.26 – 7.22 (m, 1H), 6.83 (d, *J* = 8.9 Hz, 2H), 6.71 (d, *J* = 16.0 Hz, 1H), 6.37 (dt, *J* = 16.0, 5.8 Hz, 1H), 4.65 (dd, *J* = 5.9, 1.6 Hz, 2H). **^{13}C NMR** (101 MHz, $CDCl_3$) δ 157.8, 136.3, 133.3, 132.3, 128.7, 128.1, 126.6, 124.0, 116.7, 113.1, 68.9.

2-(cinnamyloxy)naphthalene[21] (17)

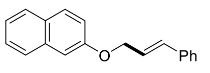

17

According to general procedure starting from 2-(naphthalen-2-yloxy)acetic acid (0.2 mmol, 1.0 equiv), styrene (1.0 mmol, 5.0 equiv), Et_3N (0.2 mmol, 1.0 equiv), $dmgH_2$ (5 mol%) and $Co_{1\%}$-$K_{11\%}$-PHI (2 mg, 0.17 mol% Co) in toluene (2 mL) for 48 h, the product **17** was isolated as white solid after flash chromatography (petroleum ether/ethyl acetate 100/1), 32.8 mg (63% yield). **^{1}H NMR** (400 MHz, $CDCl_3$) δ 7.80 – 7.70 (m, 3H), 7.46 – 7.40 (m, 3H), 7.36 – 7.31 (m, 3H), 7.29 – 7.25 (m, 1H), 7.79 – 7.72 (m, 2H), 6.79 (d, *J* = 16.0 Hz, 1H), 6.48 (dt, *J* = 15.9, 5.8 Hz, 1H), 4.81 (dd, *J* = 5.8, 1.5 Hz, 2H). **^{13}C NMR** (101 MHz, $CDCl_3$) δ 156.6, 136.5, 134.5, 133.2, 129.5, 129.1, 128.6, 128.0, 127.7, 126.8, 126.6, 126.4, 124.3, 123.7, 119.0, 107.1, 68.7.

(*E*)-(3-methoxyprop-1-en-1-yl)benzene[22] (18)

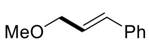

18

According to general procedure starting from 2-methoxyacetic acid (0.2 mmol, 1.0 equiv), styrene (1.0 mmol, 5.0 equiv), Et_3N (0.2 mmol, 1.0 equiv), $dmgH_2$ (5 mol%) and $Co_{1\%}$-$K_{11\%}$-PHI (2 mg, 0.17 mol% Co) in toluene (2 mL) for 48 h, the product **18** was isolated as yellow oil after flash chromatography (petroleum ether/ethyl acetate 80/1), 17 mg (57% yield). **^{1}H NMR** (400 MHz, $CDCl_3$) δ 7.43 – 7.36 (m, 2H), 7.35 – 7.28 (m, 2H), 7.26 – 7.20 (m, 1H), 6.60 (d, *J* = 16.0 Hz, 1H), 6.28 (dt, *J* = 15.9, 6.0 Hz, 1H), 4.08 (dd, *J* = 6.0, 1.5 Hz, 2H), 3.38 (s, 3H). **^{13}C NMR** (101 MHz, $CDCl_3$) δ 136.8, 132.5, 128.6, 127.7, 126.5, 126.0, 73.1, 58.0.

(*E*)-(3-ethoxyprop-1-en-1-yl)benzene[22] (19)

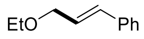

19

According to general procedure starting from 2-ethoxyacetic acid (0.2 mmol, 1.0 equiv), styrene (1.0 mmol, 5.0 equiv), Et_3N (0.2 mmol, 1.0 equiv), $dmgH_2$ (5 mol%) and $Co_{1\%}$-$K_{11\%}$-PHI (2 mg, 0.17 mol% Co) in toluene (2 mL) for 48 h, the product **19** was isolated as yellow oil after flash chromatography (petroleum ether/ethyl acetate 80/1), 17.5 mg (54% yield). **^{1}H NMR** (400 MHz, $CDCl_3$) δ 7.42 – 7.35 (m, 2H), 7.34 – 7.25 (m, 2H), 7.26 – 7.17 (m, 1H), 6.60 (dt, *J* = 15.8, 1.5 Hz, 1H), 6.30 (dt, *J* = 15.9, 6.0 Hz, 1H), 4.13 (dd, *J* = 6.0, 1.5 Hz, 2H), 3.54 (q, *J* = 7.0 Hz, 2H), 1.24 (t, *J* = 7.0 Hz, 3H). **^{13}C NMR** (101 MHz, $CDCl_3$) δ 136.8, 132.2, 128.6, 127.6, 126.5, 126.4, 71.3, 65.7, 15.3.

(*R*,*E*)-2-styryltetrahydrofuran[23] **(20)**

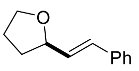

20

According to general procedure starting from 2-Furoic acid (0.2 mmol, 1.0 equiv), styrene (1.0 mmol, 5.0 equiv), Et_3N (0.2 mmol, 1.0 equiv), $dmgH_2$ (5 mol%) and $Co_{1\%}$-$K_{11\%}$-PHI (2 mg, 0.17 mol% Co) in toluene (2 mL) for 48 h, the product **20** was isolated as yellow oil after flash chromatography (petroleum ether/ethyl acetate 80/1), 12.5 mg (36% yield). **^{1}H NMR** (400 MHz, $CDCl_3$) δ 7.41 – 7.35 (m, 2H), 7.32 – 7.27 (m, 2H), 7.24 – 7.20 (m, 1H), 6.58 (d, *J* = 15.9 Hz, 1H), 6.20 (dd, *J* = 15.8, 6.6 Hz, 1H), 4.47 (q, *J* = 6.3 Hz, 1H), 4.06 – 3.91 (m, 1H), 3.87 – 3.81 (m, 1H), 2.22 – 2.05 (m, 1H), 2.02 – 1.83 (m, 2H), 1.75 – 1.62 (m, 1H). **^{13}C NMR** (101 MHz, $CDCl_3$) δ 136.9, 130.6, 130.5, 128.5, 127.5, 126.5, 79.7, 68.2, 32.4, 25.9.

1-cinnamyl-4-methoxybenzene[24] **(21)**

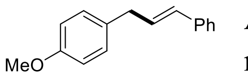

21

According to general procedure starting from 2-(4-methoxyphenyl)acetic acid (0.2 mmol, 1.0 equiv), styrene (1.0 mmol, 5.0 equiv), Et_3N (0.2 mmol, 1.0 equiv), $dmgH_2$ (5 mol%) and $Co_{1\%}$-$K_{11\%}$-PHI (2 mg, 0.17 mol% Co) in toluene (2 mL) for 48 h, the product **21** was isolated as colorless oil after flash chromatography (petroleum ether/ethyl acetate 50/1), 19.3 mg (43% yield). **^{1}H NMR** (400 MHz, $CDCl_3$) δ 7.38 – 7.32 (m, 2H), 7.30 – 7.26 (m, 2H), 7.20 – 7.14 (m, 3H), 6.89 – 6.82 (m, 2H), 6.43 (d, *J* = 15.8 Hz, 1H), 6.33 (dt, *J* = 15.7, 6.5 Hz, 1H), 3.79 (s, 3H), 3.49 (d, *J* = 6.5 Hz, 2H). **^{13}C NMR** (101 MHz, $CDCl_3$) δ 158.1, 137.6, 132.2, 130.8, 129.7, 129.6, 128.5, 127.1, 126.1, 113.9, 55.3, 38.5.

4-cinnamyl-1,1'-biphenyl[25] **(22)**

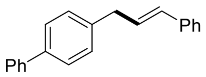

22

According to general procedure starting from 2-([1,1'-biphenyl]-4-yl)acetic acid (0.2 mmol, 1.0 equiv), styrene (1.0 mmol, 5.0 equiv), Et_3N (0.2 mmol, 1.0 equiv), $dmgH_2$ (5 mol%) and $Co_{1\%}$-$K_{11\%}$-PHI (2 mg, 0.17 mol% Co) in toluene (2 mL) for 48 h, the product **22** was isolated as colorless oil after flash chromatography (petroleum ether), 21.5 mg (40% yield). **^{1}H NMR** (400 MHz, $CDCl_3$) δ 7.60 – 7.50 (m, 4H), 7.44 – 7.33 (m, 4H), 7.33 – 7.27 (m, 4H), 7.23 – 7.16 (m, 2H), 6.48 (d, *J* = 15.8 Hz, 1H), 6.37 (dt, *J* = 15.8, 6.7 Hz, 1H), 3.57 (d, *J* = 6.8 Hz, 2H). **^{13}C NMR** (101 MHz, $CDCl_3$) δ 141.1, 139.3, 139.3, 137.5, 131.3, 129.2, 129.1, 128.8, 128.6, 127.3, 127.2, 127.2, 127.1, 126.2, 39.1.

***tert*-butyl (4-cinnamylphenyl)carbamate (23)**

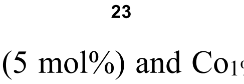

23

According to general procedure starting from 2-(4-((*tert*-butoxycarbonyl)amino)phenyl)acetic acid (0.2 mmol, 1.0 equiv), styrene (1.0 mmol, 5.0 equiv), Et_3N (0.2 mmol, 1.0 equiv). $dmgH_2$ (5 mol%) and $Co_{1\%}$-$K_{11\%}$-PHI (2 mg, 0.17 mol% Co) in toluene (2 mL) for 48 h, the product **23** was isolated as white solid after flash chromatography (petroleum ether/ethyl acetate 20/1), 21 mg (34% yield). mp = 58–60 ºC. **^{1}H NMR** (400 MHz, $CDCl_3$) δ 7.36 – 7.33 (m, 2H), 7.31 – 7.26 (m, 4H), 7.22 – 7.18 (m, 1H), 7.17 – 7.14 (m, 2H), 6.42 (d, *J* = 17.5 Hz, 2H), 6.32 (dt, *J* = 15.8, 6.6 Hz, 1H), 3.49 (d, *J* = 6.5 Hz, 2H), 1.51 (s, 9H). **^{13}C NMR** (101 MHz, $CDCl_3$) δ 152.9, 137.5, 136.5, 134.8, 130.9, 129.4, 129.2, 128.5, 127.1, 126.1, 118.8, 80.4, 38.6, 28.4. **HRMS (ESI)** m/z calcd. For

$C_{20}H_{23}NNaO_2$ $[M+Na]^+$ 332.1621, found 332.1628.

2-cinnamyldibenzo[*b*,*e*]oxepin-11(6*H*)-one[24] (24)

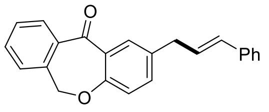

24

According to general procedure starting from isoxepac (0.2 mmol, 1.0 equiv), styrene (1.0 mmol, 5.0 equiv), Et_3N (0.2 mmol, 1.0 equiv), $dmgH_2$ (5 mol%) and $Co_{1\%}$-$K_{11\%}$-PHI (2 mg, 0.17 mol% Co) in toluene (2 mL) for 48 h, the product **24** was isolated as yellow oil after flash chromatography (petroleum ether/ethyl acetate 40/1), 26.1 mg (40% yield). **^{1}H NMR** (400 MHz, $CDCl_3$) δ 8.10 (d, *J* = 2.5 Hz, 1H), 7.89 (dd, *J* = 7.7, 1.6 Hz, 1H), 7.54 (td, *J* = 7.4, 1.6 Hz, 1H), 7.45 (td, *J* = 7.6, 1.4 Hz, 1H), 7.38 – 7.33 (m, 4H), 7.28 (t, *J* = 7.6 Hz, 2H), 7.24 – 7.15 (m, 1H), 7.00 (d, *J* = 8.4 Hz, 1H), 6.46 (d, *J* = 15.8 Hz, 1H), 6.33 (dt, *J* = 15.8, 6.8 Hz, 1H), 5.16 (s, 2H), 3.55 (d, *J* = 6.8 Hz, 2H). **^{13}C NMR** (101 MHz, $CDCl_3$) δ 191.2, 159.9, 140.6, 137.4, 136.0, 135.7, 133.9, 132.7, 131.5, 131.4, 129.5, 129.2, 128.8, 128.5, 127.8, 127.2, 126.2, 125.2, 120.9, 73.7, 38.4.

(*E*)-chalcone[26] (25)

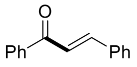

25

According to general procedure starting from benzoylformic acid (0.2 mmol, 1.0 equiv), styrene (1.0 mmol, 5.0 equiv), Et_3N (0.2 mmol, 1.0 equiv), $dmgH_2$ (5 mol%) and $Co_{1\%}$-$K_{11\%}$-PHI (2 mg, 0.17 mol% Co) in toluene (2 mL) for 48 h, the product **25** was isolated as white solid after flash chromatography (petroleum ether/ethyl acetate 30/1), 20.8 mg (50% yield). **^{1}H NMR** (400 MHz, $CDCl_3$) δ 8.05 – 7.99 (m, 2H), 7.81 (d, *J* = 15.7 Hz, 1H), 7.67 – 7.61 (m, 2H), 7.62 – 7.46 (m, 4H), 7.45 – 7.38 (m, 3H). **^{13}C NMR** (101 MHz, $CDCl_3$) δ 190.6, 144.9, 138.2, 134.9, 132.8, 130.6, 129.0, 128.7, 128.5, 128.5, 122.1.

***tert*-butyl (*E*)-methyl(3-(*p*-tolyl)allyl)carbamate[27] (26)**

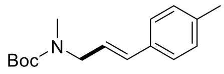

26

According to general procedure starting from *N*-Boc-*N*-methylglycine (0.2 mmol, 1.0 equiv), 1-methyl-4-vinylbenzene (1.0 mmol, 5.0 equiv), Et_3N (0.2 mmol, 1.0 equiv), $dmgH_2$ (5 mol%) and $Co_{1\%}$-$K_{11\%}$-PHI (2 mg, 0.17 mol% Co) in toluene (2 mL) for 48 h, the product **26** was isolated as yellow oil after flash chromatography (petroleum ether/ethyl acetate 20/1), 28.7 mg (55% yield). **^{1}H NMR** (400 MHz, $CDCl_3$) δ 7.26 (d, *J* = 7.5 Hz, 2H), 7.12 (d, *J* = 7.8 Hz, 2H), 6.43 (d, *J* = 15.8 Hz, 1H), 6.16 – 5.98 (m, 1H), 3.96 (s, 2H), 2.85 (s, 3H), 2.33 (s, 3H), 1.47 (s, 9H). **^{13}C NMR** (101 MHz, $CDCl_3$) δ 155.8, 137.4, 134.0, 131.7, 129.3, 126.3, 124.2, 79.5, 50.7, 33.7, 28.5, 21.2.

***tert*-butyl (*E*)-(3-(4-methoxyphenyl)allyl)(methyl)carbamate[27] (27)**

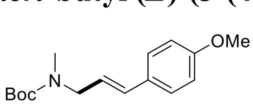

27

According to general procedure starting from *N*-Boc-*N*-methylglycine (0.2 mmol, 1.0 equiv), 1-methoxy-4-vinylbenzene (1.0 mmol, 5.0 equiv), Et_3N (0.2 mmol, 1.0 equiv), $dmgH_2$ (5 mol%) and $Co_{1\%}$-$K_{11\%}$-PHI (2 mg, 0.17 mol% Co) in toluene (2 mL) for 48 h, the product **27** was isolated as white solid after flash chromatography (petroleum ether/ethyl acetate 20/1), 19 mg (34% yield). **^{1}H NMR** (400 MHz, $CDCl_3$) δ 7.26 – 7.20 (m, 2H), 6.78 (d, *J* = 8.7 Hz, 2H), 6.33 (d, *J* = 15.8 Hz, 1H), 5.94 (dd, *J* = 14.6, 7.6 Hz, 1H), 3.88 (s, 2H), 3.73 (s, 3H), 2.78 (s, 3H), 1.40 (s, 9H). **^{13}C NMR** (101 MHz, $CDCl_3$) δ 158.2, 154.7, 130.2, 128.5, 126.5, 122.0, 113.0, 78.5, 54.3, 50.1, 32.7,

27.5.

***tert*-butyl (*E*)-(3-(4-fluorophenyl)allyl)(methyl)carbamate (28)**

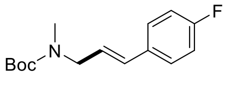

28

According to general procedure starting from *N*-Boc-*N*-methylglycine (0.2 mmol, 1.0 equiv), 1-fluoro-4-vinylbenzene (1.0 mmol, 5.0 equiv), Et_3N (0.2 mmol, 1.0 equiv), $dmgH_2$ (5 mol%) and $Co_{1\%}$-$K_{11\%}$-PHI (2 mg, 0.17 mol% Co) in toluene (2 mL) for 48 h, the product **28** was isolated as yellow oil after flash chromatography (petroleum ether/ethyl acetate 20/1), 36.1 mg (68% yield). **^{1}H NMR** (400 MHz, $CDCl_3$) δ 7.78 – 7.37 (m, 2H), 7.00 (t, *J* = 8.7 Hz, 2H), 6.42 (d, *J* = 15.8 Hz, 1H), 6.06 (dt, *J* = 14.3, 6.1 Hz, 1H), 3.95 (s, 2H), 2.86 (s, 3H), 1.48 (s, 9H). **^{13}C NMR** (101 MHz, $CDCl_3$) δ 162.3 (d, *J* = 246.7 Hz), 155.7, 132.9 (d, *J* = 3.3 Hz), 130.8, 127.9 (d, *J* = 8.0 Hz), 125.1, 115.5 (d, *J* = 21.7 Hz), 79.6, 50.7, 33.8, 28.5. **^{19}F NMR** (377 MHz, $CDCl_3$) δ -114.5 (s). **HRMS (ESI)** m/z calcd. For $C_{15}H_{20}FNNaO_2$ $[M+Na]^+$ 288.1370, found 288.1370.

***tert*-butyl (*E*)-(3-(4-chlorophenyl)allyl)(methyl)carbamate (29)**

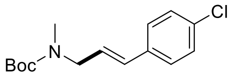

29

According to general procedure starting from *N*-Boc-*N*-methylglycine (0.2 mmol, 1.0 equiv), 1-chloro-4-vinylbenzene (1.0 mmol, 5.0 equiv), Et_3N (0.2 mmol, 1.0 equiv), $dmgH_2$ (5 mol%) and $Co_{1\%}$-$K_{11\%}$-PHI (2 mg, 0.17 mol% Co) in toluene (2 mL) for 48 h, the product **29** was isolated as colorless oil after flash chromatography (petroleum ether/ethyl acetate 30/1), 41 mg (73% yield). **^{1}H NMR** (400 MHz, $CDCl_3$) δ 7.27 (d, *J* = 6.8 Hz, 4H), 6.41 (d, *J* = 15.9 Hz, 1H), 6.12 (dt, *J* = 16.1, 6.2 Hz, 1H), 3.97 (s, 2H), 2.86 (s, 3H), 1.48 (s, 9H). **^{13}C NMR** (101 MHz, $CDCl_3$) δ 155.7, 135.3, 133.2, 130.6, 128.7, 127.6, 126.1, 79.6, 50.9, 33.9, 28.5. **HRMS (ESI)** m/z calcd. For $C_{15}H_{20}ClNNaO_2$ $[M+Na]^+$ 304.1075, found 304.1075.

***tert*-butyl (*E*)-(3-(4-bromophenyl)allyl)(methyl)carbamate[27] (30)**

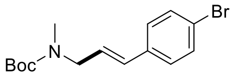

30

According to general procedure starting from *N*-Boc-*N*-methylglycine (0.2 mmol, 1.0 equiv), 1-bromo-4-vinylbenzene (1.0 mmol, 5.0 equiv), Et_3N (0.2 mmol, 1.0 equiv), $dmgH_2$ (5 mol%) and $Co_{1\%}$-$K_{11\%}$-PHI (2 mg, 0.17 mol% Co) in toluene (2 mL) for 48 h, the product **30** was isolated as yellow oil after flash chromatography (petroleum ether/ethyl acetate 20/1), 51 mg (78% yield). **^{1}H NMR** (400 MHz, $CDCl_3$) δ 7.48 – 7.37 (m, 2H), 7.25 – 7.19 (m, 2H), 6.39 (d, *J* = 15.9 Hz, 1H), 6.14 (dt, *J* = 16.0, 6.1 Hz, 1H), 3.96 (s, 2H), 2.86 (s, 3H), 1.47 (s, 9H). **^{13}C NMR** (101 MHz, $CDCl_3$) δ 155.7, 135.7, 131.7, 130.5, 127.9, 126.2, 121.3, 79.6, 50.6, 33.9, 28.5.

***tert*-butyl (*E*)-methyl(3-(4-(trifluoromethyl)phenyl)allyl)carbamate (31)**

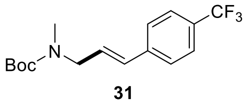

31

According to general procedure starting from *N*-Boc-*N*-methylglycine (0.2 mmol, 1.0 equiv), 1-(trifluoromethyl)-4-vinylbenzene (1.0 mmol, 5.0 equiv), Et_3N (0.2 mmol, 1.0 equiv), $dmgH_2$ (5 mol%) and $Co_{1\%}$-$K_{11\%}$-PHI (2 mg, 0.17 mol% Co) in toluene (2 mL) for 48 h, the product **31** was isolated as yellow oil after flash chromatography (petroleum ether/ethyl acetate 20/1), 50.5 mg (80% yield). **^{1}H NMR** (400 MHz, $CDCl_3$) δ 7.78 – 7.37 (m, 2H), 7.00 (t, *J* = 8.7 Hz, 2H), 6.42 (d, *J* = 15.8 Hz, 1H), 6.06 (dt, *J* = 14.3, 6.1

Hz, 1H), 3.95 (s, 2H), 2.86 (s, 3H), 1.48 (s, 9H). **^{13}C NMR** (101 MHz, $CDCl_3$) δ 155.7, 140.2, 130.3, 129.4 (q, J = 32.4 Hz), 128.2 (q, J = 2.5 Hz), 126.5, 125.5 (q, J = 4.1 Hz), 121.5 (q, J = 273.2 Hz), 79.7, 50.7, 34.0, 28.4. **^{19}F NMR** (377 MHz, $CDCl_3$) δ -62.5 (s). **HRMS (ESI)** m/z calcd. For $C_{16}H_{20}F_3NNaO_2$ $[M+Na]^+$ 338.1338, found 338.1339.

Methyl (*E*)-4-(3-((*tert*-butoxycarbonyl)(methyl)amino)prop-1-en-1-yl)benzoate[27] (32)

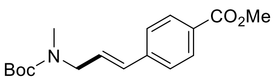

32

According to general procedure starting from *N*-Boc-*N*-methylglycine (0.2 mmol, 1.0 equiv), methyl 4-vinylbenzoate (1.0 mmol, 5.0 equiv), Et_3N (0.2 mmol, 1.0 equiv), $dmgH_2$ (5 mol%) and $Co_{1\%}$-$K_{11\%}$-PHI (2 mg, 0.17 mol% Co) in toluene (2 mL) for 48 h, the product **32** was isolated as yellow oil after flash chromatography (petroleum ether/ethyl acetate 10/1), 30.5 mg (50% yield). **^{1}H NMR** (400 MHz, $CDCl_3$) δ 7.98 (d, J = 8.0 Hz, 2H), 7.42 (d, J = 8.0 Hz, 2H), 6.49 (d, J = 15.9 Hz, 1H), 6.28 (dd, J = 14.2, 7.7 Hz, 1H), 4.01 (s, 2H), 3.91 (s, 3H), 2.88 (s, 3H), 1.48 (s, 9H). **^{13}C NMR** (101 MHz, $CDCl_3$) δ 166.9, 155.7, 141.2, 130.9, 129.9, 129.0, 128.2, 126.2, 79.7, 52.1, 50.8, 34.0, 28.5.

(*E*)-4-(3-((*tert*-butoxycarbonyl)(methyl)amino)prop-1-en-1-yl)phenyl acetate (33)

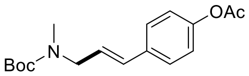

33

According to general procedure starting from *N*-Boc-*N*-methylglycine (0.2 mmol, 1.0 equiv), 4-vinylphenyl acetate (1.0 mmol, 5.0 equiv), Et_3N (0.2 mmol, 1.0 equiv), $dmgH_2$ (5 mol%) and $Co_{1\%}$-$K_{11\%}$-PHI (2 mg, 0.17 mol% Co) in toluene (2 mL) for 48 h, the product **33** was isolated as yellow oil after flash chromatography (petroleum ether/ethyl acetate 20/1), 45 mg (74% yield). **^{1}H NMR** (400 MHz, $CDCl_3$) δ 7.37 (d, J = 8.6 Hz, 2H), 7.04 (d, J = 8.6 Hz, 2H), 6.44 (d, J = 15.9 Hz, 1H), 6.10 (t, J = 8.3 Hz, 1H), 3.97 (s, 2H), 2.86 (s, 3H), 2.30 (s, 3H), 1.48 (s, 9H). **^{13}C NMR** (101 MHz, $CDCl_3$) δ 169.5, 155.8, 150.0, 134.6, 127.3, 125.6, 121.7, 79.6, 50.8, 33.8, 28.5, 21.1. **HRMS (ESI)** m/z calcd. For $C_{17}H_{23}NNaO_4$ $[M+Na]^+$ 328.1519, found 328.1524.

***tert*-butyl (*E*)-(3-(3-chlorophenyl)allyl)(methyl)carbamate (34)**

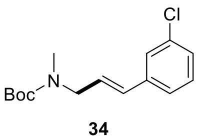

34

According to general procedure starting from *N*-Boc-*N*-methylglycine (0.2 mmol, 1.0 equiv), 1-chloro-3-vinylbenzene (1.0 mmol, 5.0 equiv), Et_3N (0.2 mmol, 1.0 equiv), $dmgH_2$ (5 mol%) and $Co_{1\%}$-$K_{11\%}$-PHI (2 mg, 0.17 mol% Co) in toluene (2 mL) for 48 h, the product **34** was isolated as yellow oil after flash chromatography (petroleum ether/ethyl acetate 20/1), 40 mg (71% yield). **^{1}H NMR** (400 MHz, $CDCl_3$) δ 7.51 (dd, J = 7.6, 1.9 Hz, 1H), 7.35 (dd, J = 7.6, 1.7 Hz, 1H), 7.23 – 7.15 (m, 2H), 6.95 – 6.77 (m, 1H), 6.12 (dt, J = 15.9, 6.2 Hz, 1H), 4.01 (s, 2H), 2.89 (s, 3H), 1.48 (s, 9H). **^{13}C NMR** (101 MHz, $CDCl_3$) δ 155.7, 134.9, 132.9, 129.7, 128.6, 128.1, 126.9, 126.9, 79.7, 51.1, 33.9, 28.5. **HRMS (ESI)** m/z calcd. For $C_{15}H_{20}ClNNaO_2$ $[M+Na]^+$ 304.1075, found 304.1080.

***tert*-butyl (*E*)-(3-(2-chlorophenyl)allyl)(methyl)carbamate (35)**

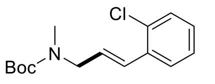

35

According to general procedure starting from *N*-Boc-*N*-methylglycine (0.2 mmol, 1.0 equiv), (*E*)-1-chloro-2-(prop-1-en-1-yl)benzene (1.0 mmol, 5.0 equiv), Et_3N (0.2 mmol, 1.0 equiv), $dmgH_2$ (5 mol%) and $Co_{1\%}$-$K_{11\%}$-PHI (2 mg, 0.17 mol% Co) in toluene (2 mL) for 48 h, the product **35** was isolated as yellow oil after flash chromatography (petroleum ether/ethyl acetate 20/1), 36.5 mg (65% yield). **^{1}H NMR** (400 MHz, $CDCl_3$) δ 7.38 – 7.32 (m, 1H), 7.25 – 7.16 (m, 3H), 6.39 (d, *J* = 15.9 Hz, 1H), 6.16 (dt, *J* = 15.1, 5.8 Hz, 1H), 3.98 (s, 2H), 2.86 (s, 3H), 1.48 (s, 9H). **^{13}C NMR** (101 MHz, $CDCl_3$) δ 155.7, 138.6, 134.5, 130.2, 129.8, 127.5, 127.0, 126.3, 124.6, 79.7, 50.2, 33.9, 28.5. **HRMS (ESI)** m/z calcd. For $C_{15}H_{20}ClNNaO_2$ $[M+Na]^+$ 304.1075, found 304.1080.

(*E*)-4-(3-((*tert*-butoxycarbonyl)(methyl)amino)prop-1-en-1-yl)-2-methoxyphenyl acetate (36)

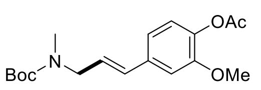

36

According to general procedure starting from *N*-Boc-*N*-methylglycine (0.2 mmol, 1.0 equiv), 2-methoxy-4-vinylphenyl acetate (1.0 mmol, 5.0 equiv), Et_3N (0.2 mmol, 1.0 equiv), $dmgH_2$ (5 mol%) and $Co_{1\%}$-$K_{11\%}$-PHI (2 mg, 0.17 mol% Co) in toluene (2 mL) for 48 h, the product **36** was isolated as yellow oil after flash chromatography (petroleum ether/ethyl acetate 5/1), 48.5 mg (72% yield). **^{1}H NMR** (400 MHz, $CDCl_3$) δ 7.12 – 6.83 (m, 3H), 6.42 (d, *J* = 15.8 Hz, 1H), 6.09 (d, *J* = 16.0 Hz, 1H), 3.98 (d, *J* = 6.0 Hz, 2H), 3.85 (s, 3H), 2.86 (s, 3H), 2.31 (s, 3H), 1.48 (s, 9H). **^{13}C NMR** (101 MHz, $CDCl_3$) δ 169.1, 155.8, 151.1, 139.2, 135.8, 131.3, 126.3, 122.8, 119.1, 110.0, 79.6, 55.9, 50.7, 33.8, 28.5, 20.7. **HRMS (ESI)** m/z calcd. For $C_{18}H_{25}NNaO_5$ $[M+Na]^+$ 358.1625, found 358.1633.

(*E*)-4-(3-((*tert*-butoxycarbonyl)amino)prop-1-en-1-yl)-2-methoxyphenyl acetate (37)

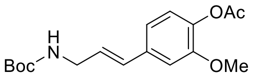

37

According to general procedure starting from *N*-Boc-*N*-glycine (0.2 mmol, 1.0 equiv), 2-methoxy-4-vinylphenyl acetate (1.0 mmol, 5.0 equiv), Et_3N (0.2 mmol, 1.0 equiv), $dmgH_2$ (10 mol%) and $Co_{1\%}$-$K_{11\%}$-PHI (4 mg, 0.34 mol% Co) in toluene (2 mL) for 48 h, the product **37** was isolated as white solid after flash chromatography (petroleum ether/ethyl acetate 5/1), 26 mg (40% yield). mp = 76–78 °C. **^{1}H NMR** (400 MHz, $CDCl_3$) δ 7.06 – 6.85 (m, 3H), 6.47 (d, *J* = 15.8 Hz, 1H), 6.14 (dt, *J* = 15.7, 6.1 Hz, 1H), 4.68 (s, 1H), 3.91 (d, *J* = 6.4 Hz, 2H), 3.84 (s, 3H), 2.30 (s, 3H), 1.47 (s, 9H). **^{13}C NMR** (101 MHz, $CDCl_3$) δ 169.1, 155.8, 151.1, 139.3, 135.8, 130.9, 126.8, 122.8, 119.1, 110.0, 79.6, 55.9, 42.7, 28.4, 20.7. **HRMS (ESI)** m/z calcd. For $C_{17}H_{23}NNaO_5$ $[M+Na]^+$ 344.1468, found 344.1476.

***tert*-butyl (*R*,*E*)-2-(4-acetoxy-3-methoxystyryl)pyrrolidine-1-carboxylate (38)**

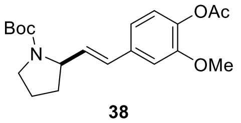

38

According to general procedure starting from *N*-Boc-*N*-D-proline (0.2 mmol, 1.0 equiv), 2-methoxy-4-vinylphenyl acetate (1.0 mmol, 5.0 equiv), Et_3N (0.2 mmol, 1.0 equiv), $dmgH_2$ (10 mol%) and $Co_{1\%}$-$K_{11\%}$-PHI (4 mg, 0.34 mol% Co) in toluene (2 mL) for 48 h, the product **38** was isolated as yellow oil after flash chromatography (petroleum

ether/ethyl acetate 5/1), 30.5 mg (42% yield). **^{1}H NMR** (400 MHz, $CDCl_3$) δ 7.07 – 6.86 (m, 3H), 6.35 (d, *J* = 15.3 Hz, 1H), 6.04 (d, *J* = 17.3 Hz, 1H), 4.63 – 4.31 (m, 1H), 3.84 (s, 3H), 3.46 (s, 2H), 2.30 (s, 3H), 2.17 – 2.03 (m, 1H), 1.97 – 1.82 (m, 2H), 1.85 – 1.73 (m, 1H), 1.43 (s, 9H). **^{13}C NMR** (101 MHz, $CDCl_3$) δ 169.1, 154.7, 151.1, 139.0, 136.2, 131.1, 128.8, 122.8, 118.9, 110.0, 79.3, 58.8, 55.9, 46.3, 32.5, 28.5, 23.0, 20.7. **HRMS (ESI)** m/z calcd. For $C_{20}H_{27}NNaO_5$ $[M+Na]^+$ 384.1781, found 384.1786.

***tert*-butyl (*E*)-(3-(3-(trifluoromethyl)phenyl)allyl)carbamate**[27] **(39)**

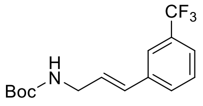

39

According to general procedure starting from *N*-Boc-*N*-glycine (0.2 mmol, 1.0 equiv), 1-(trifluoromethyl)-3-vinylbenzene (1.0 mmol, 5.0 equiv), Et_3N (0.2 mmol, 1.0 equiv), $dmgH_2$ (5 mol%) and $Co_{1\%}$-$K_{11\%}$-PHI (2 mg, 0.17 mol% Co) in toluene (2 mL) for 48 h, the product **39** was isolated as white solid after flash chromatography (petroleum ether/ethyl acetate 20/1), 41 mg (68% yield). **^{1}H NMR** (400 MHz, $CDCl_3$) δ 7.59 (s, 1H), 7.49 (dd, *J* = 15.0, 7.7 Hz, 2H), 7.40 (t, *J* = 7.8 Hz, 1H), 6.53 (dt, *J* = 15.8, 1.7 Hz, 1H), 6.27 (dt, *J* = 15.9, 5.9 Hz, 1H), 4.80 (br, 1H), 3.93 (t, *J* = 6.1 Hz, 2H), 1.47 (s, 9H). **^{13}C NMR** (101 MHz, $CDCl_3$) δ 155.8, 137.5, 131 (q, *J* = 32.0 Hz), 129.8, 129.5 (q, *J* = 1.6 Hz), 129.0, 128.7, 124.1 (q, *J* = 3.7 Hz), 123.0 (q, *J* = 4.0 Hz), 124.1 (q, *J* = 272.3 Hz), 79.6, 42.5, 28.4. **^{19}F NMR** (377 MHz, $CDCl_3$) δ -62.8 (s).

***tert*-butyl (*E*)-methyl(3-(naphthalen-1-yl)allyl)carbamate**[28] **(40)**

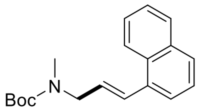

40

According to general procedure starting from *N*-Boc-*N*-methylglycine (0.2 mmol, 1.0 equiv), 1-vinylnaphthalene (1.0 mmol, 5.0 equiv), Et_3N (0.2 mmol, 1.0 equiv), $dmgH_2$ (5 mol%) and $Co_{1\%}$-$K_{11\%}$-PHI (2 mg, 0.17 mol% Co) in toluene (2 mL) for 48 h, the product **40** was isolated as yellow oil after flash chromatography (petroleum ether/ethyl acetate 20/1), 37.4 mg (63% yield). **^{1}H NMR** (400 MHz, $CDCl_3$) δ 8.13 – 8.06 (m, 1H), 7.84 (dd, *J* = 7.8, 1.8 Hz, 1H), 7.77 (d, *J* = 8.1 Hz, 1H), 7.57 (d, *J* = 7.1 Hz, 1H), 7.53 – 7.40 (m, 3H), 7.21 (d, *J* = 15.7 Hz, 1H), 6.17 (dt, *J* = 15.3, 6.0 Hz, 1H), 4.09 (s, 2H), 2.95 (s, 3H), 1.50 (s, 9H). **^{13}C NMR** (101 MHz, $CDCl_3$) δ 155.8, 134.6, 133.6, 131.1, 129.3, 128.6, 128.0, 126.1, 125.8, 125.6, 124.0, 123.7, 79.6, 51.4, 33.9, 28.5.

***tert*-butyl (*E*)-methyl(3-(naphthalen-2-yl)allyl)carbamate**[28] **(41)**

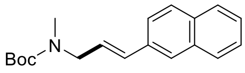

41

According to general procedure starting from *N*-Boc-*N*-methylglycine (0.2 mmol, 1.0 equiv), 2-vinylnaphthalene (1.0 mmol, 5.0 equiv), Et_3N (0.2 mmol, 1.0 equiv), $dmgH_2$ (5 mol%) and $Co_{1\%}$-$K_{11\%}$-PHI (2 mg, 0.17 mol% Co) in toluene (2 mL) for 48 h, the product **41** was isolated as yellow oil after flash chromatography (petroleum ether/ethyl acetate 20/1), 43 mg (72% yield). **^{1}H NMR** (400 MHz, $CDCl_3$) δ 7.82 – 7.75 (m, 3H), 7.73 – 7.68 (m, 1H), 7.58 (dd, *J* = 8.6, 1.8 Hz, 1H), 7.50 – 7.39 (m, 2H), 6.62 (d, *J* = 15.8 Hz, 1H), 6.28 (dd, *J* = 14.0, 7.4 Hz, 1H), 4.03 (s, 2H), 2.89 (s, 3H), 1.49 (s, 9H). **^{13}C NMR** (101 MHz, $CDCl_3$) δ 155.8, 134.2, 133.6, 133.0, 132.0, 128.2, 128.0, 127.7, 126.3, 126.2, 125.9, 125.7, 123.6, 79.6, 50.9, 33.9, 28.5.

***tert*-butyl (*E*)-methyl(3-(2-methylbenzo[d]oxazol-5-yl)allyl)carbamate (42)**

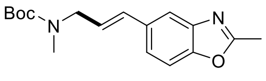

42

According to general procedure starting from *N*-Boc-*N*-methylglycine (0.2 mmol, 1.0 equiv), 2-methyl-5-vinylbenzo[*d*]oxazole (1.0 mmol, 5.0 equiv), Et_3N (0.2 mmol, 1.0 equiv), $dmgH_2$ (5 mol%) and $Co_{1\%}$-$K_{11\%}$-PHI (2 mg, 0.17 mol% Co) in toluene (2 mL) for 48 h, the product **42** was isolated as yellow oil after flash chromatography (petroleum ether/ethyl acetate 5/1), 41 mg (68% yield). **^{1}H NMR** (400 MHz, $CDCl_3$) δ 7.65 (s, 1H), 7.45 (s, 1H), 7.31 (d, *J* = 8.4 Hz, 1H), 6.55 (d, *J* = 15.8 Hz, 1H), 6.15 (dd, *J* = 14.6, 7.4 Hz, 1H), 3.99 (s, 2H), 2.88 (s, 3H), 2.65 (s, 3H), 1.49 (s, 9H). **^{13}C NMR** (101 MHz, $CDCl_3$) δ 155.8, 150.7, 144.8, 133.4, 131.3, 124.7, 123.2, 117.1, 110.2, 80.6, 50.7, 33.8, 28.5, 14.5. **HRMS (ESI)** m/z calcd. For $C_{17}H_{23}N_2O_3$ $[M+H]^+$ 303.1703, found 3030.1711.

***tert*-butyl (3,3-diphenylallyl)(methyl)carbamate (43)**

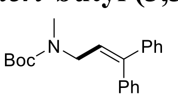

43

According to general procedure starting from *N*-Boc-*N*-methylglycine (0.2 mmol, 1.0 equiv), ethene-1,1-diyldibenzene (0.4 mmol, 2.0 equiv), Et_3N (0.2 mmol, 1.0 equiv), $dmgH_2$ (5 mol%) and $Co_{1\%}$-$K_{11\%}$-PHI (2 mg, 0.17 mol% Co) in toluene (2 mL) for 48 h, the product **43** was isolated as yellow oil after flash chromatography (petroleum ether/ethyl acetate 30/1), 61 mg (94% yield). **^{1}H NMR** (400 MHz, $CDCl_3$) δ 7.41 – 7.30 (m, 3H), 7.27 – 7.11 (m, 7H), 6.05 (t, *J* = 6.5 Hz, 1H), 3.91 (s, 2H), 2.77 (s, 3H), 1.45 (s, 9H). **^{13}C NMR** (101 MHz, $CDCl_3$) δ 163.1, 155.7, 144.2, 141.8, 139.2, 129.8, 128.4, 128.2, 127.5, 127.4, 125.3, 79.5, 48.0, 33.7, 28.5. **HRMS (ESI)** m/z calcd. For $C_{21}H_{25}NNaO_2$ $[M+Na]^+$ 346.1778, found 346.1786.

***tert*-butyl (3,3-diphenylallyl)carbamate[29] (44)**

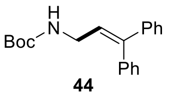

44

According to general procedure starting from *N*-Boc-*N*-glycine (0.2 mmol, 1.0 equiv), ethene-1,1-diyldibenzene (0.4 mmol, 2.0 equiv), Et_3N (0.2 mmol, 1.0 equiv), $dmgH_2$ (5 mol%) and $Co_{1\%}$-$K_{11\%}$-PHI (2 mg, 0.17 mol% Co) in toluene (2 mL) for 48 h, the product **44** was isolated as white solid after flash chromatography (petroleum ether/ethyl acetate 20/1), 45.1 mg (73% yield). **^{1}H NMR** (400 MHz, $CDCl_3$) 7.44 – 7.31 (m, 3H), 7.28 – 7.19 (m, 5H), 7.19 – 7.10 (m, 2H), 6.08 (t, *J* = 6.9 Hz, 1H), 4.61 (s, 1H), 3.81 (t, *J* = 6.5 Hz, 2H), 1.44 (s, 9H). **^{13}C NMR** (101 MHz, $CDCl_3$) δ 155.8, 148.1, 144.1, 141.8, 139.1, 129.7, 128.4, 128.2, 128.2, 127.5, 127.5, 126.9, 125.9, 125.4, 79.4, 40.0, 28.4.

1-(3-(phenylamino)phenyl)ethan-1-one (45)

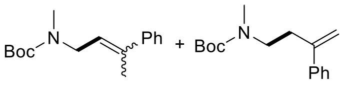

45 **45'**

According to general procedure starting from *N*-Boc-*N*-methylglycine (0.2 mmol, 1.0 equiv), prop-1-en-2-ylbenzene (1.0 mmol, 5.0 equiv), Et_3N (0.2 mmol, 1.0 equiv), $dmgH_2$ (5 mol%) and $Co_{1\%}$-$K_{11\%}$-PHI (2 mg, 0.17 mol% Co) in toluene (2 mL) for 48 h. **HRMS (ESI)** m/z calcd. For $C_{16}H_{24}NO_2$ $[M+H]^+$ 262.1802, found 262.1807.

(*E*)-1-methyl-4-(3-phenoxyprop-1-en-1-yl)benzene[30] (46)

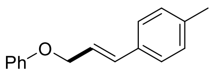

46

According to general procedure starting from 2-phenoxyacetic acid (0.2 mmol, 1.0 equiv), 1-methyl-4-vinylbenzene (0.4 mmol, 2.0 equiv), Et_3N (0.2 mmol, 1.0 equiv), $dmgH_2$ (5 mol%) and $Co_{1\%}$-$K_{11\%}$-PHI (2 mg, 0.17 wt % Co) in toluene (2 mL) for 48 h, the product **46** was isolated as yellow oil after flash chromatography (petroleum ether/ethyl acetate 80/1), 33.2 mg (74% yield). **^{1}H NMR** (400 MHz, $CDCl_3$) δ 7.33 – 7.24 (m, 4H), 7.14 – 7.10 (m, 2H), 6.98 – 6.92 (m, 3H), 6.69 (d, *J* = 15.7 Hz, 1H), 6.36 (dt, *J* = 15.9, 5.9 Hz, 1H), 4.67 (dd, *J* = 5.9, 1.5 Hz, 2H), 2.33 (s, 3H). **^{13}C NMR** (101 MHz, $CDCl_3$) δ 157.0, 136.1, 132.0, 131.4, 127.8, 127.6, 124.9, 121.8, 119.2, 113.1, 68.3, 19.6.

(*E*)-1-fluoro-4-(3-phenoxyprop-1-en-1-yl)benzene[30] (47)

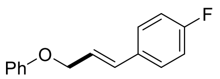

47

According to general procedure starting from 2-phenoxyacetic acid (0.2 mmol, 1.0 equiv), 1-fluoro-4-vinylbenzene (0.4 mmol, 2.0 equiv), Et_3N (0.2 mmol, 1.0 equiv), $dmgH_2$ (5 mol%) and $Co_{1\%}$-$K_{11\%}$-PHI (2 mg, 0.17 mol% Co) in toluene (2 mL) for 48 h, the product **47** was isolated as yellow oil after flash chromatography (petroleum ether/ethyl acetate 80/1), 34.7 mg (76% yield). **^{1}H NMR** (400 MHz, $CDCl_3$) δ 7.41 – 7.33 (m, 2H), 7.34 – 7.25 (m, 2H), 7.05 – 6.93 (m, 5H), 6.73 – 6.64 (m, 1H), 6.33 (dt, *J* = 15.9, 5.8 Hz, 1H), 4.68 (dd, *J* = 5.8, 1.6 Hz, 2H). **^{13}C NMR** (101 MHz, $CDCl_3$) δ 162.5 (d, *J* = 247.2 Hz), 158.6, 132.7 (d, *J* = 3.5 Hz), 131.8, 129.5, 128.1 (d, *J* = 8.0 Hz), 124.3 (d, *J* = 2.4 Hz), 121.0, 115.6 (d, *J* = 21.7 Hz), 114.8, 68.5. **^{19}F NMR** (377 MHz, $CDCl_3$) δ -114.0 (s).

(*E*)-1-chloro-4-(3-phenoxyprop-1-en-1-yl)benzene[30] (48)

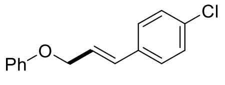

48

According to general procedure starting from 2-phenoxyacetic acid (0.2 mmol, 1.0 equiv), 1-chloro-4-vinylbenzene (0.4 mmol, 2.0 equiv), Et_3N (0.2 mmol, 1.0 equiv), $dmgH_2$ (5 mol%) and $Co_{1\%}$-$K_{11\%}$-PHI (2 mg, 0.17 mol% Co) in toluene (2 mL) for 48 h, the product **48** was isolated as yellow oil after flash chromatography (petroleum ether/ethyl acetate 80/1), 37.7 mg (77% yield). **^{1}H NMR** (400 MHz, $CDCl_3$) δ 7.36 – 7.25 (m, 6H), 6.99 – 6.91 (m, 3H), 6.68 (d, *J* = 16.0 Hz, 1H), 6.38 (dt, *J* = 16.0, 5.8 Hz, 1H), 4.68 (dd, *J* = 5.7, 1.6 Hz, 2H). **^{13}C NMR** (101 MHz, $CDCl_3$) δ 158.6, 135.0, 133.6, 131.6, 129.5, 128.8, 127.8, 125.3, 121.0, 114.8, 68.3.

(*E*)-1-bromo-4-(3-phenoxyprop-1-en-1-yl)benzene[31] (49)

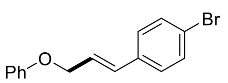

49

According to general procedure starting from 2-phenoxyacetic acid (0.2 mmol, 1.0 equiv), 1-bromo-4-vinylbenzene (0.4 mmol, 2.0 equiv), Et_3N (0.2 mmol, 1.0 equiv), $dmgH_2$ (5 mol%) and $Co_{1\%}$-$K_{11\%}$-PHI (2 mg, 0.17 mol% Co) in toluene (2 mL) for 48 h, the product **49** was isolated as yellow oil after flash chromatography (petroleum ether/ethyl acetate 80/1), 35.3 mg (61% yield). **^{1}H NMR** (400 MHz, $CDCl_3$) δ 7.52 – 7.41 (m, 2H), 7.34 – 7.23 (m, 4H), 7.01 – 6.91 (m, 3H), 6.68 (d, *J* = 16.0 Hz, 1H), 6.41 (dt, *J* = 16.0, 5.6 Hz, 1H), 4.68 (dd, *J* = 5.7, 1.6 Hz, 2H). **^{13}C NMR** (101 MHz, $CDCl_3$) δ 158.5, 135.4, 131.7, 131.6, 129.5, 128.1, 125.4, 121.7, 121.0, 114.8, 68.3.

(*E*)-1-(3-phenoxyprop-1-en-1-yl)-4-(trifluoromethyl)benzene[30] (50)

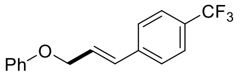

50

According to general procedure starting from 2-phenoxyacetic acid (0.2 mmol, 1.0 equiv), 1-(trifluoromethyl)-4-vinylbenzene (0.4 mmol, 2.0 equiv), Et_3N (0.2 mmol, 1.0 equiv), $dmgH_2$ (5 mol%) and $Co_{1\%}$-$K_{11\%}$-PHI (2 mg, 0.17 mol% Co) in toluene (2 mL) for 48 h, the product **50** was isolated as yellow oil after flash chromatography (petroleum ether/ethyl acetate 80/1), 36 mg (65% yield). **^{1}H NMR** (400 MHz, $CDCl_3$) δ 7.58 (d, *J* = 8.1 Hz, 2H), 7.50 (d, *J* = 8.2 Hz, 2H), 7.31 (t, *J* = 7.8 Hz, 2H), 7.03 – 6.89 (m, 3H), 6.77 (d, *J* = 16.0 Hz, 1H), 6.51 (dt, *J* = 16.0, 5.5 Hz, 1H), 4.73 (d, *J* = 5.4 Hz, 2H). **^{13}C NMR** (101 MHz, $CDCl_3$) δ 158.5, 140.0, 131.2, 129.7 (q, *J* = 32.6 Hz), 129.6, 127.4, 126.7, 125.6 (q, *J* = 3.9 Hz). 124.3 (q, *J* = 270.1 Hz), 121.1, 114.7, 68.1. **^{19}F NMR** (377 MHz, $CDCl_3$) δ -62.5 (s).

Methyl (*E*)-4-(3-phenoxyprop-1-en-1-yl)benzoate (51)

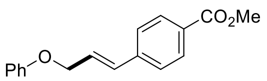

51

According to general procedure starting from 2-phenoxyacetic acid (0.2 mmol, 1.0 equiv), methyl 4-vinylbenzoate (0.4 mmol, 2.0 equiv), Et_3N (0.2 mmol, 1.0 equiv), $dmgH_2$ (5 mol%) and $Co_{1\%}$-$K_{11\%}$-PHI (2 mg, 0.17 mol% Co) in toluene (2 mL) for 48 h, the product **51** was isolated as white soild after flash chromatography (petroleum ether/ethyl acetate 50/1), 28 mg (52% yield). mp = 114 – 116 ºC. **^{1}H NMR** (400 MHz, $CDCl_3$) δ 8.03 – 7.96 (m, 2H), 7.51 – 7.40 (m, 2H), 7.36 – 7.27 (m, 2H), 7.01 – 6.93 (m, 3H), 6.78 (d, *J* = 16.0 Hz, 1H), 6.54 (dt, *J* = 16.0, 5.5 Hz, 1H), 4.73 (dd, *J* = 5.5, 1.6 Hz, 2H), 3.91 (s, 3H). **^{13}C NMR** (101 MHz, $CDCl_3$) δ 166.8, 158.5, 140.9, 131.6, 130.0, 129.6, 129.3, 127.4, 126.4, 121.1, 114.8, 68.2, 52.1. **HRMS (ESI)** m/z calcd. For $C_{17}H_{17}O_3$ $[M+H]^+$ 269.1172, found 269.1172.

(*E*)-4-(3-phenoxyprop-1-en-1-yl)phenyl acetate (52)

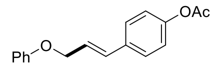

52

According to general procedure starting from 2-phenoxyacetic acid (0.2 mmol, 1.0 equiv), 4-vinylphenyl acetate (0.4 mmol, 2.0 equiv), Et_3N (0.2 mmol, 1.0 equiv), $dmgH_2$ (5 mol%) and $Co_{1\%}$-$K_{11\%}$-PHI (2 mg, 0.17 mol% Co) in toluene (2 mL) for 48 h, the product **52** was isolated as white soild after flash chromatography (petroleum ether/ethyl acetate 80/1), 42 mg (78% yield). mp = 104 – 106 ºC. **^{1}H NMR** (400 MHz, $CDCl_3$) δ 7.41 (d, *J* = 8.3 Hz, 2H), 7.29 (t, *J* = 7.8 Hz, 2H), 7.05 (d, *J* = 8.3 Hz, 2H), 7.01 – 6.91 (m, 3H), 6.71 (d, *J* = 15.9 Hz, 1H), 6.37 (dt, *J* = 16.0, 5.7 Hz, 1H), 4.69 (d, *J* = 5.7 Hz, 2H), 2.29 (s, 3H). **^{13}C NMR** (101 MHz, $CDCl_3$) δ 169.5, 158.6, 150.3, 134.3, 131.9, 129.5, 127.6, 124.8, 121.7, 121.0, 114.8, 68.5, 21.2. **HRMS (ESI)** m/z calcd. For $C_{17}H_{17}O_3$ $[M+H]^+$ 269.1172, found 269.1172.

(*E*)-1-chloro-3-(3-phenoxyprop-1-en-1-yl)benzene[30] (53)

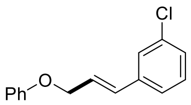

53

According to general procedure starting from 2-phenoxyacetic acid (0.2 mmol, 1.0 equiv), 1-chloro-3-vinylbenzene (0.4 mmol, 2.0 equiv), Et_3N (0.2 mmol, 1.0 equiv), $dmgH_2$ (5 mol%) and $Co_{1\%}$-$K_{11\%}$-PHI (2 mg, 0.17 mol% Co) in toluene (2 mL) for 48 h, the product **53** was isolated as yellow oil after flash chromatography (petroleum ether/ethyl acetate 80/1), 31 mg (64% yield). **^{1}H NMR** (400 MHz, $CDCl_3$) δ 7.39 (s, 1H), 7.35 – 7.26 (m, 3H),

7.26 – 7.21 (m, 2H), 7.01 – 6.92 (m, 3H), 6.72 – 6.63 (m, 1H), 6.43 (dt, J = 16.0, 5.6 Hz, 1H), 4.70 (dd, J = 5.5, 1.6 Hz, 2H). **^{13}C NMR** (101 MHz, $CDCl_3$) δ 158.5, 138.4, 134.6, 131.3, 129.8, 129.6, 127.8, 126.5, 126.2, 124.8, 121.0, 114.8, 68.2.

(*E*)-1-chloro-2-(3-phenoxyprop-1-en-1-yl)benzene (54)

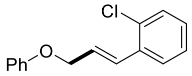

54

According to general procedure starting from 2-phenoxyacetic acid (0.2 mmol, 1.0 equiv), 1-chloro-2-vinylbenzene (0.4 mmol, 2.0 equiv), Et_3N (0.2 mmol, 1.0 equiv), $dmgH_2$ (5 mol%) and $Co_{1\%}$-$K_{11\%}$-PHI (2 mg, 0.17 mol% Co) in toluene (2 mL) for 48 h, the product **54** was isolated as yellow oil after flash chromatography (petroleum ether/ethyl acetate 80/1), 30 mg (62% yield). **^{1}H NMR** (400 MHz, $CDCl_3$) δ 7.57 (dd, J = 7.5, 2.0 Hz, 1H), 7.36 (dd, J = 7.6, 1.7 Hz, 1H), 7.33 – 7.28 (m, 2H), 7.26 – 7.17 (m, 2H), 7.17 – 7.09 (m, 1H), 7.01 – 6.93 (m, 3H), 6.41 (dt, J = 15.9, 5.8 Hz, 1H), 4.74 (dd, J = 5.9, 1.6 Hz, 2H). **^{13}C NMR** (101 MHz, $CDCl_3$) δ 158.6, 134.7, 133.2, 129.7, 129.5, 129.2, 128.9, 127.5, 127.0, 126.9, 121.0, 114.8, 68.5. **HRMS (ESI)** m/z calcd. For $C_{15}H_{14}ClO$ $[M+H]^+$ 245.0728, found 245.0727.

(*E*)-2-methoxy-4-(3-phenoxyprop-1-en-1-yl)phenyl acetate (55)

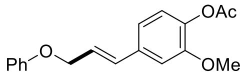

55

According to general procedure starting from 2-phenoxyacetic acid (0.2 mmol, 1.0 equiv), 2-methoxy-4-vinylphenyl acetate (1.0 mmol, 5.0 equiv), Et_3N (0.2 mmol, 1.0 equiv), $dmgH_2$ (5 mol%) and $Co_{1\%}$-$K_{11\%}$-PHI (2 mg, 0.17 mol% Co) in toluene (2 mL) for 48 h, the product **55** was isolated as white soild after flash chromatography (petroleum ether/ethyl acetate 20/1), 38.5 mg (64% yield). mp = 78 – 80 °C. **^{1}H NMR** (400 MHz, $CDCl_3$) δ 7.34 – 7.26 (m, 2H), 7.02 – 6.92 (m, 6H), 6.74 – 6.65 (m, 1H), 6.37 (dt, J = 15.9, 5.7 Hz, 1H), 4.69 (dd, J = 5.8, 1.6 Hz, 2H), 3.84 (s, 3H), 2.31 (s, 3H). **^{13}C NMR** (101 MHz, $CDCl_3$) δ 169.1, 158.6, 151.1, 139.5, 135.6, 132.2, 129.5, 124.9, 122.9, 121.0, 119.3, 114.8, 110.2, 68.4, 55.9, 20.7. **HRMS (ESI)** m/z calcd. For $C_{18}H_{19}O_4$ $[M+H]^+$ 299.1278, found 299.1277.

(*E*)-2-(3-phenoxyprop-1-en-1-yl)pyridine (56)

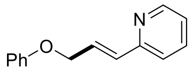

56

According to general procedure starting from 2-phenoxyacetic acid (0.2 mmol, 1.0 equiv), 2-vinylpyridine (1.0 mmol, 5.0 equiv), Et_3N (0.2 mmol, 1.0 equiv), $dmgH_2$ (5 mol%) and $Co_{1\%}$-$K_{11\%}$-PHI (2 mg, 0.17 mol% Co) in toluene (2 mL) for 48 h, the product **56** was isolated as yellow oil after flash chromatography (petroleum ether/ethyl acetate 5/1), 20.3 mg (48% yield). **^{1}H NMR** (400 MHz, $CDCl_3$) δ 8.56 (d, J = 5.8 Hz, 1H), 7.63 (td, J = 7.7, 1.9 Hz, 1H), 7.34 – 7.26 (m, 3H), 7.18 – 7.08 (m, 1H), 7.04 – 6.87 (m, 4H), 6.83 (d, J = 15.9 Hz, 1H), 4.77 (dd, J = 4.9, 1.5 Hz, 2H). **^{13}C NMR** (101 MHz, $CDCl_3$) δ 158.6, 154.9, 149.6, 136.6, 131.7, 129.5, 129.3, 122.4, 121.8, 121.0, 114.8, 67.9. **HRMS (ESI)** m/z calcd. For $C_{14}H_{14}NO$ $[M+H]^+$ 212.1070, found 212.1075.

(*E*)-1-(3-phenoxyprop-1-en-1-yl)naphthalene (57)

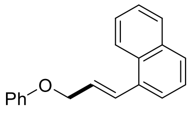

57

According to general procedure starting from 2-phenoxyacetic acid (0.2 mmol, 1.0 equiv), 1-vinylnaphthalene (1.0 mmol, 5.0 equiv), Et_3N (0.2 mmol, 1.0 equiv), $dmgH_2$ (5 mol%) and $Co_{1\%}$-$K_{11\%}$-PHI (2 mg, 0.17 mol% Co) in toluene (2 mL) for 48 h, the product **57** was isolated as yellow oil after flash chromatography (petroleum ether/ethyl acetate 20/1), 27.5 mg (53% yield). **^{1}H NMR** (400 MHz, $CDCl_3$) δ 8.23 – 8.03 (m, 1H), 7.86 – 7.82 (m, 1H), 7.78 (d, *J* = 8.2 Hz, 1H), 7.62 (d, *J* = 7.1 Hz, 1H), 7.52 – 7.48 (m, 2H), 7.47 – 7.41 (m, 2H), 7.36 – 7.25 (m, 2H), 7.05 – 6.93 (m, 3H), 6.44 (dt, *J* = 15.7, 5.6 Hz, 1H), 4.80 (dd, *J* = 5.6, 1.6 Hz, 2H). **^{13}C NMR** (101 MHz, $CDCl_3$) δ 158.7, 134.3, 133.6, 131.2, 130.1, 129.6, 128.6, 128.2, 127.8, 126.1, 125.8, 125.6, 124.1, 123.8, 121.0, 114.9, 68.7. **HRMS (ESI)** m/z calcd. For $C_{19}H_{17}O$ $[M+H]^+$ 261.1274, found 261.1275.

(*E*)-2-(3-phenoxyprop-1-en-1-yl)naphthalene[30] (58)

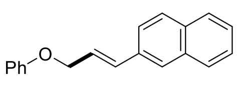

58

According to general procedure starting from 2-phenoxyacetic acid (0.2 mmol, 1.0 equiv), 2-vinylnaphthalene (1.0 mmol, 5.0 equiv), Et_3N (0.2 mmol, 1.0 equiv), $dmgH_2$ (5 mol%) and $Co_{1\%}$-$K_{11\%}$-PHI (2 mg, 0.17 mol% Co) in toluene (2 mL) for 48 h, the product **58** was isolated as yellow oil after flash chromatography (petroleum ether/ethyl acetate 20/1), 31 mg (60% yield). **^{1}H NMR** (400 MHz, $CDCl_3$) δ 7.86 – 7.74 (m, 4H), 7.63 (dd, *J* = 8.6, 1.8 Hz, 1H), 7.49 – 7.42 (m, 2H), 7.35 – 7.26 (m, 2H), 7.03 – 6.93 (m, 3H), 6.90 (d, *J* = 16.0 Hz, 1H), 6.55 (dt, *J* = 16.0, 5.8 Hz, 1H), 4.76 (dd, *J* = 5.7, 1.6 Hz, 2H). **^{13}C NMR** (101 MHz, $CDCl_3$) δ 158.7, 133.9, 133.6, 133.1, 133.1, 129.5, 128.3, 128.1, 127.7, 126.7, 126.3, 126.0, 124.9, 123.6, 121.0, 114.8, 68.6.

(*E*)-2-methyl-5-(3-phenoxyprop-1-en-1-yl)benzo[d]oxazole (59)

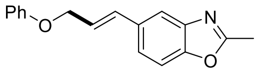

59

According to general procedure starting from 2-phenoxyacetic acid (0.2 mmol, 1.0 equiv), 2-methyl-5-vinylbenzo[*d*]oxazole (1.0 mmol, 5.0 equiv), Et_3N (0.2 mmol, 1.0 equiv), $dmgH_2$ (5 mol%) and $Co_{1\%}$-$K_{11\%}$-PHI (2 mg, 0.17 mol% Co) in toluene (2 mL) for 48 h, the product **59** was isolated as yellow oil after flash chromatography (petroleum ether/ethyl acetate 20/1), 36 mg (68% yield). mp = 68 – 70 °C. **^{1}H NMR** (400 MHz, $CDCl_3$) δ 7.68 (s, 1H), 7.45 – 7.26 (m, 4H), 7.03 – 6.92 (m, 3H), 6.89 – 6.76 (m, 1H), 6.43 (dt, *J* = 15.9, 5.8 Hz, 1H), 4.72 (dd, *J* = 5.8, 1.5 Hz, 2H), 2.64 (s, 3H). **^{13}C NMR** (101 MHz, $CDCl_3$) δ 164.7, 158.6, 150.6, 142.1, 133.2, 132.8, 129.5, 124.2, 123.4, 121.0, 117.3, 114.8, 110.2, 68.5, 14.6. **HRMS (ESI)** m/z calcd. For $C_{17}H_{16}NO_2$ $[M+H]^+$ 266.1176, found 266.1187.

(*R*)-3,7-dimethyloct-6-en-1-yl(*E*)-4-(3-((*tert*-butoxycarbonyl)(methyl)amino)prop-1-en-1-yl)benzoate (60)

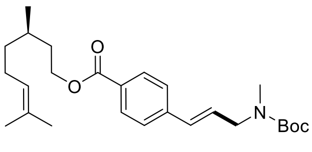

60

According to general procedure starting from *N*-Boc-*N*-methylglycine (0.1 mmol, 1.0 equiv), (*R*)-3,7-dimethyloct-6-en-1-yl 4-vinylbenzoate (0.5 mmol, 5.0 equiv), Et_3N (0.1 mmol, 1.0 equiv), $dmgH_2$ (5 mol%) and $Co_{1\%}$-$K_{11\%}$-PHI (2 mg, 0.34 mol% Co) in toluene (2 mL) for 48 h, the product **60** was isolated as yellow oil after flash chromatography (petroleum ether/ethyl acetate

25/1), 26 mg (60% yield). **^{1}H NMR** (400 MHz, $CDCl_3$) δ 7.98 (d, *J* = 8.1 Hz, 2H), 7.43 (s, 2H), 6.49 (d, *J* = 15.9 Hz, 1H), 6.27 (dt, *J* = 16.2, 6.1 Hz, 1H), 5.21 – 4.97 (m, 1H), 4.43 – 4.26 (m, 2H), 4.01 (s, 2H), 2.88 (s, 3H), 2.20 – 1.96 (m, 2H), 1.87 – 1.76 (m, 1H), 1.68 (s, 3H), 1.60 (s, 5H), 1.48 (s, 9H), 1.46 – 1.35 (m, 1H), 1.29 – 1.16 (m, 1H), 0.97 (d, *J* = 6.5 Hz, 3H). **^{13}C NMR** (101 MHz, $CDCl_3$) δ 166.4, 155.7, 141.1, 131.3, 130.7, 129.9, 129.4, 128.1, 126.2, 124.6, 79.7, 63.5, 50.3, 37.0, 35.5, 34.0, 29.6, 28.4, 25.7, 25.4, 19.5, 17.7. **HRMS (ESI)** m/z calcd. For $C_{26}H_{40}NO_4$ $[M+H]^+$ 430.2952, found 430.2940.

(*R*)-3,7-dimethyloct-6-en-1-yl (*E*)-4-(3-phenoxyprop-1-en-1-yl)benzoate (61)

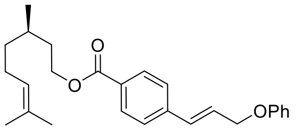

61

According to general procedure starting from 2-phenoxyacetic acid (0.1 mmol, 1.0 equiv), (*R*)-3,7-dimethyloct-6-en-1-yl 4-vinylbenzoate (0.5 mmol, 5.0 equiv), Et_3N (0.1 mmol, 1.0 equiv), $dmgH_2$ (5 mol%) and $Co_{1\%}$-$K_{11\%}$-PHI (2 mg, 0.34 mol% Co) in toluene (2 mL) for 48 h, the product **61** was isolated as colorless oil after flash chromatography (petroleum ether/ethyl acetate 50/1), 24.3 mg (62% yield). **^{1}H NMR** (400 MHz, $CDCl_3$) δ 8.01 (d, *J* = 8.0 Hz, 2H), 7.46 (d, *J* = 8.1 Hz, 2H), 7.36 – 7.26 (m, 2H), 7.01 – 6.93 (m, 3H), 6.78 (d, *J* = 16.0 Hz, 1H), 6.53 (dt, *J* = 16.0, 5.4 Hz, 1H), 5.84 (s, 1H), 4.76 – 4.69 (m, 2H), 4.46 – 4.26 (m, 2H), 2.06 – 1.92 (m, 2H), 1.85 – 1.77 (m, 1H), 1.68 (s, 3H), 1.64 – 1.54 (m, 5H), 1.46 – 1.36 (m, 1H), 1.27 – 1.20 (m, 1H), 0.97 (d, *J* = 6.5 Hz, 3H). **^{13}C NMR** (101 MHz, $CDCl_3$) δ 166.4, 158.5, 140.8, 131.7, 131.4, 129.9, 129.7, 129.6, 127.3, 126.4, 124.6, 121.1, 114.8, 68.2, 63.5, 37.0, 35.5, 29.6, 25.7, 25.4, 19.5, 17.7. **HRMS (ESI)** m/z calcd. For $C_{26}H_{33}O_3$ $[M+H]^+$ 393.2424, found 393.2432.

(*R*)-(4-(prop-1-en-2-yl)cyclohex-1-en-1-yl)methyl (*E*)-4-(3-((*tert*-butoxycarbonyl)(methyl)amino)prop-1-en-1-yl)benzoate (62)

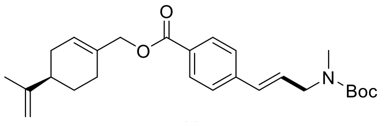

62

According to general procedure starting from *N*-Boc-*N*-methylglycine (0.1 mmol, 1.0 equiv), (*R*)-(4-(prop-1-en-2-yl) cyclohex-1-en-1-yl)methyl 4-vinylbenzoate (0.5 mmol, 5.0 equiv), Et_3N (0.1 mmol, 1.0 equiv), $dmgH_2$ (5 mol%) and $Co_{1\%}$-$K_{11\%}$-PHI (2 mg, 0.34 mol% Co) in toluene (2 mL) for 48 h, the product **62** was isolated as colorless oil after flash chromatography (petroleum ether/ethyl acetate 20/1), 23 mg (54% yield). **^{1}H NMR** (400 MHz, $CDCl_3$) δ 8.00 (d, *J* = 8.1 Hz, 2H), 7.42 (d, *J* = 8.1 Hz, 2H), 6.49 (d, *J* = 15.9 Hz, 1H), 6.27 (dt, *J* = 16.4, 6.0 Hz, 1H), 5.84 (s, 1H), 4.72 (d, *J* = 6.8 Hz, 4H), 4.01 (s, 2H), 2.88 (s, 3H), 2.23 – 2.13 (m, 4H), 2.08 – 1.93 (m, 1H), 1.91 – 1.82 (m, 1H), 1.75 (s, 3H), 1.63 – 1.53 (m, 1H), 1.48 (s, 9H). **^{13}C NMR** (101 MHz, $CDCl_3$) δ 166.2, 155.7, 149.6, 141.2, 132.7, 130.6, 130.0, 129.2, 128.2, 126.2, 125.6, 108.8, 79.7, 68.8, 50.9, 40.9, 34.0, 30.5, 28.5, 27.3, 26.5, 20.8. **HRMS (ESI)** m/z calcd. For $C_{26}H_{36}NO_4$ $[M+H]^+$ 426.2639, found 426.2636.

(*R*)-(4-(prop-1-en-2-yl)cyclohex-1-en-1-yl)methyl (*E*)-4-(3-phenoxyprop-1-en-1-yl)benzoate (63)

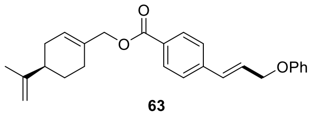
63

According to general procedure starting from 2-phenoxyacetic acid (0.1 mmol, 1.0 equiv), (*R*)-(4-(prop-1-en-2-yl)cyclohex-1-en-1-yl)methyl 4-vinylbenzoate (0.5 mmol, 5.0 equiv), Et_3N (0.1 mmol, 1.0 equiv), $dmgH_2$ (5 mol%) and $Co_{1\%}$-$K_{11\%}$-PHI (2 mg, 0.34 mol% Co) in toluene (2 mL) for 48 h, the product **63** was isolated as white solid after flash chromatography (petroleum ether/ethyl acetate 50/1), 23.3 mg (60% yield). mp = 61 – 63 °C. **^{1}H NMR** (400 MHz, $CDCl_3$) δ 8.01 (d, *J* = 8.0 Hz, 2H), 7.46 (d, *J* = 8.1 Hz, 2H), 7.36 – 7.26 (m, 2H), 7.01 – 6.93 (m, 3H), 6.78 (d, *J* = 16.0 Hz, 1H), 6.53 (dt, *J* = 16.0, 5.4 Hz, 1H), 5.84 (s, 1H), 4.76 – 4.69 (m, 6H), 2.25 – 2.13 (m, 4H), 2.06 – 1.92 (m, 1H), 1.92 – 1.82 (m, 1H), 1.75 (s, 3H), 1.58 – 1.48 (m, 1H). **^{13}C NMR** (101 MHz, $CDCl_3$) δ 166.2, 158.5, 149.6, 140.9, 132.7, 131.6, 130.0, 129.6, 129.5, 127.3, 126.4, 125.7, 121.1, 114.8, 108.8, 68.9, 68.2, 40.9, 30.5, 27.4, 26.5, 20.8. **HRMS (ESI)** m/z calcd. For $C_{26}H_{29}O_3$ $[M+H]^+$ 389.2111, found 389.2108.

(1*R*,2*S*,5*R*)-2-isopropyl-5-methylcyclohexyl 4-((*E*)-3-((*tert*-butoxycarbonyl)(methyl)amino)prop-1-en-1-yl)benzoate (64)

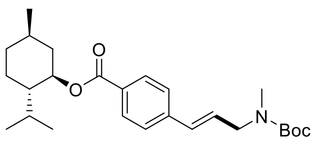
64

According to general procedure starting from *N*-Boc-*N*-methylglycine (0.1 mmol, 1.0 equiv), (1*R*,2*S*,5*R*)-2-isopropyl-5-methylcyclohexyl 4-vinylbenzoate (0.5 mmol, 5.0 equiv), Et_3N (0.1 mmol, 1.0 equiv), $dmgH_2$ (5 mol%) and $Co_{1\%}$-$K_{11\%}$-PHI (2 mg, 0.34 mol% Co) in toluene (2 mL) for 48 h, the product **64** was isolated as colorless oil after flash chromatography (petroleum ether/ethyl acetate 30/1), 26.3 mg (61% yield). **^{1}H NMR** (400 MHz, $CDCl_3$) δ 7.99 (d, *J* = 8.1 Hz, 2H), 7.42 (d, *J* = 8.2 Hz, 2H), 6.49 (d, *J* = 15.9 Hz, 1H), 6.26 (dt, *J* = 14.3, 5.5 Hz, 1H), 4.92 (td, *J* = 10.8, 4.4 Hz, 1H), 4.00 (s, 2H), 2.88 (s, 3H), 2.17 – 2.09 (m, 1H), 1.99 –1.92 (m, 1H), 1.79 – 1.69 (m, 2H), 1.62 – 1.41 (m, 12H), 1.19 – 1.03 (m, 2H), 0.95 – 0.90 (m, 6H), 0.79 (d, *J* = 6.9 Hz, 3H). **^{13}C NMR** (101 MHz, $CDCl_3$) δ 165.9, 155.7, 141.0, 130.9, 129.9, 129.7, 128.0, 126.2, 79.7, 74.8, 50.8, 47.3, 41.0, 34.3, 34.0, 31.5, 28.5, 26.5, 23.7, 22.1, 20.8, 16.6. **HRMS (ESI)** m/z calcd. For $C_{26}H_{39}NNaO_4$ $[M+Na]^+$ 452.2771, found 452.2781.

(1*R*,2*S*,5*R*)-2-isopropyl-5-methylcyclohexyl 4-((*E*)-3-phenoxyprop-1-en-1-yl)benzoate (65)

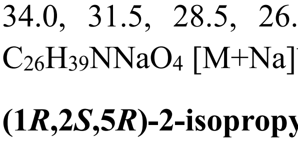
65

According to general procedure starting from 2-phenoxyacetic acid (0.1 mmol, 1.0 equiv), (1*R*,2*S*,5*R*)-2-isopropyl-5-methylcyclohexyl 4-vinylbenzoate (0.5 mmol, 5.0 equiv), Et_3N (0.1 mmol, 1.0 equiv), $dmgH_2$ (5 mol%) and $Co_{1\%}$-$K_{11\%}$-PHI (2 mg, 0.34 mol% Co) in toluene (2 mL) for 48 h, the product **65** was isolated as white solid after flash chromatography (petroleum ether/ethyl acetate 80/1), 27.5 mg (70% yield). mp = 52 – 54 °C. **^{1}H NMR** (400 MHz, $CDCl_3$) δ 8.00 (d, *J* = 8.3 Hz, 2H), 7.45 (d, *J* = 8.4 Hz, 2H), 7.31 (d, *J* = 7.7 Hz, 2H), 7.01 – 6.92 (m, 3H), 6.77 (d, *J* = 16.0 Hz, 1H), 6.52 (dt, *J* = 16.0, 5.5 Hz, 1H), 4.93 (td, *J* = 10.9, 4.4 Hz, 1H), 4.71 (dd, *J* = 5.5, 1.7 Hz, 2H), 2.17 – 2.07 (m, 1H), 2.02 – 1.88

(m, 1H), 1.77 – 1.62 (m, 2H), 1.63 – 1.47 (m, 2H), 1.30 – 1.05 (m, 3H), 0.92 (dd, *J* = 6.9, 3.4 Hz, 6H), 0.79 (d, *J* = 6.9 Hz, 3H). **^{13}C NMR** (101 MHz, $CDCl_3$) δ 165.9, 158.5, 140.8, 131.7, 130.0, 130.0, 129.6, 127.2, 126.4, 121.1, 114.8, 74.9, 68.3, 47.3, 41.0, 34.4, 31.5, 26.6, 23.7, 22.1, 20.8, 16.6. **HRMS (ESI)** m/z calcd. For $C_{26}H_{33}O_3$ $[M+H]^+$ 393.2424, found 393.2421.

(3a*S*,5*S*,6*S*,6a*S*)-5-((*R*)-2,2-dimethyl-1,3-dioxolan-4-yl)-2,2-dimethyltetrahydrofuro[2,3-*d*][1,3]dioxol-6-yl 4-((*E*)-3-((*tert*-butoxycarbonyl)(methyl)amino)prop-1- en-1-yl)benzoate (66)

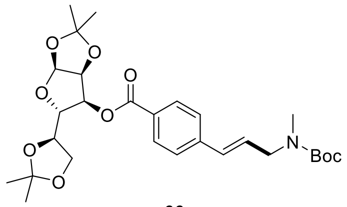

66

According to general procedure starting from *N*-Boc-*N*-methylglycine (0.1 mmol, 1.0 equiv), (3a*S*,5*S*,6*S*,6a*S*)-5-((*R*)-2,2-dimethyl-1,3-dioxolan- 4-yl)-2,2-dimethyltetrahydrofuro[2,3-*d*][1,3]dioxol-6-yl 4-vinylbenzoate (0.5 mmol, 5.0 equiv), Et_3N (0.1 mmol, 1.0 equiv), $dmgH_2$ (5 mol%) and $Co_{1\%}$-$K_{11\%}$-PHI (2 mg, 0.34 mol% Co) in toluene (2 mL) for 48 h, the product **66** was isolated as yellow oil after flash chromatography (petroleum ether/ethyl acetate 10/1), 37.9 mg (71% yield). **^{1}H NMR** (400 MHz, $CDCl_3$) δ 7.97 (d, *J* = 8.2 Hz, 2H), 7.43 (d, *J* = 8.4 Hz, 2H), 6.49 (d, *J* = 15.9 Hz, 1H), 6.29 (dt, *J* = 16.0, 6.1 Hz, 1H), 5.96 (d, *J* = 3.7 Hz, 1H), 5.50 (d, *J* = 2.6 Hz, 1H), 4.63 (d, *J* = 3.7 Hz, 1H), 4.38 – 4.30 (m, 2H), 4.10 (dd, *J* = 6.6, 4.9 Hz, 2H), 4.01 (s, 2H), 2.88 (s, 3H), 1.56 (s, 3H), 1.48 (s, 9H), 1.42 (s, 3H), 1.32 (s, 3H), 1.27 (s, 3H). **^{13}C NMR** (101 MHz, $CDCl_3$) δ 164.9, 155.7, 141.8, 130.1, 129.9, 128.7, 128.3, 126.4, 112.4, 109.4, 105.1, 83.4, 80.0, 79.8, 76.6, 72.6, 67.2, 50.7, 34.0, 28.5, 26.8, 26.7, 26.2, 25.2. **HRMS (ESI)** m/z calcd. For $C_{28}H_{39}NNaO_9$ $[M+Na]^+$ 556.2517, found 556.2528.

(3a*S*,5*S*,6*S*,6a*S*)-5-((*R*)-2,2-dimethyl-1,3-dioxolan-4-yl)-2,2-dimethyltetrahydrofuro[2,3-*d*][1,3]dioxol-6-yl 4-((*E*)-3-phenoxyprop-1-en-1-yl)benzoate (67)

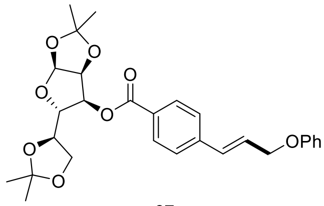

67

According to general procedure starting from 2-phenoxyacetic acid (0.1 mmol, 1.0 equiv), (3a*S*,5*S*,6*S*,6a*S*)-5-((*R*)-2,2-dimethyl-1,3-dioxolan-4-yl)-2,2-dimethyltetrahydrofuro[2,3-*d*][1,3]dioxol-6-yl 4-vinylbenzoate (0.5 mmol, 5.0 equiv), Et_3N (0.1 mmol, 1.0 equiv), $dmgH_2$ (5 mol%) and $Co_{1\%}$-$K_{11\%}$-PHI (2 mg, 0.34 mol% Co) in toluene (2 mL) for 48 h, the product **67** was isolated as colorless oil after flash chromatography (petroleum ether/ethyl acetate 20/1), 37.5 mg (75% yield). **^{1}H NMR** (400 MHz, $CDCl_3$) δ 7.98 (d, *J* = 8.5 Hz, 2H), 7.48 (d, *J* = 8.3 Hz, 2H), 7.36 – 7.23 (m, 2H), 7.04 – 6.90 (m, 3H), 6.79 (d, *J* = 16.0 Hz, 1H), 6.55 (dt, *J* = 16.0, 5.4 Hz, 1H), 5.96 (d, *J* = 3.7 Hz, 1H), 5.50 (d, *J* = 2.6 Hz, 1H), 4.76 – 4.69 (m, 2H), 4.63 (d, *J* = 3.7 Hz, 1H), 4.41 – 4.28 (m, 2H), 4.16 – 4.05 (m, 2H), 1.56 (s, 3H), 1.42 (s, 3H), 1.32 (s, 3H), 1.27 (s, 3H). **^{13}C NMR** (101 MHz, $CDCl_3$) δ 164.9, 158.5, 141.6, 131.4, 130.1, 129.6, 128.6, 127.9, 126.6, 121.1, 114.7, 112.4, 109.4, 105.2, 83.4, 80.0, 76.6, 72.6, 68.1, 67.3, 26.8, 26.8, 26.2, 25.2. **HRMS (ESI)** m/z calcd. For $C_{28}H_{32}NaO_8$ $[M+Na]^+$ 519.1989, found 519.2008.

((3a*S*)-2,2,7,7-tetramethyltetrahydro-3a*H*-bis([1,3]dioxolo)[4,5-*b*:4',5'-*d*]pyran-3a-yl)methyl 4-((*E*)-3-((*tert*-butoxycarbonyl)(methyl)amino)prop-1-en-1-yl) benzoate (68)

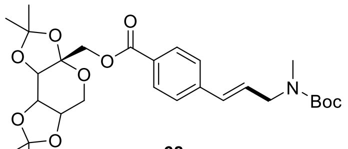

68

According to general procedure starting from *N*-Boc-*N*-methylglycine (0.1 mmol, 1.0 equiv), ((3aS)-2,2,7,7-tetramethyltetrahydro-3a*H*-bis([1,3] dioxolo) [4,5-*b*:4',5'-*d*]pyran-3a-yl)methyl 4-vinylbenzoate (0.5 mmol, 5.0 equiv), Et_3N (0.1 mmol, 1.0 equiv), $dmgH_2$ (5 mol%) and $Co_{1\%}$-$K_{11\%}$-PHI (2 mg, 0.34 mol% Co) in toluene (2 mL) for 48 h, the product **68** was isolated as colorless oil after flash chromatography (petroleum ether/ethyl acetate 8/1), 42 mg (79% yield). **^{1}H NMR** (400 MHz, $CDCl_3$) δ 8.02 (d, *J* = 8.1 Hz, 2H), 7.42 (d, *J* = 8.0 Hz, 2H), 6.49 (d, *J* = 15.8 Hz, 1H), 6.28 (dt, *J* = 15.7, 6.5 Hz, 1H), 4.70 – 4.61 (m, 2H), 4.47 (d, *J* = 2.6 Hz, 1H), 4.32 (d, *J* = 11.8 Hz, 1H), 4.27 (d, *J* = 8.6 Hz, 1H), 4.10 – 3.92 (m, 3H), 3.80 (d, *J* = 13.0 Hz, 1H), 2.88 (s, 3H), 1.57 – 1.44 (m, 15H), 1.36 (d, *J* = 8.1 Hz, 6H). **^{13}C NMR** (101 MHz, $CDCl_3$) δ 163.8, 153.8, 139.6, 128.7, 128.3, 126.9, 126.5, 124.4, 107.3, 107.0, 99.8, 77.8, 68.9, 68.7, 68.3, 63.3, 59.5, 48.6, 32.1, 26.6, 24.7, 24.0, 23.7, 22.2. **HRMS (ESI)** m/z calcd. For $C_{28}H_{40}NO_9$ $[M+H]^+$ 534.2698, found 534.2707.

((3a*S)*-2,2,7,7-tetramethyltetrahydro-3a*H*-bis([1,3]dioxolo)[4,5-*b*:4',5'-*d*]pyran-3a-yl)methyl 4-((*E*)-3-phenoxyprop-1-en-1-yl)benzoate (69)

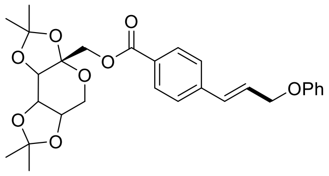

69

According to general procedure starting from 2-phenoxyacetic acid (0.1 mmol, 1.0 equiv), ((3aS)-2,2,7,7-tetramethyltetrahydro-3a*H*-bis([1,3]dioxolo)[4,5-*b*:4',5'-*d*]pyran-3a-yl)methyl 4-vinylbenzoate (0.5 mmol, 5.0 equiv), Et_3N (0.1 mmol, 1.0 equiv), $dmgH_2$ (5 mol%) and $Co_{1\%}$-$K_{11\%}$-PHI (2 mg, 0.34 mol% Co) in toluene (2 mL) for 48 h, the product **69** was isolated as colorless oil after flash chromatography (petroleum ether/ethyl acetate 10/1), 40 mg (80% yield). **^{1}H NMR** (400 MHz, $CDCl_3$) δ 8.03 (d, *J* = 8.4 Hz, 2H), 7.46 (d, *J* = 8.4 Hz, 2H), 7.35 – 7.25 (m, 2H), 7.03 – 6.89 (m, 3H), 6.77 (d, *J* = 16.0 Hz, 1H), 6.53 (dt, *J* = 16.0, 5.4 Hz, 1H), 4.75 – 4.67 (m, 3H), 4.64 (dd, *J* = 7.9, 2.6 Hz, 1H), 4.47 (d, *J* = 2.6 Hz, 1H), 4.33 (d, *J* = 11.8 Hz, 1H), 4.26 (dd, *J* = 7.9, 1.7 Hz, 1H), 3.96 (dd, *J* = 13.0, 1.9 Hz, 1H), 3.80 (d, *J* = 13.0 Hz, 1H), 1.55 (s, 3H), 1.47 (s, 3H), 1.36 (d, *J* = 8.9 Hz, 6H). **^{13}C NMR** (101 MHz, $CDCl_3$) δ 165.7, 158.5, 141.1, 131.5, 130.2, 129.6, 129.0, 127.6, 126.5, 121.1, 114.7, 109.2, 108.9, 101.7, 70.8, 70.6, 70.1, 68.1, 65.3, 61.4, 26.6, 25.9, 25.5, 24.1. **HRMS (ESI)** m/z calcd. For $C_{28}H_{33}O_8$ $[M+H]^+$ 497.2170, found 497.2179.

(3*R*,5*S*,8*S*,10*R*,13*R*,14*R*,17*R*)-17-acetyl-10,13-dimethyl-2,3,4,5,8,9,10,11,12,13,14,15,16,17-tetradecahydro-1*H*-cyclopenta[*a*]phenanthren-3-yl 4-((*E*)-3- ((*tert*-butoxycarbonyl)(methyl)amino)prop-1-en-1-yl)benzoate (70)

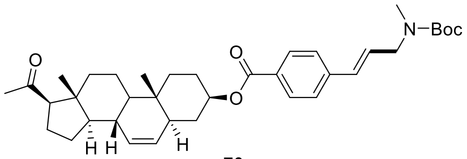

70

According to general procedure starting from *N*-Boc-*N*-methylglycine (0.1 mmol, 1.0 equiv), (3*R*,5*S*,8*S*,10*R*,13*R*,14*R*,17*R*)-17-acetyl-10,13-dimethyl-2,3,4,5,8,9,10,11,12,13,14,15,16,17-tetradecahydro-1*H*-cyclopenta[*a*]phenanthren-3-yl 4-vinylbenzoate (0.5 mmol, 5.0 equiv), Et_3N (0.1 mmol, 1.0 equiv), $dmgH_2$ (5 mol%) and $Co_{1\%}$-$K_{11\%}$-PHI (2 mg, 0.34 mol% Co) in toluene (2 mL) for 48 h, the product **70** was isolated as white solid after flash chromatography (petroleum ether/ethyl acetate 10/1), 36.5 mg (62% yield). mp = 128–130 ºC. **^{1}H NMR** (400 MHz, $CDCl_3$) δ 7.98 (d, *J* = 8.1 Hz, 2H), 7.41 (d, *J* = 8.1 Hz, 2H), 6.49 (d, *J* = 15.9 Hz, 1H), 6.27 (dd, *J* = 14.1, 7.6 Hz, 1H), 5.53 – 5.33 (m, 1H), 4.90 – 4.83 (m, 1H), 4.01 (s, 2H), 2.88 (s, 3H), 2.55 (t, *J* = 8.9 Hz, 1H), 2.47 (d, *J* = 8.0 Hz, 2H), 2.25 – 2.15 (m, 1H), 2.13 (s, 3H), 2.10 – 1.97 (m, 3H), 1.93 (dt, *J* = 13.3, 3.5 Hz, 1H), 1.81 – 1.53 (m, 8H), 1.48 (s, 9H), 1.32 – 1.14 (m, 4H), 1.07 (s, 3H), 0.65 (s, 3H). **^{13}C NMR** (101 MHz, $CDCl_3$) δ 209.6, 165.8, 155.7, 141.1, 130.1, 139.7, 129.9, 129.7, 129.6, 128.3, 128.1, 126.2, 122.5, 79.7, 74.4, 63.7, 56.8, 50.6, 49.9, 44.0, 38.8, 38.2, 37.1, 36.7, 34.0, 31.8, 31.8, 31.6, 28.5, 27.9, 24.5, 22.8, 21.1, 19.4, 13.2. **HRMS (ESI)** m/z calcd. For $C_{37}H_{51}NNaO_5$ $[M+Na]^+$ 612.3659, found 612.3672.

(3*R*,5*S*,8*S*,10*R*,13*R*,14*R*,17*R*)-17-acetyl-10,13-dimethyl-2,3,4,5,8,9,10,11,12,13,14,15,16,17-tetradecahydro-1*H*-cyclopenta[*a*]phenanthren-3-yl 4-((*E*)-3-phenoxyprop-1-en-1-yl)benzoate (71)

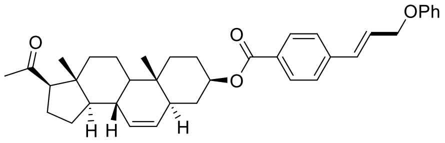

71

According to general procedure starting from 2-phenoxyacetic acid (0.1 mmol, 1.0 equiv), (3*R*,5*S*,8*S*,10*R*,13*R*,14*R*,17*R*)-17-acetyl-10,13-dimethyl-2,3,4,5,8,9,10,11,12,13,14,15,16,17-tetradecahydro-1*H*-cyclopenta[*a*]phenanthren-3-yl 4-vinylbenzoate (0.5 mmol, 5.0 equiv), Et_3N (0.1 mmol, 1.0 equiv), $dmgH_2$ (5 mol%) and $Co_{1\%}$-$K_{11\%}$-PHI (2 mg, 0.34 mol% Co) in toluene (2 mL) for 48 h, the product **71** was isolated as white solid after flash chromatography (petroleum ether/ethyl acetate 10/1), 33 mg (60% yield). mp = 146 – 148 ºC. **^{1}H NMR** (400 MHz, $CDCl_3$) δ 7.99 (d, *J* = 8.4 Hz, 2H), 7.46 (d, *J* = 8.3 Hz, 2H), 7.41 – 7.20 (m, 2H), 7.04 – 6.90 (m, 3H), 6.78 (d, *J* = 16.1 Hz, 1H), 6.53 (dt, *J* = 16.0, 5.5 Hz, 1H), 5.42 (d, *J* = 3.1 Hz, 1H), 4.93 – 4.78 (m, 1H), 4.72 (dd, *J* = 5.5, 1.7 Hz, 2H), 2.54 (t, *J* = 8.9 Hz, 1H), 2.47 (d, *J* = 7.6 Hz, 2H), 2.23 – 2.15 (m, 1H), 2.13 (s, 3H), 2.10 – 1.97 (m, 3H), 1.95 – 1.89 (m, 1H), 1.81 – 1.59 (m, 6H), 1.58 – 1.44 (m, 2H), 1.32 – 1.13 (m, 4H), 1.07 (s, 3H), 0.64 (s, 3H). **^{13}C NMR** (101 MHz, $CDCl_3$) δ 209.6, 165.7, 158.5, 140.8, 139.7, 131.7, 129.9, 129.6, 127.2, 126.4, 122.5, 121.1, 114.7, 74.5, 68.2, 63.7, 56.9, 49.9, 44.0, 38.8, 38.2, 37.1, 36.7, 31.9, 31.8, 31.6, 27.9, 24.5, 22.8, 21.1, 19.4, 13.2. **HRMS (ESI)** m/z calcd. For $C_{37}H_{44}NaO_4$ $[M+Na]^+$ 575.3132, found 575.3141.

isopropyl (*E*)-2-(4-(4-(3-((*tert*-butoxycarbonyl)(methyl)amino)prop-1-en-1-yl) benzoyl)phenoxy)-2-methylpropanoate (72)

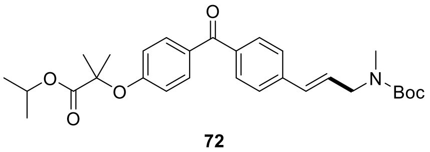

72

According to general procedure starting from *N*-Boc-*N*-methylglycine (0.1 mmol, 1.0 equiv), isopropyl 2-methyl-2-(4-(4-vinylbenzoyl)phenoxy)propanoate (0.5 mmol, 5.0 equiv), Et_3N (0.1 mmol, 1.0 equiv), $dmgH_2$ (5 mol%) and $Co_{1\%}$-$K_{11\%}$-PHI (2 mg, 0.34 mol% Co) in toluene (2 mL) for 48 h, the product **72** was isolated as colorless oil after flash chromatography (petroleum ether/ethyl acetate 10/1), 30.2 mg (61% yield). **^{1}H NMR** (400 MHz, $CDCl_3$) δ 7.73 (t, *J* = 9.2 Hz, 4H), 7.45 (d, *J* = 7.9 Hz, 2H), 6.87 (dd, *J* = 9.1, 2.7 Hz, 2H), 6.51 (d, *J* = 15.9 Hz, 1H), 6.29 (d, *J* = 16.1 Hz, 1H), 5.15 – 5.00 (m, 1H), 4.02 (s, 2H), 2.89 (s, 3H), 1.66 (s, 6H), 1.49 (s, 9H), 1.21 (d, *J* = 6.2 Hz, 6H). **^{13}C NMR** (101 MHz, $CDCl_3$) δ 195.0, 173.2, 159.5, 146.0, 140.4, 137.0, 131.9, 130.7, 130.3, 128.6, 126.1, 117.2, 79.7, 79.4, 69.3, 58.9, 34.0, 28.5, 25.4, 21.5. **HRMS (ESI)** m/z calcd. For $C_{29}H_{38}NO_6$ $[M+H]^+$ 496.2694, found 496.2691.

isopropyl (*E*)-2-methyl-2-(4-(4-(3-phenoxyprop-1-en-1-yl)benzoyl)phenoxy) propanoate (73)

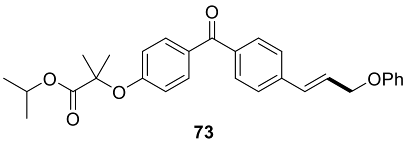

73

According to general procedure starting from 2-phenoxyacetic acid (0.1 mmol, 1.0 equiv), isopropyl 2-methyl-2-(4-(4-vinylbenzoyl)phenoxy)propanoate (0.5 mmol, 5.0 equiv), Et_3N (0.1 mmol, 1.0 equiv), $dmgH_2$ (5 mol%) and $Co_{1\%}$-$K_{11\%}$-PHI (2 mg, 0.34 mol% Co) in toluene (2 mL) for 48 h, the product **73** was isolated as yellow oil after flash chromatography (petroleum ether/ethyl acetate 20/1), 29 mg (63% yield). **^{1}H NMR** (400 MHz, $CDCl_3$) δ 7.74 (t, *J* = 8.6 Hz, 4H), 7.49 (d, *J* = 8.3 Hz, 2H), 7.35 – 7.28 (m, 2H), 7.01 – 6.92 (m, 3H), 6.90 – 6.84 (m, 2H), 6.80 (d, *J* = 16.0 Hz, 1H), 6.54 (dt, *J* = 16.0, 5.5 Hz, 1H), 5.16 – 5.03 (m, 1H), 4.73 (dd, *J* = 5.6, 1.6 Hz, 2H), 1.66 (s, 6H), 1.20 (d, *J* = 6.3 Hz, 6H). **^{13}C NMR** (101 MHz, $CDCl_3$) δ 193.1, 171.3, 157.7, 156.6, 138.3, 135.4, 130.1, 129.8, 128.9, 128.4, 127.7, 125.4, 124.4, 119.2, 115.4, 112.9, 77.5, 67.5, 66.4, 23.5, 19.7. **HRMS (ESI)** m/z calcd. For $C_{29}H_{31}O_5$ $[M+H]^+$ 459.2170, found 459.2179.

(*E*)-4-(3-((*tert*-butoxycarbonyl)(methyl)amino)prop-1-en-1-yl)phenyl (*R*)-2-(6-methoxynaphthalen-2-yl)propanoate (74)

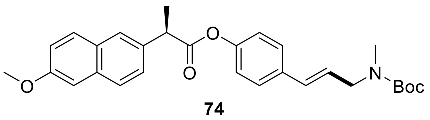

74

According to general procedure starting from *N*-Boc-*N*-methylglycine (0.1 mmol, 1.0 equiv), 4-vinylphenyl(*R*)-2-(6- methoxynaphthalen-2-yl)propanoate (0.5 mmol, 5.0 equiv), Et_3N (0.1 mmol, 1.0 equiv), $dmgH_2$ (5 mol%) and $Co_{1\%}$-$K_{11\%}$-PHI (2 mg, 0.34 mol% Co) in toluene (2 mL) for 48 h, the product **74** was isolated as white solid after flash chromatography (petroleum ether/ethyl acetate 20/1), 25.5 mg (54% yield). mp = 101–103 ºC. **^{1}H NMR** (400 MHz, $CDCl_3$) δ 7.83 – 7.67 (m, 3H), 7.49 (dd, *J* = 8.4, 1.9 Hz, 1H), 7.30 (d, *J* = 8.6 Hz, 2H), 7.21 – 7.10 (m, 2H), 6.93 (d, *J* = 8.6 Hz, 2H), 6.40 (d, *J* = 15.8 Hz, 1H), 6.07 (dd, *J* = 14.0, 7.5 Hz, 1H), 4.08 (q, *J* = 7.1 Hz, 1H), 4.00 – 3.87

(m, 5H), 2.84 (s, 3H), 1.68 (d, J = 7.2 Hz, 3H), 1.46 (s, 9H). **^{13}C NMR** (101 MHz, $CDCl_3$) δ 173.2, 157.8, 155.7, 150.2, 135.1, 134.5, 133.8, 131.0, 129.3, 129.0, 127.4, 127.2, 126.2, 126.1, 125.5, 121.5, 119.1, 105.6, 79.6, 55.3, 50.6, 45.6, 33.8, 28.5, 18.5. **HRMS (ESI)** m/z calcd. For $C_{29}H_{33}NNaO_5$ $[M+Na]^+$ 498.2251, found 498.2254.

(*E*)-4-(3-phenoxyprop-1-en-1-yl)phenyl (*R*)-2-(6-methoxynaphthalen-2-yl)propanoate (75)

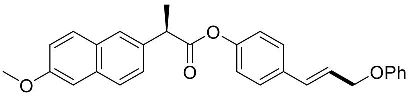

75

According to general procedure starting from 2-phenoxyacetic acid (0.1 mmol, 1.0 equiv), 4-vinylphenyl (*R*)-2-(6-methoxynaphthalen-2-yl)propanoate (0.5 mmol, 5.0 equiv), Et_3N (0.1 mmol, 1.0 equiv), $dmgH_2$ (5 mol%) and $Co_{1\%}$-$K_{11\%}$-PHI (2 mg, 0.34 mol% Co) in toluene (2 mL) for 48 h, the product **75** was isolated as white solid after flash chromatography (petroleum ether/ethyl acetate 30/1), 23.4 mg (53% yield). mp = 114 – 116 °C. **^{1}H NMR** (400 MHz, $CDCl_3$) δ 7.78 – 7.68 (m, 3H), 7.49 (dd, J = 8.5, 1.9 Hz, 1H), 7.34 (d, J = 8.6 Hz, 2H), 7.32 – 7.24 (m, 2H), 7.21 – 7.11 (m, 2H), 7.00 – 6.90 (m, 5H), 6.72 – 6.62 (m, 1H), 6.33 (dt, J = 16.0, 5.7 Hz, 1H), 4.66 (dd, J = 5.7, 1.5 Hz, 2H), 4.08 (q, J = 7.1 Hz, 1H), 3.91 (s, 3H), 1.68 (d, J = 7.1 Hz, 3H). **^{13}C NMR** (101 MHz, $CDCl_3$) δ 173.2, 158.6, 157.8, 150.5, 135.1, 134.2, 133.9, 131.9, 129.5, 129.4, 129.0, 127.5, 127.4, 126.2, 126.1, 124.7, 121.6, 121.0, 119.2, 114.8, 105.7, 68.5, 55.4, 45.6, 18.5. **HRMS (ESI)** m/z calcd. For $C_{29}H_{27}O_4$ $[M+H]^+$ 439.1904, found 439.1914.

4-((*E*)-3-((*tert*-butoxycarbonyl)(methyl)amino)prop-1-en-1-yl)phenyl (1*R*)-4,7,7-trimethyl-3-oxo-2-oxabicyclo[2.2.1]heptane-1-carboxylate (76)

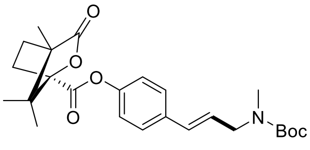

76

According to general procedure starting from *N*-Boc-*N*-methylglycine (0.1 mmol, 1.0 equiv), 4-vinylphenyl (1*R*)-4,7,7-trimethyl-3-oxo- 2-oxabicyclo[2.2.1]heptane-1-carboxylate (0.5 mmol, 5.0 equiv), Et_3N (0.1 mmol, 1.0 equiv), $dmgH_2$ (5 mol%) and $Co_{1\%}$-$K_{11\%}$-PHI (2 mg, 0.34 mol% Co) in toluene (2 mL) for 48 h, the product **76** was isolated as white solid after flash chromatography (petroleum ether/ethyl acetate 2/1), 30 mg (68% yield). mp = 83–85 °C. **^{1}H NMR** (400 MHz, $CDCl_3$) δ 7.39 (d, J = 8.7 Hz, 2H), 7.08 (d, J = 8.6 Hz, 2H), 6.45 (d, J = 15.9 Hz, 1H), 6.25 – 6.02 (m, 1H), 4.02 – 3.85 (m, 2H), 2.87 (s, 3H), 2.60 – 2.53 (m, 1H), 2.24 – 2.15 (m, 1H), 2.02 – 1.94 (m, 1H), 1.80 – 1.73 (m, 1H), 1.48 (s, 9H), 1.16 (d, J = 8.2 Hz, 6H), 1.11 (s, 3H). **^{13}C NMR** (101 MHz, $CDCl_3$) δ 177.8, 166.1, 155.7, 149.3, 135.2, 130.6, 127.4, 126.1, 121.4, 90.8, 79.6, 54.9, 54.7, 50.8, 33.9, 30.8, 29.0, 28.5, 16.9, 16.9, 9.7. **HRMS (ESI)** m/z calcd. For $C_{25}H_{33}NNaO_6$ $[M+Na]^+$ 466.2200, found 466.2205.

4-((*E*)-3-phenoxyprop-1-en-1-yl)phenyl (1*R*)-4,7,7-trimethyl-3-oxo-2-oxabicyclo[2.2.1]heptane-1-carboxylate (77)

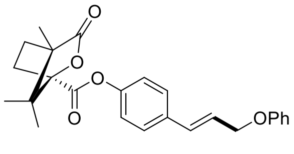

77

According to general procedure starting from 2-phenoxyacetic acid (0.1 mmol, 1.0 equiv), 4-vinylphenyl (1*R*)-4,7,7-trimethyl-3-oxo-2-oxabicyclo[2.2.1]heptane-1-carboxylate (0.5 mmol, 5.0 equiv), Et_3N (0.1 mmol, 1.0 equiv), $dmgH_2$ (5 mol%) and $Co_{1\%}$-$K_{11\%}$-PHI (2 mg, 0.34 mol% Co) in toluene (2 mL) for 48 h, the product **77** was isolated as white solid after flash chromatography (petroleum ether/ethyl acetate 10/1), 30.5 mg (75% yield). mp = 113 – 115 °C. **^{1}H NMR** (400 MHz, $CDCl_3$) δ 7.44 (d, *J* = 8.7 Hz, 2H), 7.30 (dd, *J* = 8.7, 7.3 Hz, 2H), 7.10 (d, *J* = 8.6 Hz, 2H), 7.04 – 6.90 (m, 3H), 6.73 (d, *J* = 16.1 Hz, 1H), 6.40 (dt, *J* = 16.0, 5.7 Hz, 1H), 4.70 (dd, *J* = 5.8, 1.6 Hz, 2H), 2.61 – 2.50 (m, 1H), 2.24 – 2.16 (m, 1H), 2.03 – 1.95 (m, 1H), 1.80 – 1.73 (m, 1H), 1.16 (d, *J* = 7.8 Hz, 6H), 1.11 (s, 3H). **^{13}C NMR** (101 MHz, $CDCl_3$) δ 177.9, 166.1, 158.6, 149.5, 134.9, 131.6, 129.5, 127.7, 125.3, 121.5, 121.0, 114.8, 90.9, 68.4, 54.9, 54.7, 30.8, 29.0, 16.9, 16.9, 9.8. **HRMS (ESI)** m/z calcd. For $C_{25}H_{27}O_5$ $[M+H]^+$ 407.1853, found 407.1849.

(*E*)-4-(3-((*tert*-butoxycarbonyl)(methyl)amino)prop-1-en-1-yl)benzyl 4-(*N*,*N*-dipropylsulfamoyl)benzoate (78)

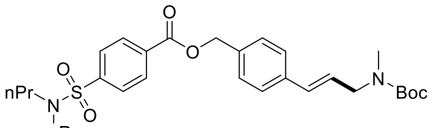

78

According to general procedure starting from *N*-Boc-*N*-methylglycine (0.1 mmol, 1.0 equiv), 4-vinylbenzyl 4-(*N*,*N*-dipropylsulfamoyl) benzoate (0.5 mmol, 5.0 equiv), Et_3N (0.1 mmol, 1.0 equiv), $dmgH_2$ (5 mol%) and $Co_{1\%}$-$K_{11\%}$-PHI (2 mg, 0.34 mol% Co) in toluene (2 mL) for 48 h, the product **78** was isolated as yellow oil after flash chromatography (petroleum ether/ethyl acetate 10/1), 39.5 mg (72% yield). **^{1}H NMR** (400 MHz, $CDCl_3$) δ 8.18 (d, *J* = 8.5 Hz, 2H), 7.87 (d, *J* = 8.5 Hz, 2H), 7.40 (s, 4H), 6.47 (d, *J* = 15.9 Hz, 1H), 6.18 (dt, *J* = 15.8, 6.1 Hz, 1H), 5.37 (s, 2H), 3.99 (s, 2H), 3.15 – 3.02 (m, 4H), 2.87 (s, 3H), 1.59 – 1.51 (m, 4H), 1.48 (s, 9H), 0.86 (t, *J* = 7.4 Hz, 6H). **^{13}C NMR** (101 MHz, $CDCl_3$) δ 165.1, 155.7, 144.4, 137.1, 134.7, 133.4, 130.8, 130.3, 128.8, 127.0, 126.6, 126.1, 79.6, 67.1, 51.6, 49.9, 33.8, 28.5, 21.9, 11.1. **HRMS (ESI)** m/z calcd. For $C_{29}H_{40}N_2NaO_6S$ $[M+Na]^+$ 567.2499, found 567.2501.

(*E*)-4-(3-phenoxyprop-1-en-1-yl)benzyl 4-(*N*,*N*-dipropylsulfamoyl)benzoate (79)

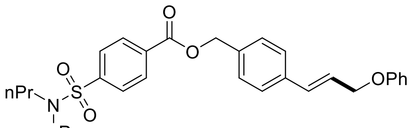

79

According to general procedure starting from 2-phenoxyacetic acid (0.1 mmol, 1.0 equiv), 4-vinylbenzyl 4-(*N*,*N*-dipropylsulfamoyl)benzoate (0.5 mmol, 5.0 equiv), Et_3N (0.1 mmol, 1.0 equiv), $dmgH_2$ (5 mol%) and $Co_{1\%}$-$K_{11\%}$-PHI (2 mg, 0.34 mol% Co) in toluene (2 mL) for 48 h, the product **79** was isolated as white solid after flash chromatography (petroleum ether/ethyl acetate 20/1), 33.5 mg (66% yield). mp = 56 – 58 °C. **^{1}H NMR** (400 MHz, $CDCl_3$) δ 8.17 (d, *J* = 8.5 Hz, 2H), 7.87 (d, *J* = 8.5 Hz, 2H), 7.47 – 7.35 (m, 4H), 7.36 – 7.22 (m, 2H), 7.01 – 6.86 (m, 3H), 6.74 (d, *J* = 16.0 Hz, 1H), 6.44 (dt, *J* = 16.0, 5.7 Hz, 1H), 5.36 (s, 2H), 4.70 (dd, *J* = 5.7, 1.6 Hz, 2H), 3.13 – 3.05 (m, 4H), 1.59 – 1.48 (m, *J* = 7.4 Hz, 4H), 0.86 (t, *J* = 7.4 Hz, 6H). **^{13}C NMR**

(101 MHz, $CDCl_3$) δ 165.1, 158.6, 144.4, 136.9, 135.0, 133.4, 132.2, 130.4, 129.5, 128.8, 127.0, 126.9, 125.4, 121.0, 114.8, 68.4, 67.1, 49.9, 21.9, 11.2. **HRMS (ESI)** m/z calcd. For $C_{29}H_{34}NO_5S$ $[M+H]^+$ 508.2152, found 508.2163.

(*E*)-4-(3-((*tert*-butoxycarbonyl)(methyl)amino)prop-1-en-1-yl)benzyl 2-(4-chlorophenoxy)-2-methylpropanoate (80)

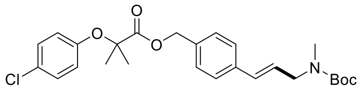

80

According to general procedure starting from *N*-Boc-*N*-methylglycine (0.1 mmol, 1.0 equiv), 4-vinylbenzyl 2-(4-chlorophenoxy)-2-methylpropanoate (0.5 mmol, 5.0 equiv), Et_3N (0.1 mmol, 1.0 equiv), $dmgH_2$ (5 mol%) and $Co_{1\%}$-$K_{11\%}$-PHI (2 mg, 0.34 mol% Co) in toluene (2 mL) for 48 h, the product **80** was isolated as colorless oil after flash chromatography (petroleum ether/ethyl acetate 20/1), 36.5 mg (77% yield). **^{1}H NMR** (400 MHz, $CDCl_3$) δ 7.32 (d, *J* = 8.2 Hz, 2H), 7.22 (d, *J* = 8.1 Hz, 2H), 7.14 – 7.02 (m, 2H), 6.72 – 6.63 (m, 2H), 6.45 (d, *J* = 15.8 Hz, 1H), 6.17 (dd, *J* = 14.2, 7.6 Hz, 1H), 5.16 (s, 2H), 3.98 (s, 2H), 2.87 (s, 3H), 1.58 (s, 6H), 1.48 (s, 9H). **^{13}C NMR** (101 MHz, $CDCl_3$) δ 173.7, 155.7, 154.0, 137.0, 134.4, 130.9, 129.1, 128.9, 127.2, 126.5, 126.1, 120.4, 79.6, 79.5, 66.9, 50.7, 33.8, 28.5, 25.3. **HRMS (ESI)** m/z calcd. For $C_{26}H_{33}ClNO_5$ $[M+H]^+$ 474.2042, found 474.2065.

(*E*)-4-(3-phenoxyprop-1-en-1-yl)benzyl 2-(4-chlorophenoxy)-2-methylpropanoate (81)

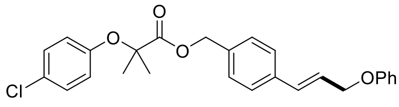

81

According to general procedure starting from 2-phenoxyacetic acid (0.1 mmol, 1.0 equiv), 4-vinylbenzyl 2-(4-chlorophenoxy)-2-methylpropanoate (0.5 mmol, 5.0 equiv), Et_3N (0.1 mmol, 1.0 equiv), $dmgH_2$ (5 mol%) and $Co_{1\%}$-$K_{11\%}$-PHI (2 mg, 0.34 mol% Co) in toluene (2 mL) for 48 h, the product **81** was isolated as white solid after flash chromatography (petroleum ether/ethyl acetate 30/1), 28.4 mg (65% yield). mp = 62 – 64 °C. **^{1}H NMR** (400 MHz, $CDCl_3$) δ 7.35 (d, *J* = 8.1 Hz, 2H), 7.29 (t, *J* = 7.9 Hz, 2H), 7.21 (d, *J* = 7.9 Hz, 2H), 7.11 – 7.06 (m, 2H), 6.99 – 6.91 (m, 3H), 6.76 – 6.65 (m, 3H), 6.42 (dt, *J* = 15.9, 5.6 Hz, 1H), 5.16 (s, 2H), 4.69 (dd, *J* = 5.6, 1.6 Hz, 2H), 1.57 (s, 6H). **^{13}C NMR** (101 MHz, $CDCl_3$) δ 173.7, 158.6, 154.0, 136.8, 134.8, 132.2, 129.6, 129.1, 128.8, 127.2, 126.7, 125.4, 121.0, 120.5, 114.8, 79.6, 68.4, 66.9, 25.4. **HRMS (ESI)** m/z calcd. For $C_{26}H_{25}ClNaO_4$ $[M+Na]^+$ 459.1334, found 459.1333.

(*E*)-4-(3-((*tert*-butoxycarbonyl)(methyl)amino)prop-1-en-1-yl)benzyl 2-(4-isobutylphenyl)propanoate (82)

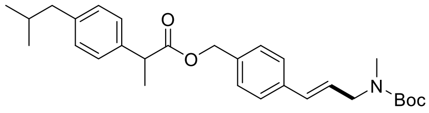

82

According to general procedure starting from *N*-Boc-*N*-methylglycine (0.1 mmol, 1.0 equiv), 4-vinylbenzyl 2-(4-isobutylphenyl) propanoate (0.5 mmol, 5.0 equiv), Et_3N (0.1 mmol, 1.0 equiv), $dmgH_2$ (5 mol%) and $Co_{1\%}$-$K_{11\%}$-PHI (2 mg, 0.34 mol% Co) in toluene (2 mL) for 48 h, the product **82** was isolated as colorless oil after flash chromatography (petroleum ether/ethyl acetate 20/1), 30.7 mg (66% yield). **^{1}H NMR** (400 MHz, $CDCl_3$) δ 7.29 (d, *J* = 8.1 Hz, 2H), 7.22 – 7.14 (m, 4H), 7.08 (d, *J* = 8.1 Hz, 2H), 6.43 (d, *J* = 15.9 Hz, 1H), 6.12 (dt, *J* = 14.4, 5.6 Hz, 1H), 5.12 – 5.03 (m, 2H), 3.97 (s, 2H), 3.74 (q, *J* = 7.2

Hz, 1H), 2.86 (s, 3H), 2.45 (d, J = 7.2 Hz, 2H), 1.89 – 1.79 (m, 1H), 1.53 – 1.43 (m, 12H), 0.90 (d, J = 6.7 Hz, 6H). **^{13}C NMR** (101 MHz, $CDCl_3$) δ 174.5, 155.8, 140.6, 137.6, 136.6, 135.4, 131.5, 129.3, 128.1, 127.2, 126.4, 125.7, 79.6, 66.0, 50.2, 45.2, 45.0, 33.8, 30.2, 28.5, 22.4, 18.4. **HRMS (ESI)** m/z calcd. For $C_{29}H_{39}NNaO_4$ $[M+Na]^+$ 488.2771, found 488.2772.

(*E*)-4-(3-phenoxyprop-1-en-1-yl)benzyl 2-(4-isobutylphenyl)propanoate (83)

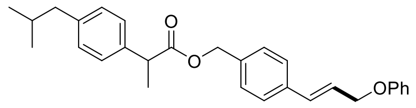

83

According to general procedure starting from 2-phenoxyacetic acid (0.1 mmol, 1.0 equiv), 4-vinylbenzyl 2-(4-isobutylphenyl)propanoate (0.5 mmol, 5.0 equiv), Et_3N (0.1 mmol, 1.0 equiv), $dmgH_2$ (5 mol%) and $Co_{1\%}$-$K_{11\%}$-PHI (2 mg, 0.34 mol% Co) in toluene (2 mL) for 48 h, the product **83** was isolated as white solid after flash chromatography (petroleum ether/ethyl acetate 20/1), 30.5 mg (71% yield). mp = 42 – 44 ºC. **^{1}H NMR** (400 MHz, $CDCl_3$) δ 7.35 – 7.21 (m, 4H), 7.23 – 7.12 (m, 4H), 7.08 (d, J = 7.8 Hz, 2H), 6.99 – 6.91 (m, 3H), 6.69 (d, J = 16.1 Hz, 1H), 6.39 (dt, J = 16.0, 5.7 Hz, 1H), 5.12 – 5.02 (m, 2H), 4.67 (dd, J = 5.7, 1.6 Hz, 2H), 3.73 (q, J = 7.1 Hz, 1H), 2.44 (d, J = 7.2 Hz, 2H), 1.91 – 1.75 (m, 1H), 1.50 (d, J = 7.2 Hz, 3H), 0.89 (d, J = 6.7 Hz, 6H). **^{13}C NMR** (101 MHz, $CDCl_3$) δ 174.5, 158.6, 140.6, 137.6, 136.3, 135.8, 132.4, 129.6, 129.4, 128.1, 127.3, 126.7, 125.0, 121.0, 114.8, 68.5, 66.0, 45.2, 45.1, 30.3, 22.4, 18.5. **HRMS (ESI)** m/z calcd. For $C_{29}H_{32}NaO_3$ $[M+Na]^+$ 451.2244, found 451.2235.

9 Synthetic Applications

9.1 Synthesis of Naftifine Hydrochloride

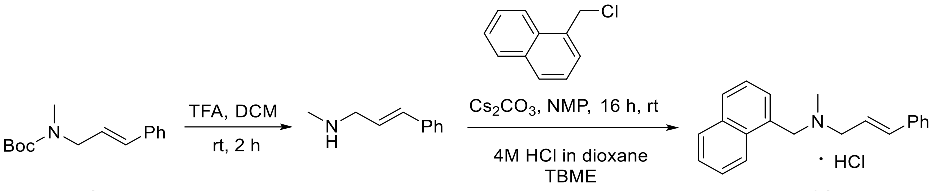

1 **84** **Naftifine hydrochloride**

To a stirred solution of *tert*-butyl cinnamyl(methyl)carbamate (1.0 mmol, 1.0 equiv) in DCM (0.5 M), trifluoroacetic acid (10 mmol, 10 equiv) was added. The reaction was stirred at room temperature for 2 h and monitored by TLC for completion. Then, the solvent was removed under reduced pressure, and the residue was dissolved in TBME (4 mL). The organic phase was extracted with 1 M HCl (3 × 4 mL). The combined aqueous phases were then adjusted to pH 11-12 with 2 M NaOH (aq) and extracted with DCM (3 × 4 mL). The combined organic layers were washed with brine. Subsequent drying over anhydrous Na_2SO_4 and evaporation of the organic solvent afforded (*E*)-*N*-methyl-3-phenylprop-2-en-1-amine (free base) in 94% yield.

(*E*)-*N*-methyl-3-phenylprop-2-en-1-amine (0.68 mmol, 1.0 equiv) was added to a 5 mL round-bottom flask equipped with a magnetic stirrer bar and dissolved in 0.8 mL NMP (0.85 M). Then, Cs_2CO_3 (1.36 mmol, 2.0 equiv) and 1-(chloromethyl)naphtha (1.02 mmol, 1.5 equiv) were added added, and the reaction was stirred at room temperature for 16 h, monitored by TLC. Upon completion, 8 mL of distilled water was added, and the aqueous phase was extracted with EtOAc (2 × 4 mL). The combined organic phases were washed with distilled water (2 × 4 mL), 5 wt% $NaHCO_3$ (4 mL), and brine (4 mL). The organic phase was dried over Na_2SO_4, and the solvent was removed under reduced pressure to afford a yellow oily residue. This residue was dissolved in 0.3 mL TBME, and 0.3 mL of 4 M HCl in dioxane was added dropwise to yield a white solid. The solid was centrifuged, washed with 1 mL TBME, and dried under vacuum at 40 °C for 16 h (**84**, 80% yield).

(*E*)-*N*-methyl-3-phenylprop-2-en-1-amine[29]

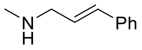

^{1}H NMR (400 MHz, $CDCl_3$) δ 7.91 (br, 1H), 7.44 – 7.20 (m, 5H), 6.68 (d, *J* = 15.8 Hz, 1H), 6.18 (dt, *J* = 15.3, 7.2 Hz, 1H), 3.62 (d, *J* = 7.3 Hz, 2H), 2.57 (s, 3H). **^{13}C NMR** (101 MHz, $CDCl_3$) δ 138.7, 135.2, 128.8, 128.8, 126.8, 118.0, 50.8, 31.7.

Naftifine hydrochloride[29] **(84)**

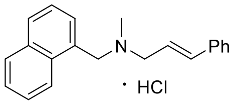

84

^{1}H NMR (400 MHz, $CDCl_3$) δ 8.17 – 8.03 (m, 2H), 8.01 – 7.86 (m, 2H), 7.68 – 7.53 (m, 3H), 7.45 – 7.40 (m, 2H), 7.38 – 7.30 (m, 3H), 6.70 (d, *J* = 15.9 Hz, 1H), 6.64 – 6.52 (m, 1H), 4.81 (dd, *J* = 13.6, 4.9 Hz, 1H), 4.65 (dd, *J* = 13.7, 5.9 Hz, 1H), 4.05 – 3.69 (m, 1H), 3.83 – 3.67 (m, 1H), 2.69 (d, *J* = 4.9 Hz, 3H). **^{13}C NMR** (101 MHz, $CDCl_3$) δ 140.3, 134.7, 134.0, 132.1, 131.7, 131.1, 129.5, 129.2, 128.8, 127.7, 127.1, 126.5, 125.8, 124.5,

122.5, 116.8, 58.2, 54.3, 38.8.

9.2 Synthesis the Key Intermediate of Reboxetine Mesylate

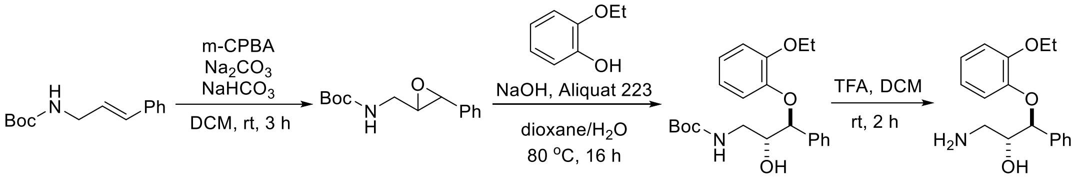

3 **85**

To a solution of *tert*-butyl cinnamylcarbamate (2.0 mmol, 1.0 equiv) in DCM (0.75 M) was added mCPBA (5.2 mmol, 2.6 equiv) followed by $NaHCO_3$ (4.7 mmol, 2.35 equiv) and Na_2CO_3 (3.7 mmol, 1.85 equiv). The reaction mixture was stirred at room temperature for 3 h. Aqueous $NaHCO_3$ (10%) was added and the product was extracted with DCM three times. Combined organic layers were washed with brine-$NaHCO_3$, dried over anhydrous Na_2SO_3 and concentrated under reduced pressure. Purification by flash chromatography (10% to 50% EtOAc-hexanes gradient) gave epoxide as a colorless oil in 80% yield which was used in the next step without further purification. (The product could not be fully purified and was used in its crude form in the next step without further purification).

tert-butyl ((3-phenyloxiran-2-yl)methyl)carbamate (1.5 mmol, 1.0 equiv) was added to a 25 mL round-bottom flask equipped with a magnetic stirrer bar and dissolved in dioxane (1.33 M). Tetrabutylammonium bromide (0.255 mmol, 0.17 equiv), NaOH (1.65 mmol, 1.1 equiv), H_2O (0.8 M), and 2-ethoxyphenol (2.25 mmol, 1.5 equiv) were added to the flask, and the reaction was heated at 80 °C for 16 h, monitored by TLC. The reaction was cooled to room temperature. 10 mL TBME was added to the mixture, and the organic phase was washed with 2 M KOH (2 × 5 mL), water (2 × 5 mL), and brine (1 × 5 mL). After drying over Na_2SO_4, the solvent was removed under reduced pressure to afford an orange oil in 91% yield.

To a stirred solution of *tert*-butyl ((2*R*,3*S*)-3-(2-ethoxyphenoxy)-2-hydroxy-3-phenylpropyl)carbamate (1.0 mmol, 1.0 equiv) in DCM (0.5 M), trifluoroacetic acid (10.0 mmol, 10.0 equiv) was added. The reaction was stirred at room temperature for 2 h and monitored by TLC for completion. Then, the solvent was removed under reduced pressure, and the residue was dissolved in TBME (4 mL). The organic phase was extracted with 1 M HCl (3 × 4 mL). The combined aqueous phases were then adjusted to pH 11-12 with 2 M NaOH (aq) and extracted with DCM (3 × 4 mL). The combined organic layers were washed with brine. Subsequent drying over anhydrous Na_2SO_4 and evaporation of the organic solvent afforded the free-base amine (**85**, 95% yield).

***tert*-butyl ((2*R*,3*S*)-3-(2-ethoxyphenoxy)-2-hydroxy-3-phenylpropyl)carbamate**[29]

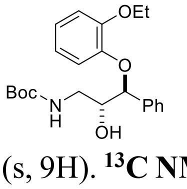

^{1}H NMR (400 MHz, $CDCl_3$) δ 7.40 (d, J = 6.8 Hz, 2H), 7.35 (t, J = 7.5 Hz, 2H), 7.31 – 7.27 (m, 1H), 6.96 – 6.85 (m, 2H), 6.79 – 6.61 (m, 2H), 5.39 (s, 1H), 5.07 (d, J = 4.7 Hz, 1H), 4.22 – 4.08 (m, 2H), 3.96 (s, 1H), 3.72 – 3.32 (m, 2H), 3.31 – 3.18 (m, 1H), 1.51 (t, J = 7.0 Hz, 3H), 1.43 (s, 9H). **^{13}C NMR** (101 MHz, $CDCl_3$) δ 149.7, 147.6, 138.0, 128.6, 128.1, 126.8, 122.9,

121.0, 118.2, 113.1, 85.6, 79.4, 73.8, 64.4, 42.4, 28.4, 14.9.

(1*S*,2*R*)-3-amino-1-(2-ethoxyphenoxy)-1-phenylpropan-2-ol[29] (85)

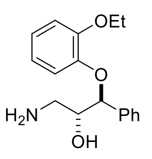

85

^{1}H NMR (400 MHz, $CDCl_3$) δ 7.39 (d, *J* = 7.1 Hz, 2H), 7.32 (t, *J* = 7.5 Hz, 2H), 7.30 – 7.21 (m, 1H), 6.91 – 6.81 (m, 2H), 6.74 – 6.62 (m, 2H), 5.10 (d, *J* = 4.4 Hz, 1H), 4.08 (q, *J* = 7.0 Hz, 2H), 3.82 (d, *J* = 5.1 Hz, 1H), 3.04 – 2.74 (m, 2H), 2.41 (br, 3H), 1.46 (t, *J* = 7.0 Hz, 3H). **^{13}C NMR** (101 MHz, $CDCl_3$) δ 149.7, 147.8, 138.6, 128.5, 127.9, 126.9, 122.4, 120.9, 117.6, 113.3, 85.3, 74.9, 64.4, 42.5, 14.9.

9.3 Synthesis the Key Intermediate of KN-93

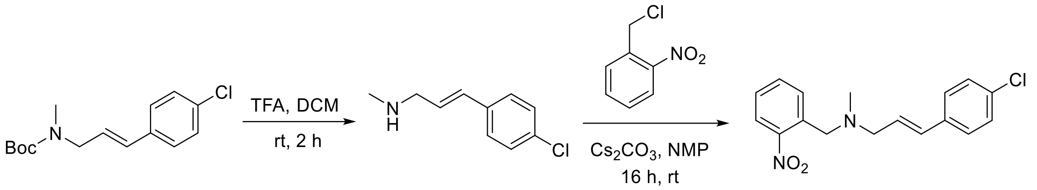

29 **86**

To a stirred solution of *tert*-butyl (*E*)-(3-(4-chlorophenyl)allyl)(methyl)carbamate (1.0 mmol, 1.0 equiv) in DCM (0.5 M), trifluoroacetic acid (10 mmol, 10.0 equiv) was added. The reaction was stirred at room temperature for 2 h and monitored by TLC for completion. Then, the solvent was removed under reduced pressure, and the residue was dissolved in TBME (4 mL). The organic phase was extracted with 1 M HCl (3 × 4 mL). The combined aqueous phases were then adjusted to pH 11-12 with 2 M NaOH (aq) and extracted with DCM (3 × 4 mL). The combined organic layers were washed with brine. Subsequent drying over anhydrous Na_2SO_4 and evaporation of the organic solvent afforded (*E*)-3-(4-chlorophenyl)-*N*-methylprop-2-en-1-amine (free base) in 96% yield.

(*E*)-3-(4-chlorophenyl)-*N*-methylprop-2-en-1-amine (0.68 mmol, 1.0 equiv) was added to a 5 mL round-bottom flask equipped with a magnetic stirrer bar and dissolved in 0.8 mL NMP (0.85 M). Then, Cs_2CO_3 (1.36 mmol, 2.0 equiv) and 1-(chloromethyl)-2-nitrobenzene (1.02 mmol, 1.5 equiv) were added, and the reaction was stirred at room temperature for 16 h, monitored by TLC. Upon completion, 8 mL of distilled water was added, and the aqueous phase was extracted with EtOAc (2 × 4 mL). The combined organic phases were washed with distilled water (2 × 4 mL), 5 wt% $NaHCO_3$ (4 mL), and brine (4 mL). The organic phase was dried over Na_2SO_4, and the solvent was removed under reduced pressure to afford a yellow oily residue. Finally, the crude residue was purified by silica gel column chromatography to afford the product **86** in 75% yield.

(*E*)-3-(4-chlorophenyl)-*N*-methylprop-2-en-1-amine

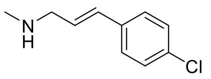

^{1}H NMR (400 MHz, $CDCl_3$) δ 7.34 – 7.09 (m, 4H), 6.59 – 6.41 (m, 1H), 6.29 – 6.14 (m, 1H), 3.33 (d, *J* = 6.3 Hz, 2H), 2.44 (s, 3H). **^{13}C NMR** (101 MHz, $CDCl_3$) δ 135.6, 132.9, 130.2, 128.7, 128.6, 127.4, 53.5, 35.8. **HRMS (ESI)** m/z calcd. For $C_{10}H_{13}ClN$ $[M+H]^+$ 182.0731, found 182.0730.

(*E*)-3-(4-chlorophenyl)-*N*-methyl-*N*-(2-nitrobenzyl)prop-2-en-1-amine[32] (86)

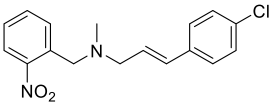

86

^{1}H NMR (400 MHz, $CDCl_3$) δ 7.81 (dd, *J* = 8.1, 1.3 Hz, 1H), 7.63 (dd, *J* = 7.8, 1.5 Hz, 1H), 7.53 (td, *J* = 7.6, 1.3 Hz, 1H), 7.38 (td, *J* = 7.7, 1.5 Hz, 1H), 7.33 – 7.23 (m, 4H), 6.46 (d, *J* = 15.9 Hz, 1H), 6.20 (dt, *J* = 15.9, 6.5 Hz, 1H), 3.82 (s, 2H), 3.16 (dd, *J* = 6.6, 1.4 Hz, 2H), 2.20 (s, 3H). **^{13}C NMR** (101 MHz, $CDCl_3$) δ 149.8, 135.5, 134.7, 133.0, 132.4, 131.2, 131.0, 128.7, 128.1, 127.9, 127.5, 124.4, 60.1, 58.2, 42.5.

9.4 Synthesis of Abamine

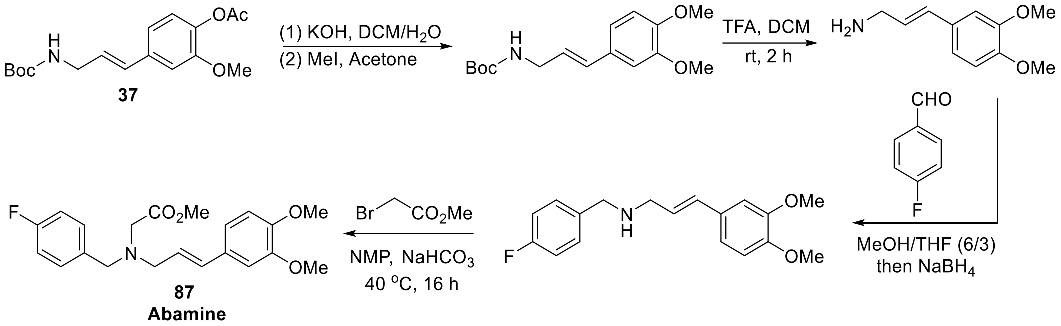

A stirred solution of (*E*)-4-(3-((*tert*-butoxycarbonyl)amino)prop-1-en-1-yl)-2-methoxyphenyl acetate (2.0 mmol, 1.0 equiv) in DCM/H_2O (4 mL, 10:1 v/v) was prepared, and potassium hydroxide (6.0 mmol, 3.0 equiv) was added to the mixture. The reaction mixture was stirred for 3 h. A solution of methyl iodide (3.0 mmol, 1.5 equiv) in acetone (0.75 M) was added dropwise over the period of an hour, and the temperature was kept constant at room temperature using a water bath. The resulting suspension was stirred at room temperature for 2 days, and the volatiles were removed in vacuo. The remaining aqueous phase was extracted four times with chloroform. The combined organic phases were filtered over cotton wool, and the solvent was removed in vacuo. The crude product was purified by column chromatography to afford the product in 92% yield.

To a stirred solution of *tert*-butyl *tert*-butyl (*E*)-2-(4-hydroxy-3-methoxystyryl) pyrrolidine-1-carboxylate (1.5 mmol, 1.0 equiv) in DCM (0.5 M), trifluoroacetic acid (15 mmol, 10.0 equiv) was added. The reaction was stirred at room temperature for 2 h and monitored by TLC for completion. Then, the solvent was removed under reduced pressure, and the residue was dissolved in TBME (4 mL). The organic phase was extracted with 1 M HCl (3 × 4 mL). The combined aqueous phases were then adjusted to pH 11-12 with 2 M NaOH (aq) and extracted with DCM (3 × 4 mL). The combined organic layers were washed with brine. Subsequent drying over anhydrous Na_2SO_4 and evaporation of the organic solvent afforded (*E*)-3-(3,4-dimethoxyphenyl)prop-2-en-1-amine (free base) in 95% yield.

(*E*)-3-(3,4-dimethoxyphenyl)prop-2-en-1-amine (1.0 mmol, 1.0 equiv) and p-fluorobenzaldehyde (0.95 mmol, 0.95 equiv) were added in a 5 mL roundbottom flask. MeOH (0.86 M) and THF (1.7 M) were added and the reaction was stirred for 16 h at room temperature. The reaction was cooled at 0 °C and $NaBH_4$ (3.0 mmol, 3.0 equiv) were added in the flask. The mixture was stirred for 2 h at 0 °C monitored by TLC.

Upon completion, the reaction mixture was concentrated under reduced pressure to evaporate the solvent, and the crude residue was purified by silica gel column chromatography to afford the product in 70% yield.

(*E*)-3-(3,4-dimethoxyphenyl)-*N*-(4-fluorobenzyl)prop-2-en-1-amine (0.5 mmol, 1.0 equiv) was added to a 5 mL round-bottom flask and dissolved in 0.45 mL of NMP (10 vol, 0.33 M). $NaHCO_3$ (1.5 mmol, 3.0 equiv) and methylbromoacetate (5.5 mmol, 1.1 equiv) were added, and the reaction was stirred for 16 h at room temperature, monitored by TLC. Upon completion, 0.5 mL of water was added. The aqueous phase was extracted with EtOAc (2 × 2 mL). The combined organic phases were washed with H_2O (2 × 2 mL), 5 wt% $NaHCO_3$ (2 × 2 mL), and brine (2 mL). The organic phase was dried over Na_2SO_4, and the solvent was removed under reduced pressure to afford a yellow oily residue. Finally, the crude residue was purified by silica gel column chromatography (**87**, 86%).

***tert*-butyl (E)-(3-(3,4-dimethoxyphenyl)allyl)carbamate**[29]

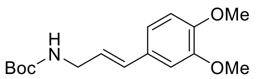

^{1}H NMR (400 MHz, $CDCl_3$) δ 6.92 – 6.84 (m, 2H), 6.79 (d, *J* = 8.2 Hz, 1H), 6.42 (d, *J* = 15.9 Hz, 1H), 6.05 (dt, *J* = 15.8, 6.2 Hz, 1H), 4.86 (s, 1H), 3.92 – 2.72 (m, 8H), 1.46 (s, 9H). **^{13}C NMR** (101 MHz, $CDCl_3$) δ 155.8, 149.0, 148.8, 131.2, 129.8, 124.4, 119.5, 111.1, 108.8, 79.3, 55.9, 55.8, 42.7, 28.4.

(*E*)-3-(3,4-dimethoxyphenyl)prop-2-en-1-amine[29]

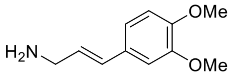

^{1}H NMR (400 MHz, $CDCl_3$) δ 6.95 – 6.86 (m, 2H), 6.81 (d, *J* = 8.2 Hz, 1H), 6.44 (d, *J* = 16.1 Hz, 1H), 6.19 (dt, *J* = 15.8, 6.0 Hz, 1H), 3.88 (d, *J* = 8.0 Hz, 6H), 3.46 (dd, *J* = 6.0, 1.5 Hz, 2H), 2.14 (s, 2H). **^{13}C NMR** (101 MHz, $CDCl_3$) δ 149.0, 148.6, 130.3, 129.5, 129.0, 119.3, 111.2, 108.7, 55.9, 55.8, 44.2.

(*E*)-3-(3,4-dimethoxyphenyl)-*N*-(4-fluorobenzyl)prop-2-en-1-amine[29]

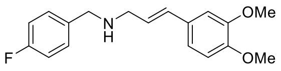

^{1}H NMR (400 MHz, $CDCl_3$) δ 7.33 – 7.27 (m, 2H), 7.05 – 6.96 (m, 2H), 6.94 (d, *J* = 2.0 Hz, 1H), 6.89 (dd, *J* = 8.2, 2.0 Hz, 1H), 6.81 (d, *J* = 8.2 Hz, 1H), 6.51 – 6.42 (m, 1H), 6.18 (dt, *J* = 15.8, 6.4 Hz, 1H), 3.88 (d, *J* = 6.1 Hz, 6H), 3.80 (s, 2H), 3.41 (dd, *J* = 6.4, 1.5 Hz, 2H). **^{13}C NMR** (101 MHz, $CDCl_3$) δ 161.95 (d, *J* = 244.6 Hz), 149.1, 148.7, 136.0 (d, *J* = 3.2 Hz), 131.3, 130.2, 129.7 (d, *J* = 7.9 Hz), 126.3, 119.4, 115.2 (d, *J* = 21.1 Hz), 111.1, 108.7, 55.9, 55.8, 52.6, 51.2. **^{19}F NMR** (377 MHz, $CDCl_3$) δ -116.0 (s).

Abamine[29] **(87)**

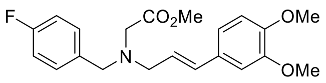

87

^{1}H NMR (400 MHz, $CDCl_3$) δ 7.36 – 7.31 (m, 2H), 7.04 – 6.97 (m, 2H), 6.95 – 6.87 (m, 2H), 6.81 (d, *J* = 8.2 Hz, 1H), 6.47 (d, *J* = 15.8 Hz, 1H), 6.11 (dt, *J* = 15.8, 6.8 Hz, 1H), 3.88 (d, J = 9.4 Hz, 6H), 3.77 (s, 2H), 3.67 (s, 3H), 3.38 (dd, *J* = 6.7, 1.4 Hz, 2H), 3.35 (s, 2H). **^{13}C NMR** (101 MHz, $CDCl_3$) δ 171.8, 162.1 (d, *J* = 244.9 Hz), 149.1, 148.8, 134.3 (d, *J* = 3.2 Hz), 132.9, 130.6 (d, *J* = 7.9 Hz), 130.0, 125.0, 119.6, 115.1 (d, *J* = 21.1 Hz), 111.1, 108.7, 57.5, 56.5, 55.9, 55.8, 53.7, 51.4. **^{19}F NMR** (377 MHz, $CDCl_3$) δ -115.6 (s).

9.5 Synthesis of (±) Cinacalcet

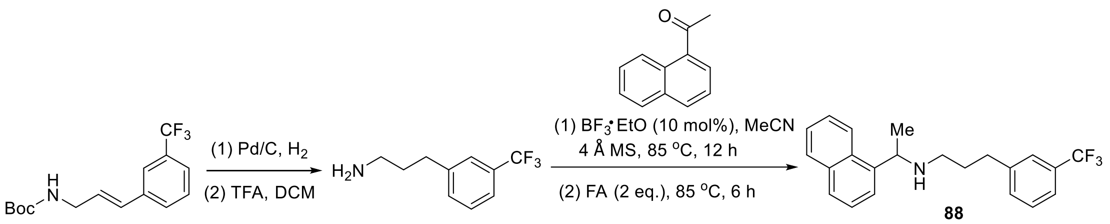

39 88 (±) Cinacalcet

Add Pd/C (10 wt%, 212 mg) to a solution of *tert*-butyl (*E*)-(3-(3-(trifluoromethyl)phenyl)allyl)carbamate (2.0 mmol, 1.0 equiv) in EtOH (40 mL). Stir the mixture at room temperature under H_2 (balloon, 1 atmosphere) for 24 h. Upon completion, filter the reaction mixture through a pad of celite. Concentrate the reaction mixture under reduced pressure. Purify the product by silica gel chromatography to afford the product in 96% yield.

To a stirred solution of *tert*-butyl (3-(3-(trifluoromethyl)phenyl)propyl)carbamate (1.5 mmol, 1.0 equiv) in DCM (0.5 M), trifluoroacetic acid (15.0 mmol, 10.0 equiv) was added. The reaction was stirred at room temperature for 2 h and monitored by TLC for completion. Then, the solvent was removed under reduced pressure, and the residue was dissolved in TBME (4 mL). The organic phase was extracted with 1 M HCl (3 × 4 mL). The combined aqueous phases were then adjusted to pH 11-12 with 2 M NaOH (aq) and extracted with DCM (3 × 4 mL). The combined organic layers were washed with brine. Subsequent drying over anhydrous Na_2SO_4 and evaporation of the organic solvent afforded 3-(3-(trifluoromethyl)phenyl)propan-1-amine (free base) in 95% yield.

To an oven-dried 50 mL round-bottom flask equipped with a reflux condenser was sequentially added 1-(naphthalen-1-yl)ethan-1-one (1.0 mmol, 1.0 equiv), 3-(3(trifluoromethyl)phenyl)propan-1-amine (1.2 mmol, 1.2 equiv), $BF_3{\cdot}Et_2O$ (0.1 mmol, 0.1 equiv), 4Å MS (100 mg) and MeCN (10 mL). Then the mixture was stirred at 85 °C for 12 h. After that, HCO_2H (2.0 mmol, 1.0 equiv) was added, and the reaction mixture was stirred at 85 °C for another 6 h. After being cooled to room temperature, the mixture was basified with saturated aqueous $NaHCO_3$ (2.0 mL) solution and extracted with DCM (3 × 2.0 mL). The organic layers were combined, washed with brine (2.0 mL), dried over anhydrous Na_2SO_4 and filtered. The filtrate was concentrated in vacuo to provide the crude product, which was purified by silica gel column chromatography using a mixture of ethyl acetate and hexane (1:5 to 1:2) with 1% Et_3N to give pure (±) cinacalcet as a colorless oil (**88**, 75% yield).

3-(3-(trifluoromethyl)phenyl)propan-1-amine[33]

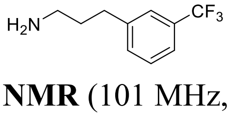

^{1}H NMR (400 MHz, $CDCl_3$) δ 7.49 – 7.40 (m, 2H), 7.39 – 7.31 (m, 2H), 2.82 – 2.64 (m, 4H), 1.84 – 1.72 (m, 2H), 1.38 (br, 2H). **^{13}C NMR** (101 MHz, $CDCl_3$) δ 143.0, 131.8 (q, *J* = 1.5 Hz), 130.6 (q, *J* = 31.8 Hz), 125.0 (q, *J* = 3.8 Hz), 124.3 (q, *J* = 272.2 Hz), 122.6 (q, *J* = 3.8 Hz), 41.6, 35.1, 33.0. **^{19}F NMR** (377 MHz, $CDCl_3$) δ -62.6 (s).

(±) Cinacalcet[33] (88)

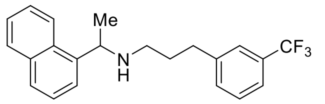

88

^{1}H NMR (400 MHz, $CDCl_3$) δ 8.18 (d, J = 7.6 Hz, 1H), 7.95 – 7.86 (m, 1H), 7.74 (d, J = 8.2 Hz, 1H), 7.65 (d, J = 7.2 Hz, 1H), 7.55 – 7.42 (m, 3H), 7.43 – 7.38 (m, 2H), 7.37 – 7.25 (m, 2H), 4.63 (q, J = 6.6 Hz, 1H), 2.84 – 2.51 (m, 4H), 1.87 – 1.80 (m, 2H), 1.50 (d, J = 6.6 Hz, 3H). **^{13}C NMR** (101 MHz, $CDCl_3$) δ 143.0, 140.9, 134.0, 131.8, 131.8, 131.3, 130.6 (q, J = 31.9 Hz), 129.0, 128.7, 127.3, 125.8, 125.7, 125.4, 125.1 (q, J = 3.7 Hz), 124.3 (q, J = 272.3 Hz), 122.9, 122.8, 122.7 (q, J = 3.8 Hz), 53.8, 47.3, 33.4, 31.8, 23.6. **^{19}F NMR** (377 MHz, $CDCl_3$) δ -62.5 (s).

9.6 Synthesis of (±) Fendiline

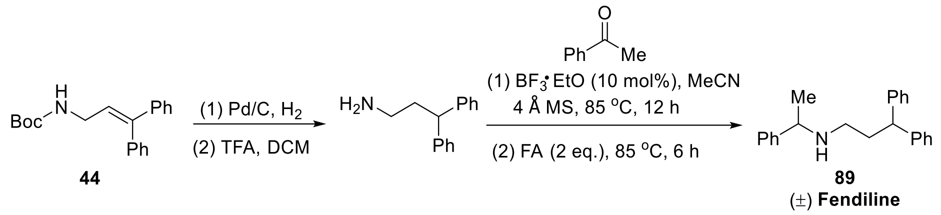

Add Pd/C (10 wt%, 212 mg) to a solution of *tert*-butyl (3,3-diphenylallyl)carbamate (2.0 mmol, 1.0 equiv) in EtOH (40 mL). Stir the mixture at room temperature under H_2 (balloon, 1 atmosphere) for 24 h. Upon completion, filter the reaction mixture through a pad of Celite. Concentrate the reaction mixture under reduced pressure. Purify the product by silica gel chromatography to afford the product in 99% yield.

To a stirred solution of *tert*-butyl (3,3-diphenylpropyl)carbamate (1.5 mmol, 1.0 equiv) in DCM (0.5 M), trifluoroacetic acid (15.0 mmol, 10.0 equiv) was added. The reaction was stirred at room temperature for 2 h and monitored by TLC for completion. Then, the solvent was removed under reduced pressure, and the residue was dissolved in TBME (4 mL). The organic phase was extracted with 1 M HCl (3 × 4 mL). The combined aqueous phases were then adjusted to pH 11-12 with 2 M NaOH (aq) and extracted with DCM (3 × 4 mL). The combined organic layers were washed with brine. Subsequent drying over anhydrous Na_2SO_4 and evaporation of the organic solvent afforded 3,3-diphenylpropan-1-amine (free base) in 96% yield.

To an oven-dried 50 mL round-bottom flask equipped with a reflux condenser was sequentially added acetophenone (1.0 mmol, 1.0 equiv), 3,3-diphenylpropan-1-amine (1.2 mmol, 1.2 equiv), $BF_3 \cdot Et_2O$ (1.0 mmol, 0.1 equiv), 4Å MS (100 mg) and MeCN (10 mL). Then the mixture was stirred at 85 °C for 12 h. After that, HCO_2H (2.0 mmol, 2 equiv) was added, and the reaction mixture was stirred at 85 °C for another 6 h. After being cooled to room temperature, the mixture was basified with saturated aqueous $NaHCO_3$ (2 mL) solution and extracted with DCM (3 × 3 mL). The organic layers were combined, washed with brine (2 mL), dried over anhydrous Na_2SO_4 and filtered. The filtrate was concentrated in vacuo to provide the crude product, which was purified by silica gel column chromatography using a mixture of ethyl acetate and hexane (1:10 to 1:5) with 1% Et_3N to give pure (±)-Fendiline as a colorless oil (**89**, 78% yield).

3,3-diphenylpropan-1-amine[33]

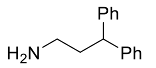

^{1}H NMR (400 MHz, $CDCl_3$) δ 7.29 – 7.20 (m, 8H), 7.20 – 7.11 (m, 2H), 4.01 (t, J = 7.9 Hz, 1H), 2.64 (t, J = 8.1 Hz, 2H), 2.19 (q, J = 7.5 Hz, 2H), 1.67 – 1.54 (br, 2H). **^{13}C NMR** (101 MHz, $CDCl_3$) δ144.8, 128.5, 127.8, 126.2, 48.8, 40.6, 39.3.

(±) Fendiline[33] (89)

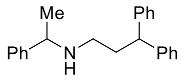

89

^{1}H NMR (400 MHz, $CDCl_3$) δ 7.42 – 6.92 (m, 15H), 3.96 (t, J = 7.8 Hz, 1H), 3.69 (q, J = 6.6 Hz, 1H), 2.54 – 2.37 (m, 2H), 2.31 – 2.09 (m, 2H), 1.29 (d, J = 6.6 Hz, 3H). **^{13}C NMR** (101 MHz, $CDCl_3$) δ 145.2, 144.9, 144.6, 128.4, 128.4, 127.9, 127.8, 126.9, 126.6, 126.2, 58.2, 49.0, 45.9, 35.8, 24.1.

9.7 Synthesis of (±)-norruspoline

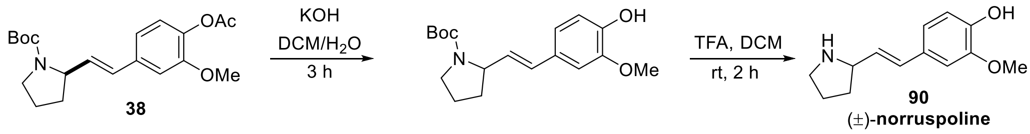

A stirred solution of *tert*-butyl (*R*,*E*)-2-(4-acetoxy-3-methoxystyryl)pyrrolidine-1-carboxylate (1.0 mmol, 1.0 equiv) in DCM/H_2O (3 mL, 10:1 v/v) was prepared, and potassium hydroxide (3.0 mmol, 3.0 equiv) was added to the mixture. The reaction mixture was stirred for 3 h. Upon completion, the reaction mixture was concentrated under reduced pressure to evaporate the solvent, and the crude residue was purified by silica gel column chromatography to afford the product in 95% yield.

To a stirred solution of *tert*-butyl *tert*-butyl (*E*)-2-(4-hydroxy-3-methoxystyryl) pyrrolidine-1-carboxylate (1.0 equiv) in DCM (0.5 M), trifluoroacetic acid (10.0 equiv) was added. The reaction was stirred at room temperature for 2 h and monitored by TLC for completion. Then, the solvent was removed under reduced pressure, and the residue was dissolved in TBME (4 mL). The organic phase was extracted with 1 M HCl (3 × 4 mL). The combined aqueous phases were then adjusted to pH 11-12 with 2 M NaOH (aq) and extracted with DCM (3 × 4 mL). The combined organic layers were washed with brine, dried over anhydrous Na_2SO_4, and concentrated to afford the free-base amine (**90**, 90% yield).

tert-butyl (*E*)-2-(4-hydroxy-3-methoxystyryl)pyrrolidine-1-carboxylate[34]

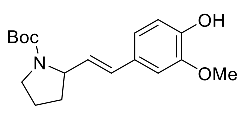

^{1}H NMR (400 MHz, $CDCl_3$) δ 6.89 – 6.77 (m, 3H), 6.32 (d, J = 15.5 Hz, 1H), 6.05 – 5.92 (d, J = 12.9 Hz, 1H), 5.78 (s, 1H), 4.59 – 4.25 (m, 1H), 3.89 (s, 3H), 3.45 (s, 2H), 2.16 – 2.00 (m, 1H), 2.00 – 1.61 (m, 3H), 1.43 (s, 9H). **^{13}C NMR** (101 MHz, $CDCl_3$) δ 154.8, 146.7, 145.3, 129.6, 129.4, 128.3, 120.0, 114.4, 108.2, 79.2, 59.0, 55.9, 46.3, 32.6, 31.7, 28.5, 23.5, 23.1.

(±)-norruspoline[34] **(90)**

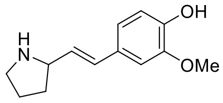

90

^{1}H NMR (400 MHz, $CDCl_3$) δ 6.86 (s, 1H), 6.81 (s, 2H), 6.41 (d, *J* = 15.7 Hz, 1H), 6.02 (dd, *J* = 15.8, 7.4 Hz, 1H), 4.10 (s, 2H), 3.85 (s, 3H), 3.70 (q, *J* = 7.3 Hz, 1H), 3.15 – 3.06 (m, 1H), 3.00 – 2.91 (m, 1H), 2.07 – 1.94 (m, 1H), 1.94 – 1.75 (m, 2H), 1.64 – 1.51 (m, 1H). **^{13}C NMR** (101 MHz, $CDCl_3$) δ 147.0, 145.7, 129.9, 129.9, 129.4, 120.0, 114.8, 108.2, 61.0, 55.8, 46.3, 32.4, 25.3.

10 References

1. (a) Y. Wu, M. Zhou, K. Chen, S. Chen, X. Xiao, Z. Ji, J. Zou, R. Liu, *Chinese Chemical Letters* 2021, **32**, 1675–1678; (b) P. Salas-Ambrosio, A. Tronnet, M. Badreldin, S. Ji, S. Lecommandoux, S. Harrisson, P. Verhaeghe, C. Bonduelle, *Polym. Chem.* 2022*,* **13***,* 6149–6161.
2. J. van Schijndel, D. Molendijk, K. van Beurden, L. A. Canalle, T. Noël, J. Meuldijk, *Eur. Polym. J.* 2020, **125**, 109534.
3. H. Cao, H. Jiang, H. Feng, J. M. C. Kwan, X. Liu, J. Wu, *J. Am. Chem. Soc.* 2018, **140**, 16360−16367.
4. X.-K. Qi, M.-J. Zheng, C. Yang, Y. Zhao, L. Guo, W. Xia, *J. Am. Chem. Soc.* 2023, **145**, 16630−16641.
5. Y.-X. Chen, J.-T. He, M.-C. Wu, Z.-L. Liu, K. Tang, P.-J. Xia, K. Chen, H.-Y. Xiang, X.-Q. Chen, H. Yang, *Org. Lett.* 2022, **24**, 3920−3925.
6. S.-Q. Guo, H.-Q. Yang, Y.-Z. Jiang, A.-L. Wang, G.-Q. Xu, Y.-C. Luo, Z.-X. Chen, H. Zheng, P.-F. Xu, *Green Chem.* 2022, **24**, 3120−3124.
7. Y.-S. Jiang, F. Liu, M.-S. Huang, X.-L. Luo, P.-J. Xia, *Org. Lett.* 2022, *24*, 8019−8024.
8. Z. Jia, L. Cheng, L. Zhang, S. Luo, *Nat. Commun.* 2024, **15***,* 4044.
9. Y. L. He, X. Dan, Y. R. Tang, Q. Yang, W. Wang, T. Y. F. Cai, *Green Chem.* 2021, **23**, 9577−9582.
10. Z. X. Ding, X. F. Chen, M. Antonietti, X. C. Wang, *ChemSusChem.* 2011, **4**, 274–281.
11. C. Zhao, Z. Chen, J. Xu, Q. Liu, H. Xu, H. Tang, G. Li, Y. Jiang, F. Qu, Z. Lin, X. Yang, *Appl. Catal. B: Environ.* 2019, **256**, 117867.
12. J. Zheng, L. Zhang, *Appl. Catal. B: Environ.* 2018, **237**, 1–8.
13. S. Sun, W. Wang, D. Li, L. Zhang, D. Jiang, *ACS Catal.* 2014, *4* 3498–3503.
14. N. G. Connelly, W. E. Geiger, *Chem. Rev.* 1996, **96**, 877–910.
15. H. Yue, C. Zhu, R. Kancherla, F. Liu, M. Rueping, *Angew. Chem., Int. Ed.* 2020, **59**, 5738–5746.
16. Y. C. Lei, R. Y. Qiu, L. J. Zhang, C. H. Xu, Y. X. Pan, X. B. Qin, H. R. Li, L. J. Xu, Deng, Y. H. *ChemCatChem.* 2015, **7**, 1275–1279.
17. H. Zhang, C. C. Huang, X.-A. Yuan, S. Y. Yu, *J. Am. Chem. Soc.* 2022, **144**, 10958–10967.
18. J. M. Ketcham, F. S. P. Cardoso, B. Biannic, H. Piras, Aponick, A. *Isr. J. Chem.* 2013, **53**, 923–931.
19. W. L. Bao, Y. Y. Liu, X. Lv, *Synthesis*. 2008, **12**, 1911–1917.
20. T. Rukkijakan, S. Akkarasamiyo, S. Sawadjoon, J. S. M. Samec, *J. Org. Chem.* 2018, **83**, 4099–4104.
21. M. Halder, M. M. Islam, S. Ahammed, S. M. Islam, *RSC Adv.* 2016, *6*, 8282–8289.
22. L. Ouyang, Y. P. Xia, R. Miao, J. H. Liao, R. S. Luo, *Org. Biomol. Chem.* 2022, **20**, 2621–2625.
23. H. C. Zheng, S. Ghanbari, S. Nakamura, D. G. Hall, *Angew. Chem., Int. Ed.* 2012, **51**, 6187–6190.
24. Y. L. Zhang, L. Yang, J. Wu, C. Zhu, P. Wang, *Org. Lett.* 2020*,* **22***,* 7768–7772.

25. Y. Lee, S. Shabbir, S. Lee, H. Ahna, H. Rhee, *Green Chem.* 2015, **17**, 3579–3583.
26. C. Schlepphorst, B. Maji, F. Glorius, *ACS Catal.* 2016, **6**, 4184–4188.
27. L. J. Zhang, C. N. Dong, C. J. Ding, J. Chen, W. J. Tang, H. Li, R. L. J. Xu, J. L. Xiao, *Adv. Synth. Catal.* 2013, **355**, 1570–1578.
28. S. Han, X. Shen, D. Kong, G. Zi, G. Hou, J. Zhang, *J. Org. Chem.* 2019, **84**, 4318–4329.
29. F. Moschona, M. Tsitopoulou, M. Efstratiou, M. Koutiva, G. Rassias, *Eur. J. Org. Chem.* 2024, **27**, e202400079.
30. W. Wang, R. Zhou, Z.-J. Jiang, K. Wang, H.-Y. Fu, X.-L. Zheng, H. Chen, R.-X. Li, *Adv. Synth. Catal.* 2014, **356**, 616–622.
31. T. Menard, A. Laverny, S. E. Denmark, *J. Org. Chem.* 2021, **86**, 14290–14310.
32. C. Bruno, G. Lentini, A. Catalano, A. Carocci, A. Lovece, A. D. Mola, M. M. Cavalluzzi, P. Tortorella, F. Loiodice, G. Iaccarino, P. Campiglia, E. Novellino, C. Franchini, *Synthesis.* 2010, **24**, 4193–4198.
33. Z. Luo, S. Wan, Y. Pan, Z. Yao, X. Zhang, B. Li, J. Li, L. Xu, Q.-H. Fan, *Asian J. Org. Chem.* 2022, **11**, e202100707.
34. K. P. Sujith, S. R. Premakumari, K.-B. Cho, A. Lee, *J. Am. Chem. Soc.* 2024, **146**, 14816−14828.

11 NMR Spectra

^{1}H NMR spectra of **a5** (400 MHz, $CDCl_3$)

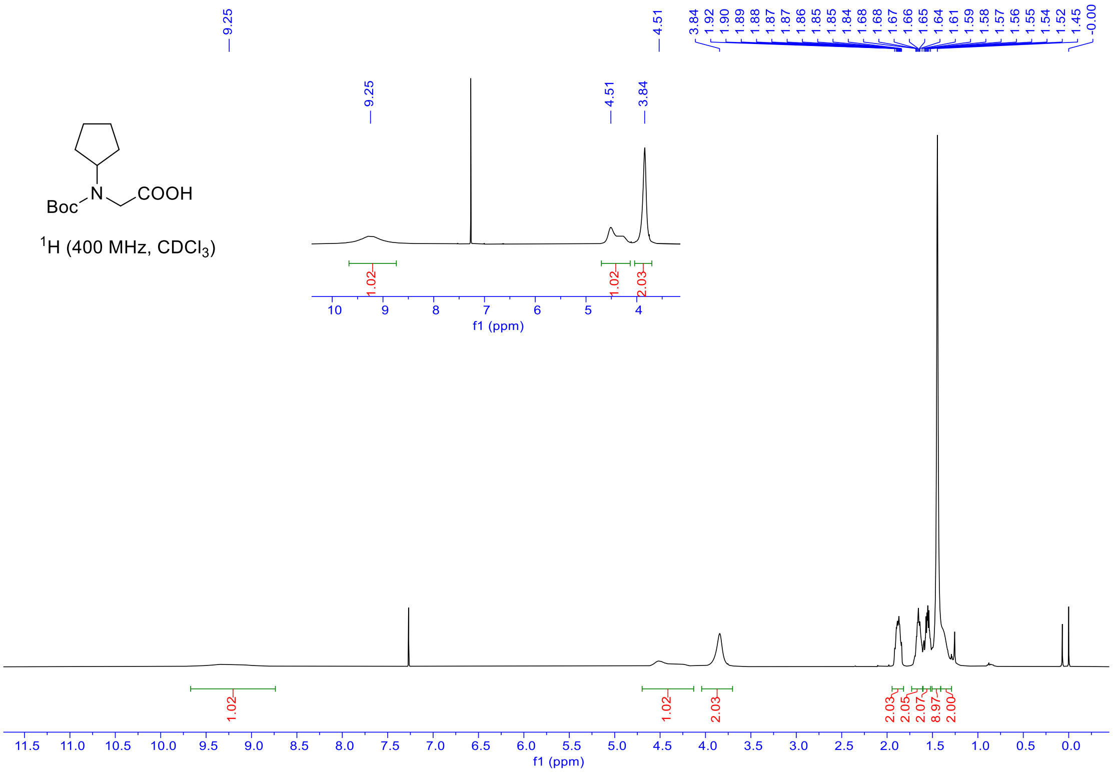

^{13}C NMR spectra of **a5** (101 MHz, $CDCl_3$)

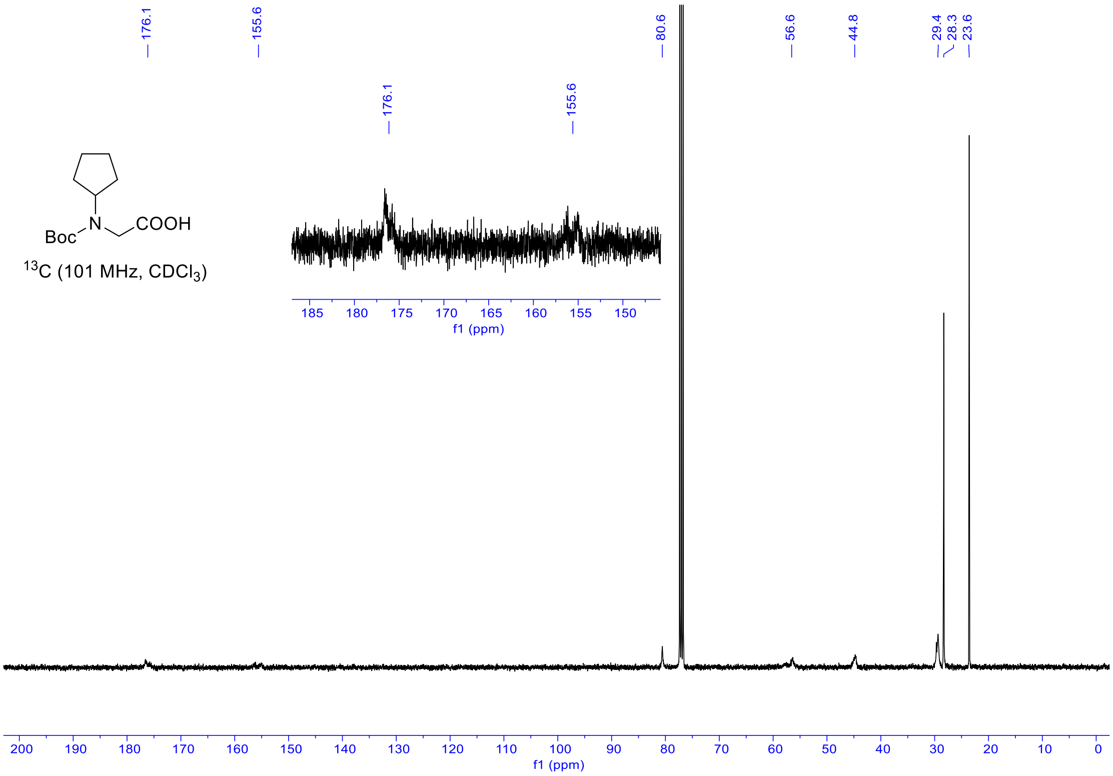

^{1}H NMR spectra of **a6** (400 MHz, $CDCl_3$)

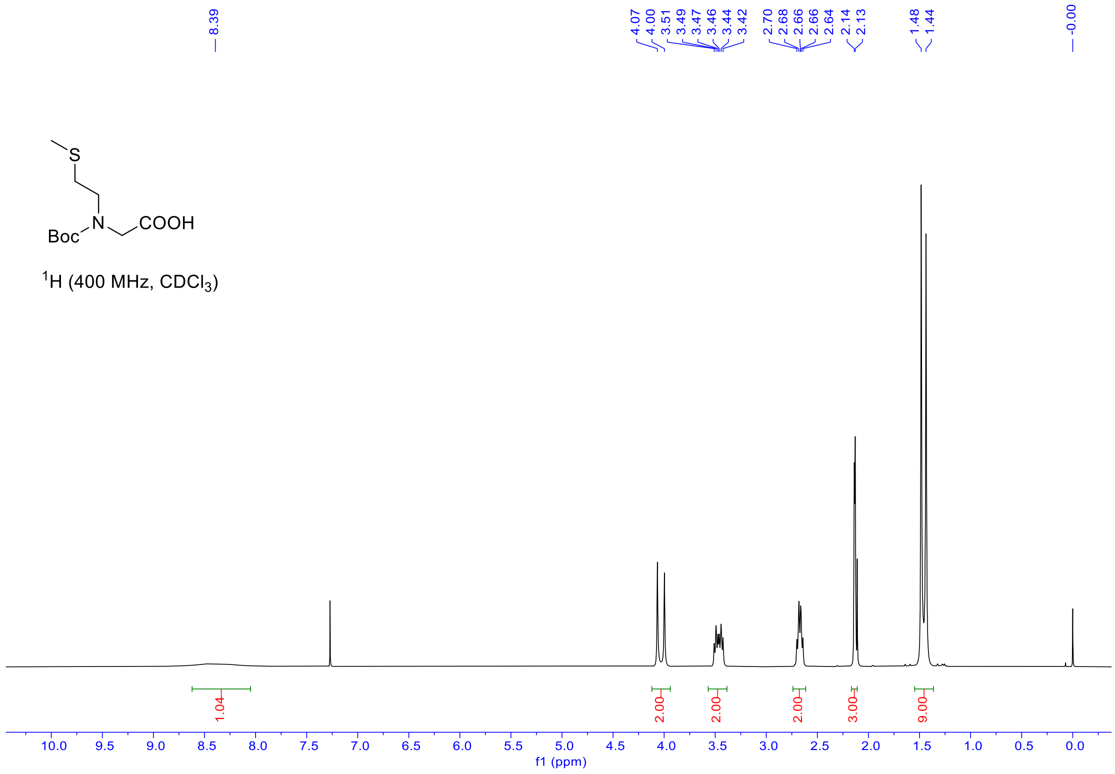

^{13}C NMR spectra of **a6** (101 MHz, $CDCl_3$)

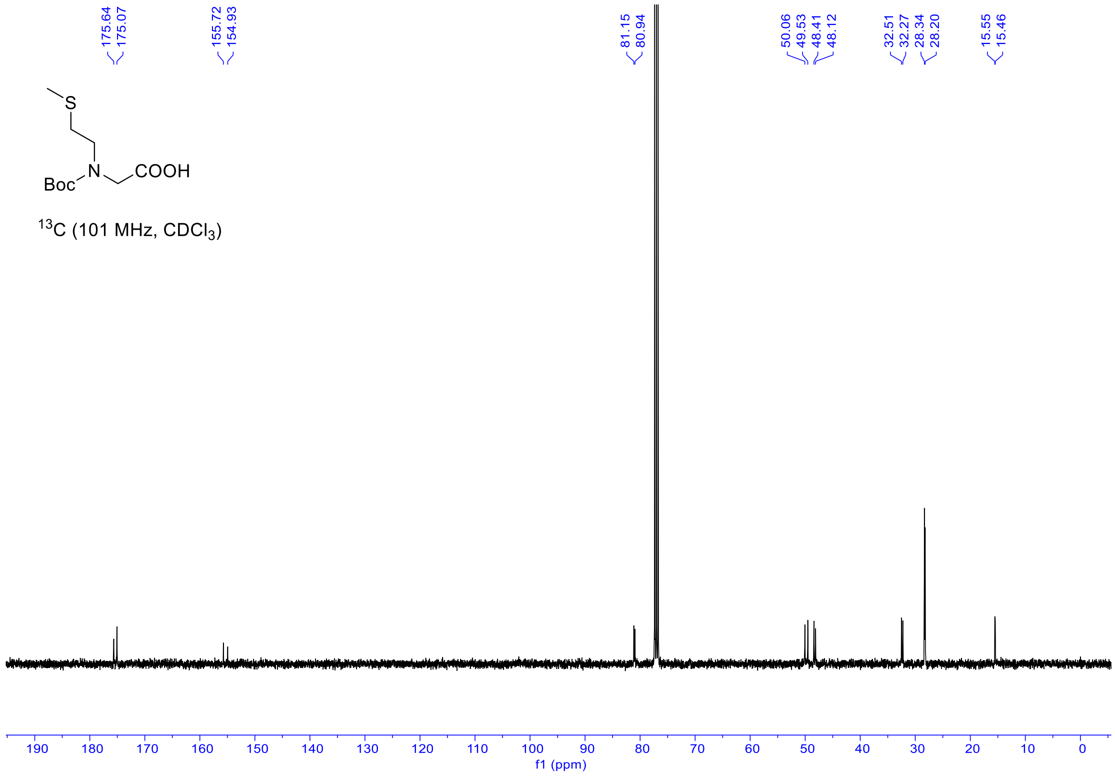

^{1}H NMR spectra of **b36** (400 MHz, $CDCl_3$)

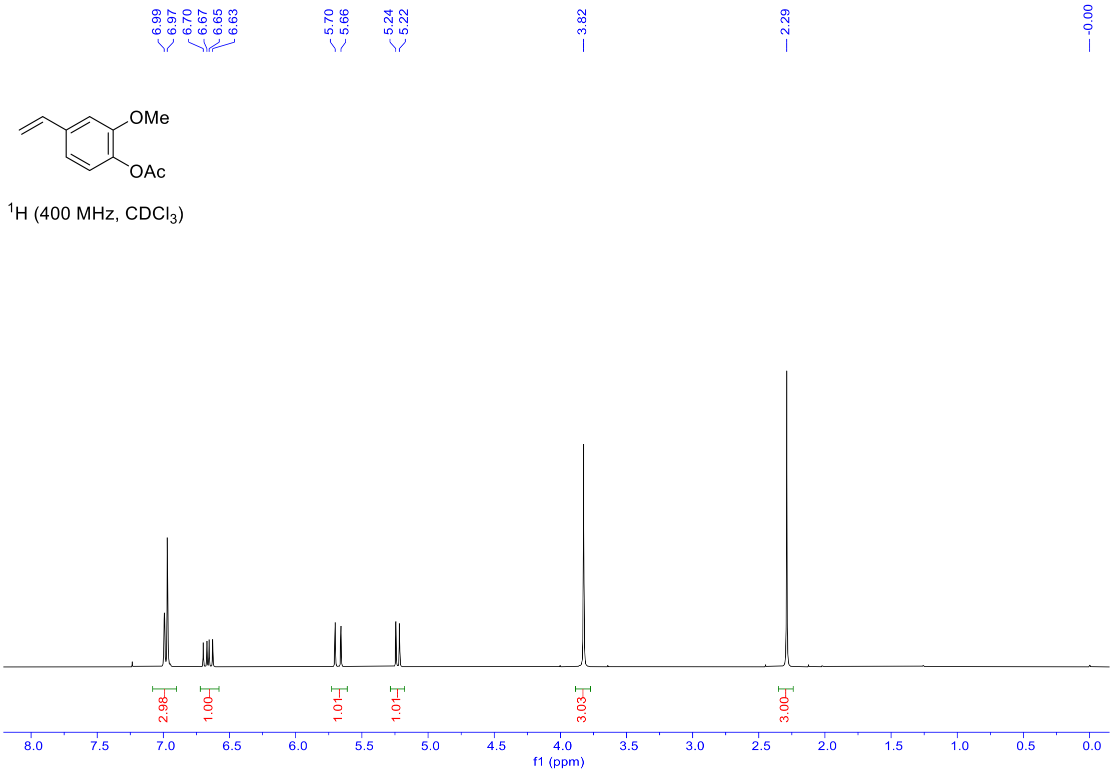

^{13}C NMR spectra of **b36** (101 MHz, $CDCl_3$)

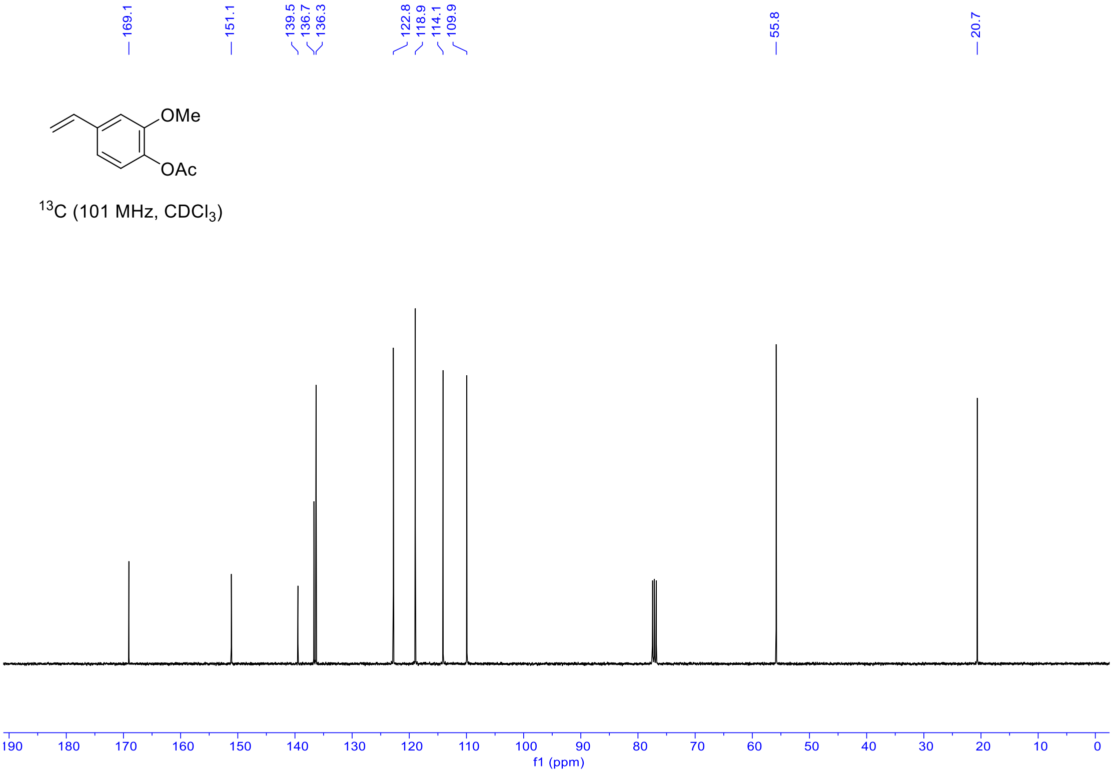

^{1}H NMR spectra of **b42** (400 MHz, CDCl$_3$)

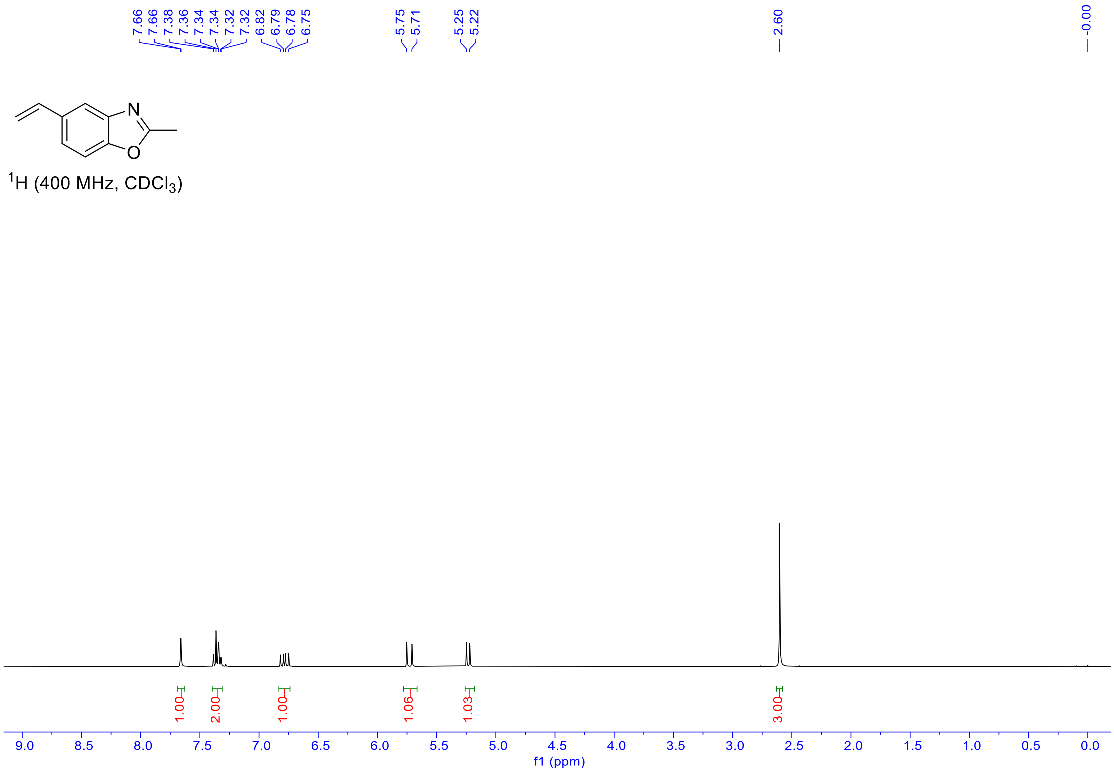

^{13}C NMR spectra of **b42** (101 MHz, CDCl$_3$)

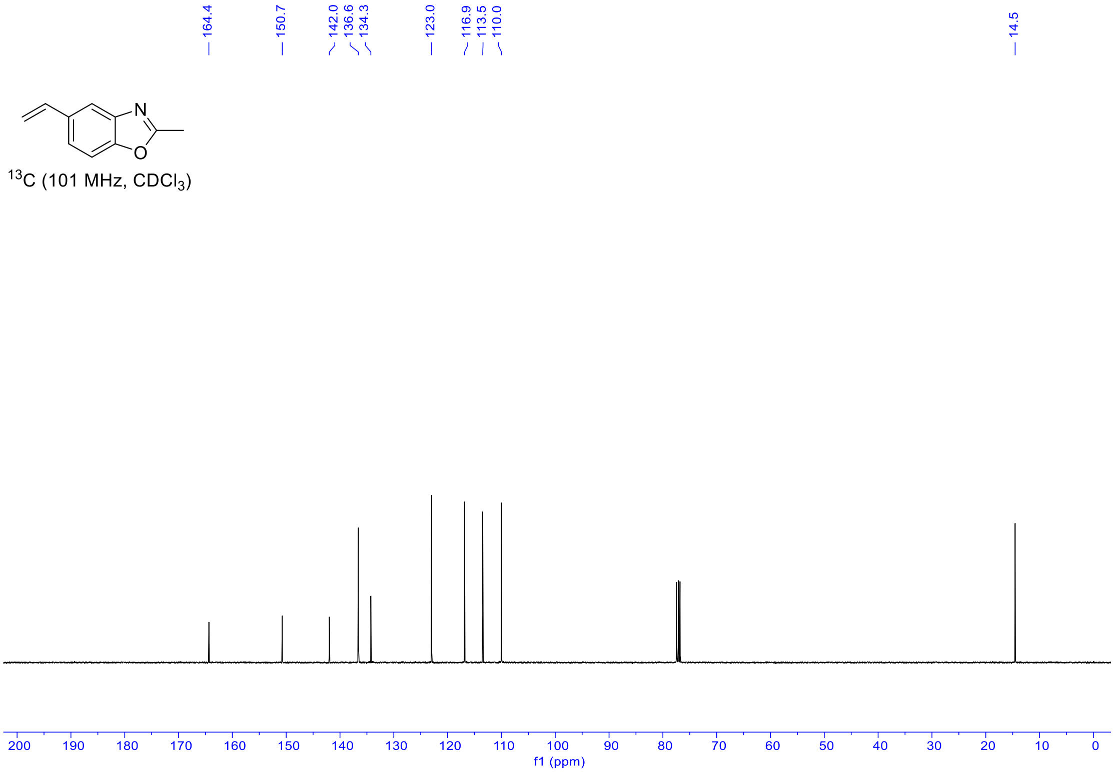

^{1}H NMR spectra of **b60** (400 MHz, $CDCl_3$)

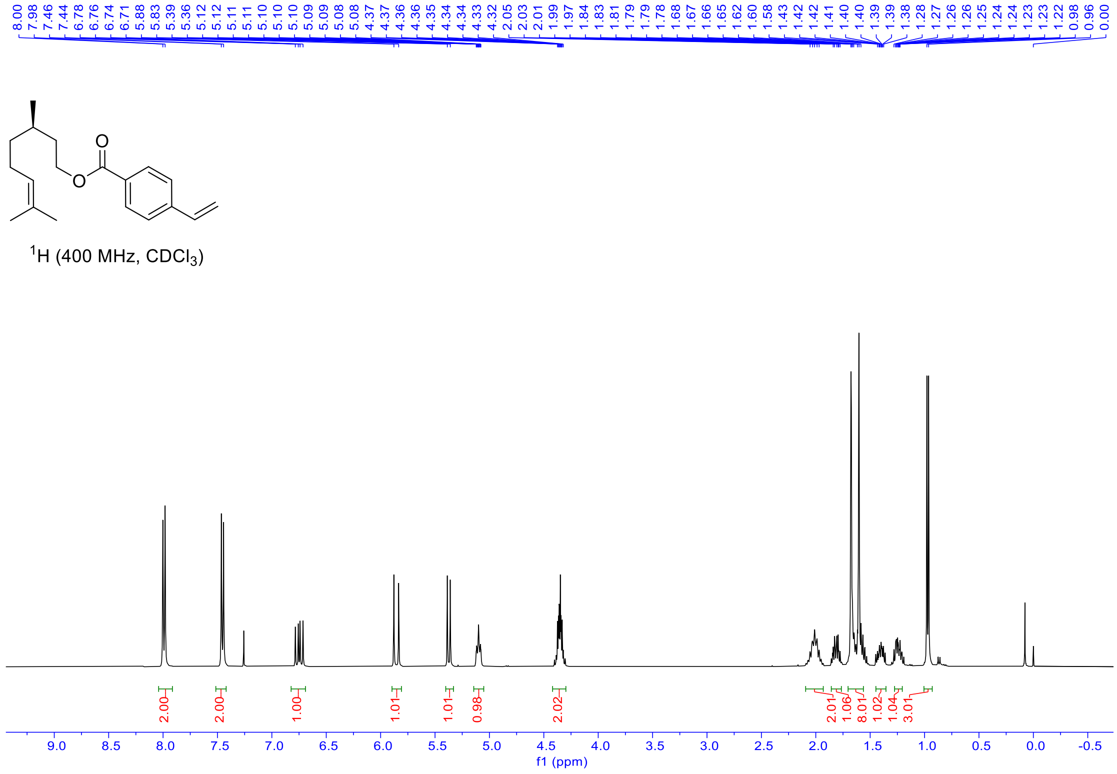

^{13}C NMR spectra of **b60** (101 MHz, $CDCl_3$)

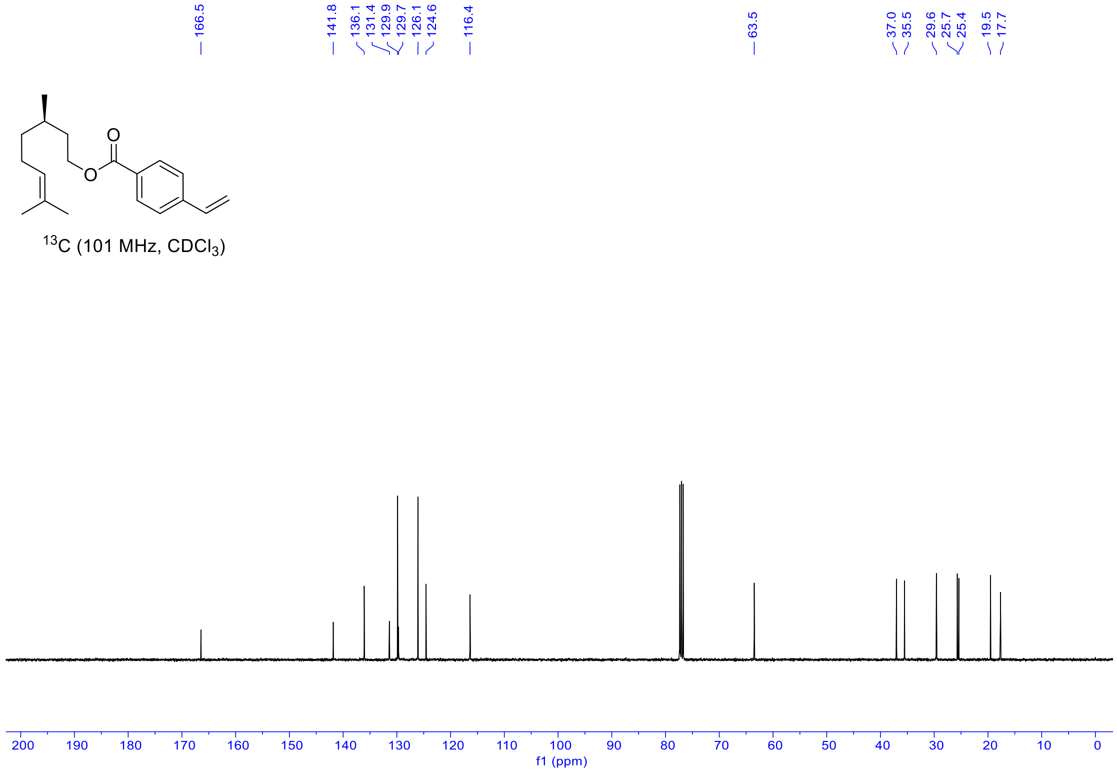

^{1}H NMR spectra of **b62** (400 MHz, $CDCl_3$)

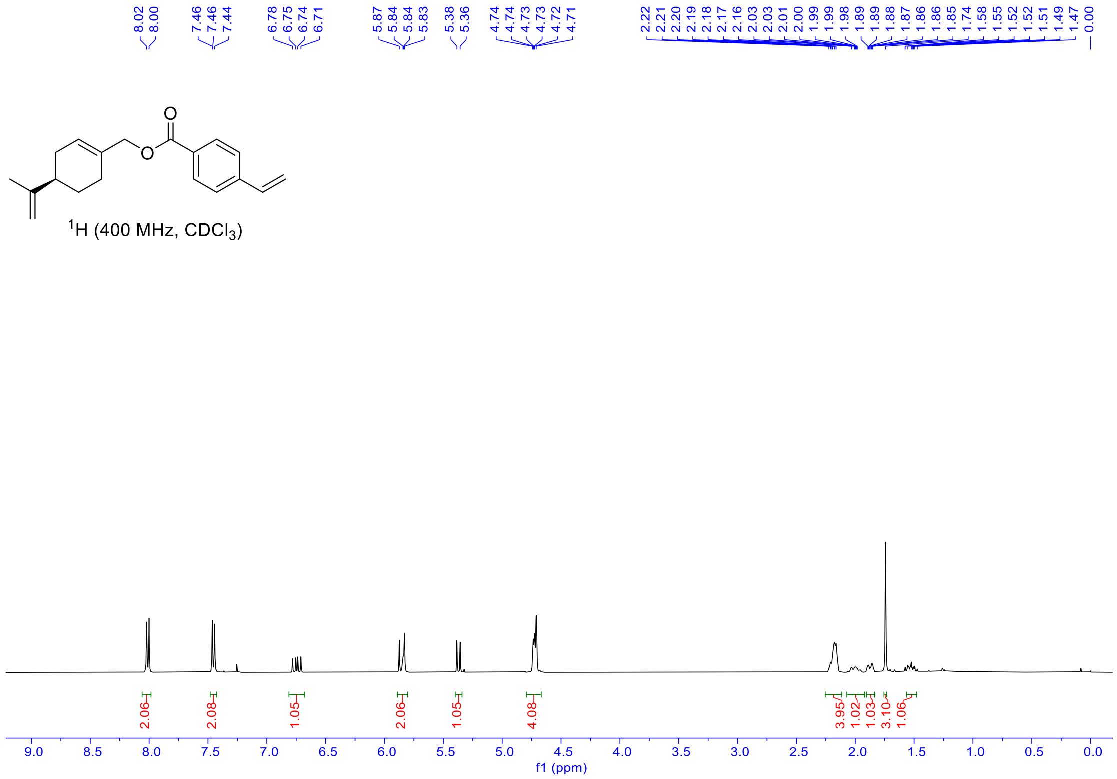

^{13}C NMR spectra of **b62** (101 MHz, $CDCl_3$)

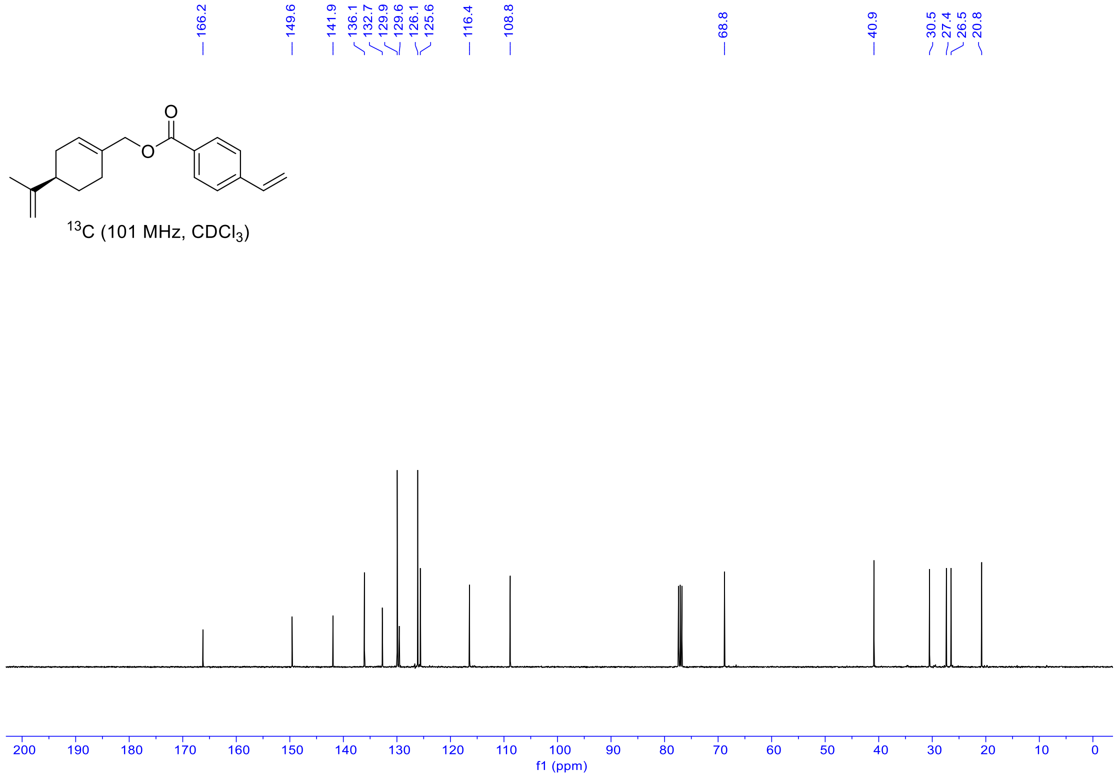

^{1}H NMR spectra of **b64** (400 MHz, $CDCl_3$)

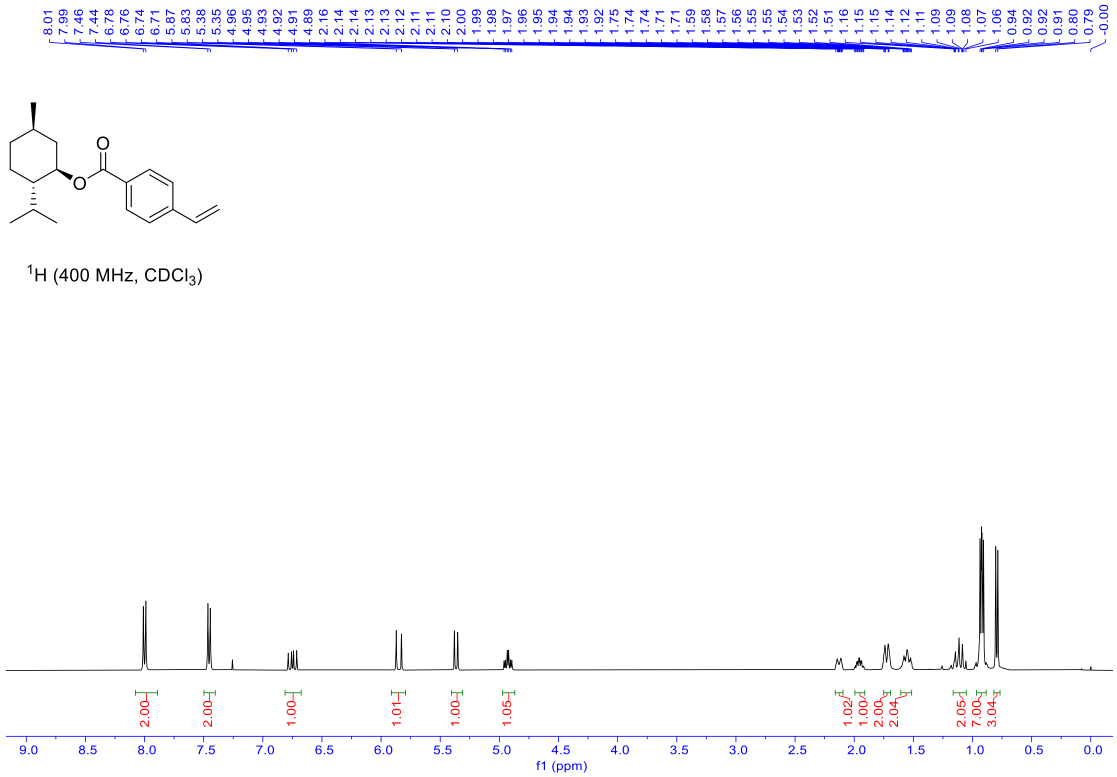

^{13}C NMR spectra of **b64** (101 MHz, $CDCl_3$)

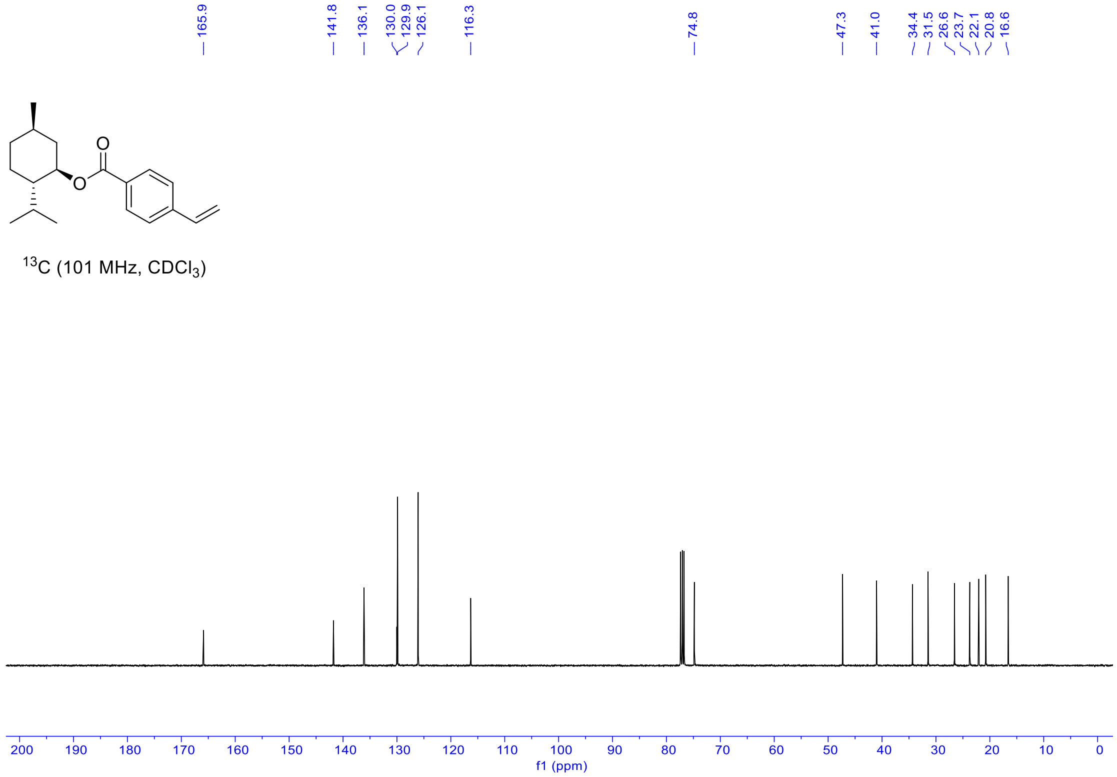

^{1}H NMR spectra of **b66** (400 MHz, $CDCl_3$)

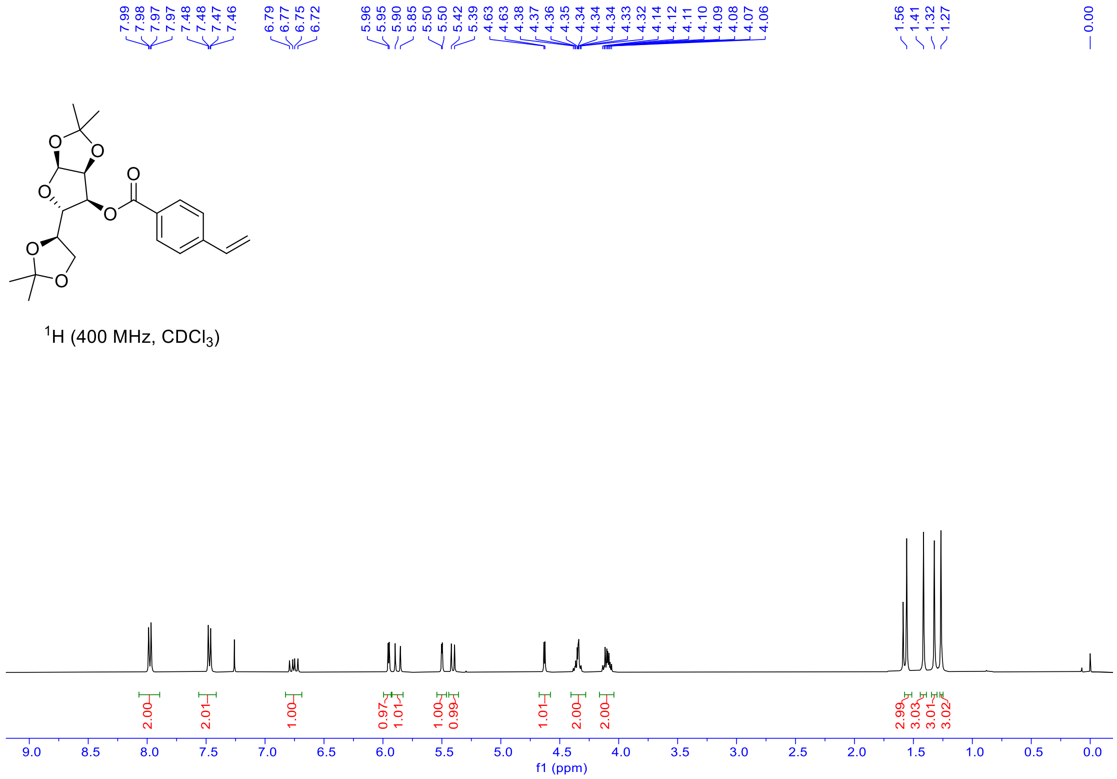

^{13}C NMR spectra of **b66** (101 MHz, $CDCl_3$)

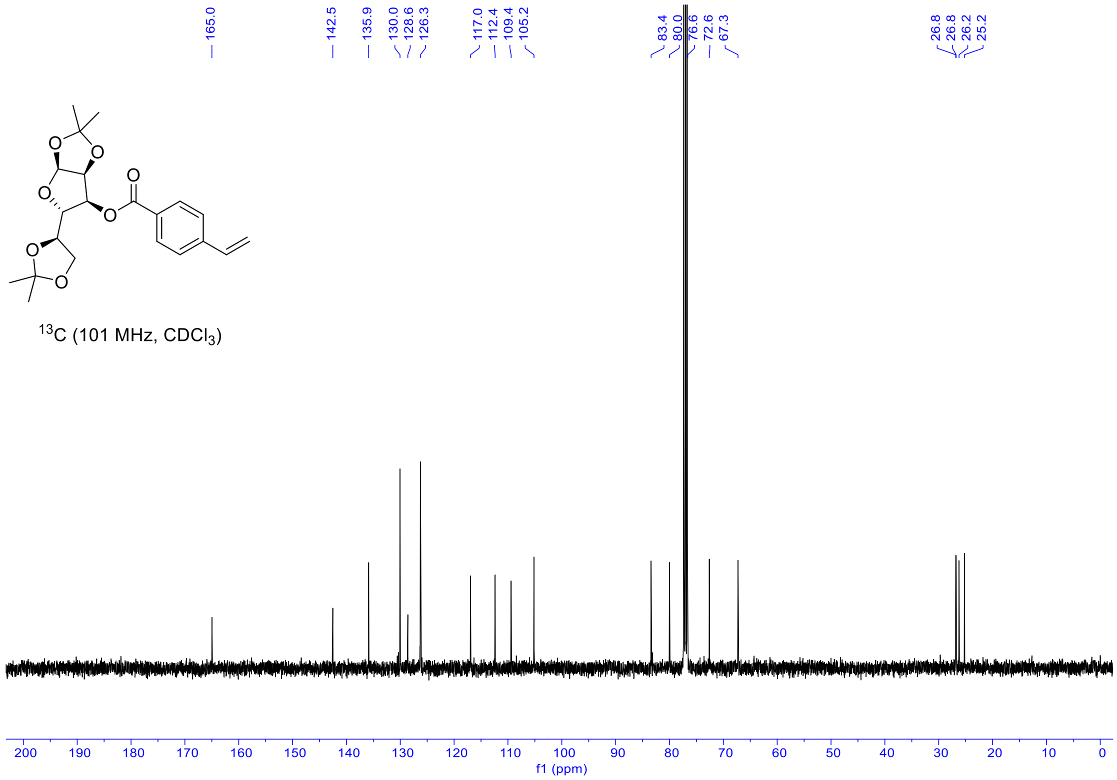

^{1}H NMR spectra of **b68** (400 MHz, $CDCl_3$)

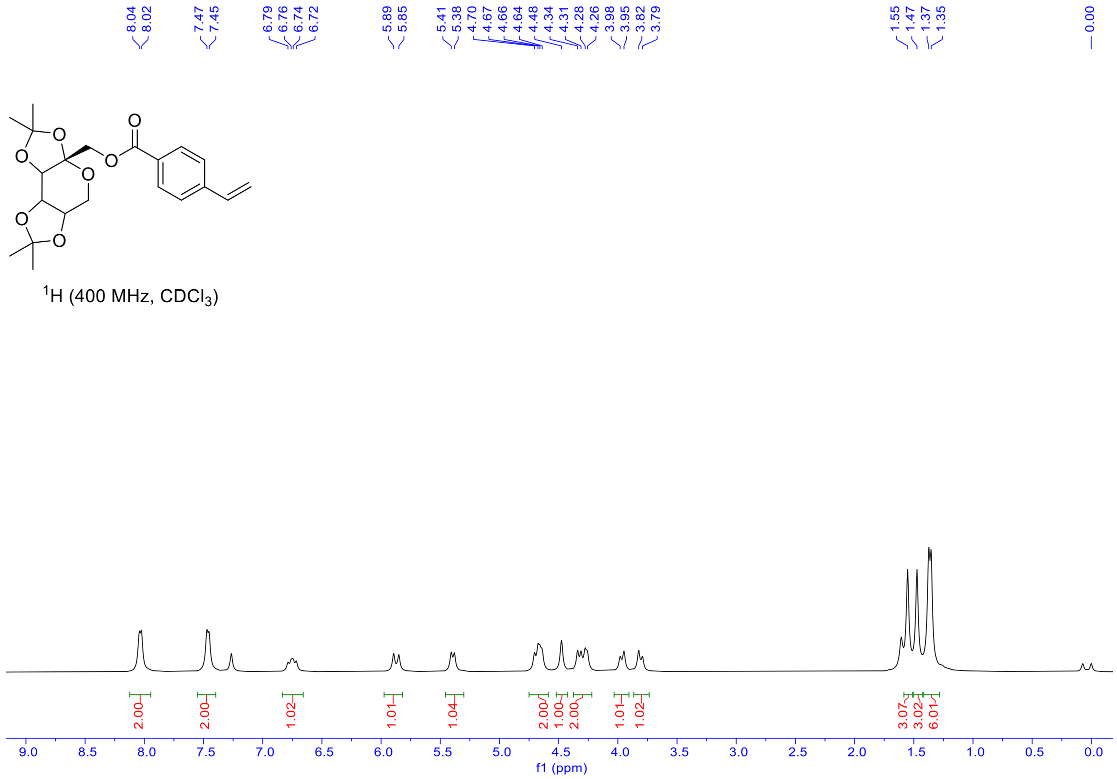

^{13}C NMR spectra of **b68** (101 MHz, $CDCl_3$)

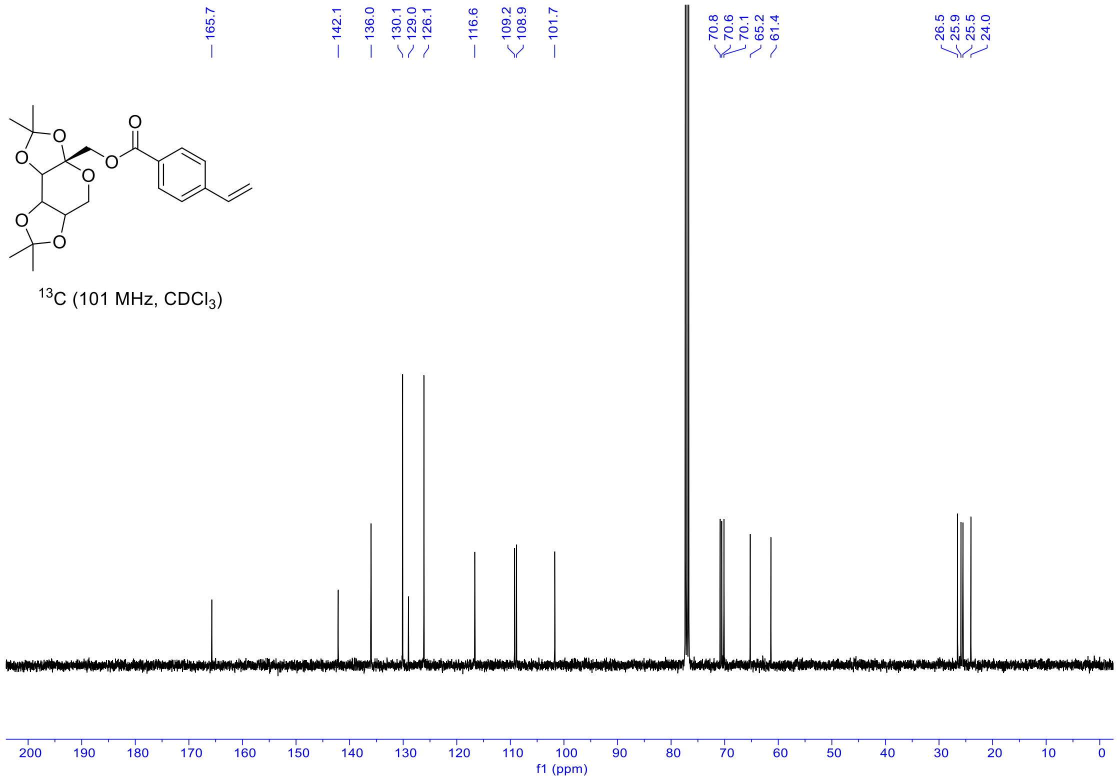

^{1}H NMR spectra of **b70** (400 MHz, $CDCl_3$)

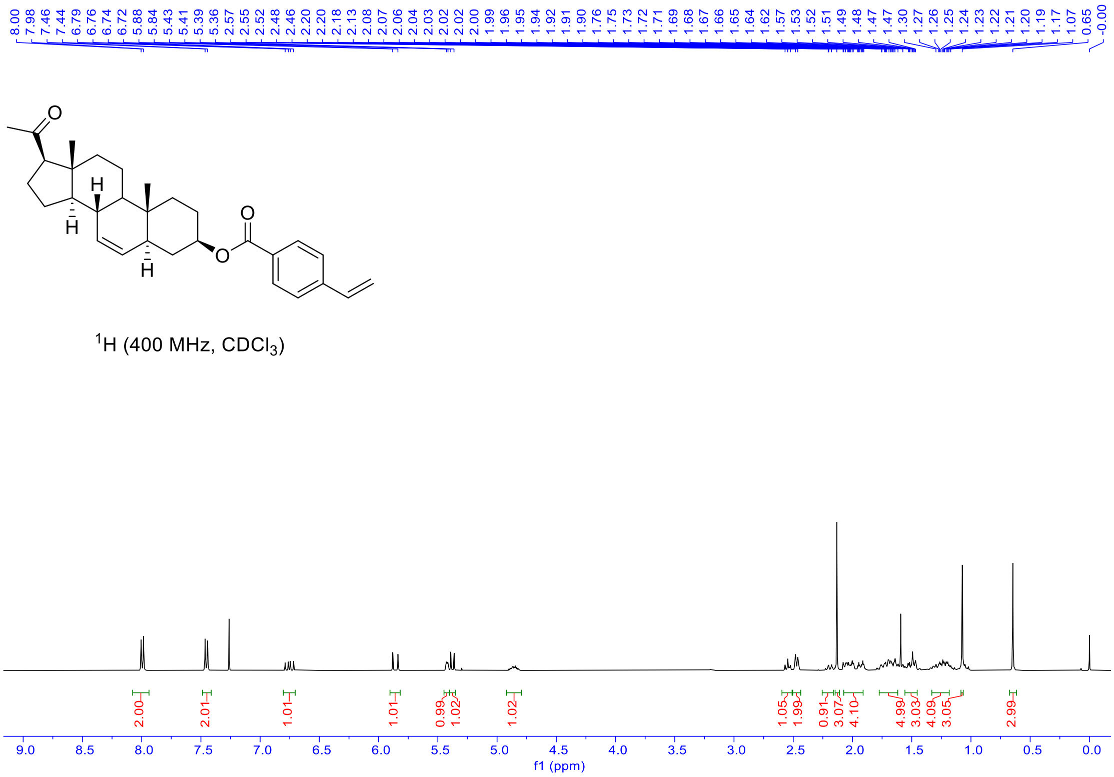

^{13}C NMR spectra of **b70** (101 MHz, $CDCl_3$)

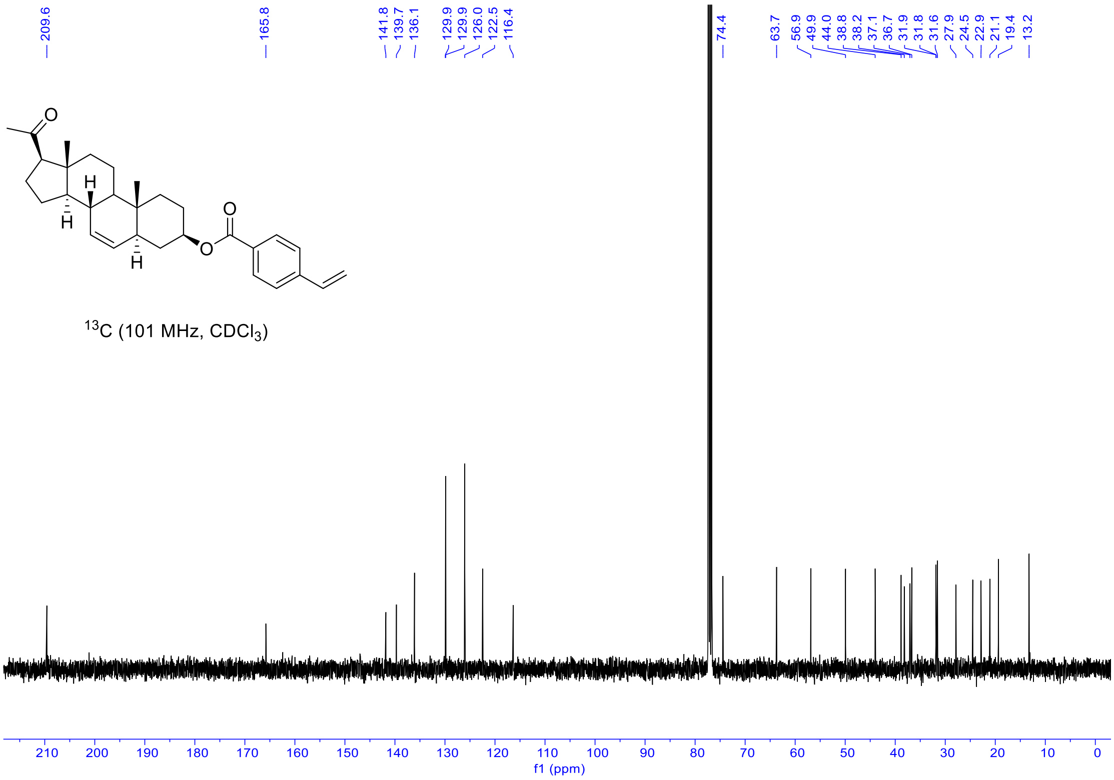

^{1}H NMR spectra of **b72** (400 MHz, $CDCl_3$)

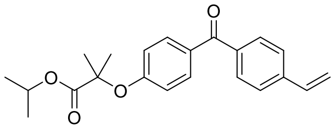

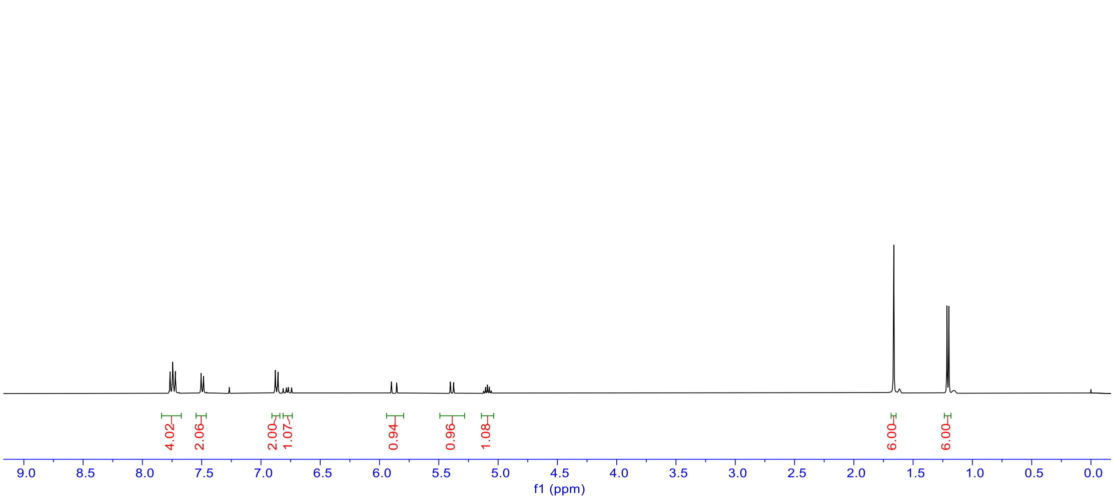

^{13}C NMR spectra of **b72** (101 MHz, $CDCl_3$)

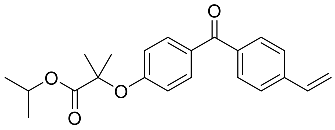

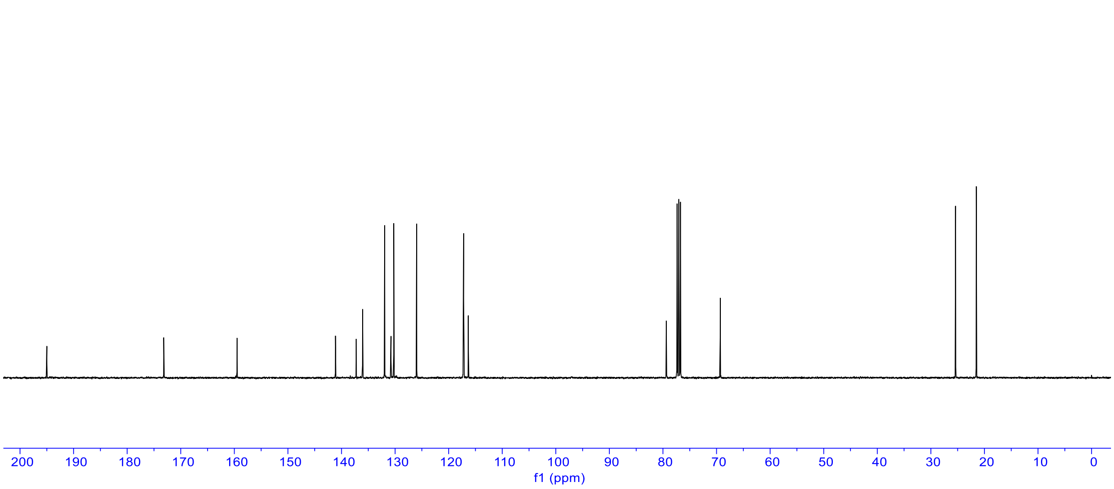

^{1}H NMR spectra of **b74** (400 MHz, $CDCl_3$)

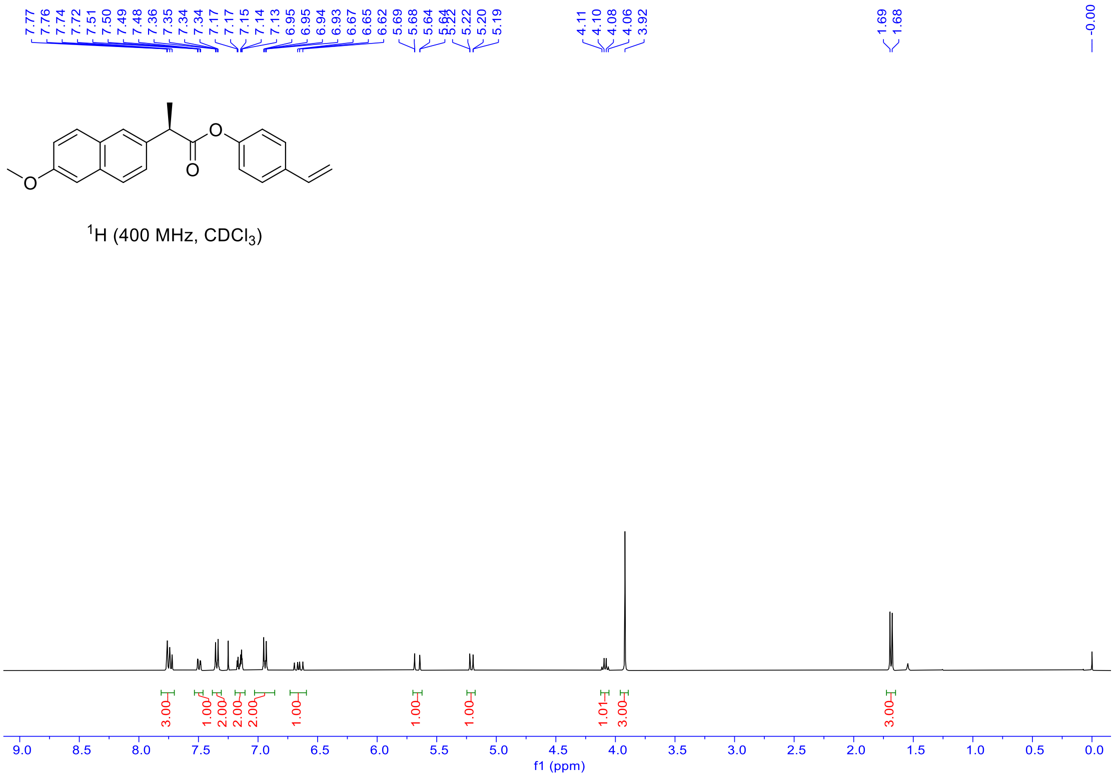

^{13}C NMR spectra of **b74** (101 MHz, $CDCl_3$)

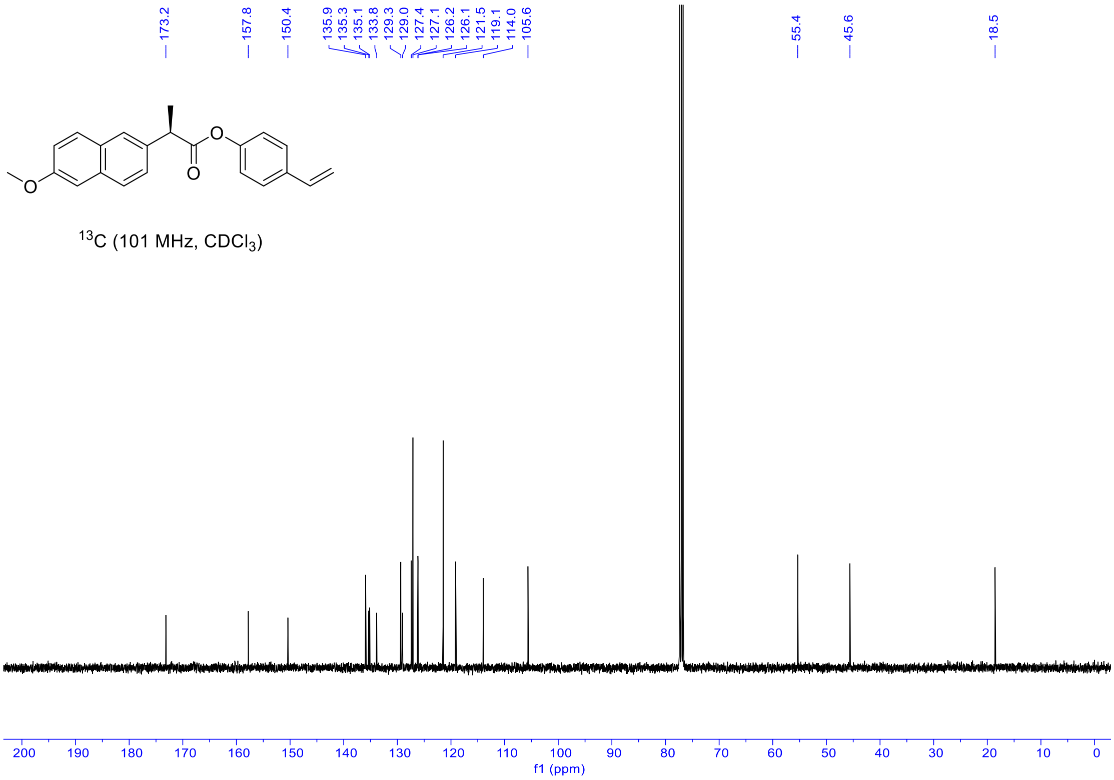

^{1}H NMR spectra of **b76** (400 MHz, $CDCl_3$)

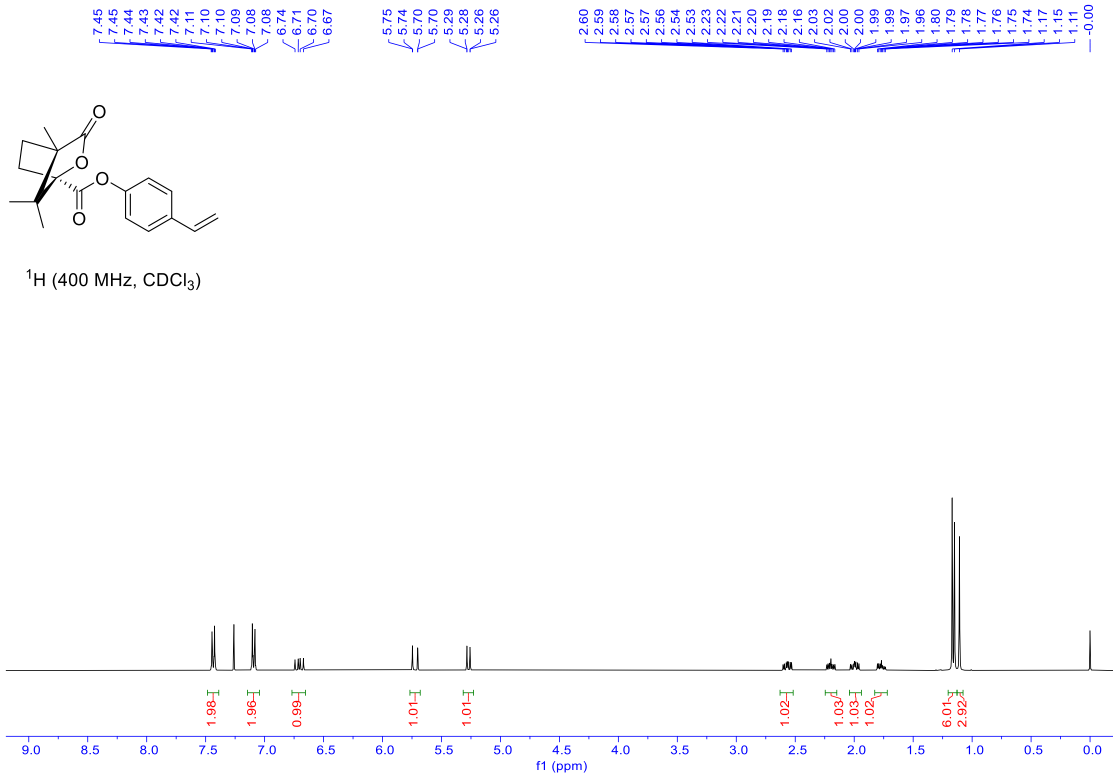

^{13}C NMR spectra of **b76** (101 MHz, $CDCl_3$)

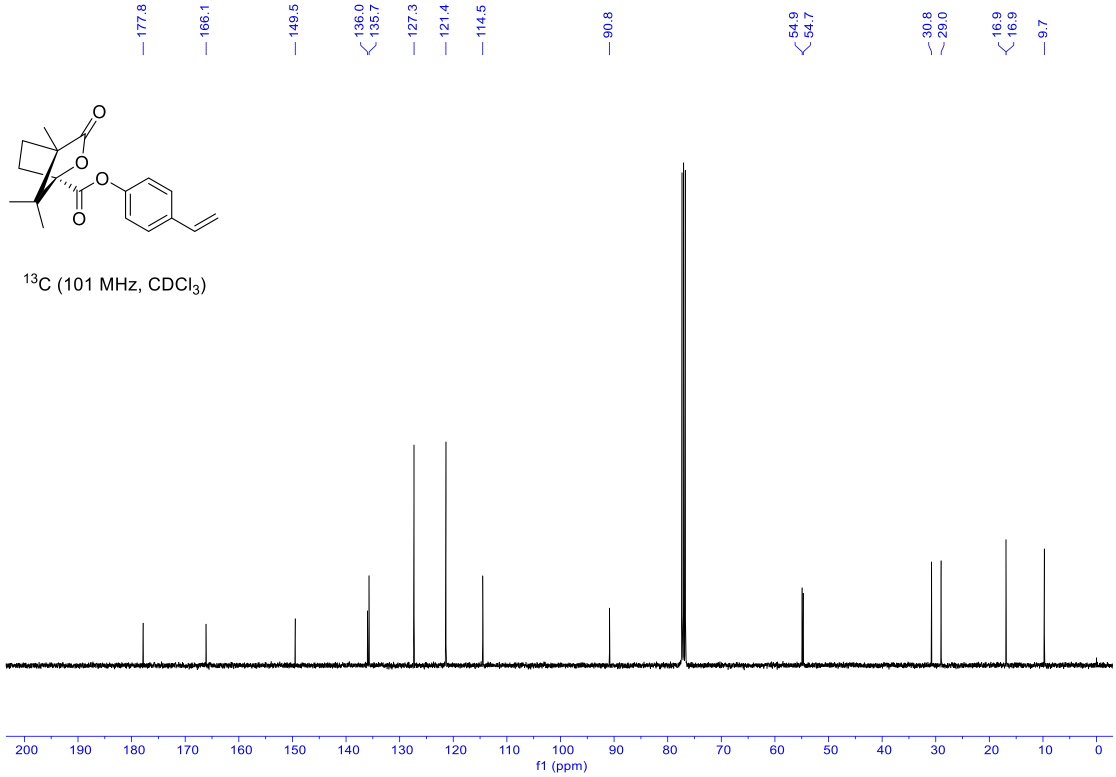

^{1}H NMR spectra of **b78** (400 MHz, $CDCl_3$)

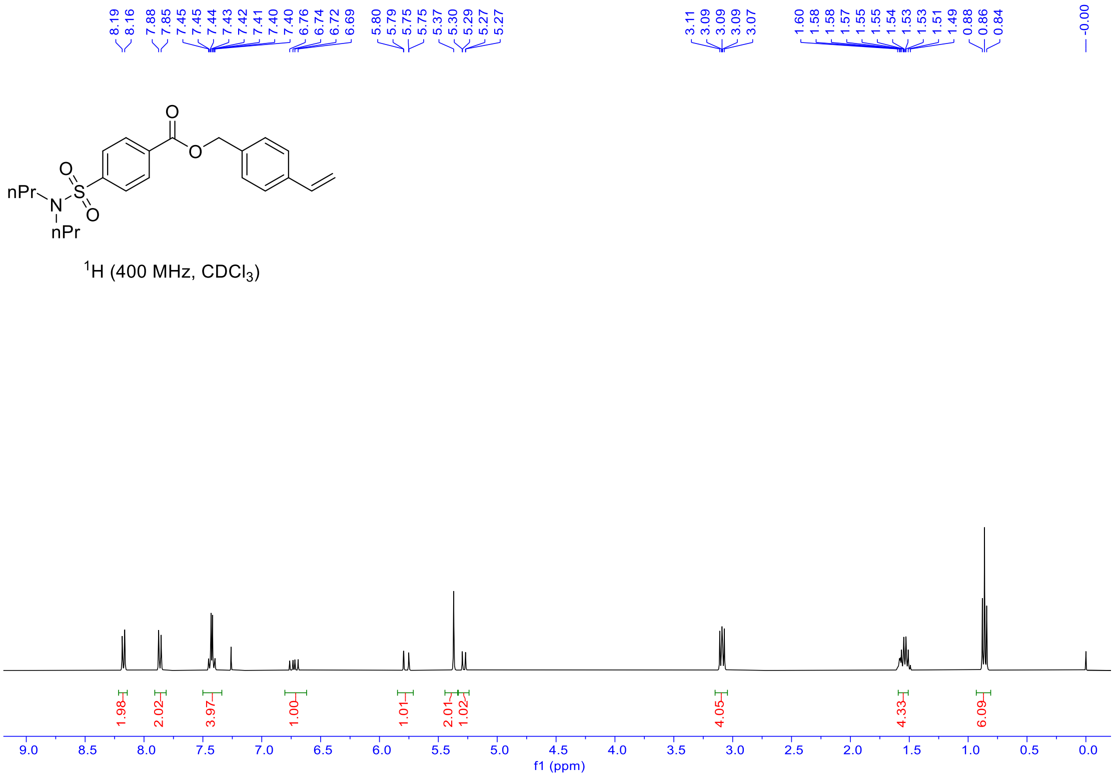

^{13}C NMR spectra of **b78** (101 MHz, $CDCl_3$)

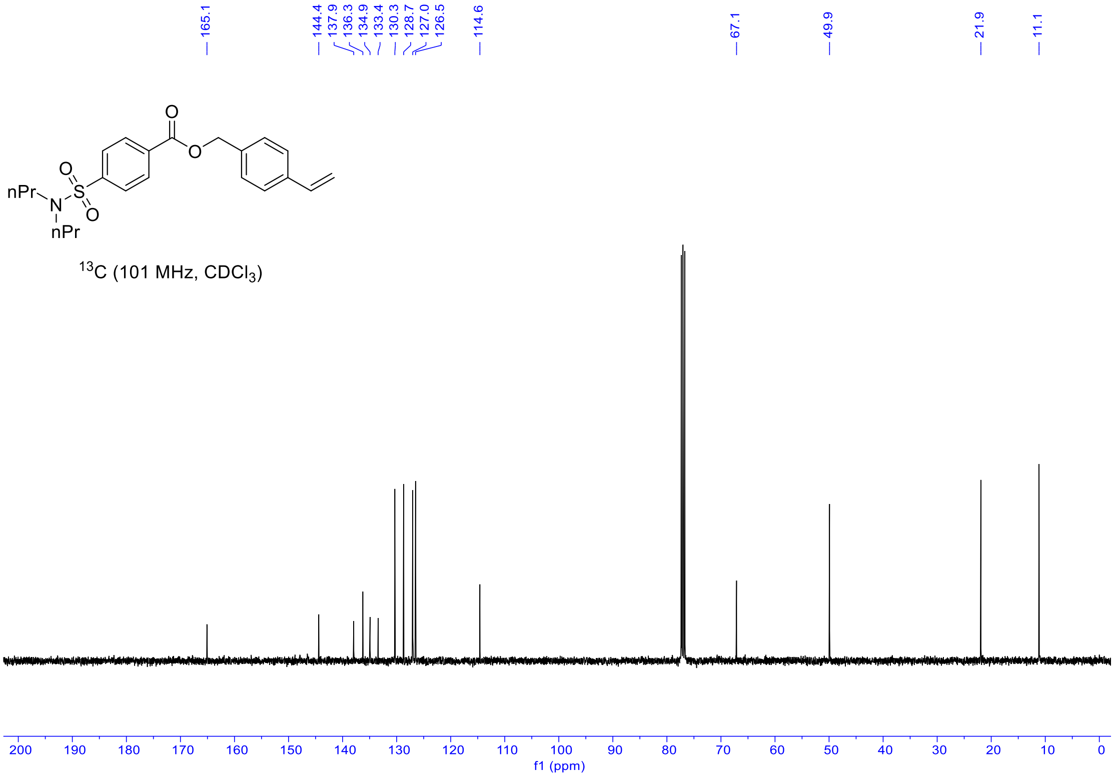

^{1}H NMR spectra of **b80** (400 MHz, $CDCl_3$)

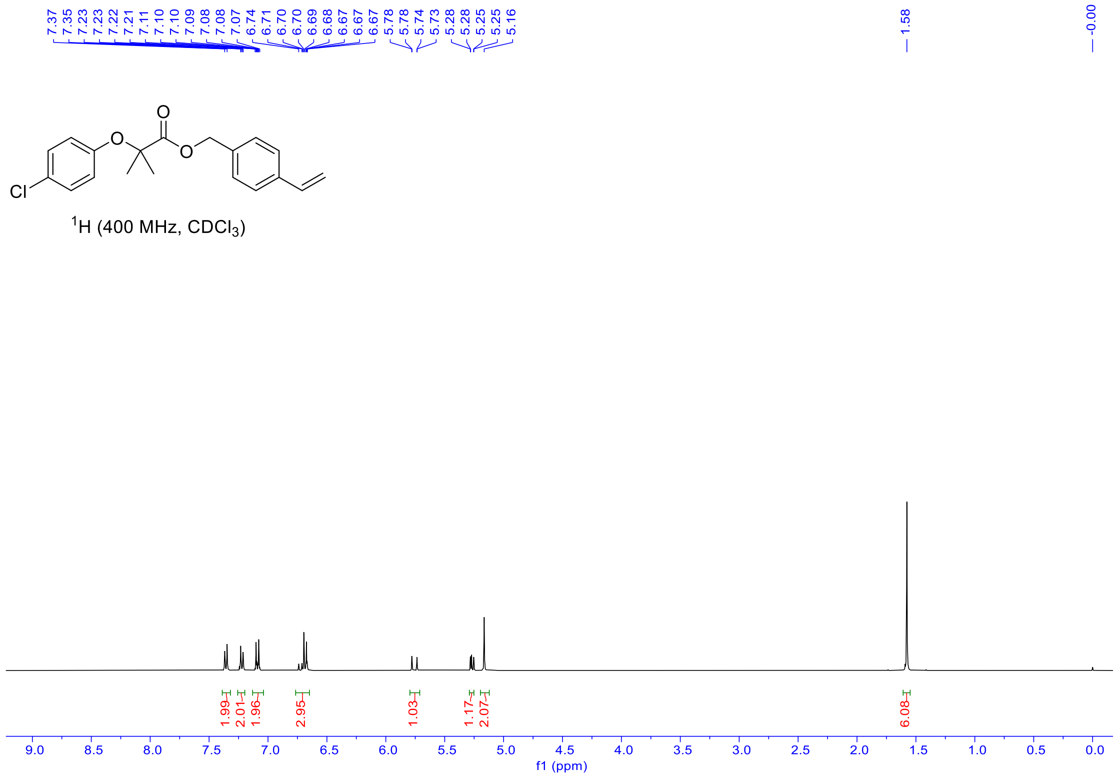

^{13}C NMR spectra of **b80** (101 MHz, $CDCl_3$)

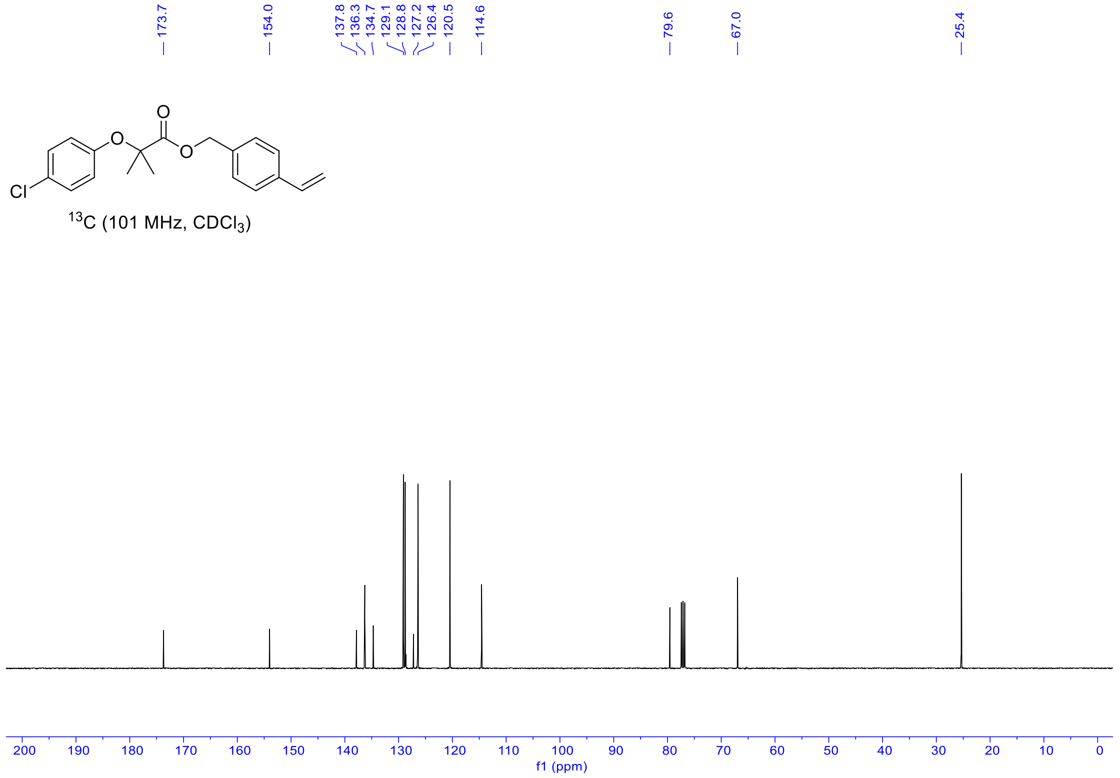

^{1}H NMR spectra of **b82** (400 MHz, $CDCl_3$)

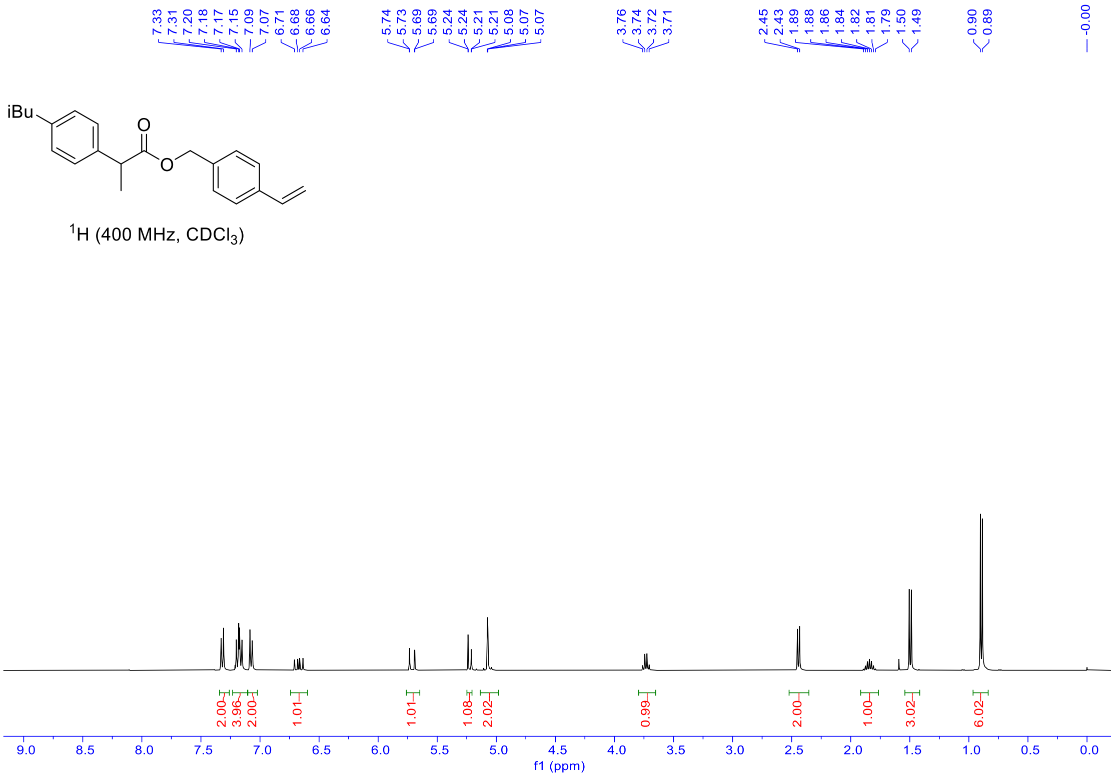

^{13}C NMR spectra of **b82** (101 MHz, $CDCl_3$)

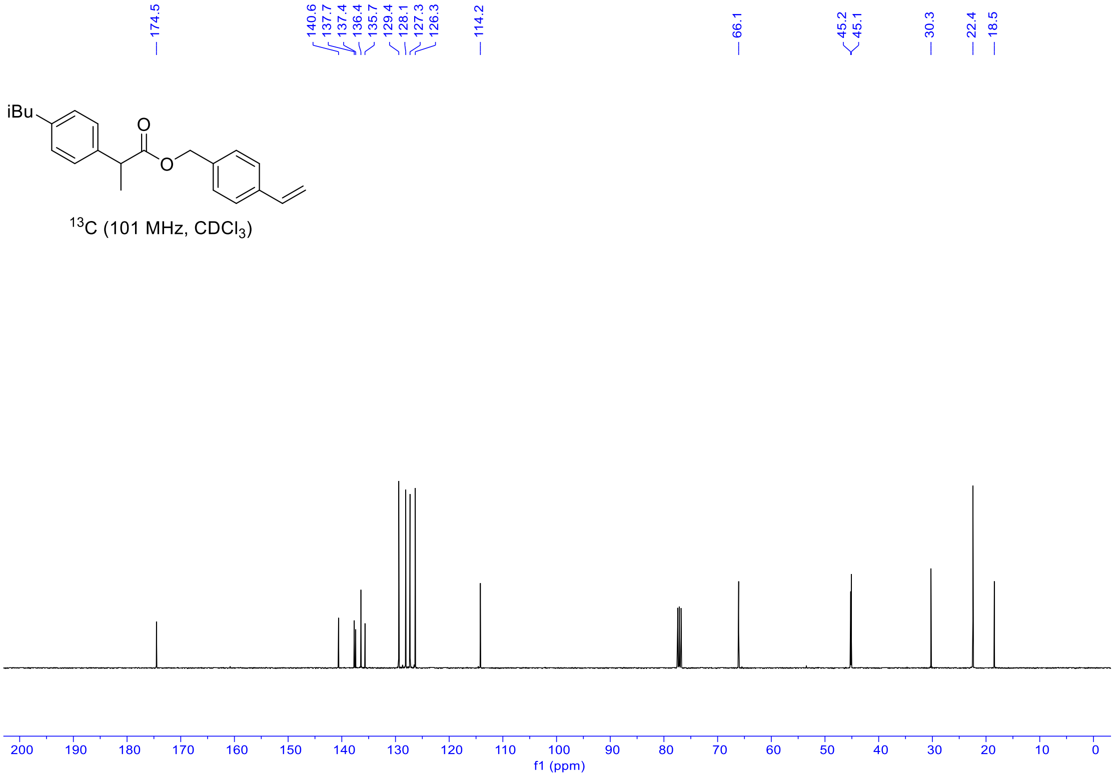

^{1}H NMR spectra of **1** (400 MHz, $CDCl_3$)

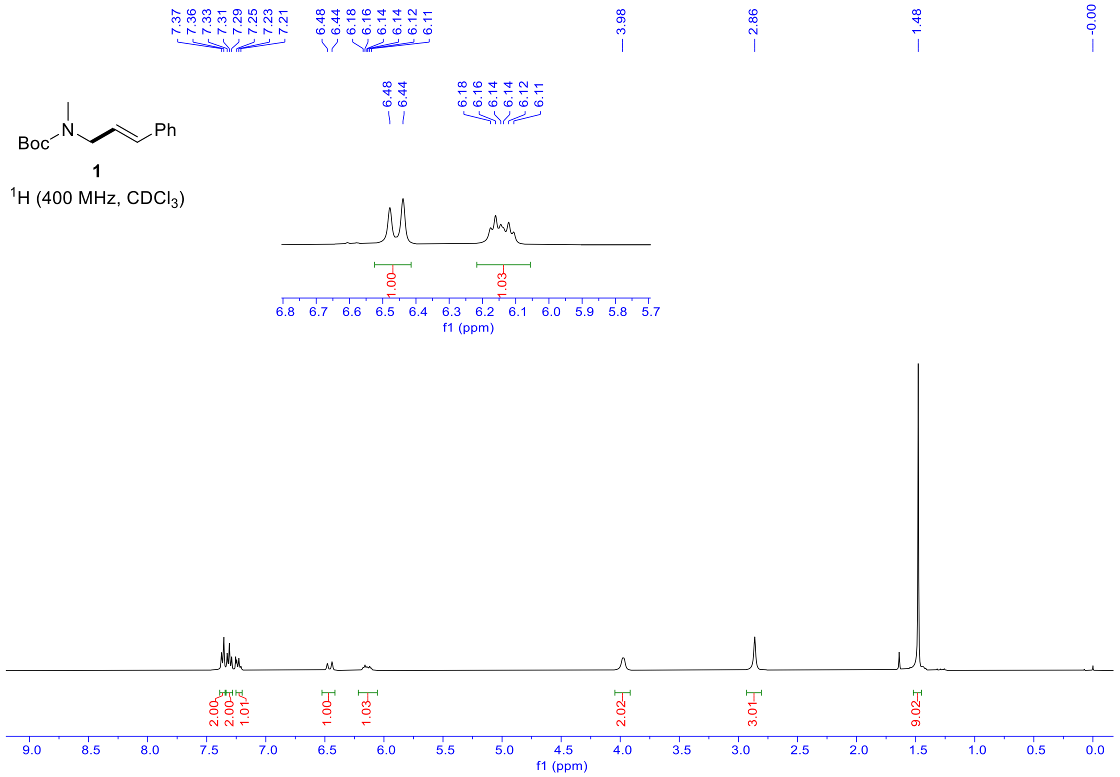

^{13}C NMR spectra of **1** (101 MHz, $CDCl_3$)

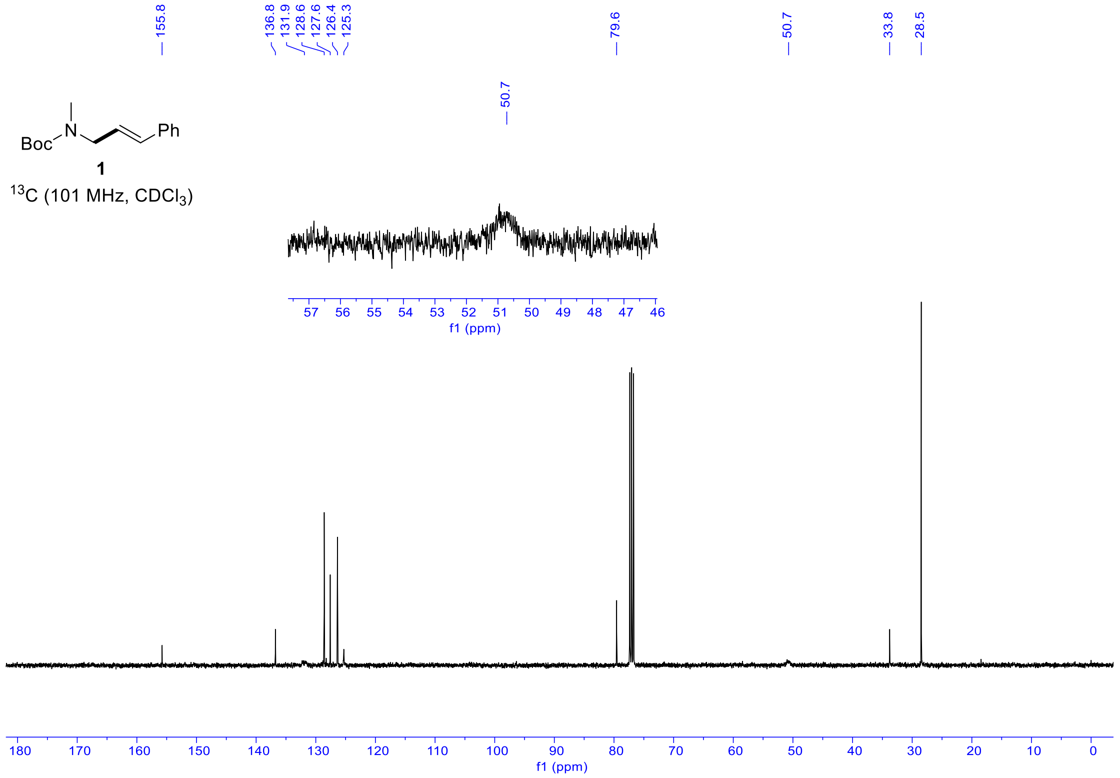

^{1}H NMR spectra of **2** (400 MHz, $CDCl_3$)

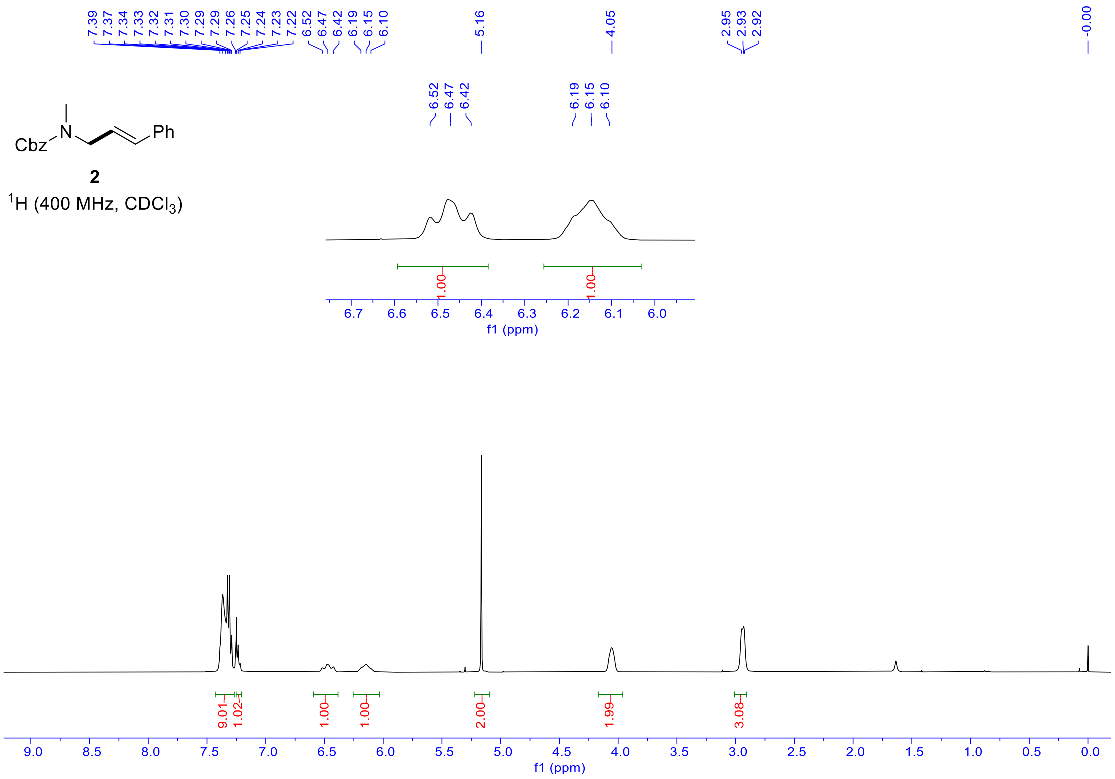

^{13}C NMR spectra of **2** (101 MHz, $CDCl_3$)

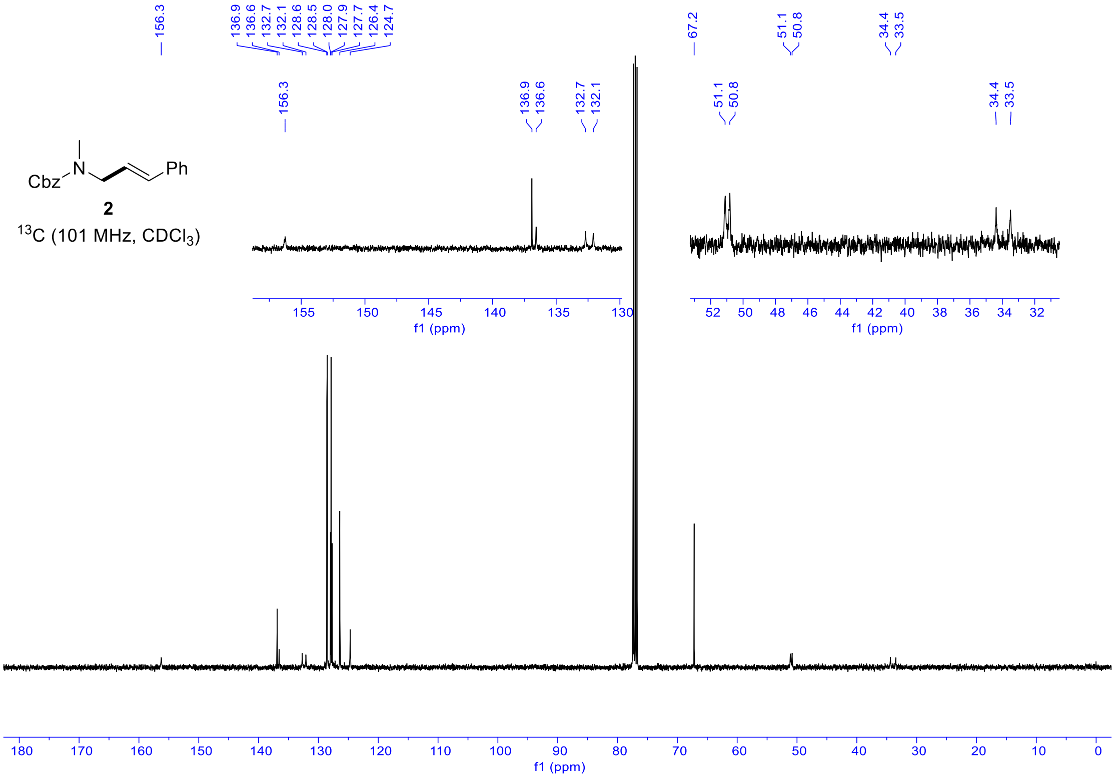

^{1}H NMR spectra of **3** (400 MHz, $CDCl_3$)

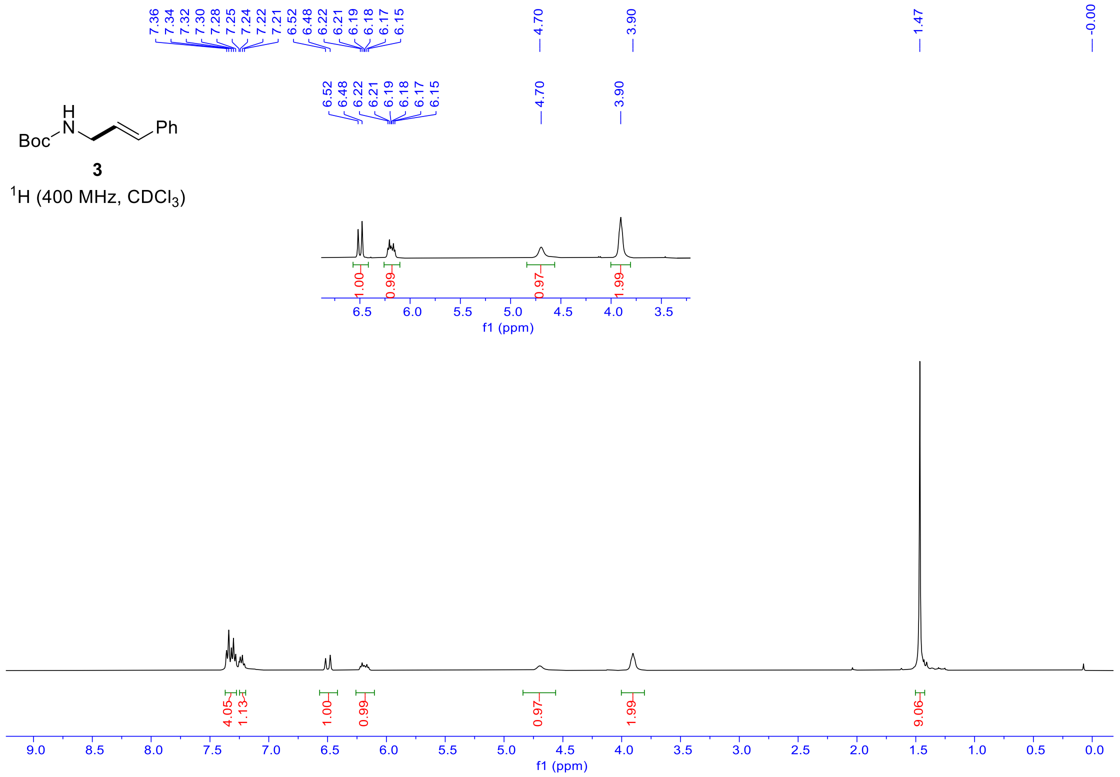

^{13}C NMR spectra of **3** (101 MHz, $CDCl_3$)

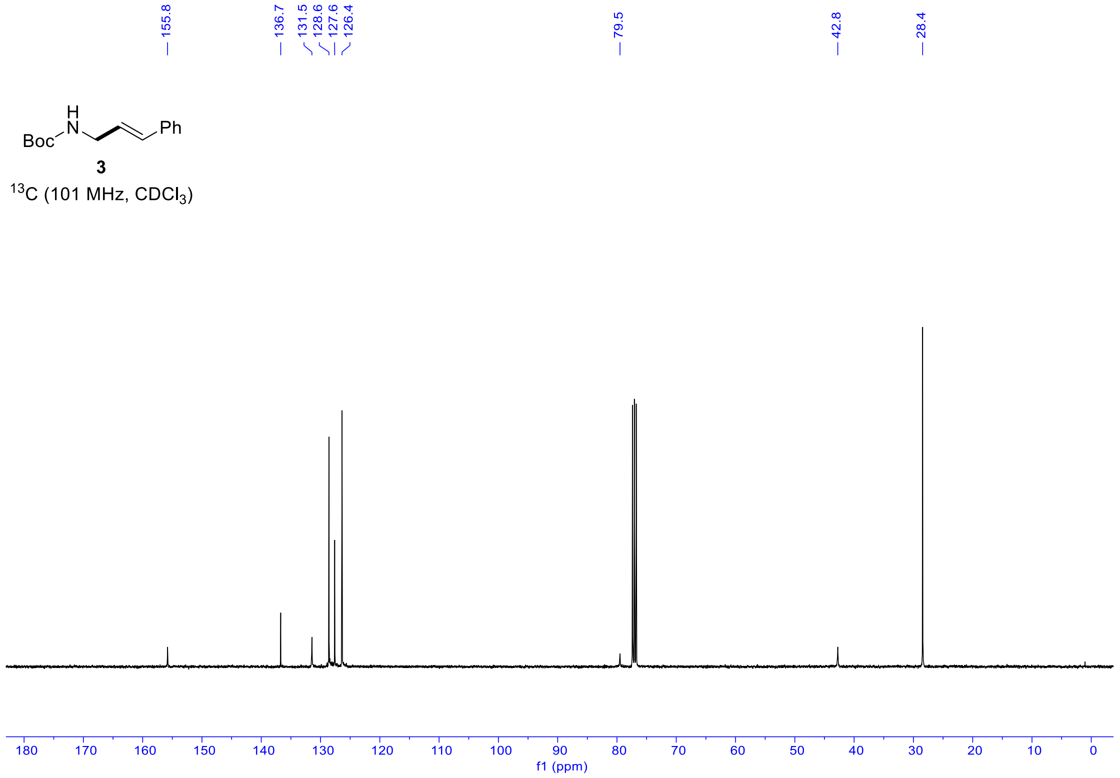

^{1}H NMR spectra of **4** (400 MHz, $CDCl_3$)

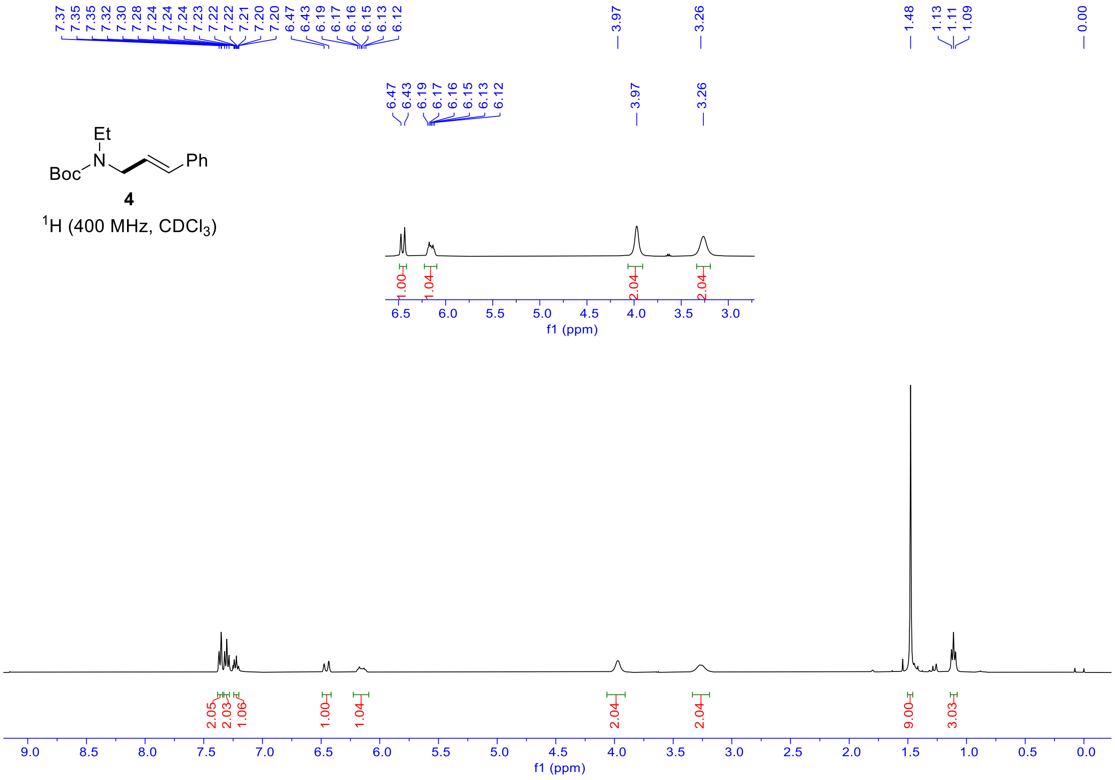

^{13}C NMR spectra of **4** (101 MHz, $CDCl_3$)

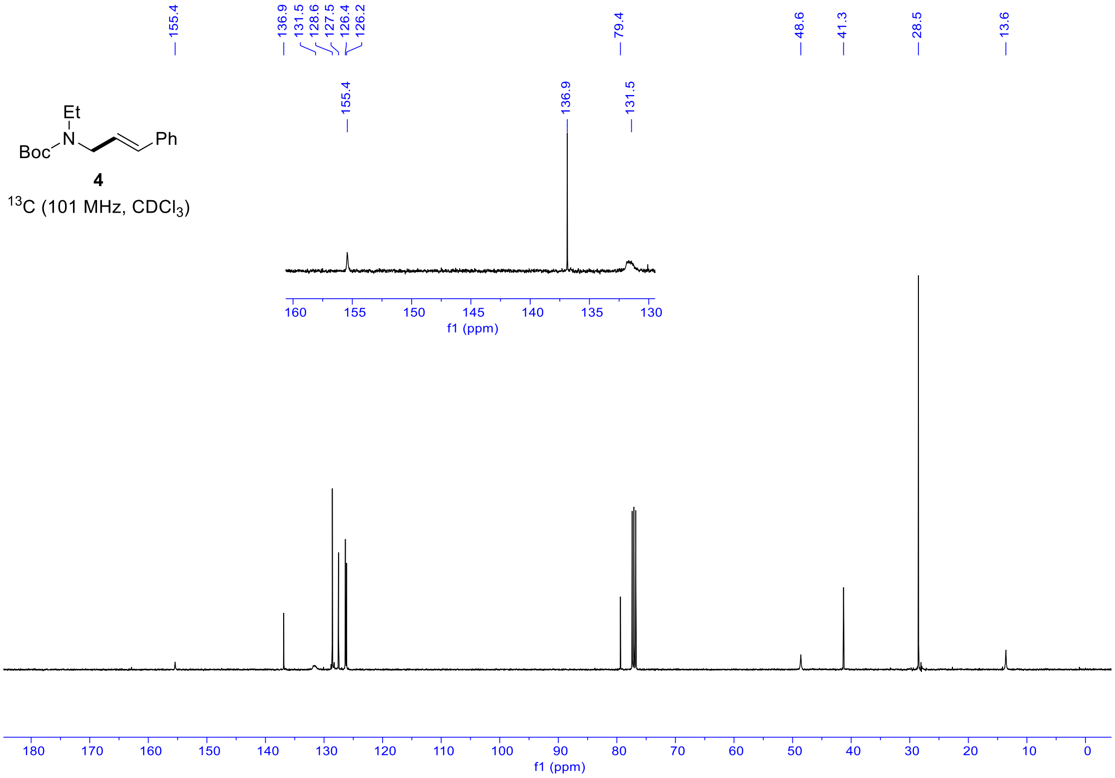

^{1}H NMR spectra of **5** (400 MHz, $CDCl_3$)

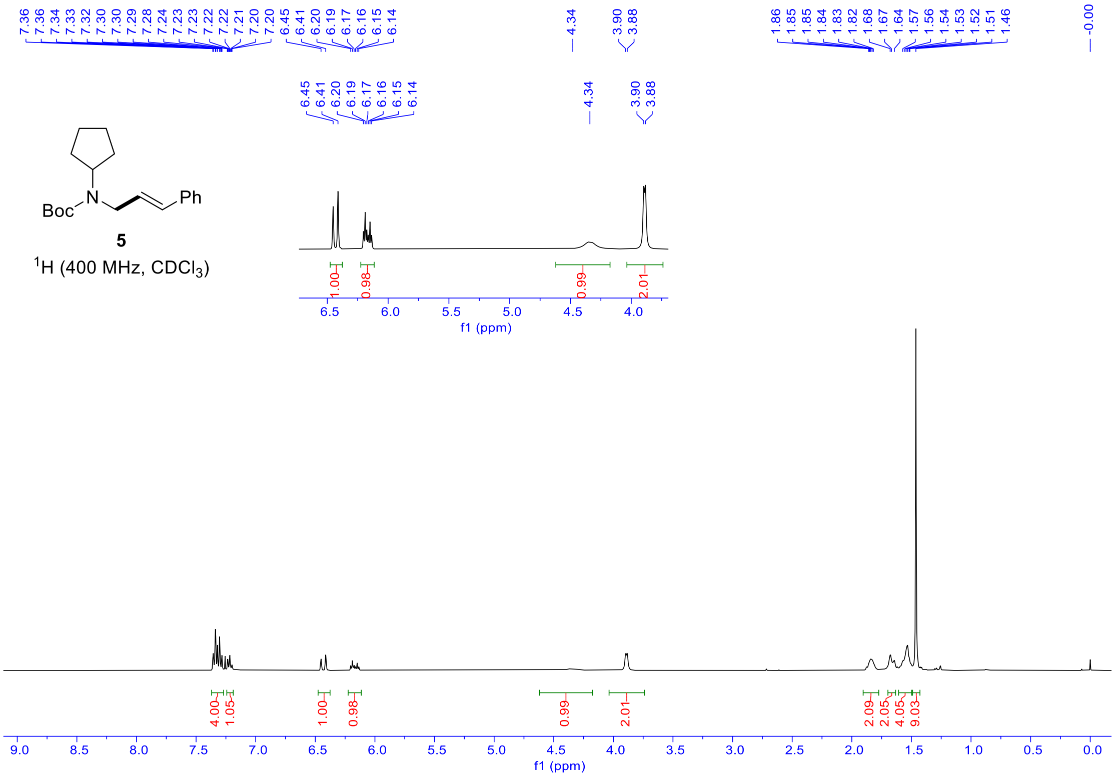

^{13}C NMR spectra of **5** (101 MHz, $CDCl_3$)

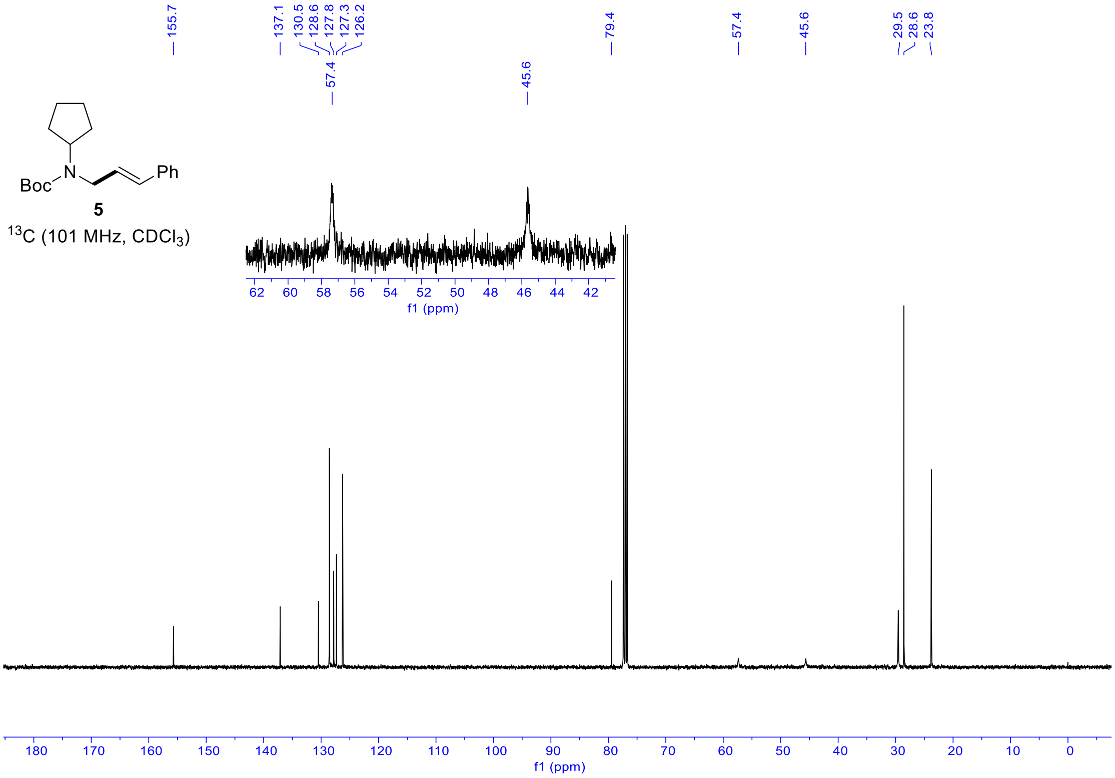

^{1}H NMR spectra of **6** (400 MHz, $CDCl_3$)

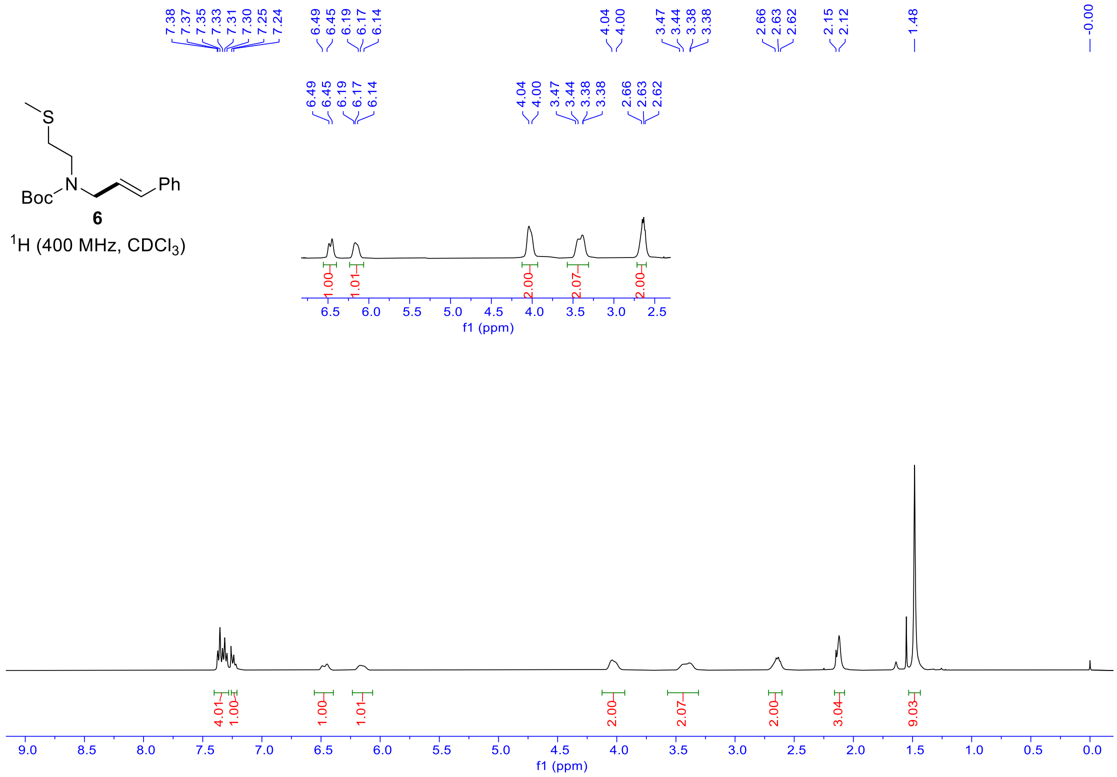

^{13}C NMR spectra of **6** (101 MHz, $CDCl_3$)

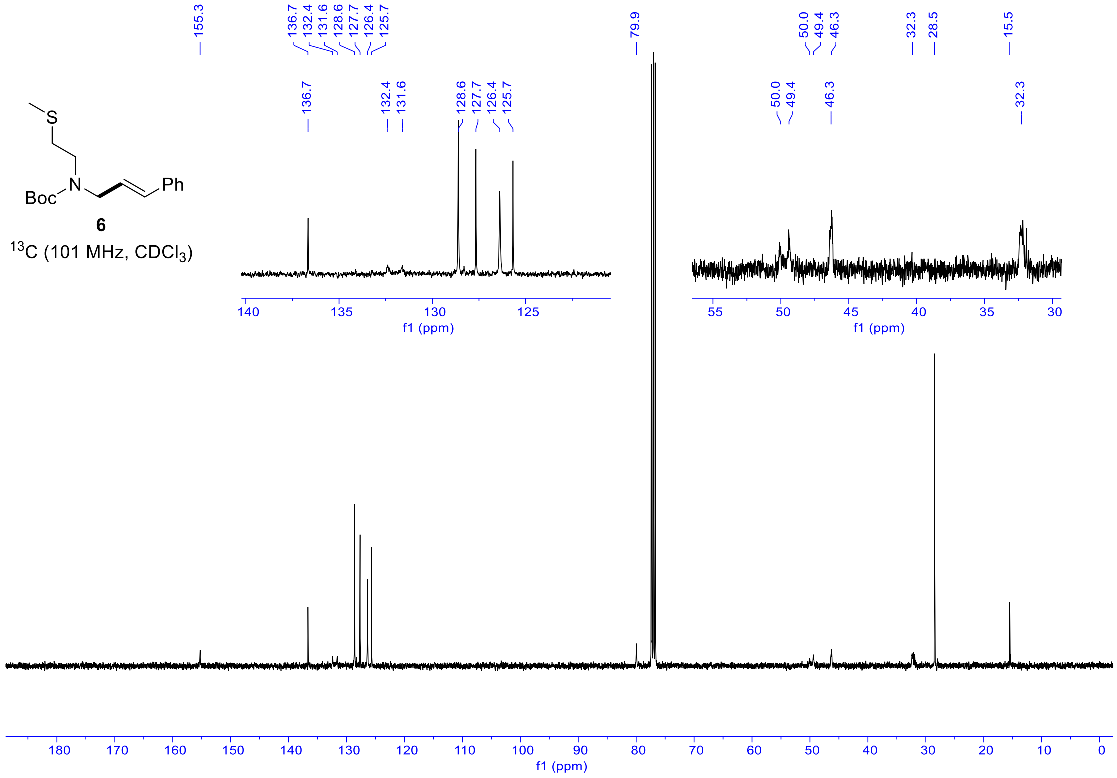

^{1}H NMR spectra of **7** (400 MHz, $CDCl_3$)

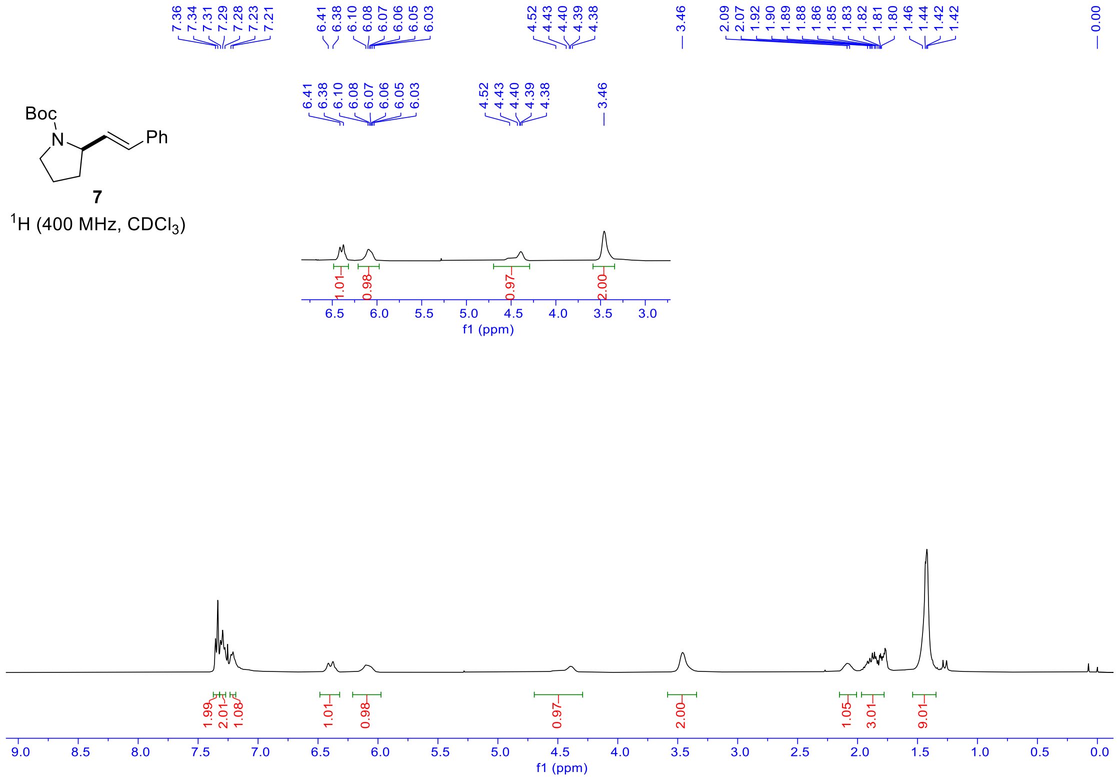

^{13}C NMR spectra of **7** (101 MHz, $CDCl_3$)

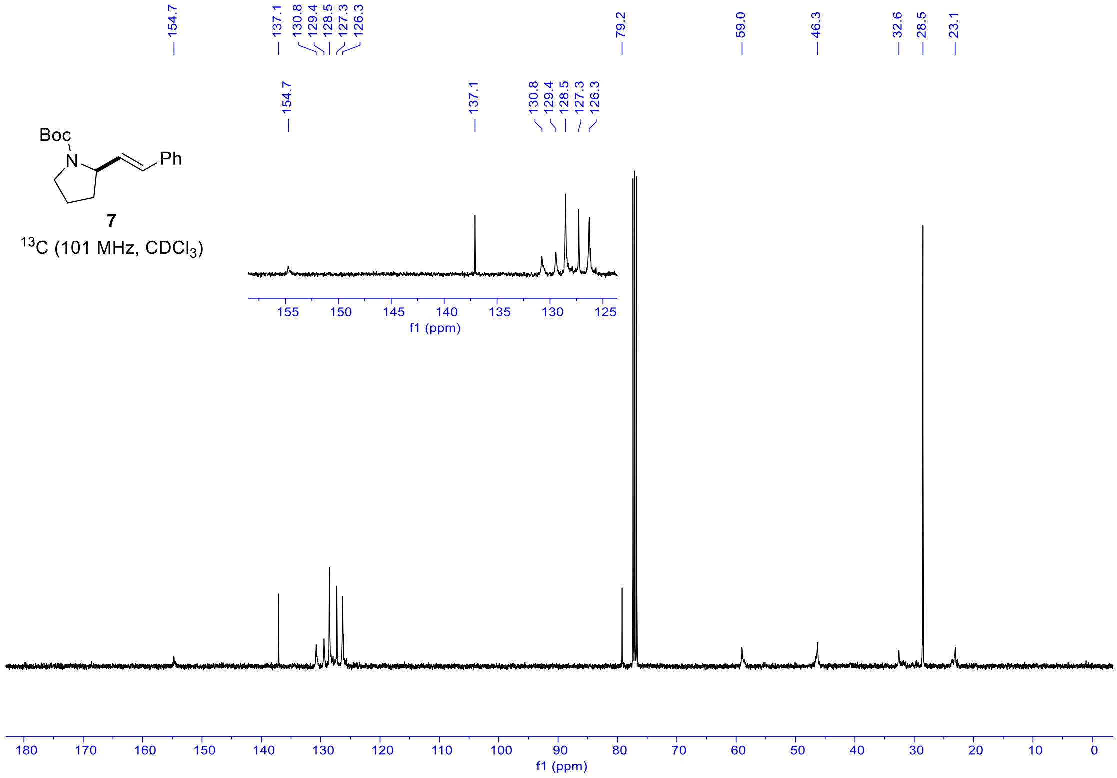

^{1}H NMR spectra of **8** (400 MHz, $CDCl_3$)

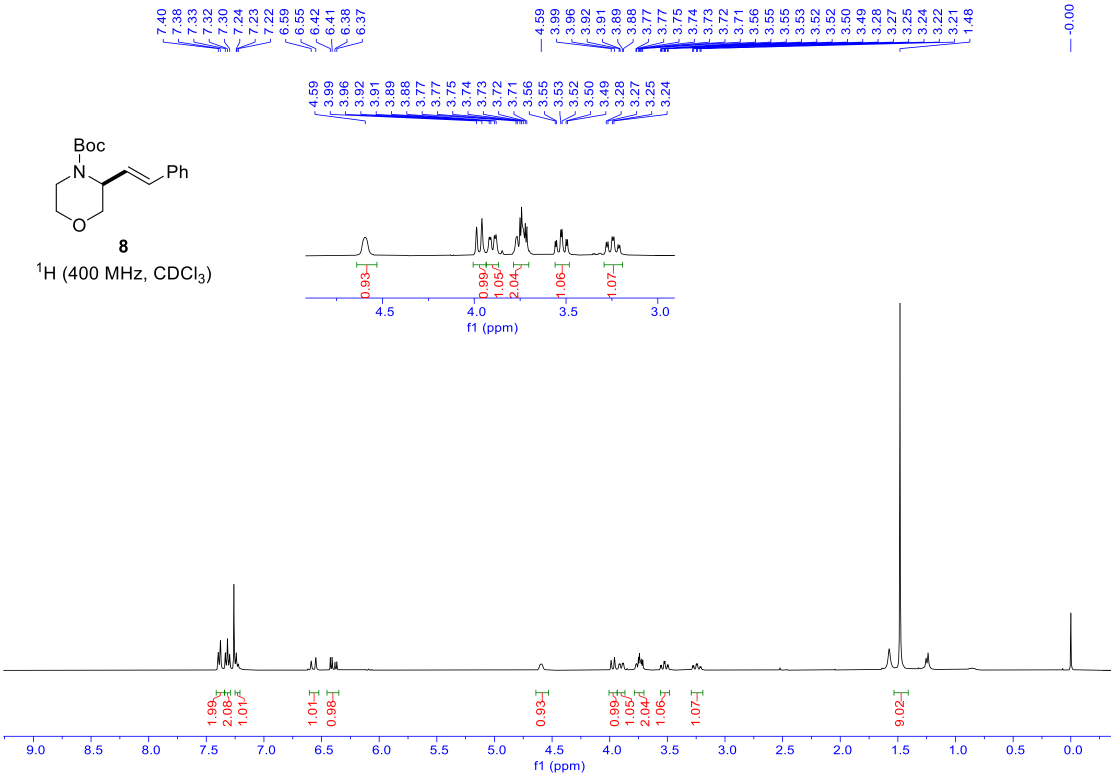

^{13}C NMR spectra of **8** (101 MHz, $CDCl_3$)

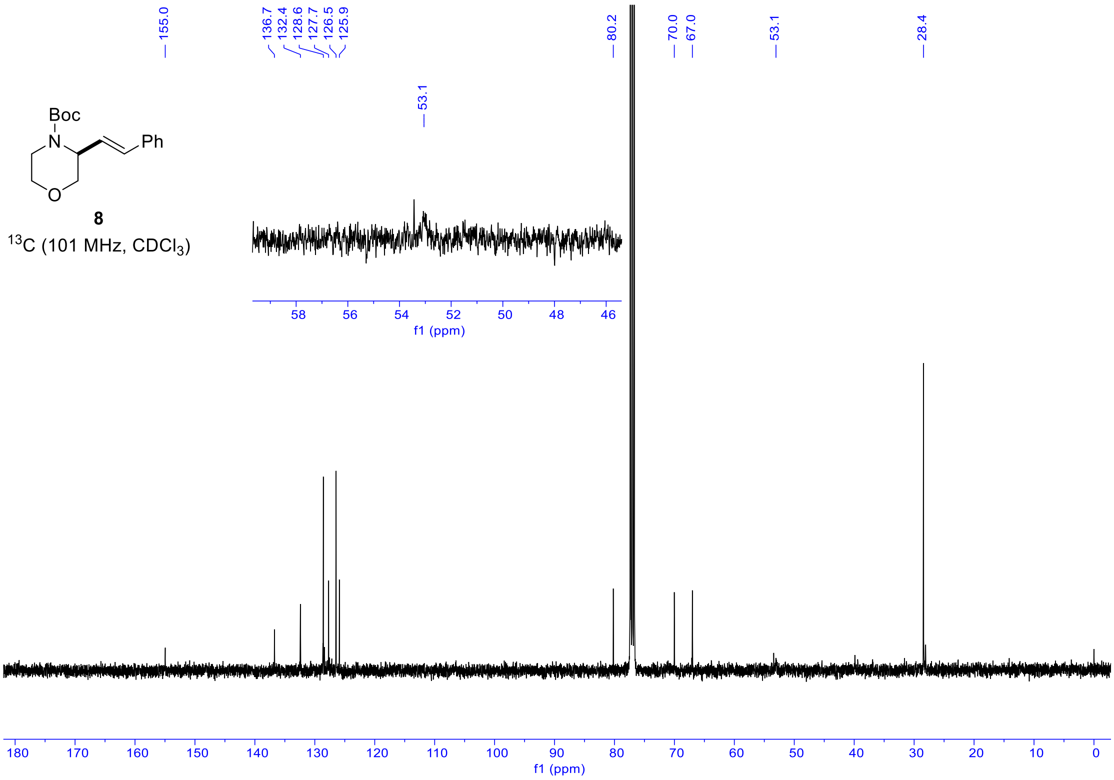

^{1}H NMR spectra of **9** (400 MHz, $CDCl_3$)

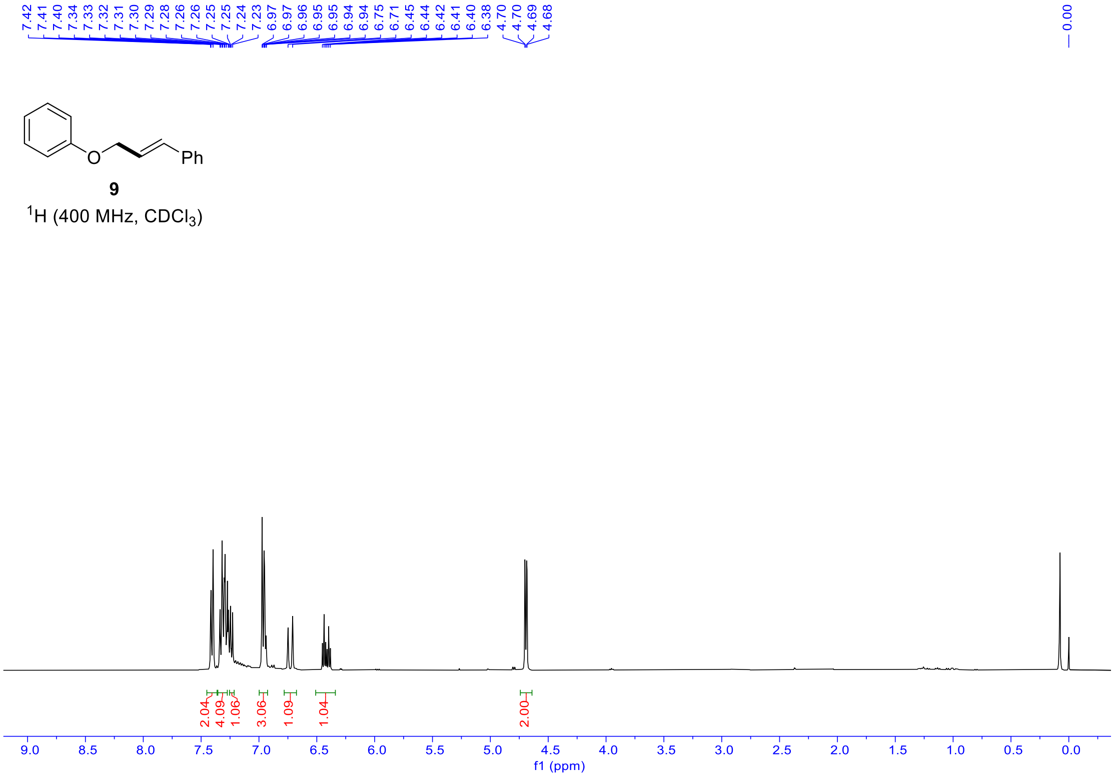

^{13}C NMR spectra of **9** (101 MHz, $CDCl_3$)

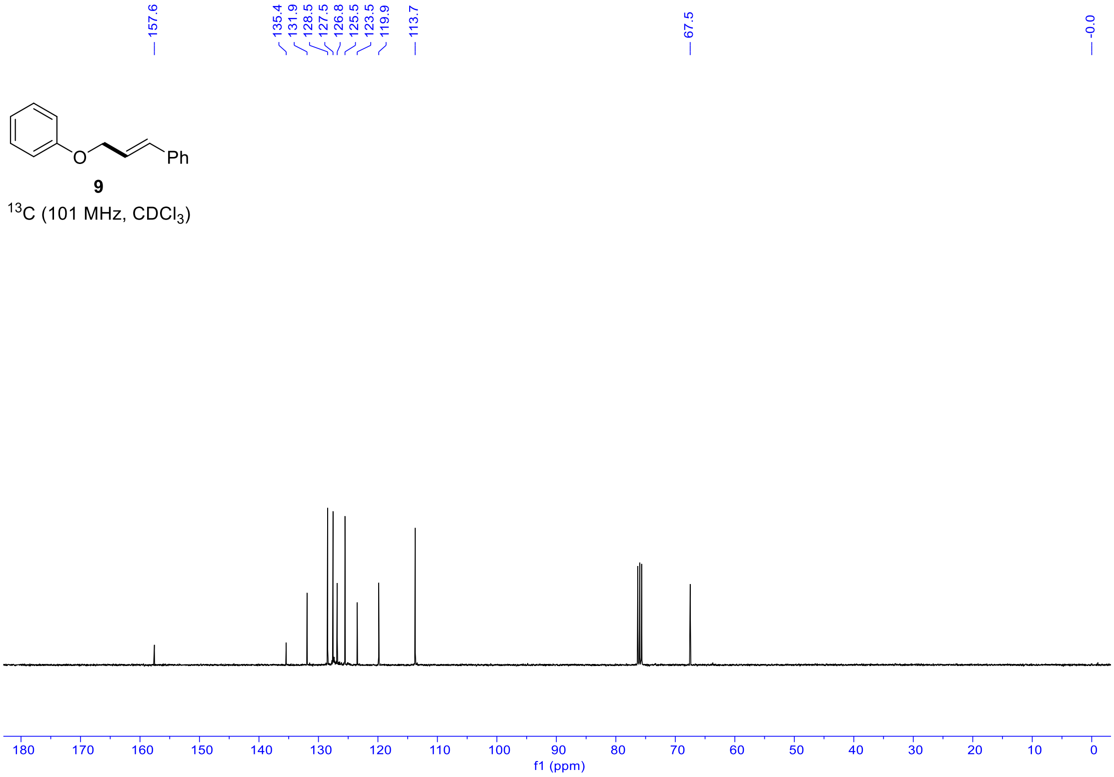

^{1}H NMR spectra of **10** (400 MHz, $CDCl_3$)

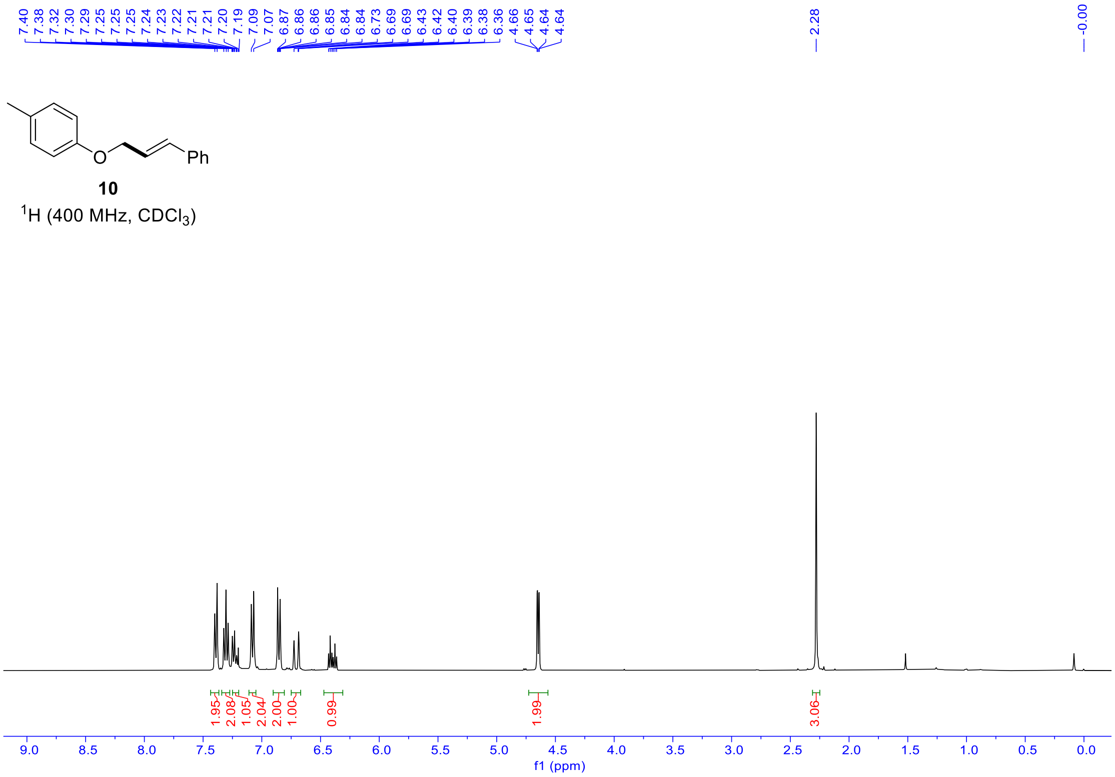

^{13}C NMR spectra of **10** (101 MHz, $CDCl_3$)

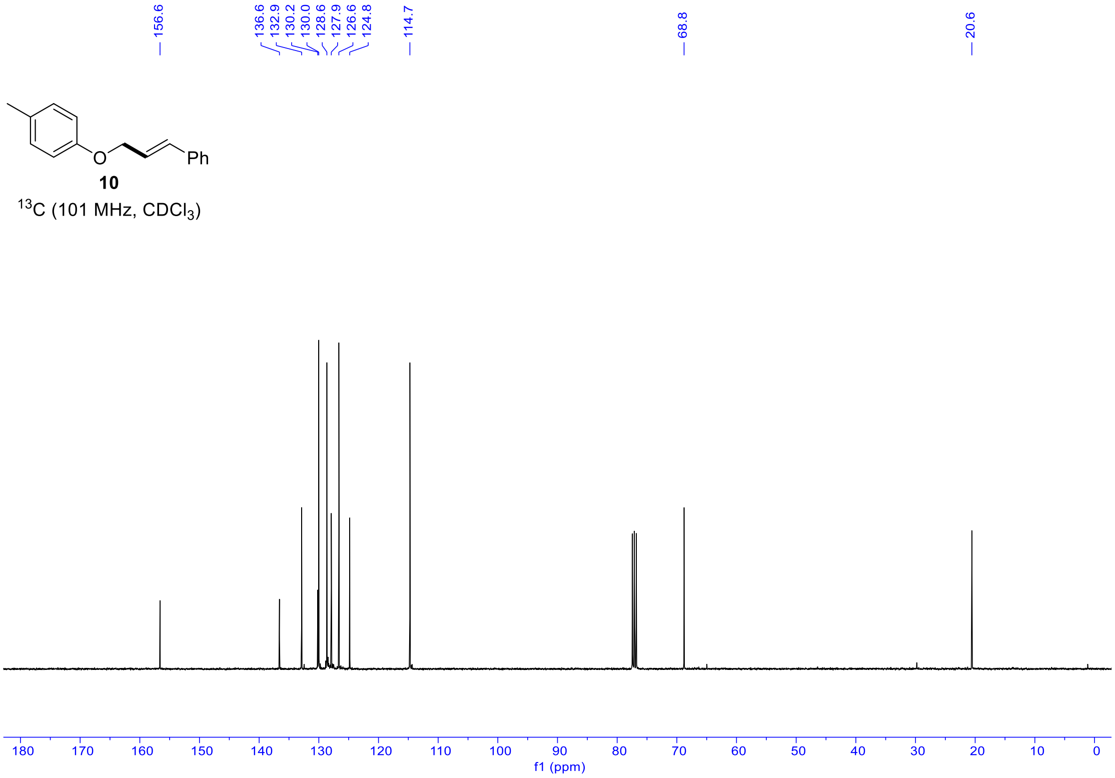

^{1}H NMR spectra of **11** (400 MHz, $CDCl_3$)

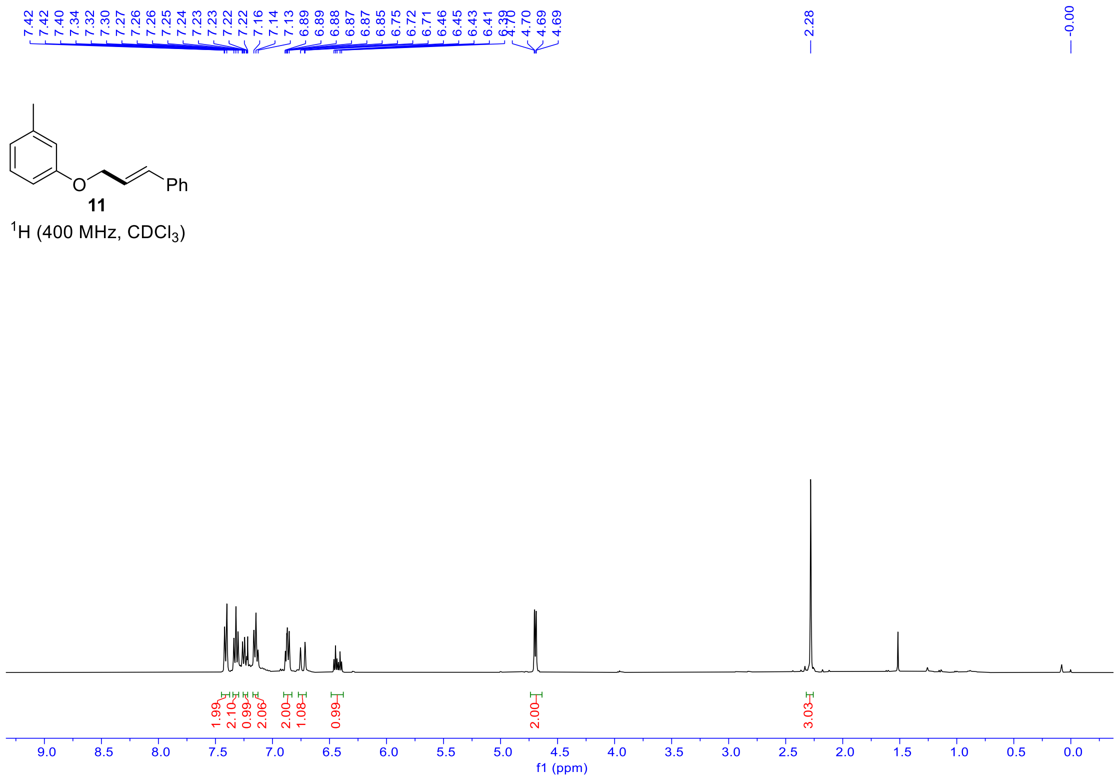

^{13}C NMR spectra of **11** (101 MHz, $CDCl_3$)

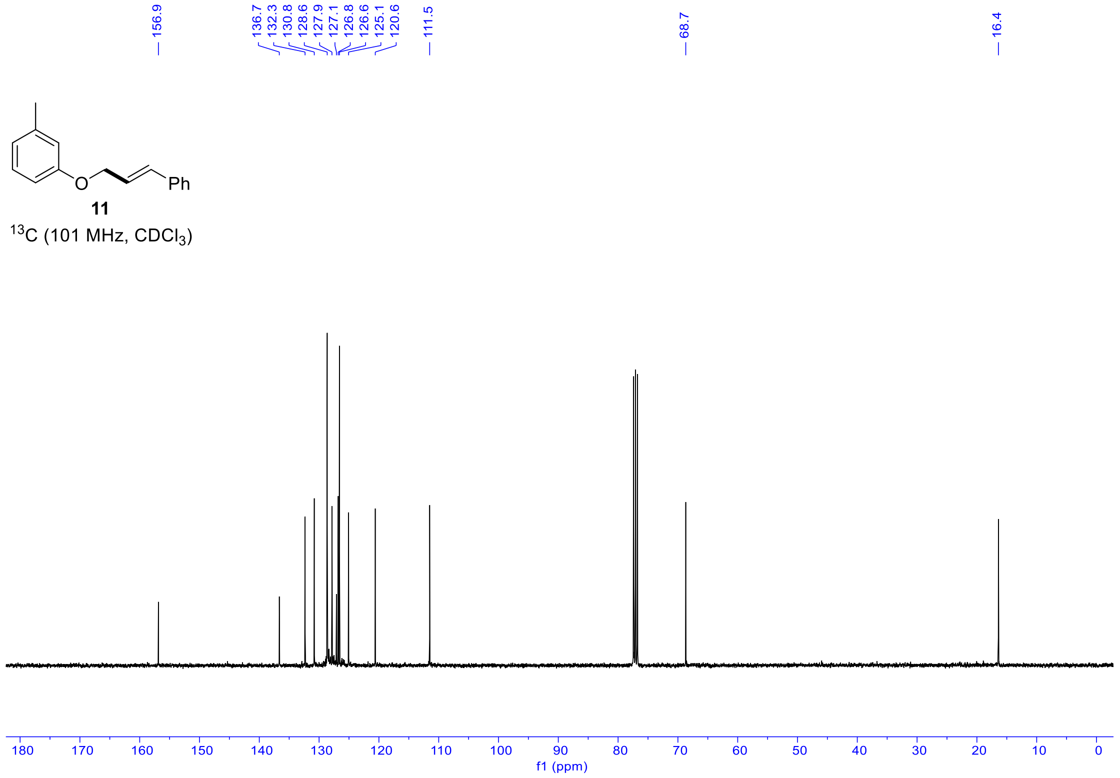

^{1}H NMR spectra of **12** (400 MHz, $CDCl_3$)

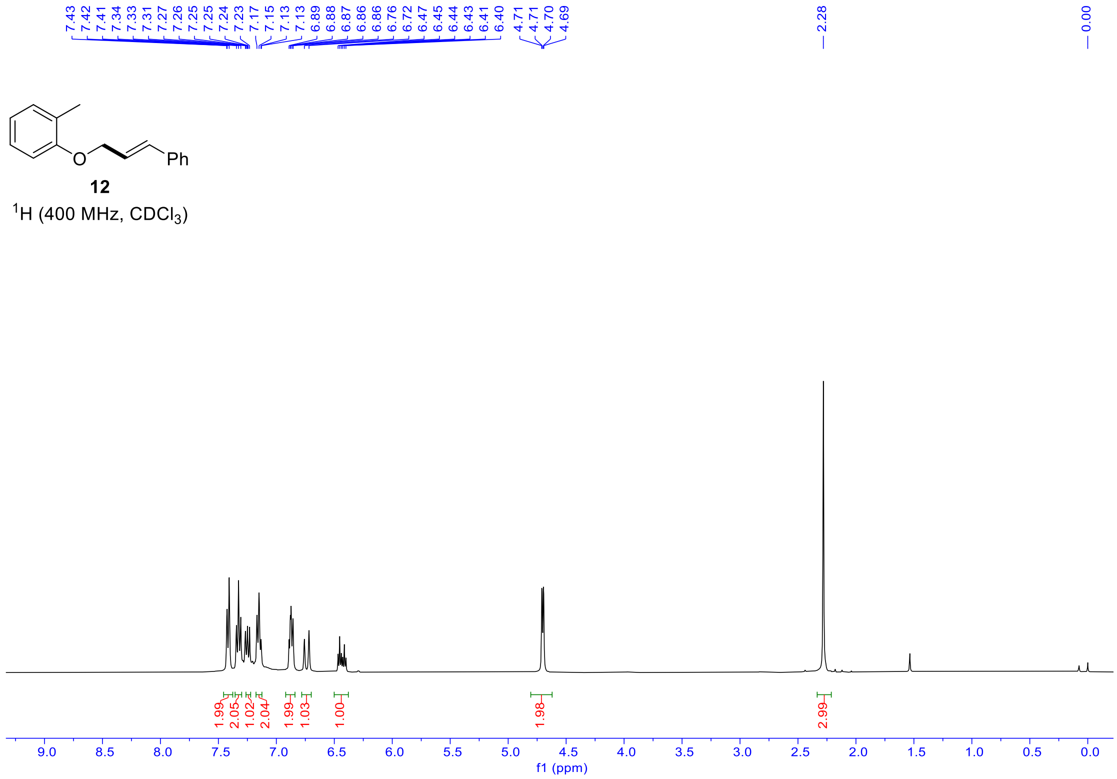

^{13}C NMR spectra of **12** (101 MHz, $CDCl_3$)

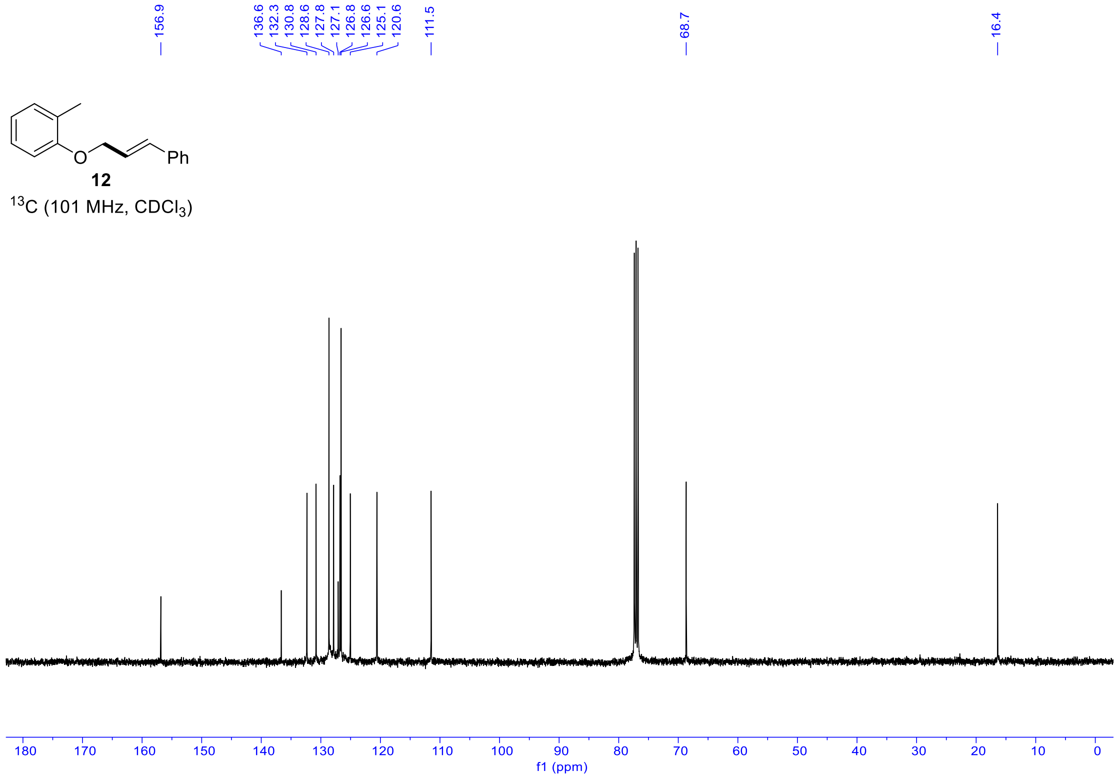

^{1}H NMR spectra of **13** (400 MHz, $CDCl_3$)

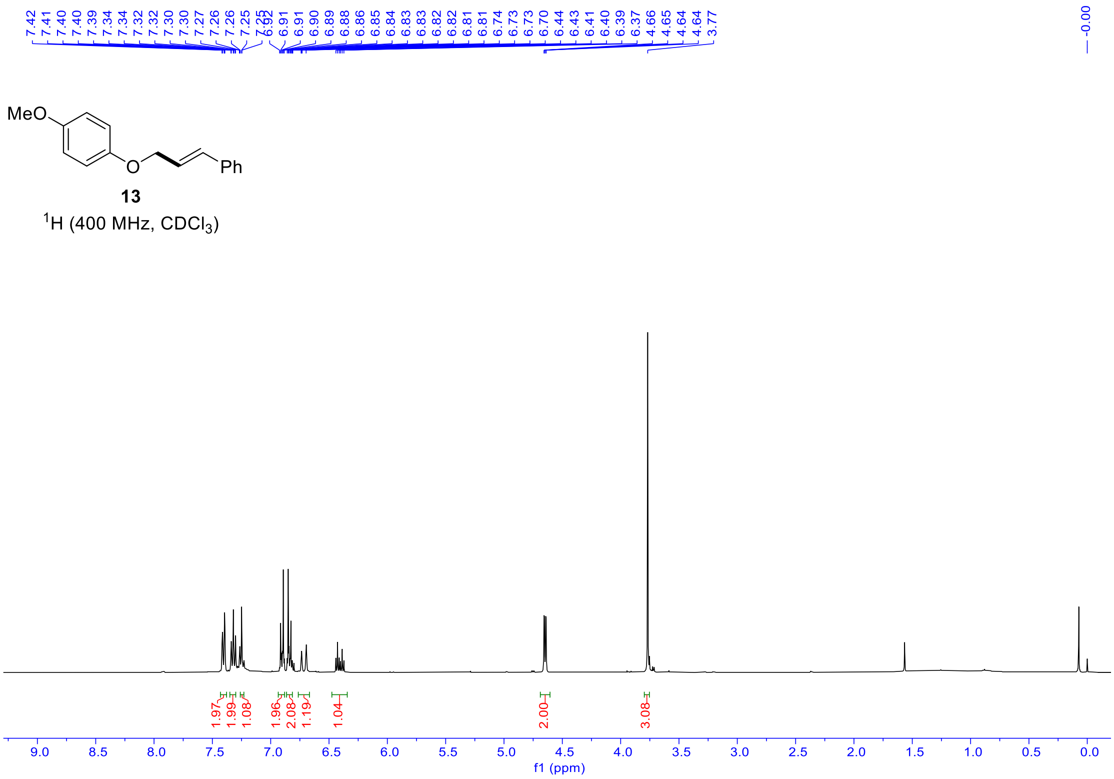

^{13}C NMR spectra of **13** (101 MHz, $CDCl_3$)

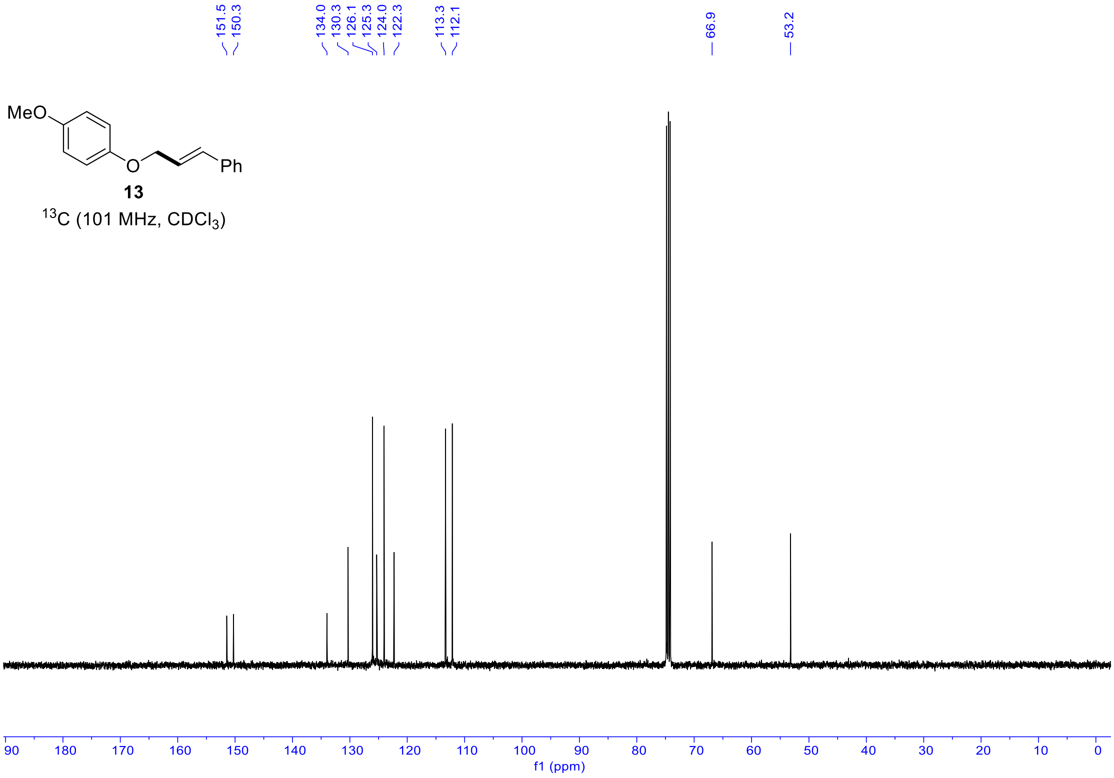

^{1}H NMR spectra of **14** (400 MHz, $CDCl_3$)

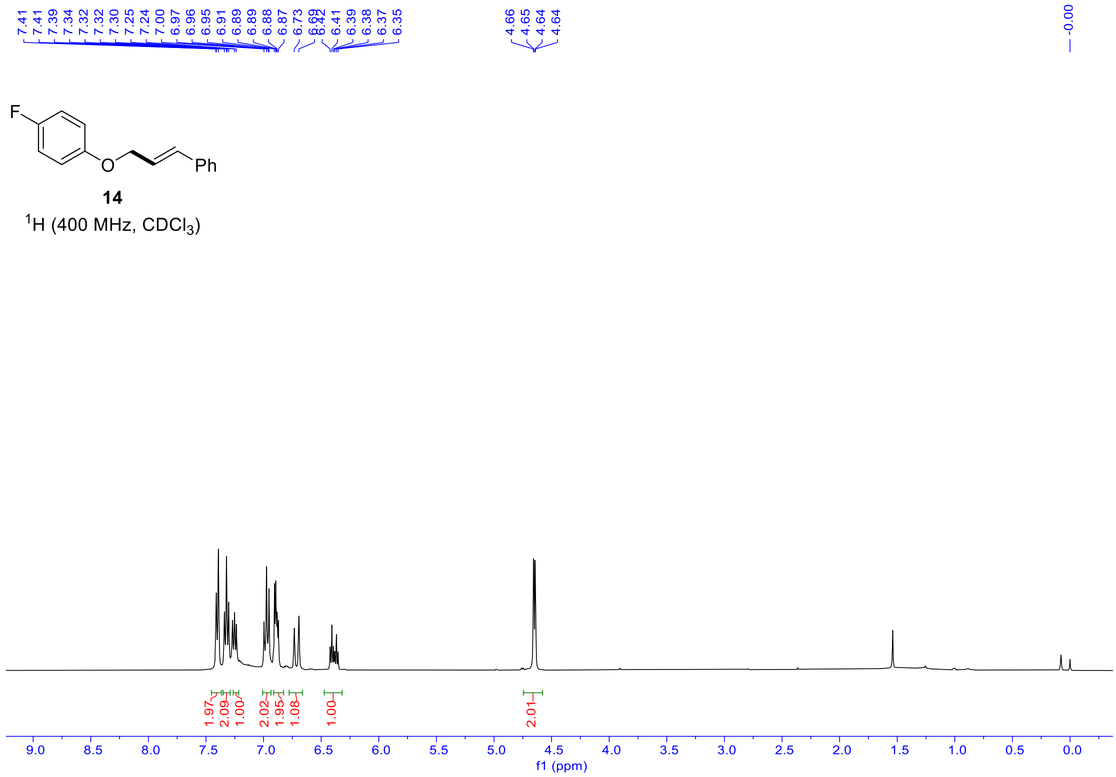

^{13}C NMR spectra of **14** (101 MHz, $CDCl_3$)

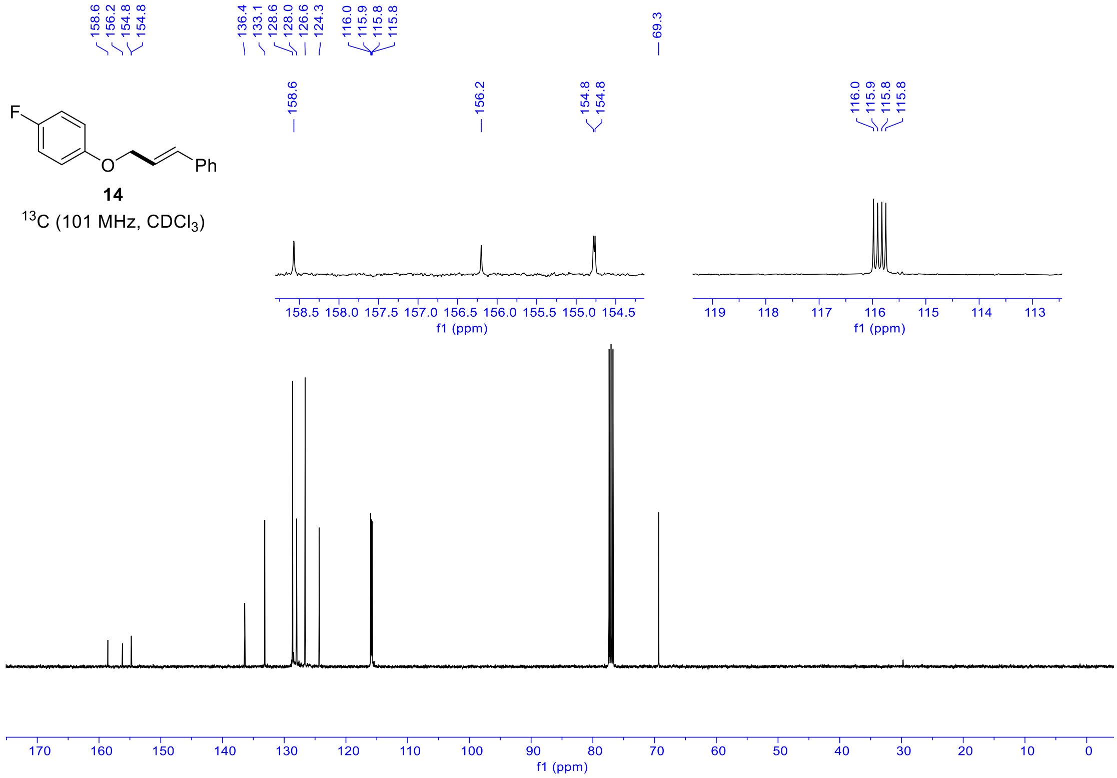

^{19}F NMR spectra of **14** (377 MHz, $CDCl_3$)

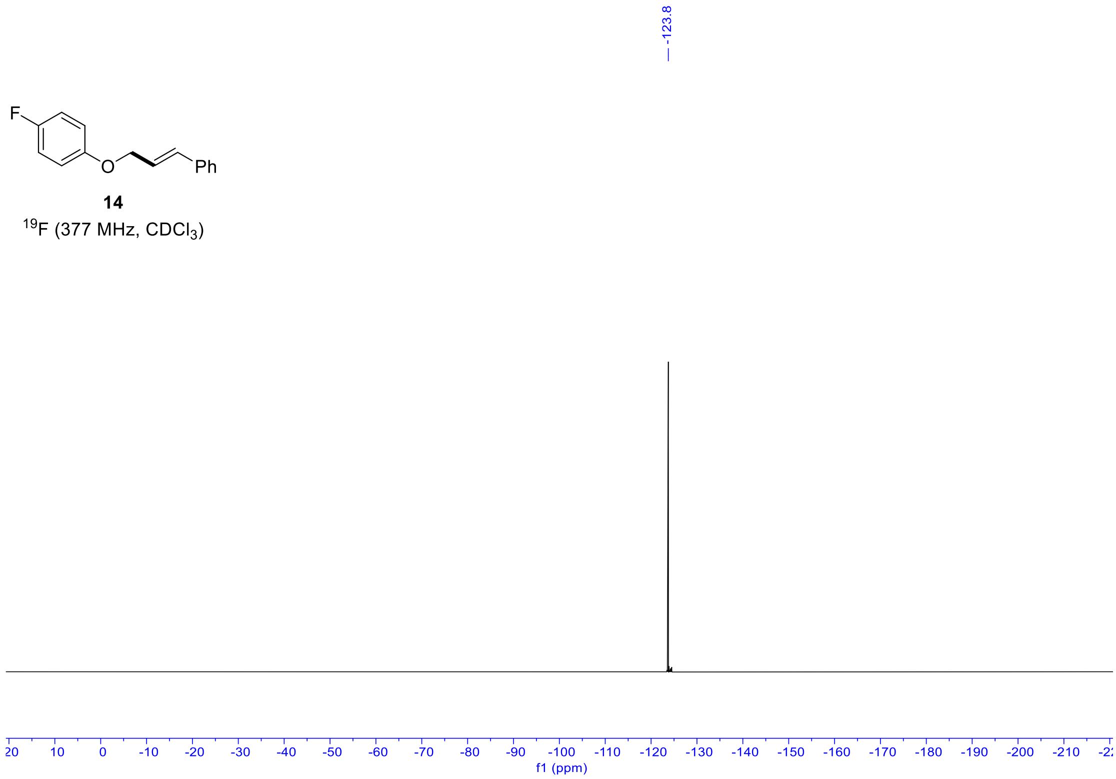

^{1}H NMR spectra of **15** (400 MHz, $CDCl_3$)

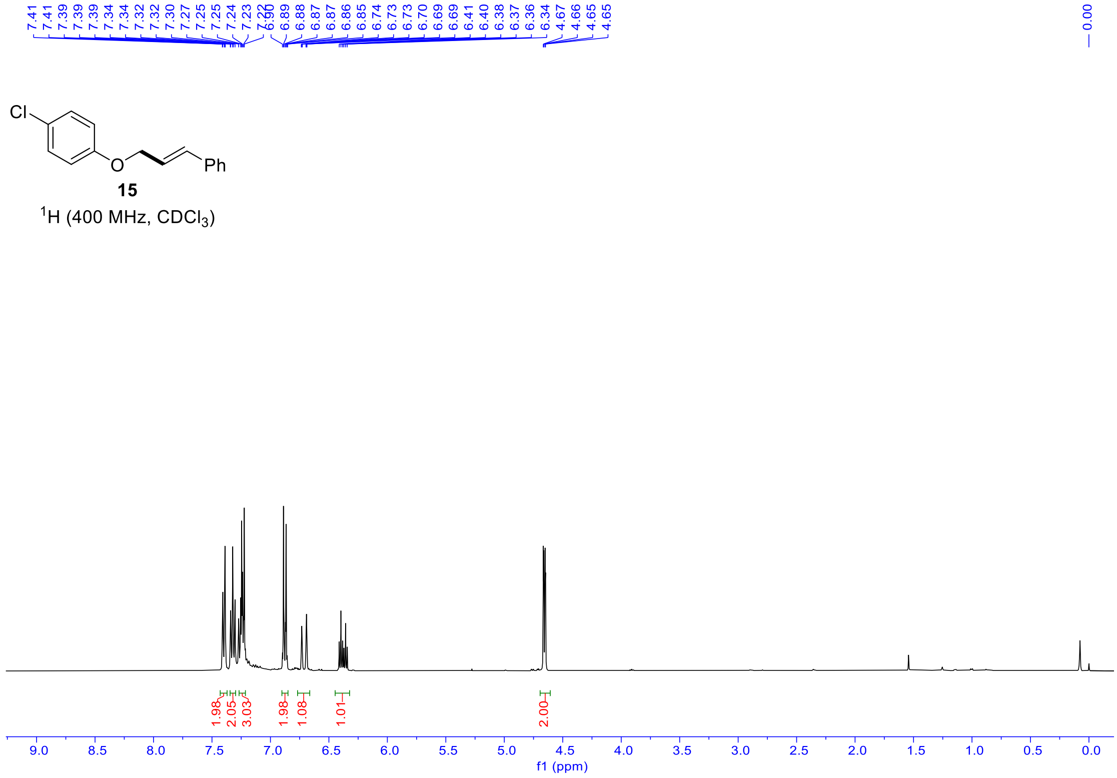

^{13}C NMR spectra of **15** (101 MHz, $CDCl_3$)

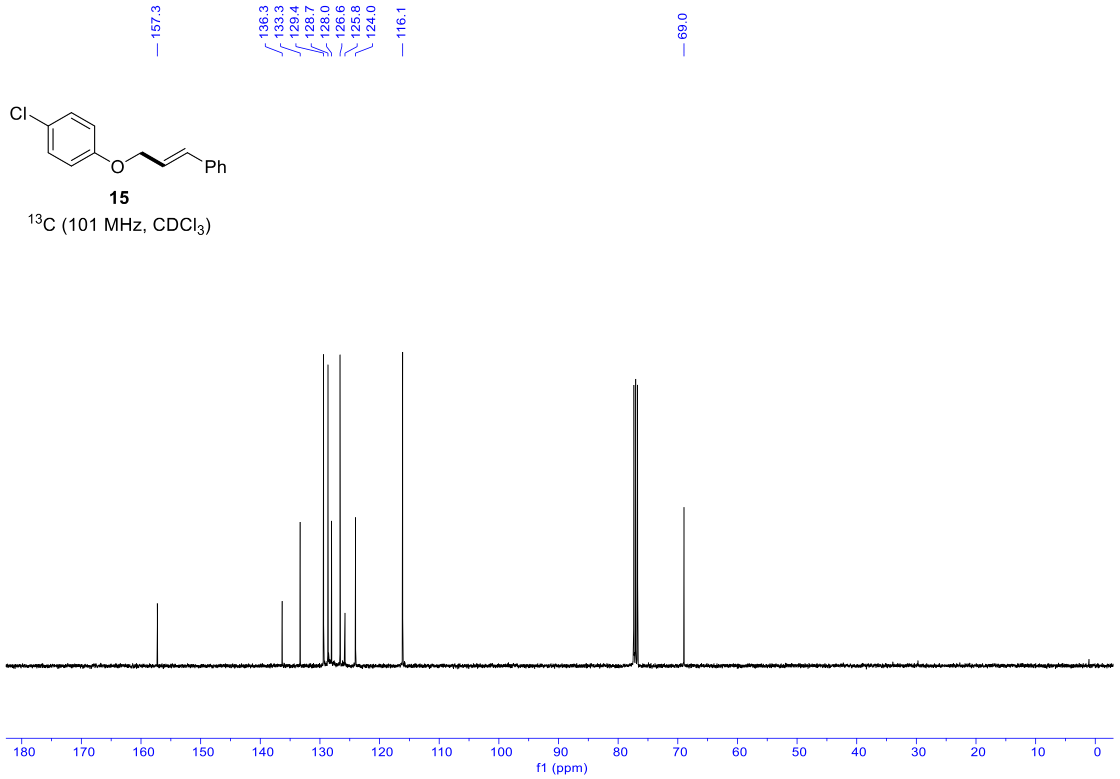

^{1}H NMR spectra of **16** (400 MHz, $CDCl_3$)

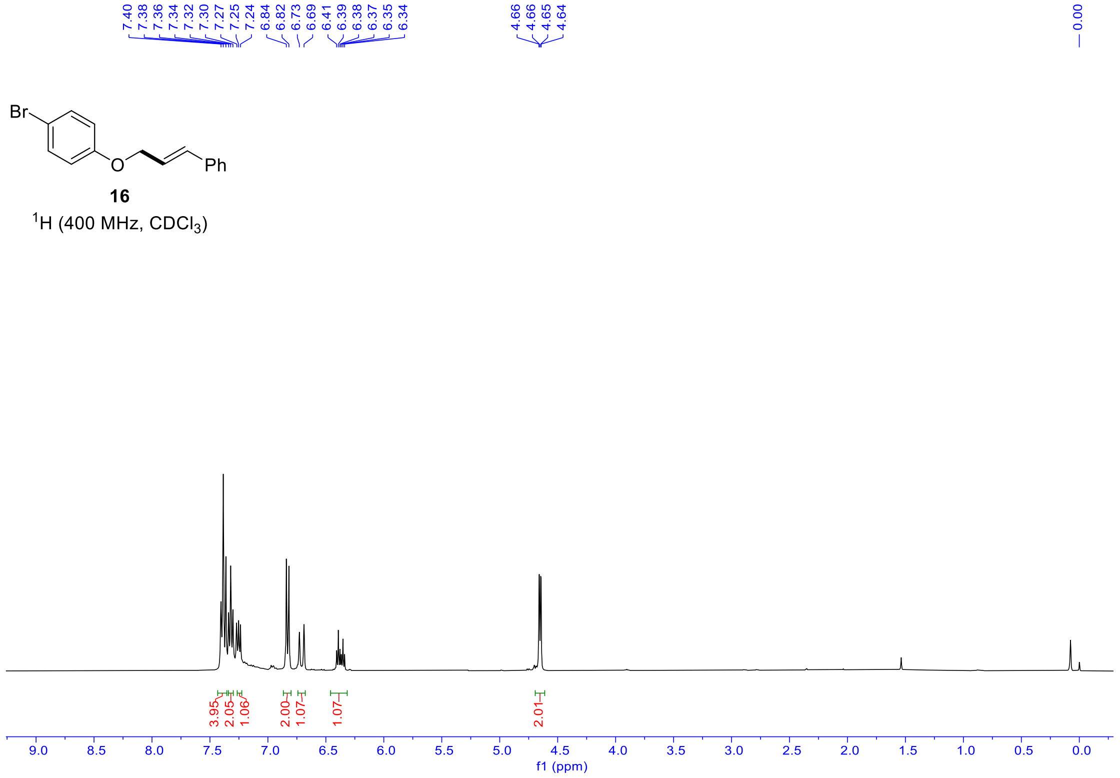

^{13}C NMR spectra of **16** (101 MHz, $CDCl_3$)

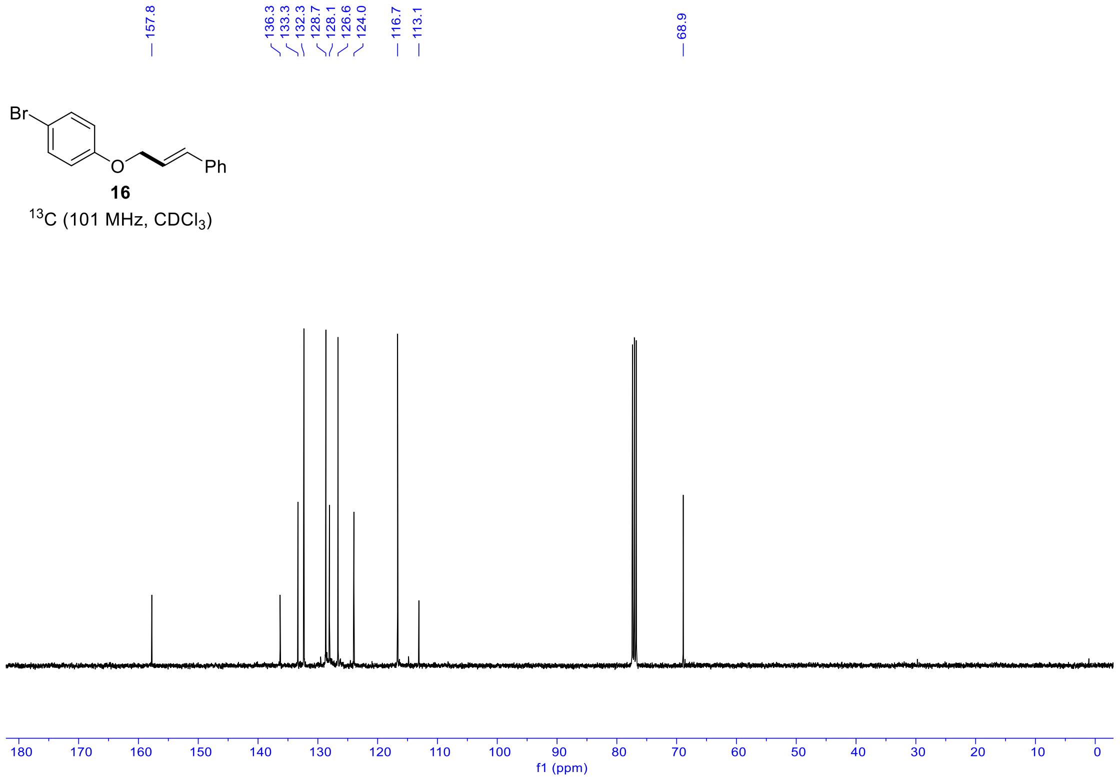

^{1}H NMR spectra of **17** (400 MHz, $CDCl_3$)

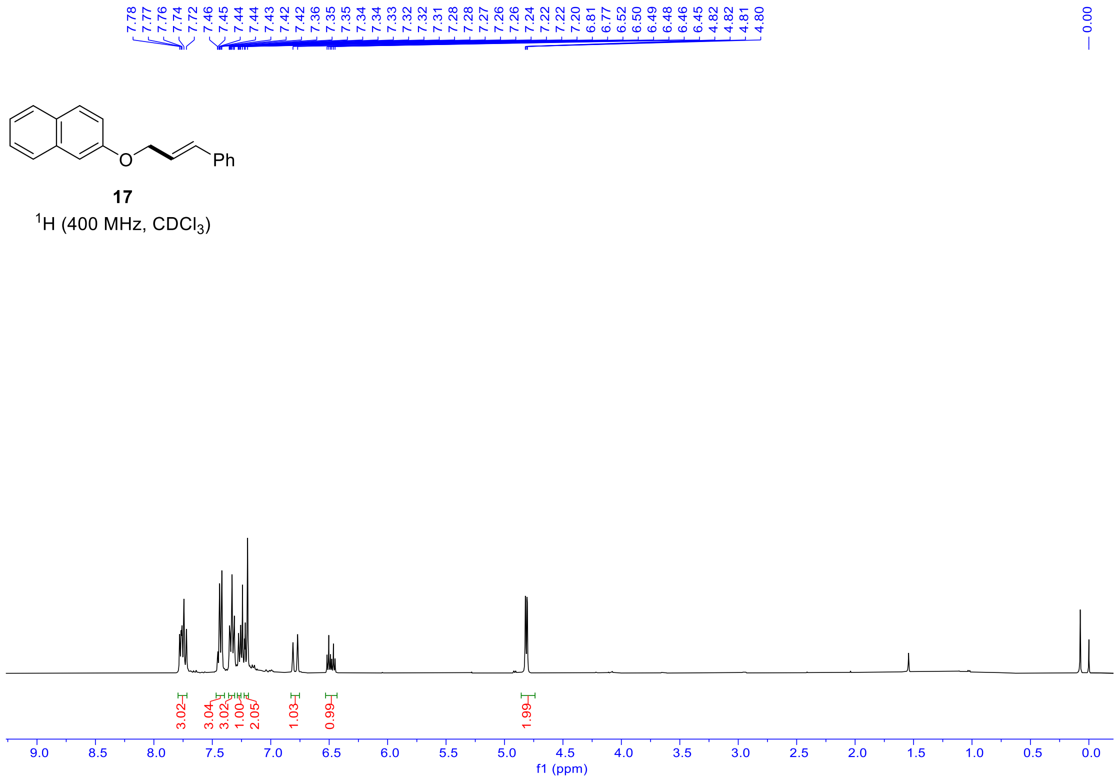

^{13}C NMR spectra of **17** (101 MHz, $CDCl_3$)

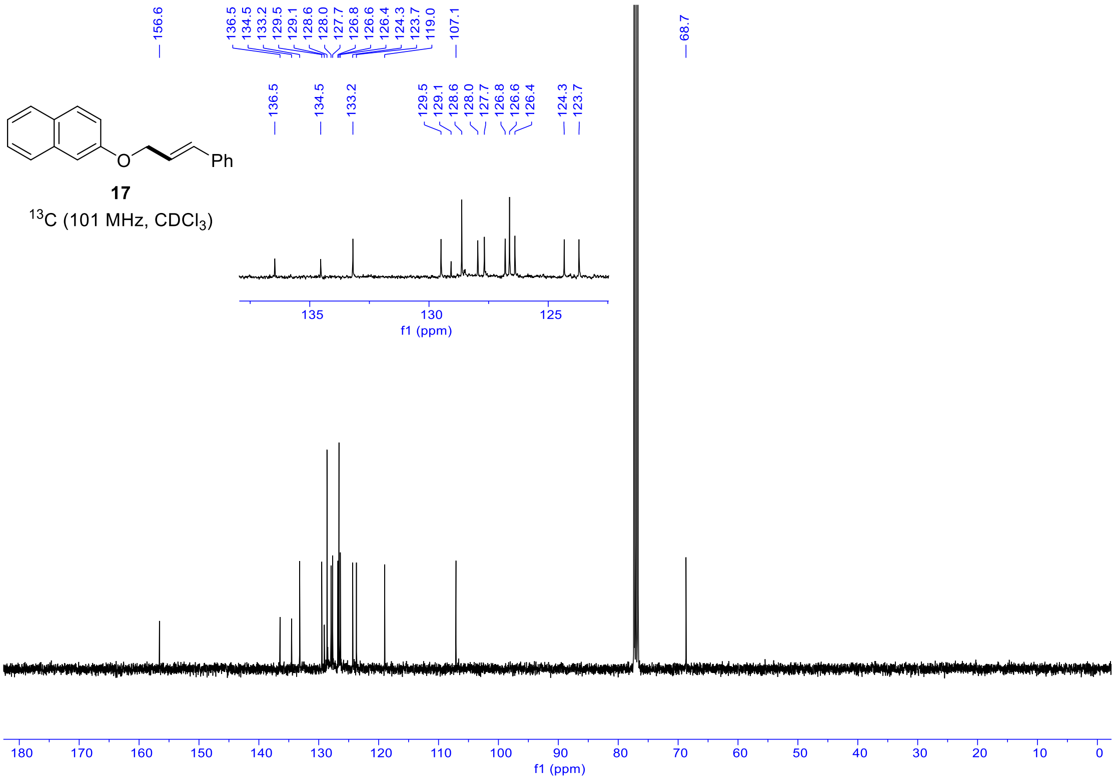

^{1}H NMR spectra of **18** (400 MHz, $CDCl_3$)

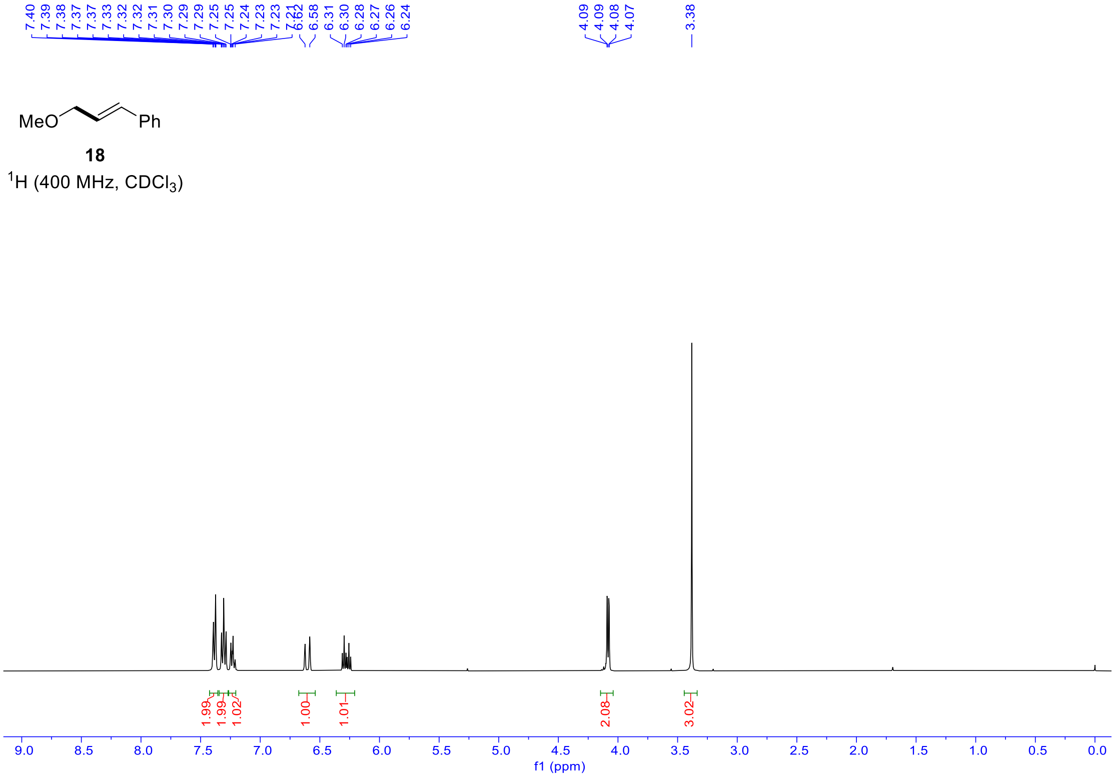

^{13}C NMR spectra of **18** (101 MHz, $CDCl_3$)

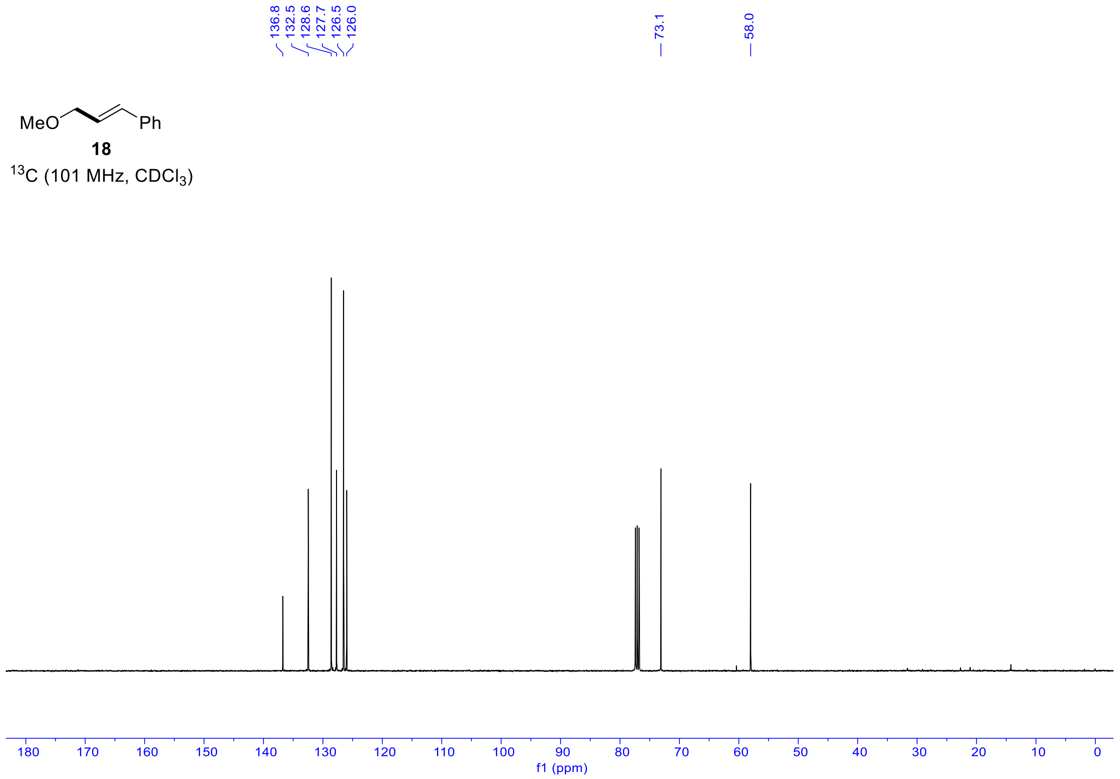

^{1}H NMR spectra of **19** (400 MHz, $CDCl_3$)

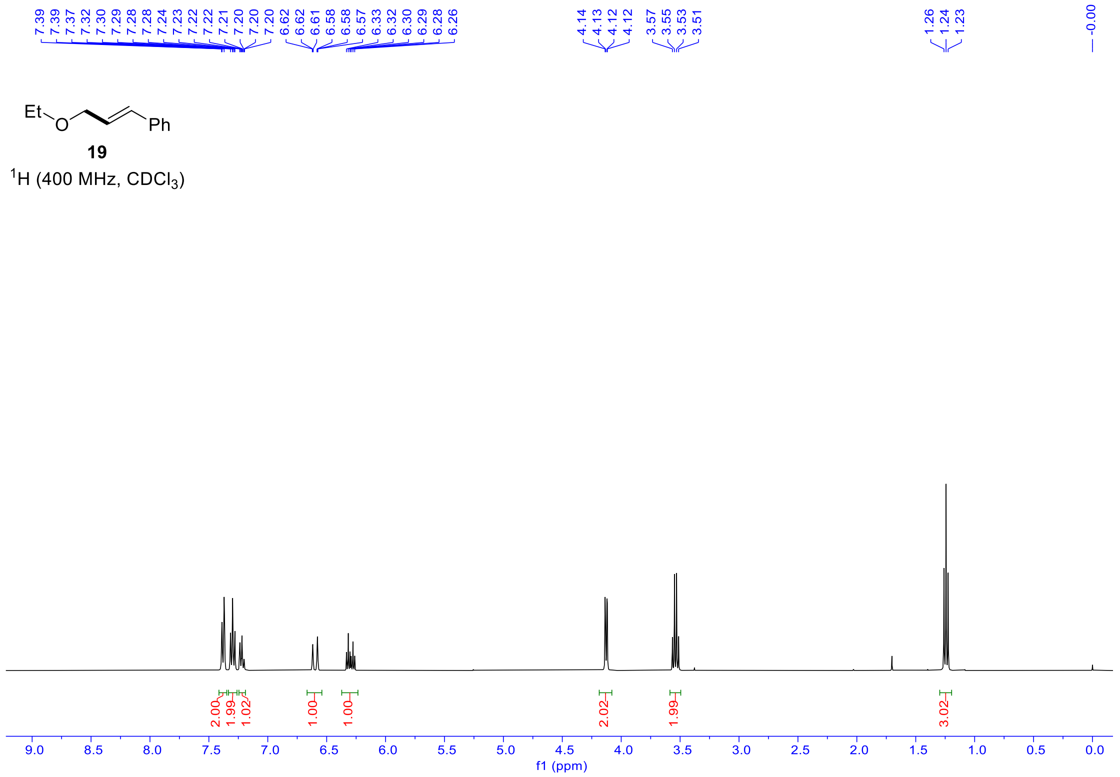

^{13}C NMR spectra of **19** (101 MHz, CDCl$_3$)

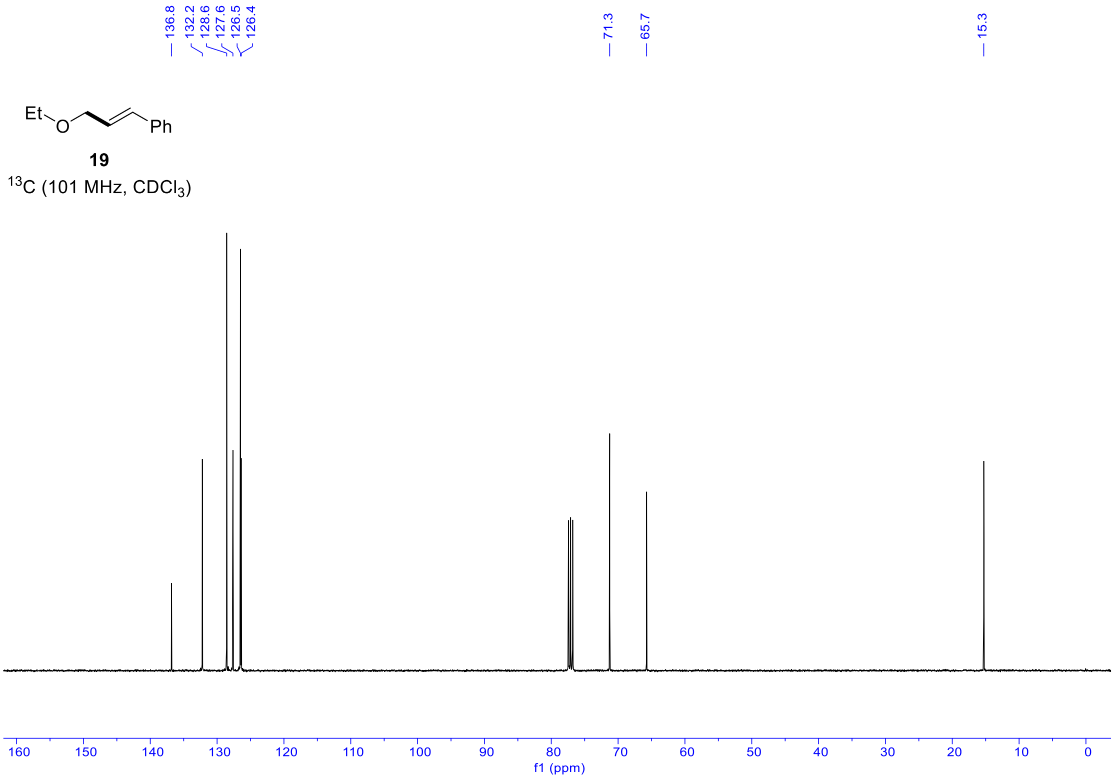

^{1}H NMR spectra of **20** (400 MHz, CDCl$_3$)

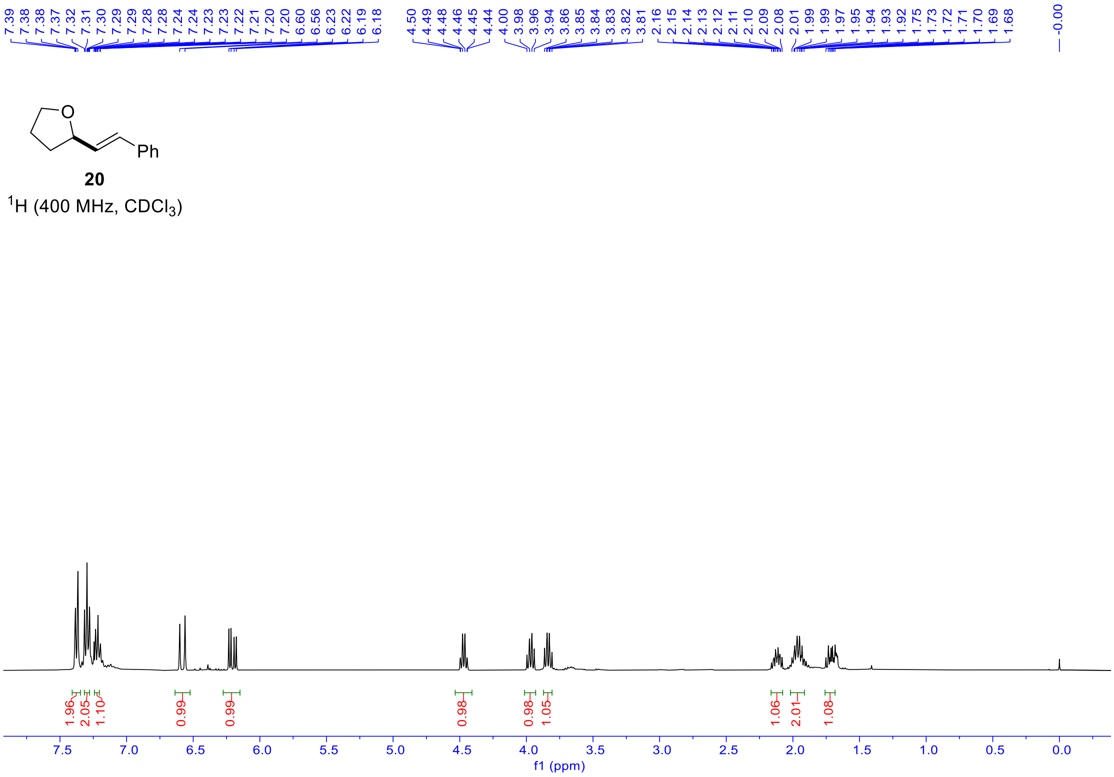

^{13}C NMR spectra of **20** (101 MHz, $CDCl_3$)

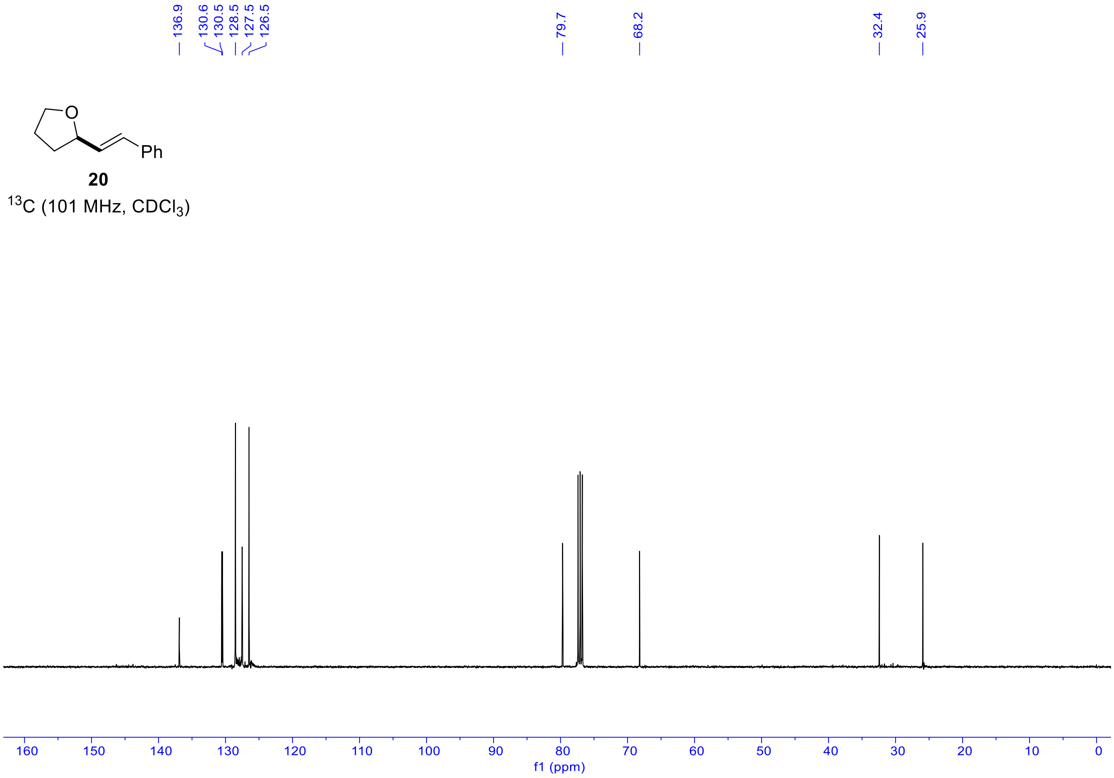

^{1}H NMR spectra of **21** (400 MHz, $CDCl_3$)

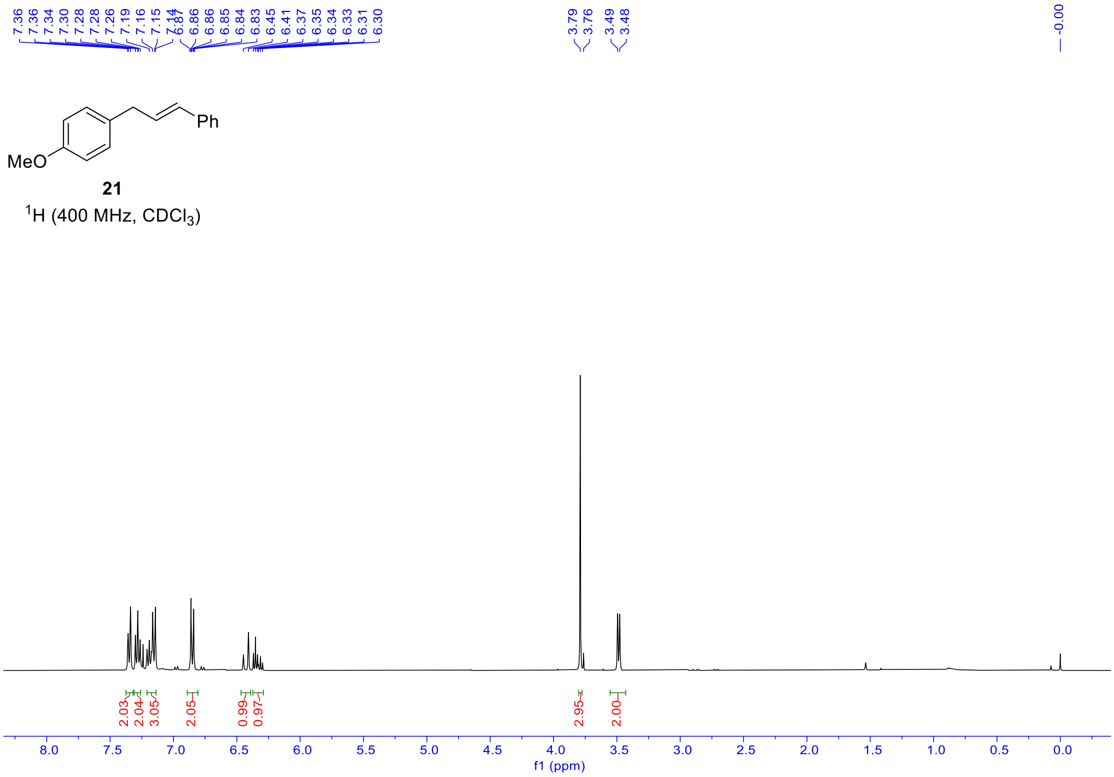

^{13}C NMR spectra of **21** (101 MHz, $CDCl_3$)

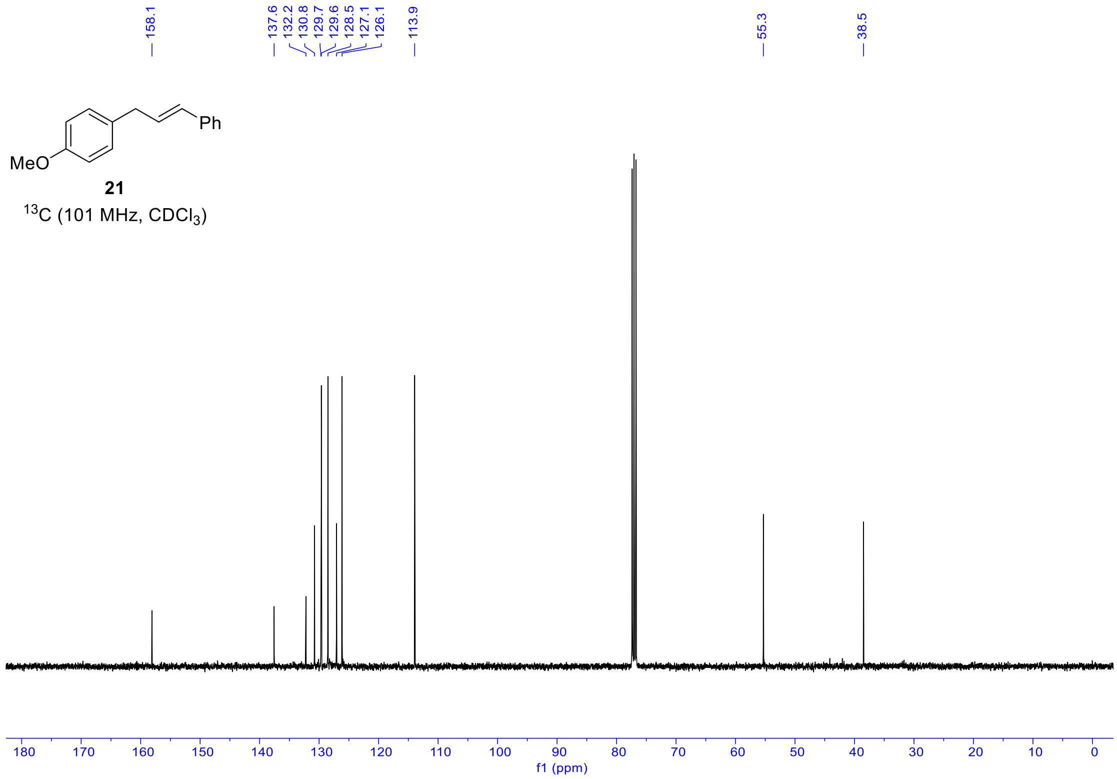

^1H NMR spectra of **22** (400 MHz, $CDCl_3$)

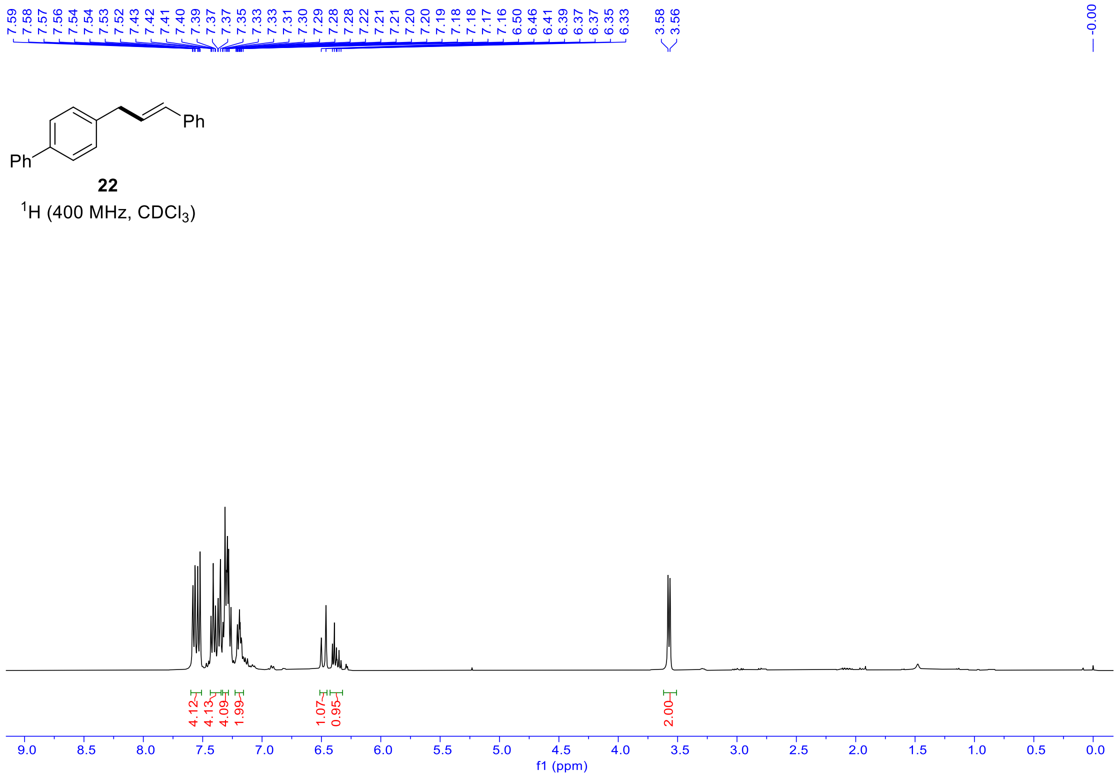

^{13}C NMR spectra of **22** (101 MHz, $CDCl_3$)

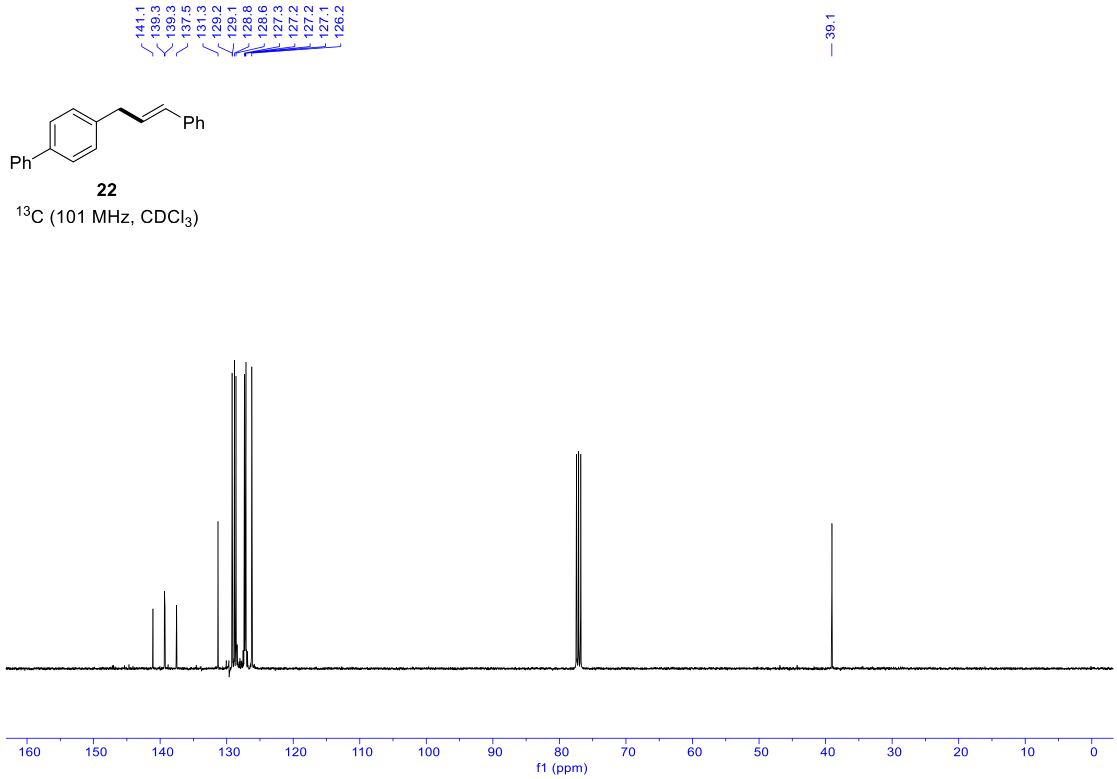

^{1}H NMR spectra of **23** (400 MHz, $CDCl_3$)

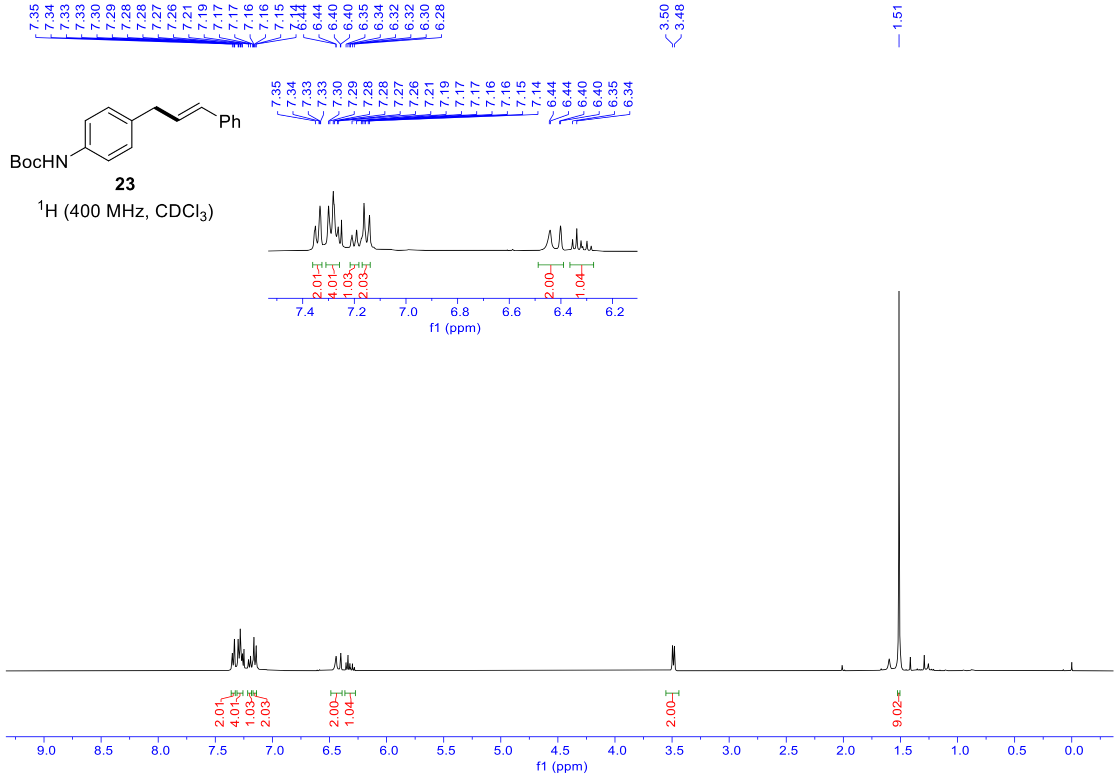

^{13}C NMR spectra of **23** (101 MHz, $CDCl_3$)

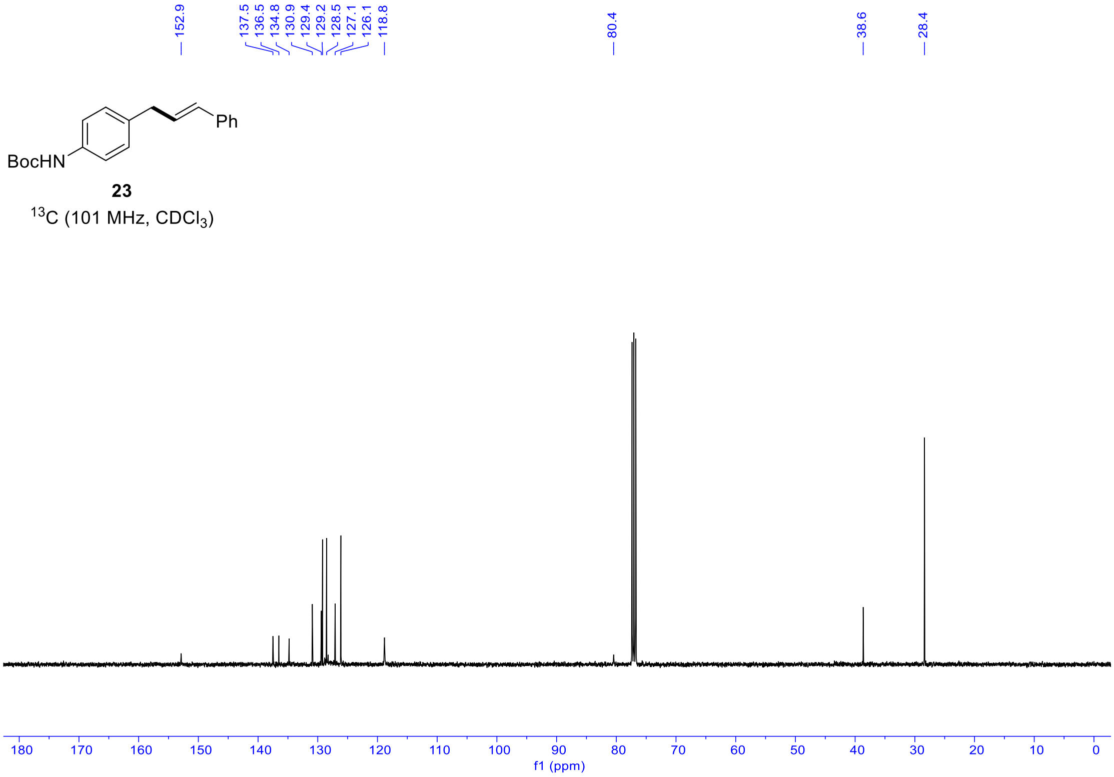

^{1}H NMR spectra of **24** (400 MHz, $CDCl_3$)

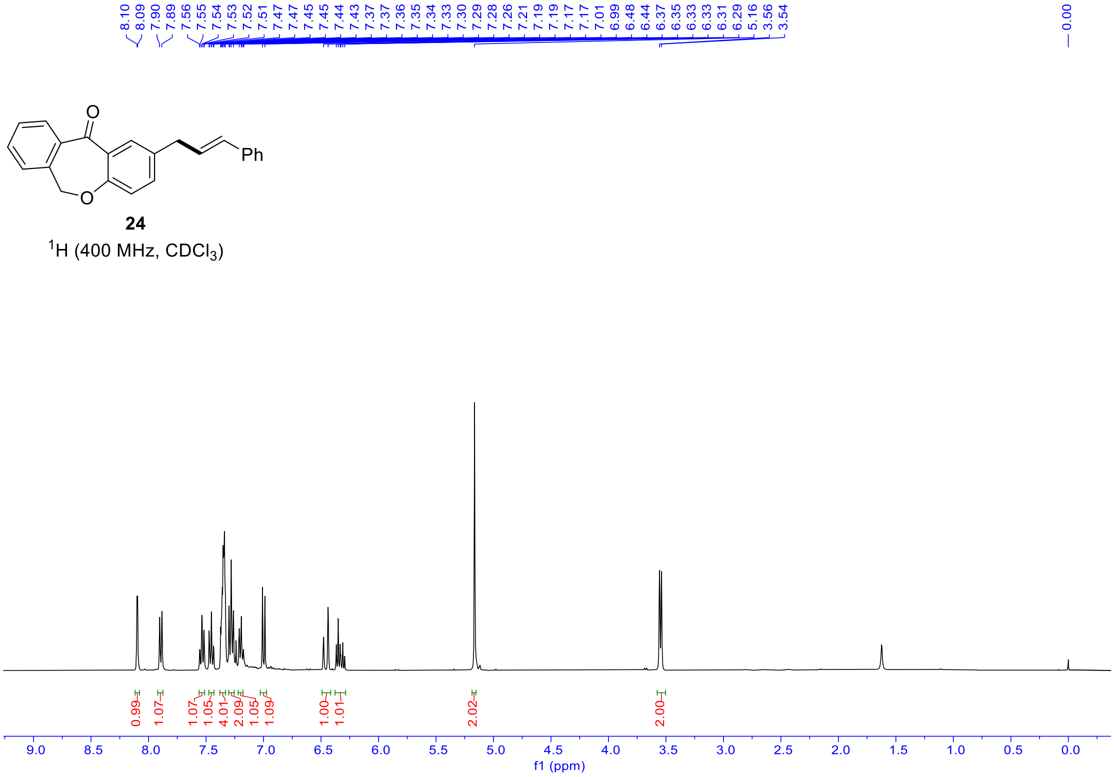

^{13}C NMR spectra of **24** (101 MHz, $CDCl_3$)

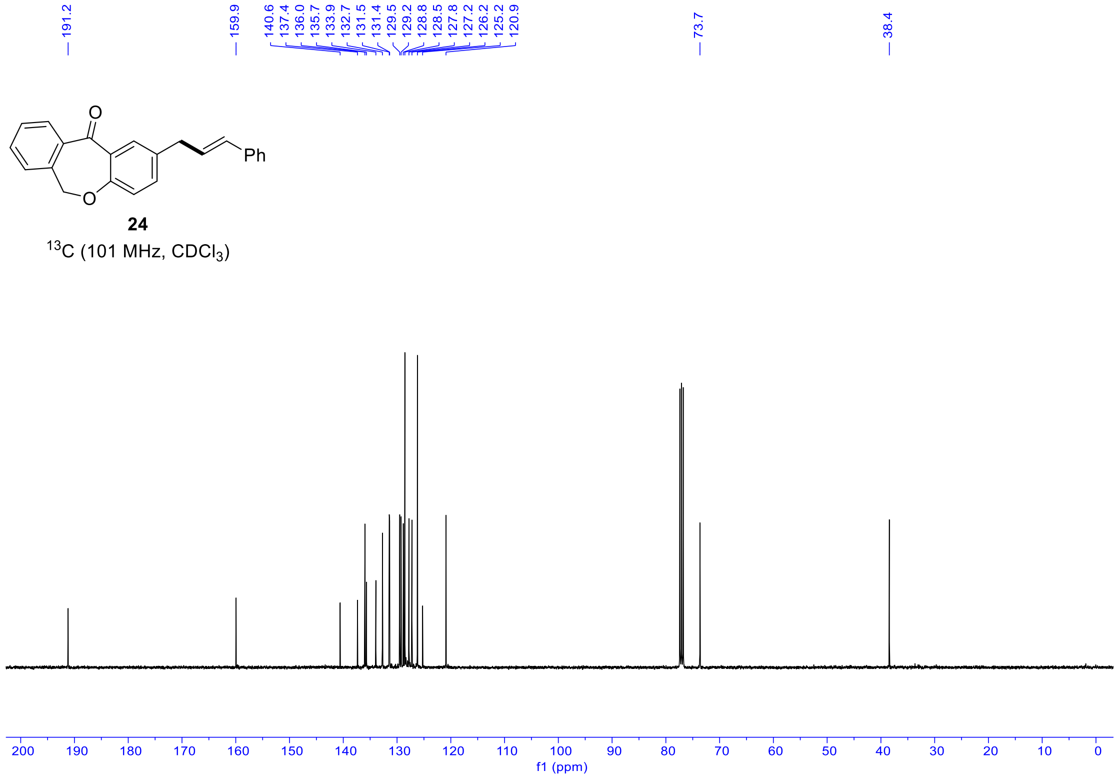

^{1}H NMR spectra of **25** (400 MHz, $CDCl_3$)

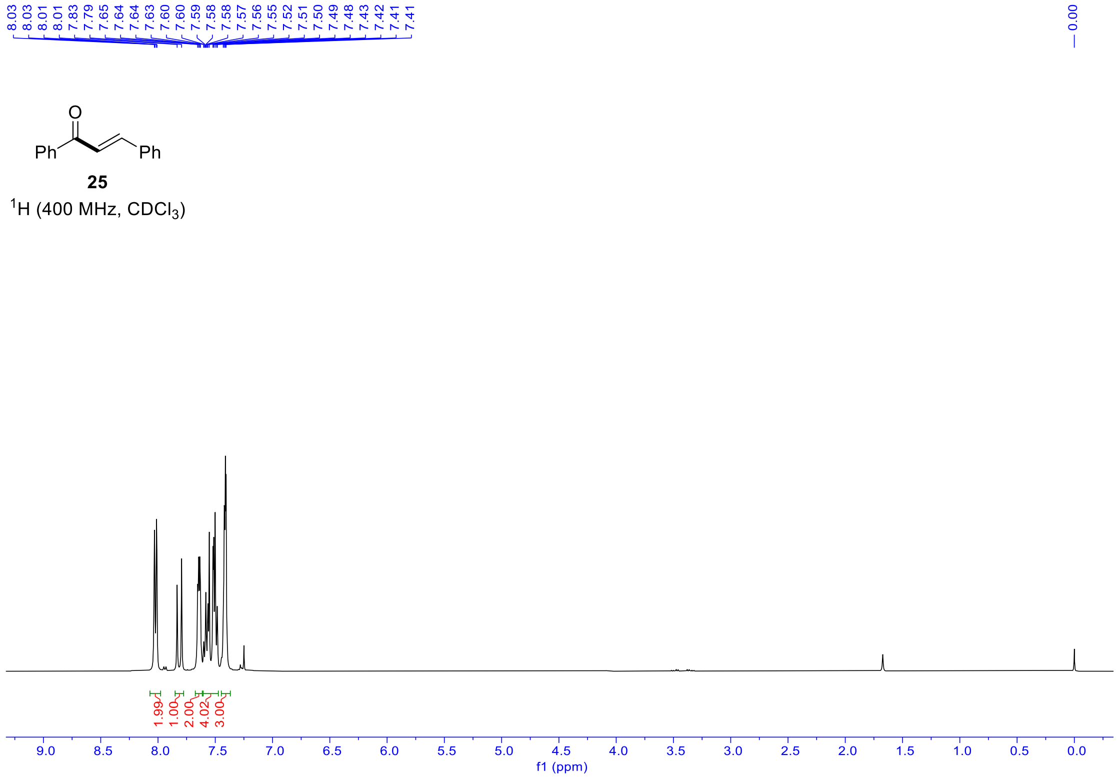

^{13}C NMR spectra of **25** (101 MHz, $CDCl_3$)

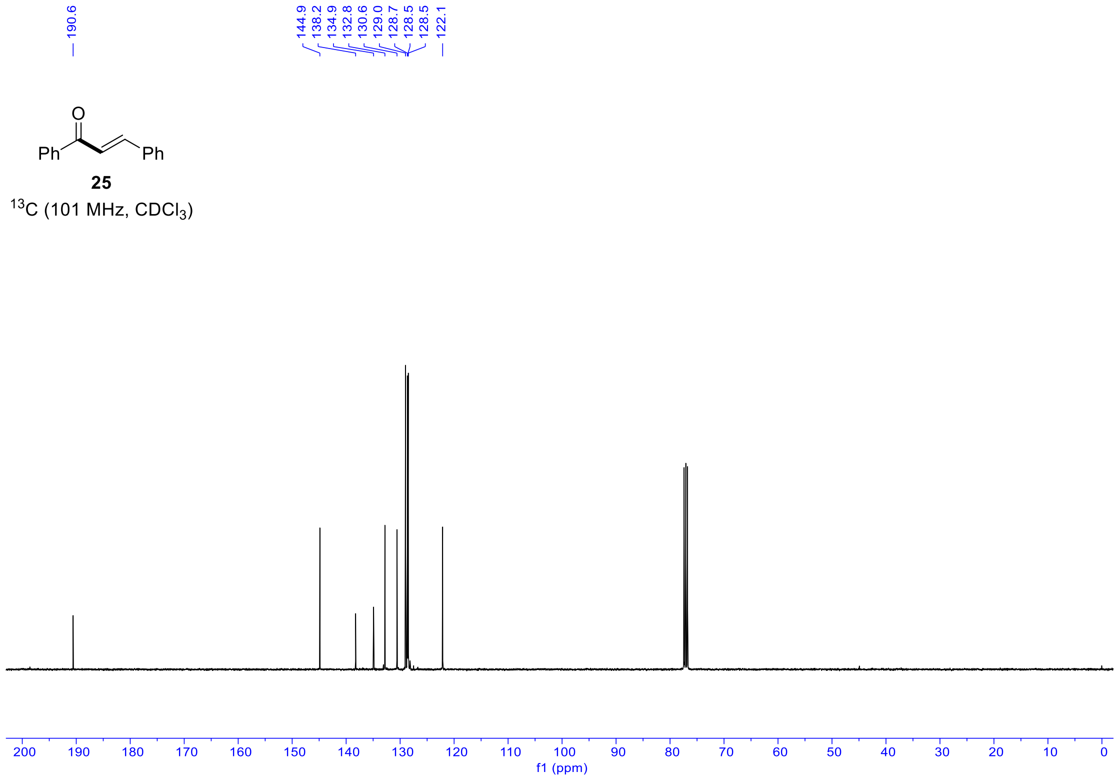

^{1}H NMR spectra of **26** (400 MHz, $CDCl_3$)

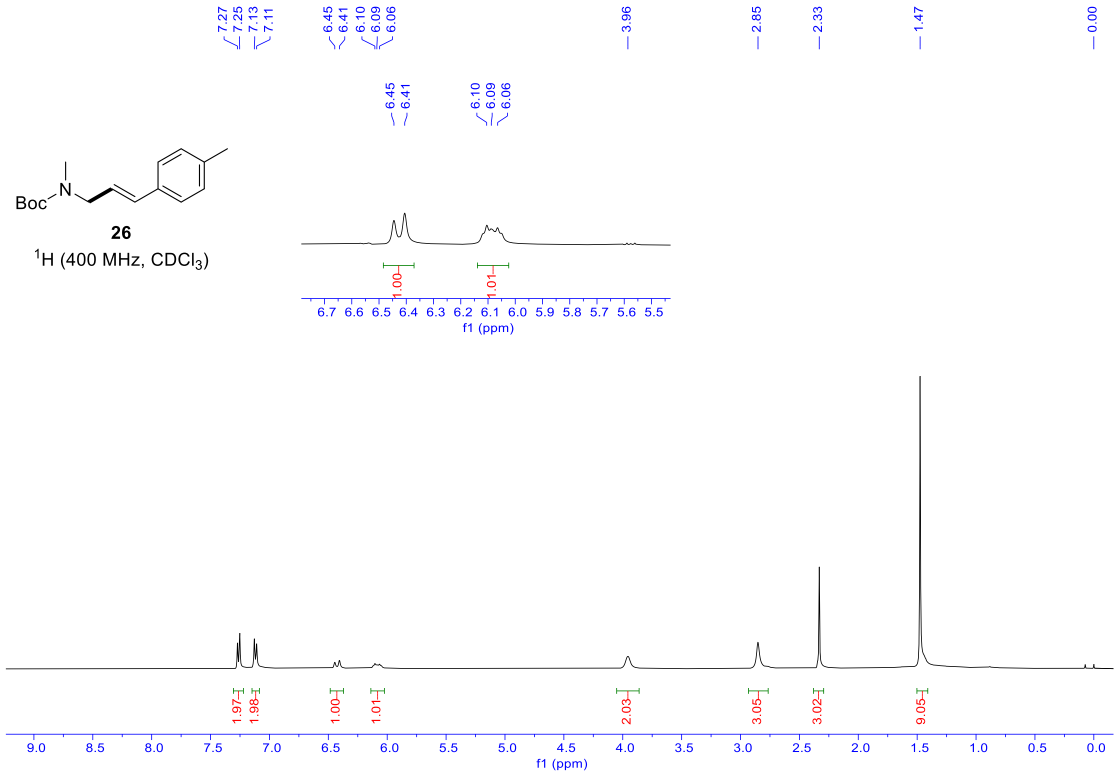

^{13}C NMR spectra of **26** (101 MHz, $CDCl_3$)

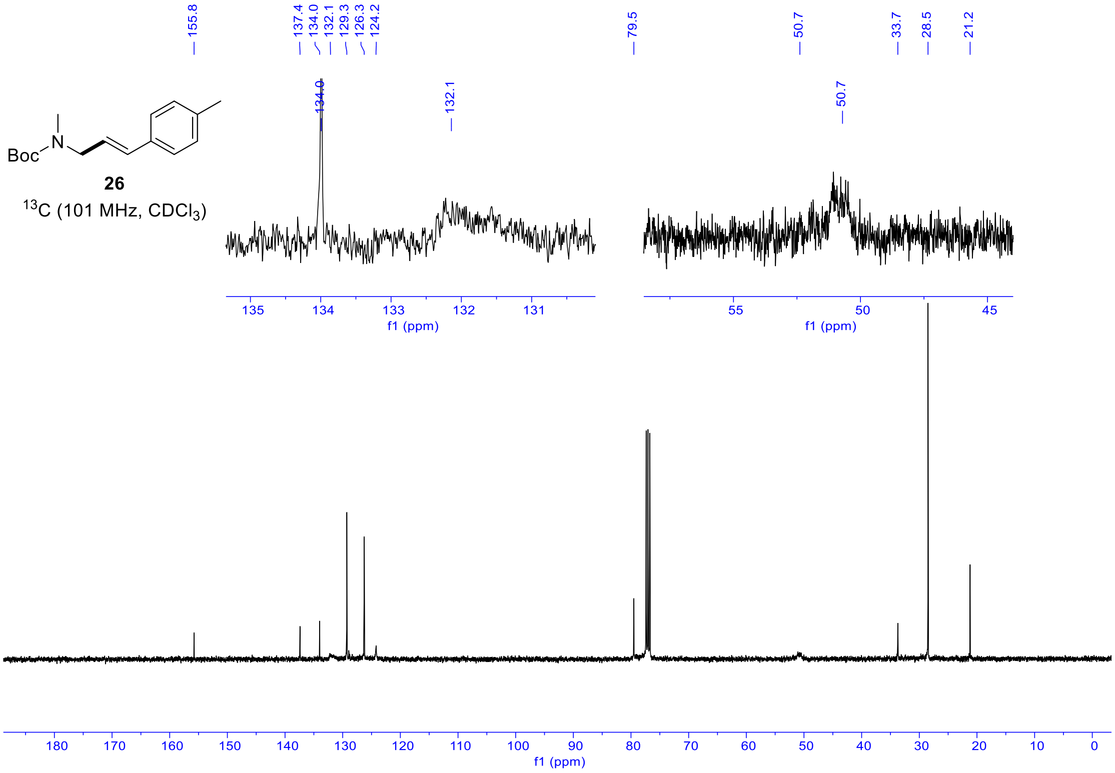

^{1}H NMR spectra of **27** (400 MHz, $CDCl_3$)

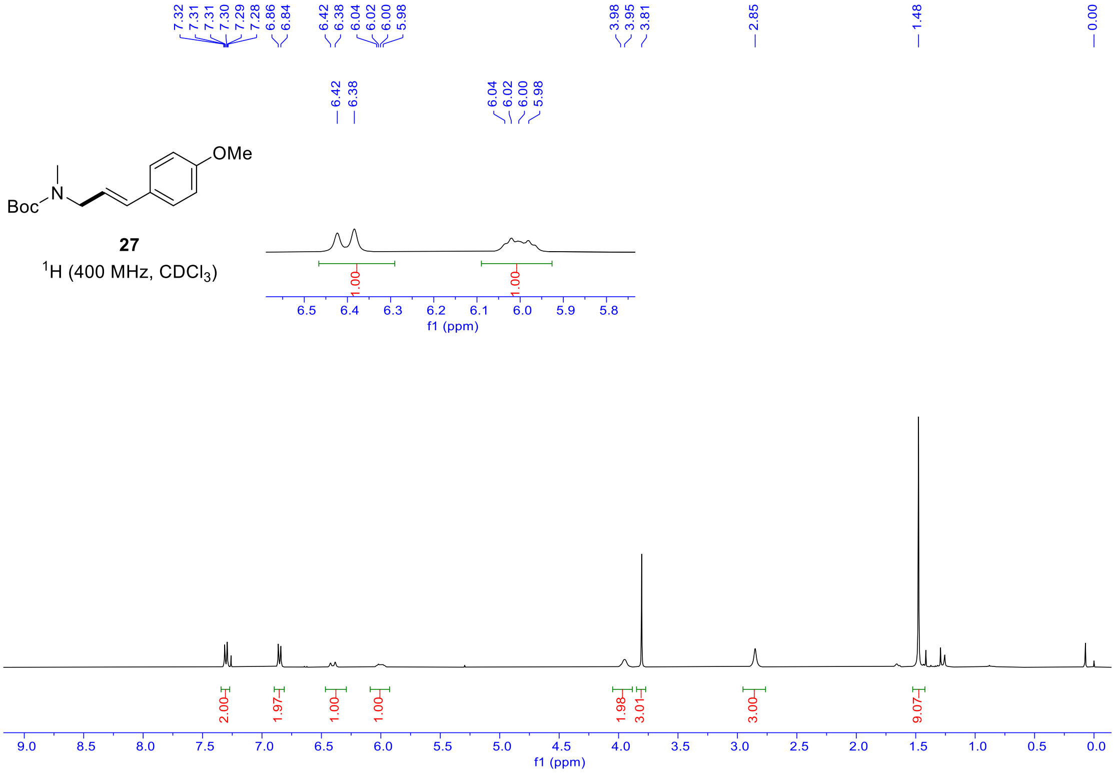

^{13}C NMR spectra of **27** (101 MHz, CDCl$_3$)

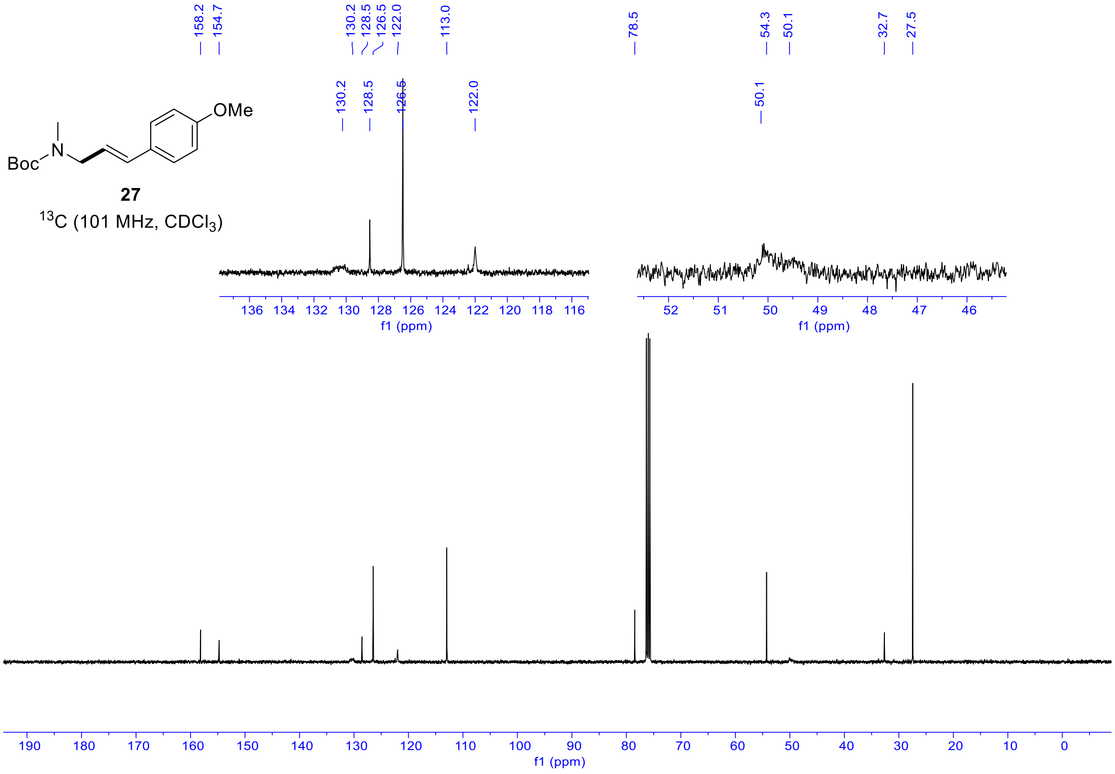

^{1}H NMR spectra of **28** (400 MHz, CDCl$_3$)

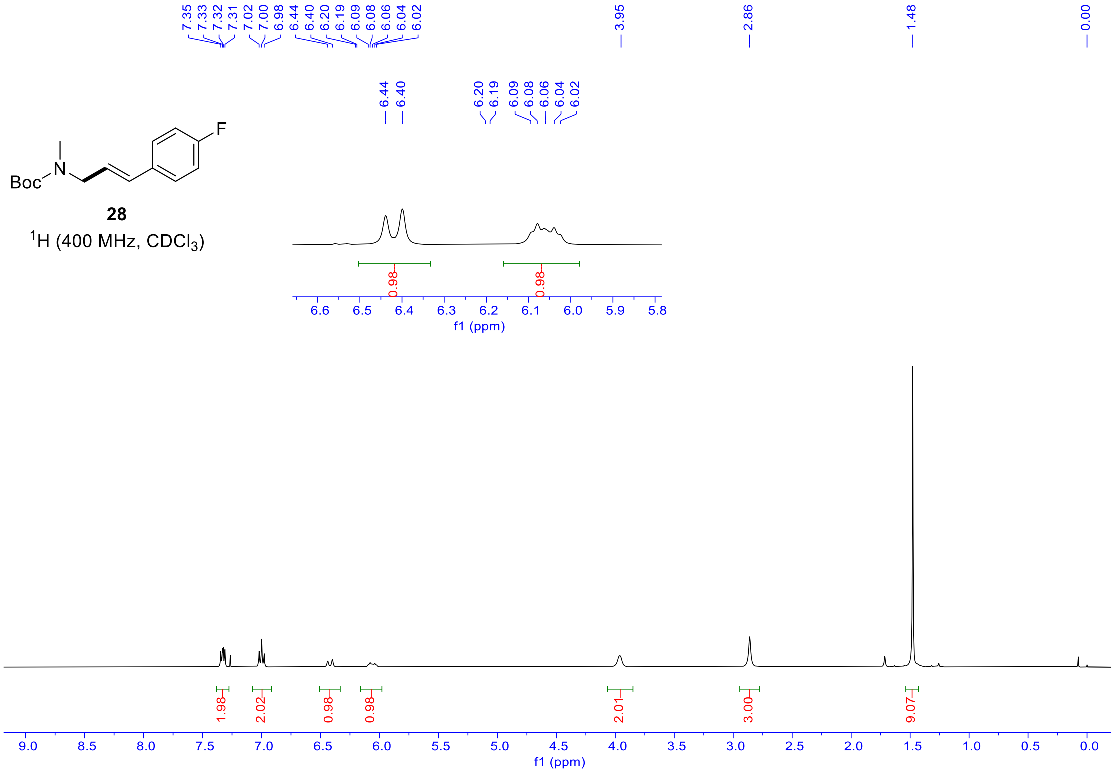

^{13}C NMR spectra of **28** (101 MHz, $CDCl_3$)

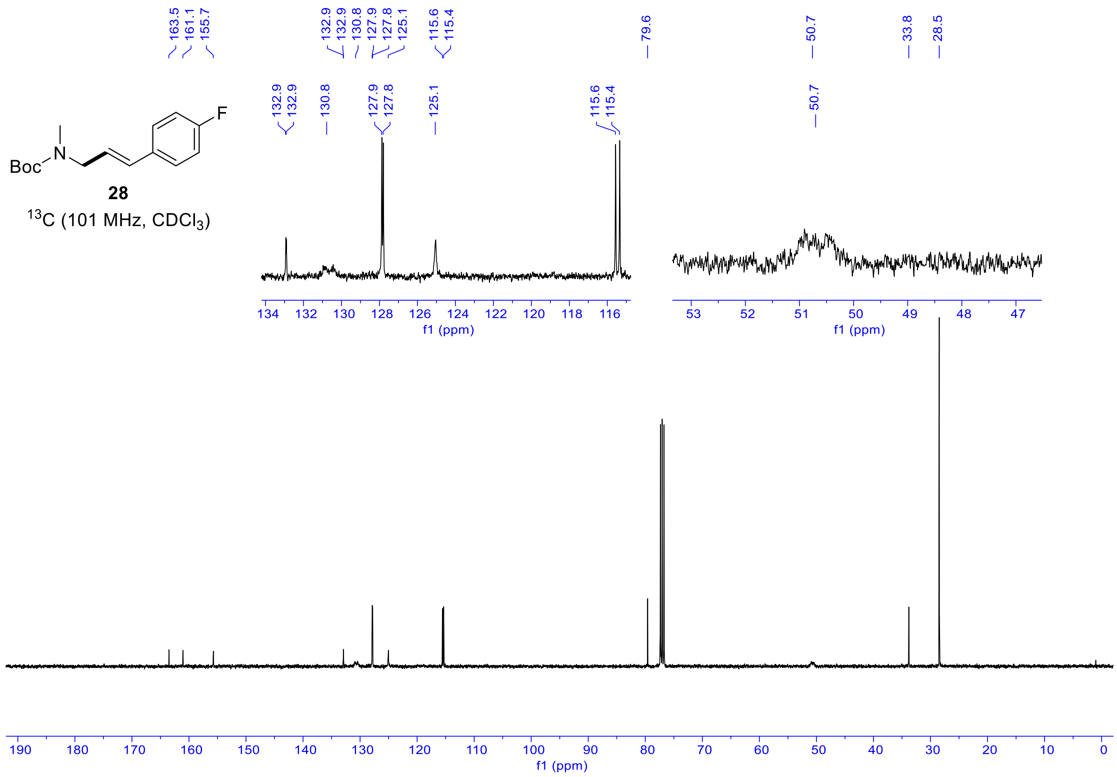

^{19}F NMR spectra of **28** (377 MHz, $CDCl_3$)

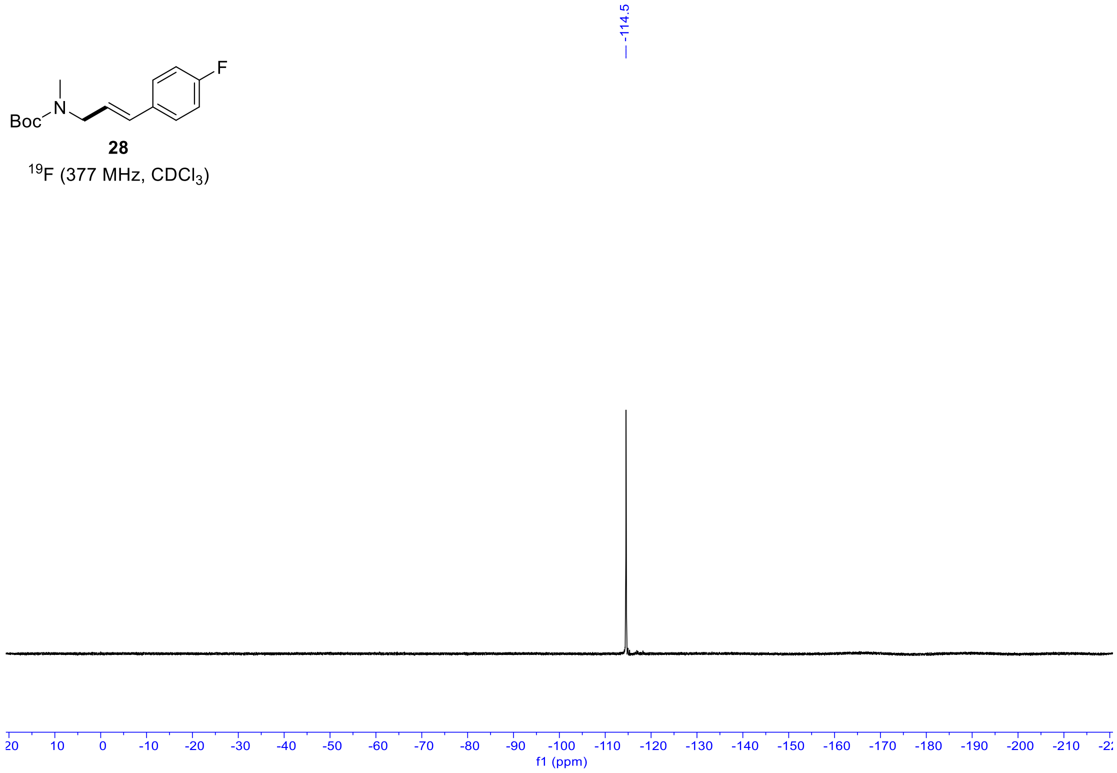

^{1}H NMR spectra of **29** (400 MHz, $CDCl_3$)

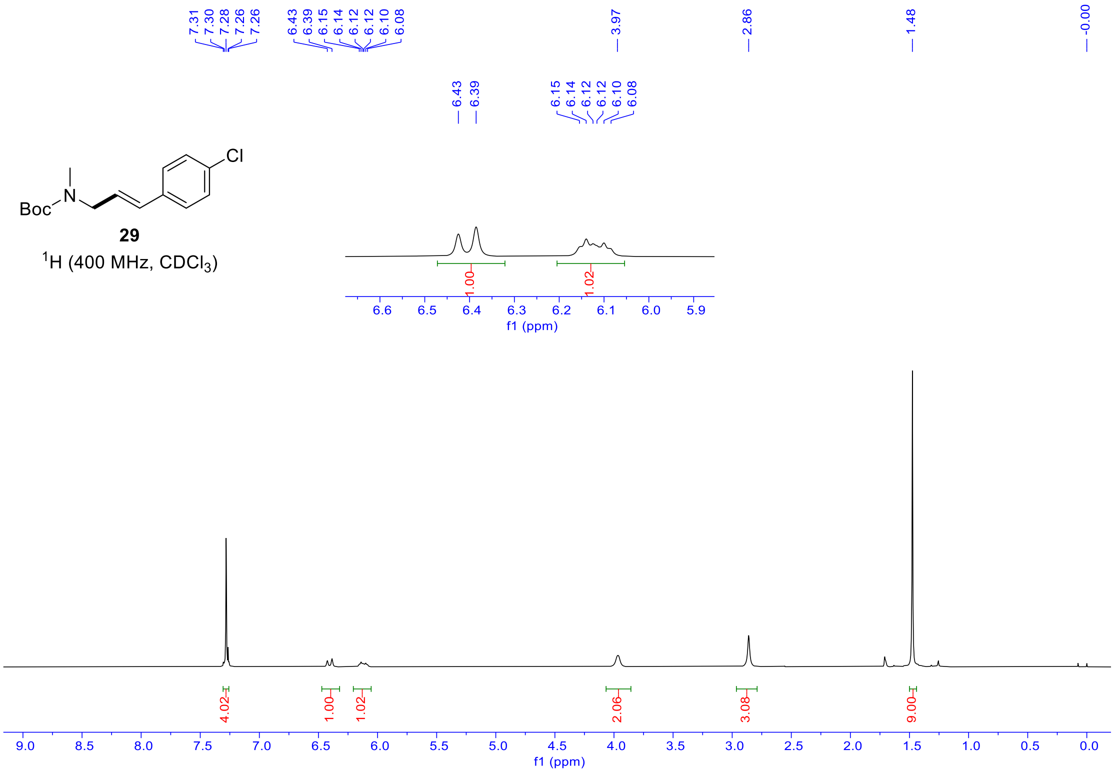

^{13}C NMR spectra of **29** (101 MHz, $CDCl_3$)

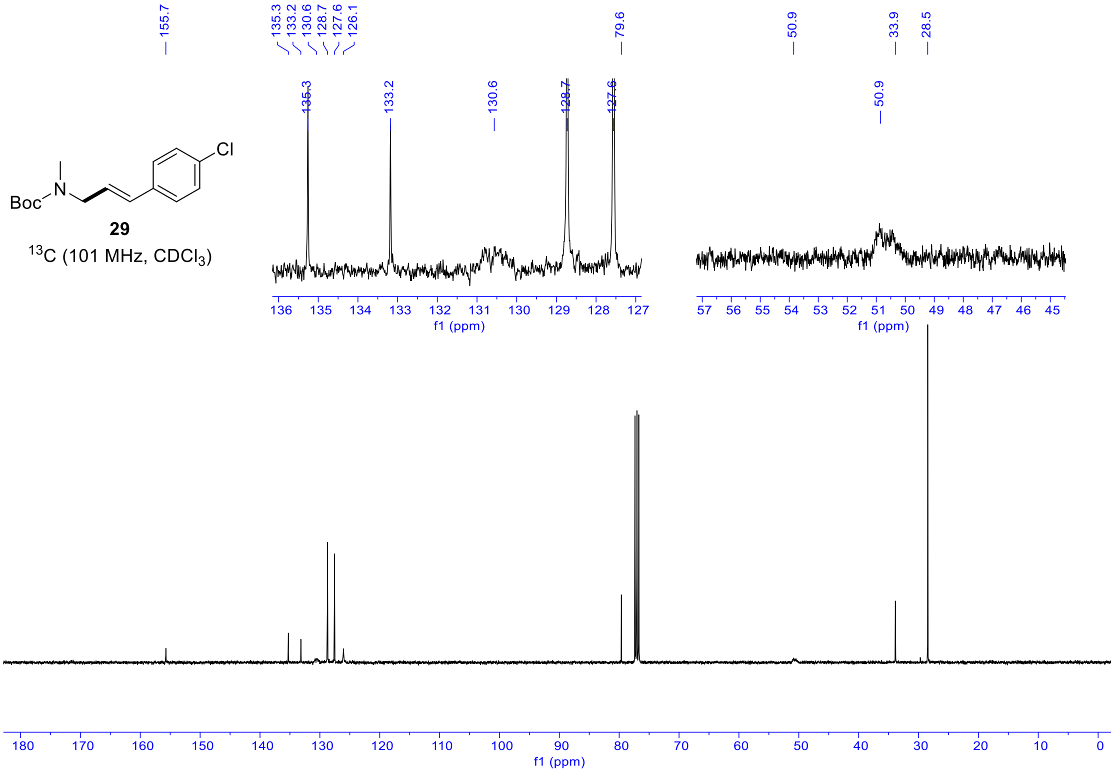

^{1}H NMR spectra of **30** (400 MHz, $CDCl_3$)

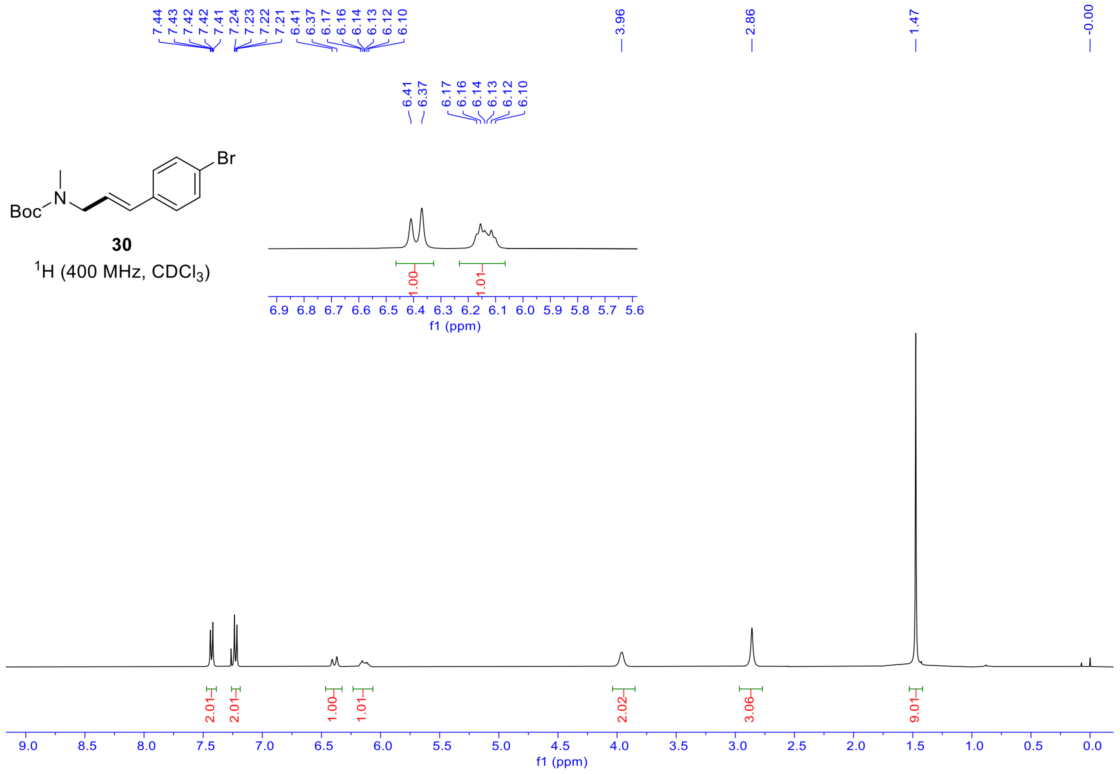

^{13}C NMR spectra of **30** (101 MHz, $CDCl_3$)

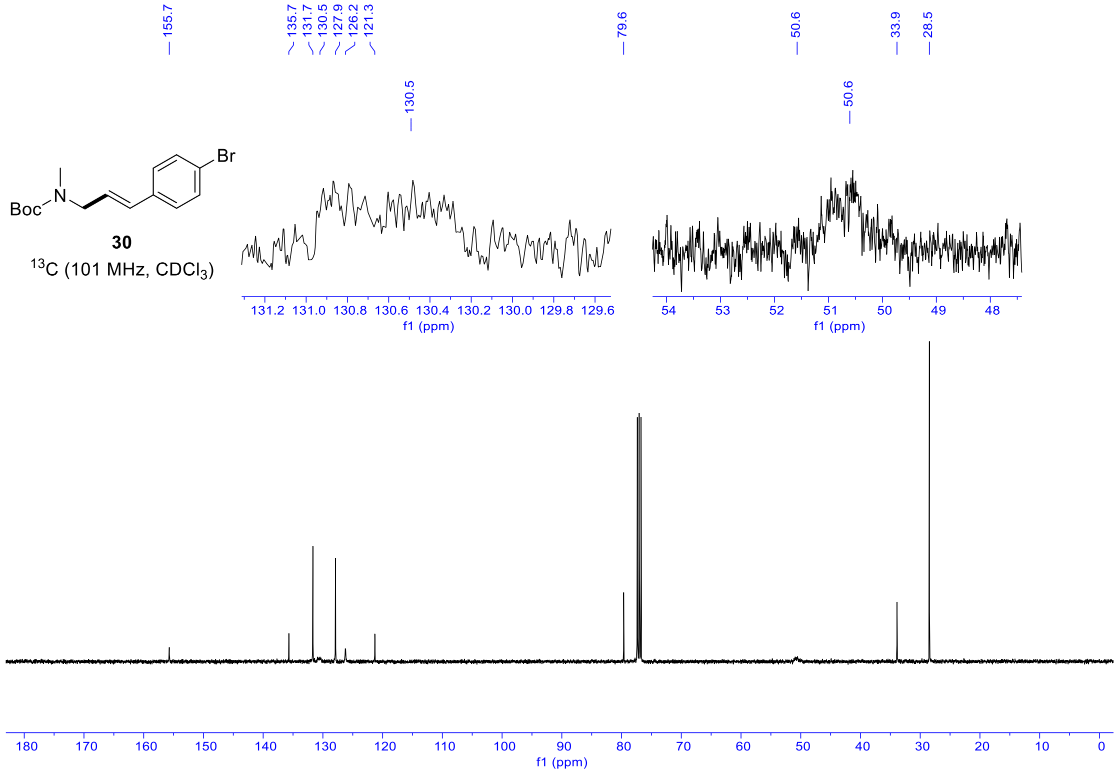

^{1}H NMR spectra of **31** (400 MHz, $CDCl_3$)

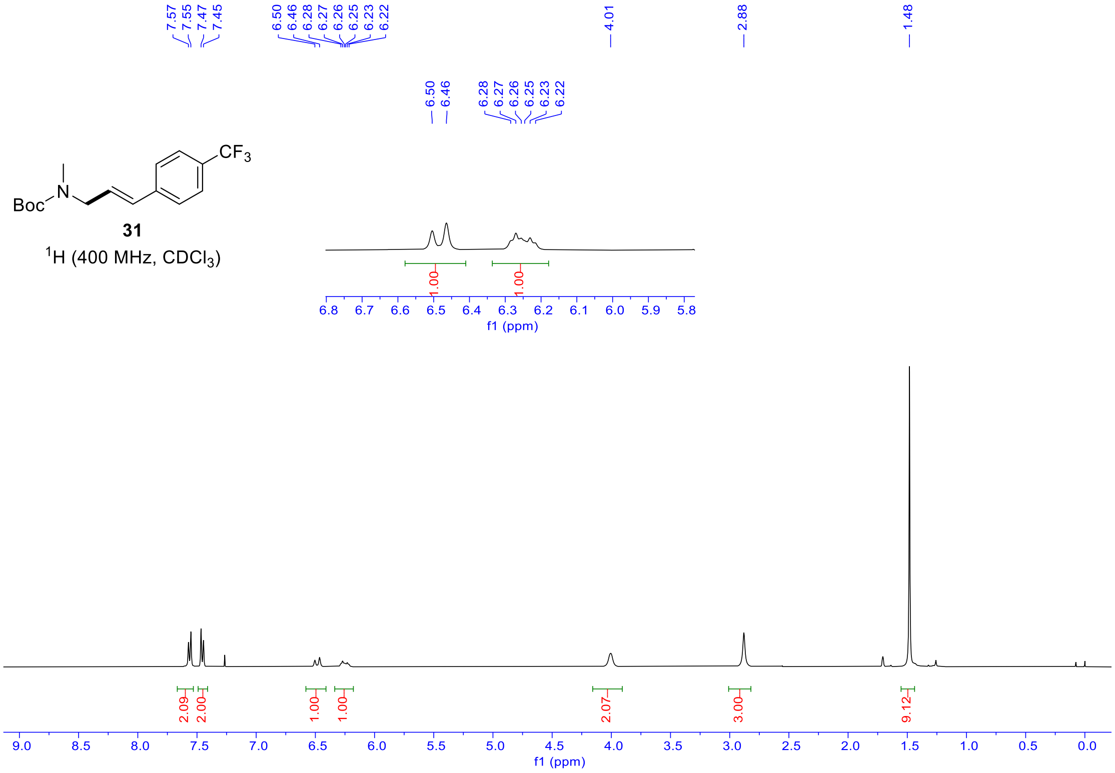

^{13}C NMR spectra of **31** (101 MHz, $CDCl_3$)

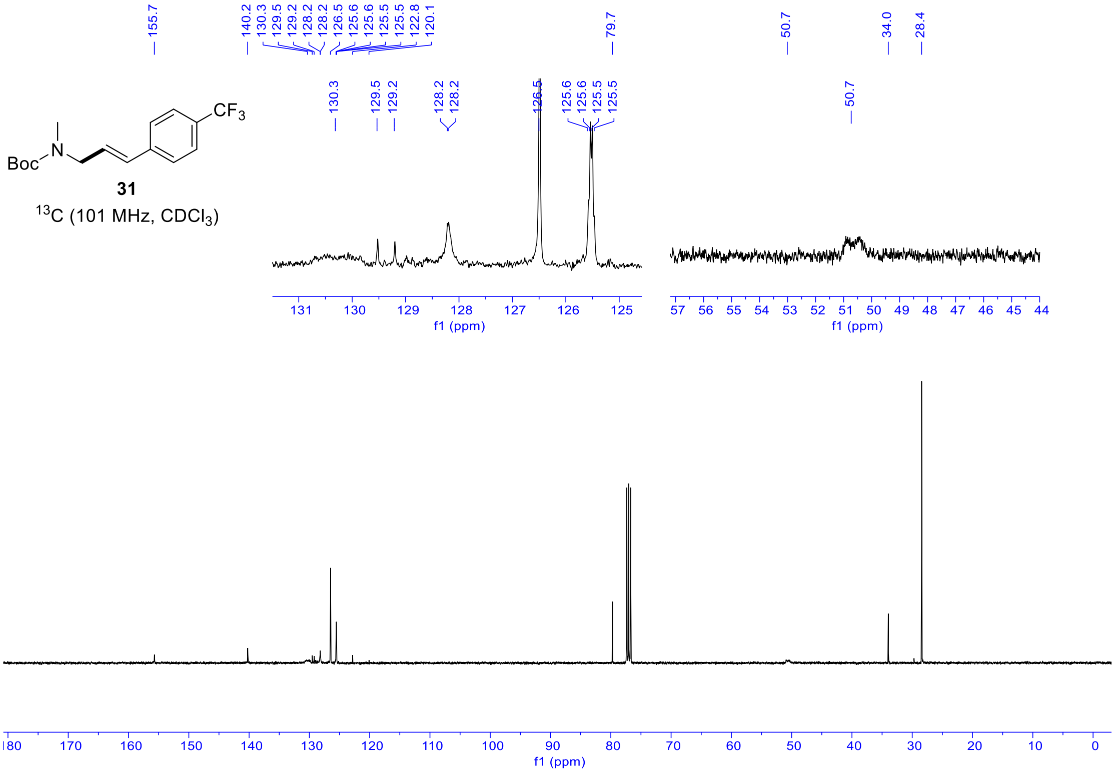

^{19}F NMR spectra of **31** (377 MHz, $CDCl_3$)

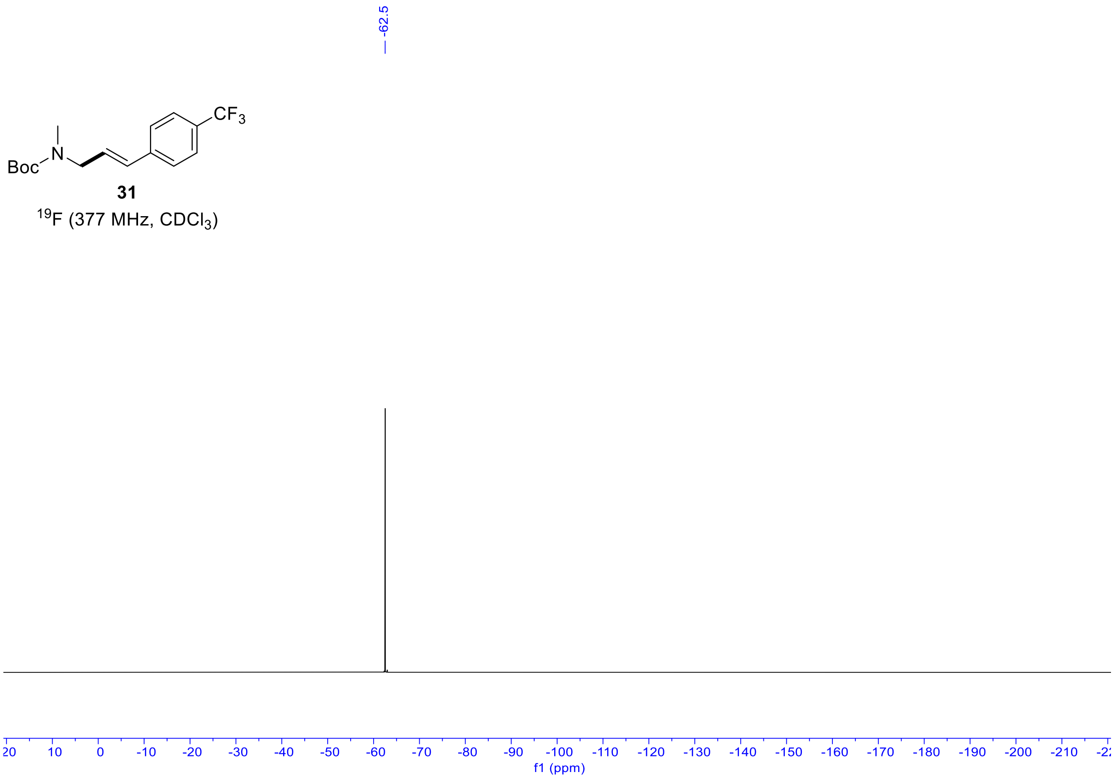

^{1}H NMR spectra of **32** (400 MHz, $CDCl_3$)

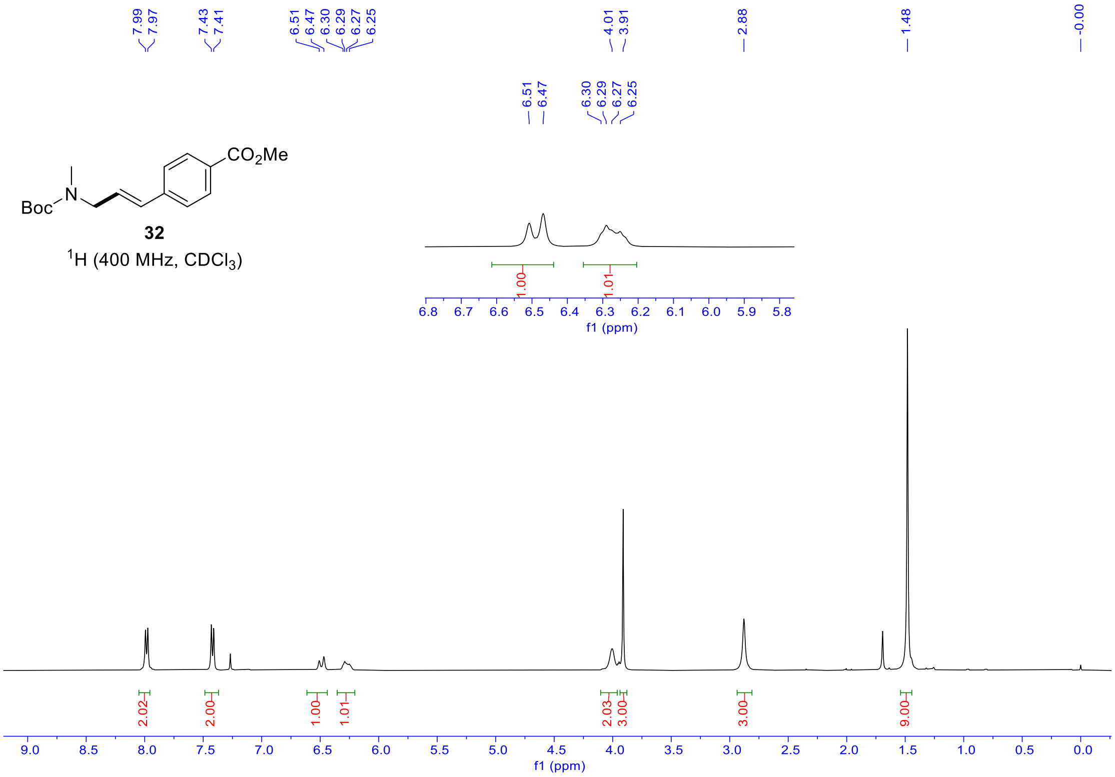

^{13}C NMR spectra of **32** (101 MHz, CDCl$_3$)

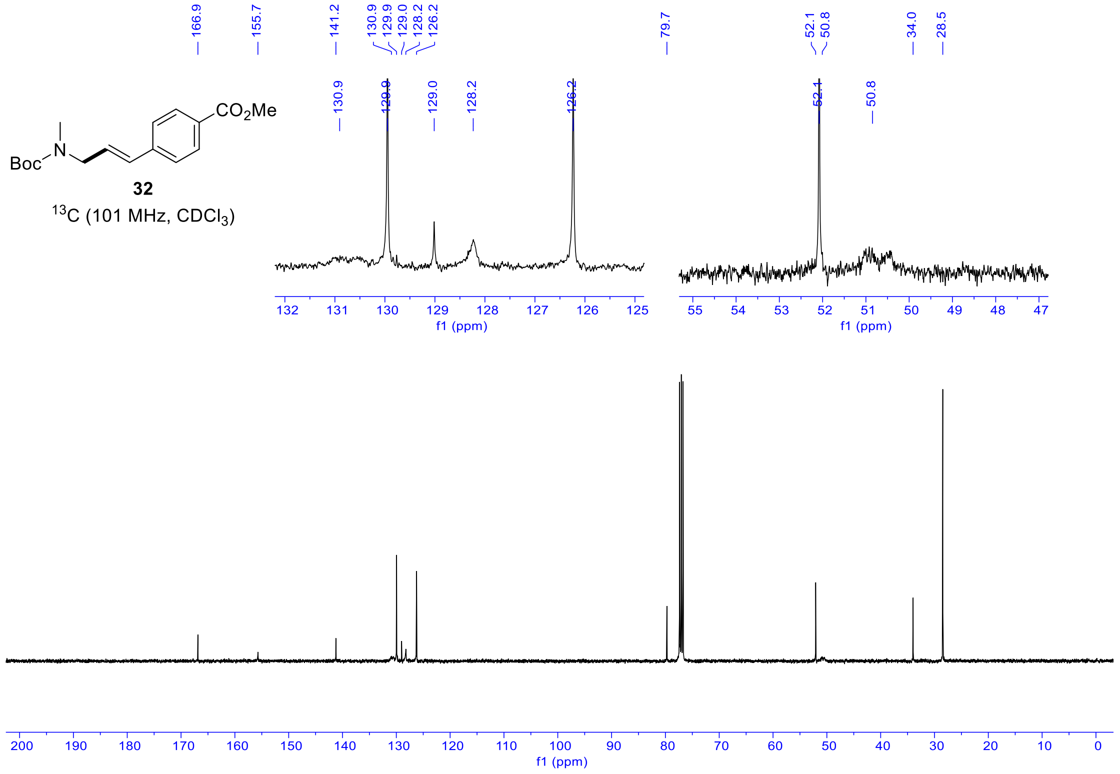

^{1}H NMR spectra of **33** (400 MHz, CDCl$_3$)

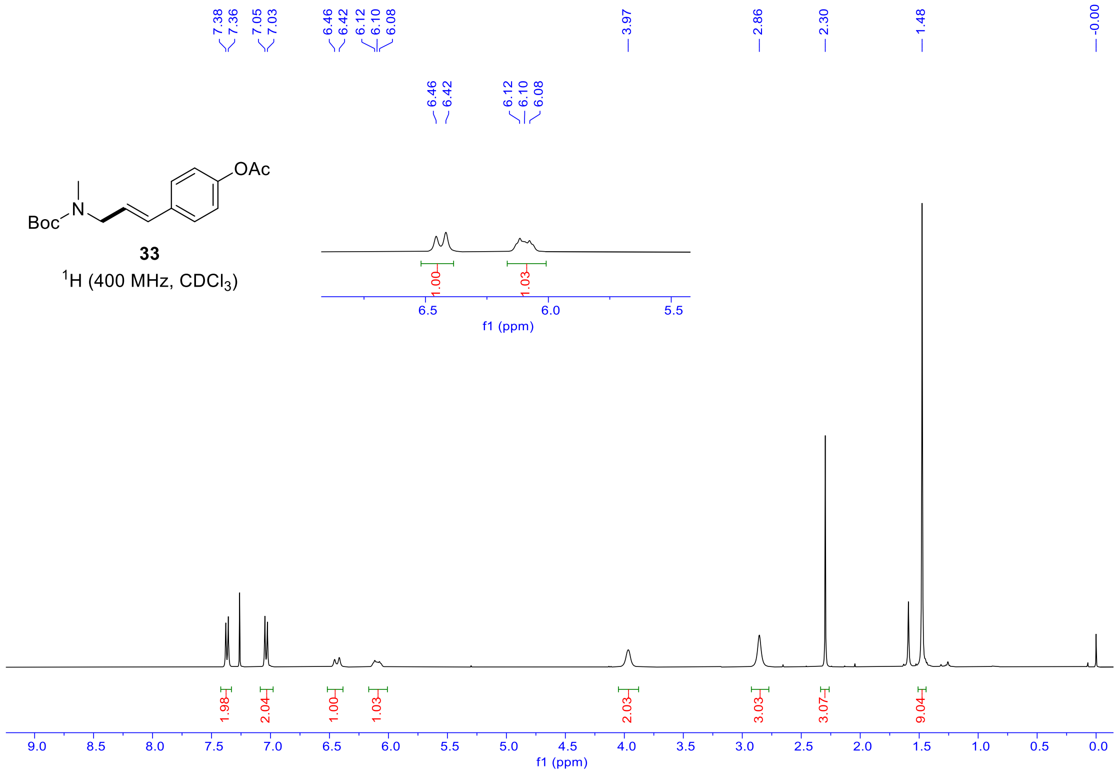

^{13}C NMR spectra of **33** (101 MHz, $CDCl_3$)

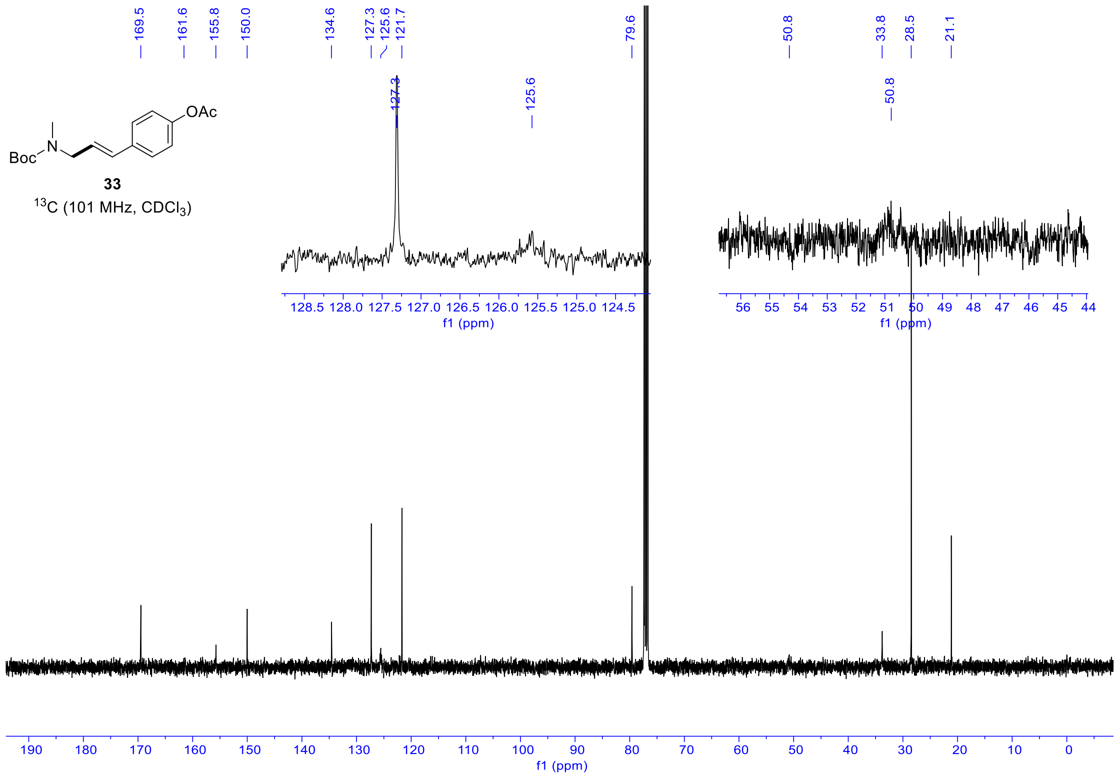

^{1}H NMR spectra of **34** (400 MHz, $CDCl_3$)

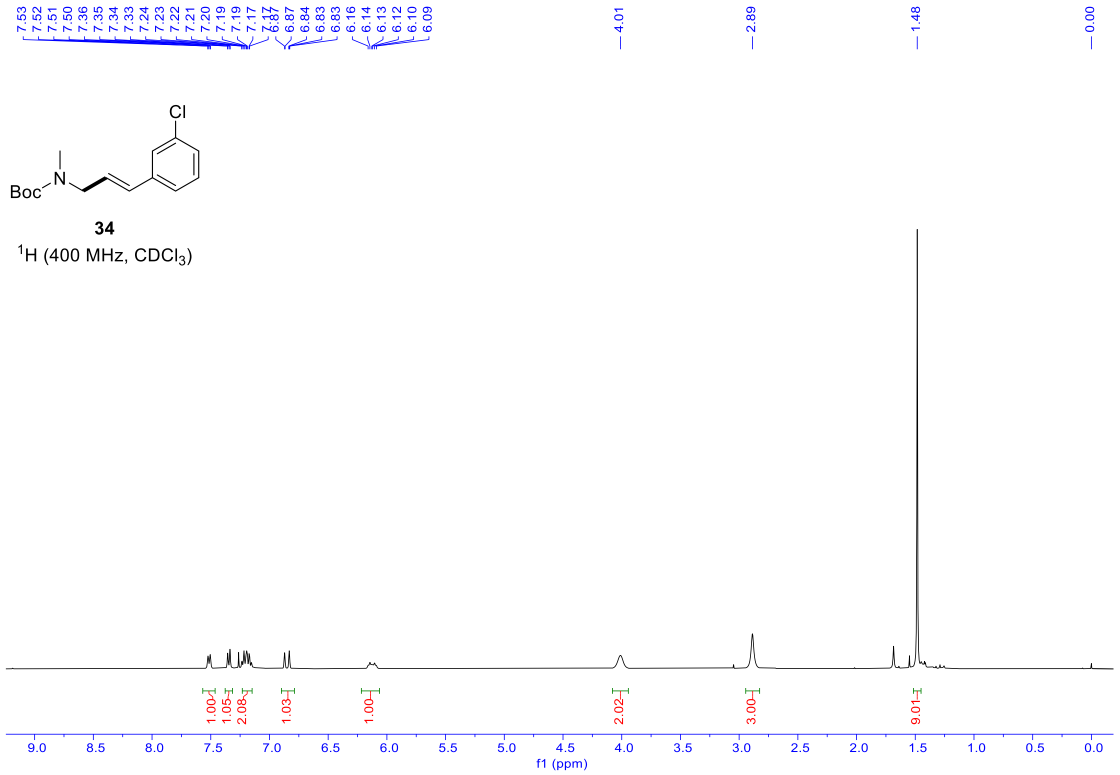

^{13}C NMR spectra of **34** (101 MHz, CDCl$_3$)

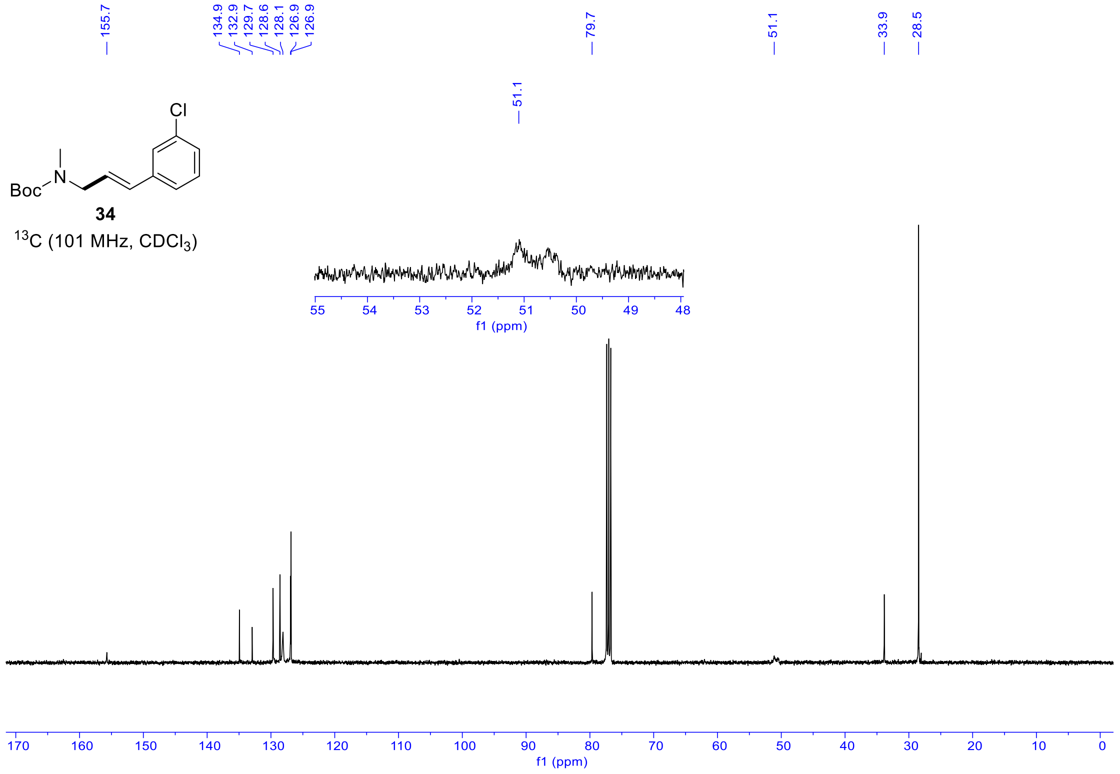

^{1}H NMR spectra of **35** (400 MHz, CDCl$_3$)

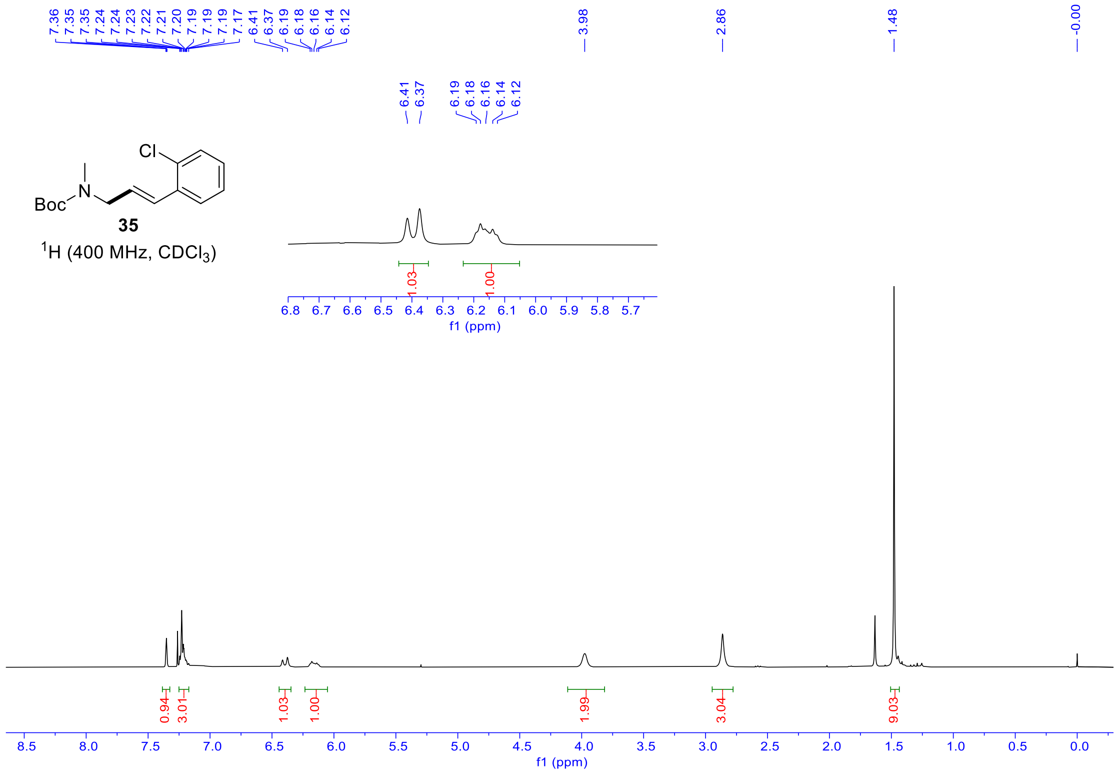

^{13}C NMR spectra of **35** (101 MHz, $CDCl_3$)

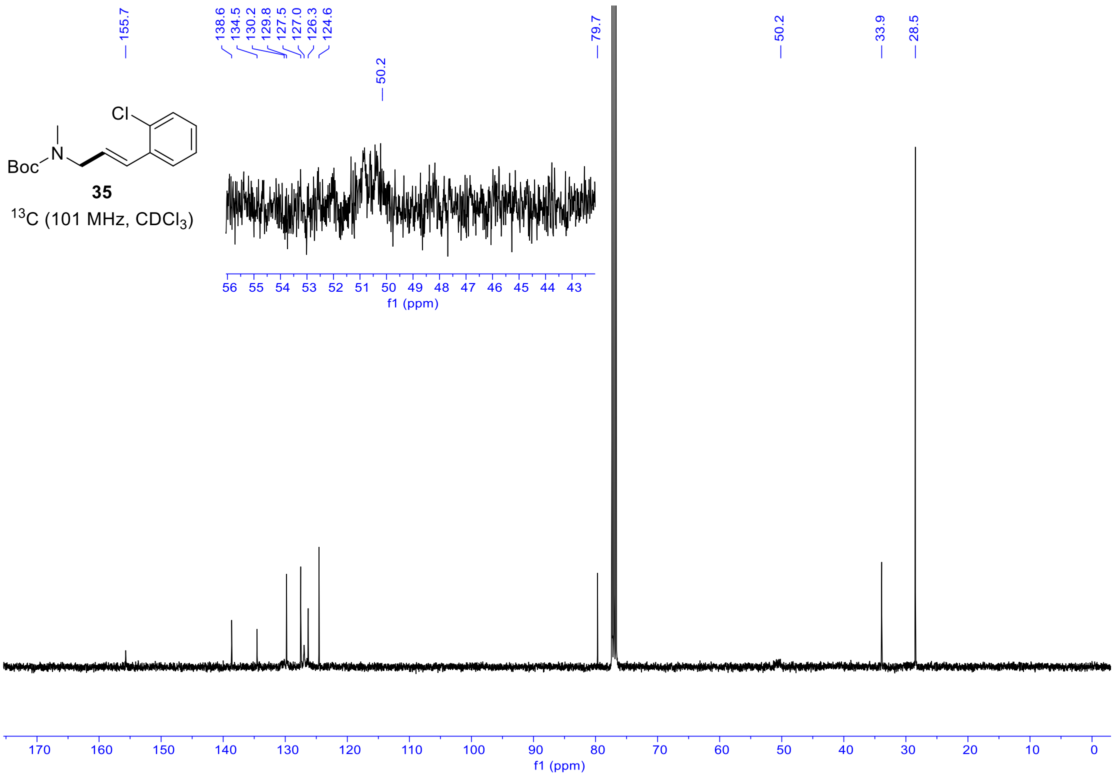

^{1}H NMR spectra of **36** (400 MHz, $CDCl_3$)

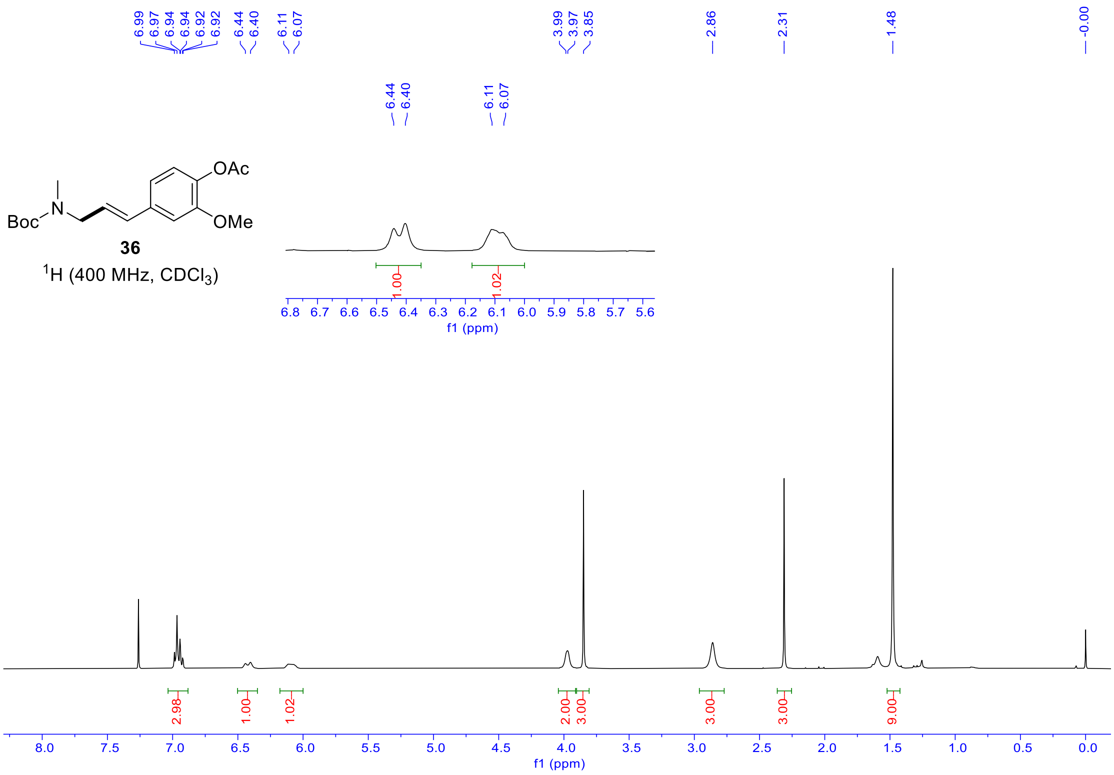

^{13}C NMR spectra of **36** (101 MHz, CDCl$_3$)

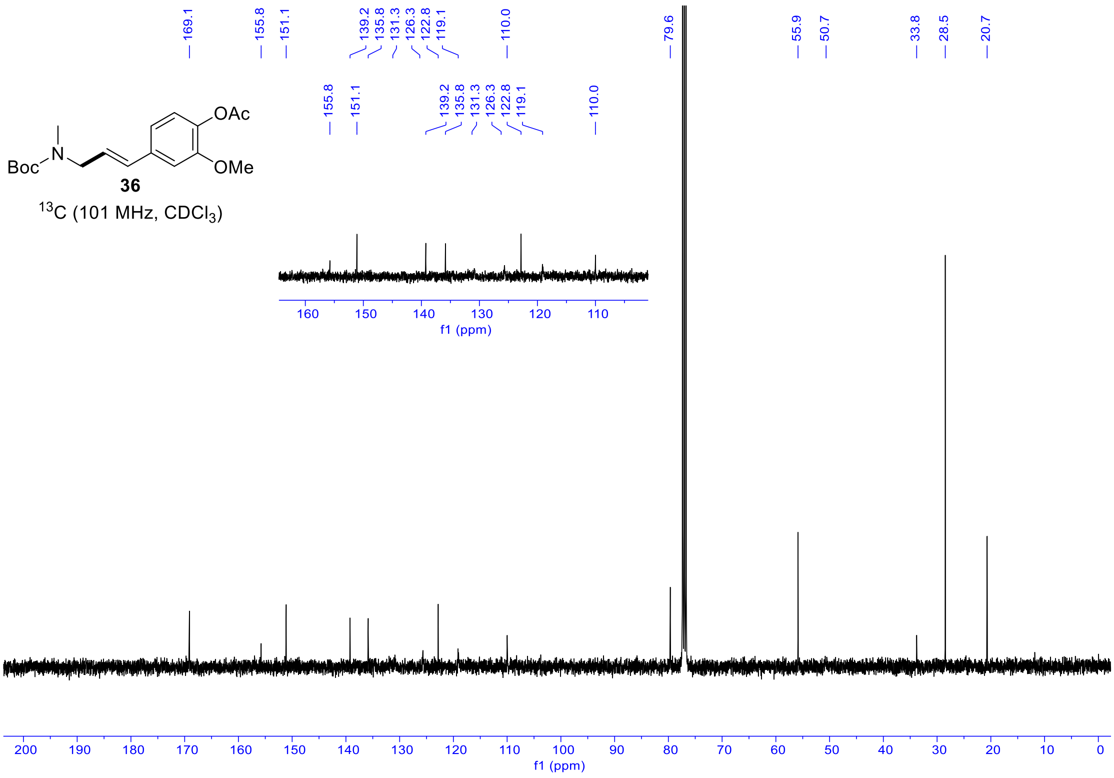

^{1}H NMR spectra of **37** (400 MHz, CDCl$_3$)

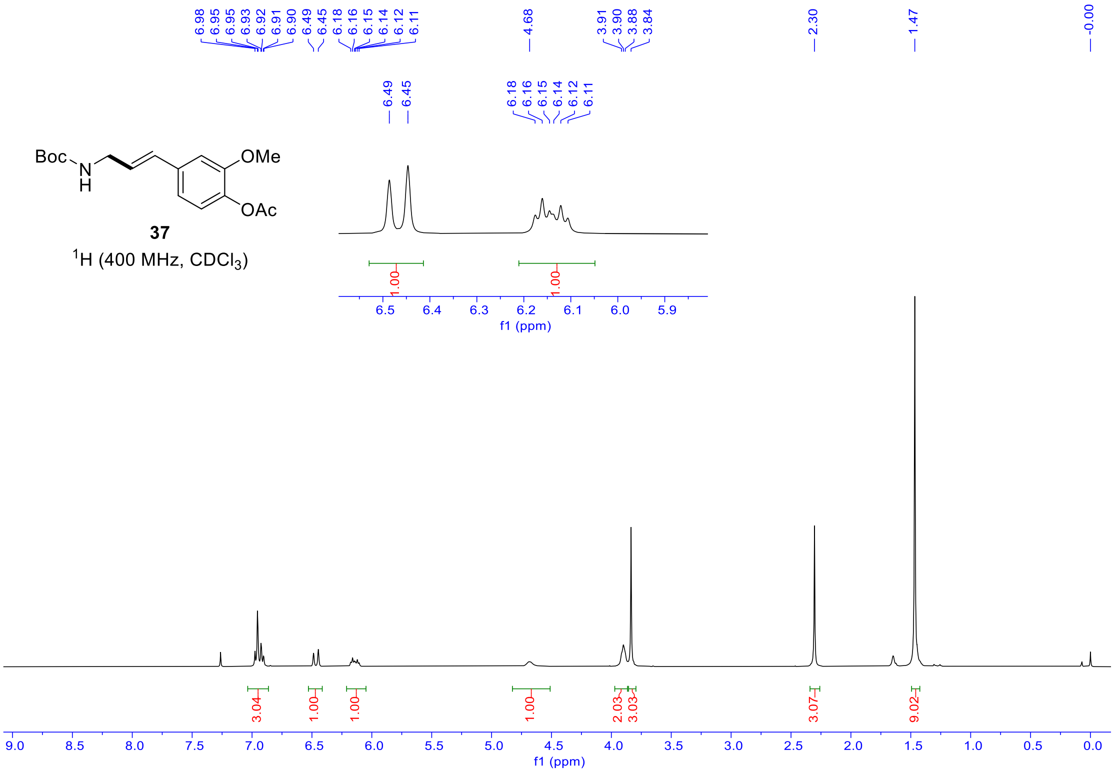

^{13}C NMR spectra of **37** (101 MHz, $CDCl_3$)

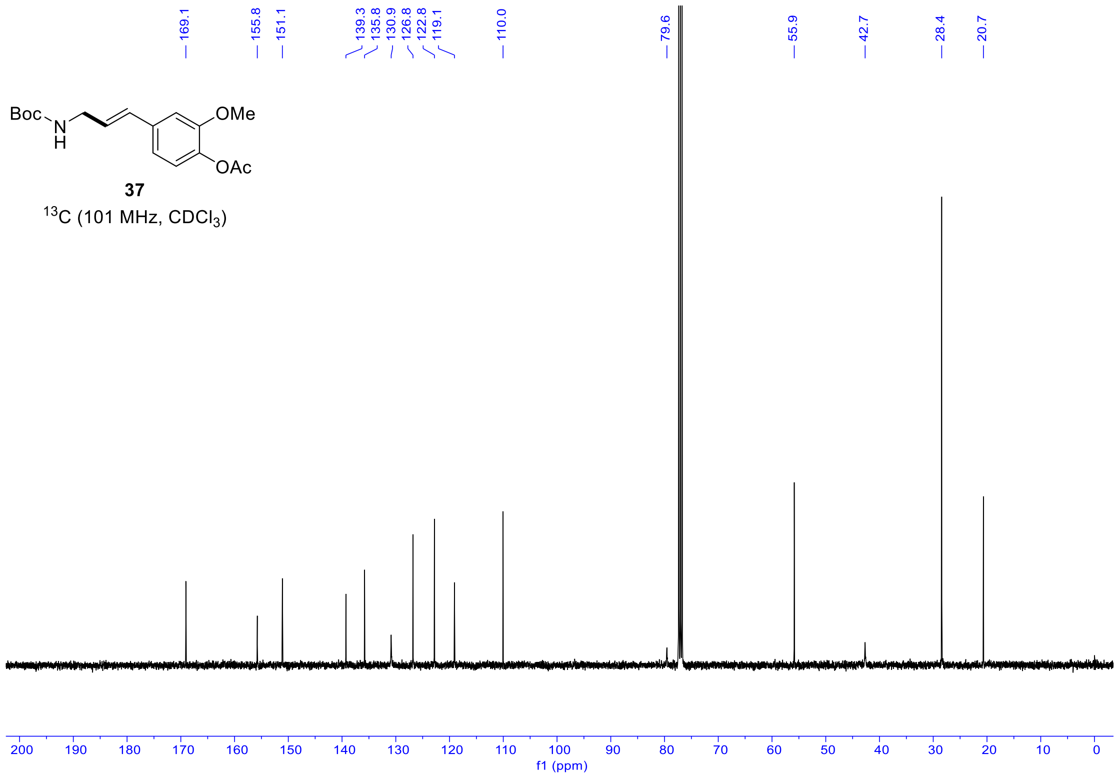

^{1}H NMR spectra of **38** (400 MHz, $CDCl_3$)

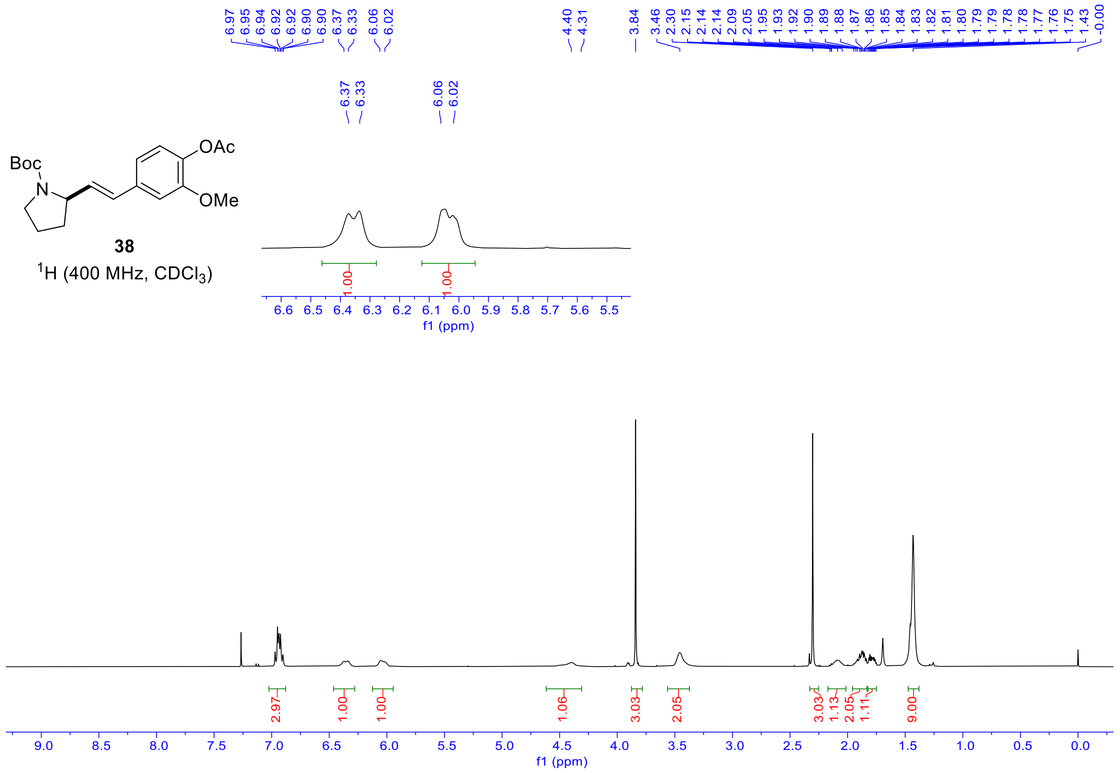

^{13}C NMR spectra of **38** (101 MHz, $CDCl_3$)

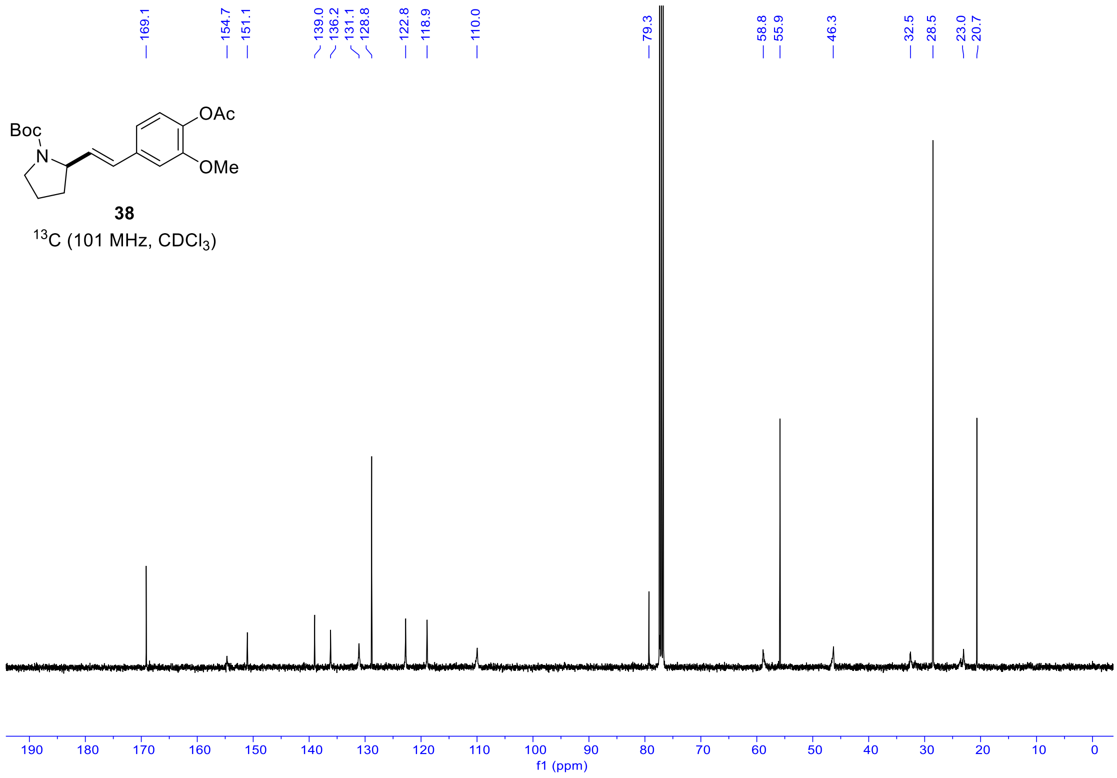

^{1}H NMR spectra of **39** (400 MHz, $CDCl_3$)

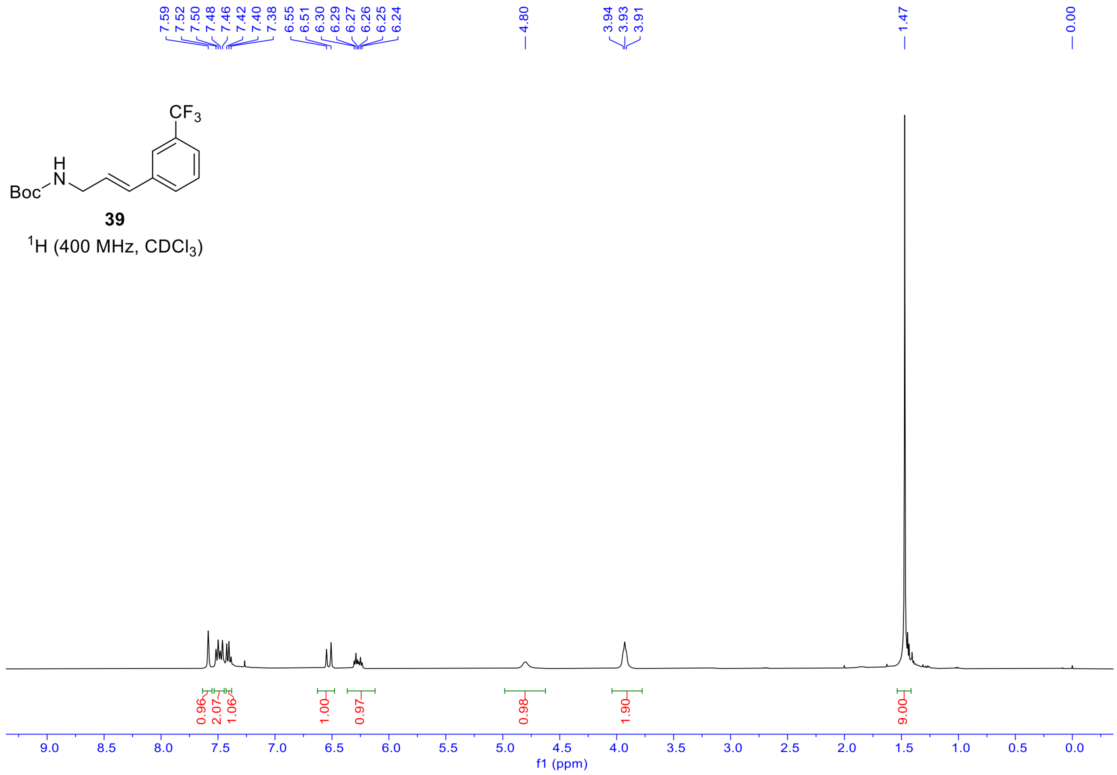

^{13}C NMR spectra of **39** (101 MHz, $CDCl_3$)

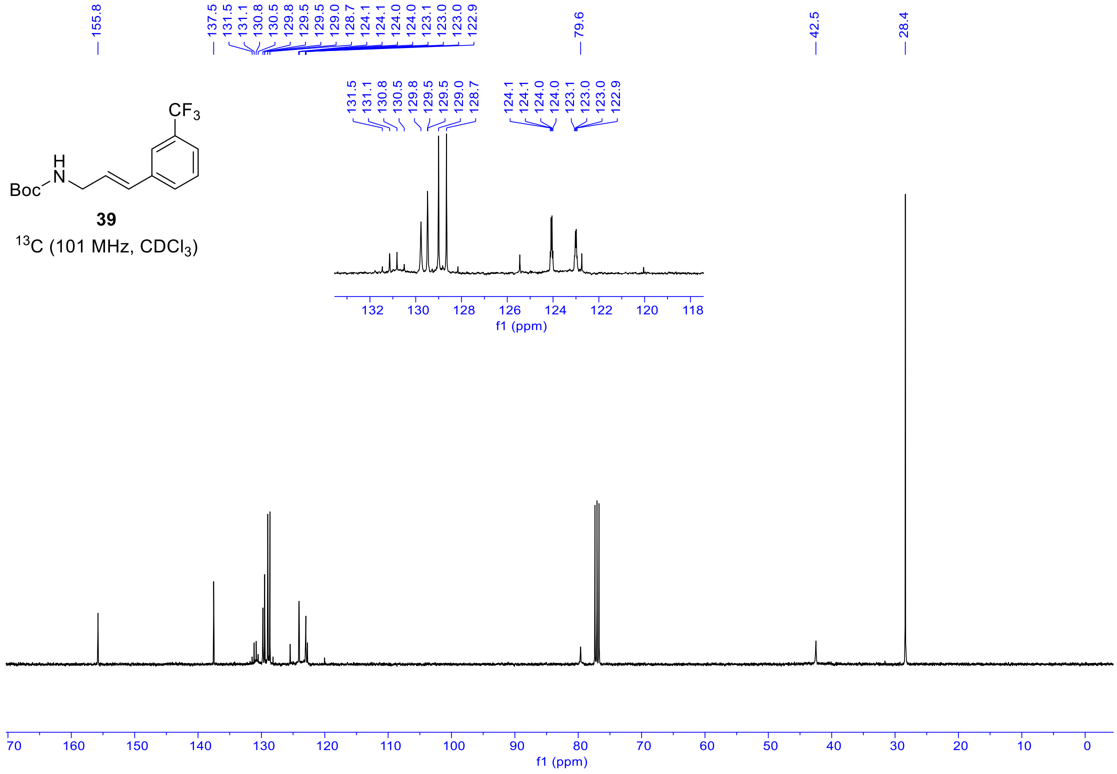

^{19}F NMR spectra of **39** (377 MHz, $CDCl_3$)

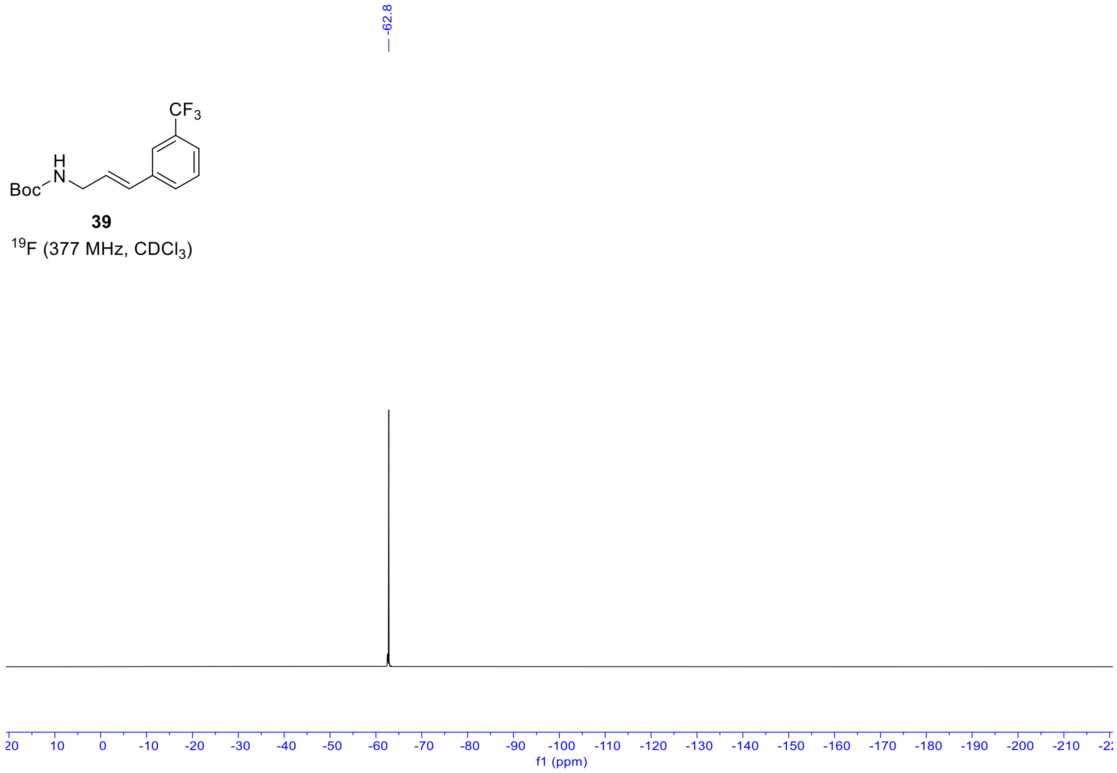

^1H NMR spectra of **40** (400 MHz, $CDCl_3$)

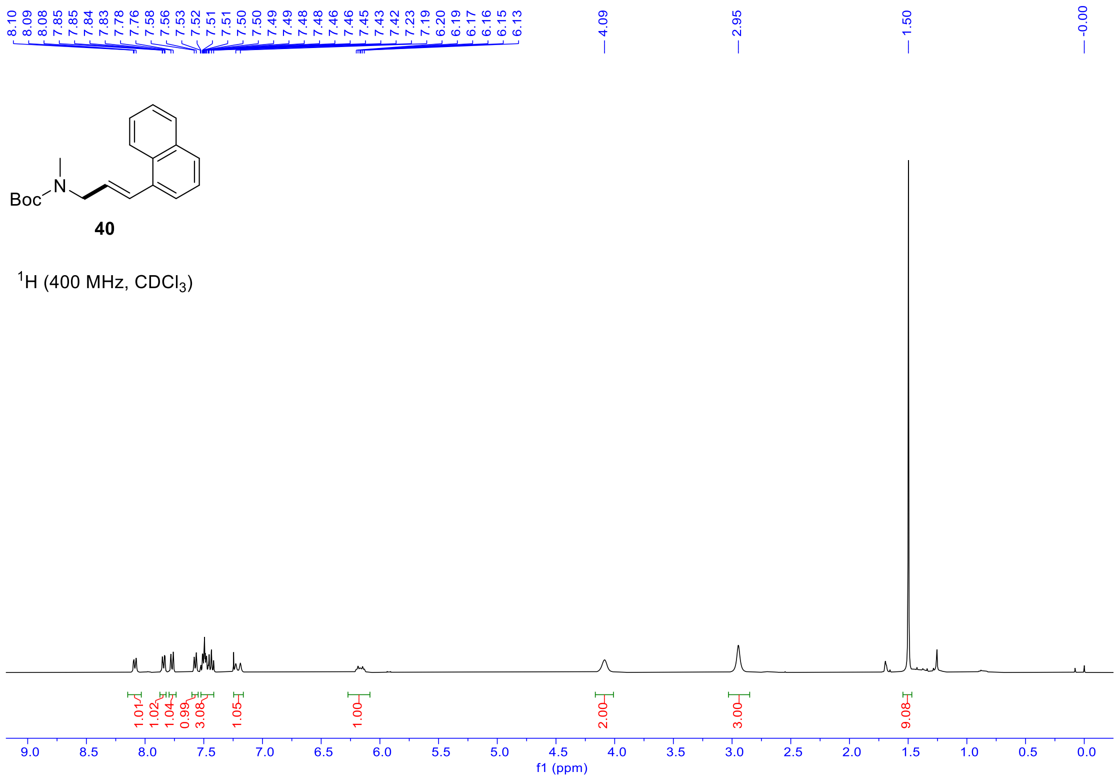

^{13}C NMR spectra of **40** (101MHz, $CDCl_3$)

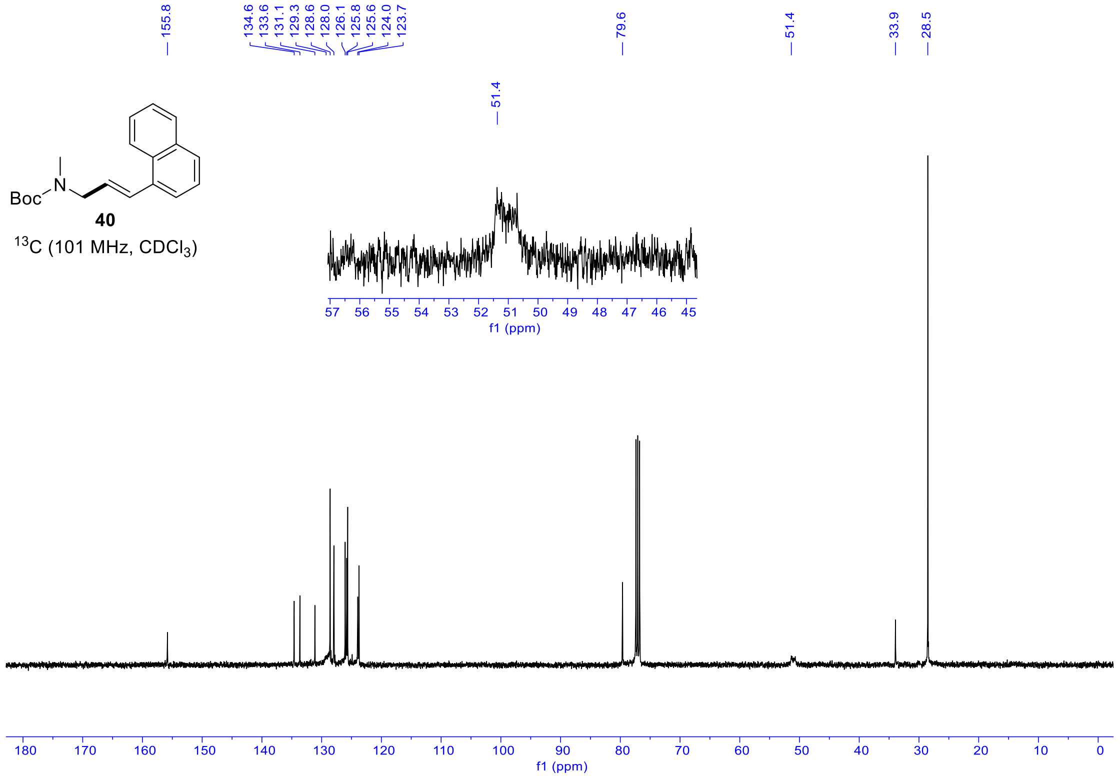

^{1}H NMR spectra of **41** (400 MHz, $CDCl_3$)

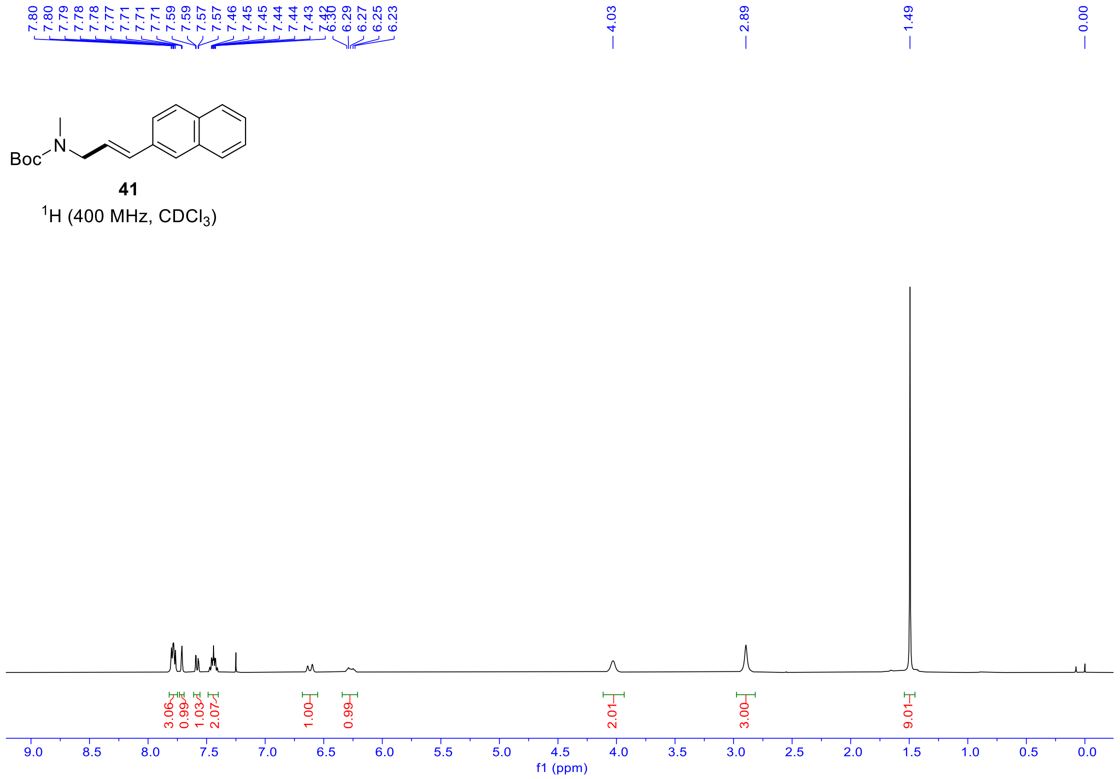

^{13}C NMR spectra of **41** (101 MHz, $CDCl_3$)

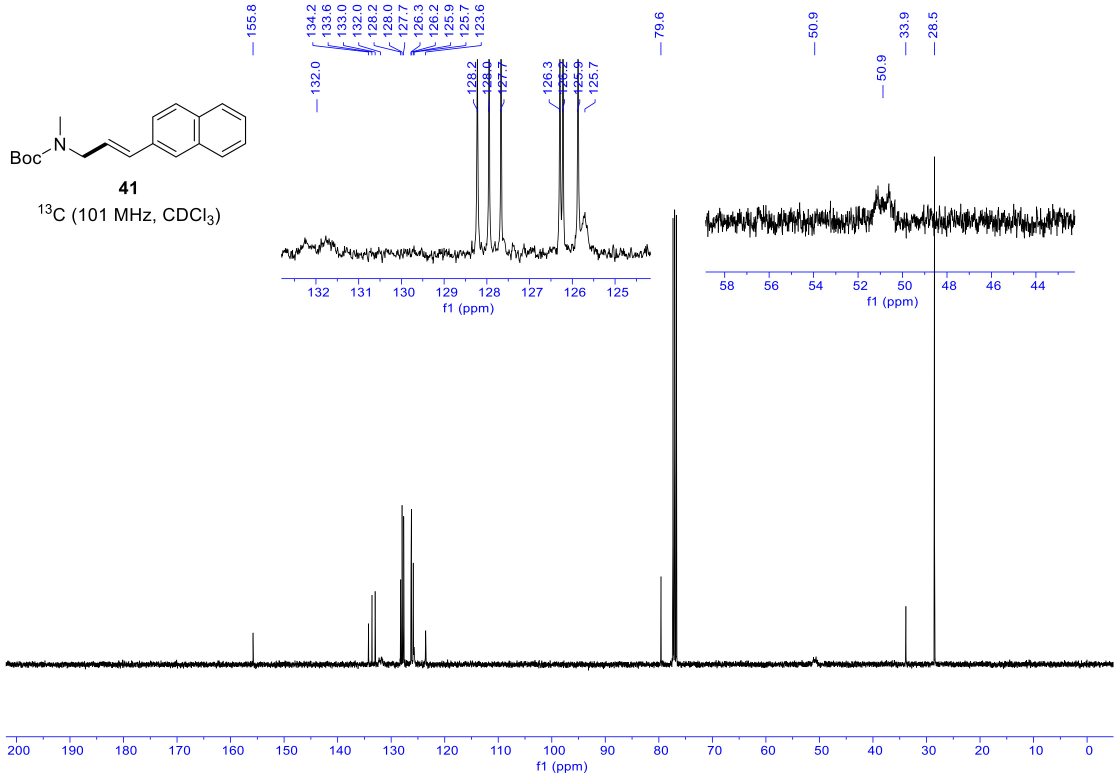

^{1}H NMR spectra of **42** (400 MHz, $CDCl_3$)

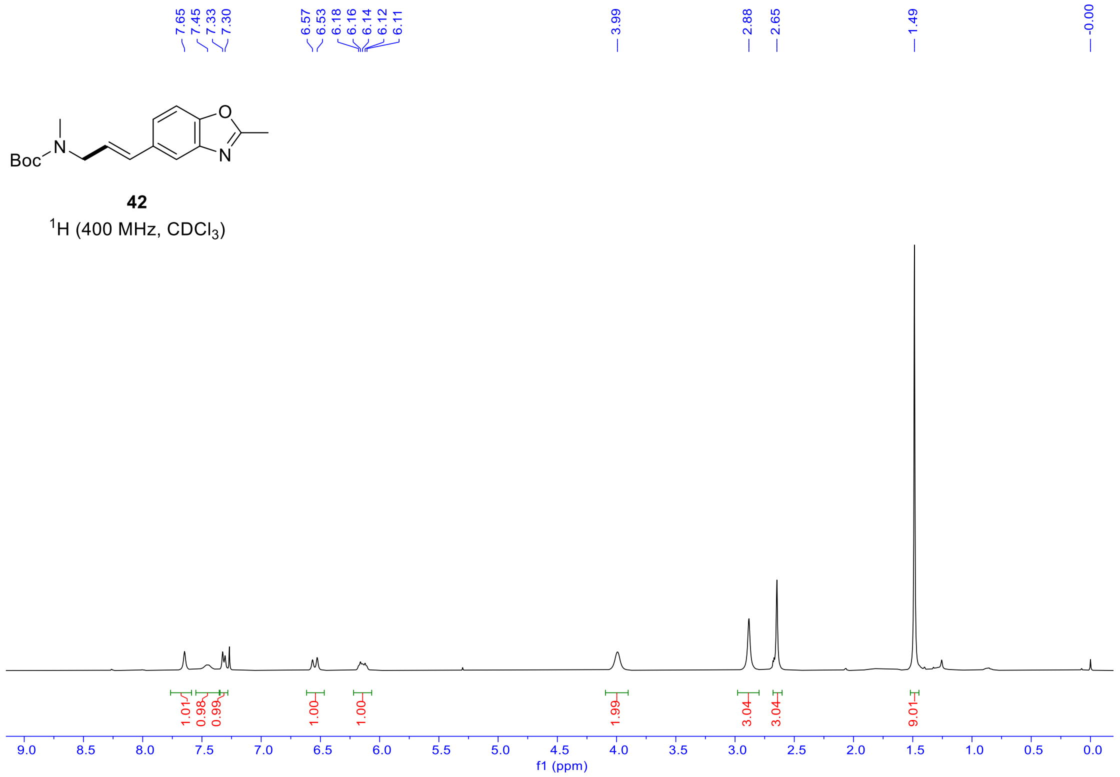

^{13}C NMR spectra of **42** (101 MHz, $CDCl_3$)

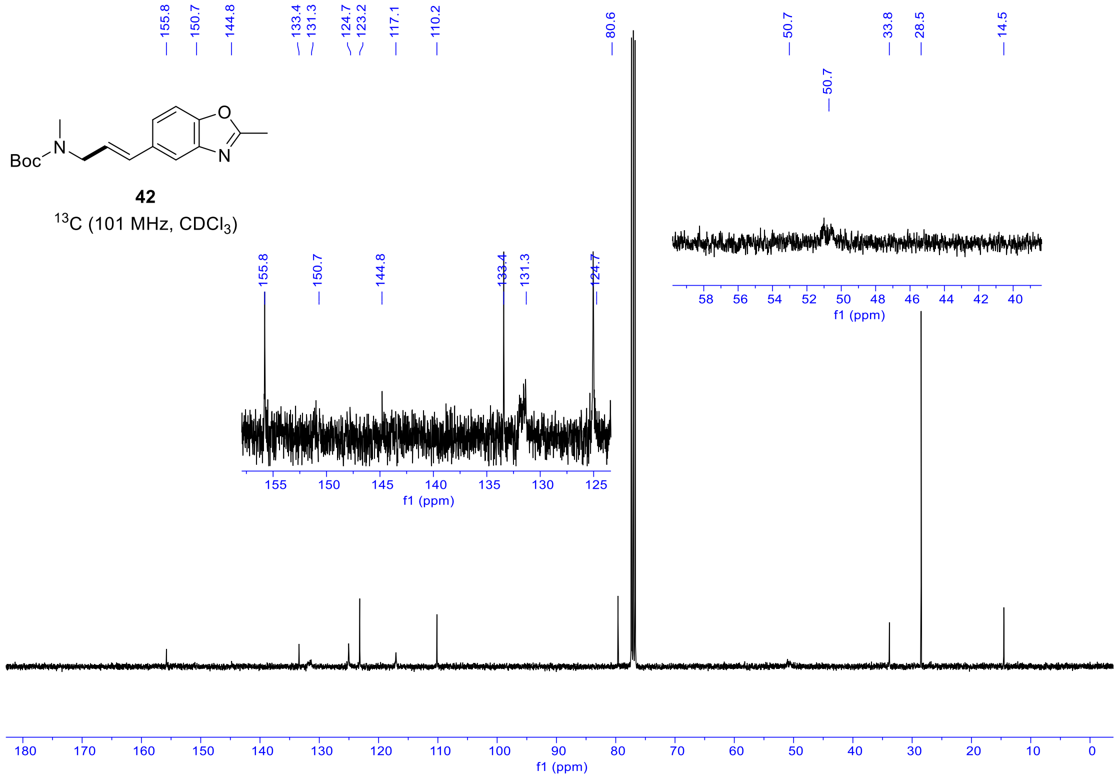

^{1}H NMR spectra of **43** (400 MHz, $CDCl_3$)

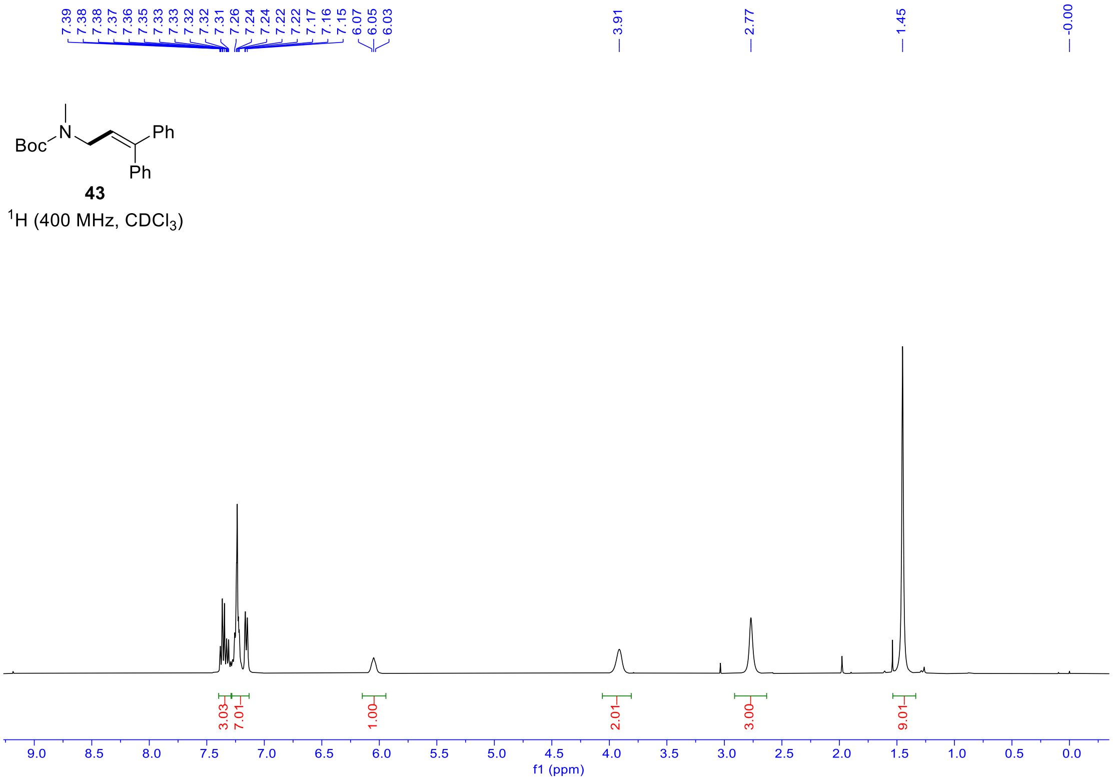

^{13}C NMR spectra of **43** (101 MHz, $CDCl_3$)

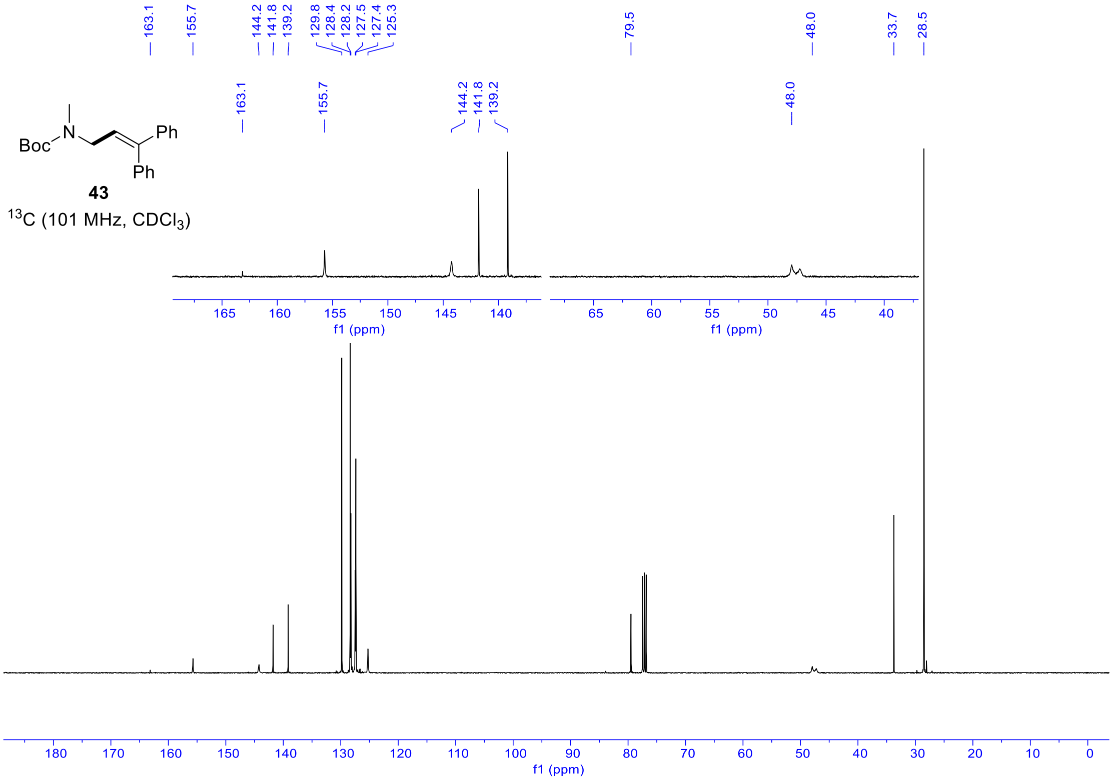

^{1}H NMR spectra of **44** (400 MHz, $CDCl_3$)

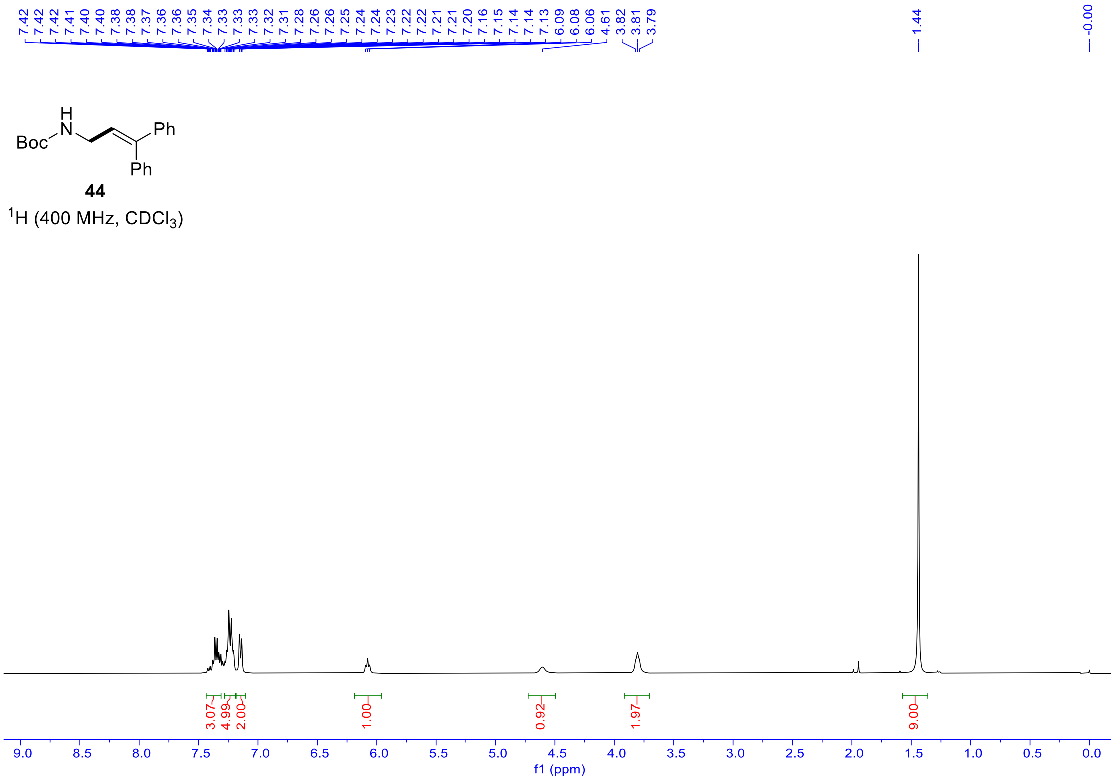

^{13}C NMR spectra of **44** (101 MHz, $CDCl_3$)

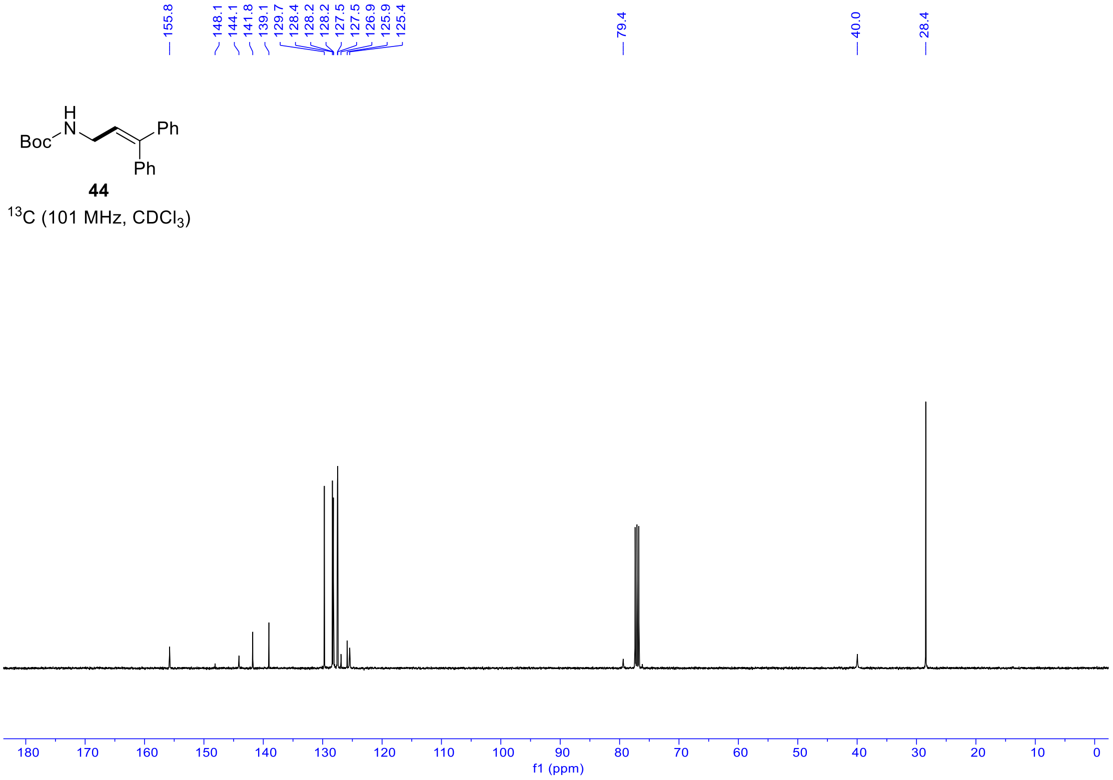

^{1}H NMR spectra of **45** (400 MHz, $CDCl_3$)

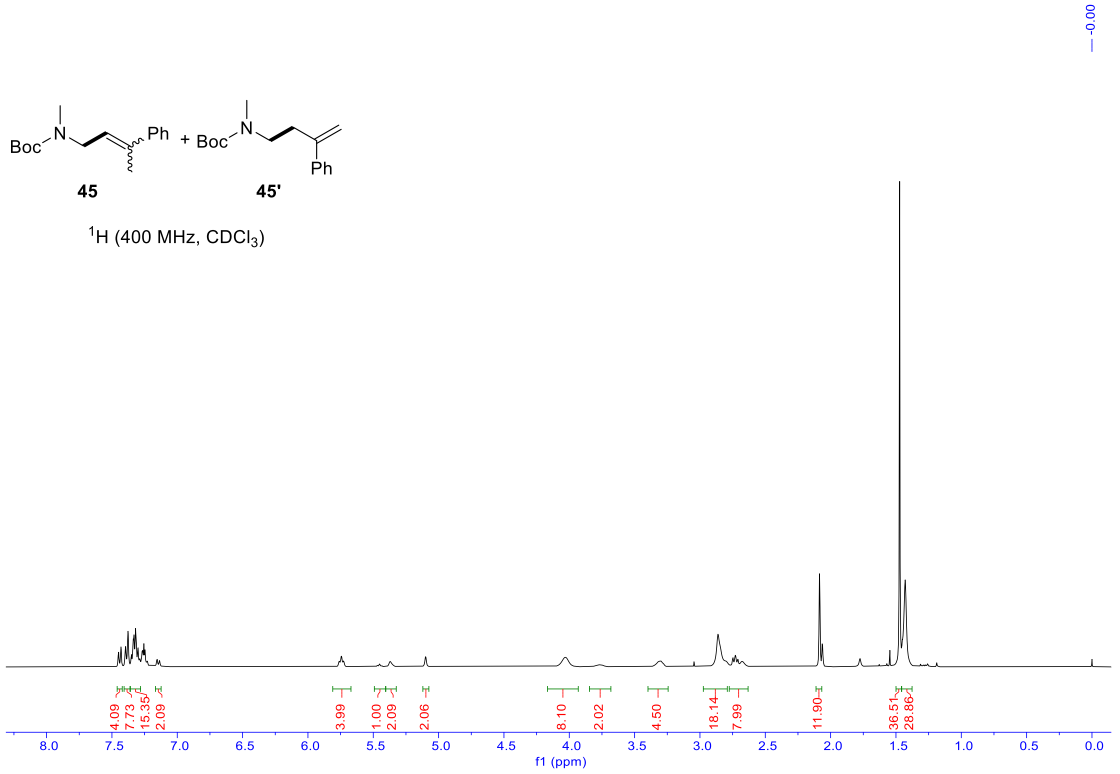

^{1}H NMR spectra of **46** (400 MHz, $CDCl_3$)

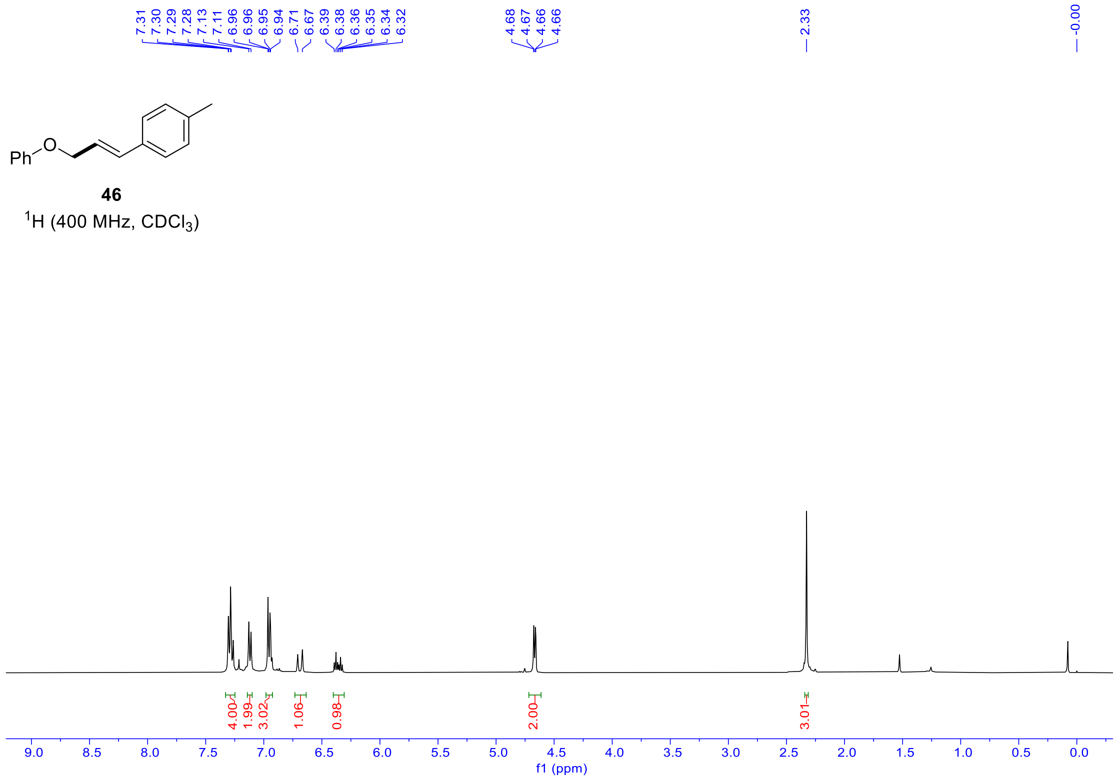

^{13}C NMR spectra of **46** (101 MHz, $CDCl_3$)

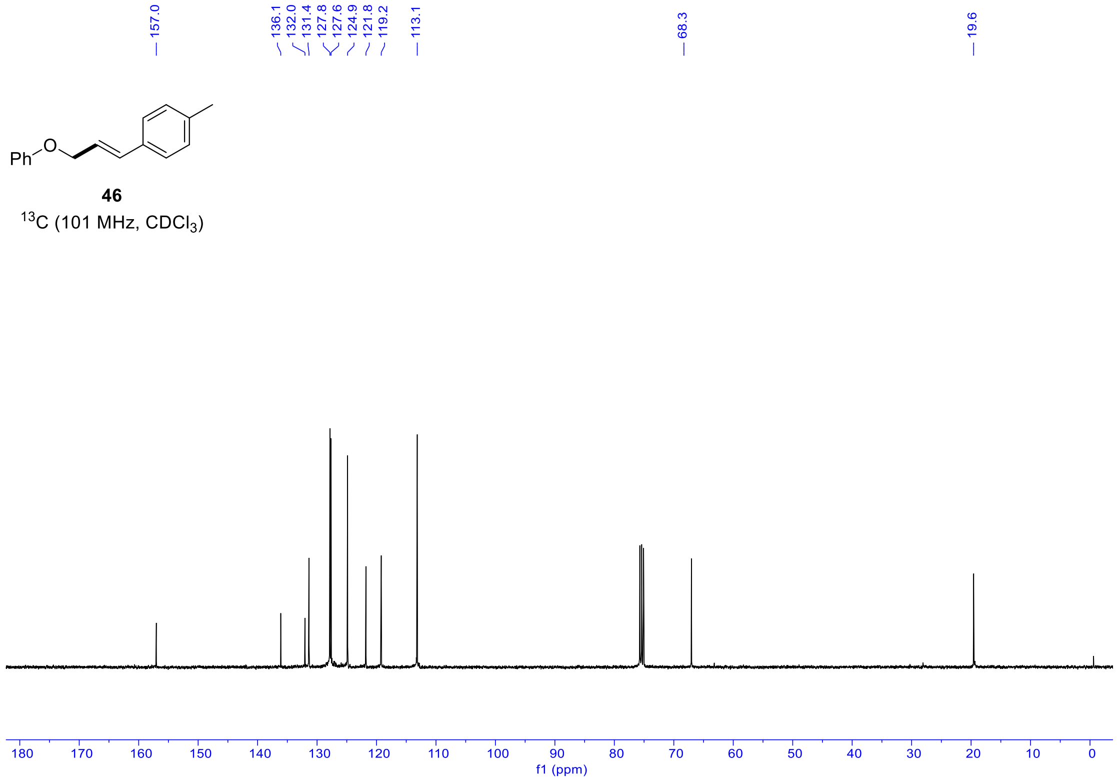

^1H NMR spectra of **47** (400 MHz, $CDCl_3$)

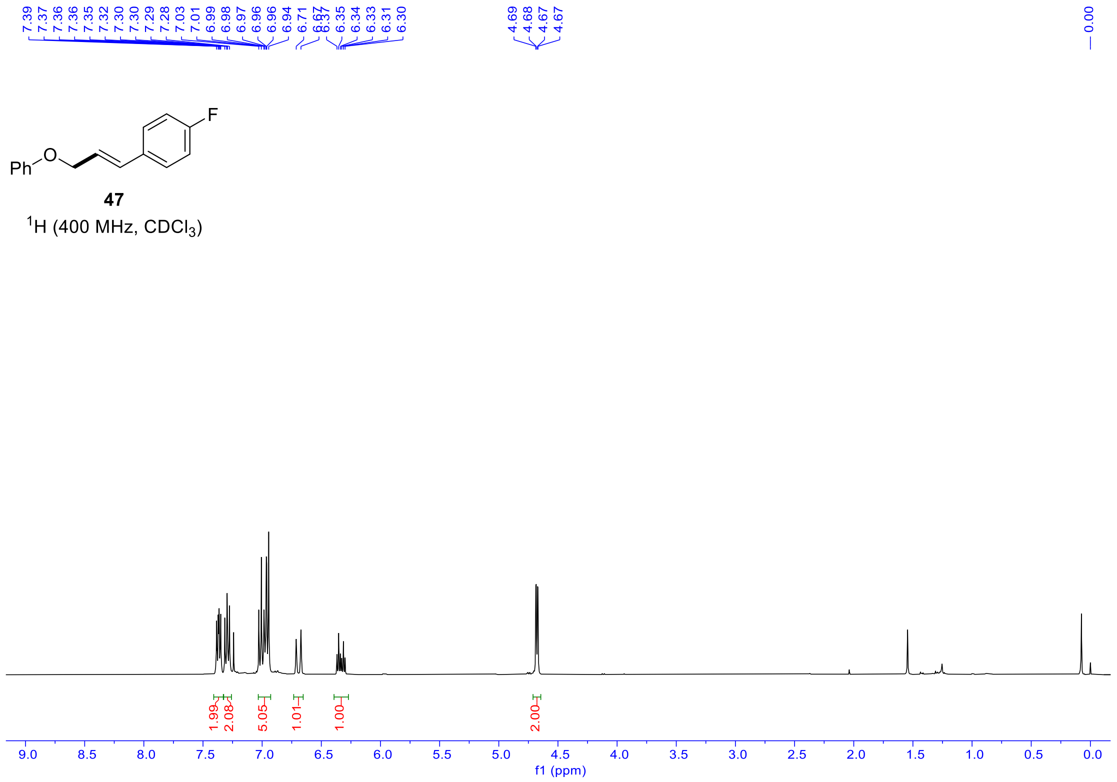

^{13}C NMR spectra of **47** (101 MHz, $CDCl_3$)

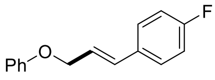

47

^{13}C (101 MHz, $CDCl_3$)

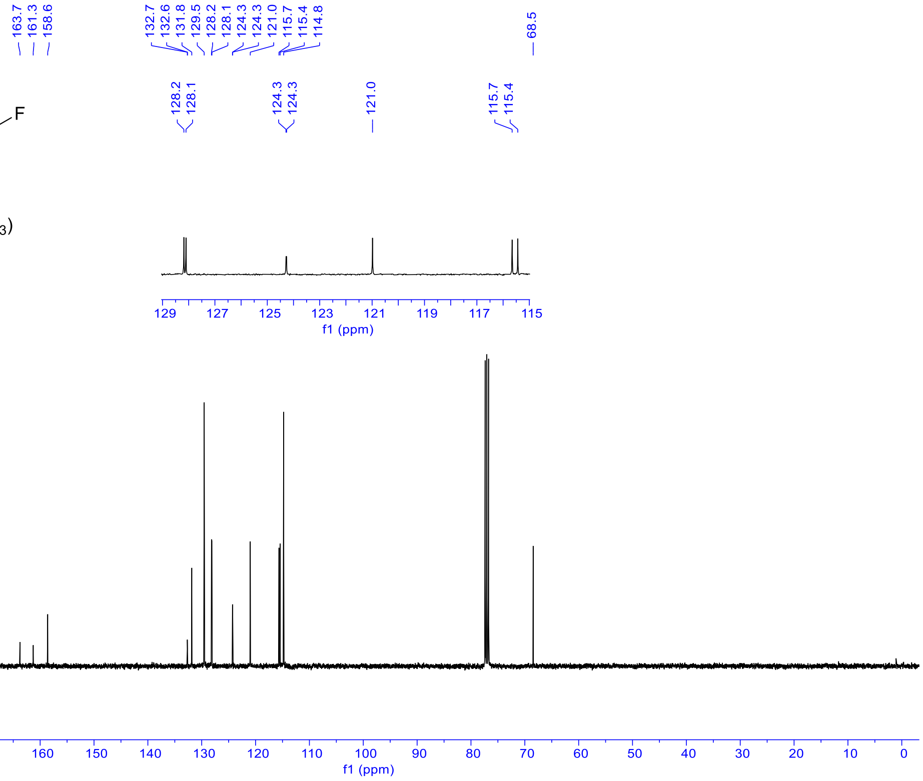

^{19}F NMR spectra of **47** (377 MHz, $CDCl_3$)

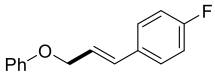

47

^{19}F (377 MHz, $CDCl_3$)

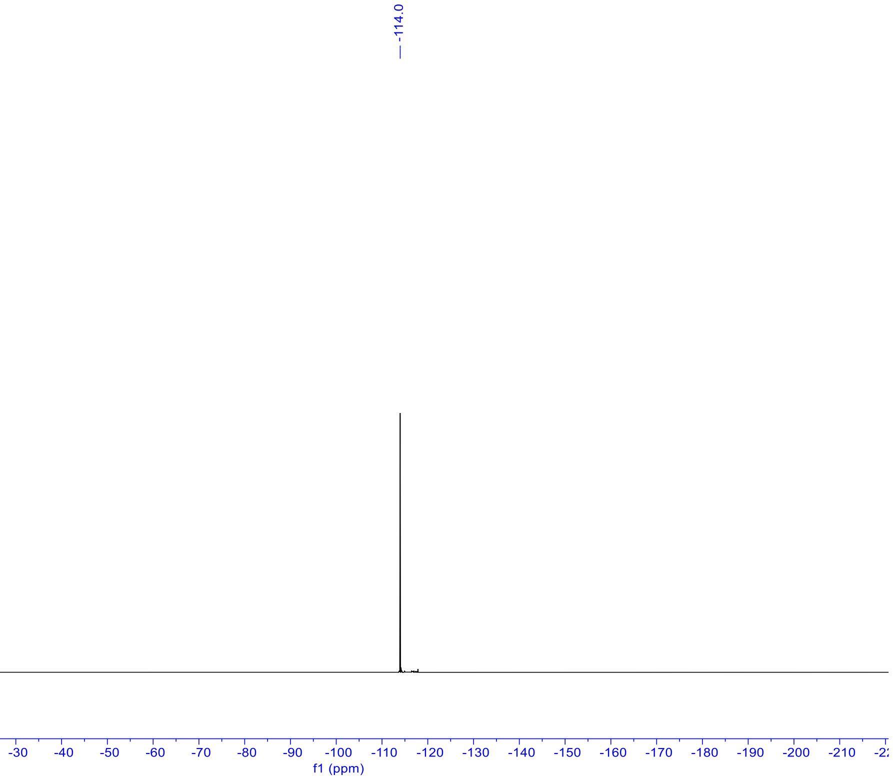

^{1}H NMR spectra of **48** (400 MHz, $CDCl_3$)

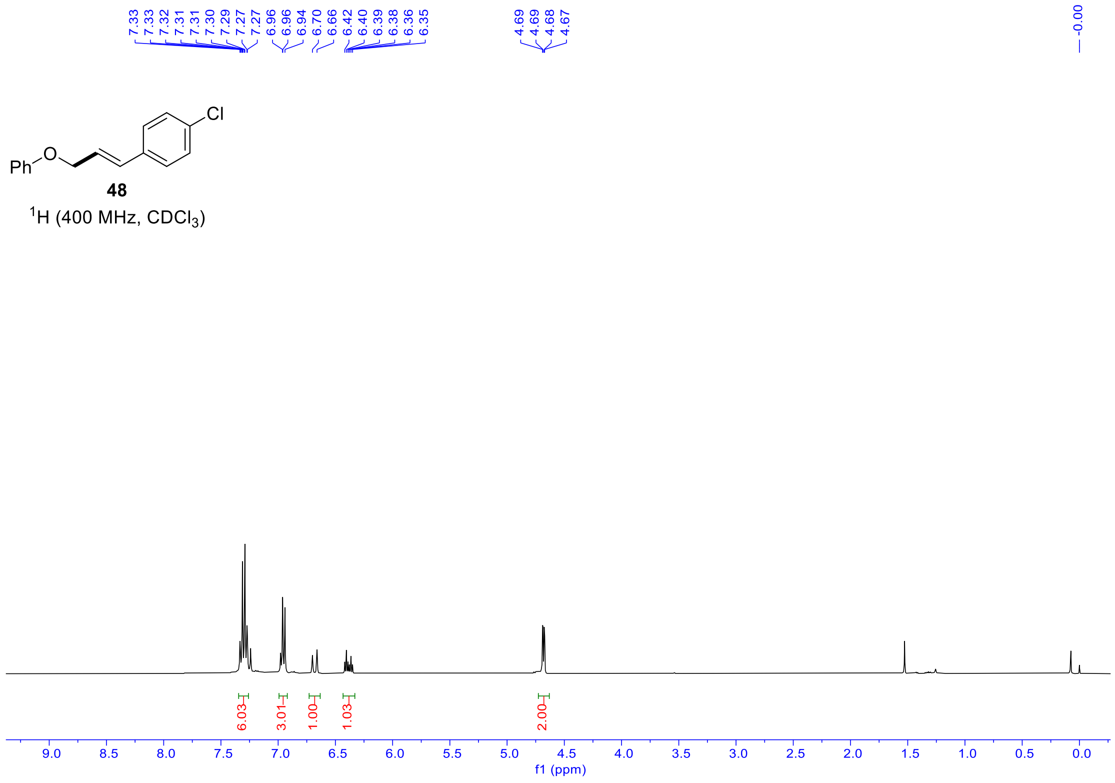

^{13}C NMR spectra of **48** (101MHz, $CDCl_3$)

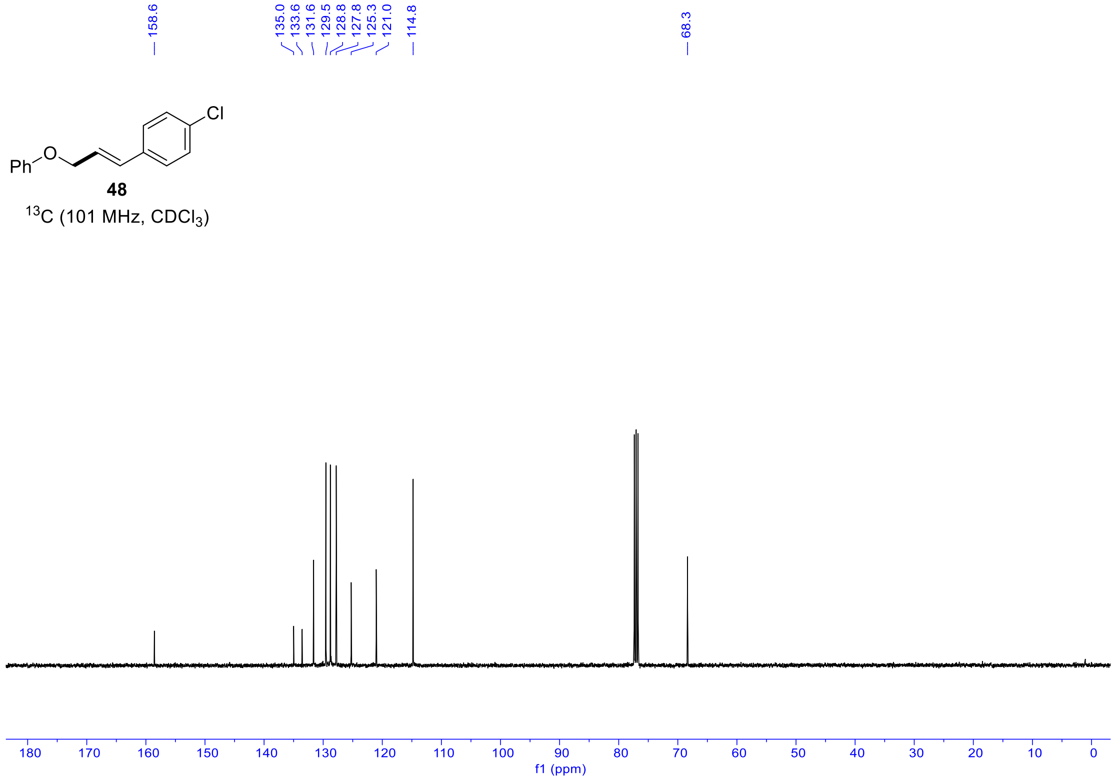

^{1}H NMR spectra of **49** (400 MHz, $CDCl_3$)

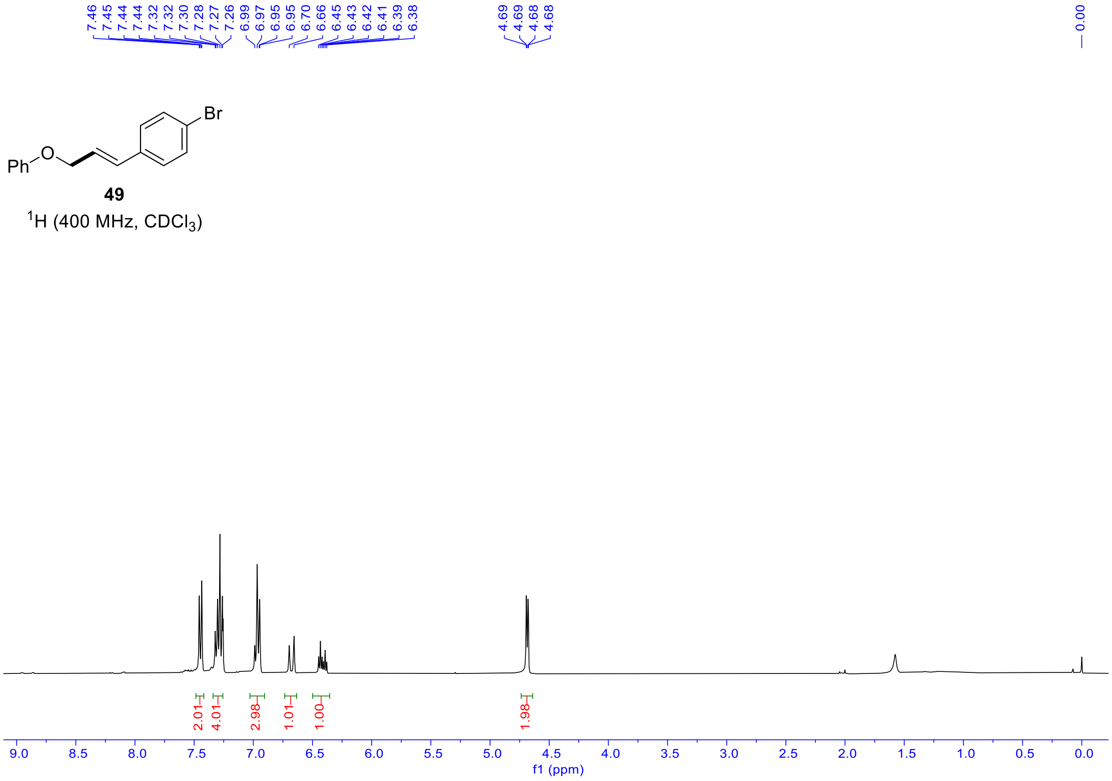

^{13}C NMR spectra of **49** (101 MHz, $CDCl_3$)

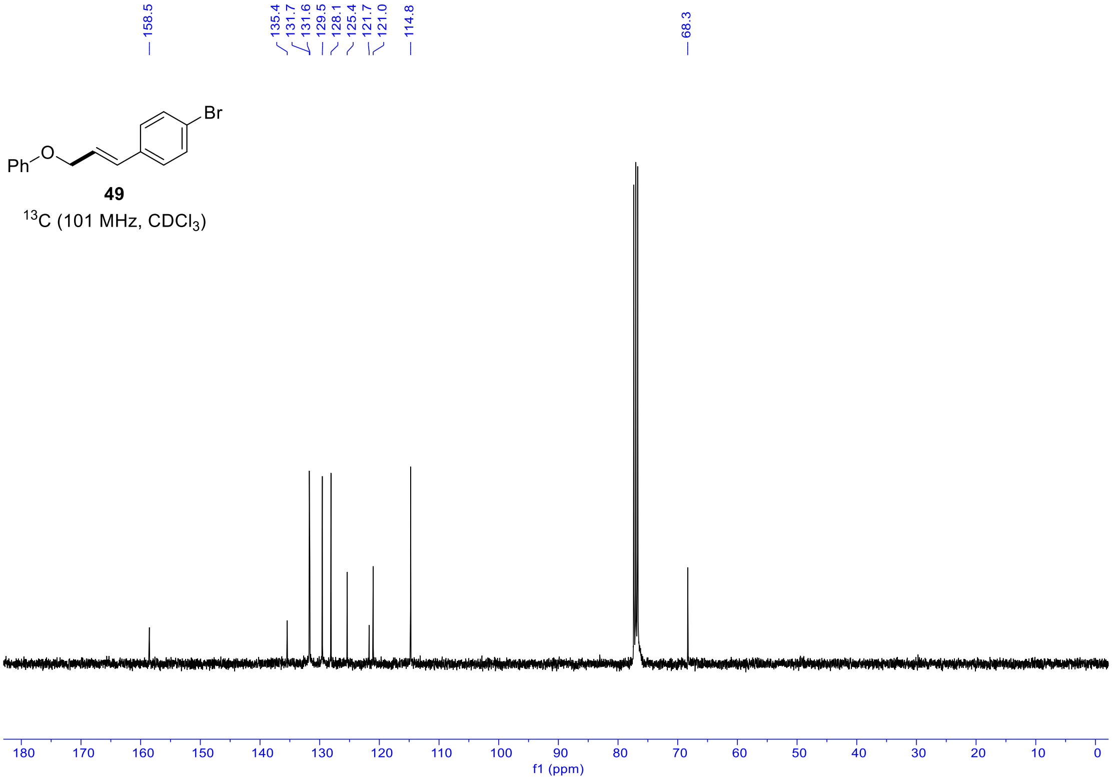

^{1}H NMR spectra of **50** (400 MHz, $CDCl_3$)

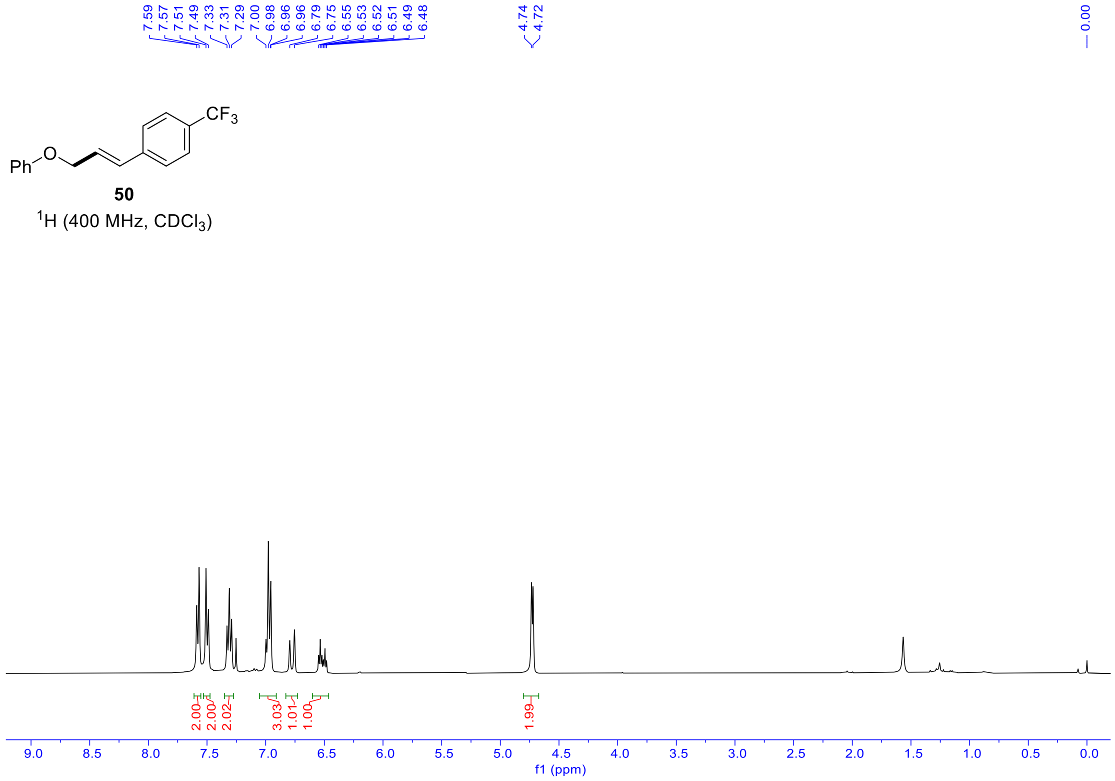

^{13}C NMR spectra of **50** (101 MHz, $CDCl_3$)

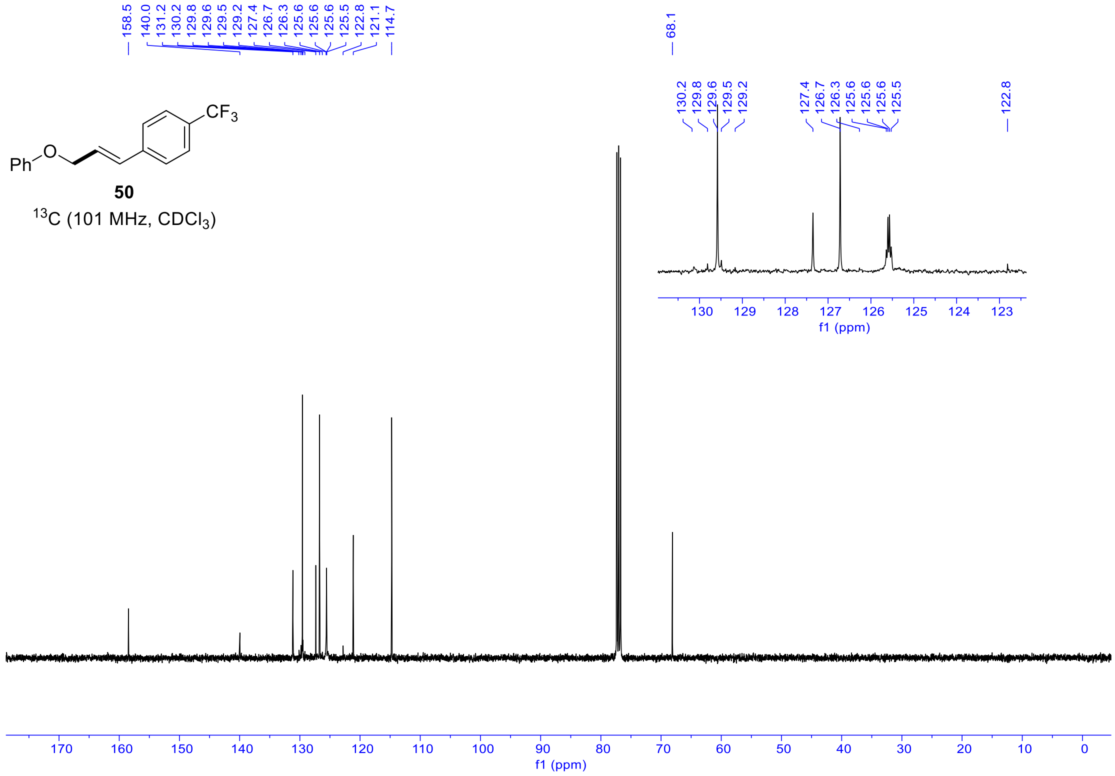

^{19}F NMR spectra of **50** (377 MHz, $CDCl_3$)

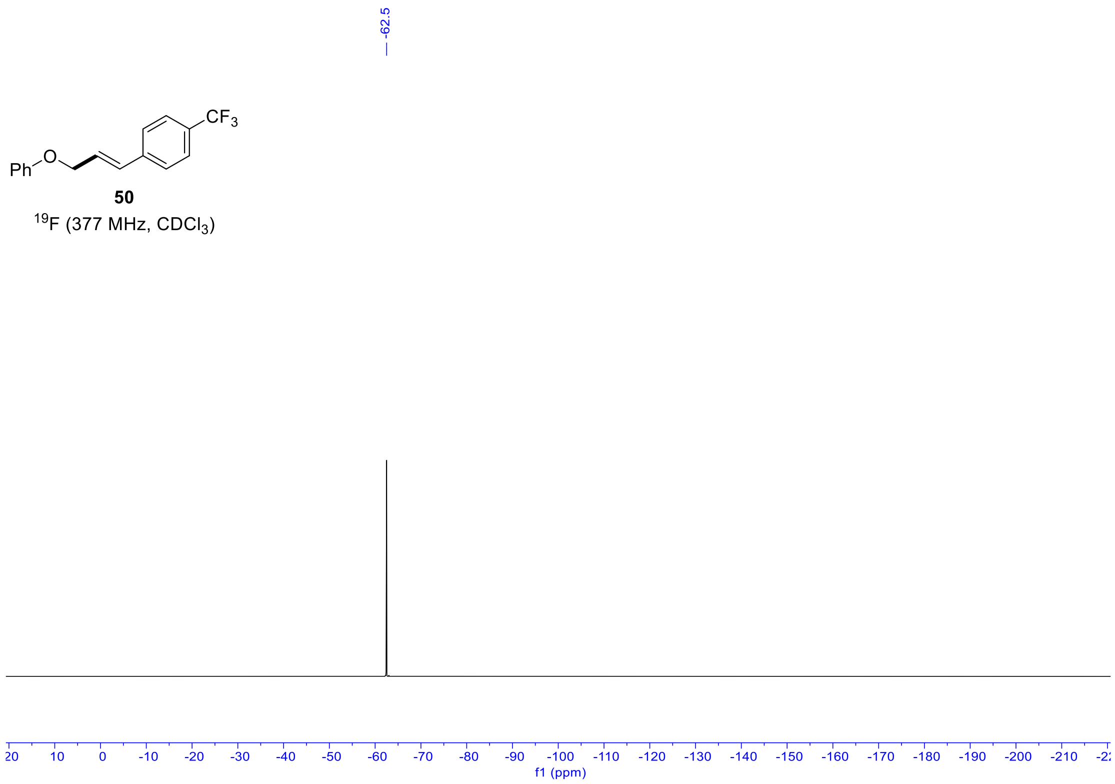

^{1}H NMR spectra of **51** (400 MHz, $CDCl_3$)

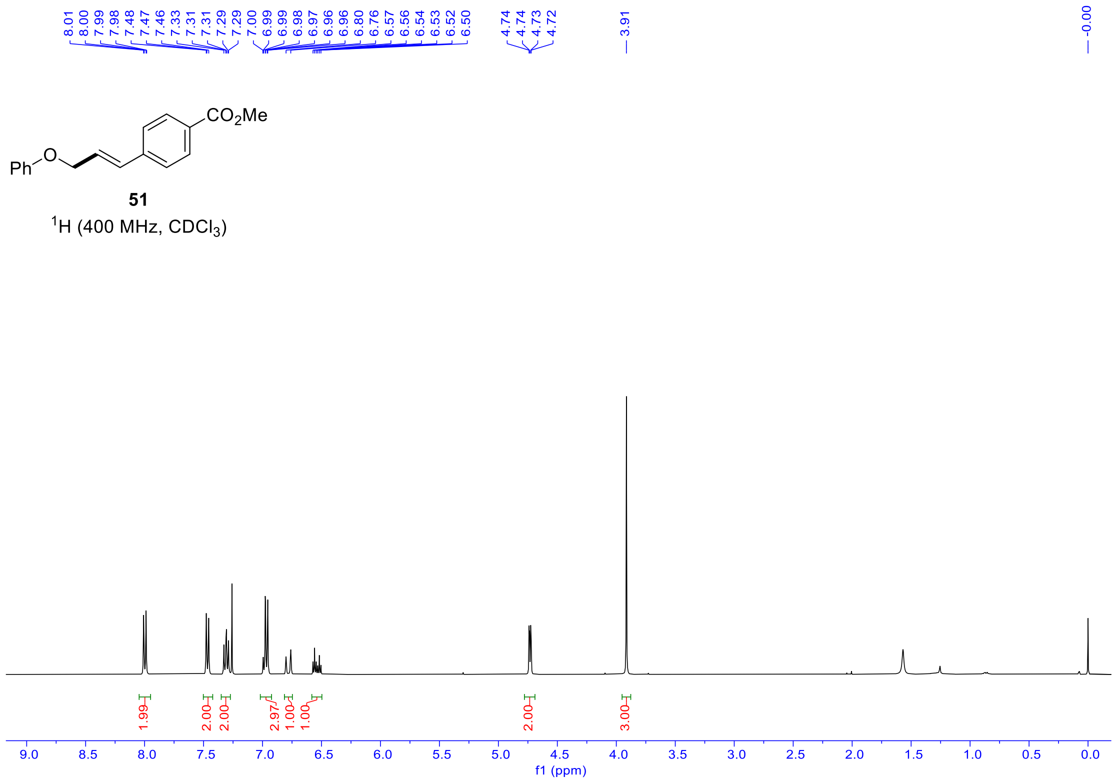

[13] C NMR spectra of **51** (101 MHz, $CDCl_3$)

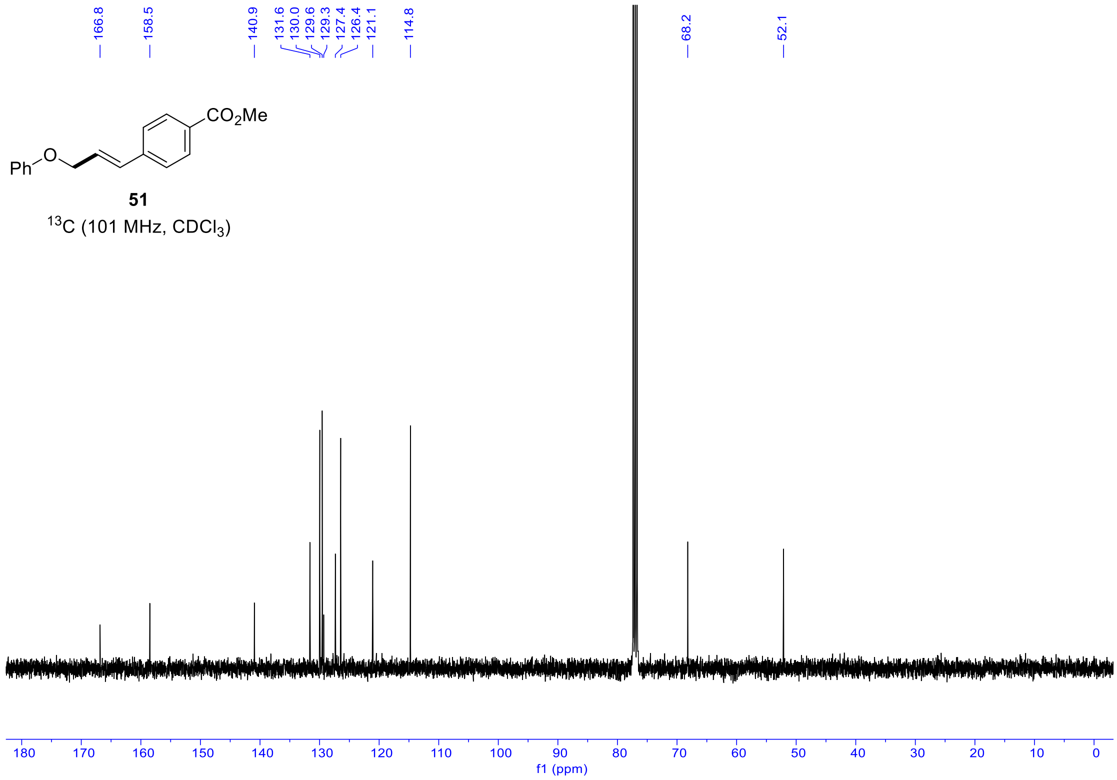

[1] H NMR spectra of **52** (400 MHz, $CDCl_3$)

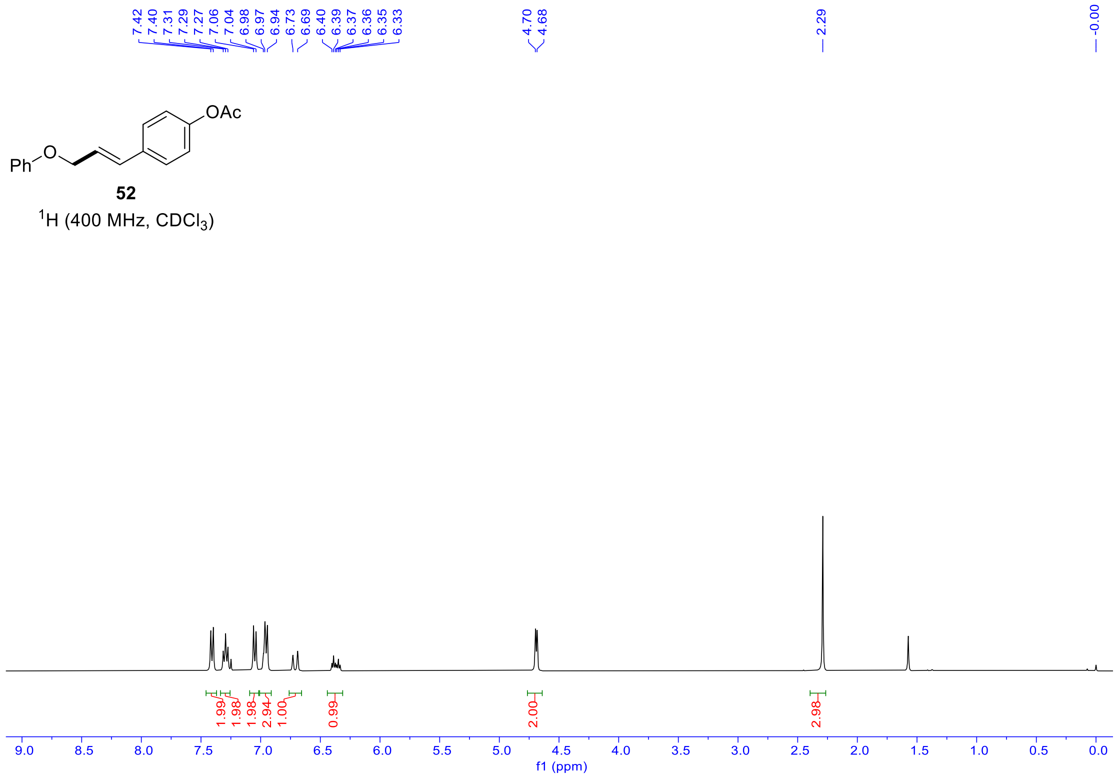

^{13}C NMR spectra of **52** (101 MHz, $CDCl_3$)

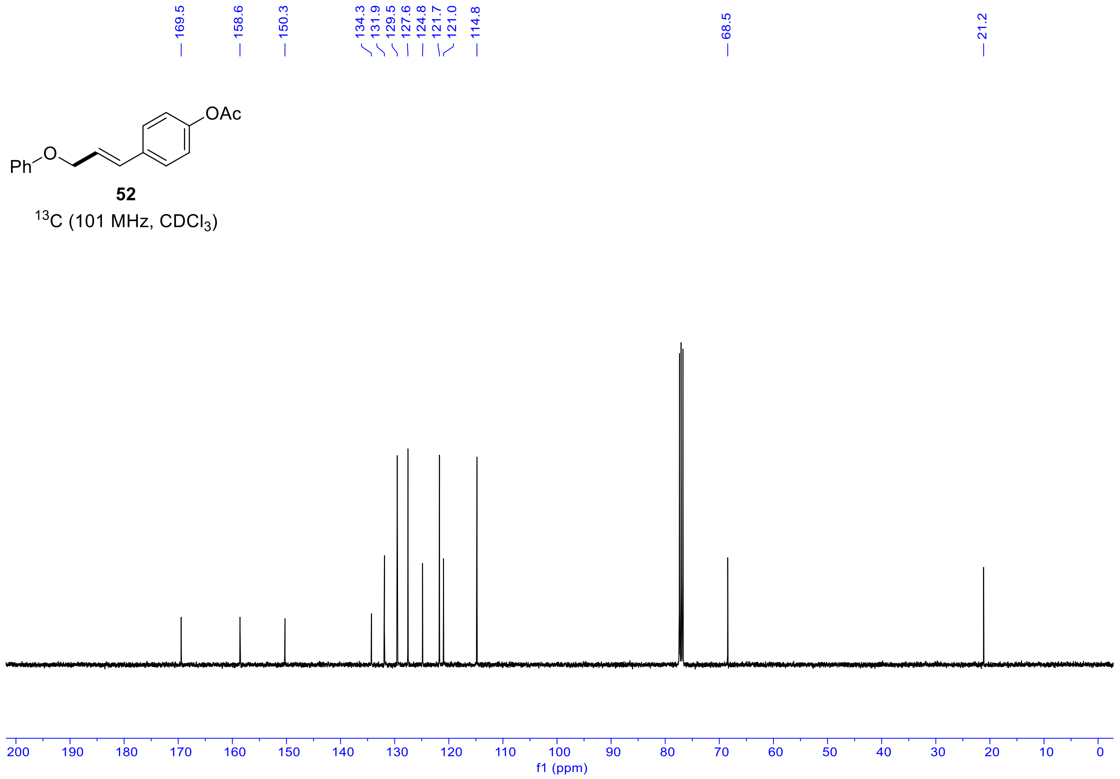

^{1}H NMR spectra of **53** (400 MHz, $CDCl_3$)

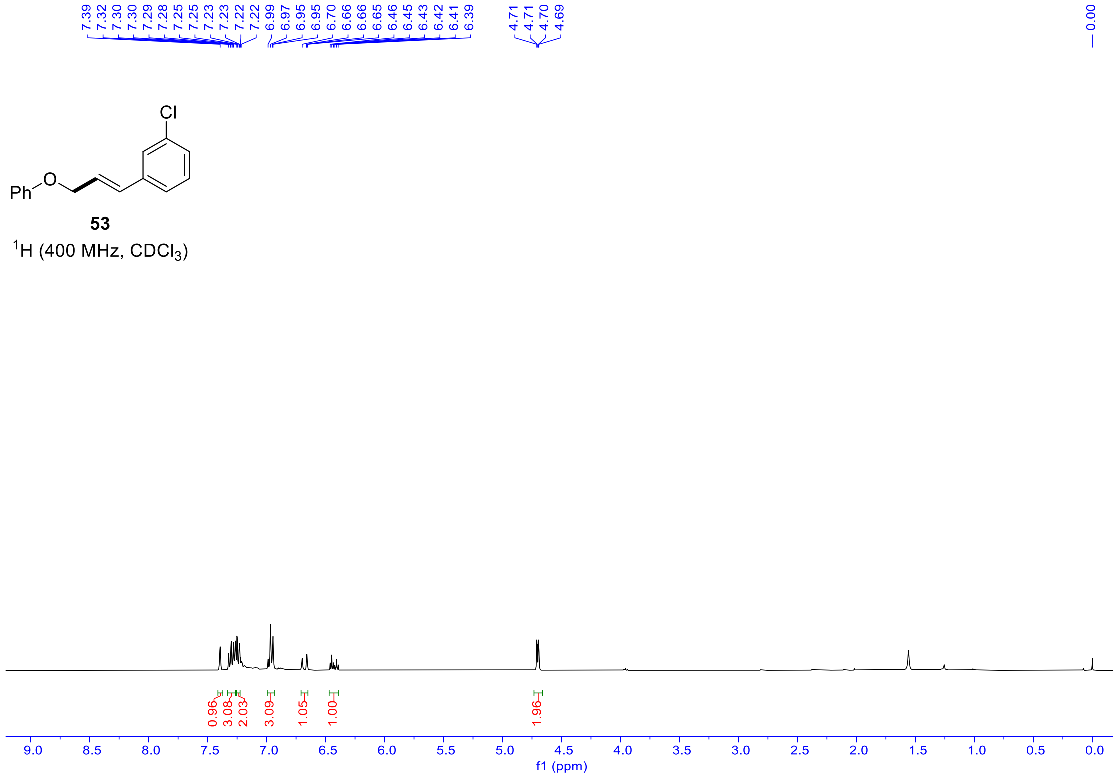

^{13}C NMR spectra of **53** (101 MHz, $CDCl_3$)

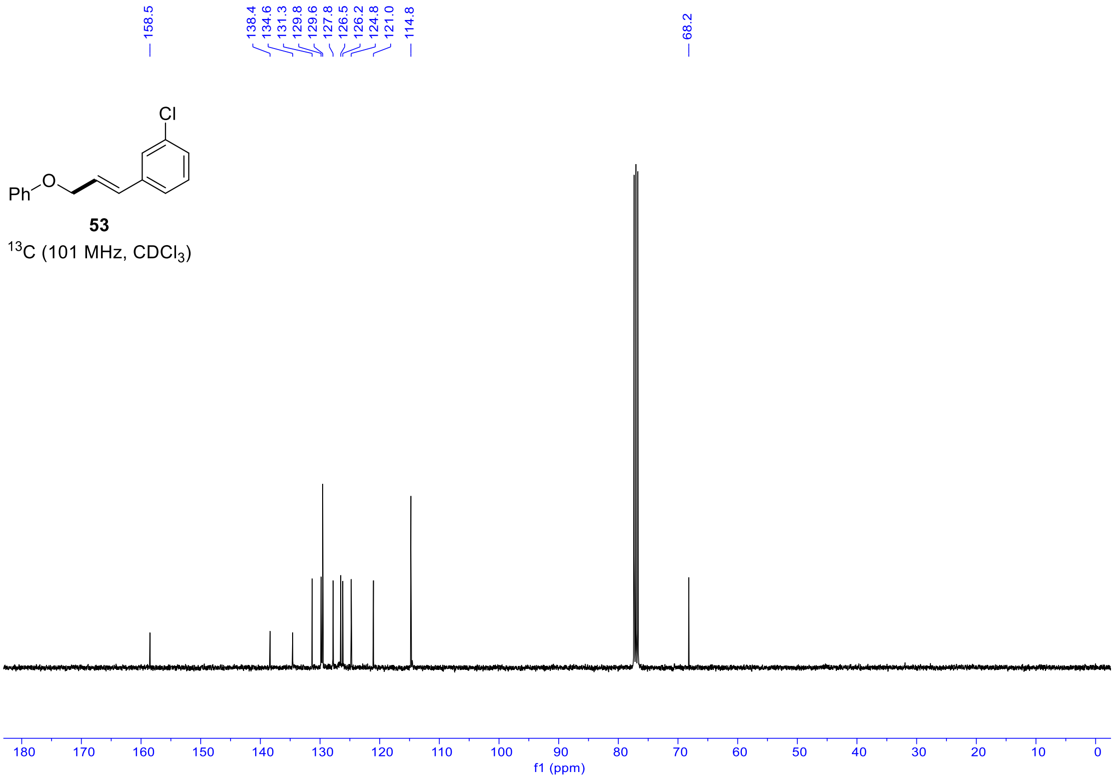

^{1}H NMR spectra of **54** (400 MHz, $CDCl_3$)

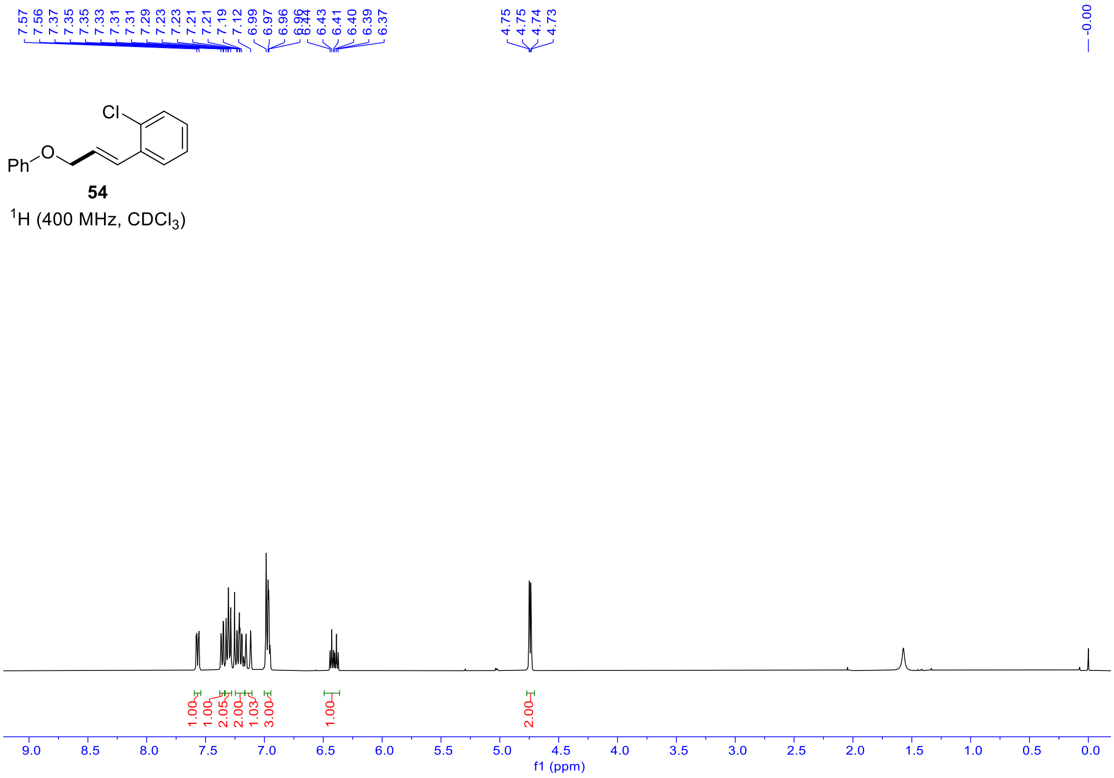

^{13}C NMR spectra of **54** (101 MHz, $CDCl_3$)

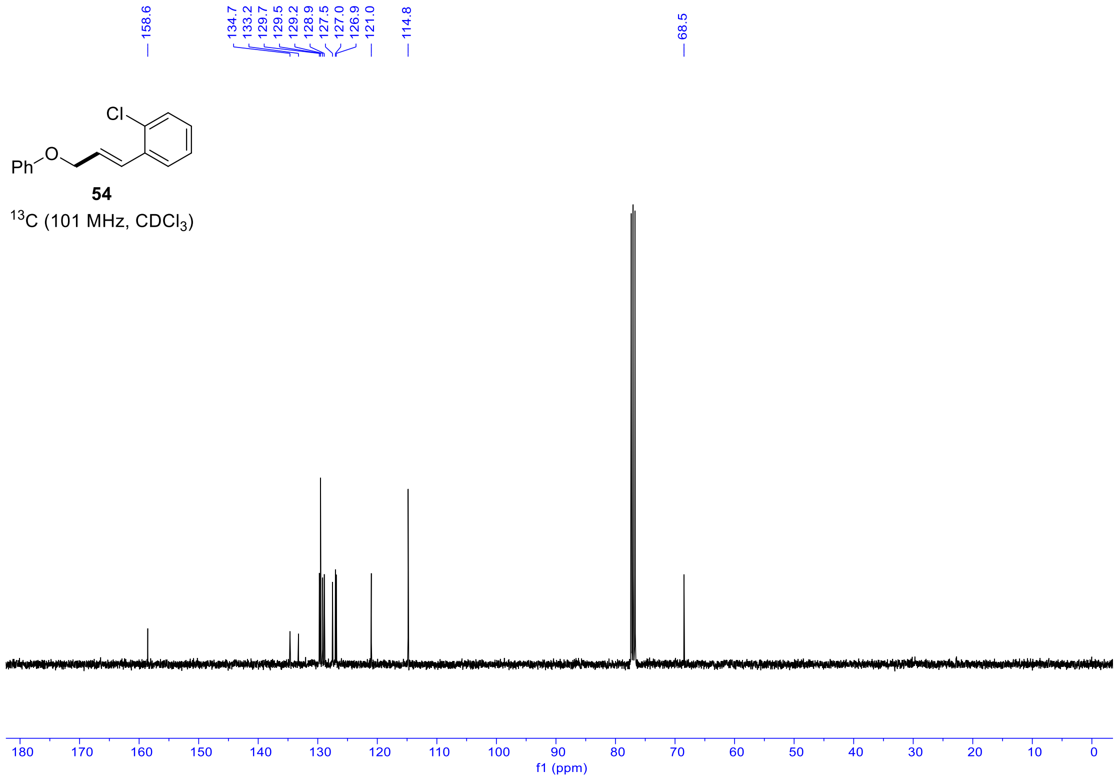

^{1}H NMR spectra of **55** (400 MHz, $CDCl_3$)

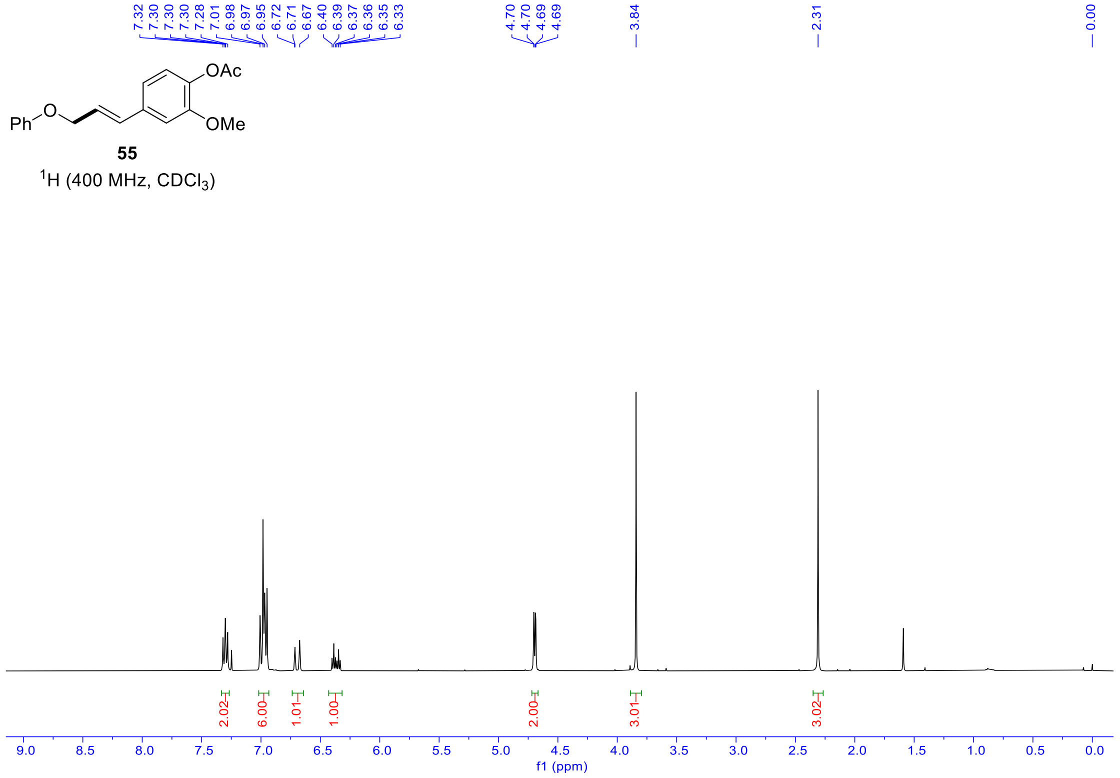

^{13}C NMR spectra of **55** (101 MHz, CDCl$_3$)

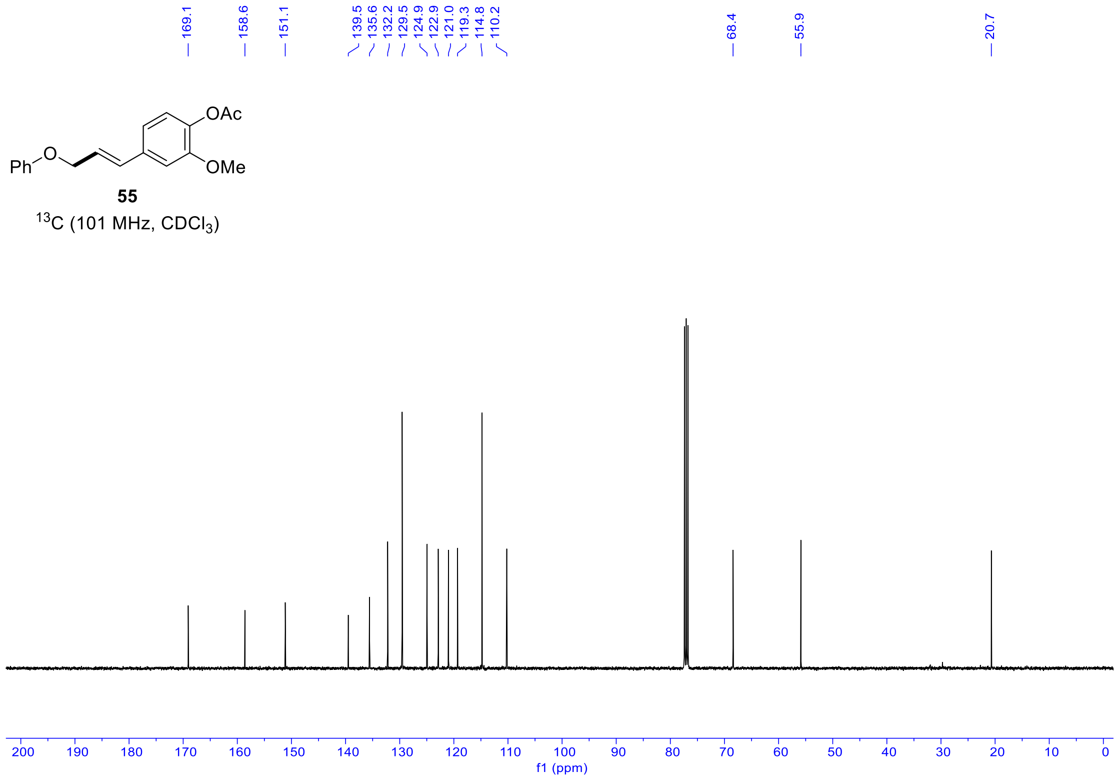

^{1}H NMR spectra of **56** (400 MHz, CDCl$_3$)

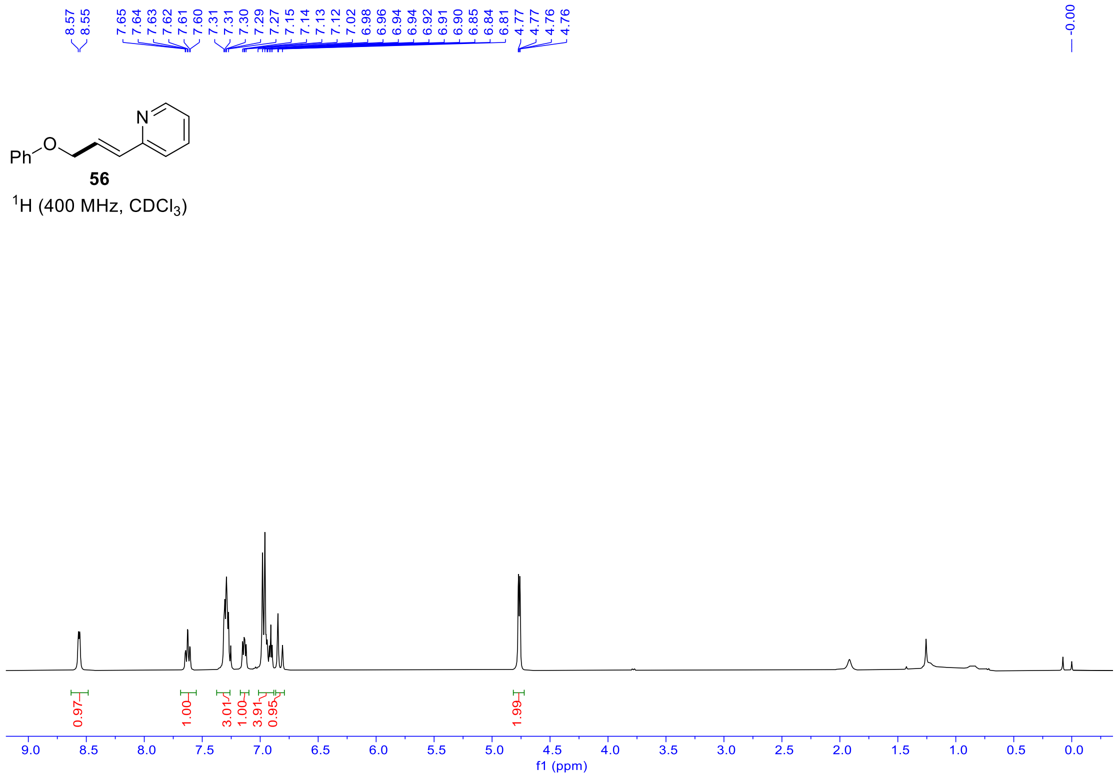

^{13}C NMR spectra of **56** (101 MHz, $CDCl_3$)

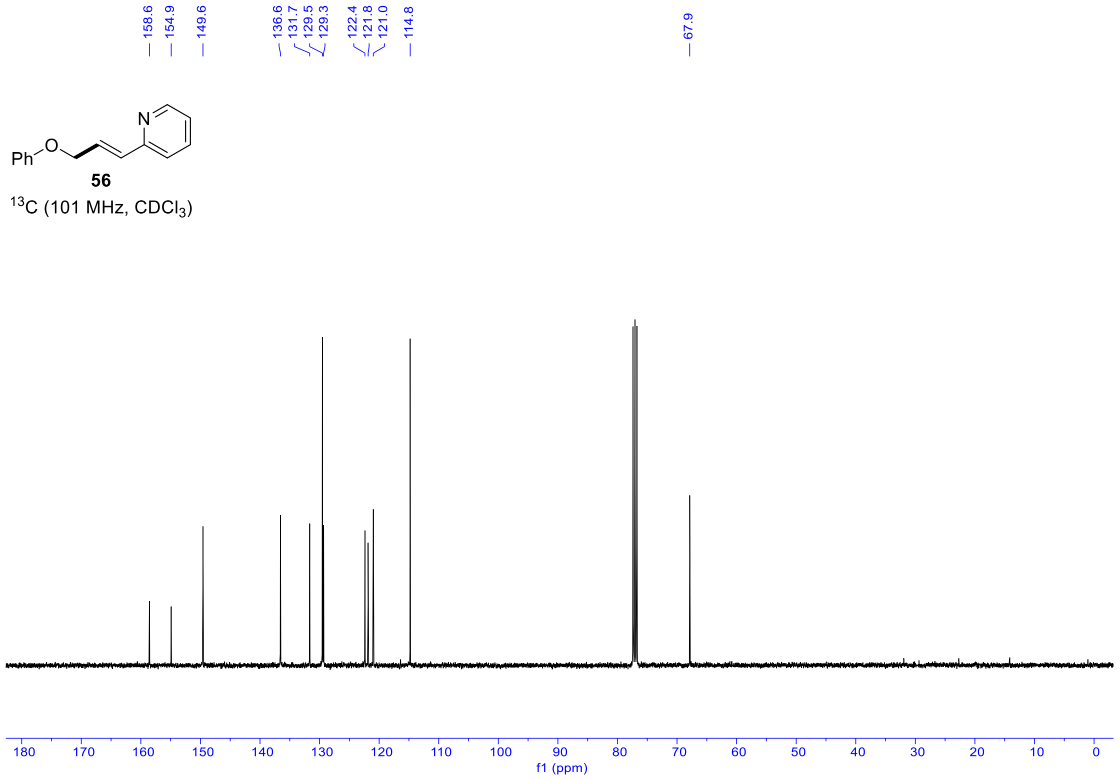

^{1}H NMR spectra of **57** (400 MHz, $CDCl_3$)

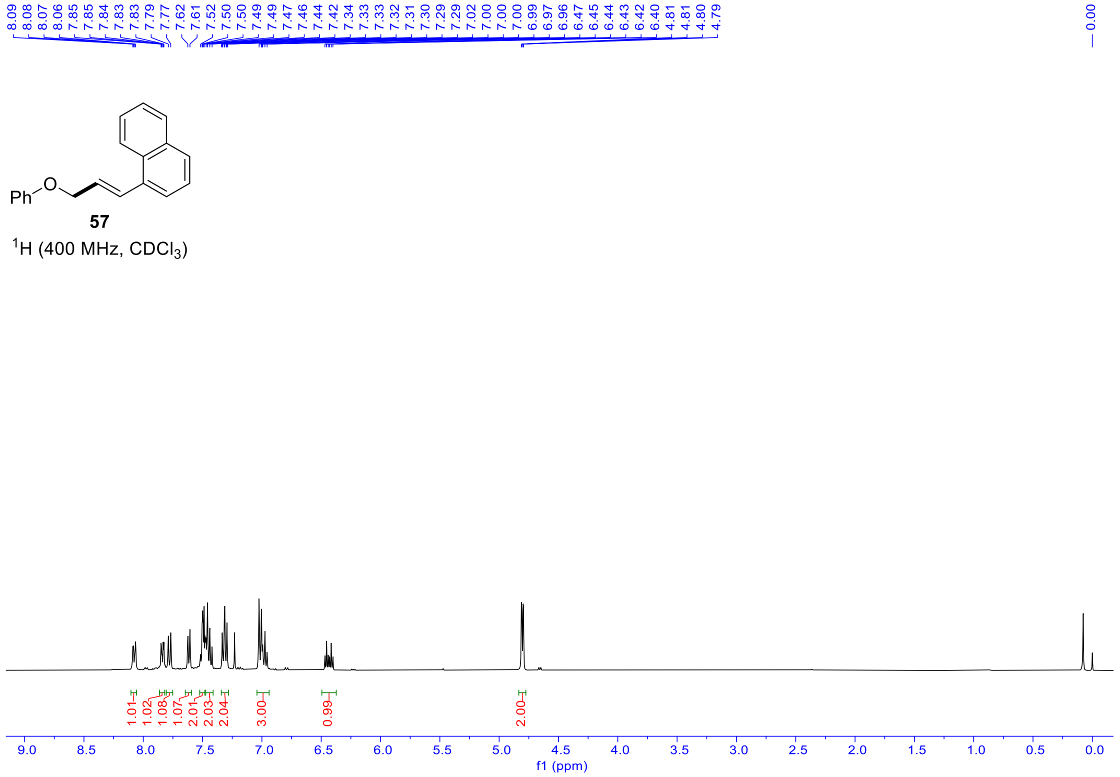

^{13}C NMR spectra of **57** (101 MHz, $CDCl_3$)

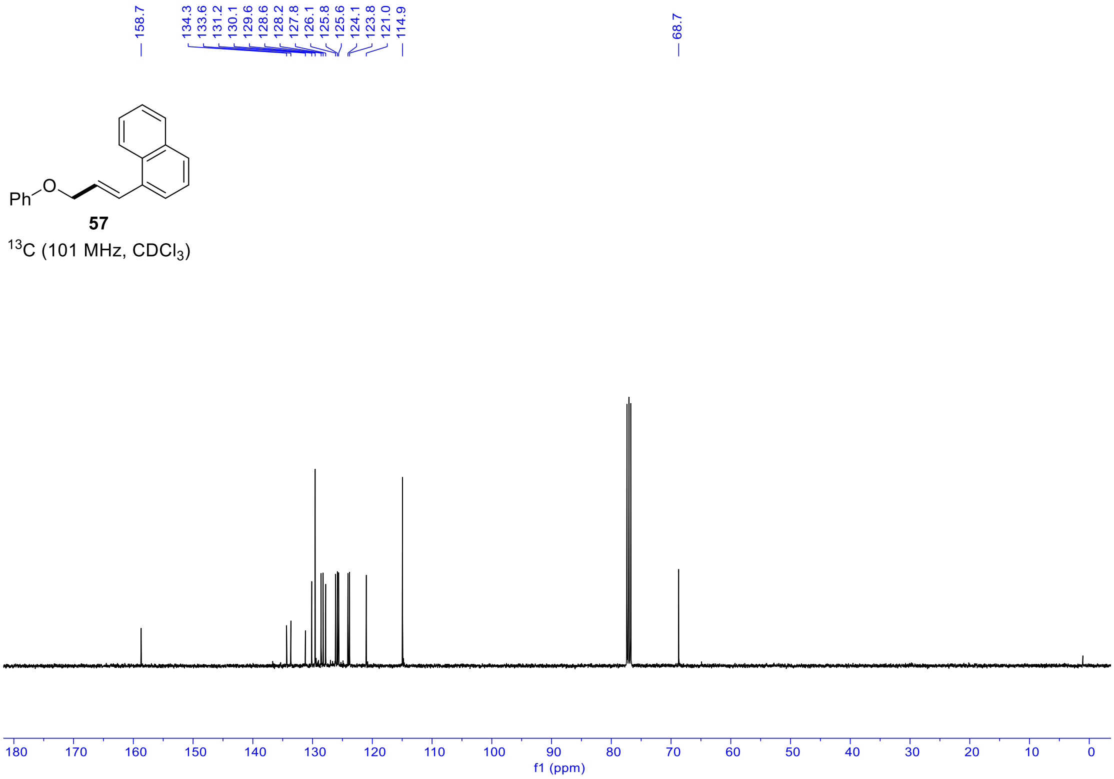

^{1}H NMR spectra of **58** (400 MHz, $CDCl_3$)

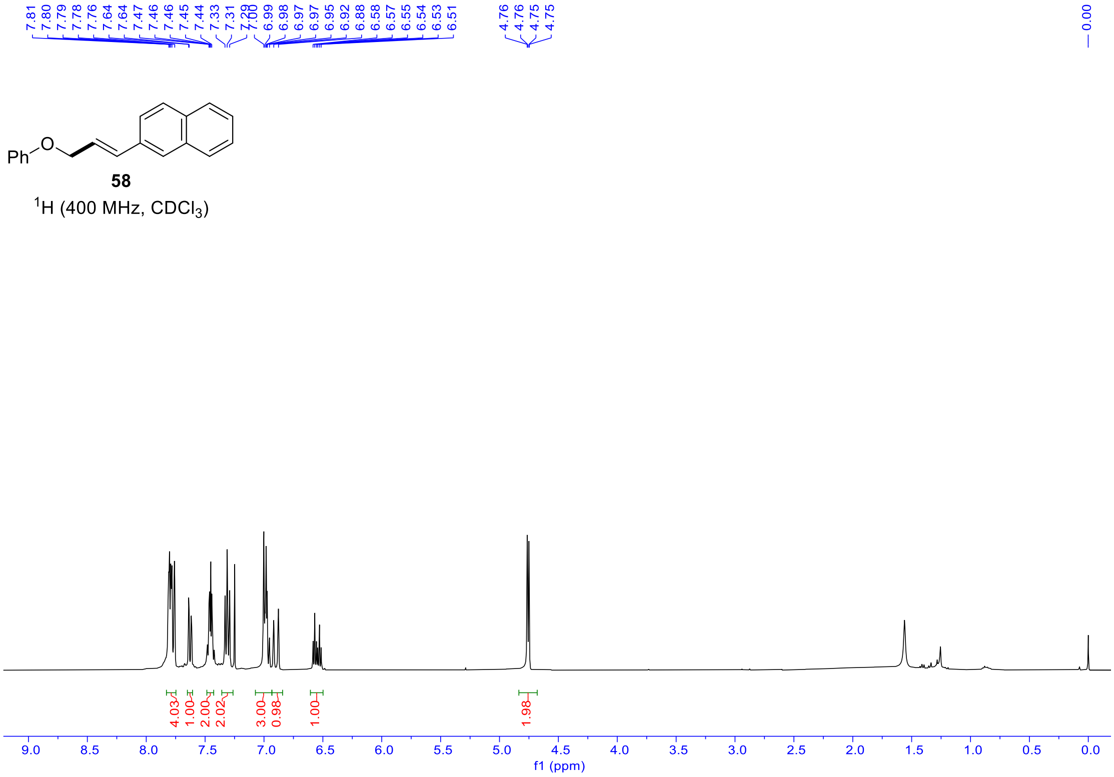

^{13}C NMR spectra of **58** (101 MHz, $CDCl_3$)

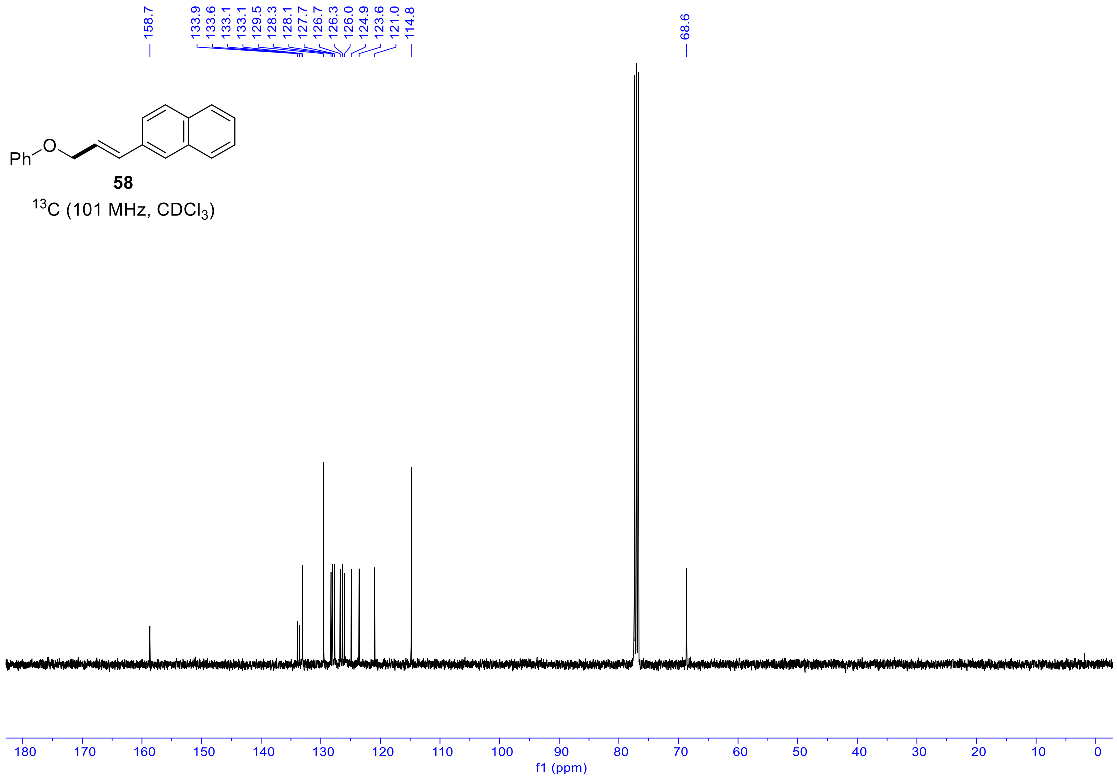

^{1}H NMR spectra of **59** (400 MHz, $CDCl_3$)

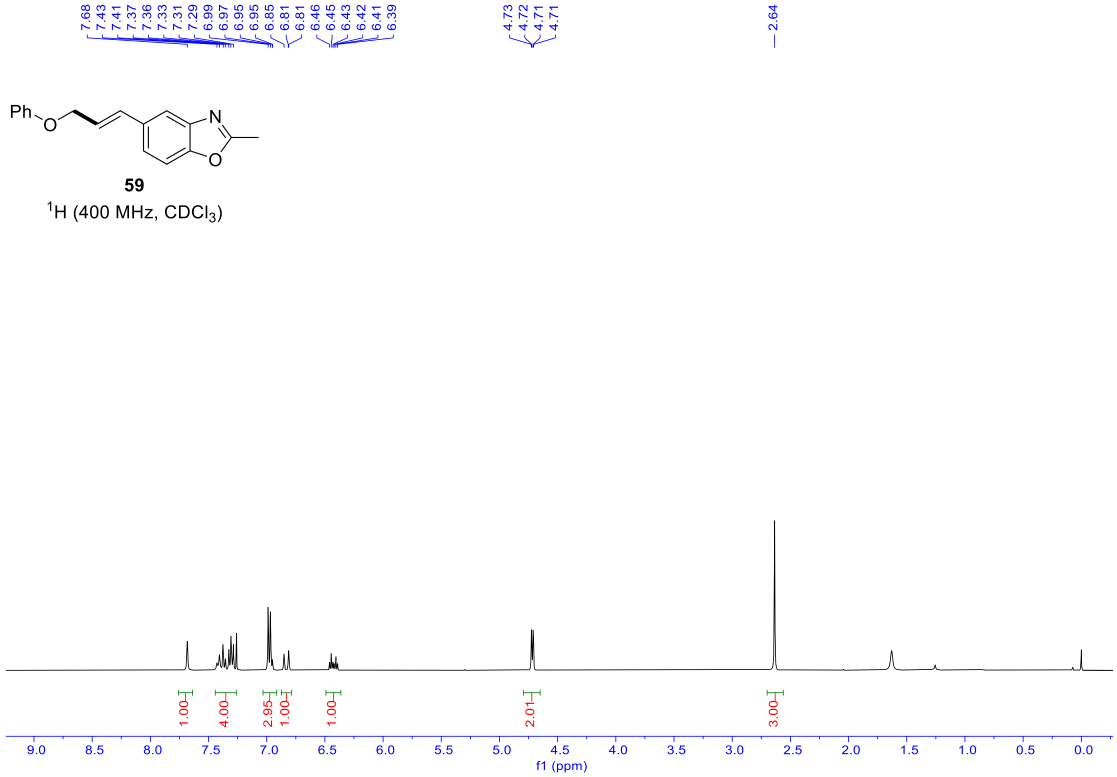

^{13}C NMR spectra of **59** (101 MHz, CDCl$_3$)

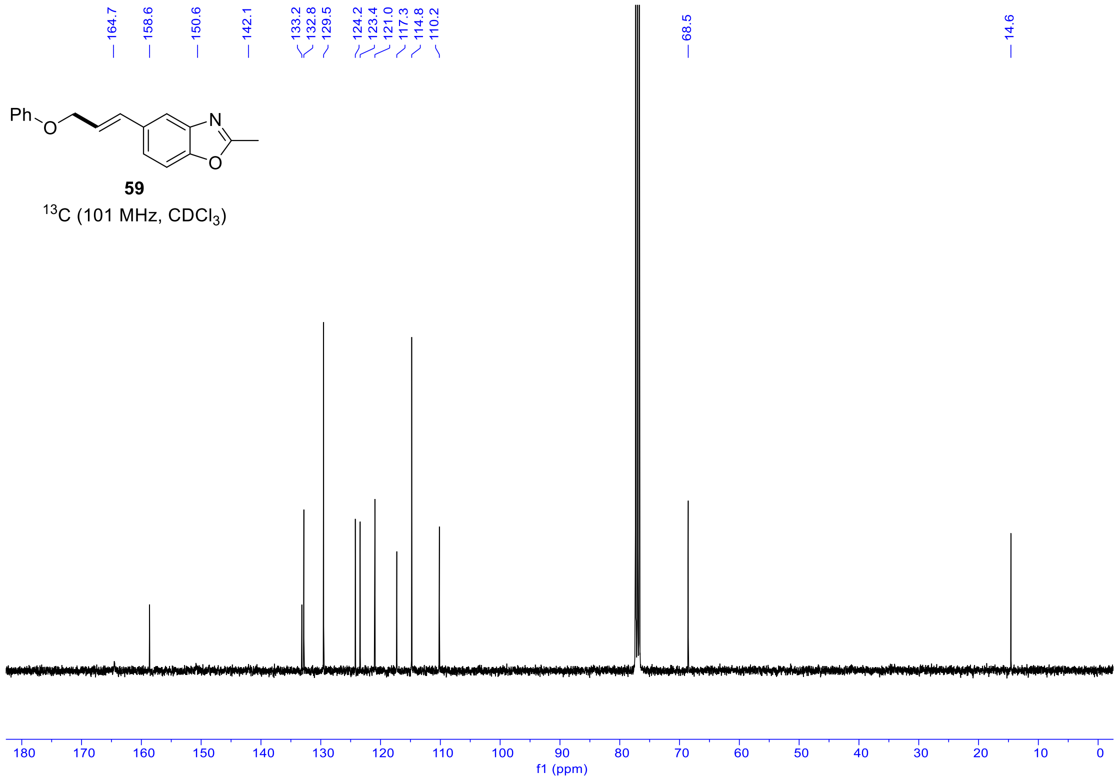

^{1}H NMR spectra of **60** (400 MHz, CDCl$_3$)

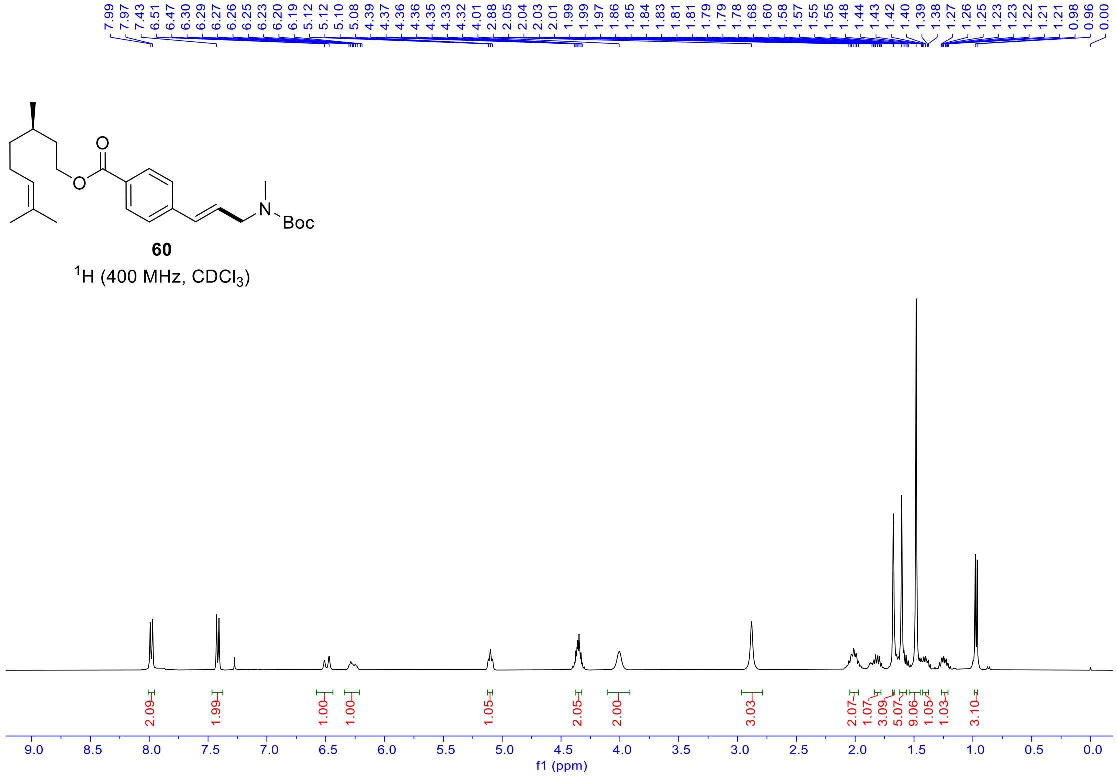

^{13}C NMR spectra of **60** (101 MHz, CDCl$_3$)

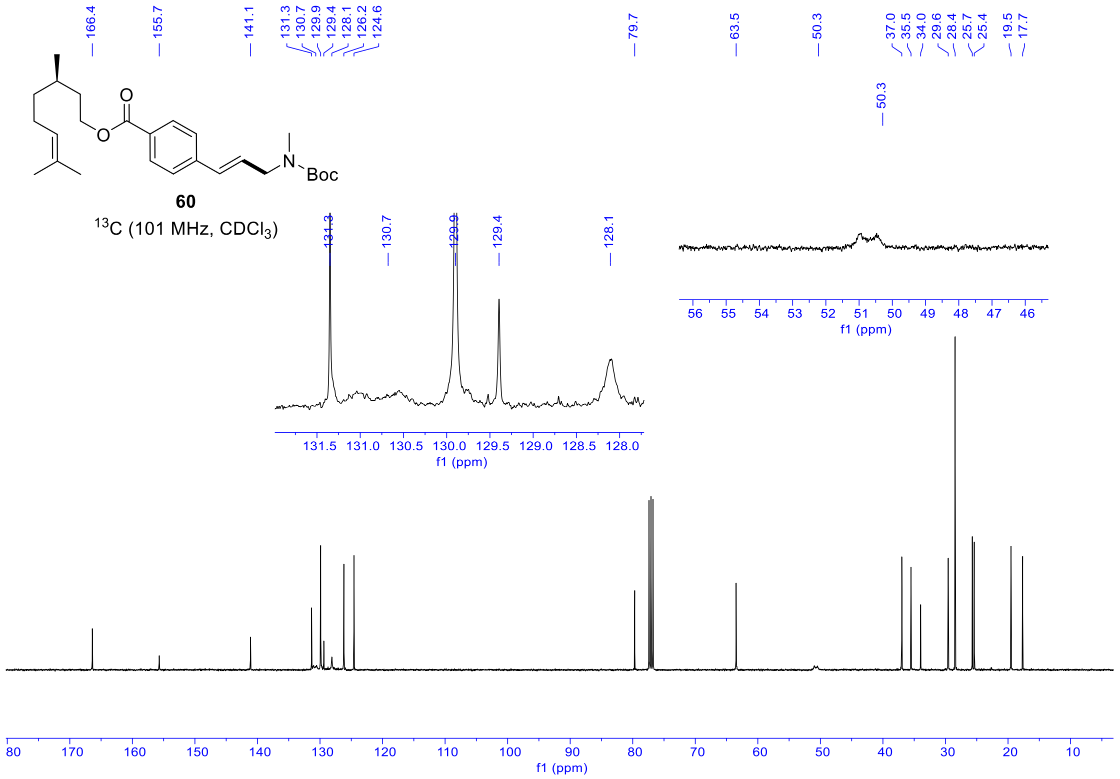

^{1}H NMR spectra of **61** (400 MHz, CDCl$_3$)

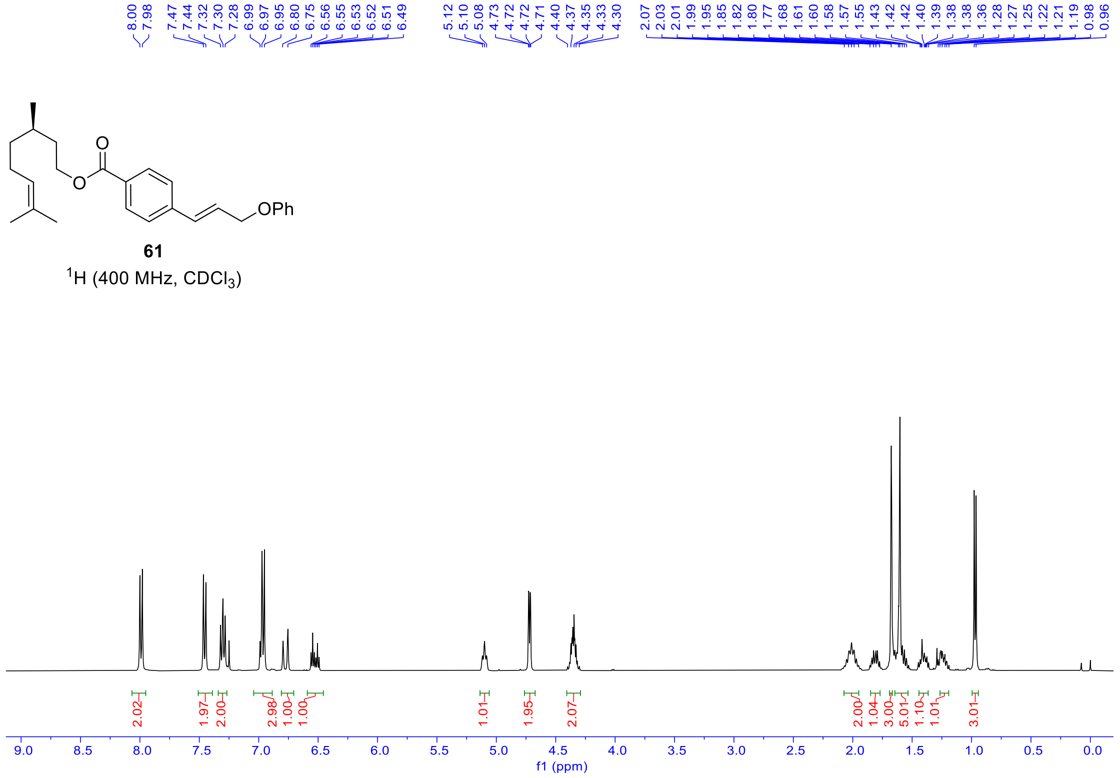

^{13}C NMR spectra of **61** (101 MHz, CDCl$_3$)

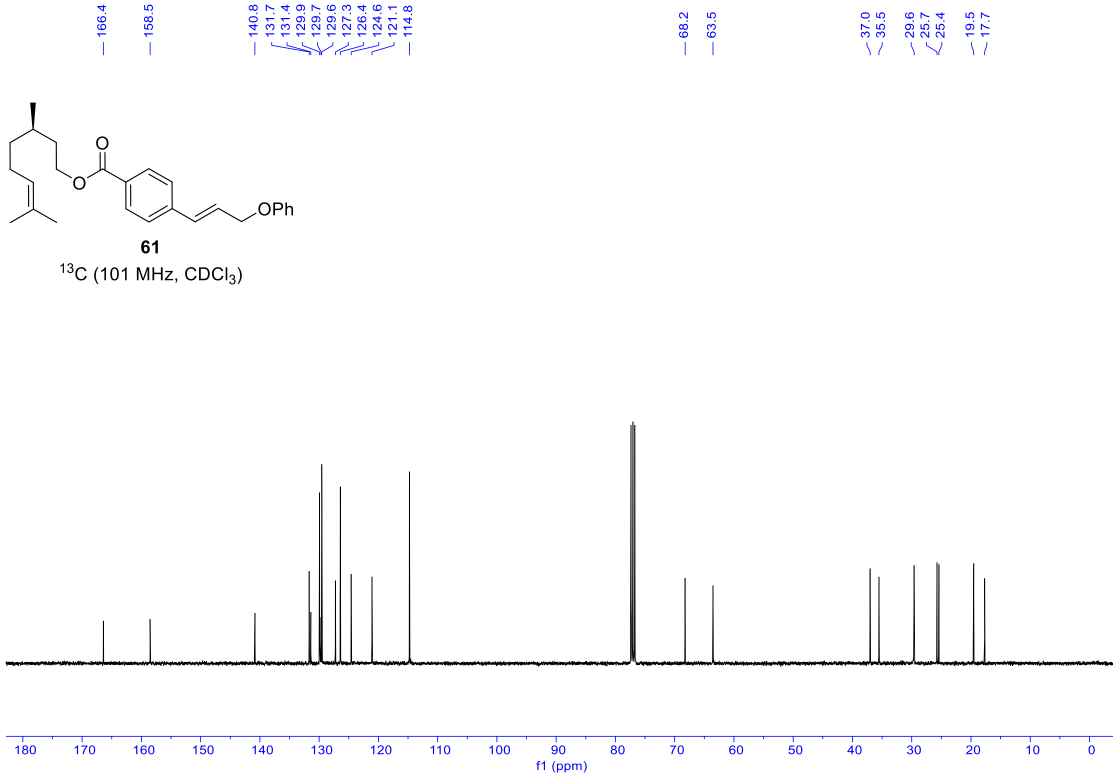

^{1}H NMR spectra of **62** (400 MHz, CDCl$_3$)

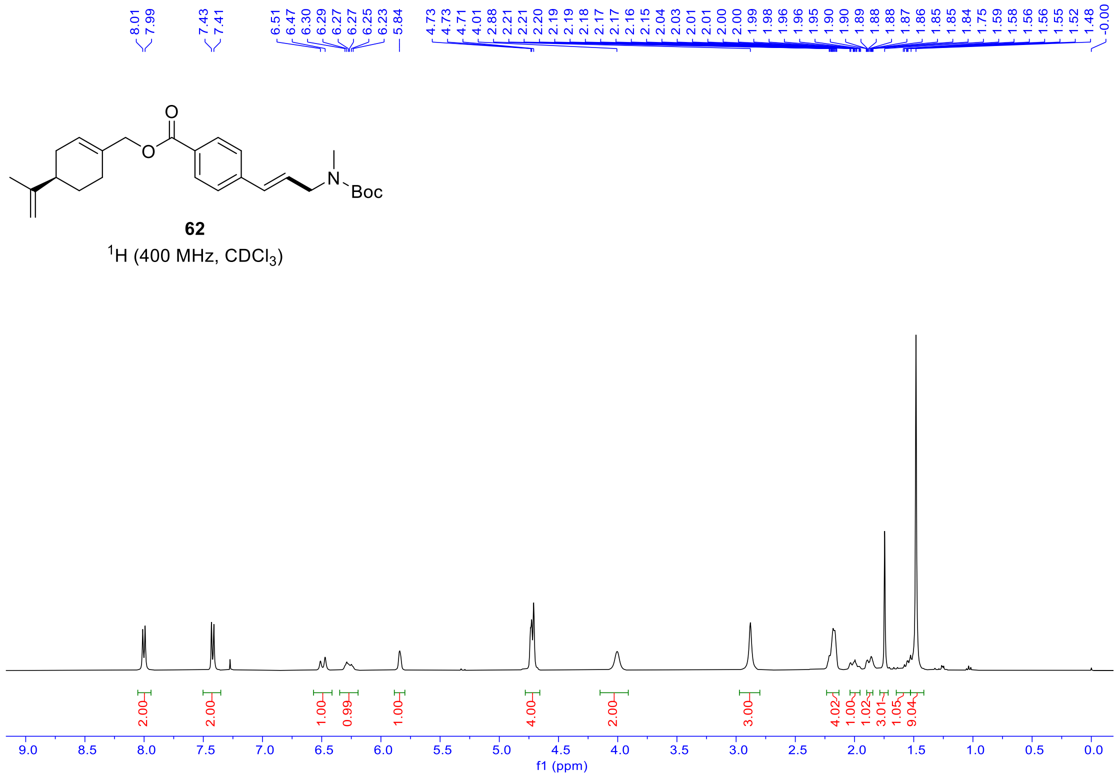

^{13}C NMR spectra of **62** (101 MHz, $CDCl_3$)

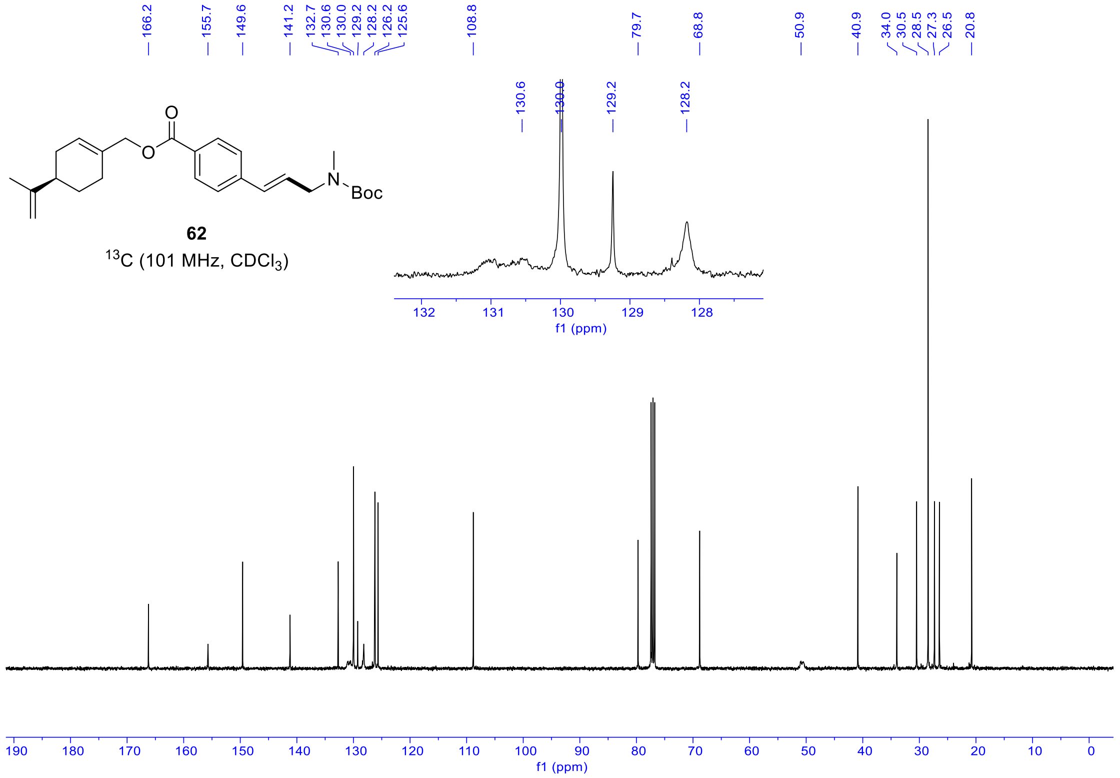

^{1}H NMR spectra of **63** (400 MHz, $CDCl_3$)

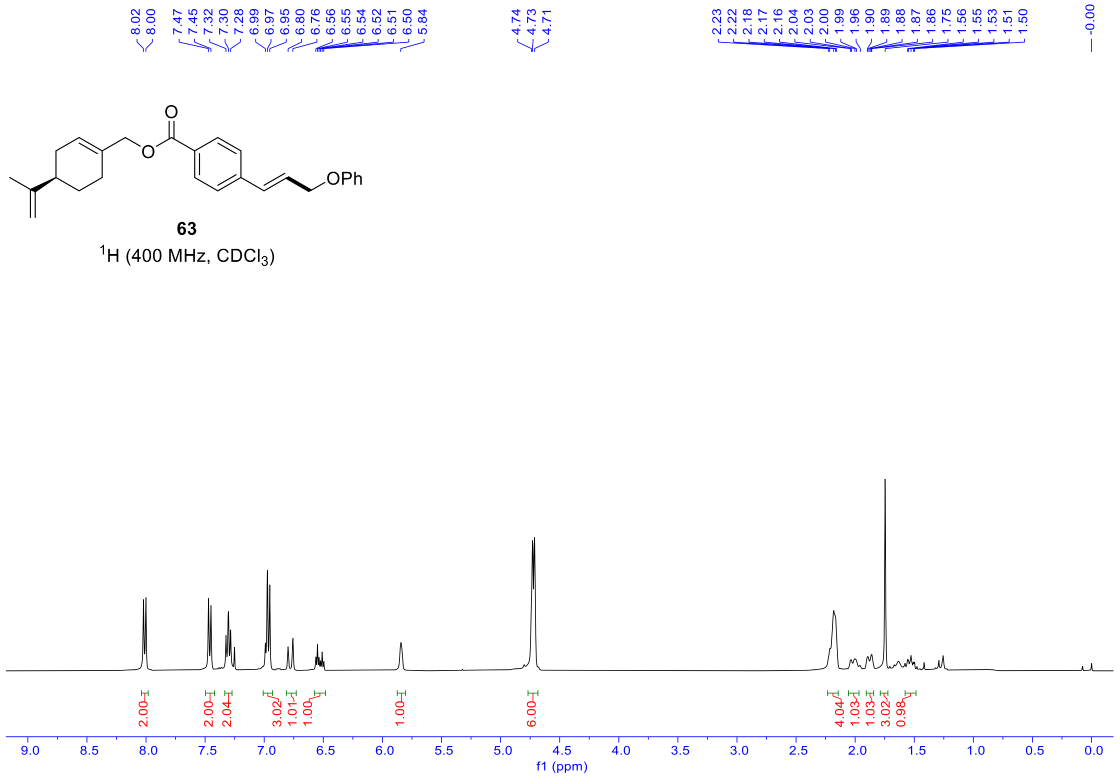

^{13}C NMR spectra of **63** (101 MHz, $CDCl_3$)

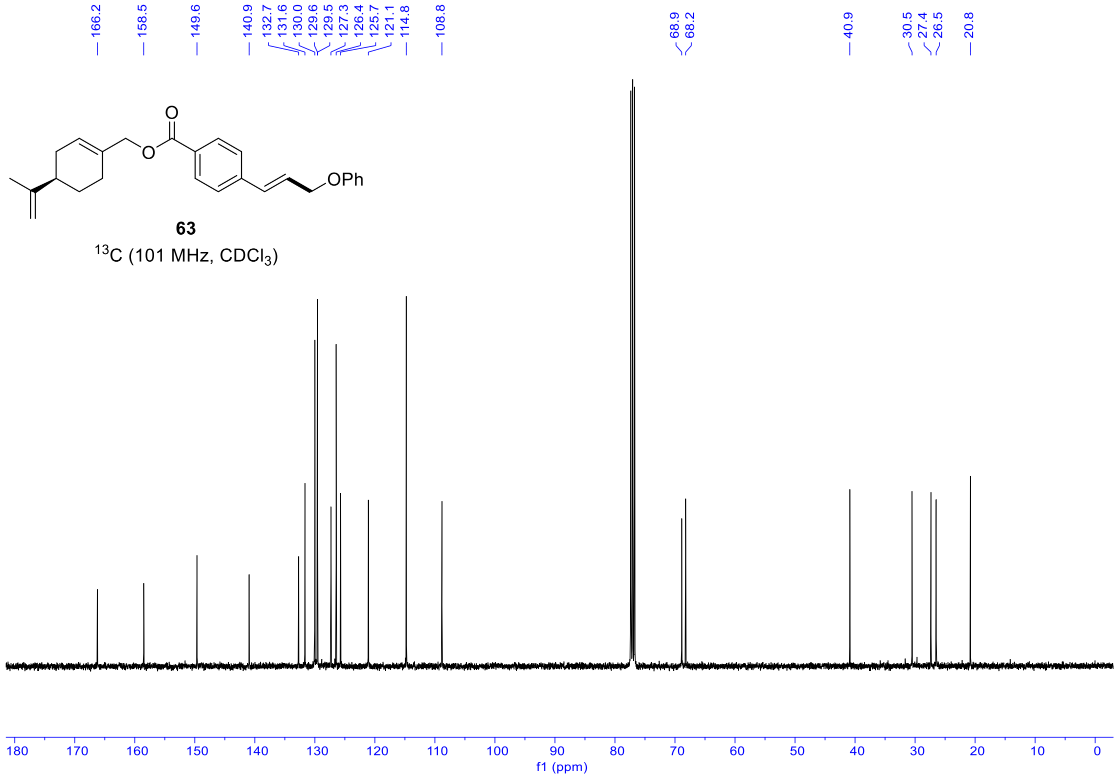

^{1}H NMR spectra of **64** (400 MHz, $CDCl_3$)

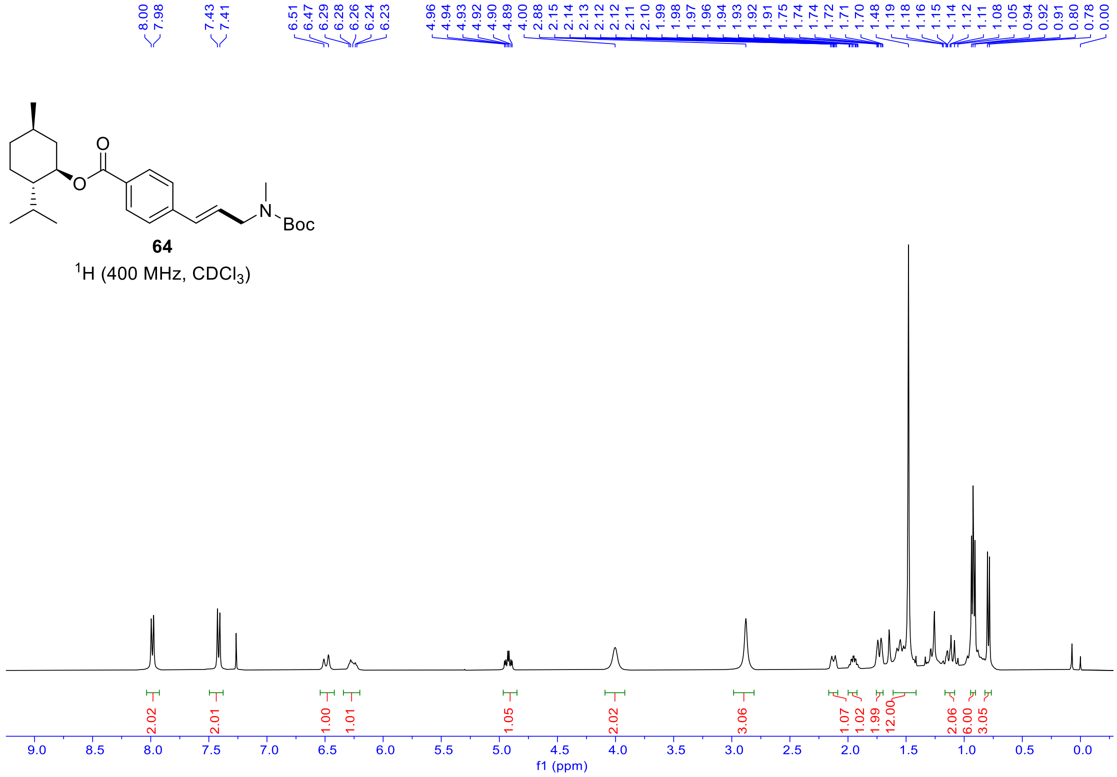

^{13}C NMR spectra of **64** (101 MHz, $CDCl_3$)

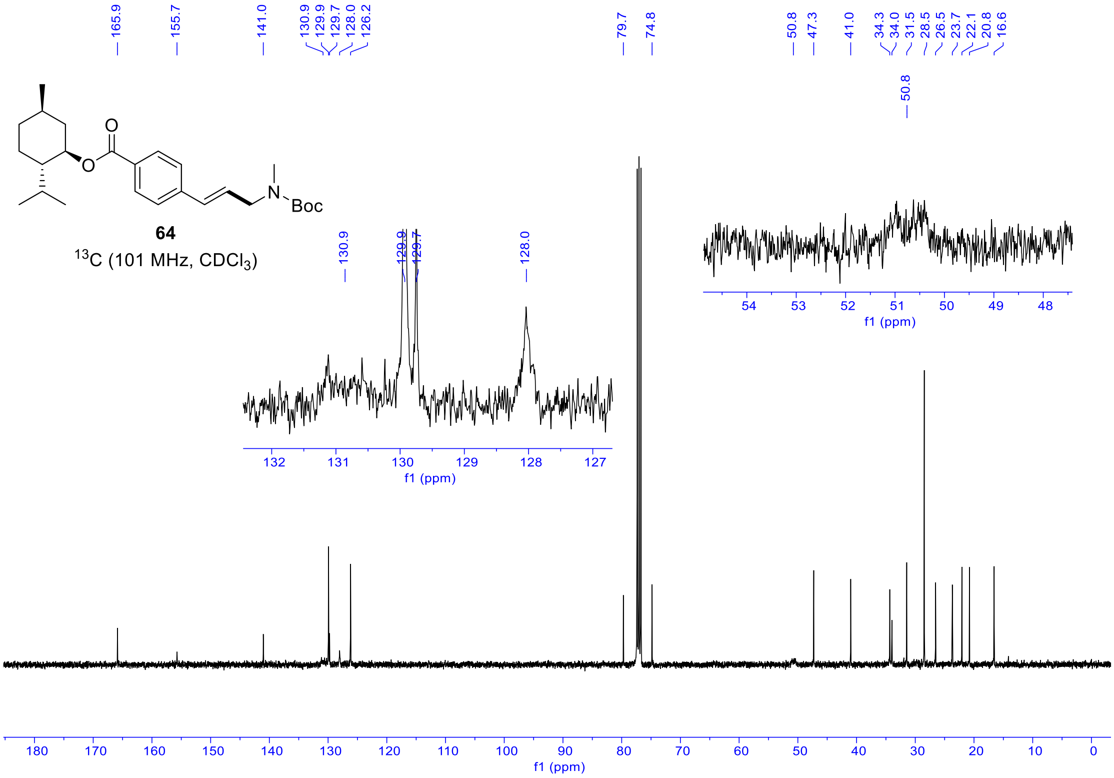

^{1}H NMR spectra of **65** (400 MHz, $CDCl_3$)

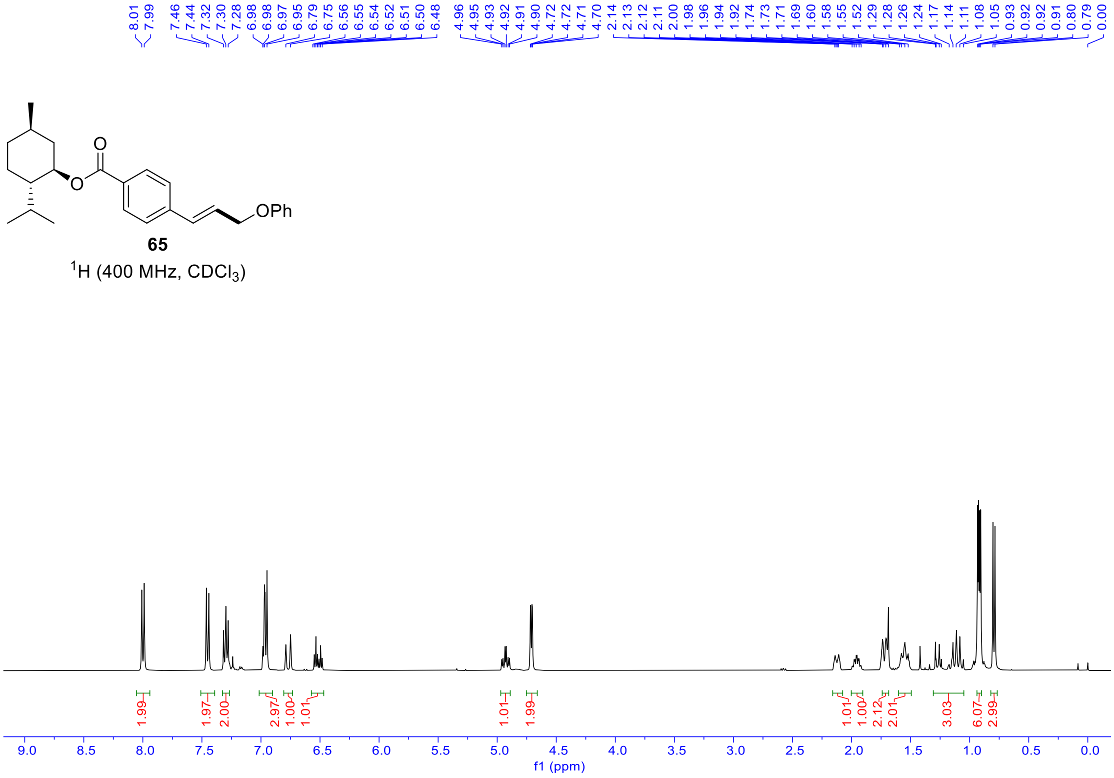

^{13}C NMR spectra of **65** (101 MHz, $CDCl_3$)

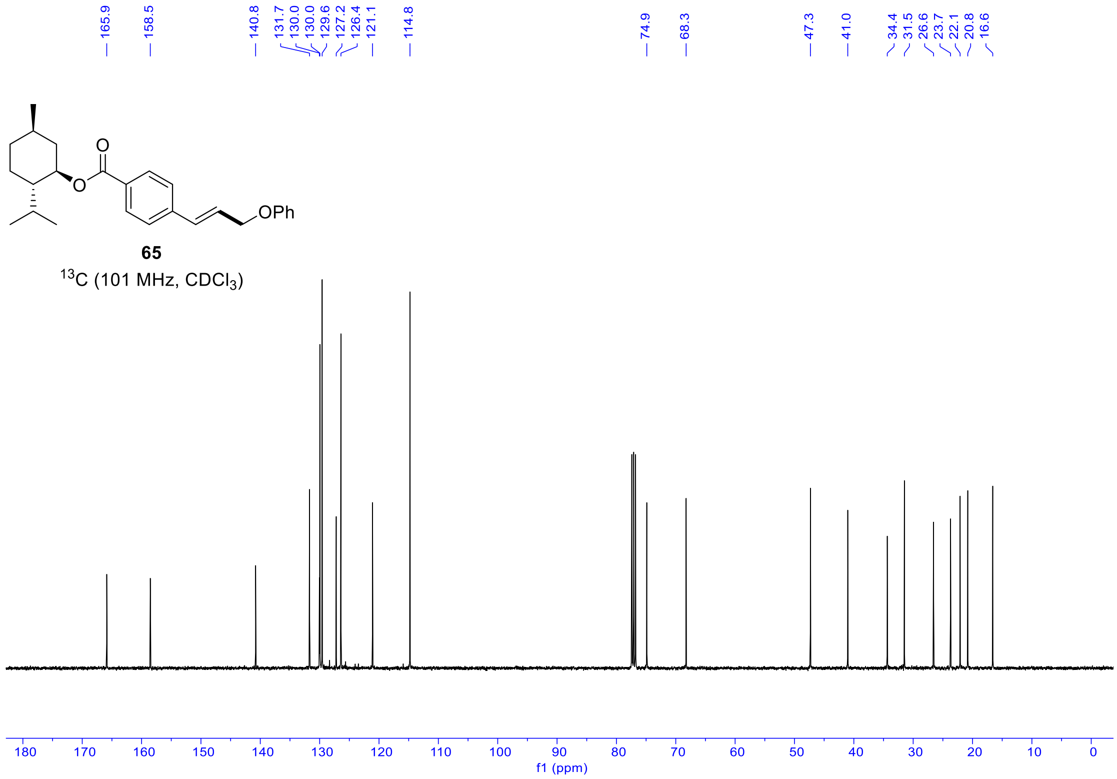

^{1}H NMR spectra of **66** (400 MHz, $CDCl_3$)

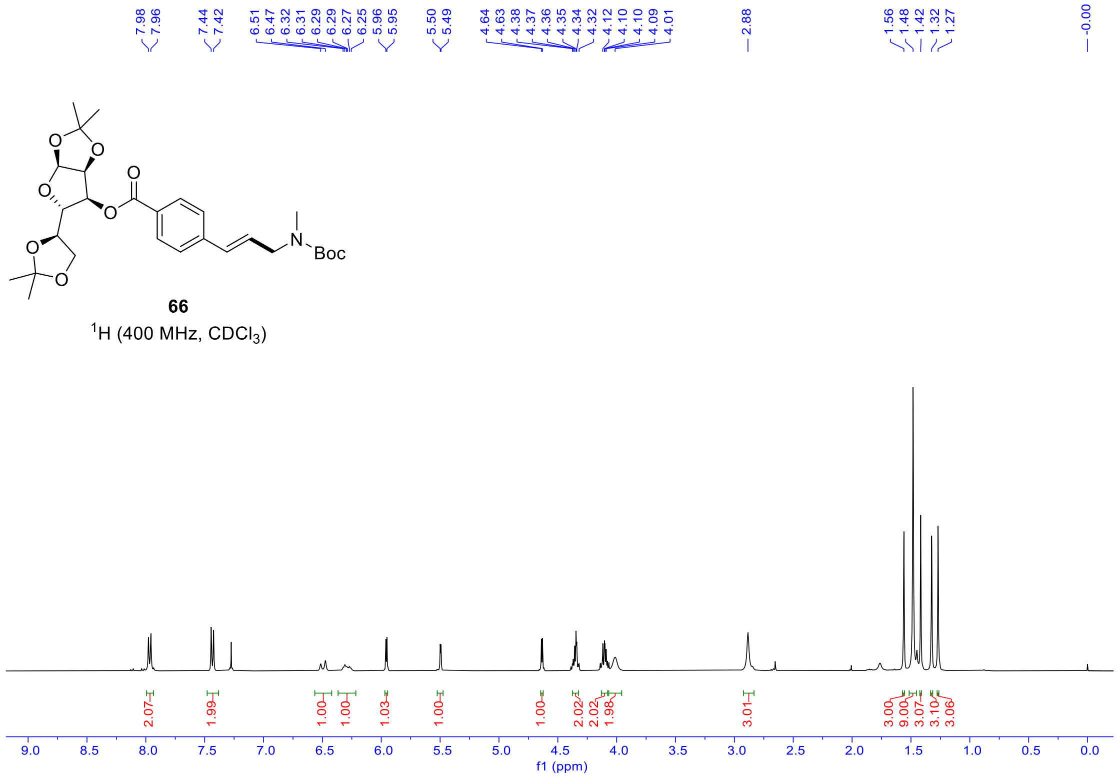

^{13}C NMR spectra of **66** (101 MHz, $CDCl_3$)

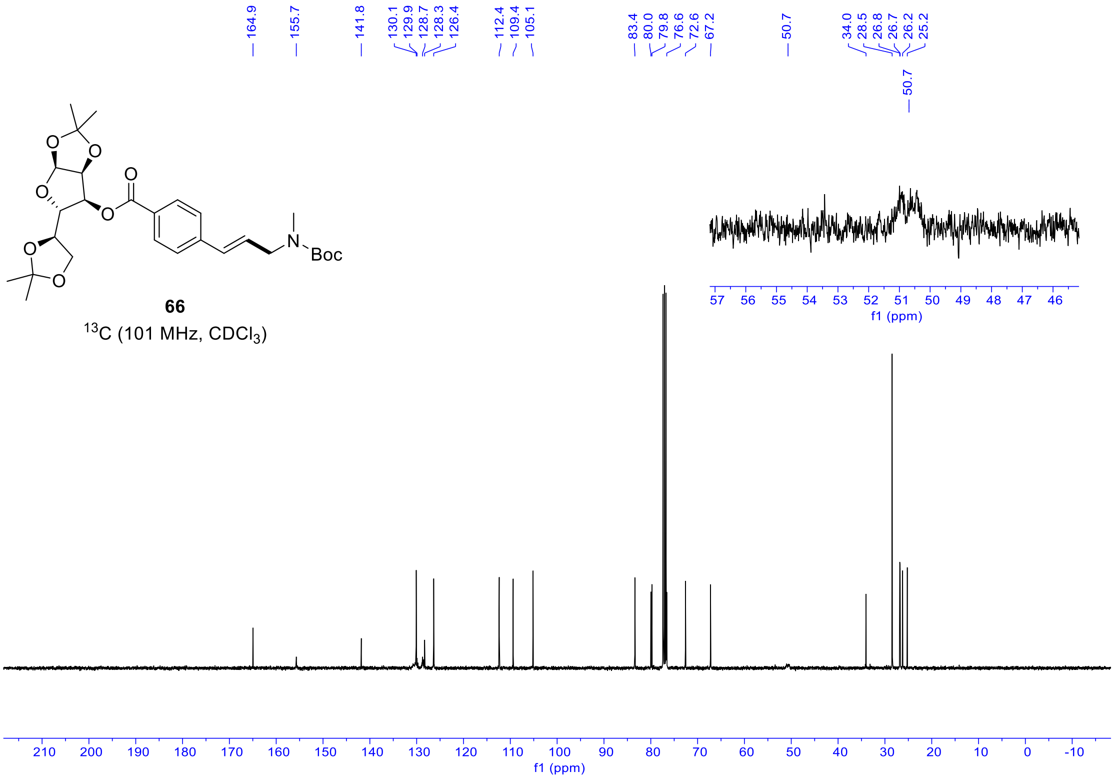

^{1}H NMR spectra of **67** (400 MHz, $CDCl_3$)

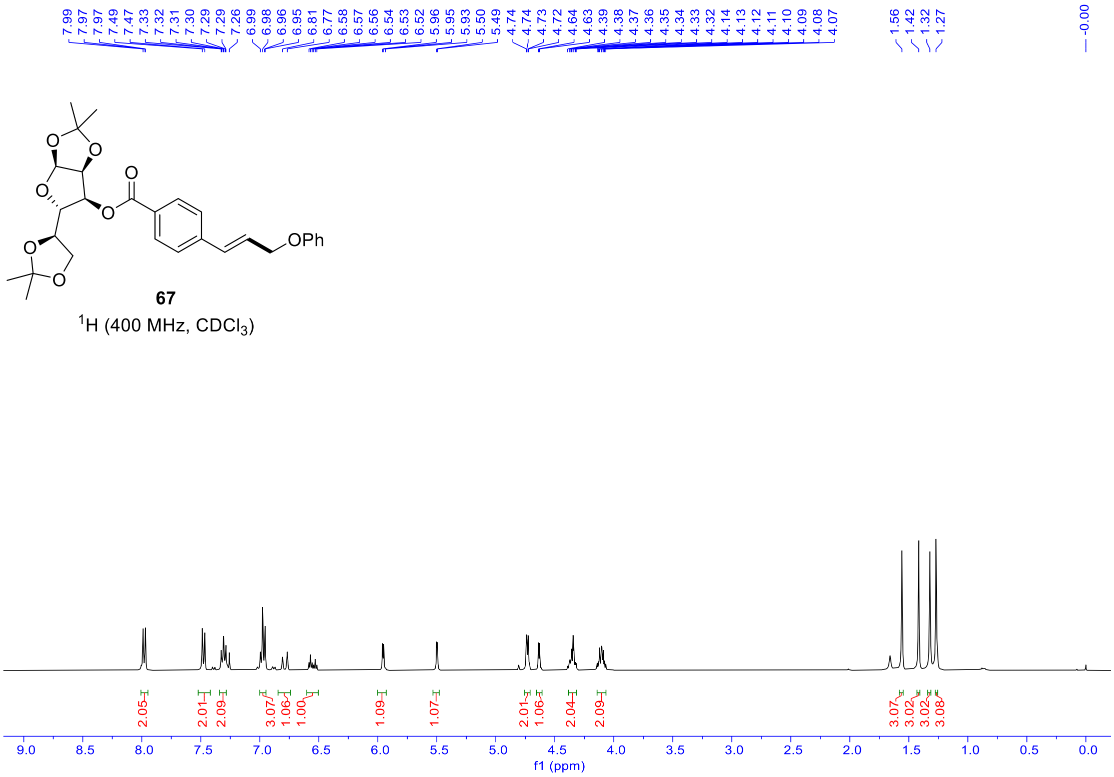

^{13}C NMR spectra of **67** (101 MHz, $CDCl_3$)

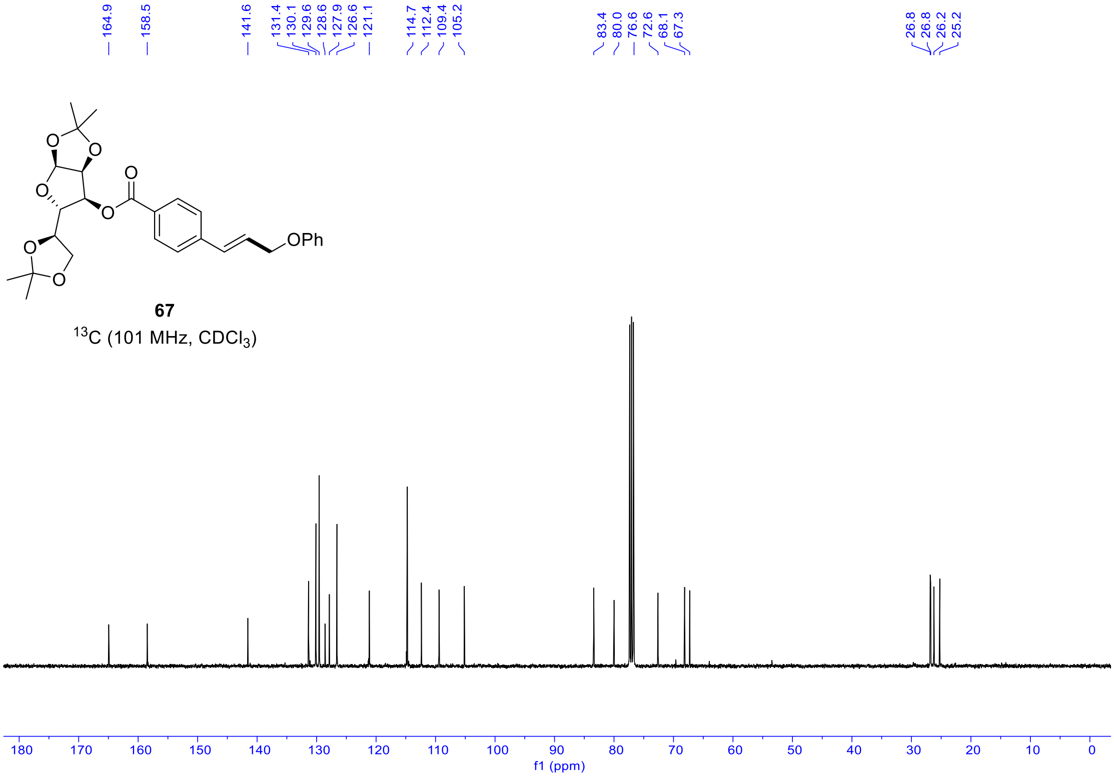

^{1}H NMR spectra of **68** (400 MHz, $CDCl_3$)

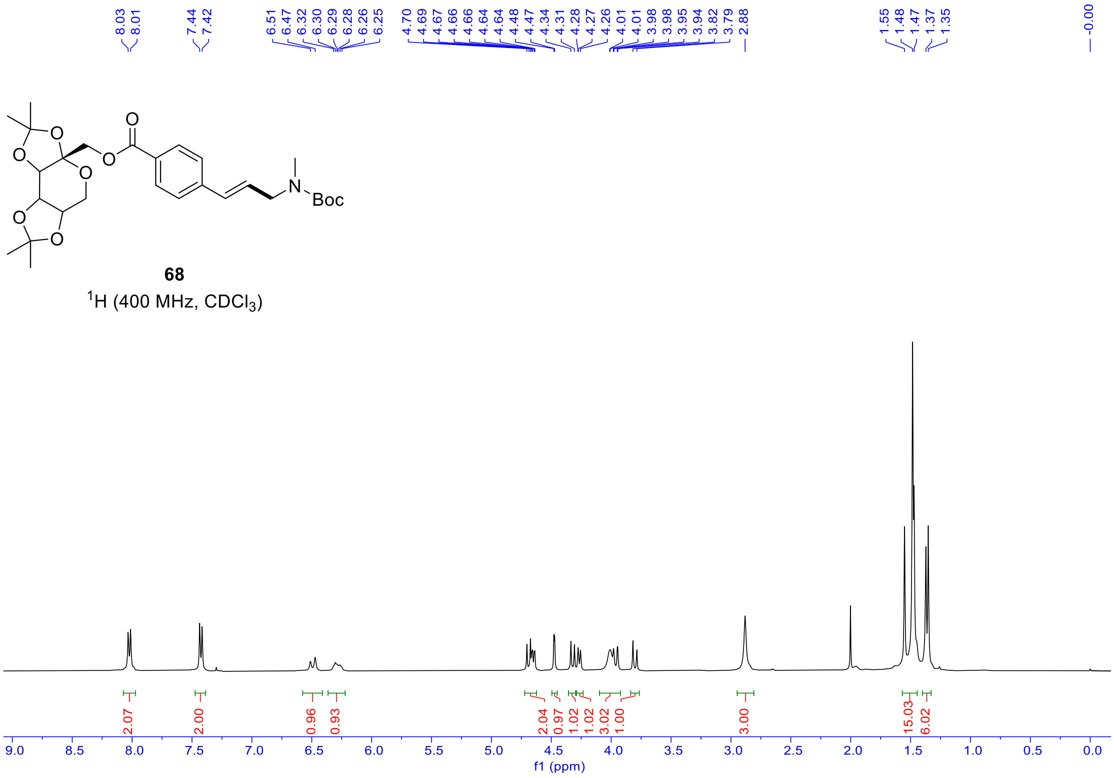

^{13}C NMR spectra of **68** (101 MHz, $CDCl_3$)

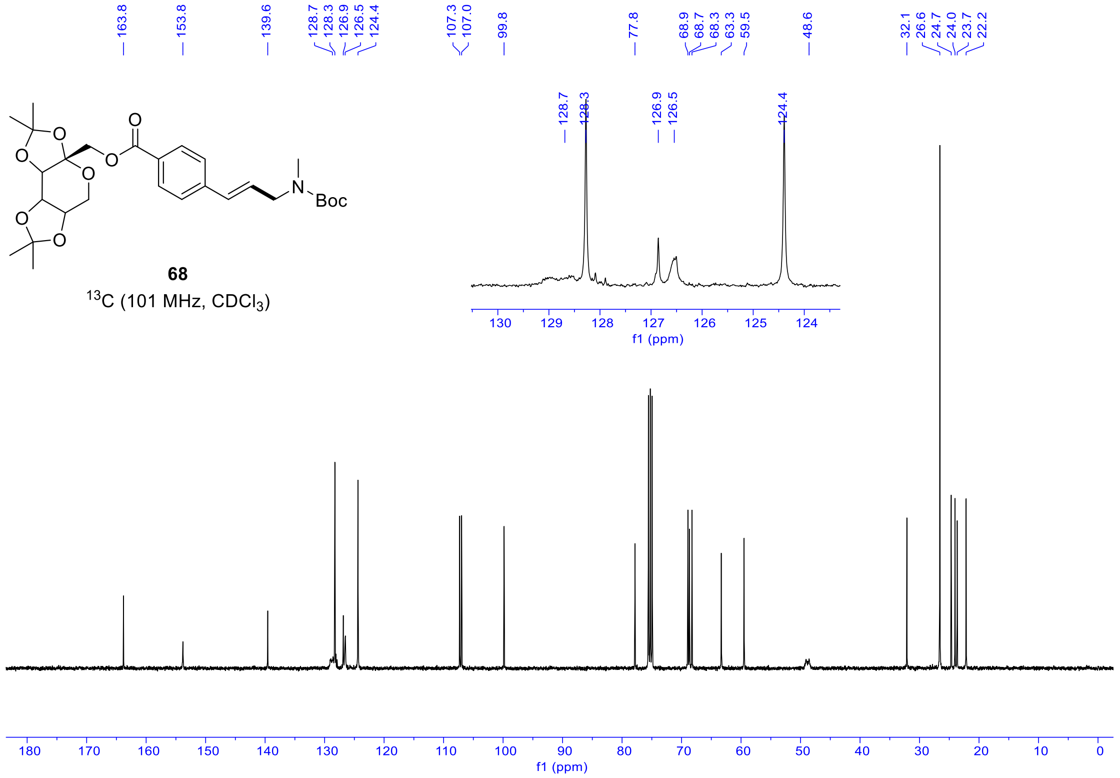

^{1}H NMR spectra of **69** (400 MHz, $CDCl_3$)

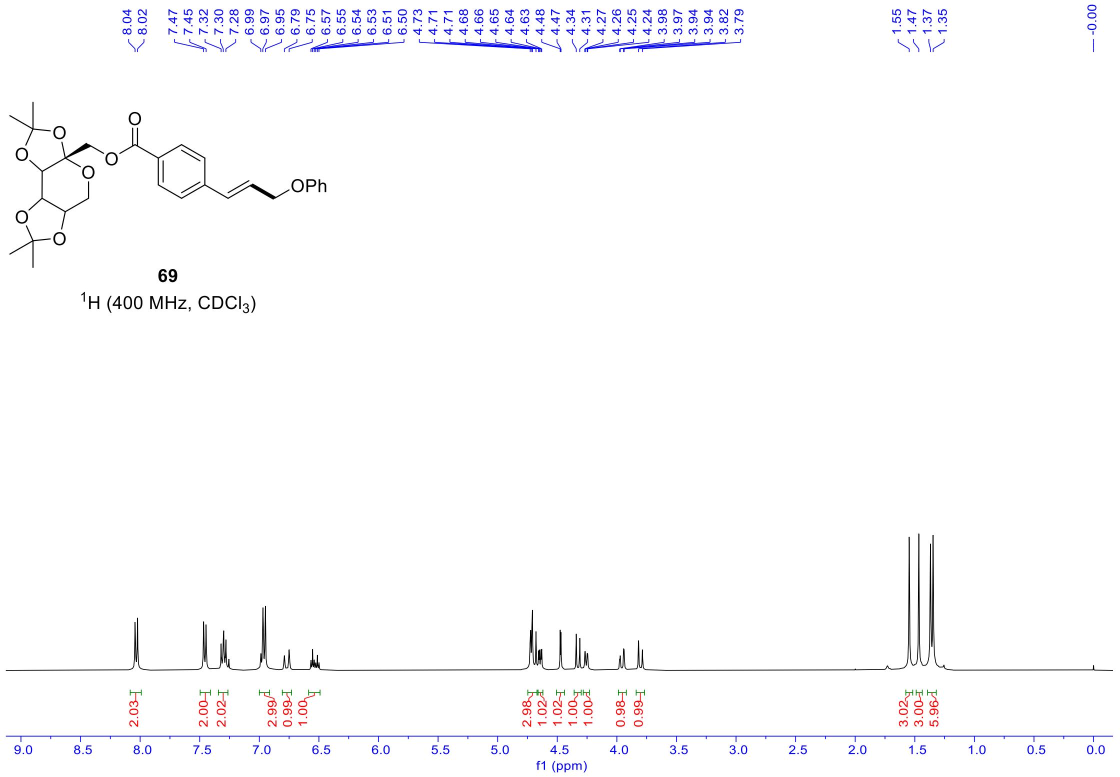

^{13}C NMR spectra of **69** (101 MHz, $CDCl_3$)

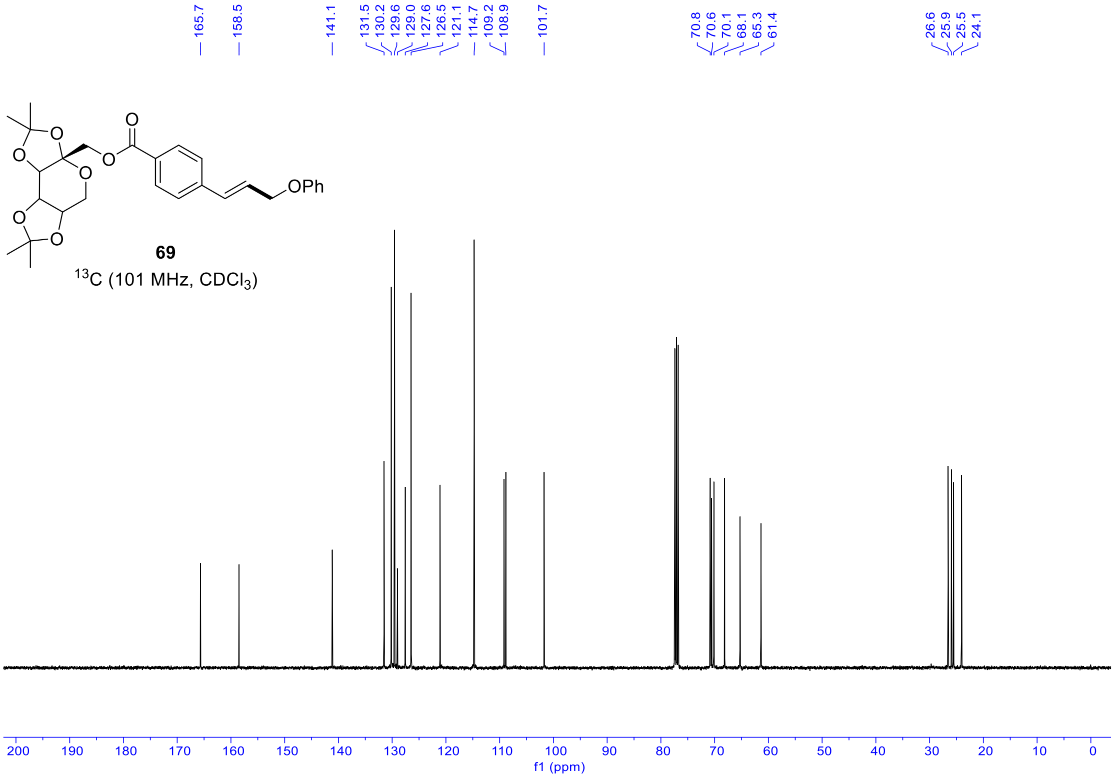

^{1}H NMR spectra of **70** (400 MHz, $CDCl_3$)

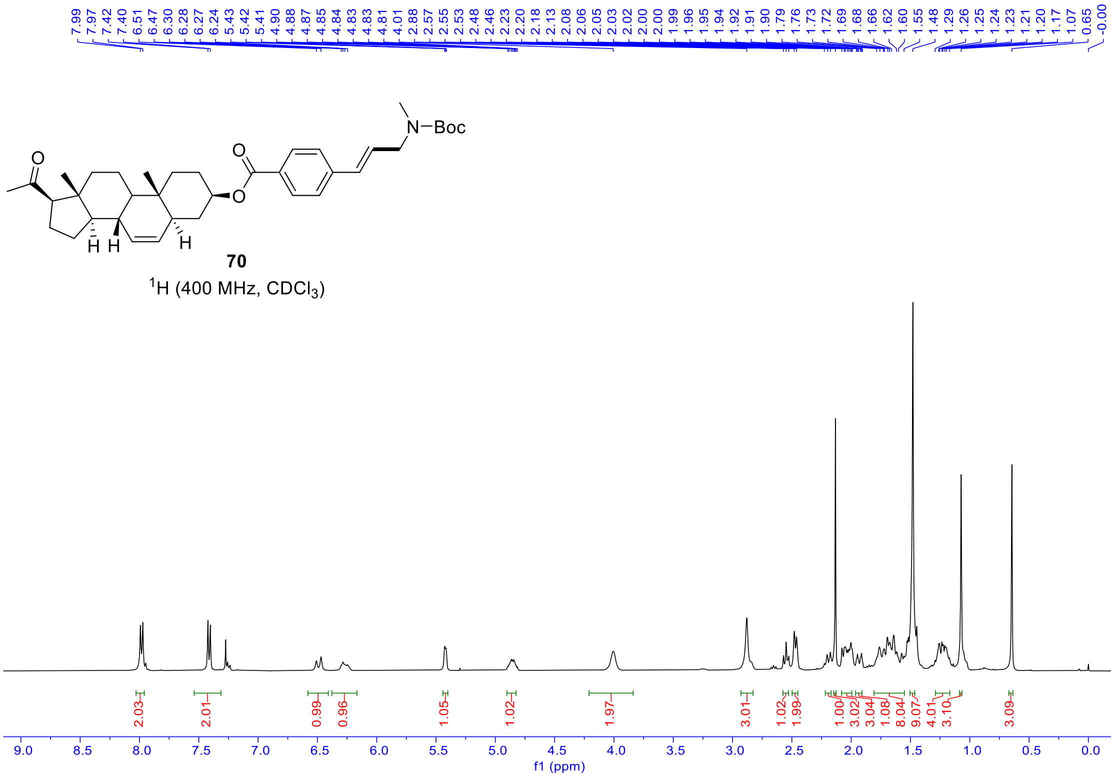

^{13}C NMR spectra of **70** (101 MHz, CDCl$_3$)

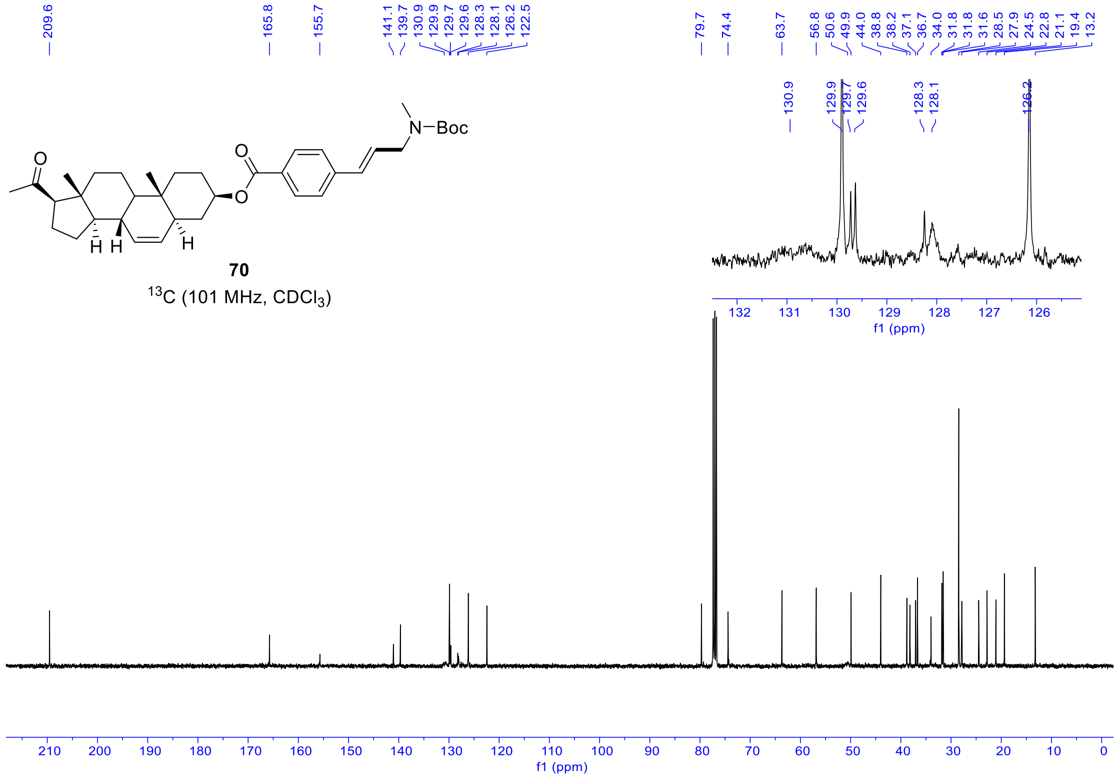

^{1}H NMR spectra of **71** (400 MHz, CDCl$_3$)

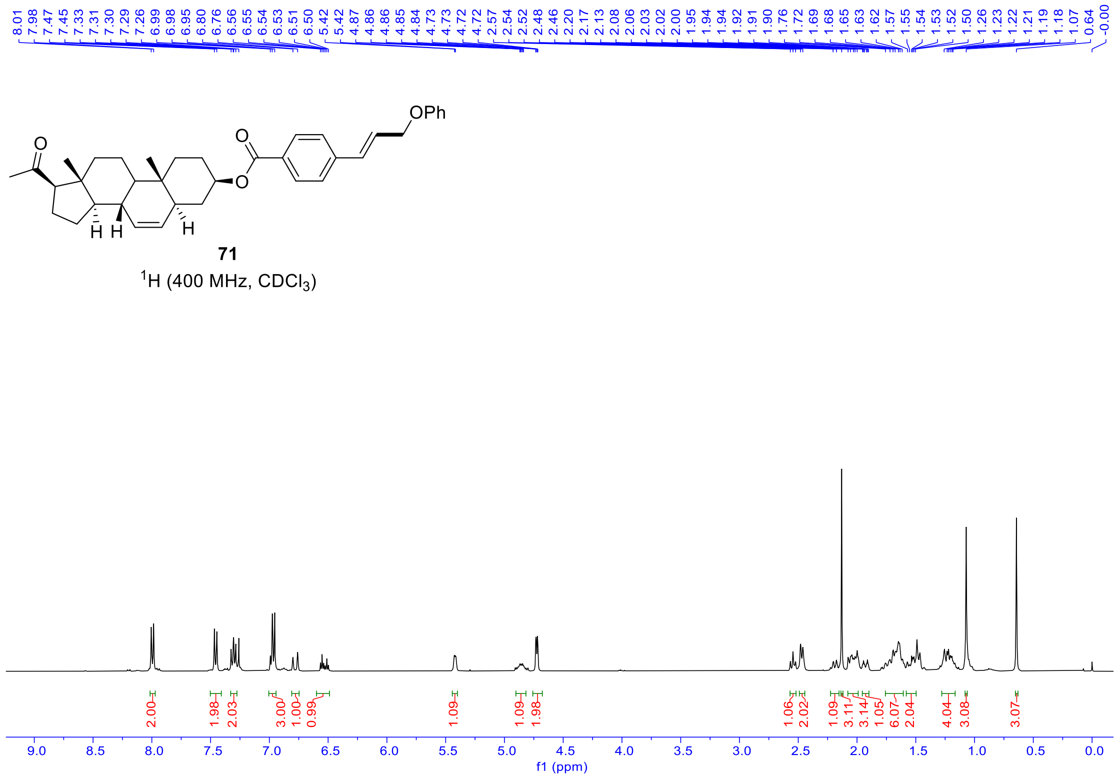

^{13}C NMR spectra of **71** (101 MHz, CDCl$_3$)

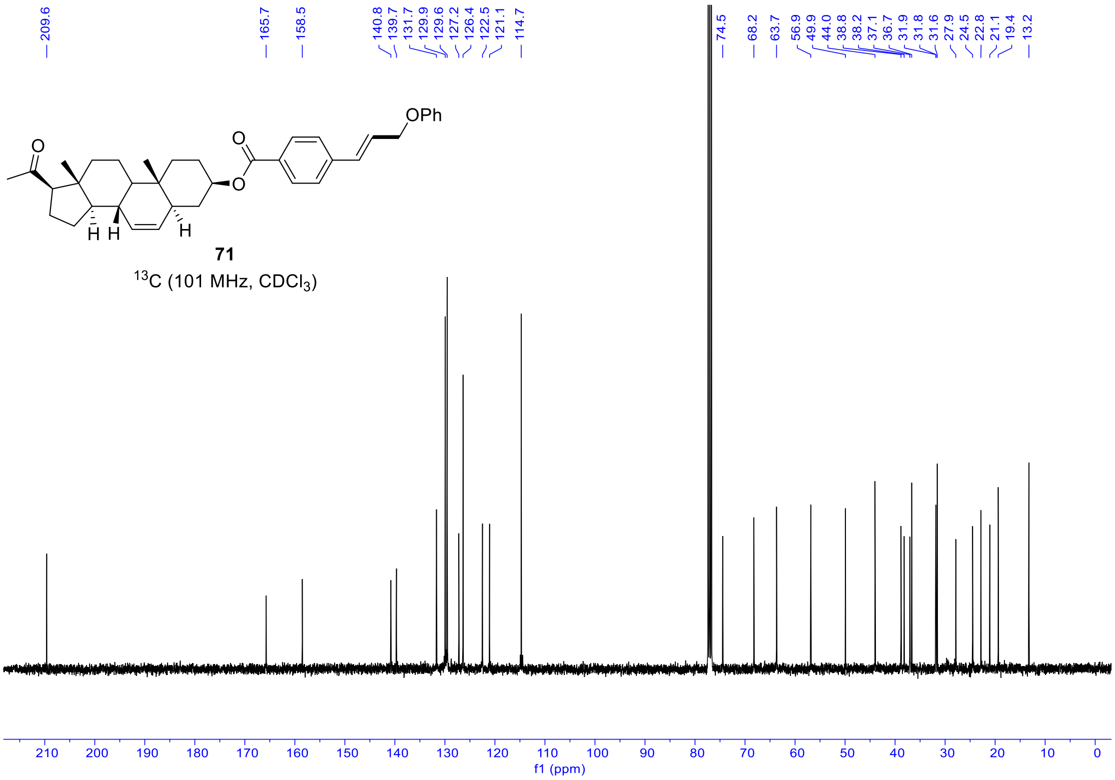

^{1}H NMR spectra of **72** (400 MHz, CDCl$_3$)

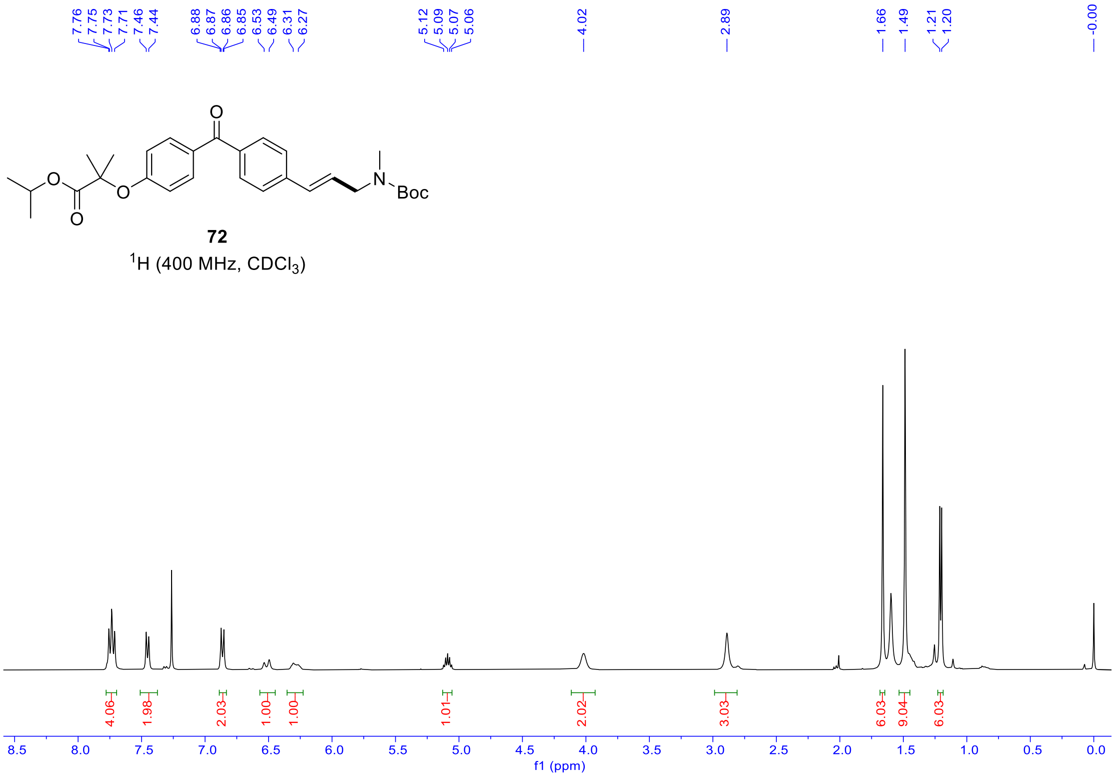

^{13}C NMR spectra of **72** (101 MHz, CDCl$_3$)

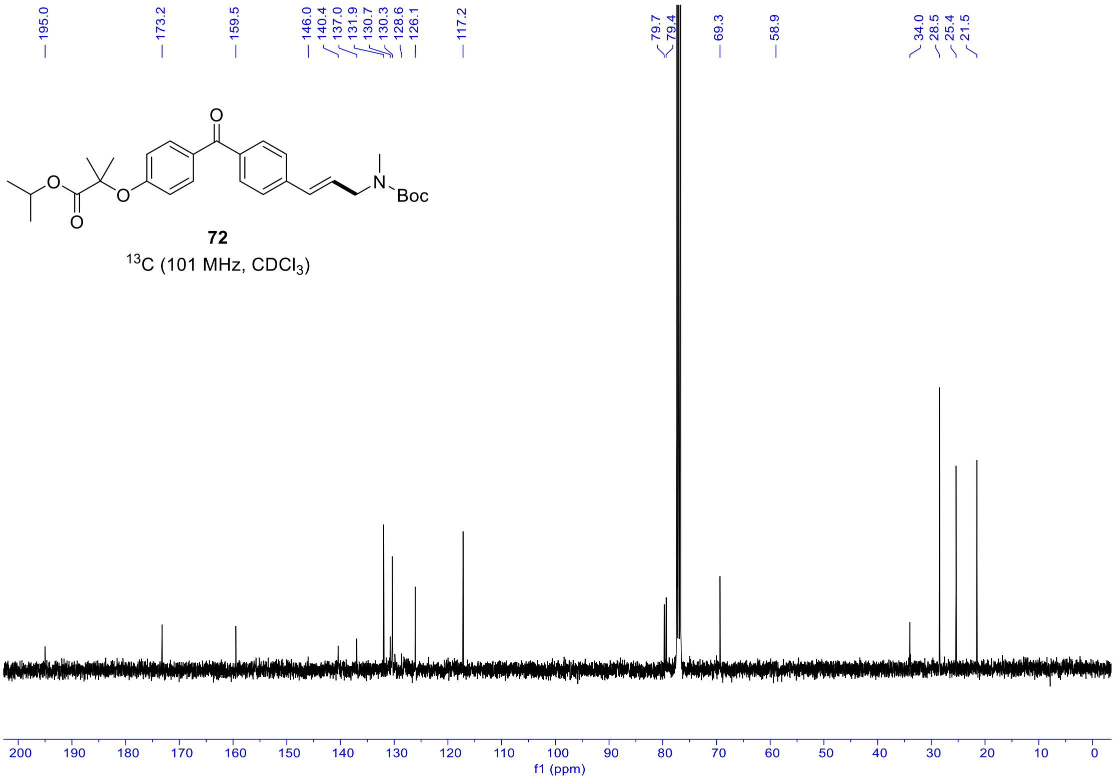

^{1}H NMR spectra of **73** (400 MHz, CDCl$_3$)

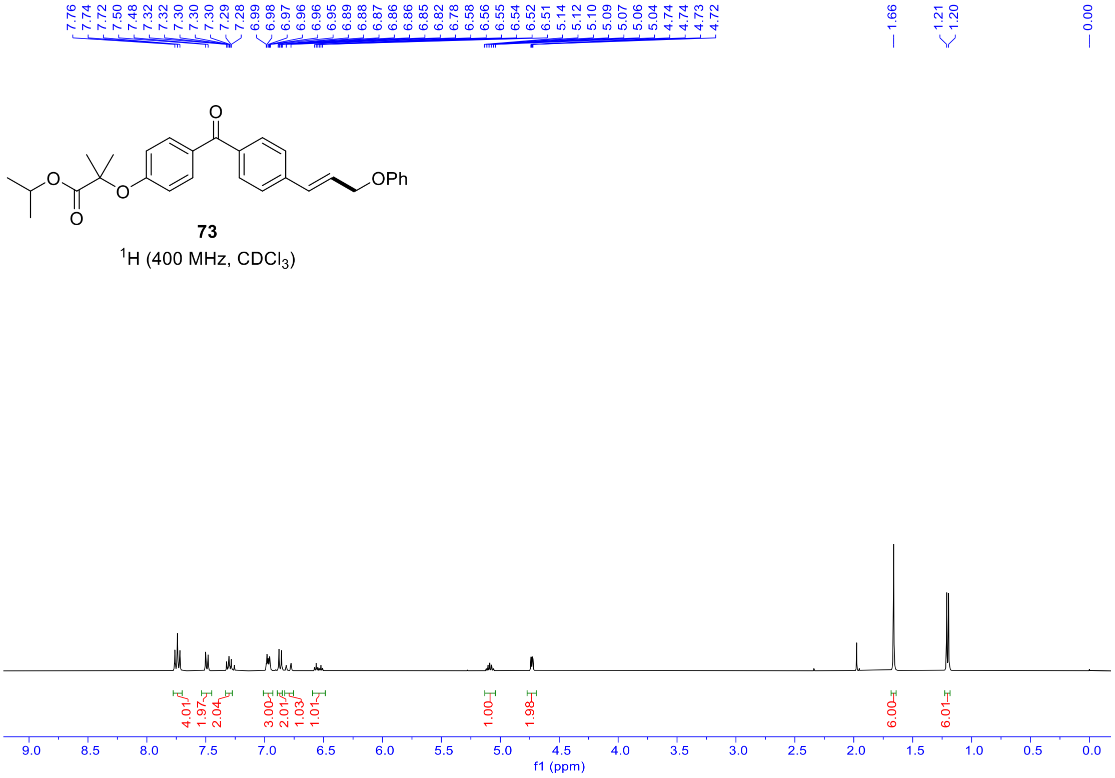

^{13}C NMR spectra of **73** (101 MHz, $CDCl_3$)

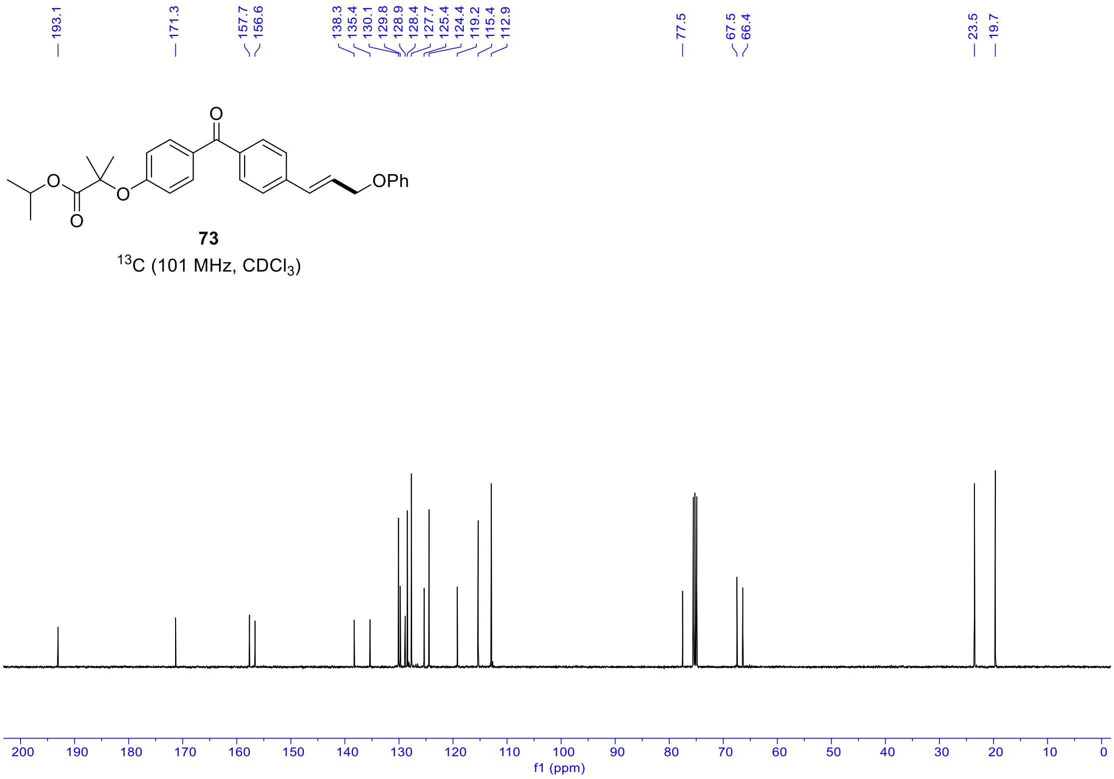

^{1}H NMR spectra of **74** (400 MHz, $CDCl_3$)

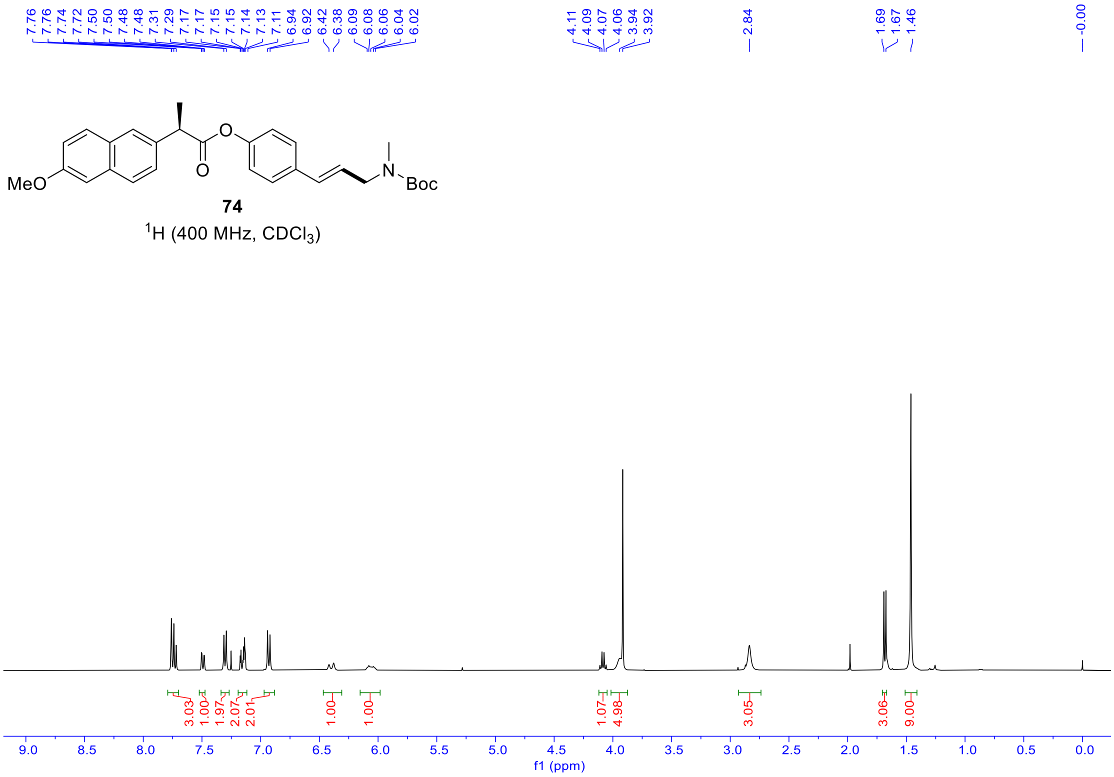

^{13}C NMR spectra of **74** (101 MHz, $CDCl_3$)

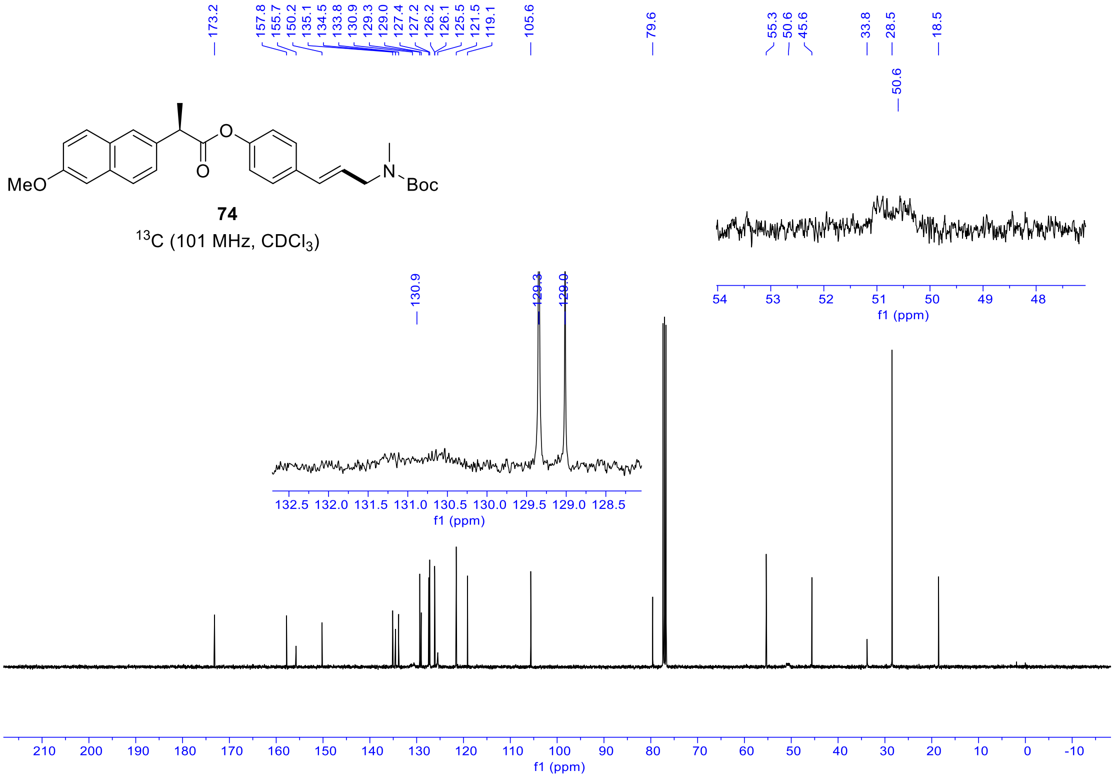

^{1}H NMR spectra of **75** (400 MHz, $CDCl_3$)

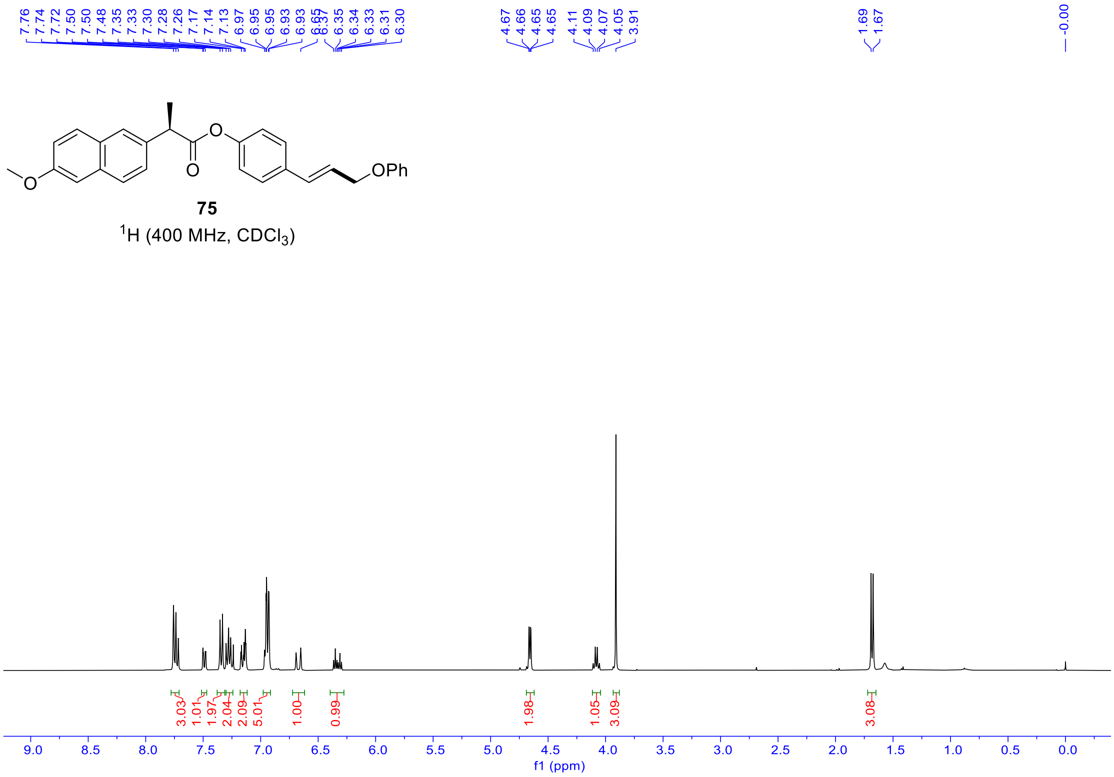

^{13}C NMR spectra of **75** (101 MHz, $CDCl_3$)

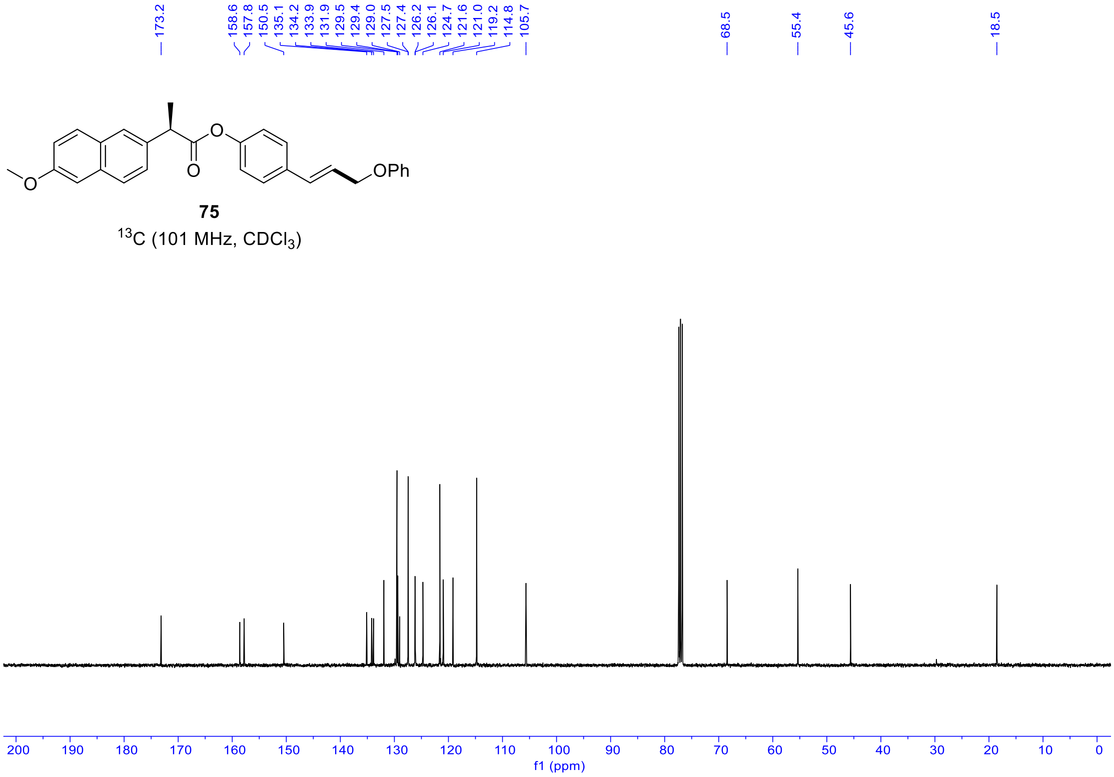

^{1}H NMR spectra of **76** (400 MHz, $CDCl_3$)

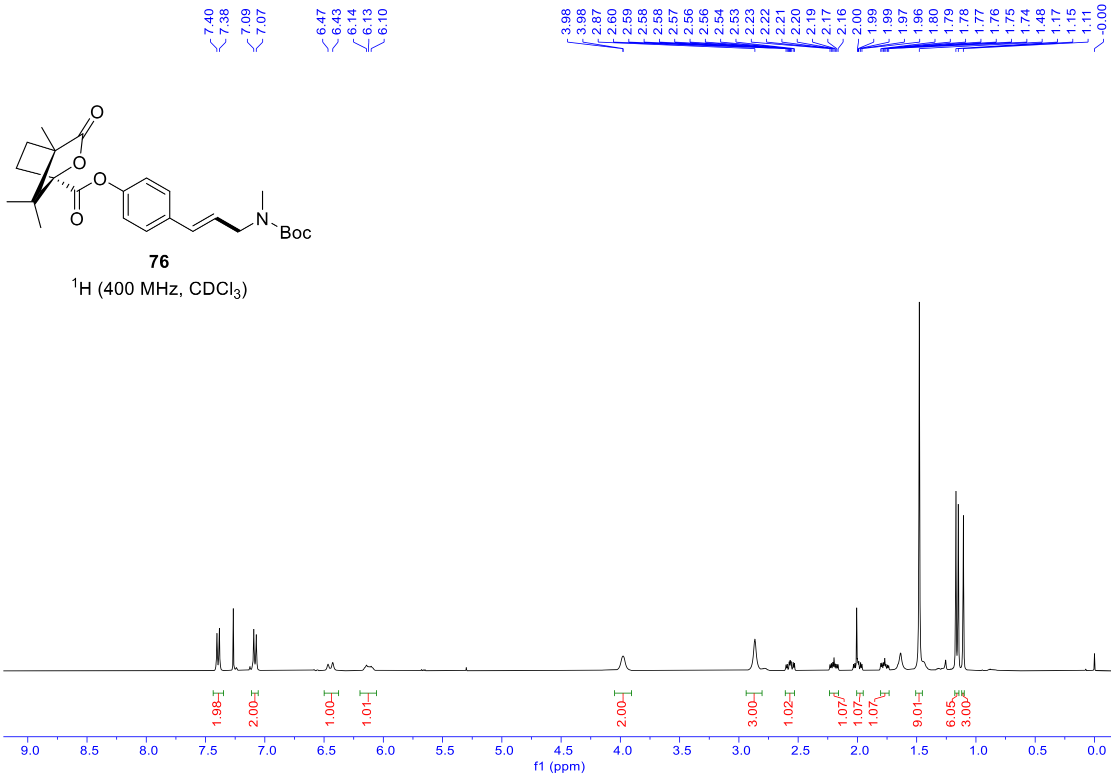

^{13}C NMR spectra of **76** (101 MHz, $CDCl_3$)

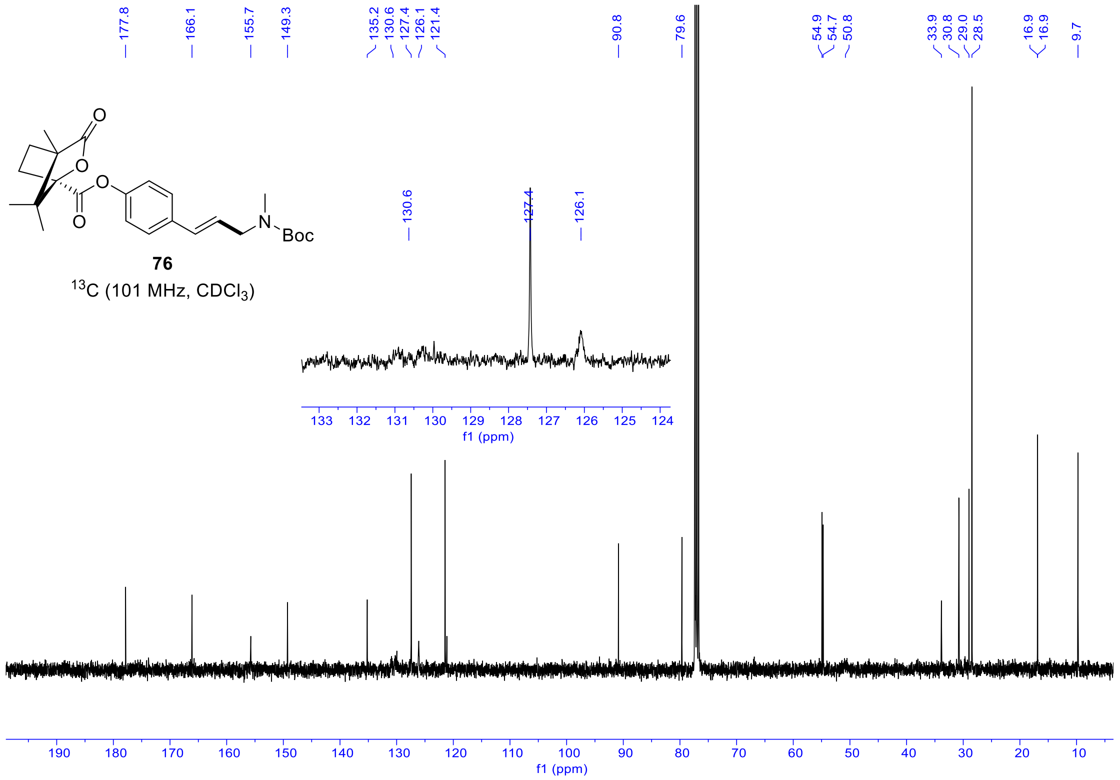

^{1}H NMR spectra of **77** (400 MHz, $CDCl_3$)

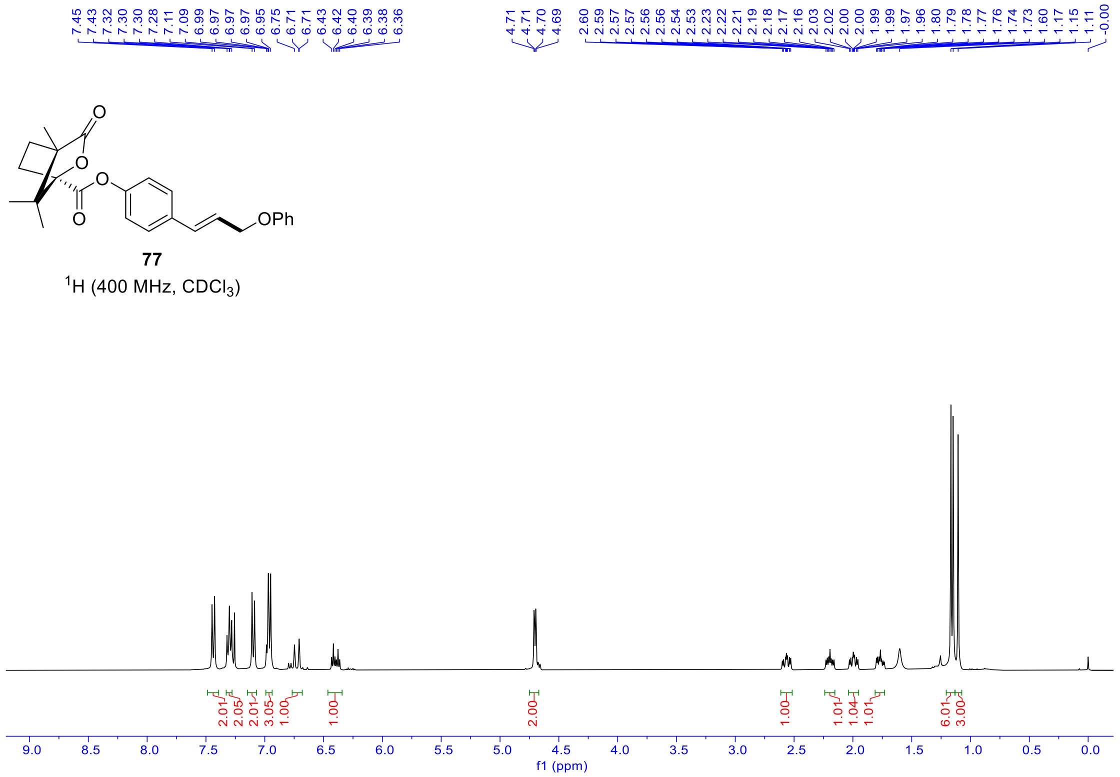

^{13}C NMR spectra of **77** (101 MHz, CDCl$_3$)

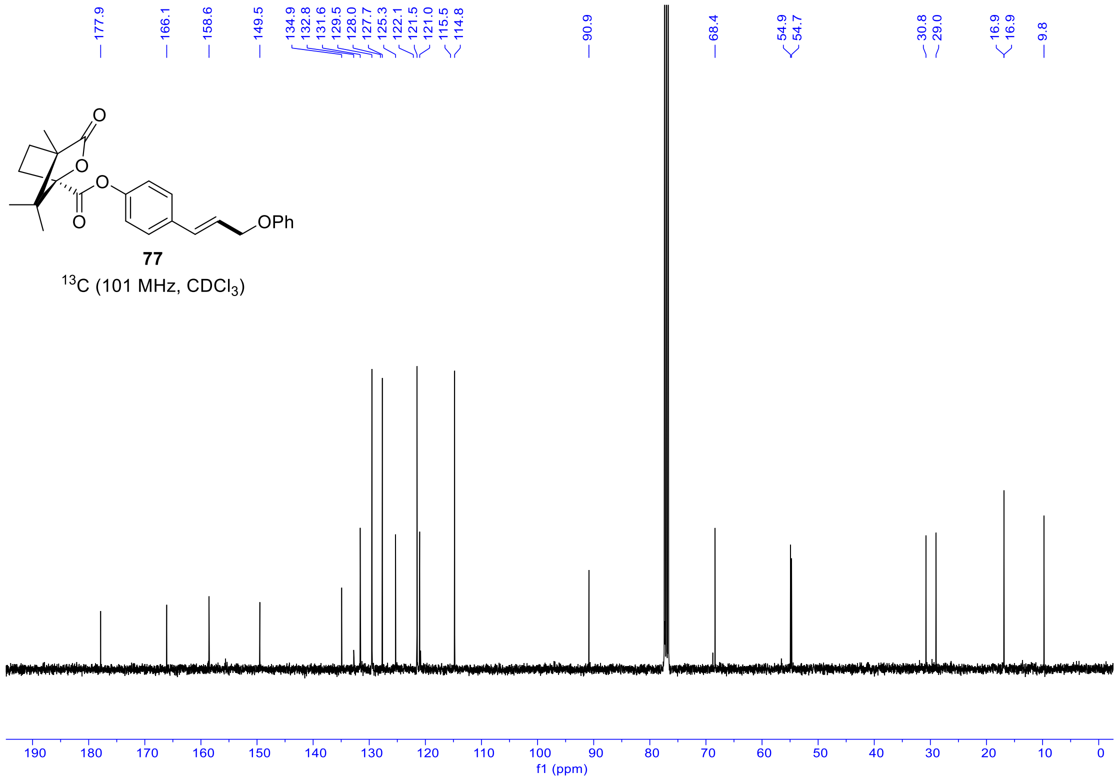

^{1}H NMR spectra of **78** (400 MHz, CDCl$_3$)

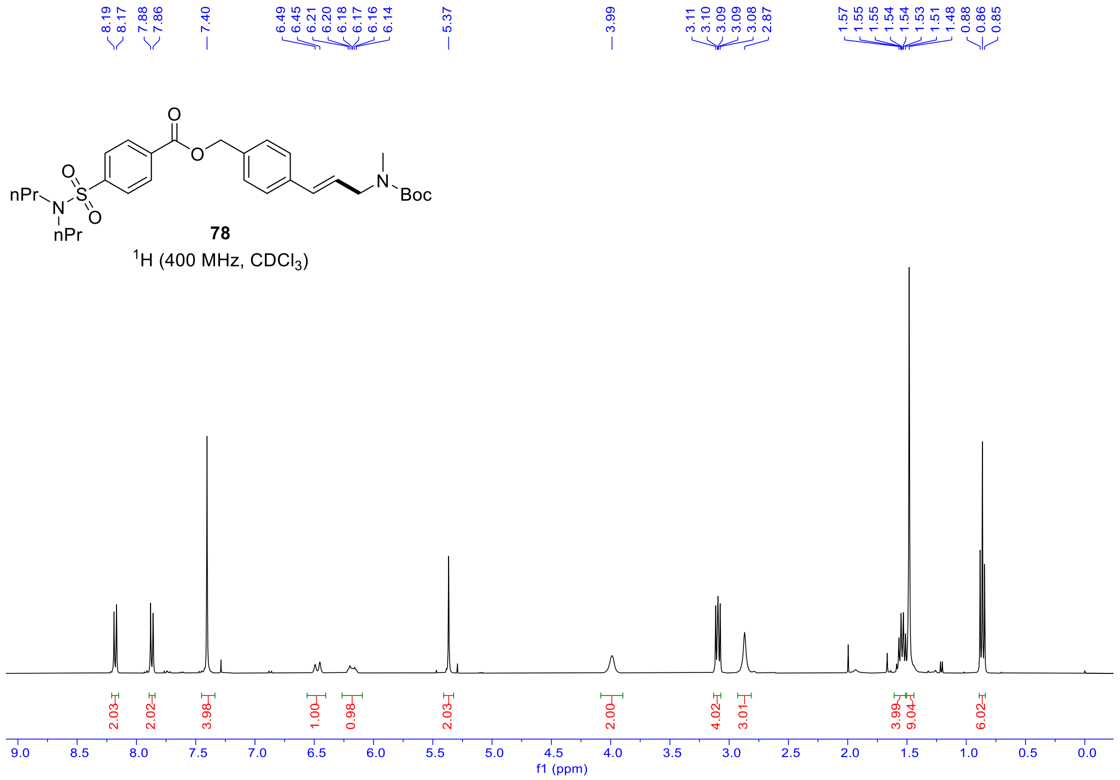

^{13}C NMR spectra of **78** (101 MHz, CDCl$_3$)

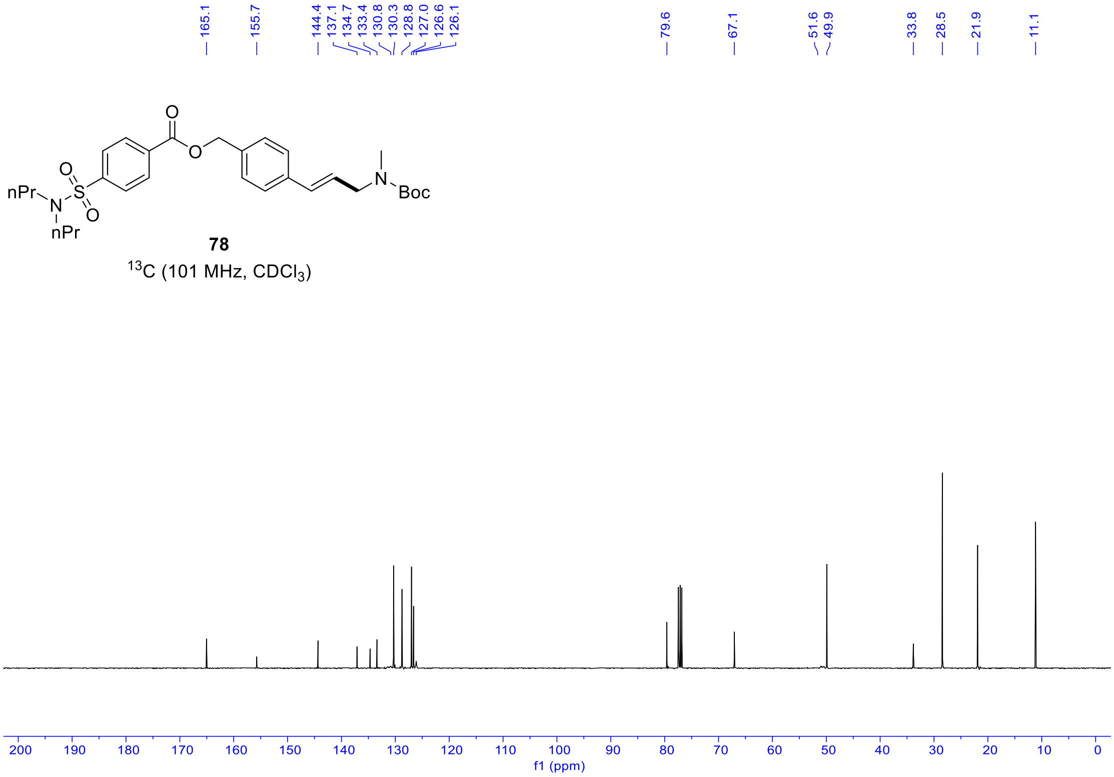

^{1}H NMR spectra of **79** (400 MHz, CDCl$_3$)

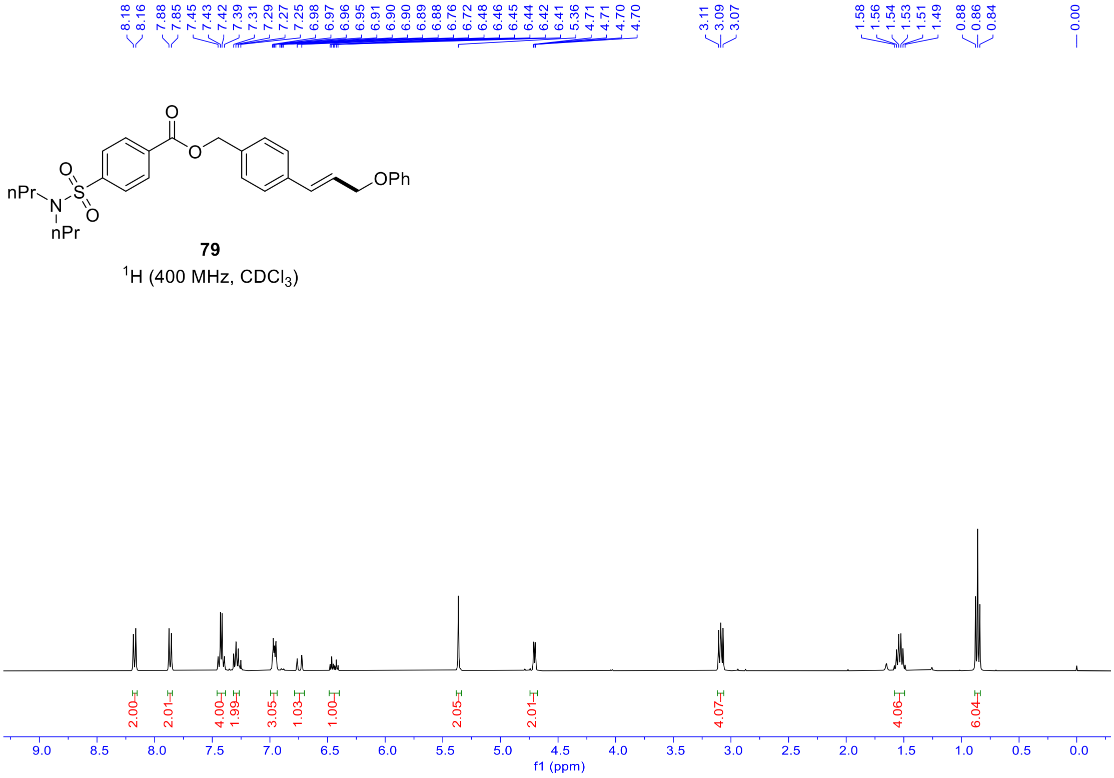

^{13}C NMR spectra of **79** (101 MHz, $CDCl_3$)

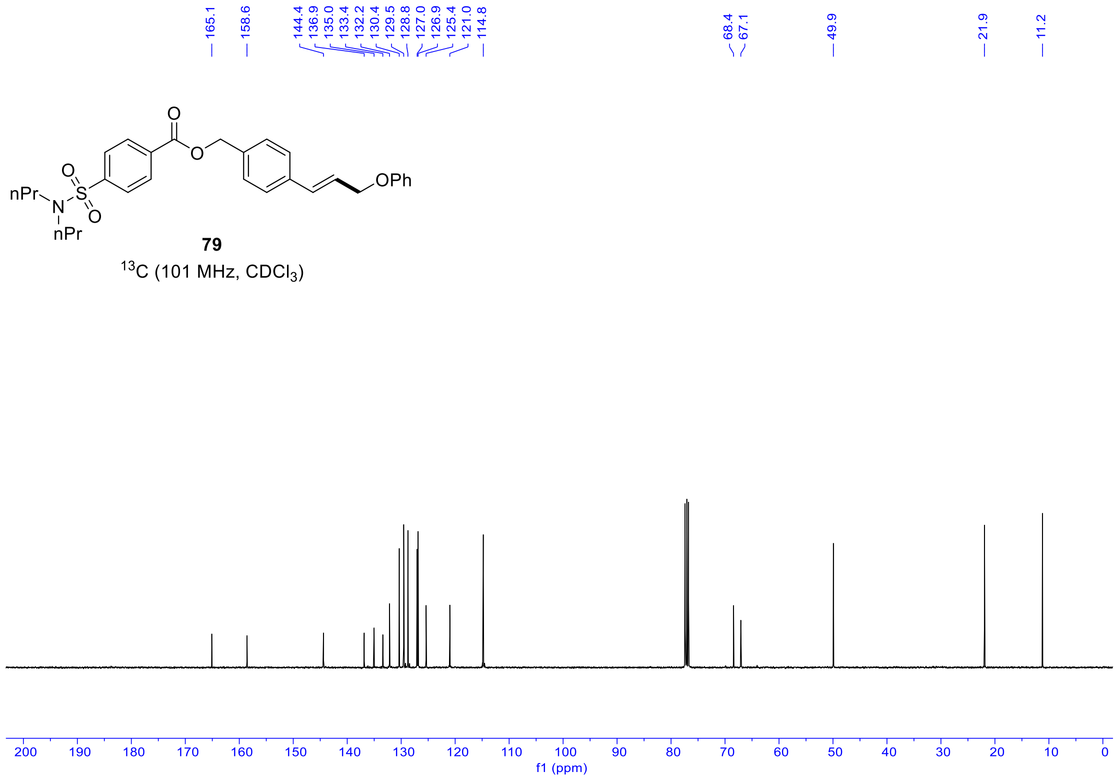

^{1}H NMR spectra of **80** (400 MHz, $CDCl_3$)

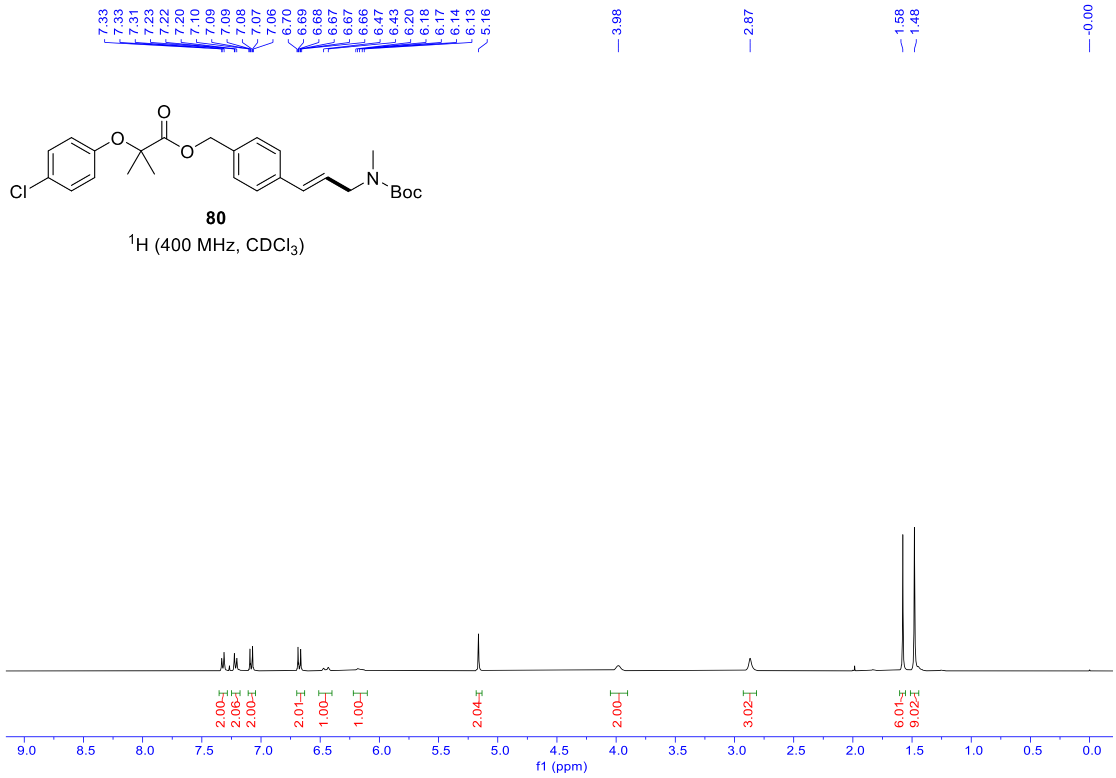

^{13}C NMR spectra of **80** (101 MHz, $CDCl_3$)

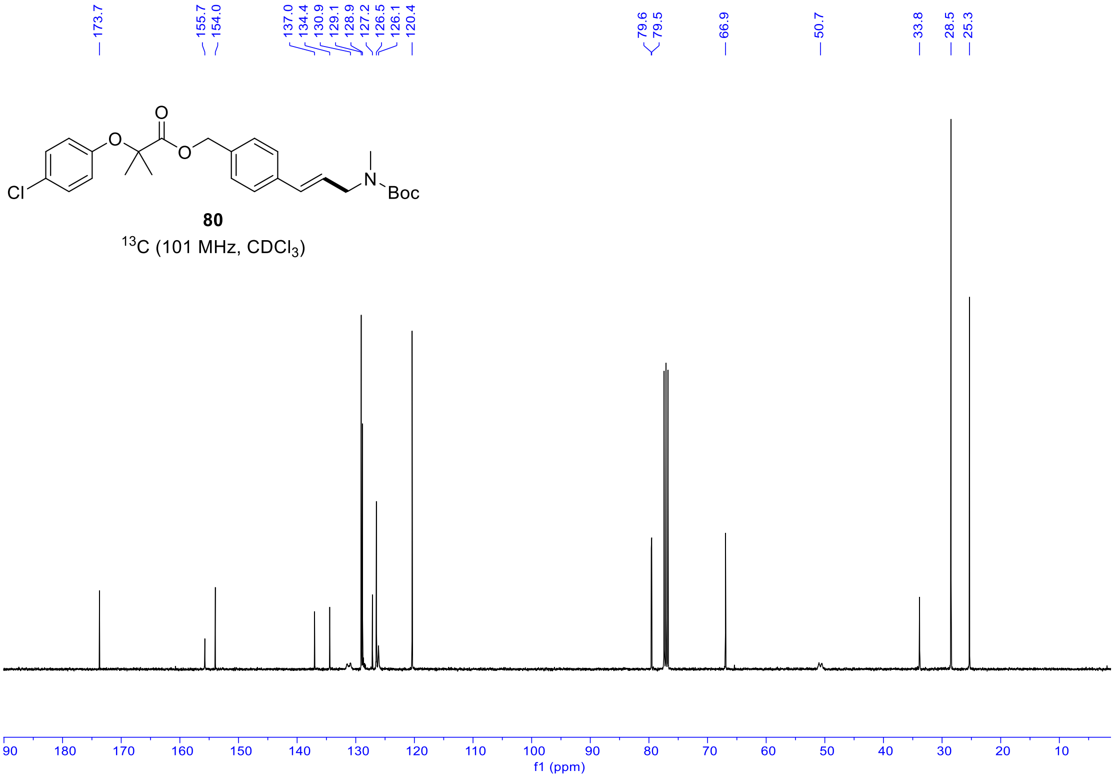

^{1}H NMR spectra of **81** (400 MHz, $CDCl_3$)

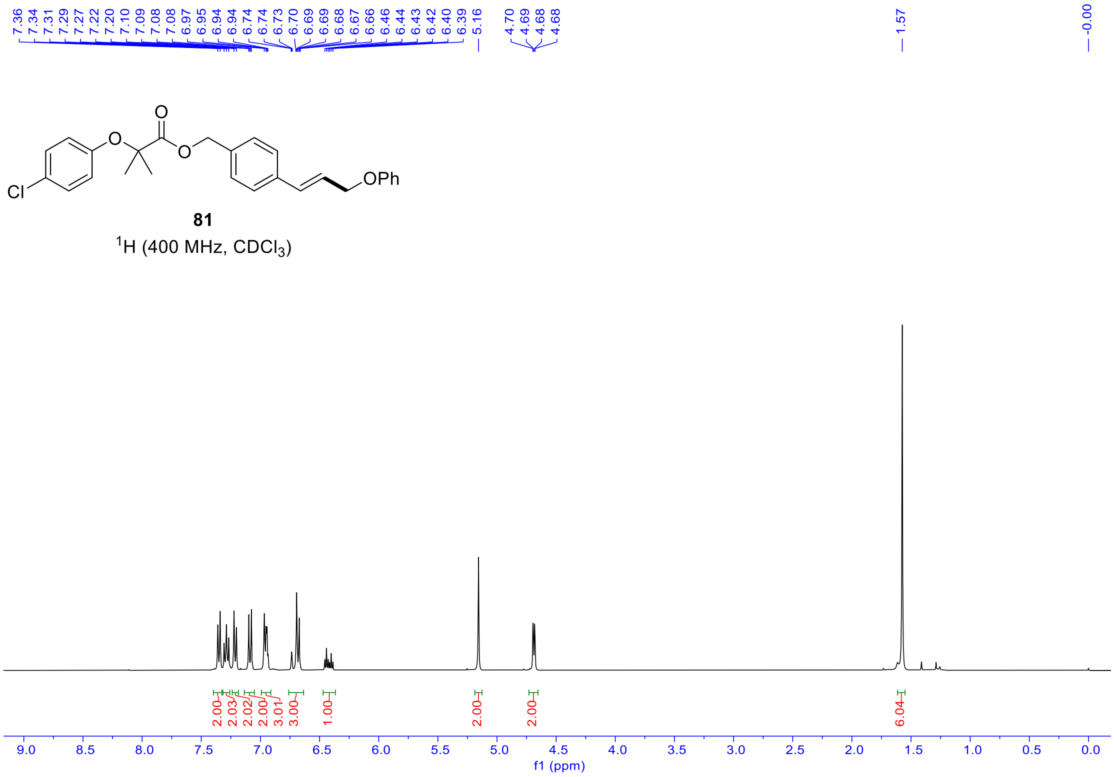

^{13}C NMR spectra of **81** (101 MHz, CDCl$_3$)

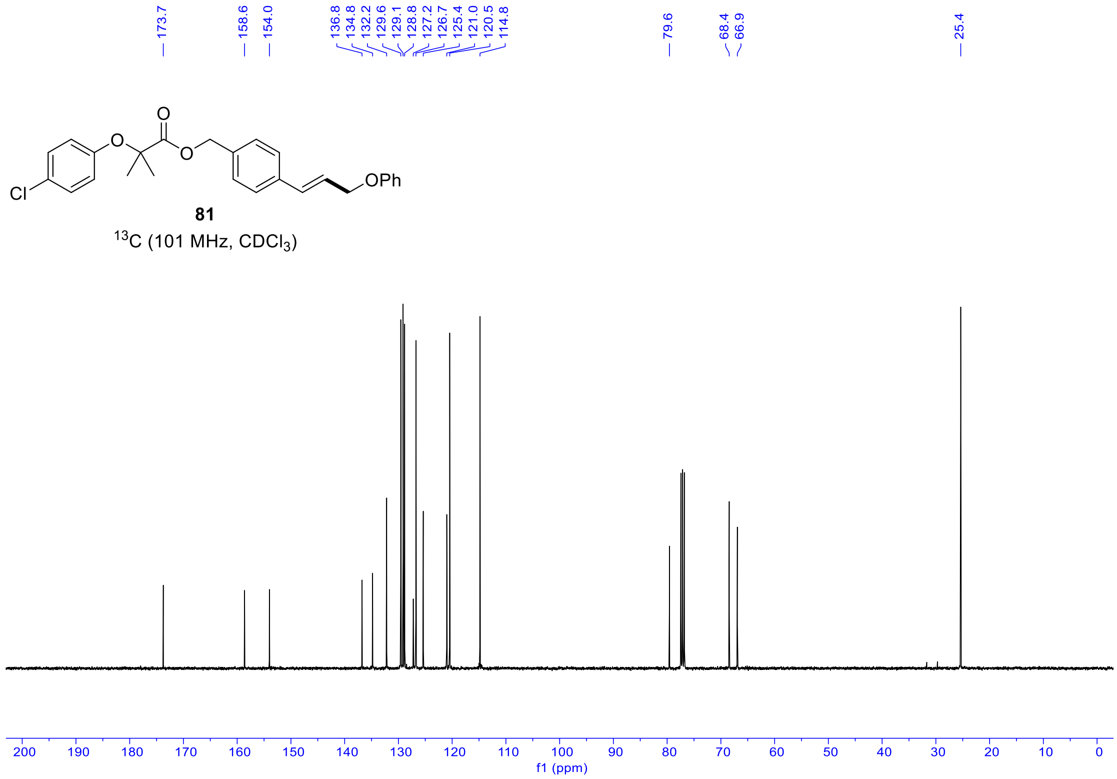

^{1}H NMR spectra of **82** (400 MHz, CDCl$_3$)

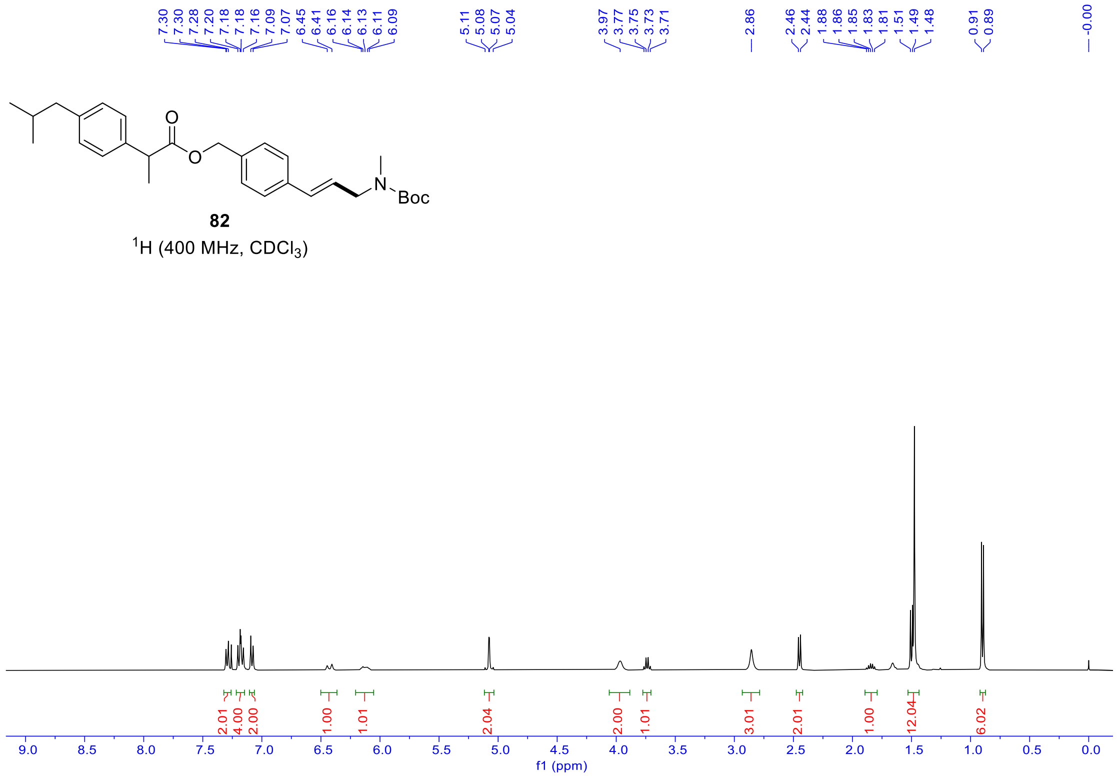

^{13}C NMR spectra of **82** (101 MHz, $CDCl_3$)

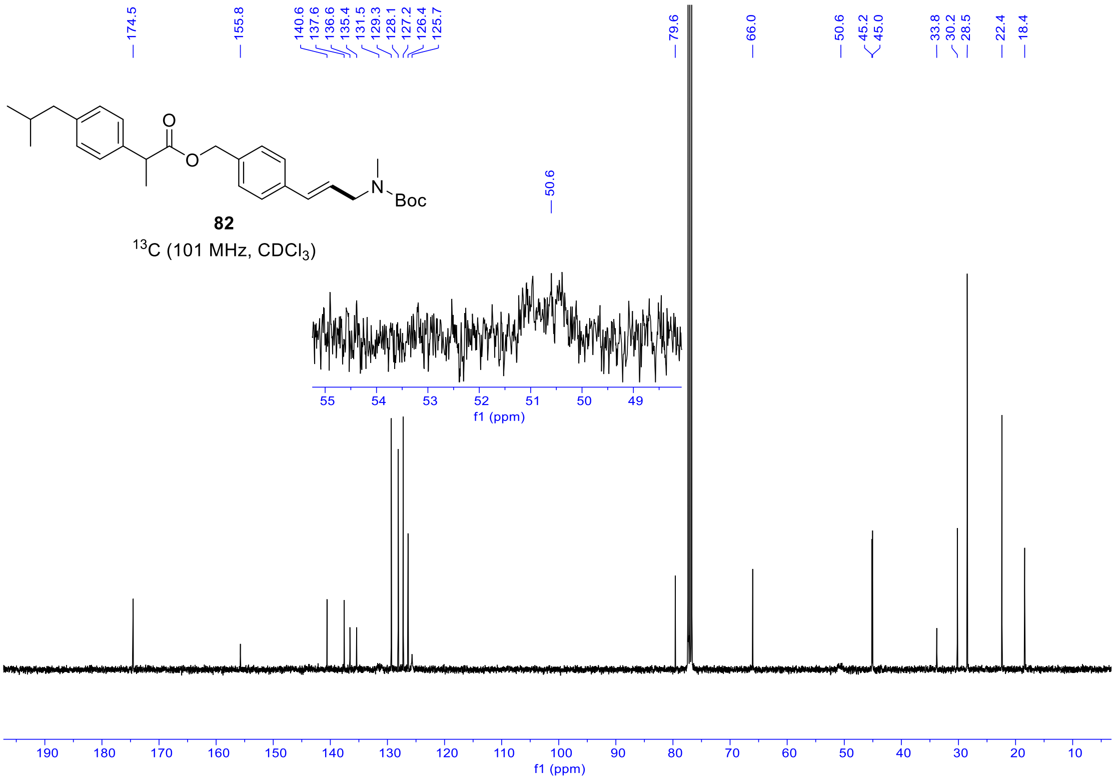

^{1}H NMR spectra **83** (400 MHz, $CDCl_3$)

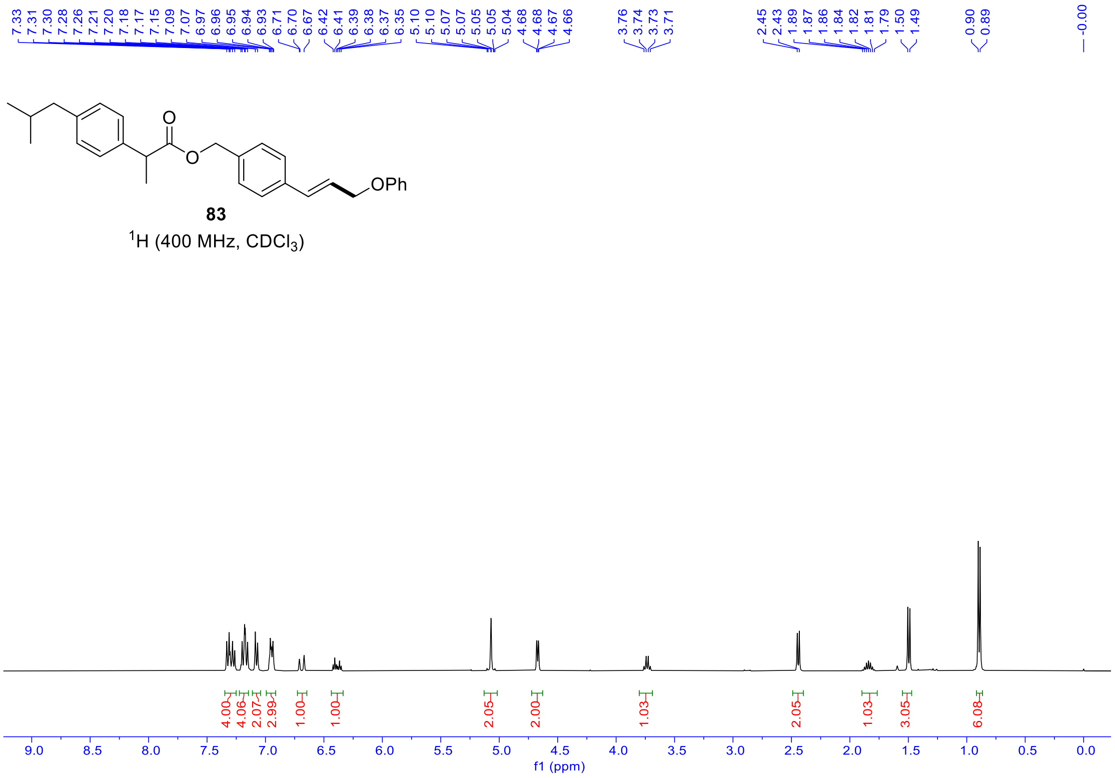

^{13}C NMR spectra of **83** (101 MHz, $CDCl_3$)

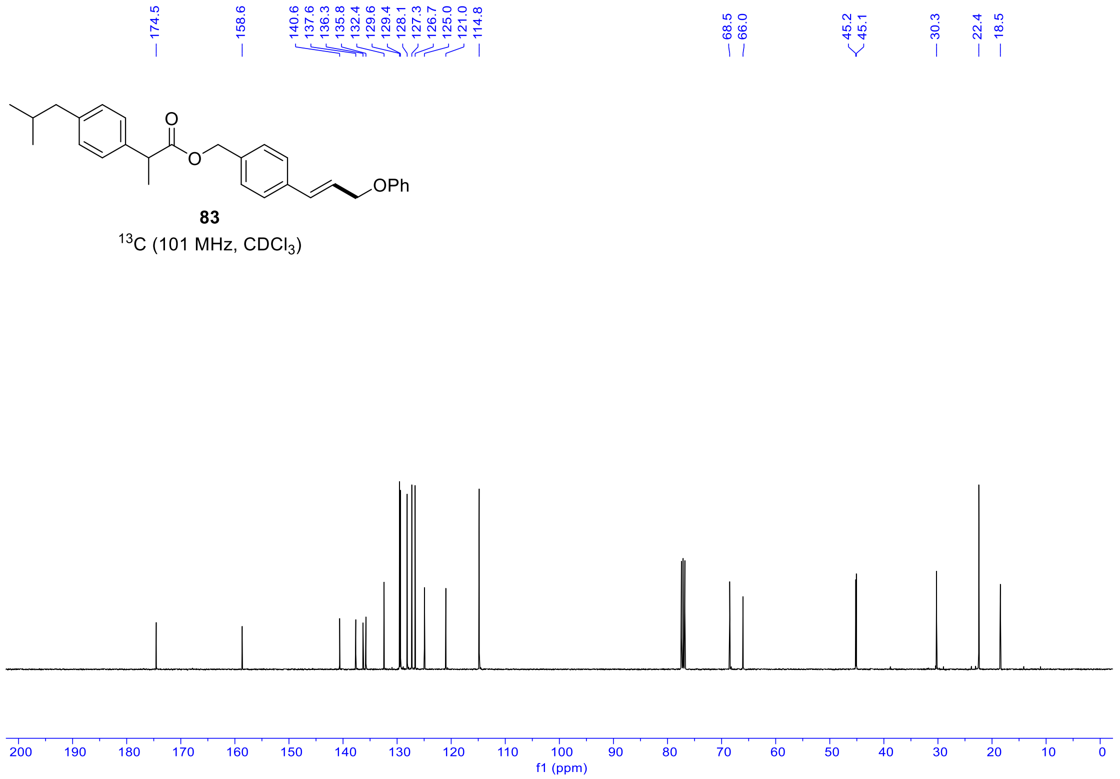

^{1}H NMR spectra (400 MHz, $CDCl_3$)

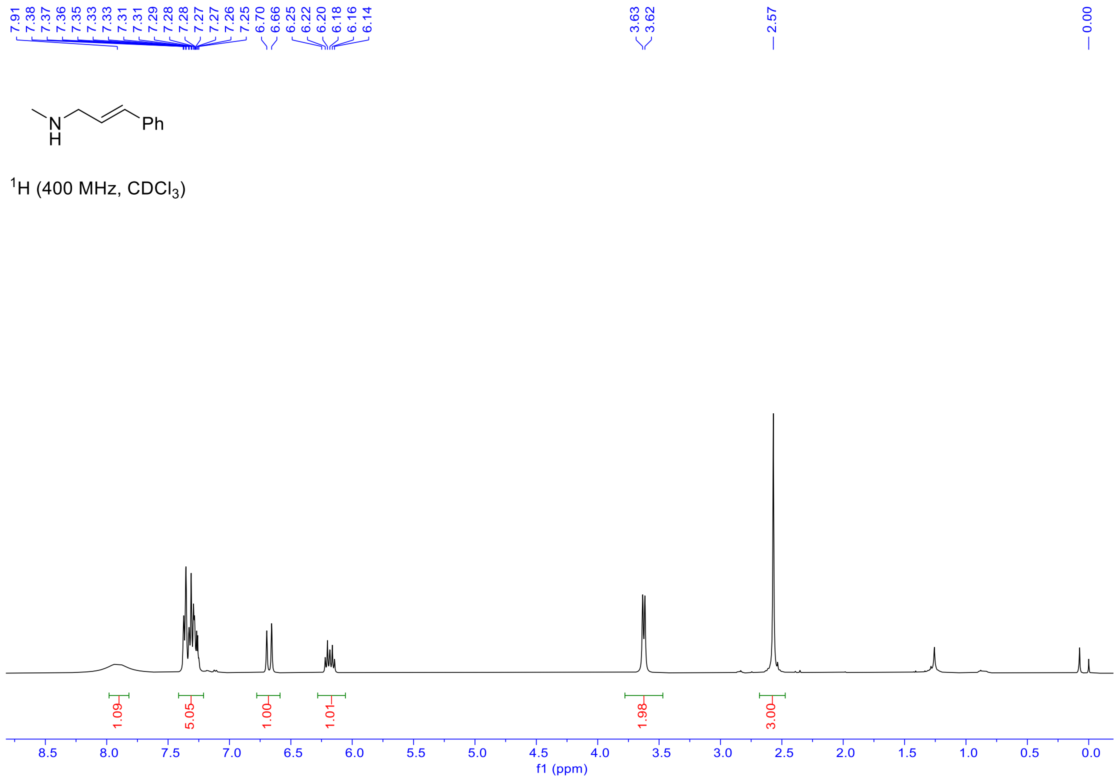

^{13}C NMR spectra (101 MHz, $CDCl_3$)

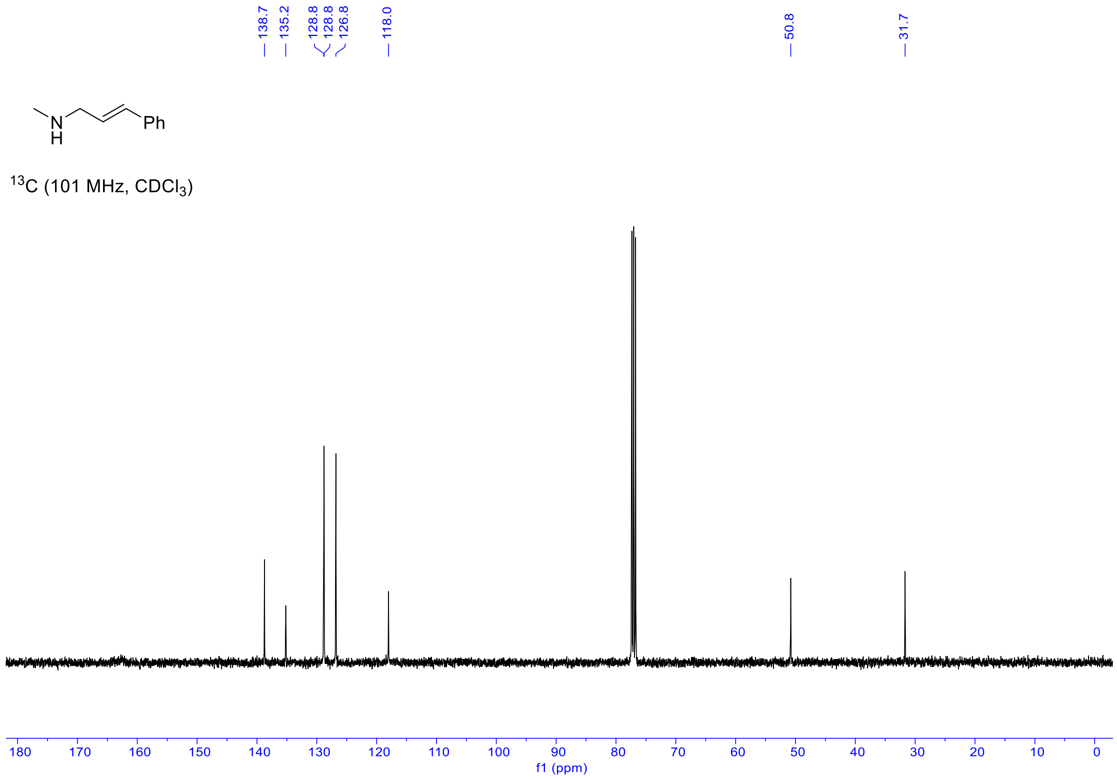

^{1}H NMR spectra of **84** (400 MHz, $CDCl_3$)

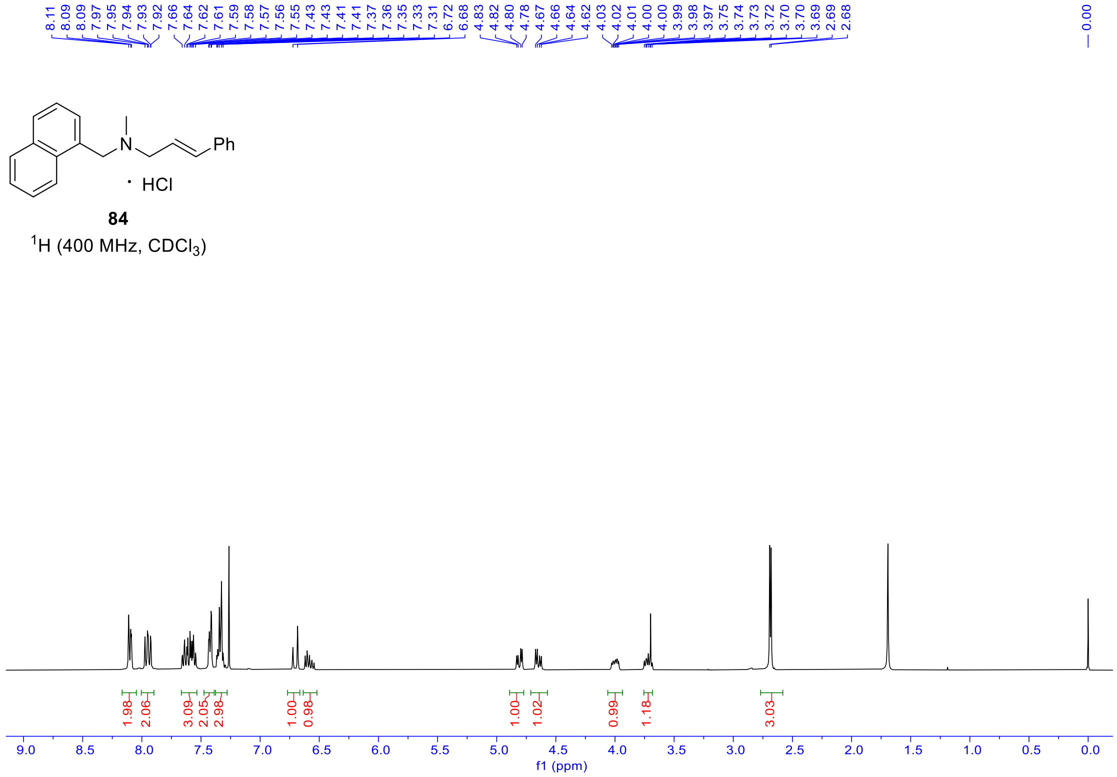

^{13}C NMR spectra of **84** (101 MHz, $CDCl_3$)

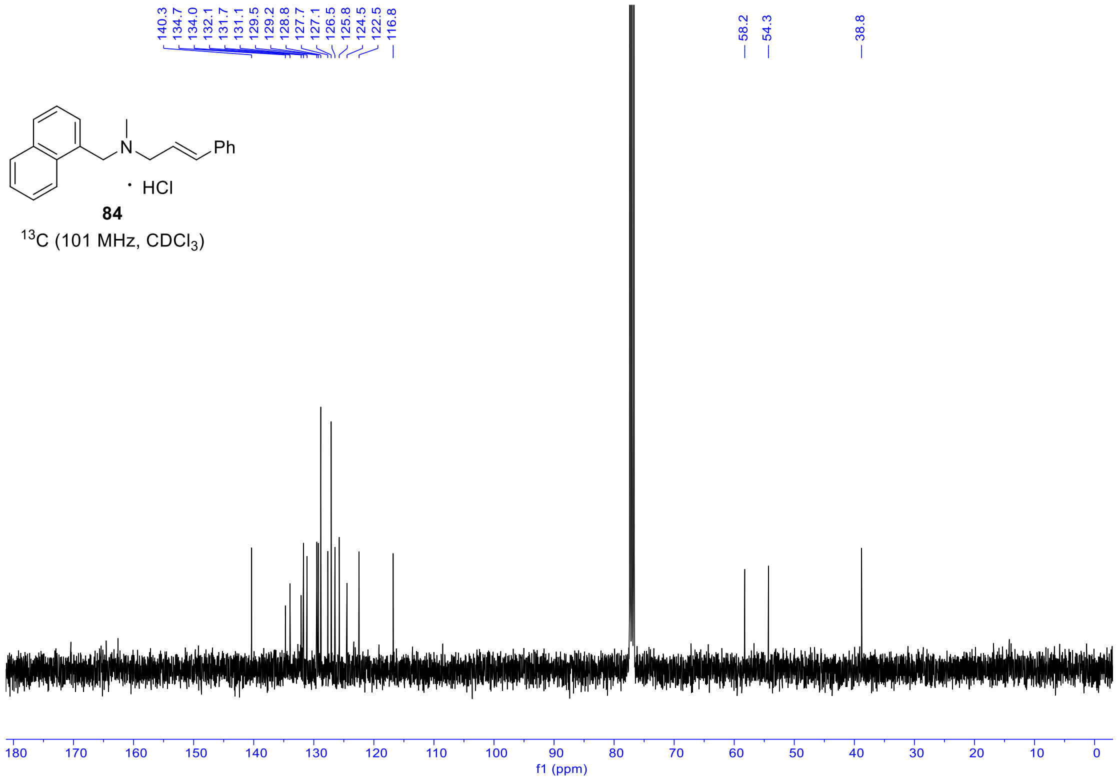

^{1}H NMR spectra (400 MHz, $CDCl_3$)

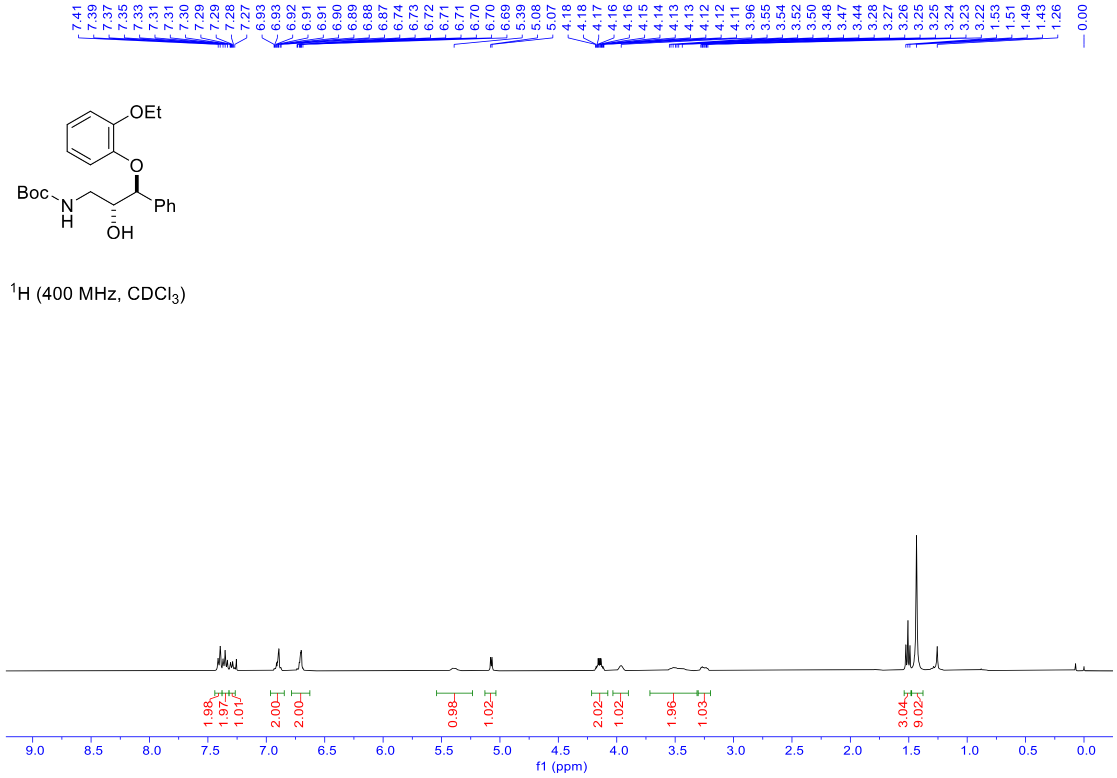

^{13}C NMR spectra (101 MHz, $CDCl_3$)

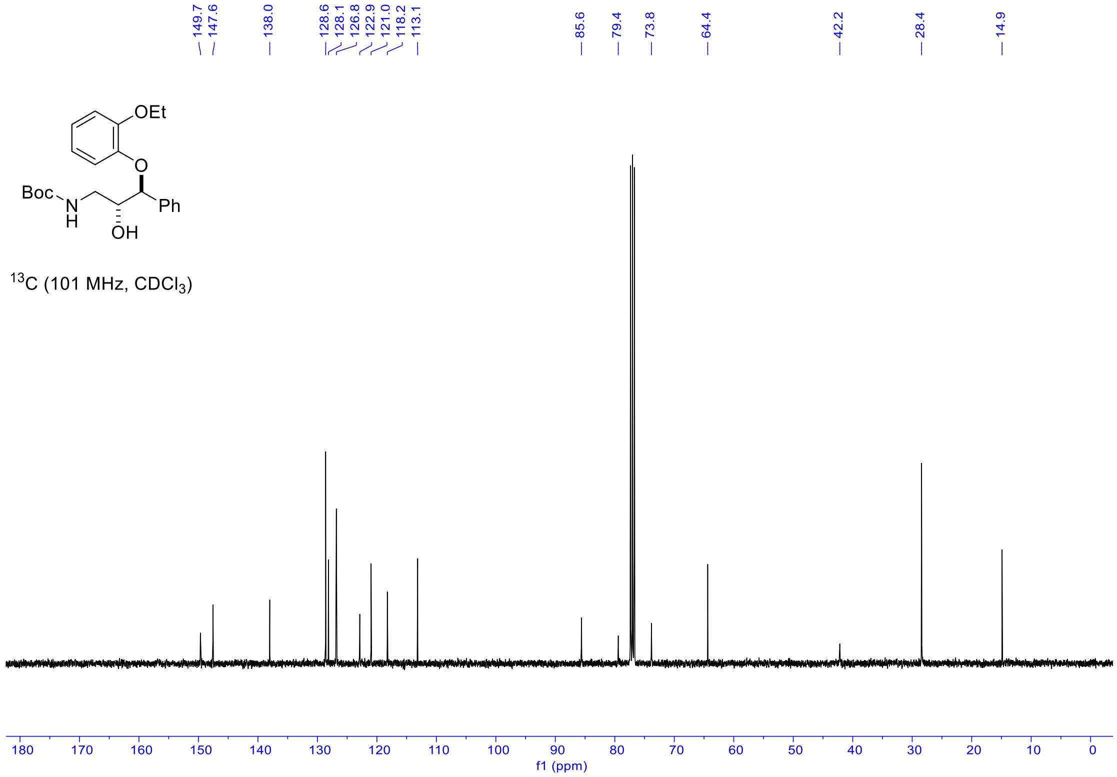

^{1}H NMR spectra of **85** (400 MHz, $CDCl_3$)

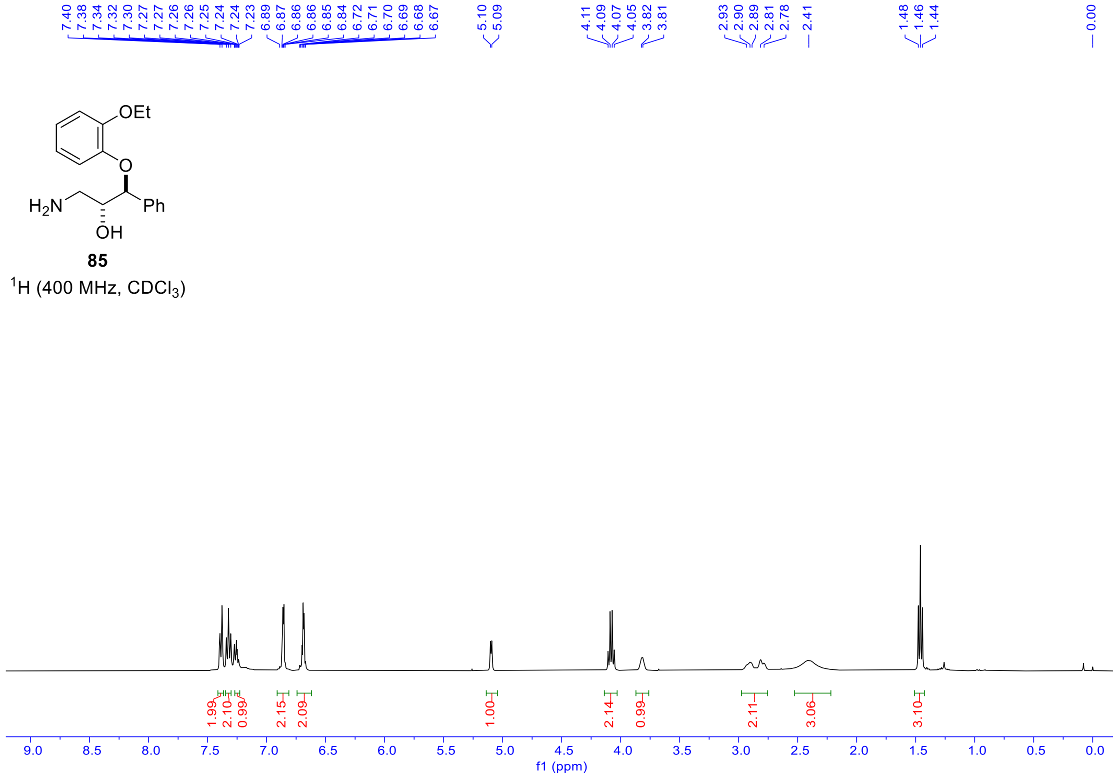

^{13}C NMR spectra of **85** (101 MHz, $CDCl_3$)

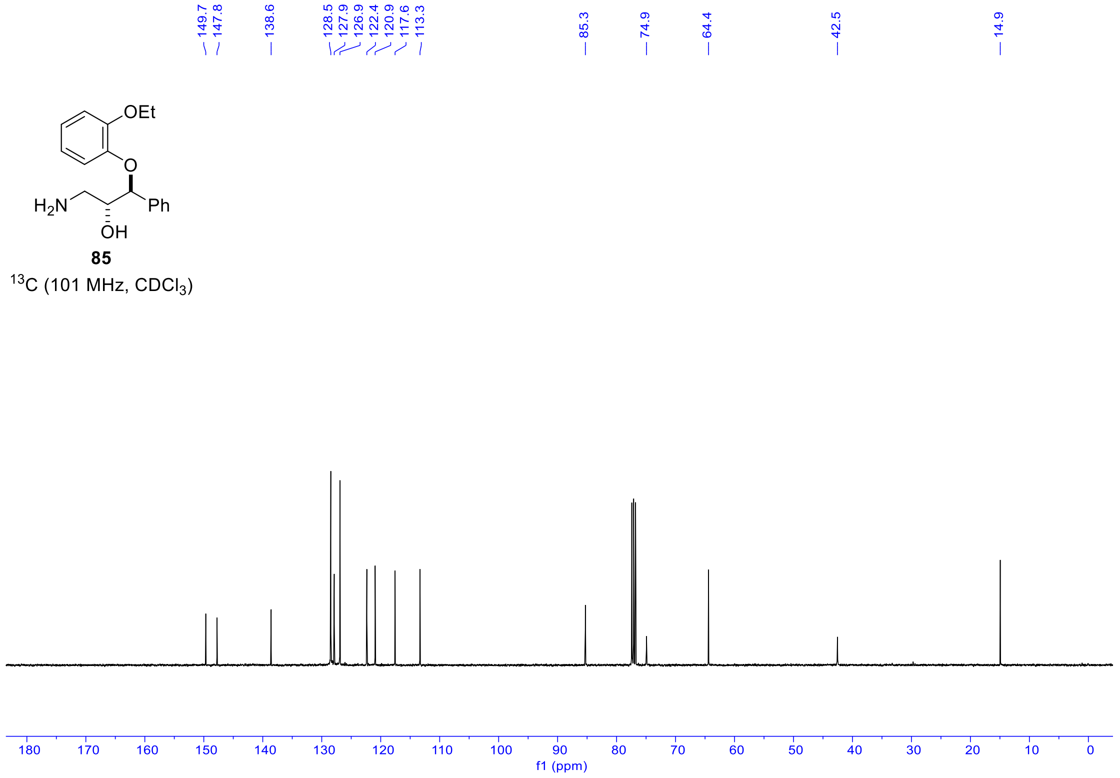

^1H NMR spectra (400 MHz, $CDCl_3$)

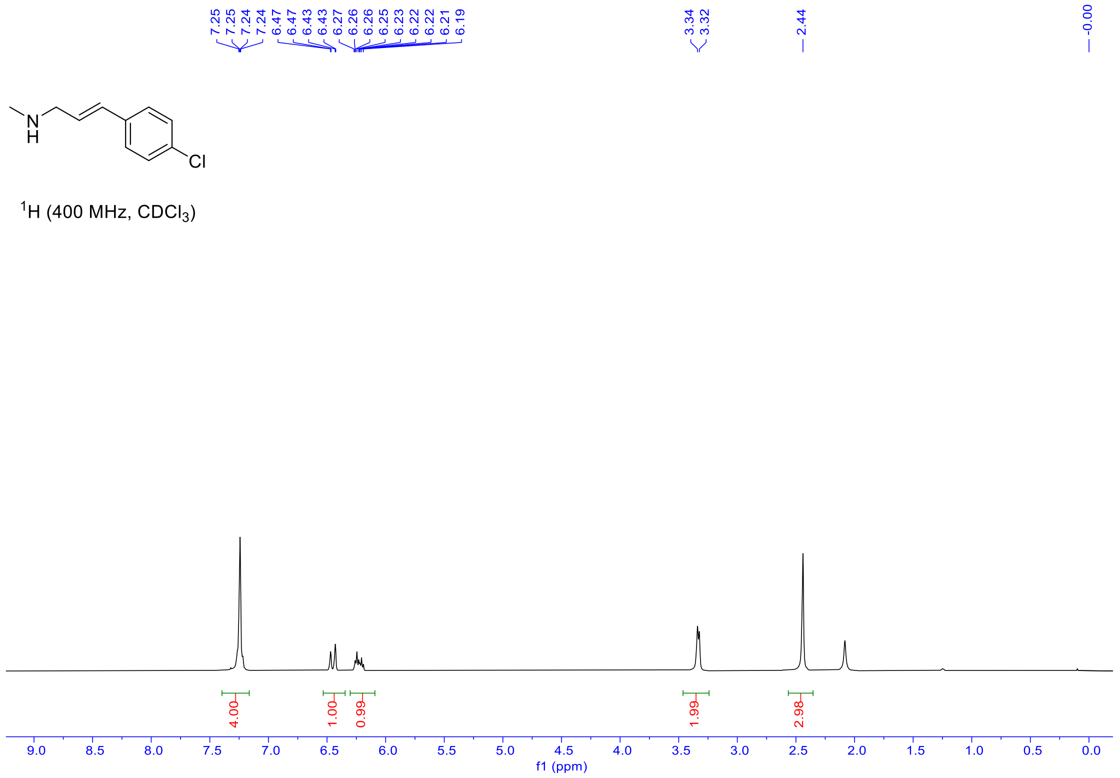

^{13}C NMR spectra (101 MHz, $CDCl_3$)

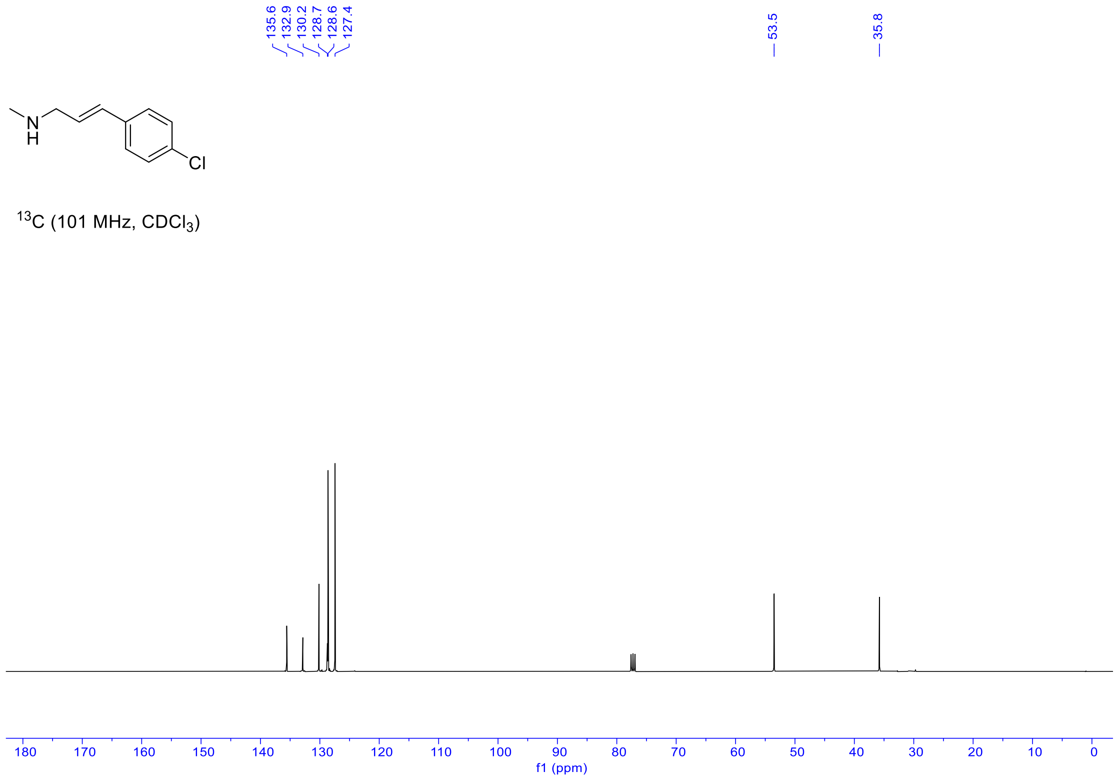

^{1}H NMR spectra of **86** (400 MHz, $CDCl_3$)

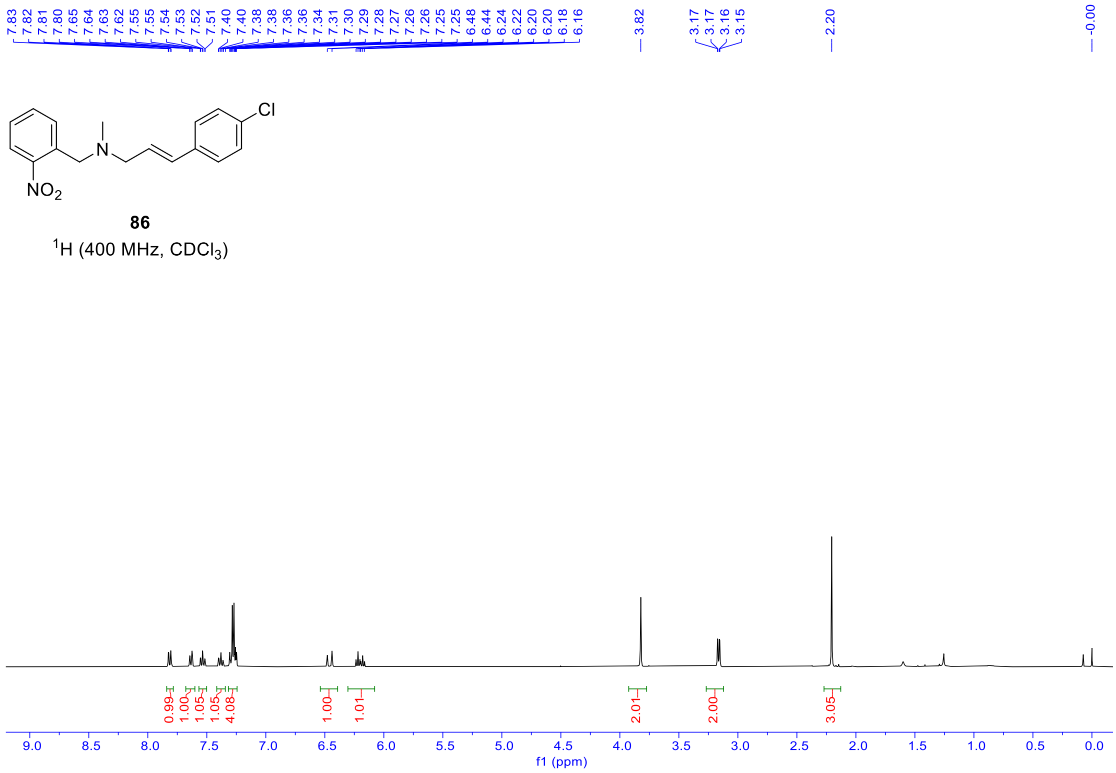

^{13}C NMR spectra of **86** (101 MHz, $CDCl_3$)

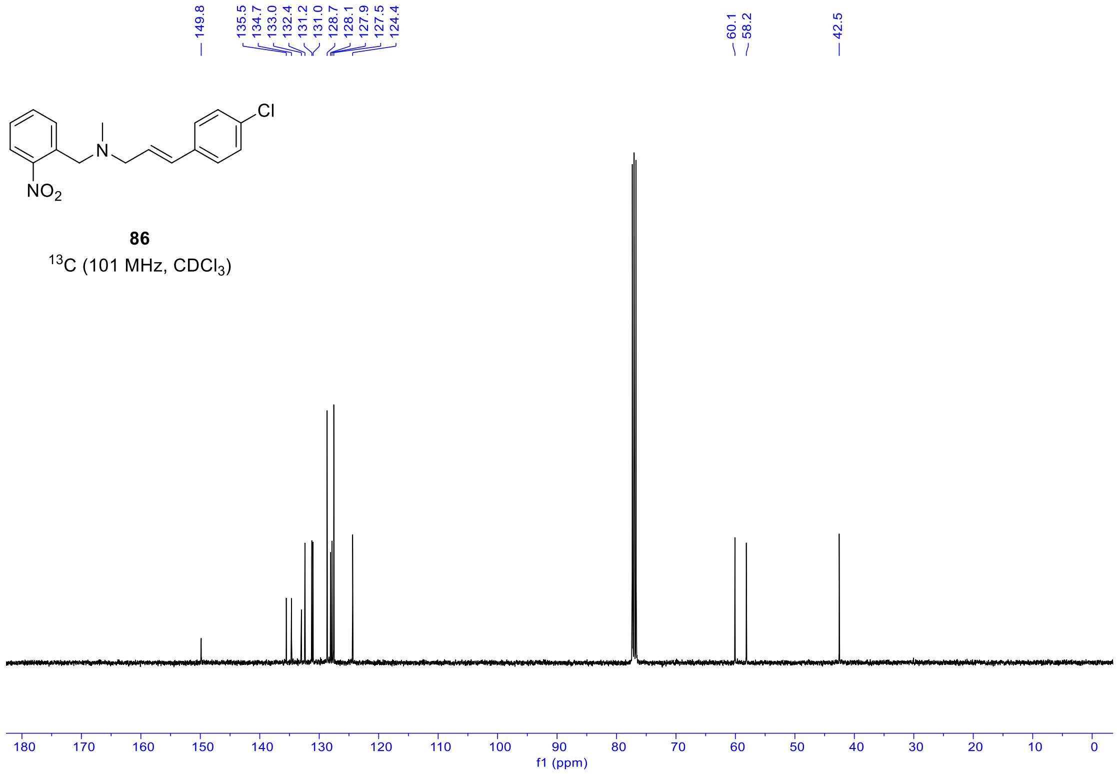

^{1}H NMR spectra (400 MHz, $CDCl_3$)

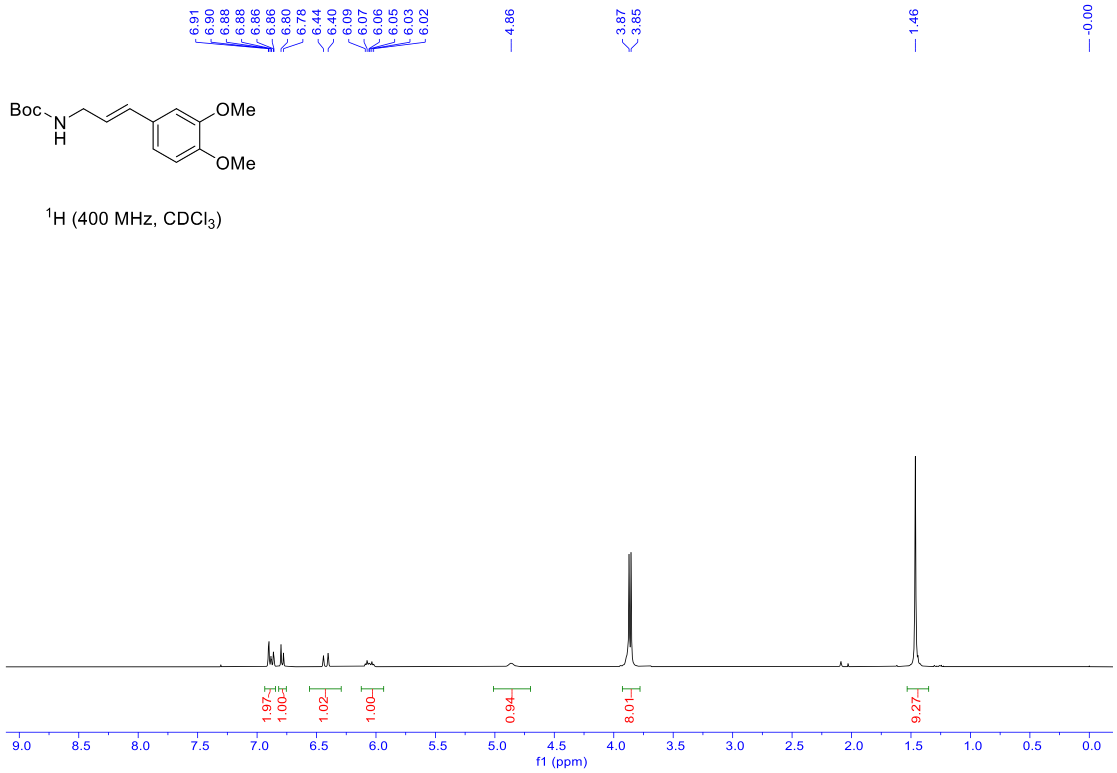

^{13}C NMR spectra (101 MHz, $CDCl_3$)

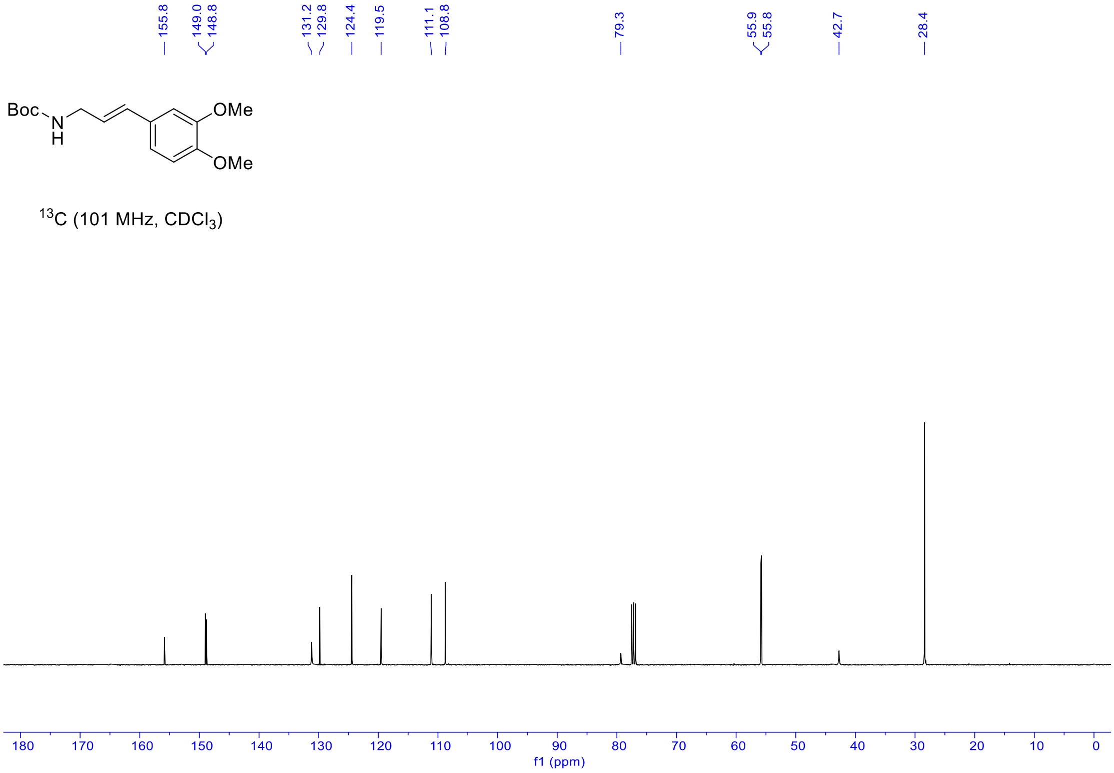

^{1}H NMR spectra (400 MHz, $CDCl_3$)

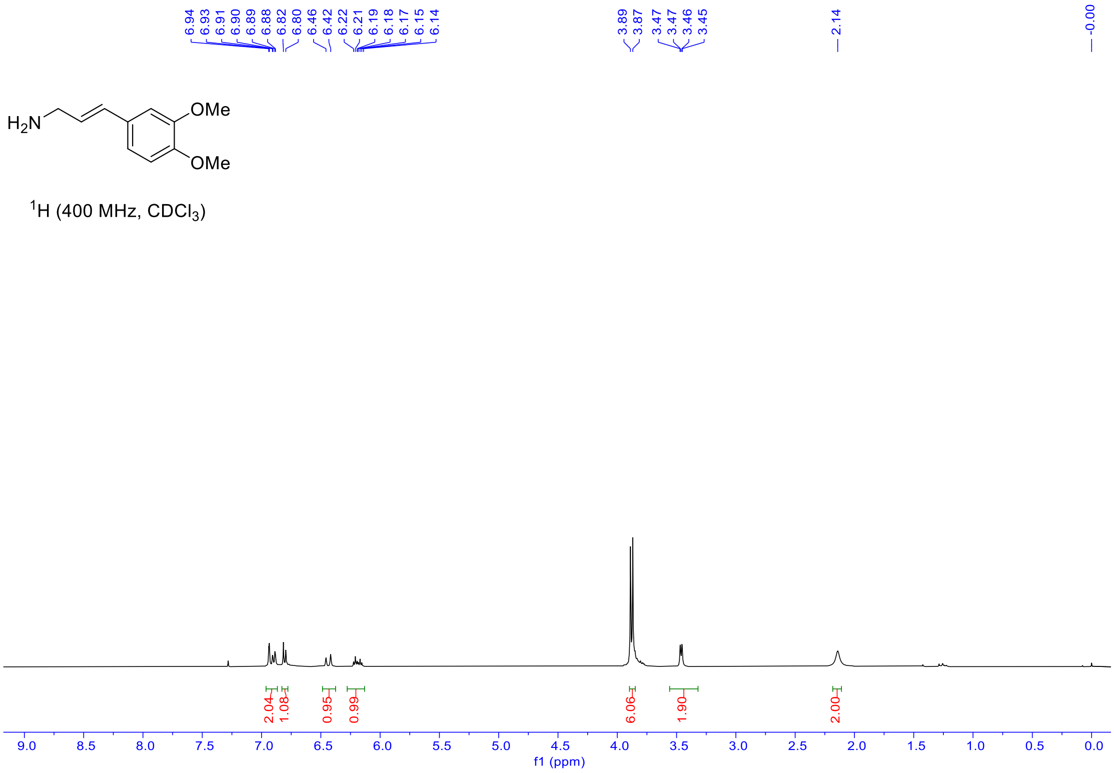

^{13}C NMR spectra (101 MHz, $CDCl_3$)

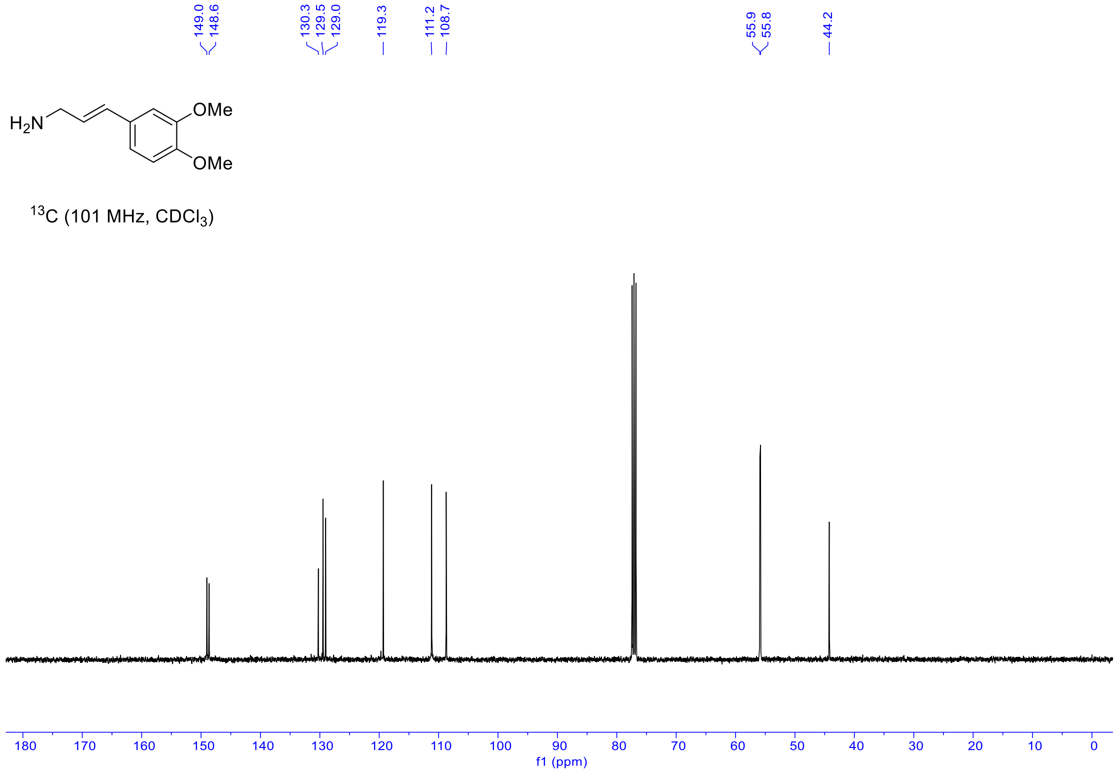

^{1}H NMR spectra (400 MHz, $CDCl_3$)

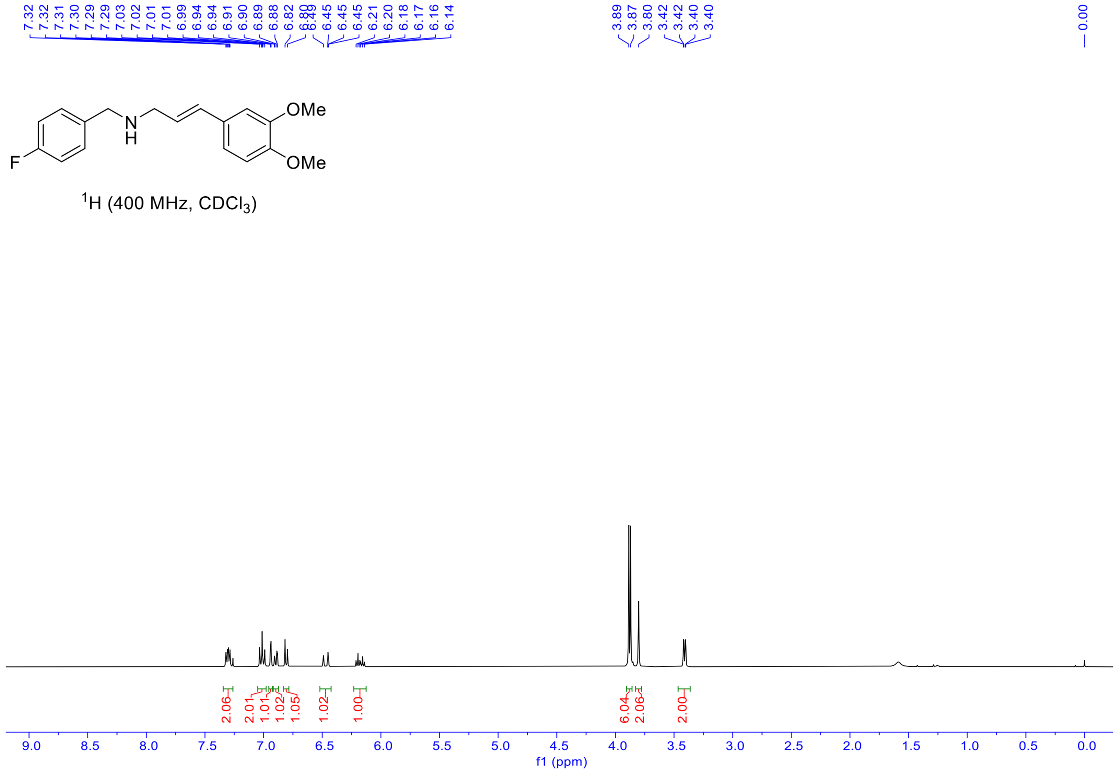

^{13}C NMR spectra (101 MHz, $CDCl_3$)

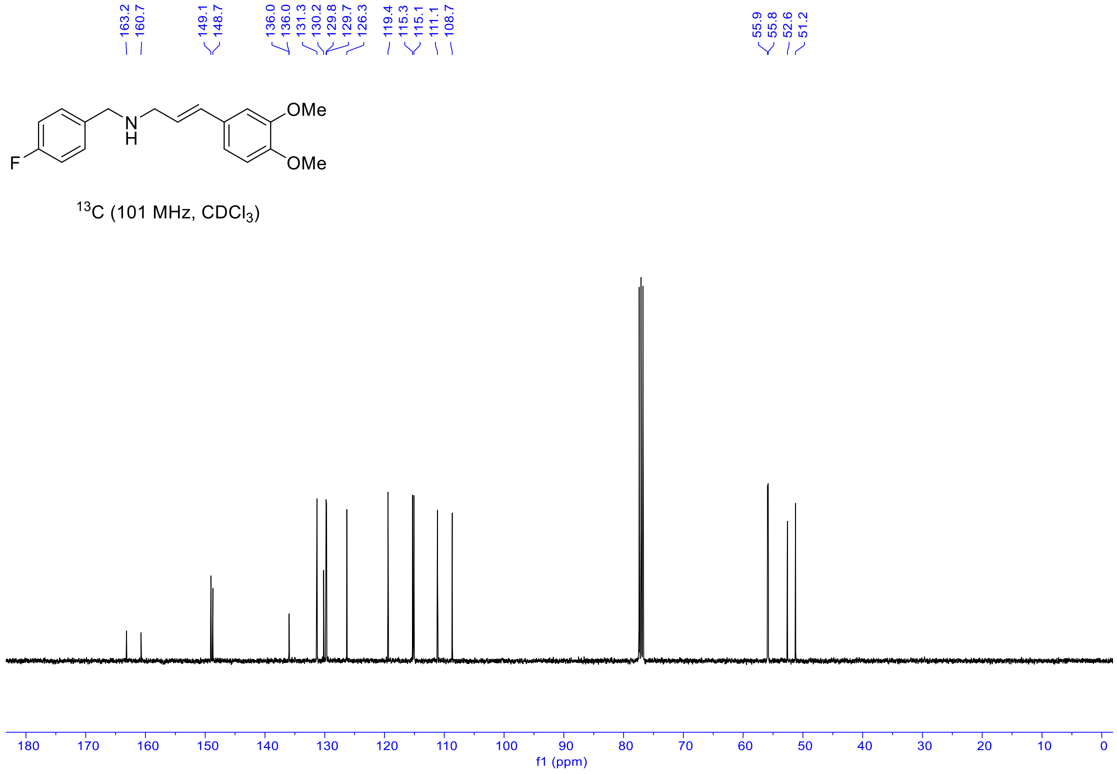

^{19}F NMR spectra (377 MHz, $CDCl_3$)

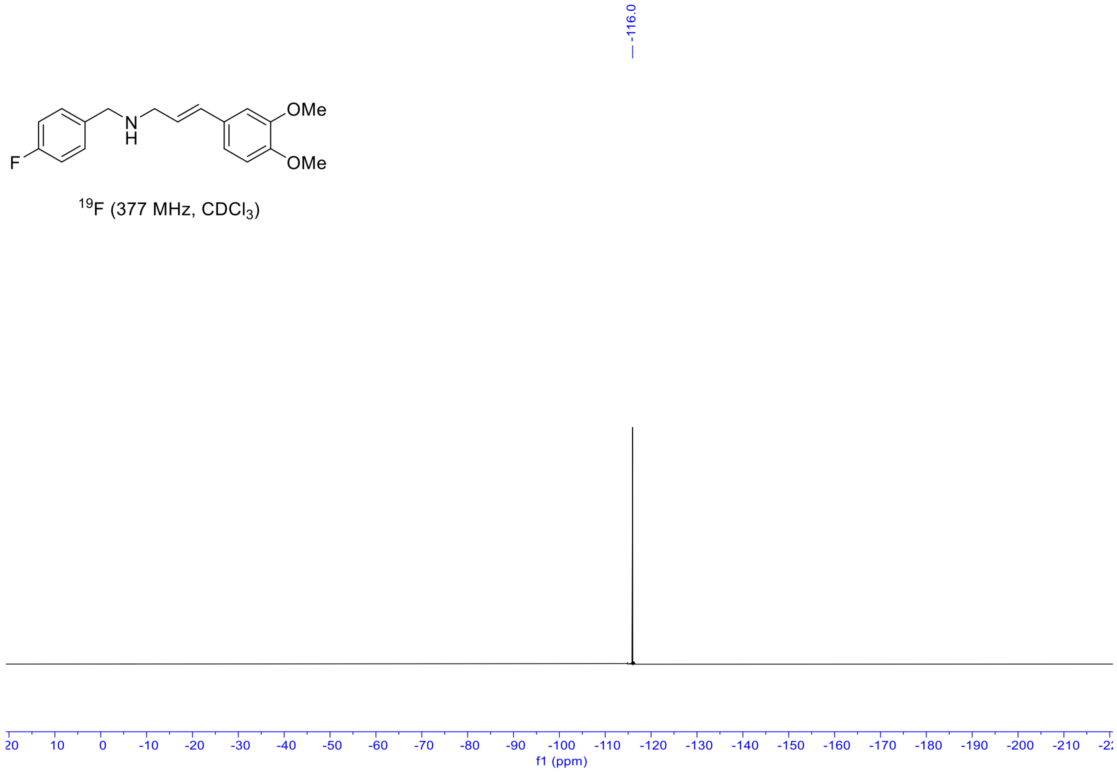

^{1}H NMR spectra of **87** (400 MHz, $CDCl_3$)

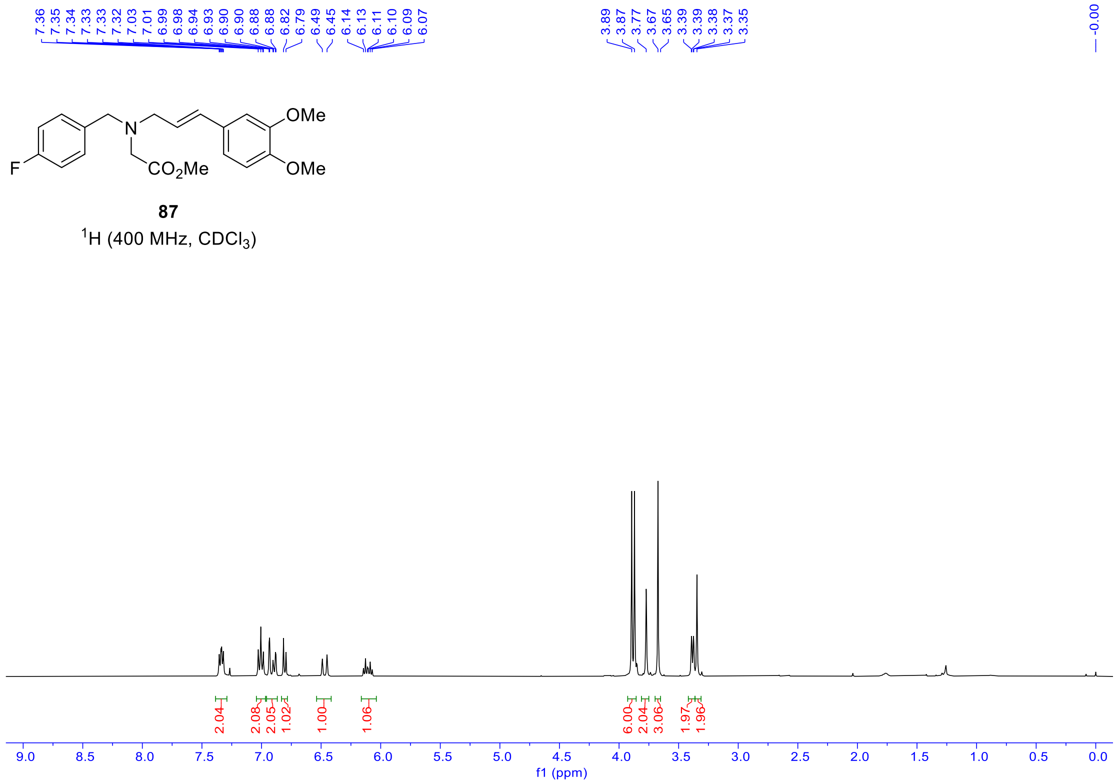

^{13}C NMR spectra of **87** (101 MHz, $CDCl_3$)

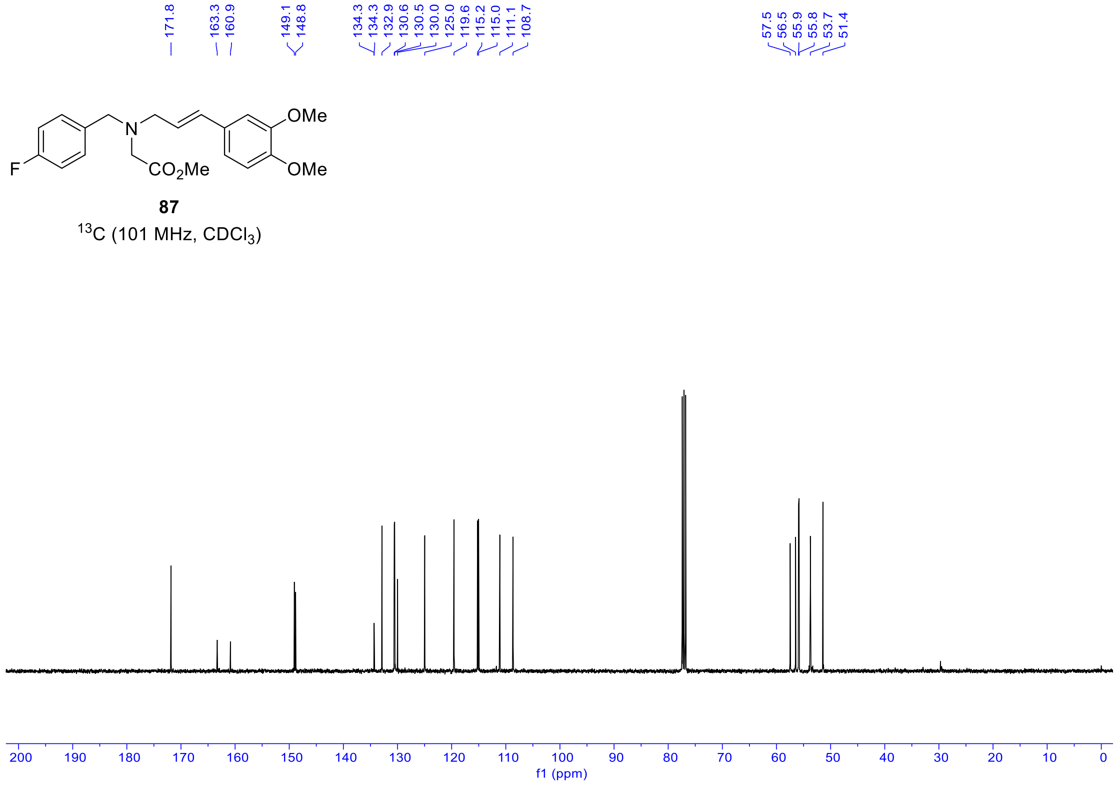

^{19}F NMR spectra of **87** (377 MHz, $CDCl_3$)

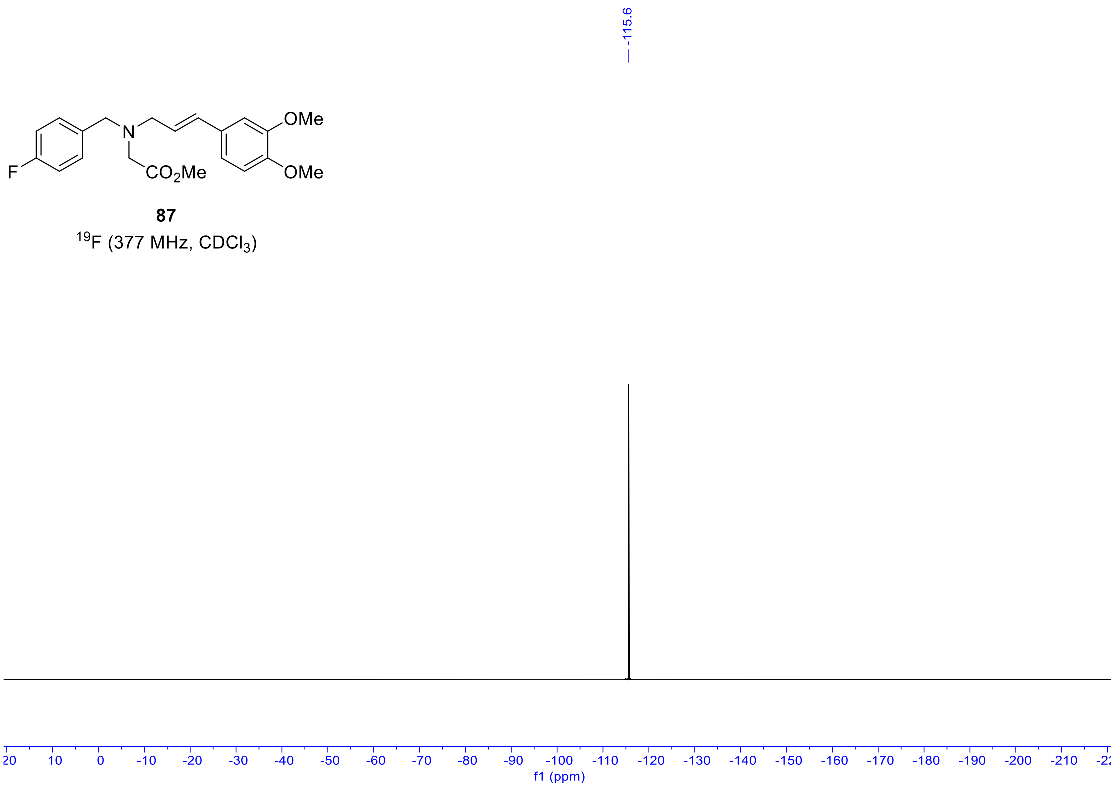

^{1}H NMR spectra (400 MHz, $CDCl_3$)

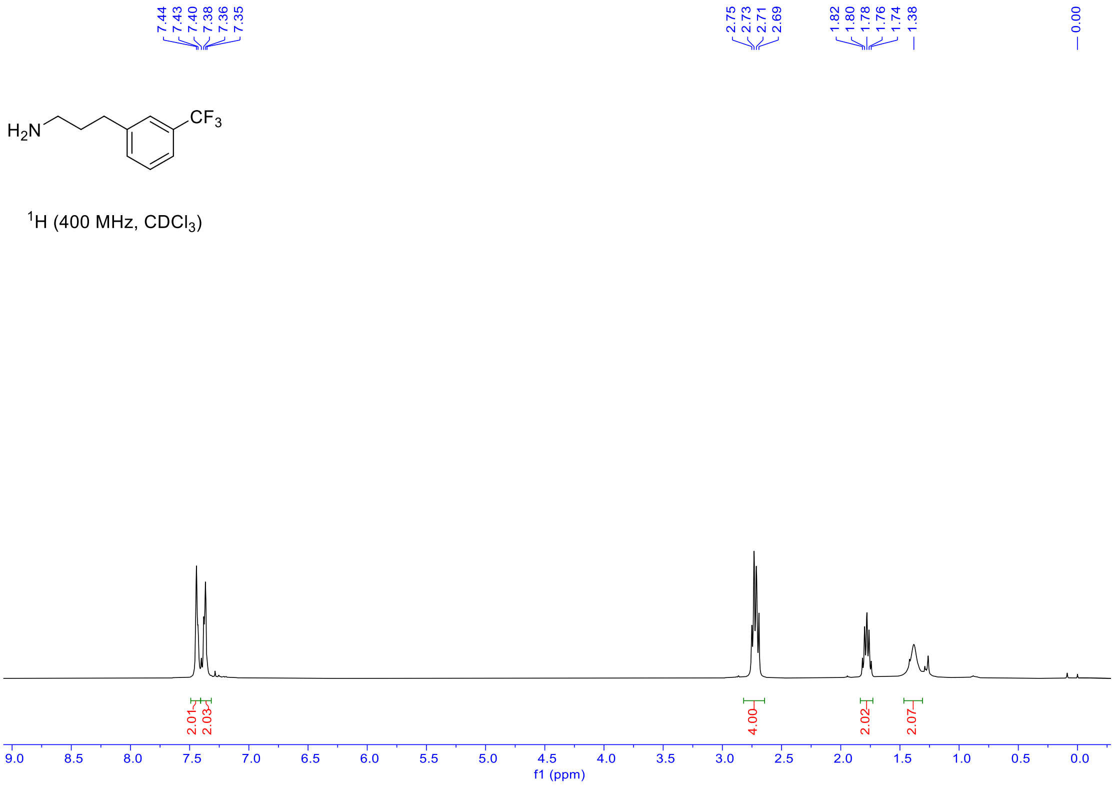

^{13}C NMR spectra (101 MHz, $CDCl_3$)

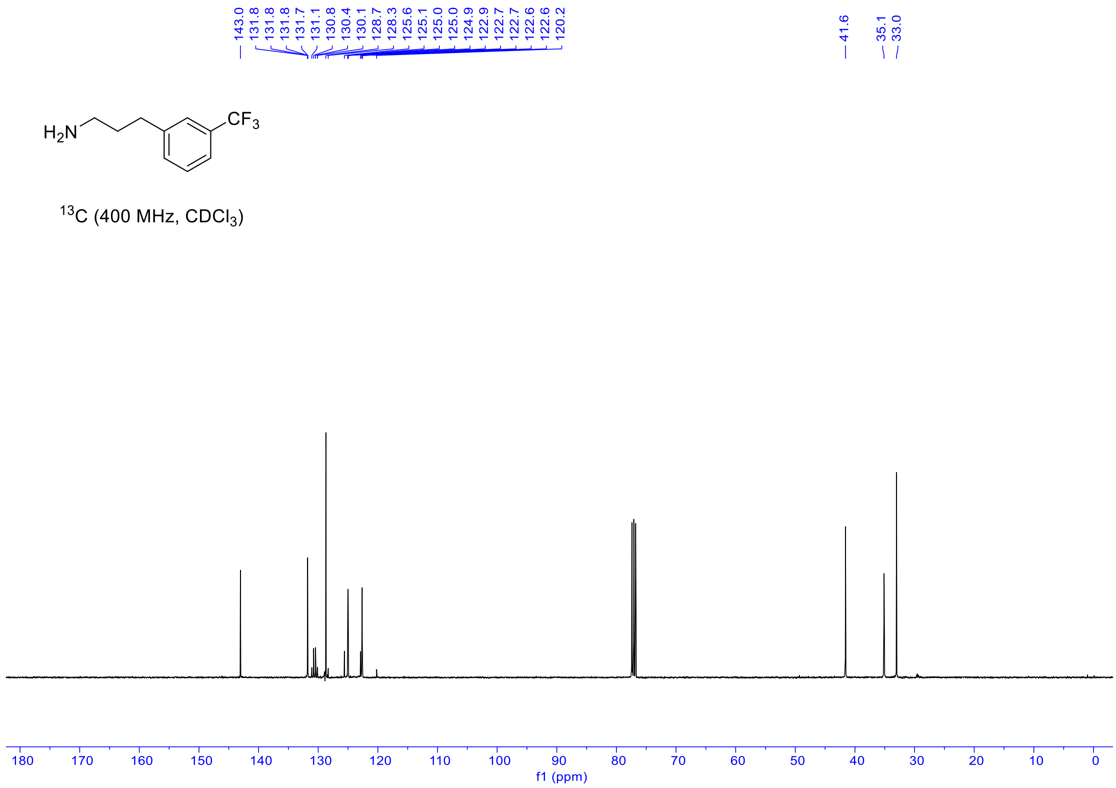

^{19}F NMR spectra (377 MHz, $CDCl_3$)

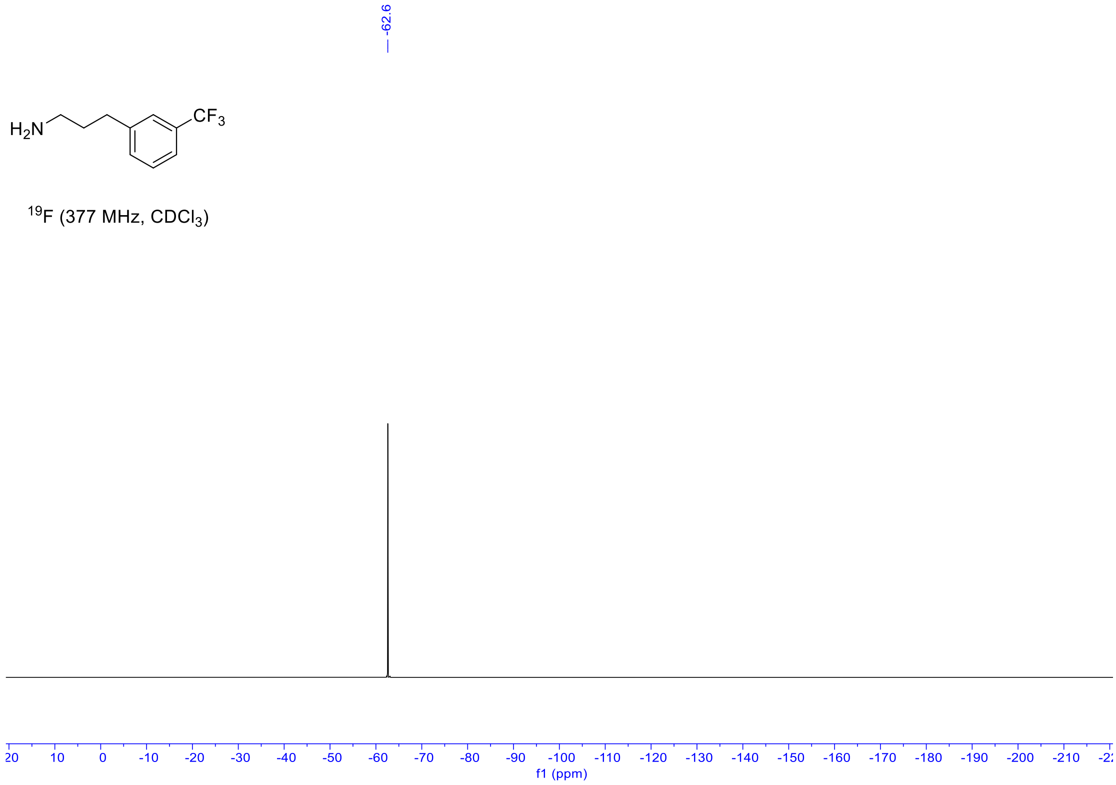

^{1}H NMR spectra of **88** (400 MHz, $CDCl_3$)

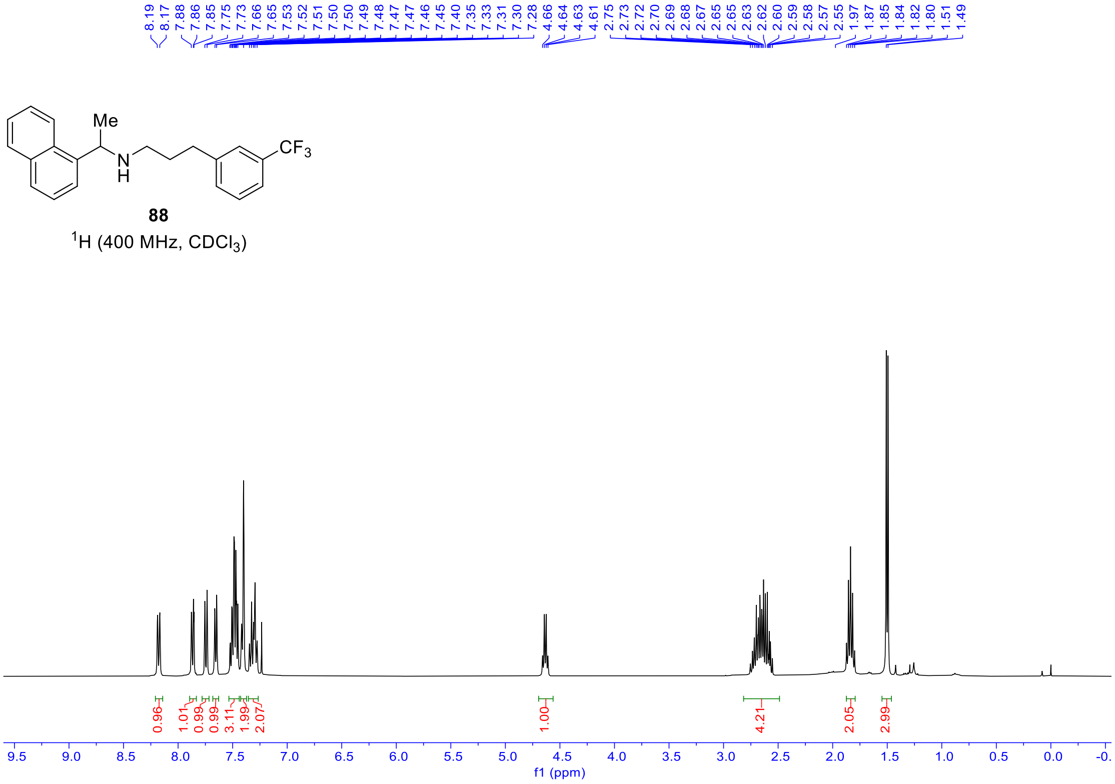

^{13}C NMR spectra of **88** (101 MHz, $CDCl_3$)

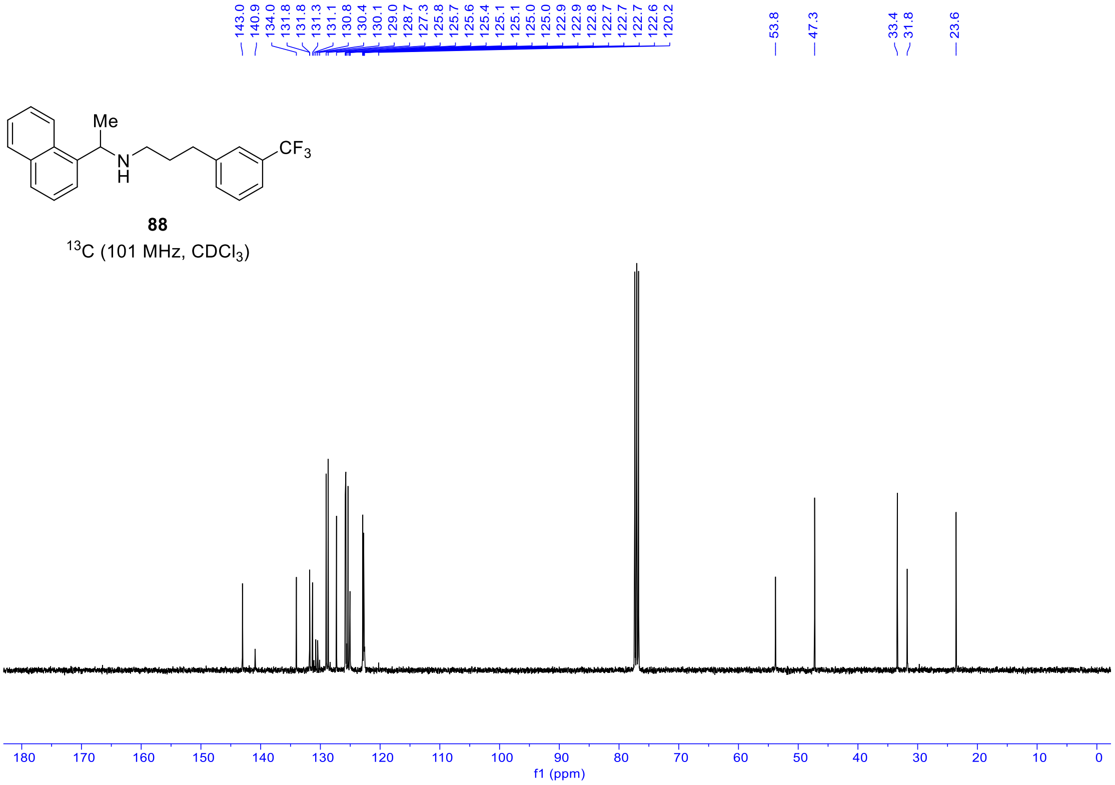

^{19}F NMR spectra of **88** (377 MHz, $CDCl_3$)

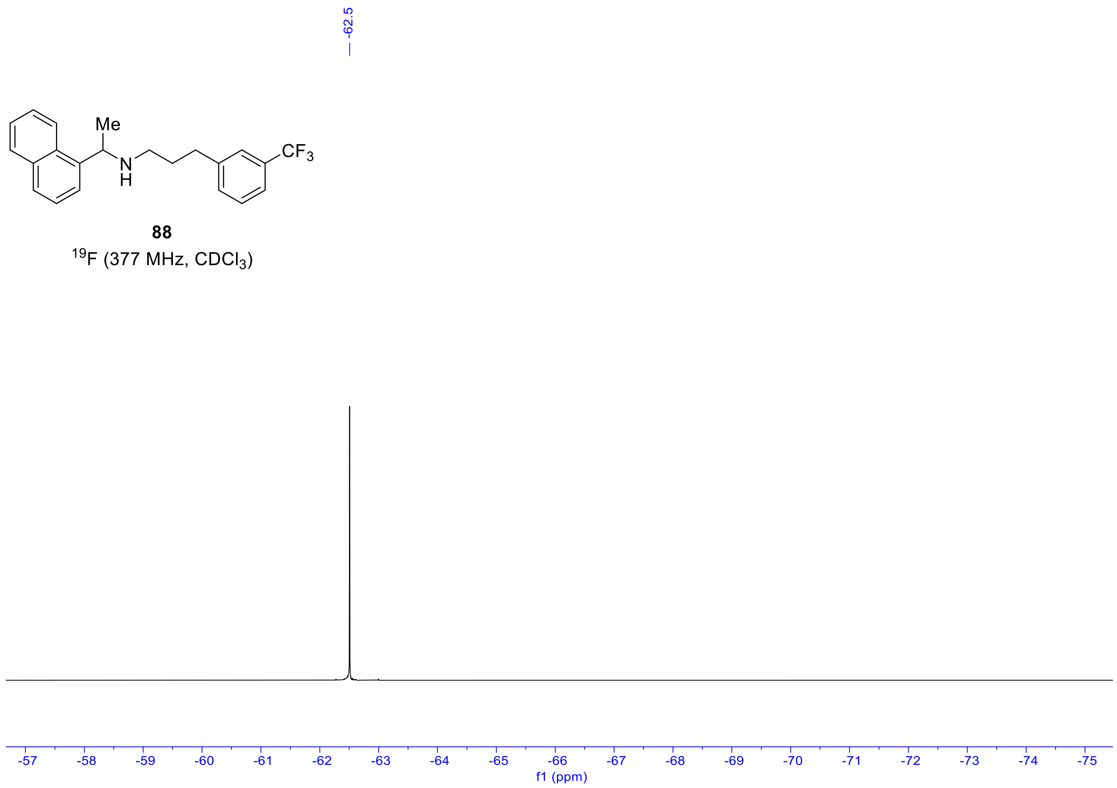

^{1}H NMR spectra (400 MHz, $CDCl_3$)

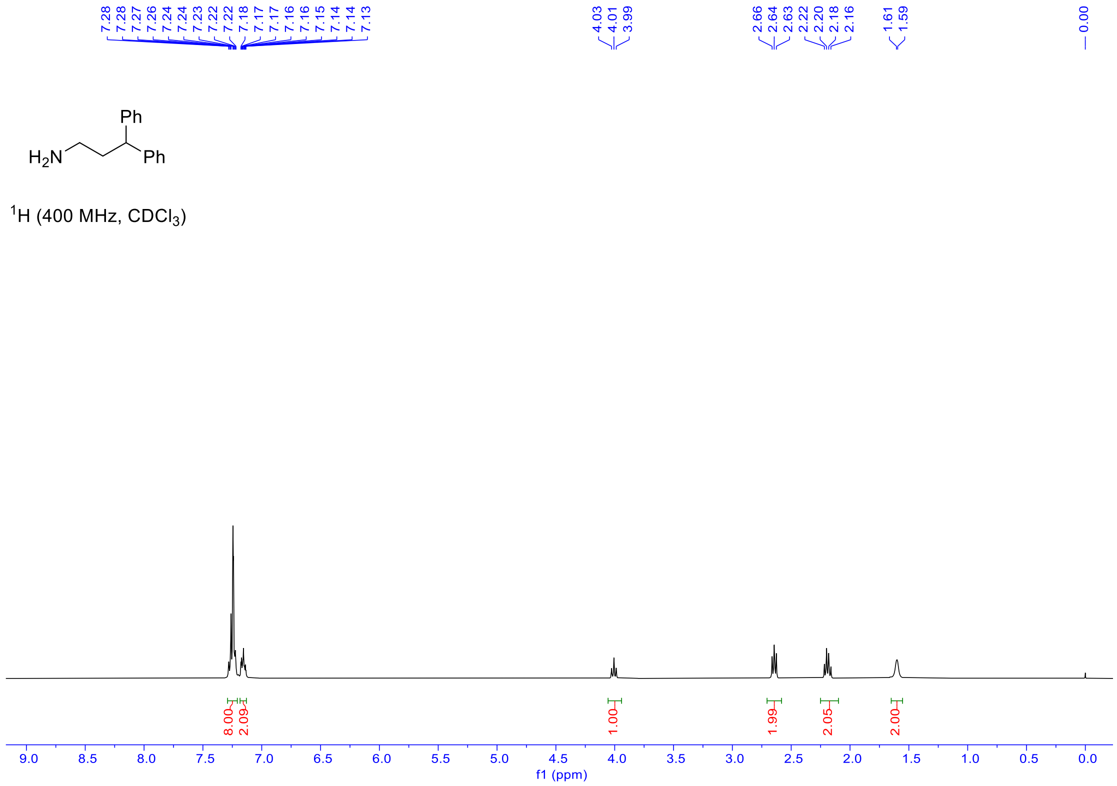

^{13}C NMR spectra (101 MHz, $CDCl_3$)

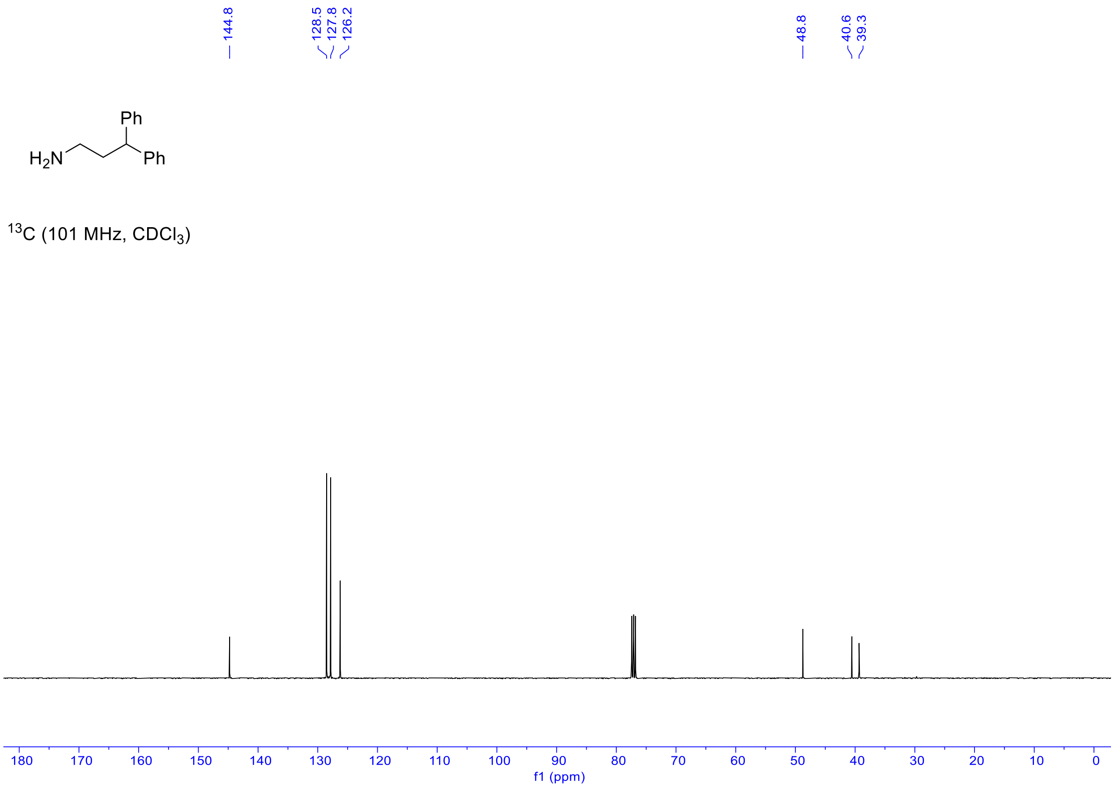

^{1}H NMR spectra of **89** (400 MHz, $CDCl_3$)

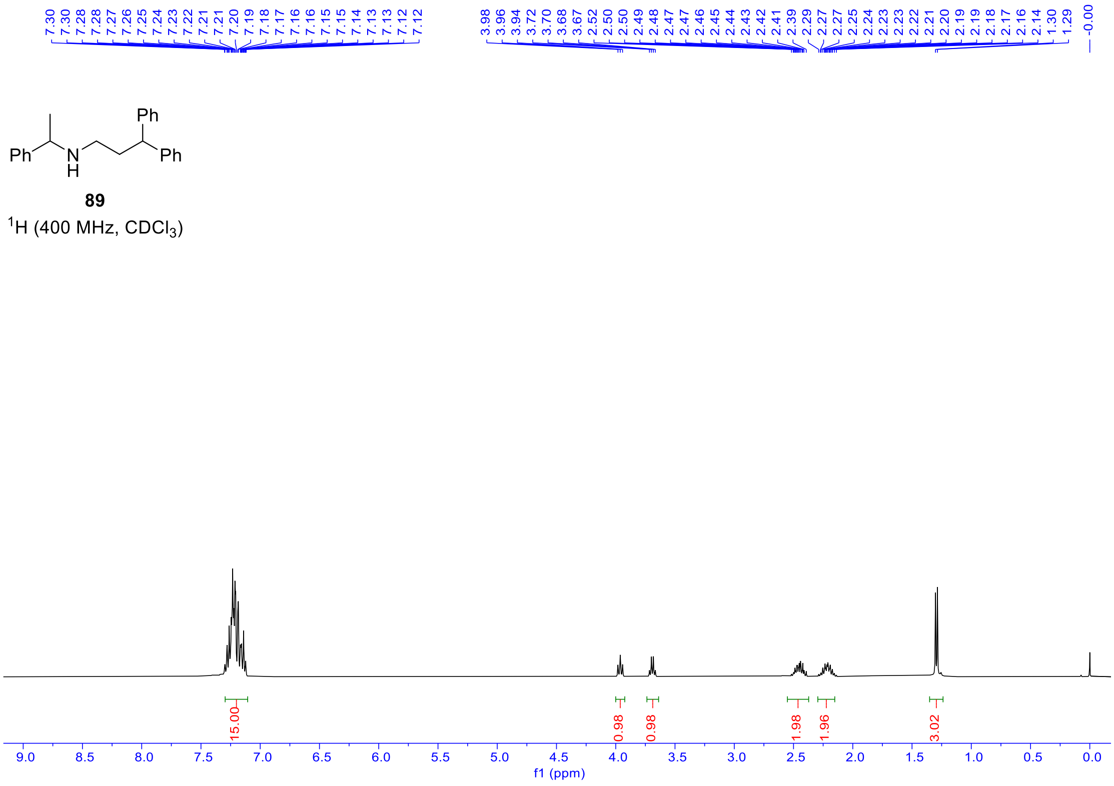

^{13}C NMR spectra of **89** (101 MHz, $CDCl_3$)

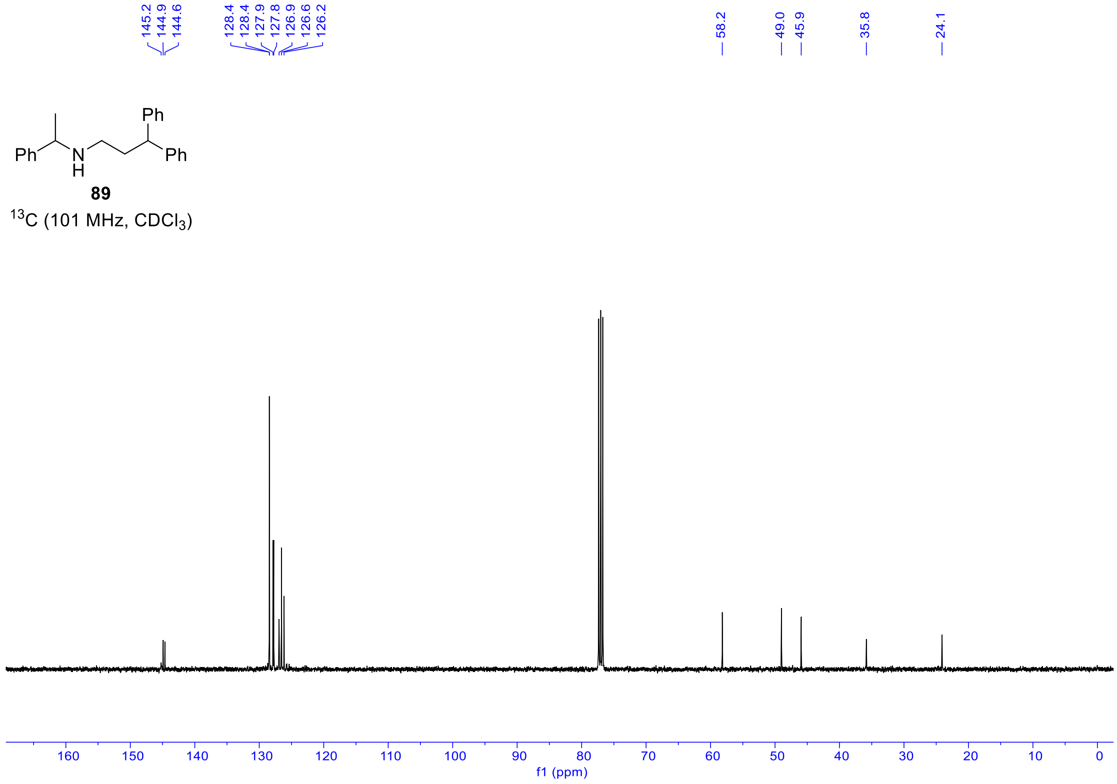

^{1}H NMR spectra (400 MHz, $CDCl_3$)

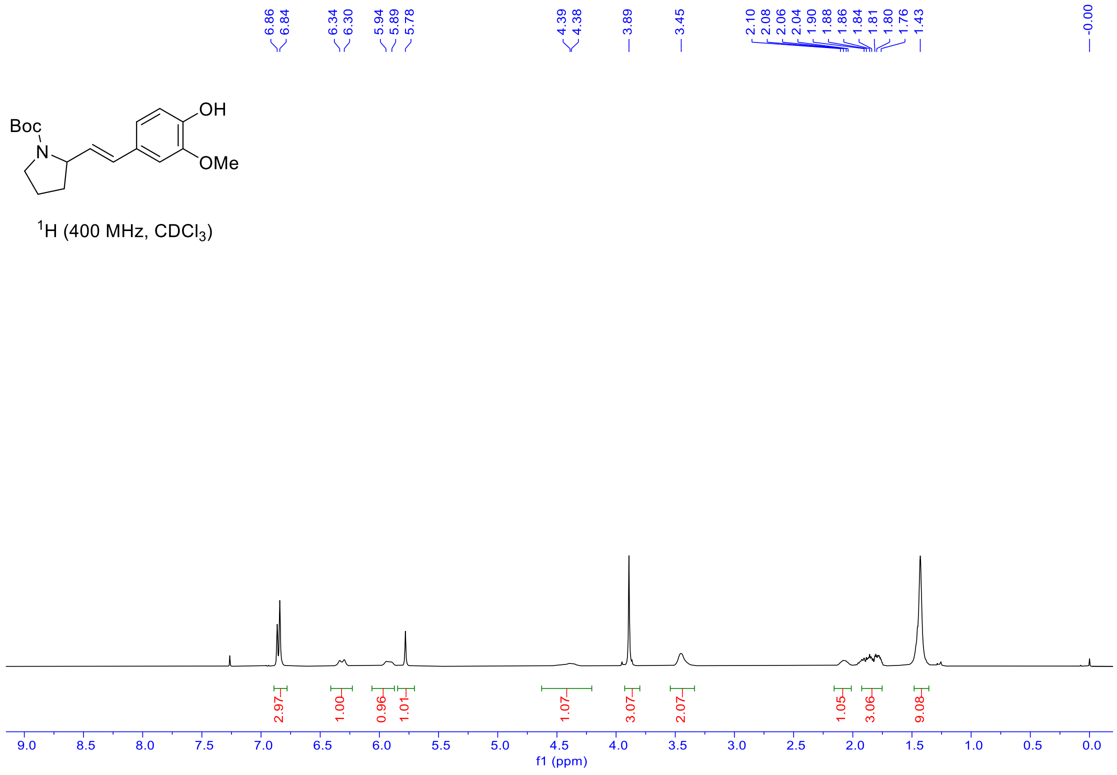

^{13}C NMR spectra (101 MHz, $CDCl_3$)

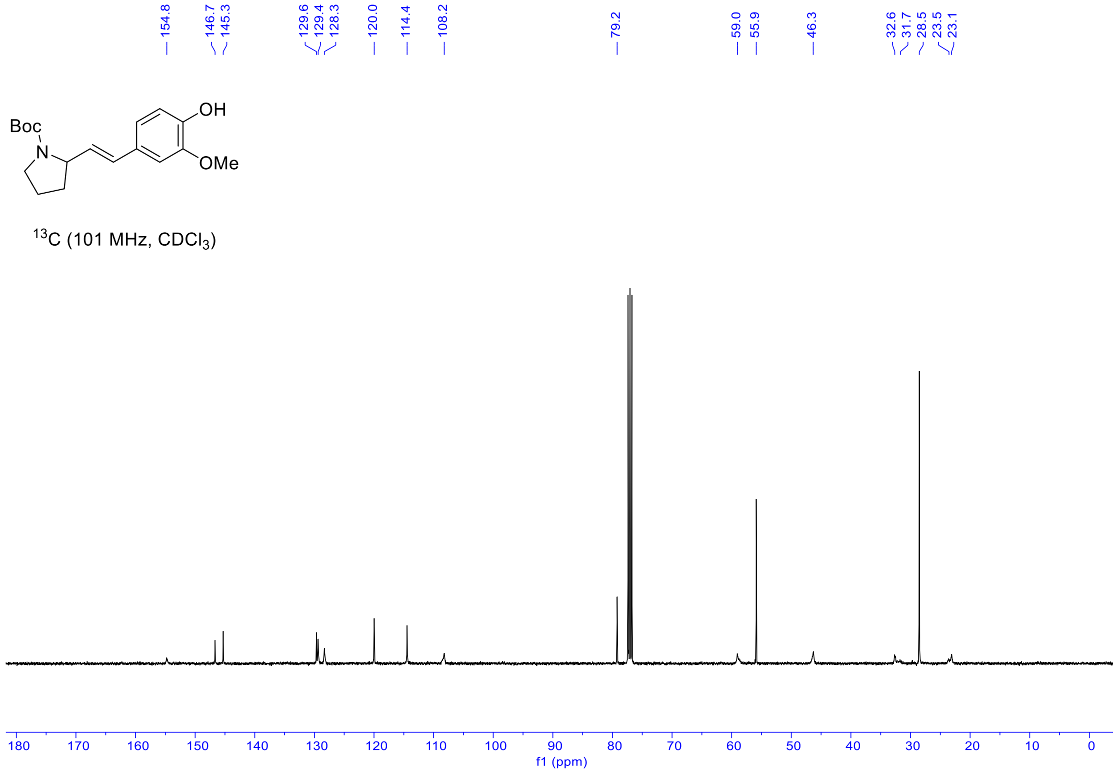

^{1}H NMR spectra of **90** (400 MHz, $CDCl_3$)

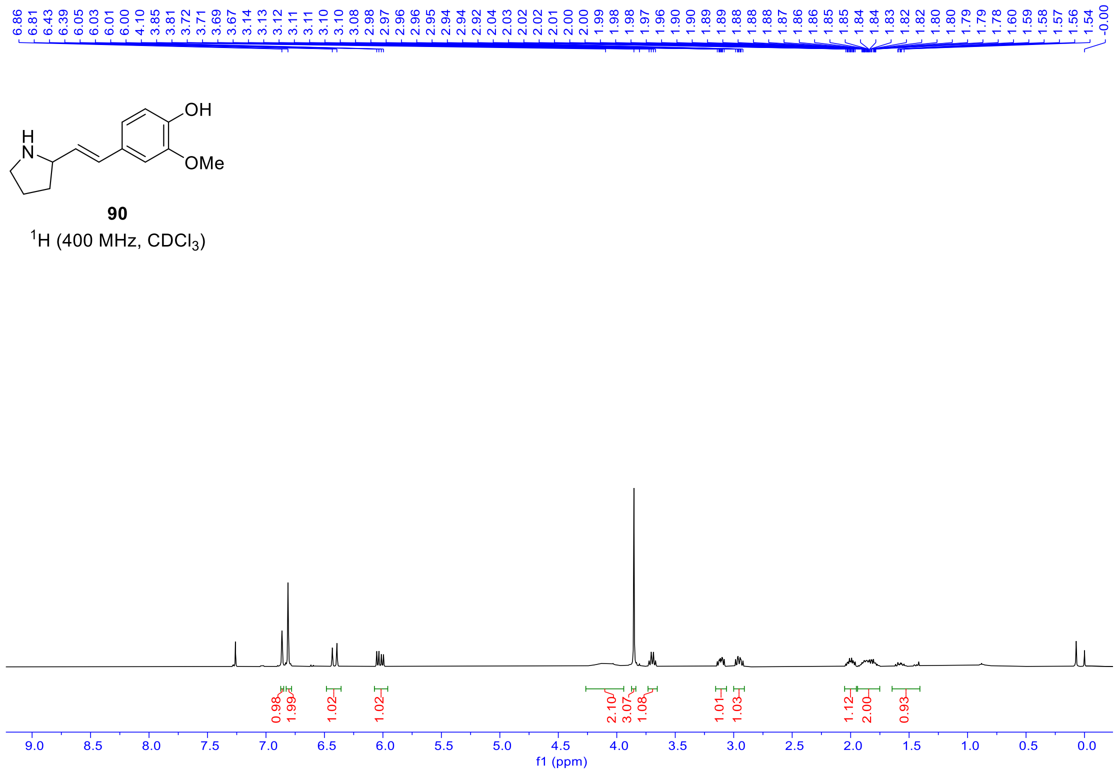

^{13}C NMR spectra of **90** (101 MHz, $CDCl_3$)

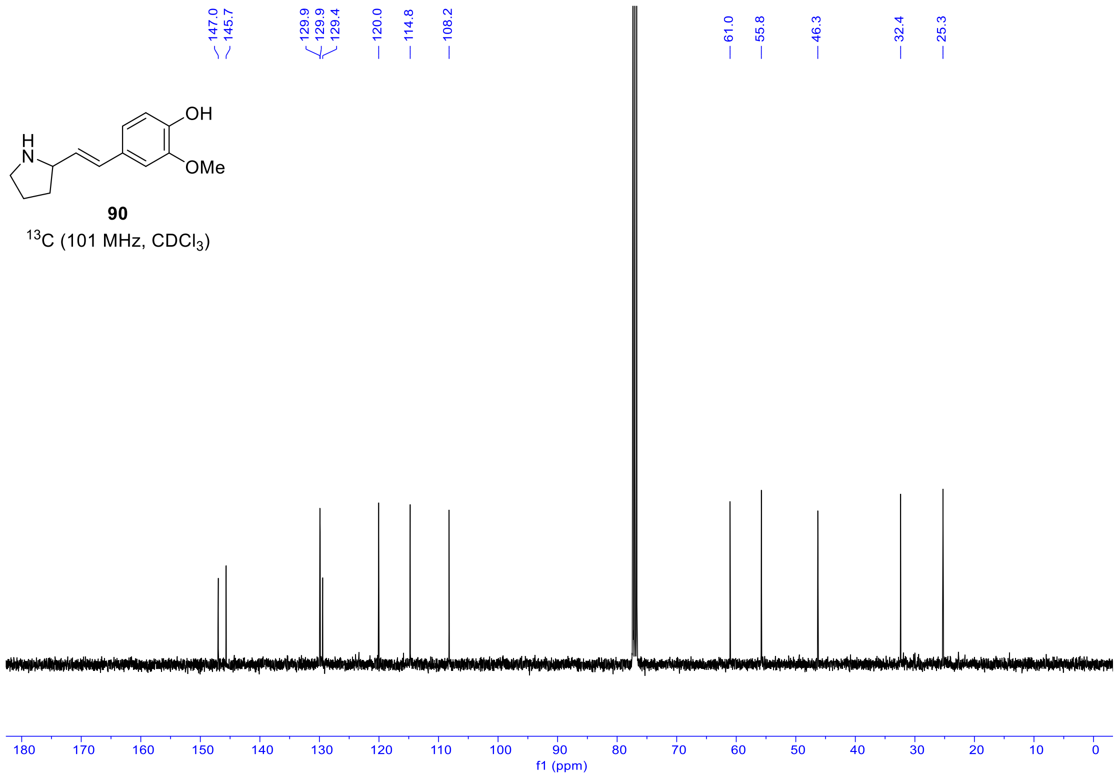

Supplement: SC-016-D5SC04589D-s001 [file SC-016-D5SC04589D-s001.pdf]
